# A Biomimetic Synthetic Strategy Can Provide Keratan Sulfate I and II Oligosaccharides with Diverse Fucosylation and Sulfation Patterns

**DOI:** 10.1021/jacs.4c00363

**Published:** 2024-03-18

**Authors:** Yunfei Wu, Gerlof P. Bosman, Digantkumar Chapla, Chin Huang, Kelley W. Moremen, Robert P. de Vries, Geert-Jan Boons

**Affiliations:** † Department of Chemical Biology and Drug Discovery, Utrecht Institute for Pharmaceutical Sciences, and Bijvoet Center for Biomolecular Research, Utrecht University, Universiteitsweg 99, 3584 CG Utrecht, The Netherlands; ‡ Complex Carbohydrate Research Center, University of Georgia, 315 Riverbend Road, Athens, Georgia 30602, United States; § Department of Biochemistry and Molecular Biology, University of Georgia, Athens, Georgia 30602, United States; ∥ Department of Chemistry, University of Georgia, Athens, Georgia 30602, United States

## Abstract

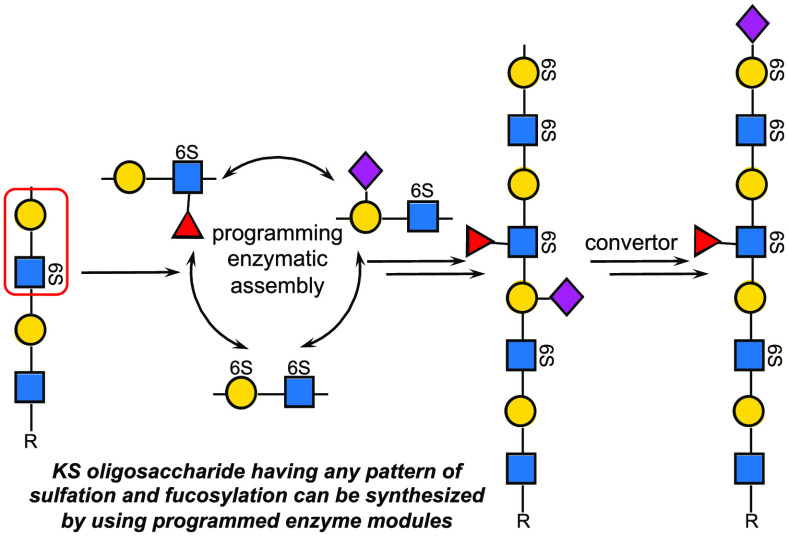

Keratan sulfate (KS) is a proteoglycan that is widely expressed in the extracellular matrix of various tissue types, where it performs multiple biological functions. KS is the least understood proteoglycan, which in part is due to a lack of panels of well-defined KS oligosaccharides that are needed for structure-binding studies, as analytical standards, to examine substrate specificities of keratinases, and for drug development. Here, we report a biomimetic approach that makes it possible to install, in a regioselective manner, sulfates and fucosides on oligo-*N*-acetyllactosamine (LacNAc) chains to provide any structural element of KS by using specific enzyme modules. It is based on the observation that α1,3-fucosides, α2,6-sialosides and C-6 sulfation of galactose (Gal6S) are mutually exclusive and cannot occur on the same LacNAc moiety. As a result, the pattern of sulfation on galactosides can be controlled by installing α1,3-fucosides or α2,6-sialosides to temporarily block certain LacNAc moieties from sulfation by keratan sulfate galactose 6-sulfotransferase (CHST1). The patterns of α1,3-fucosylation and α2,6-sialylation can be controlled by exploiting the mutual exclusivity of these modifications, which in turn controls the sites of sulfation by CHST1. Late-stage treatment with a fucosidase or sialidase to remove blocking fucosides or sialosides provides selectively sulfated KS oligosaccharides. These treatments also unmasked specific galactosides for further modification by CHST1. To showcase the potential of the enzymatic strategy, we have prepared a range of poly-LacNAc derivatives having different patterns of fucosylation and sulfation and several *N*-glycans decorated by specific arrangements of sulfates.

## Introduction

**Figure 1:**
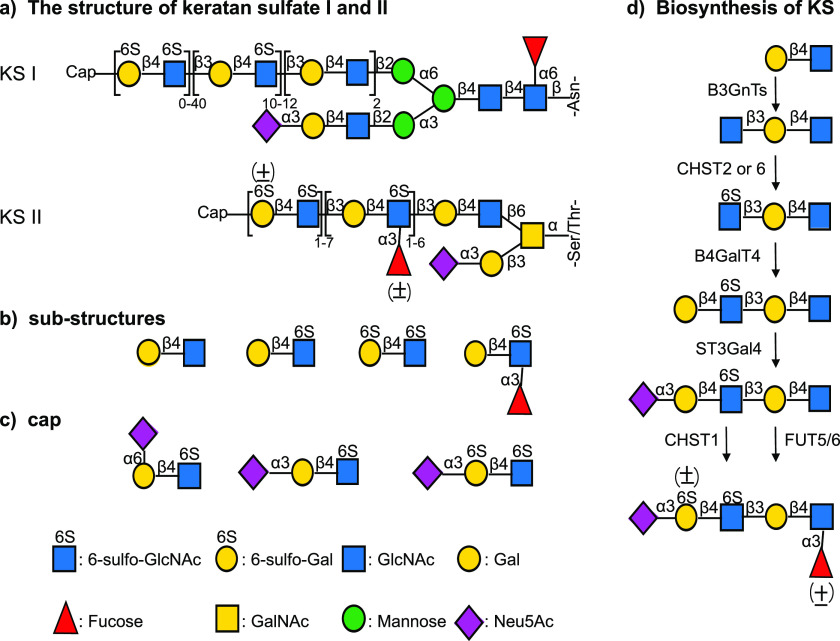
Keratan sulfate (KS) structure and biosynthesis. (a) KS are *N*- and *O*- glycans having a poly-LacNAc chain modified by sulfates and fucosides. (b) The poly-LacNAc backbone is composed of four different substructures that can be assembled in different orders, creating substantial structural diversity. (c) The terminal epitope of KS is usually a sialylated LacNAc moiety having various types of sulfation. (d) The biosynthesis of the poly-LacNAc chain of KS involves sulfation of terminal GlcNAc moieties by CHST2 to give GlcNAc6S that can be further extended by B4GalT4. CHST1 can sulfate internal Gal moieties and has a preference for residues that are flanked by a GlcNAc6S residue.

Keratan sulfate (KS) is a highly complex proteoglycan abundantly expressed in extracellular matrix of cornea, bone, cartilage, brain, and on the surface of epithelial cells.^1−4^ Corneal keratan sulfate (KS-I) is attached to *N*-linked glycans of several core proteins, whereas in cartilage it is attached to *O*-linked glycans via a core-2 structure (KS-II) (Figure 1a). A third type of KS that is mainly found in the brain is through a mannoside linked to the side chain of serine (KS-III).^3−7^ One of the antennae of the *N*- and *O*-glycans is extended by a poly-*N*-acetyl-lactosamine (poly-LacNAc) chain that is modified by sulfate esters at the C-6 positions of galactoside (Gal) and *N*-acetylglucosamine (GlcNAc) residues. The LacNAc backbone of KS-II can also be α1,3-fucosylated and because of biosynthetic restrictions, four different repeating units can be identified (Figure 1b). These can be assembled in different orders, resulting in considerable structural diversity. Furthermore, the termini of KS can be capped by α2,3- and α2,6-linked sialosides which in combination with sulfation give various terminal epitopes further increasing the structural diversity (Figure 1c).^8,9^

KS is involved in a range of biological processes such as cornea transparency, embryonic development, wound healing, cell adhesion, and migration.^2,3,10^ It also regulates inflammation and potentially can be exploited for the treatment of inflammatory conditions such as rheumatoid arthritis, asthma, and chronic obstructive pulmonary disease.^2,11^ Dysregulation of KS biosynthesis has been associated with macular degeneration and keratoconus,^1,10^ amyotrophic lateral sclerosis,^12^ Alzheimer’s disease,^13,14^ and mucopolysaccharidosis IV,^15^ and is associated with a poor prognosis of various cancers.^16−20^

The biosynthesis of KS involves the assembly of a poly-LacNAc chains by β(1,3)-N-acetylglucosaminyltransferases (B3GnT) and β(1,4)-galactosyl transferases (B4GalT) in combination with UDP-GlcNAc and UDP-Gal, respectively (Figure 1d).^21^ During the assembly of this chain, C-6 hydroxyls of terminal GlcNAc residues can be sulfated by GlcNAc-6-*O*-sulfotransferases 2 and 6 (CHST2 and 6). The enzymes B4GalT4 can attach a β1,4-linked galactose to a 6-sulfo-GlcNAc residue, whereas B4GalT1 and B4GalT7 can extend unmodified GlcNAc moieties. After the assembly of the poly-LacNAc chain, the terminal galactoside can be modified by α2,3- or α2,6-linked sialoside by β-galactoside α-2,3-sialyltransferase 4 (ST3Gal4) and β-galactoside-α-2,6-sialyltransferase 1 (ST6Gal1), respectively. C-6 hydroxyls of galactosides can be sulfated by keratan sulfate galactose 6-sulfotransferase (KSGal6ST, CHST1)^22^ or chondroitin sulfotransferase-1 (CST1).^23^ α-1,3-Fucosyltransferases (FUTs) can further modify the sulfated poly-LacNAc chain to install Lewis^x^ (Le^x^) or sulfo-Le^x^ moieties epitopes.^24,25^

The KS biosynthetic enzymes cooperate to construct specific epitopes that can recruit glycan binding proteins to mediate various biological processes. For example, CHST1 modifies only internal galactosides of a poly-LacNAc chain; however, it greatly prefers galactosides that are neighbored by a GlcNAc6S residue.^4,23^ The presence of a 2,3-linked sialoside further modulates the site of sulfation, and a galactoside that is positioned in between an α2,3-Neu5Ac and GlcNAc6S is a preferred substrate for this sulfotransferase.^23,26^ Furthermore, fucosylation of GlcNAc to give Lewis^x^ (Le^x^) blocks the activity of ST6Gal1 whereas an α2,6-sialoside prevents fucosylation by FUT5 and FUT6.^27,28^ There is an interplay between sulfation of Gal by CHST1 and fucosylation of GlcNAc by fucosyl transferases.^24,27^ In particular, fucosylated LacNAc moieties cannot be sulfated by CHST1. Furthermore, sulfation of Gal gives an epitope (Gal6S-1,4-GlcNAc6S) that cannot be fucosylated by FUT5 or FUT6. Due to these biosynthetic restrictions, the poly-LacNAc chain can be composed of four different substructures (Figure 1b) and capped by three common terminal epitopes (Figure 1c).

Despite their importance, the preparation of KS oligosaccharides has received little attention. Well-defined KS oligosaccharides are, however, needed to examine ligand requirements of glycan binding proteins as standards for analytical method development and as probes to investigate biosynthetic pathways. Chemical approaches, which require time-consuming protecting manipulations and glycosylations, have only given relatively small structural motifs such as di- and tetrasaccharides.^29−33^ Sulfated LacNAc derivatives have been chemically synthesized that could be enzymatically fucosylated and sialylated, but this approach has only yielded relatively small structural elements.^34^ In another chemoenzymatic approach, a linker modified GlcNAc derivative was extended into a di- and trisaccharide by treatment with human B4GalT1 and B3GnT from *Helicobacter pylori*, respectively followed by selective chemical sulfation of C-6 hydroxyls to give di- and tri-*O*-sulfated products that were further enzymatic elongated by B4GalT1 and B3GnT to install additional LacNAc moieties.^35^ To prepare larger KS oligosaccharides, chemically synthesized oxazolines have been linked by *trans*-glycosylation using a mutant keratanase II.^36,37^ The substrate preferences of recombinant sulfotransferases have been employed to prepare several KS oligosaccharides,^26^ however, it does not provide strict control over the exact positions of sulfate esters, and therefore cannot provide any possible sulfation patterns and may require tedious purification protocols. Currently, no synthetic methodology is available that can provide large panels of KS-I and KS-II oligosaccharides.

**Figure 2:**
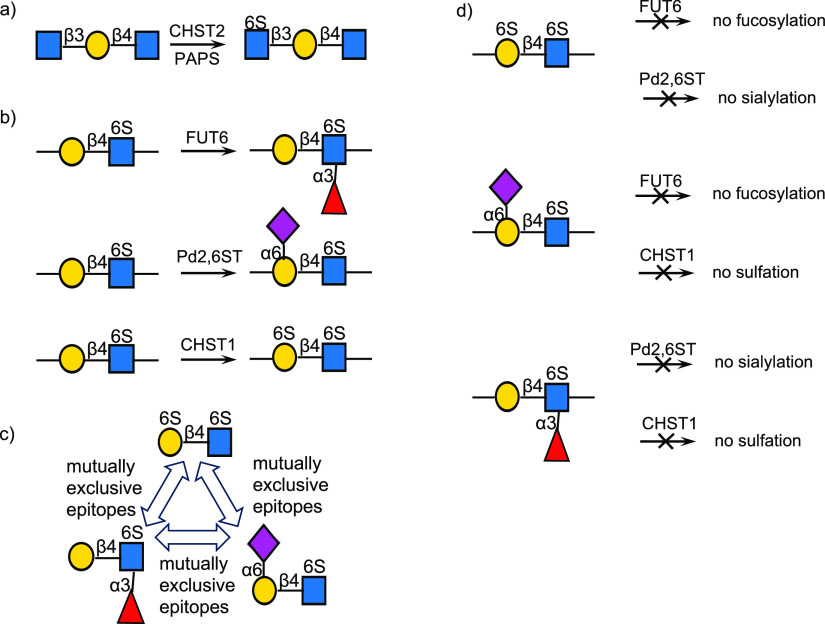
Biomimetic synthesis of KS oligosaccharides exploiting inherent substrate specificities of sulfo-, sialyl-, and fucosyl-transferases. (a) CHST2 only modifies terminal GlcNAc moieties. (b) FUT6, Pd2,6ST and CHST1 can modify Galβ(1,4)GlcNAc6S moieties. (c) Certain modifications are mutually exclusive and cannot occur at the same LacNAc moiety. (d) Transformations that are due to biochemical restrictions cannot occur.

Here, we report a biomimetic approach that makes it possible to install sulfates and fucosides in a regioselective manner at an oligo-LacNAc chains to provide any structural element observed in KS-I and KS-II. It exploits that the sulfotransferase CHST2 only modifies terminal GlcNAc moieties to give GlcNAc6S (Figure 2a).^38^ The latter residue can then be extended by an β1,4-galactoside using recombinant B4GalT4 and UDP-Gal. Furthermore, we found that FUT6, the bacterial α2,6-sialyltransferases Pd2,6ST and the sulfotransferase CHST1 can readily accept 6-sulfo-LacNAc as a substrate to give the corresponding products (Figure 2b). A critical component of the biomimetic strategy was the recognition that α1,3-fucosides, α2,6-sialosides, and Gal6S are mutually exclusive (Figure 2c) and cannot occur on the same LacNAc moiety providing opportunities to install fucosides and sulfates at specific galactosyl residues. Thus, it was expected that structures such Gal6S-1,4-GlcNAc6S cannot be modified by α1,3-fucosyl transferases such as FUT6 and α2,6-sialyltransferases such as Pd2,6ST. Furthermore, the Lewis^x^ (Le^x^) or 6-sulfo-Le^x^ moiety should be resistant to α2,6-sialylation by, for example, Pd2,6ST and sulfation by CHTS1. Finally, it was the expectation that an α2,6-sialyl-LacNAc moiety cannot be fucosylated by fucosyltransferases such as FUT6 and obviously the sialoside also blocks sulfation by CHST1. Based on these considerations, two strategies were explored to control the pattern of sulfation at Gal by installing α1,3-fucosides or α2,6-sialosides to temporarily block certain LacNAc moieties from sulfation by CHST1. The pattern of α1,3-fucosylation and α2,6-sialylation was controlled by the mutual exclusivity of these modifications, which in turn controls the sites of sulfation by CHST1. Late-stage treatment with a fucosidase or sialidase to remove blocking fucosides or sialosides provides selectively sulfated KS oligosaccharides. These treatments also unmasked specific galactosides for further controlled modification by CHST1. The methodology makes it possible to prepare any structural motif found in KS-I and KS-II by employing specific enzyme modules. To showcase its potential, we prepared a range of poly-LacNAc derivatives and *N*-glycans having various patterns of fucosylation and sulfation.

## Results and Discussion

### Controlling Sulfation at Galactose by α1,3-Fucosylation of GlcNAc

First, we explored whether α1,3-fucosylation can control the sites of sulfation of an oligo-LacNAc chain by CHST1. Analytical studies of KS-II have indicated that α1,3-fucosylation of GlcNAc (Le^x^) and 6-sulfation of galactose do not occur at the same LacNAc moiety, and thus are mutually exclusive.^4^ Thus, we anticipated that α1,3-fucosylation of LacNAc should block the action of CHST1 thereby providing a means to regioselectively install sulfates at Gal moieties.

**Scheme 1:**
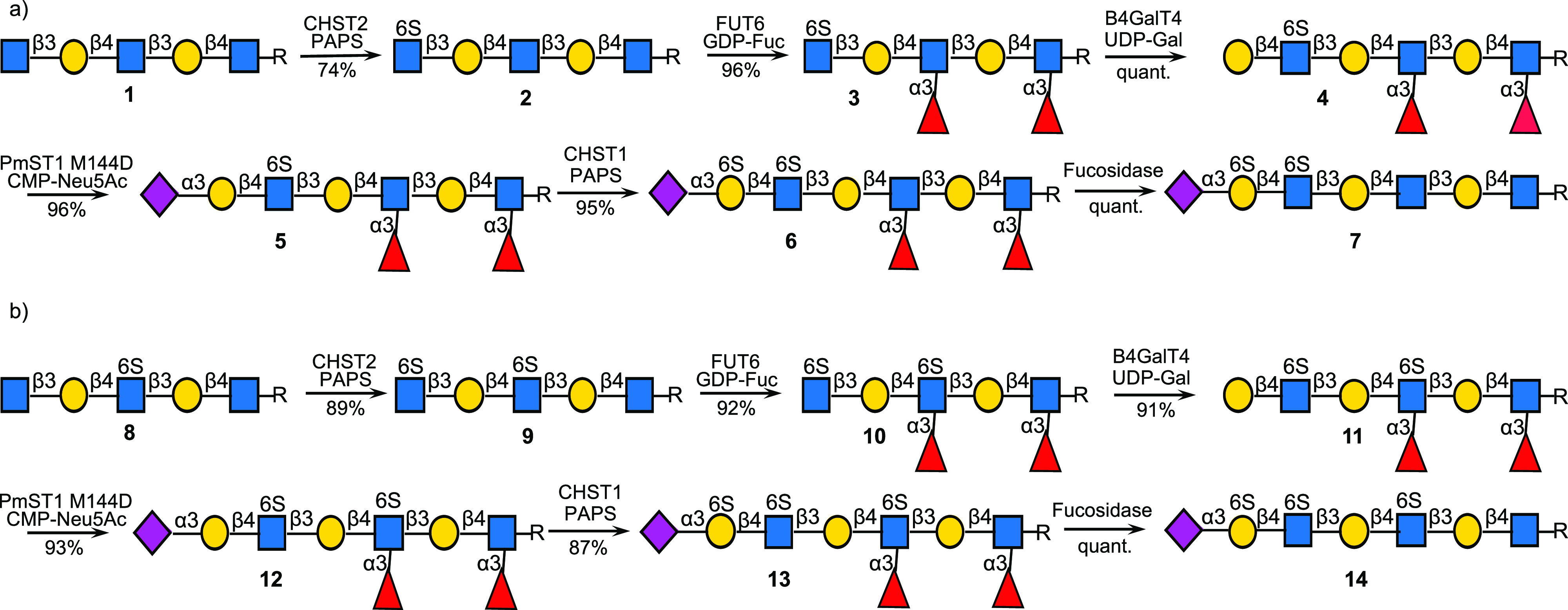
Enzymatic Synthesis of Selectively Sulfated KS Oligosaccharides by Exploiting 1,3-Fucosylation as a Blocking Group for Sulfation of Gal by CHST1 (R = (CH_2_)_5_NHCbz)

Pentasaccharide **1** was prepared starting from a chemically synthesized LacNAc derivative having a benzyloxycarbonyl (CBz) protected amino pentenyl linker at the anomeric center by consecutive actions of B3GnT2, B4GalT1, and B3GnT2 (Scheme S1). Compound **1** has a terminal GlcNAc moiety and thus could be selectively sulfated by the sulfotransferase CHST2 in the presence of the sulfate donor PAPS resulting in the formation of **2** (Scheme 1a). The latter compound was modified by FUT6, which only modifies internal GlcNAc moieties, and as anticipated, it resulted in the selective formation of heptasaccharides **3** which has two Le^x^ moieties and a terminal GlcNAc residue having a sulfate at C-6. The later residue could be extended by a β1,4-linked galactoside by treatment with B4GalT4 and UDP-Gal to provide **4** which was subjected to the prokaryotic sialyltransferase PmST1M144D^39^ and CMP-Neu5Ac to install an α2,3-sialoside resulting in the formation of glycan **5**. As expected, treatment of **5** with CHST1 and PAPS resulted only in sulfation of Gal flanked by the sialoside and GlcNAc6S to give the nonasaccharide **6**. The other Gal moieties are blocked from sulfation by the fucosides at the neighboring GlcNAc moiety. Several other fucosylated structures were prepared, and these were also resistant to sulfation by CHST1 (Scheme S2) confirming that Le^x^ and sulfo-Le^x^ are not substrates for this enzyme. KS-I oligosaccharide **7** could be prepared in a quantity of 1.0 mg by treatment of **6** with the fucosidase of the human gut symbiont *Ruminococcus gnavus*.^40^ This fucosidase can hydrolyze α1,3/4 fucosides of Lewis^x^ and Lewis^a^, respectively and can also operate on their sialic acid counter parts (sialyl Lewis^x/a^ epitopes).^40^ The facile hydrolysis of the two fucosides of **6** demonstrates that this fucosidase also accepts sulfated Le^x^ moieties as substrates. When the sequence of enzymatic transformations was changed and **5** was treated with the fucosidase of *R. gnavus* and then CHST1, a mixture of compounds was obtained (see Scheme S3). Size exclusion column chromatography over Bio-Gel P2 or P6 was employed to purify intermediates and final compounds which were fully characterized by homo- and heteronuclear two-dimensional NMR experiments and by LC-MS. Positions of sulfates were confirmed by chemical shift differences of relevant C-6 carbon and H6a,b protons (Figure S1a–c).

Next, we examined whether the methodology can be employed to prepare higher sulfate derivatives (Scheme 1b). Thus, compound **9**, which was prepared by stepwise sulfation of terminal GlcNAc moieties by CHST2 and PAPS followed by galactosylation by B4GalT4, was exposed to FUT6 and GDP-Fuc to give difucoside **10**. Thus, this transformation showed that LacNAc as well as sulfated LacNAc moieties can readily be fucosylated by FUT6. The latter compound was galactosylated by B4GalT4 to give **11** which was sialylated using PmST1M144D^39^ and CMP-Neu5Ac to provide **12**. As expected, in this case, only one of the galactosides of **12** was sulfated by CHST1 to give **13** (1.9 mg). Treatment of the latter compound with the fucosidase of *R. gnavus* gave the target compound **14**. Thus, one sequence of enzymatic transformations resulted in a range of biologically relevant KS-I and KS-II oligosaccharides (**11**–**14**).

### Blocking Sulfation of Galactose by α2,6-Sialylation

**Scheme 2:**
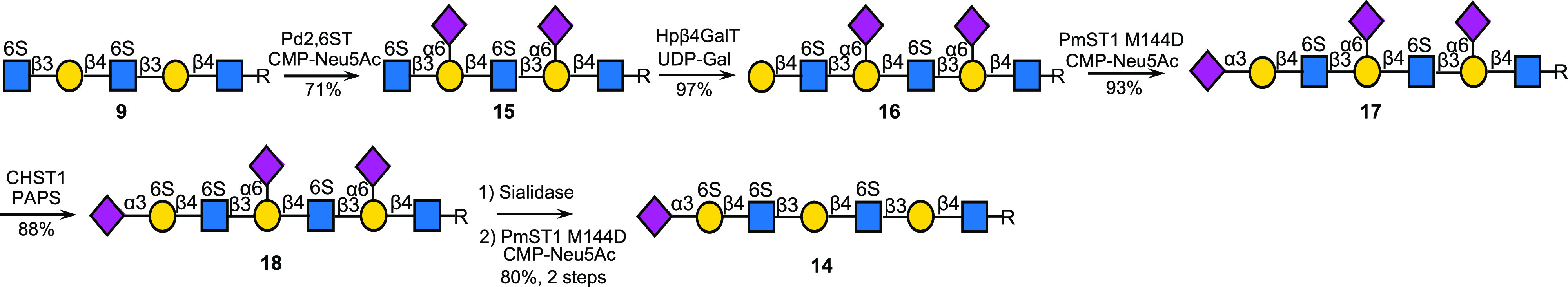
Enzymatic Synthesis of Selectively Sulfated KS Oligosaccharides by 2,6-Sialylation of Gal Moieties To Block Sulfation by CHST1 (R = (CH_2_)_5_NHCbz)

Next, we explored whether sialylation of the C-6 positions of galactosides can be employed to control sulfation at specific galactosides. The bacterial α2,6-sialyltransferase from *Photobacterium damselae* (Pd2,6ST)^41,42^ can sialylate terminal as well as internal galactosides and thus we anticipated that its activity can be exploited to block certain sites from sulfation by CHST1. To implement this strategy, it was critical to explore whether Pd2,6ST can use Gal-1,4-GlcNAc6S moieties as the substrate. Thus, compound **9** was subjected to Pd2,6ST in the presence of CMP-Neu5Ac, which gratifyingly provided compound **15** demonstrating the enzyme is compatible with substrates having sulfates at a neighboring GlcNAc moiety (Scheme 2). Galactosylation of **15** with B4GalT4 and UDP-Gal to produce **16** proceeded very sluggishly, and the reaction could not be driven to completion; thus, it appears that the unnatural α2,6-sialoside interferes with substrate recognition by B4GalT4. However, the use of the bacterial galactosyltransferase from *Helicobacter pylori* β4GalT (Hpβ4GalT)^43^ could readily convert **15** into **16**. The latter compound was sialylated by PmST1M144D resulting in the facile formation of trisialoside **17**. Only one galactoside of **17** has a free C-6 hydroxyl, and therefore treatment of this compound with CHST1 in the presence of PAPS resulted in selectively sulfation to yield compound **18** (2.7 mg). The terminal α2,3-sialoside of **17** was critical for sulfation of the neighboring galactoside because CHTS1 does not modify terminal galactosides such as in compound **16**.^23,26^ The promiscuous neuraminidase from *C. perfringens* could remove all sialosides, and exposure of the resulting compound to an α2,3-sialyltransferase (PmST1M144D) gave target compound **14**. The structural integrity and purity of the latter derivative were confirmed by LC-MS and NMR experiments (Figure S2a-c).

### Exploiting the Mutual Exclusivity of α1,3-Fucosylation and α2,6-Sialylation

**Scheme 3:**
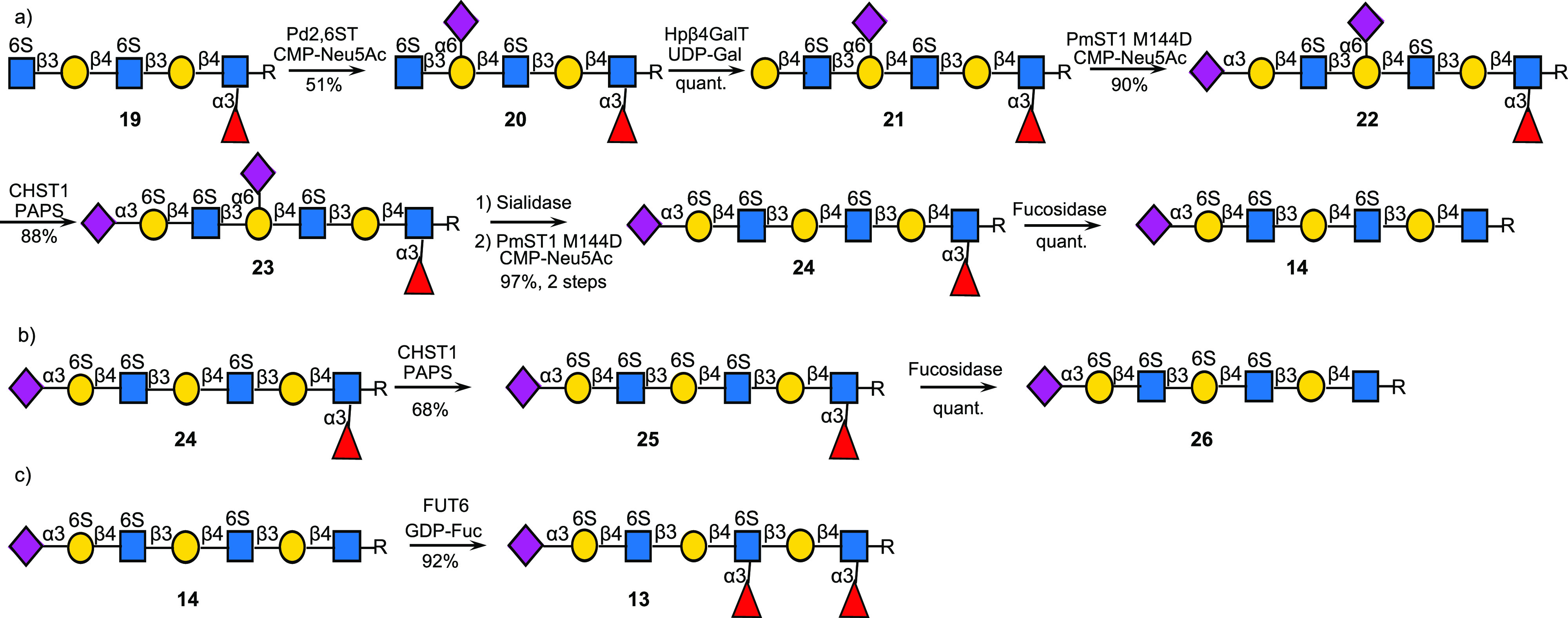
Enzymatic Synthesis of KS Oligosaccharides: (a) Fucosylation Controls the Site of α2,6-Sialylation; α2,6-Sialosides and α1,3-Fucosides Block Sulfation by CHST1; (b) Selective Removal of Sialoside Reveals New Site for Sulfation by CHST1; (c) Sulfation of Gal Blocks Fucosylation by FUT6 To Give KS-II Oligosaccharides (R = (CH_2_)_5_NHCbz)

We exploited the mutual exclusivity of α1,3-fucosylation and α2,6-sialosylation to modify polyLacNAc chains by specific patterns of sulfation and fucosylation. Compound **19** was prepared by fucosylation of spacer modified LacNAc by FUT6 followed by chain extension and sulfation of terminal GlcNAc moieties by CHST2 (see Scheme S6). The Le^x^ moiety of **19** was expected to block modification by Pd2,6ST, and indeed exposure of this compound to the enzyme in the presence of CMP-Neu5Ac resulted in monosialylation of the unmodified LacNAc moiety to give compound **20** (Scheme 3a). A two-step procedure involving galactosylation by Hpβ4GalT (to form **21**) and sialylation by PmST1M144D resulted in the formation of compound **22**. The Le^x^ moiety at the reducing end and α2,6-sialoside of the central LacNAc moiety were expected to block sulfation by CHST1. Therefore, only the terminal α2,3-sialylated LacNAc unit should be modified by this enzyme. Indeed, exposure of **22** to CHST1 and PAPS resulted in the facile formation of **23**. The latter compound was treated with the sialidase of *C. perfringens* to remove all sialosides which was followed by reinstallation of the terminal α2,3-sialoside by PmST1M144D to give compound **24** (1.6 mg). This derivative is attractive to prepare additional KS oligosaccharides, and for example, treatment of **24** with the fucosidase of *R. gnavus* resulted in the formation of **14**.

We anticipated that the fucosyl and sialosyl moieties are orthogonal masking groups allowing further modification of specific LacNAc moieties. For example, removal of the α2,6-sialoside at the central LacNAc moiety of **23** to give **24** made this structural element a substrate for CHST1. Indeed, subjecting **24** to this enzyme in the presence of PAPS resulted in the selective sulfation of the central galactoside to give **25** (Scheme 3b). Treatment of the latter derivative with the fucosidase of *R. gnavus* gave KS-I oligosaccharide **26** (0.6 mg). Interestingly, compound **26** only provided a properly resolved ^1^H NMR spectrum in PBS D_2_O (40 mM, pH 6.5) (Figure S5a,b).

The terminal α2,3-sialyl LacNAc moiety of **14** is modified by a sulfated galactose (Gal6S) that is not a substrate for fucosyltransferases. The other two LacNAc moieties were expected to be the proper substrates for FUT6. Indeed, treatment of **14** with FUT6 in the presence of GDP-Fuc resulted in the formation of difucoside **13** (2.0 mg) (Scheme 3c).

### Controlling α1,3-Fucosylation by α2,6-Sialylation

The strategy described above relies on the introduction of a fucoside at an early stage of the synthesis when only one LacNAc moiety is present. To address this limitation, we exploited the ability of α2,6-sialosides to temporarily block specific LacNAc moieties from fucosylation to install such moieties in a controlled manner. Furthermore, we exploited the orthogonality of the fucosidase and sialidases to unmask one of these residues to reveal a substrate for CHST1 to allow the diversification of specific intermediates.

**Scheme 4:**
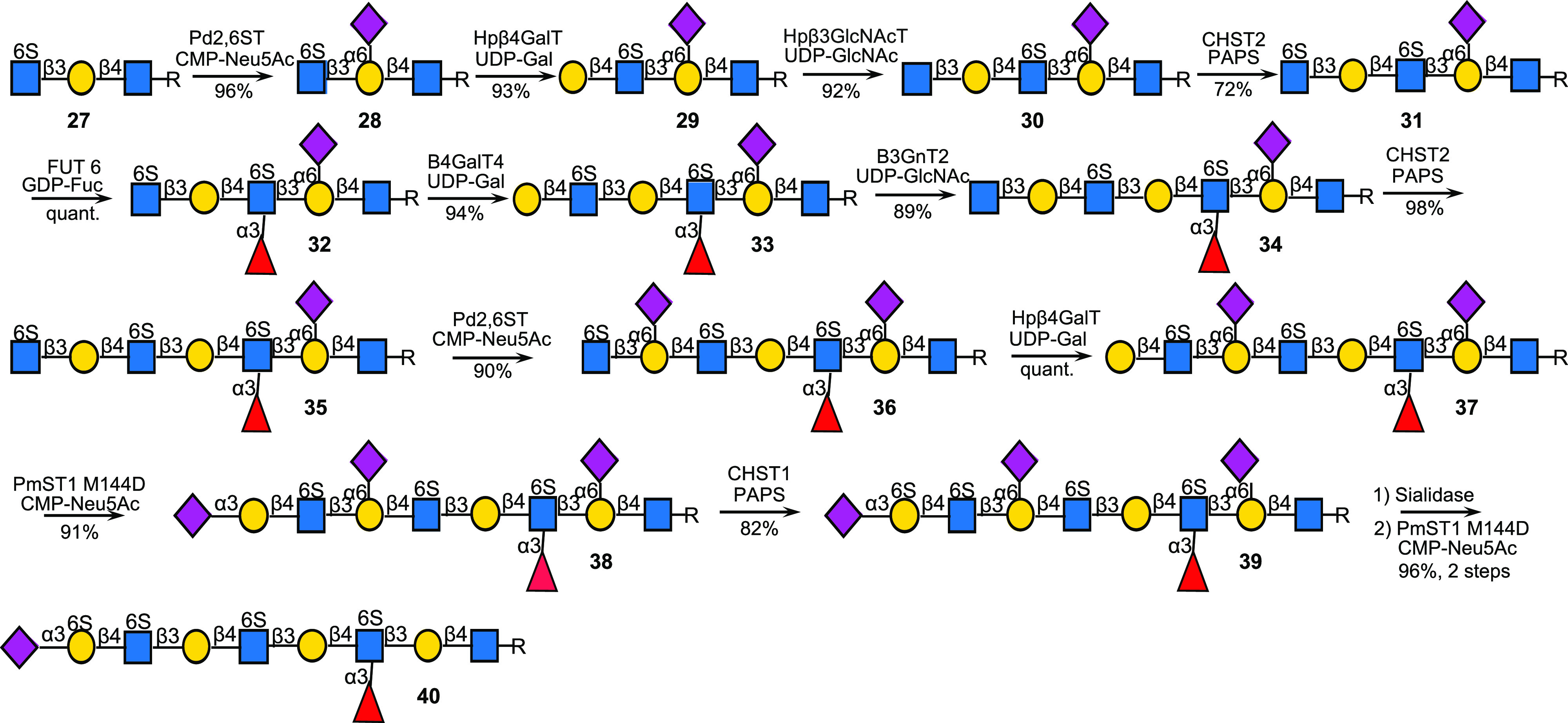
Preparation of KS-II Oligosaccharides by Controlling the Sites of Fucosylation by 2,6-Sialosides; the Fucosides and Sialosides Block Sulfation by CHST1 (R = (CH_2_)_5_NHCbz)

Thus, compound **27** was prepared using the strategy described above. It has only one galactoside that could readily be sialylated at the C-6 position by Pd2,6ST in the presence of CMP-Neu5Ac to give **28** (Scheme 4). Next, the glycan chain of **28** was extended by subsequent modifications by the bacterial glycosyltransferases Hpβ4GalT^43^ and β3GlcNAcT from *Helicobacter pylori* (Hpβ3GlcNAcT)^43,44^ to give **30** which was sulfated at the terminal GlcNAc moiety by CHST2 in the presence of PAPS to provide **31**. The GlcNAc moieties at the reducing and nonreducing end are blocked from fucosylation because of the presence of an α2,6-sialoside and being positioned terminally, respectively. Therefore, only the central LacNAc moiety was fucosylated when treated with FUT6 in the presence of GDP-Fuc resulting in the formation of compound **32**. The application of another module of enzymatic transformations by B4GalT4, B3GnT2, and CHST2 made it possible to convert **32** into **35**. The central LacNAc moiety of **35** is fucosylated and therefore is blocked from sialylation by Pd2,6ST and thus only the LacNAc moiety at the nonreducing end is a substrate for this enzyme. As expected, the sialylation of **35** with Pd2,6ST in the presence of CMP-Neu5Ac resulted in the selective formation of **36**. The latter compound was galactosylated by Hpβ4GalT and then further modified by an α2,3-linked sialoside using PmST1M144D to provide compound **38**. The Gal moiety between the sialoside and GlcNAc6S of **38** is an appropriate substrate for CHST1 whereas the others are not available for sulfation due to the presence of an α2,6-linked sialoside or an α1,3-fucoside. As expected, treatment of **38** with CHST1 and PAPS resulted in monosulfation to give **39** (5.1 mg). Finally, all sialosides were removed by treatment with the neuraminidase of *C. perfringens* followed by α2,3-sialylation of the terminal Gal6S using PmST1M144D in the presence of CMP-Neu5Ac to give the target compound **40** (1.9 mg). NMR analysis of this compound could be performed in D_2_O; however, after removal of the Cbz moiety to give compound **S23**, a PBS D_2_O buffer (40 mM, pH 6.5) was required to provide well resolved signals (Figure S6a,b).

**Scheme 5:**
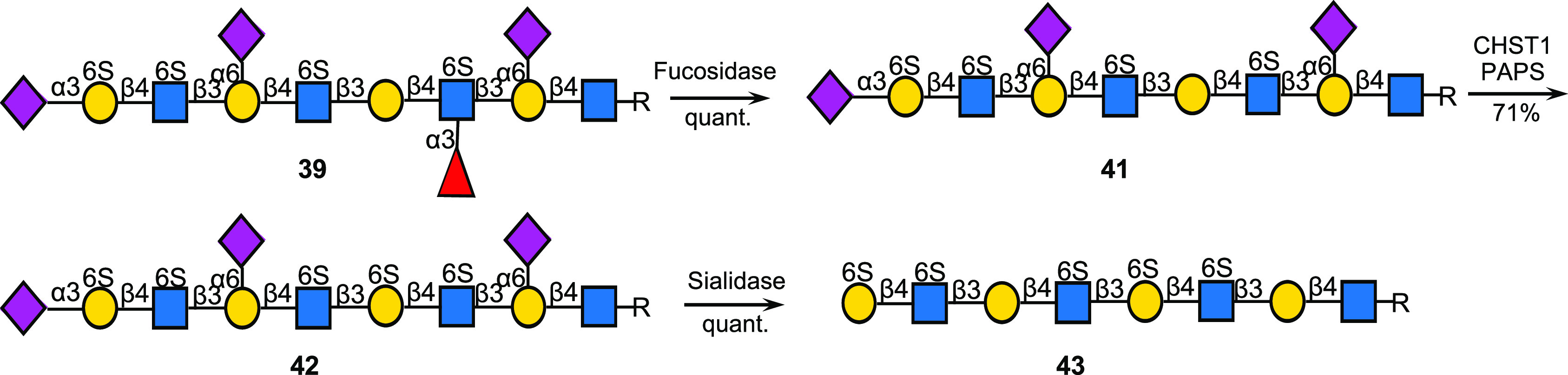
Further Diversification of KS Oligosaccharides by Selective Removal of Fucosides To Reveal a New Site for Sulfation by CHST1 (R = (CH_2_)_5_NHCbz)

Intermediate compounds can be employed to prepare additional derivatives, and for example, the fucosyl moiety of **39** could readily be removed by treatment with the fucosidase of *R. gnavus* to provide **41** (Scheme 5). Removal of the fucoside unmasks the corresponding galactosyl moiety that could readily be sulfated by CHST1 and PAPS to provide compound **42**. The latter compound could easily be transformed into selectively sulfated derivative **43** (0.7 mg) using standard procedures.

Detailed NMR analysis confirmed the positions of sulfates, α2,3- and α2,6-sialosides and α1,3-fucosides. For example, 1D ^1^H NMR and 2D ^13^C–^1^H HSQC spectra of compound **39** made it possible to assign all proton and carbon signals (Figure S3a-c). The C-6 of the three internal sulfated GlcNAc residues substantially shifted downfield from δ60.5 to δ66.3 for GlcNAc-C, δ66.6 for GlcNAc-E, and δ66.3 for GlcNAc-G. The corresponding protons also exhibited expected chemical shift differences from H6a δ3.98 and H6b δ3.82 to H6a,b δ4.35 for GlcNAc-C, H6a,b δ4.35 for GlcNAc-G and H6a δ4.40 and H6b δ4.30 for GlcNAc-E. C-6 of the internal sulfated galactosyl moiety had also substantially shifted downfield (δ60.9 → δ66.7), and the corresponding protons also showed a chemical shift difference (H6 δ3.74 → δ4.18). The H-3 of the fucosylated GlcNAc moiety had substantially shifted from δ3.77 to δ3.90, and the corresponding nearby H-2 also shifted from δ3.83 to δ3.97, which confirmed the regioselectivity of the α1,3-fucosylation of GlcNAc. C-6 of the two internal α2,6-sialylated galactoside moieties had substantially shifted downfield from δ 61.1 to δ63.5 for Gal-B and Gal-F. The corresponding H-6 protons also shifted from δ 3.76 to H6a δ4.00 and H6b δ3.55 which confirmed the regioselectivity α2,6-sialylation of the galactosides. H-3 of the terminal α2,3-sialylated galactoside had substantially shifted from δ3.72 to δ4.16, which confirmed the regioselectivity of α2,3-sialylation of galactose. Although overlap was observed in the 2D NOESY spectrum (300 ms, Figure S3c), inter-residue connectivities of Gal-H H-1 to GlcNAc-G H-4, GlcNAc-G H-1 to Gal-F H-3, Gal-F H-1 to GlcNAc-E H-4, GlcNAc-E H-1 to Gal-D H-3, Gal-D H-1 to GlcNAc-C H-4, GlcNAc-C H-1 to Gal-B H-3, and Gal-B H-1 to GlcNAc-A H-4 could be assigned, which is in agreement with the following connectivity H(1 → 4)G, G(1 → 3)F, F(1 → 4)E, E(1 → 3)D, D(1 → 4)C, C(1 → 3)B, and B(1 → 4)A linkages, respectively.

### Chemoenzymatic Synthesis of a Selectively Sulfated *N*-Glycan

**Scheme 6:**
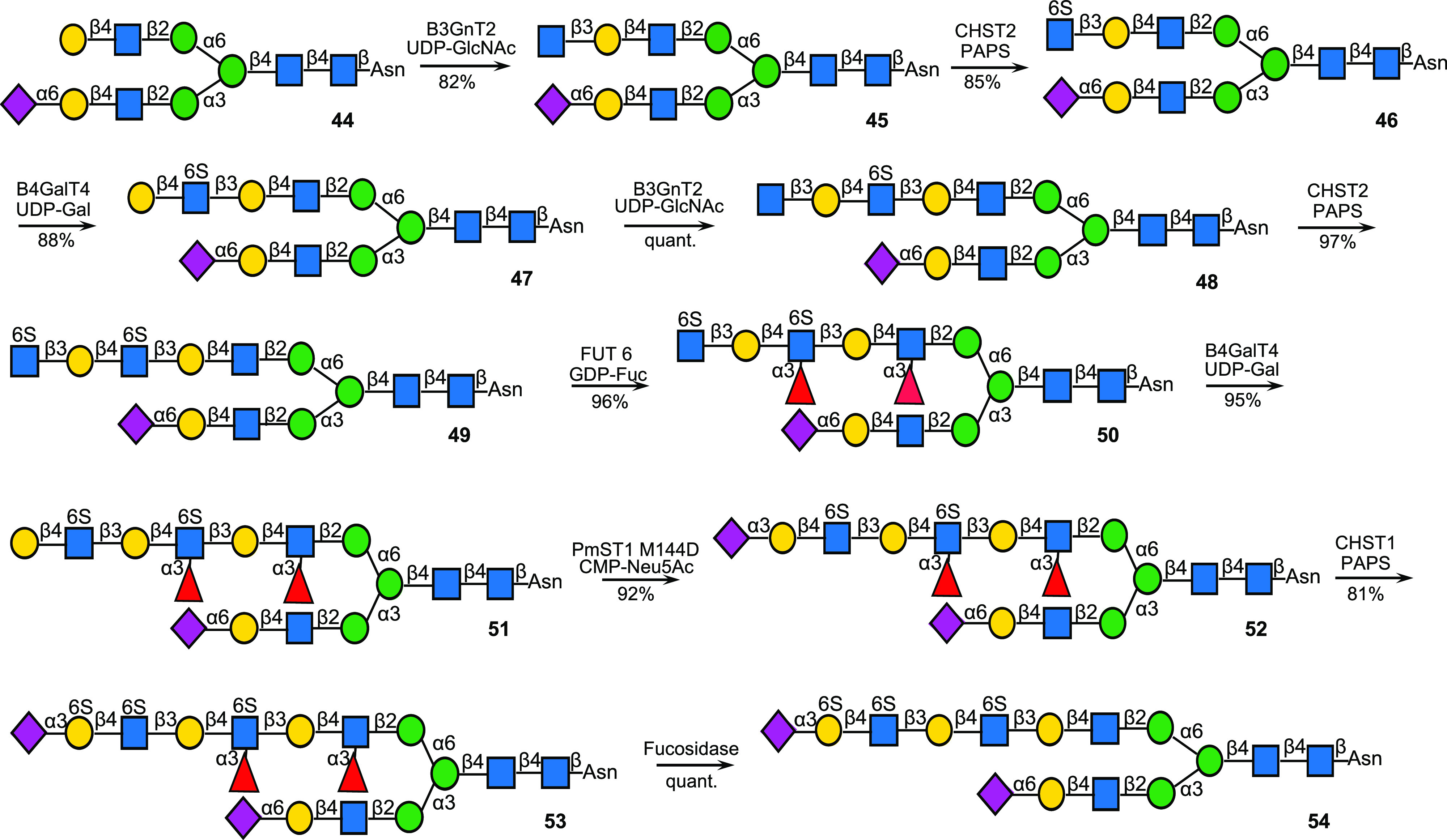
Total Synthesis of KS-I Oligosaccharide Starting from an *N*-Glycan Obtained from a Sialoglycopeptide (SGP) Isolated from Egg Yolk Powder

To showcase the scope of the methodology, we prepared KS-I derivative **54** in a quantity of 1.6 mg which has one galactoside moiety that is modified by a sulfate (Scheme 6). It made use of key intermediate **52** in which two galactosides are blocked from sulfation by neighboring fucosides and another one by the presence of an α2,6-sialoside. The synthesis started with *N*-glycan **44** derived from a sialoglycopeptide (SGP) isolated from egg yolk powder that was subsequently treated with Pronase to remove most of the peptide and leaving a single asparagine moiety, and a sialidase to remove the sialosides and selective resialylation of the α1,3-antennae using ST6Gal1.^26,45^ The terminal galactoside of the α(1,6)-arm of **44** was extended by a GlcNAc moiety by treatment with B3GnT2 in the presence of UDP-GlcNAc (→**45**), which was selectively sulfated by CHST2 and PAPS to provide **46**. Next, the GlcNAc6S moiety was extended by an additional 6-sulfo-LacNAc moiety by subsequent treatment with B4GalT4 (→**47**), B3GnT2 (→**48**), and CHST2 to give **49**. The terminal 2,6-sialylated GlcNAc moiety of **49** is not a substrate for FUT6; hence, it was possible to selectively fucosylate the two internal GlcNAc units to provide compound **50**. The terminal galactoside of compound **51** was modified by an α2,3-sialoside using PmST1M144D in the presence of CMP-Neu5Ac to give **52**. The galactoside that is neighbored by an α2,3-sialoside and GcNAc6S moiety is a proper substrate for CHST1, whereas the other galactosides are blocked from modification due to the presence of an α2,6-sialoside or an α1,3-linked fucoside at the neighboring GlcNAc moiety. As expected, treatment of **52** with CHST1 in the presence of PAPS resulted in the selective formation of **53** (2.9 mg) which after treatment with the fucosidase from *R. gnavus* provided KS-I derivative **54** having a selective sulfation pattern.

## Conclusions

Enzymatic and chemoenzymatic synthesis of glycans and glycoconjugates have progressed considerably, making it possible to prepare a wide variety of highly complex compounds.^46−50^ Many prokaryotic and eukaryotic derived glycosyltransferases have been described and can readily be expressed using *Escherichia coli* or mammalian cell-based platforms. These enzymes can install glycosidic linkages in a regio- and stereospecific manner and reactions can be driven to completion by using alkaline phosphatases to hydrolyze nucleotide products that can act as product inhibitor.^51−53^ The efficiency of glycosyltransferase mediated glycan assembly has made it possible to prepare complex oligosaccharides in an automated fashion.^54−56^ Complex glycans can have several acceptor sites for a given glycosyltransferase, making it difficult to prepare discrete compounds. Site selective glycosylations can, however, be accomplished by careful synthetic planning,^45^ the use of unnatural sugar nucleotide donors,^57,58^ chemical modifications,^59−61^ or temporary monosaccharide blocking groups.^42^

Complex carbohydrates can be modified by entities such as sulfates;^62−64^ however, methods to install such modifications in a site-specific manner are still lacking. Here, we describe a biomimetic approach that can provide a wide range of differently sulfated and fucosylated KS oligosaccharides. Although the KS biosynthetic enzymes cooperate to construct specific epitopes, they do not provide strict control over the exact positions of sulfates, fucosides, and terminal structural elements. As a result, it has not been possible to exploit enzymes for the preparation of a wide range of well-defined KS-oligosaccharides, especially those having highly complex structures. To address this deficiency, we developed a biomimetic approach that can install any structural motif of the KS in a controlled manner. It exploits the specificity of sulfotransferase CHST2 that only sulfates terminal GlcNAc moieties of a poly-LacNAc chain. The resulting terminal GlcNAc6S residue can then be extended by B4GalT4 or Hpβ4GalT to give sulfo-LacNAc, which, in turn, can be extended by further LacNAc or sulfo-LacNAc units. The strategy further exploits the mutual exclusivity of several structural elements of KS. We carefully analyzed the structural elements of KS which indicated that fucosylation of GlcNAc and sulfation of Gal cannot occur at the same LacNAc moiety.^23,65^ This observation was exploited for the selective installation of sulfates at galactosides by preparing oligo-LacNAc chains with specific patterns of fucosylation. Fucosylation could efficiently be accomplished by FUT6 that accept LacNAc as well as sulfo-LacNAc (Gal-1,4-GlcNAc6S) as substrate to give Le^x^ and sulfo-Le^x^ moieties, respectively. It was observed that the mammalian fucosyl transferase, FUT5, cannot efficiently modify sulfo-LacNAc resulting in incomplete modifications. We examined a range of fucosylated substrates and confirmed that these cannot be modified by CHST1. The pattern of fucosylation could be controlled by temporary modification of the C-6 positions of galactosides by an α2,6-sialoside. In this respect, *Photobacterium damselae* α2,6-sialyltransferase (Pd2,6ST) has flexible acceptor substrate specificity and can install α2,6-sialosides at internal as well as external LacNAc moieties.^42^ We found that Pd2,6ST can accept LacNAc as well as sulfo-LacNAc moieties and that the resulting α2,6-sialoside also blocks fucosylation by FUT6. Interestingly, a GlcNAc6S that neighbors a Gal residue having an α2,6-sialoside could not readily be galactosylated by B4GalT4; however, bacterial Hpβ4GalT could quantitatively perform this transformation. At an appropriate stage of the synthetic strategy, the α2,6-sialosides can be removed by the sialidase of *C. perfringens* and the α1,3-fucosides by the fucosidase of *R. gnavus* to give KS oligosaccharides. The fucosides and sialosides are orthogonal blocking groups and can individually be removed to reveal galactosides that can be further sulfated by CHST1. Our studies also confirm that sulfation of Gal blocks fucosylation by FUT6 thereby providing an orthogonal approach for site-specific fucosylation and an entry into KSII oligosaccharides. We also developed an alternative approach to control the site selectivity of CHST1 by installing α2,6-sialosides that block specific galactosides from sulfation.

**Figure 3:**
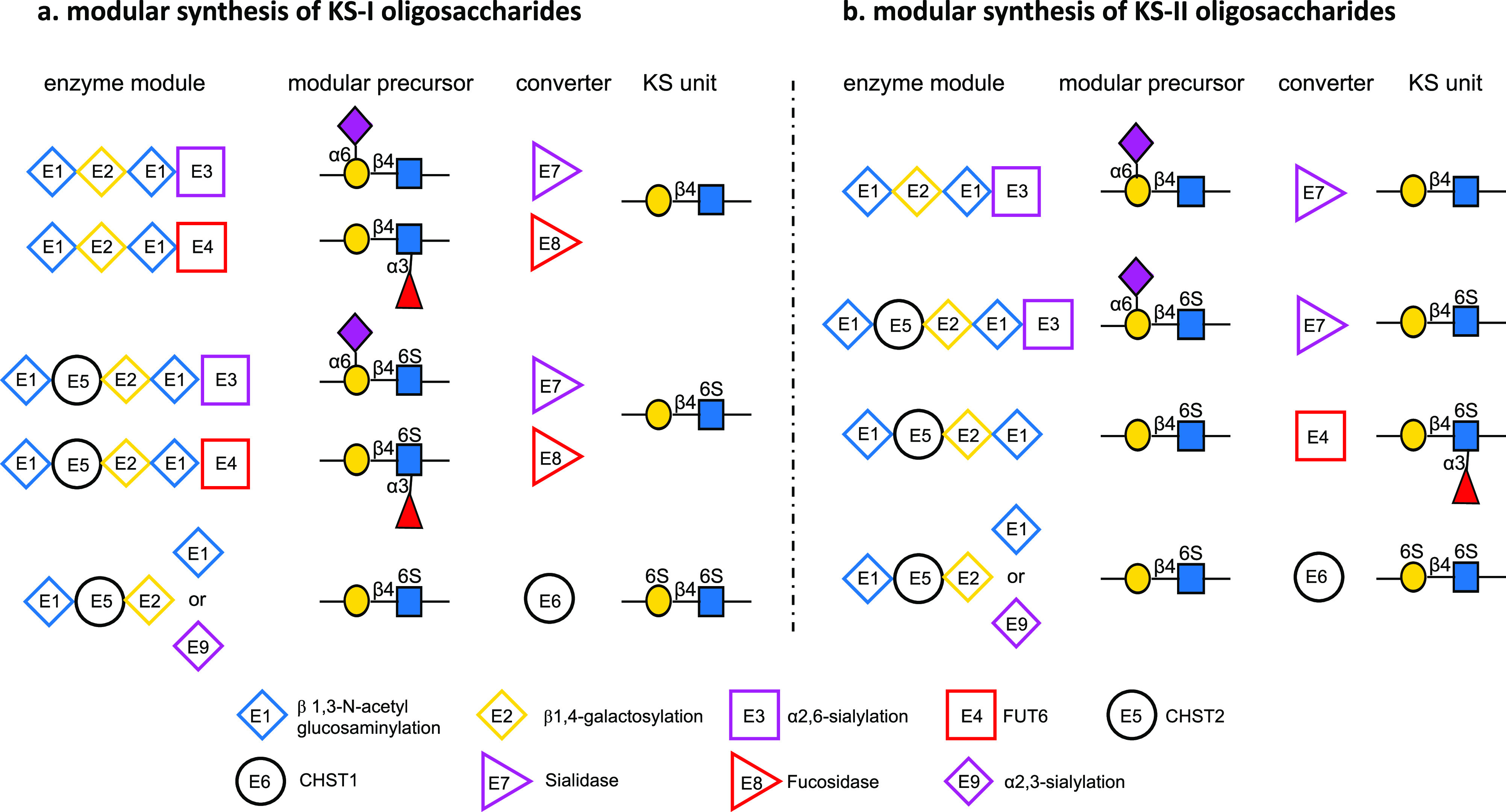
Any KS-I and K-II oligosaccharides can be prepared using specific enzyme modules to install specially modified LacNAc moieties. After oligosaccharide assembly, specific galactosides are sulfated by CHST1 and sialosides and/or fucosides removed by a sialidase or fucosidase (converter) to give target structures.

The biomimetic approach is highly modular, and by using specific enzymatic sequences (enzyme modules), it is possible to assemble the various KS substructures (Figure 1c) in any possible order (Figure 3), which can then be capped by the different terminal epitopes. In the case of KS-I oligosaccharides, the pattern of sulfation at Gal can be controlled by installation of specific patterns of α2,6-sialosides or α1,3-fucosides, and thus, specific sulfated compounds can be prepared by different approaches. KS-II is structurally more complex and is also modified by α1,3-fucosides. For the preparation of these compounds, α2,6-sialylation is used to control the pattern of fucosylation and sulfation at Gal. We have also demonstrated that by one sequence of enzymatic transformations several KS oligosaccharides can be prepared and for example the blocking fucosides and sialosides are orthogonal and can selectively be removed to give sites for further sulfation by CHST1. The biomimetic approach has given KS oligosaccharides of unprecedented complexity in milligram quantities, including *N*-glycans.

KS is the least understood member of proteoglycans,^3,63,66^ which in part is due to a lack of panels of well-defined KS oligosaccharides. The methodology described here makes it possible to prepare KS-I and KS-II oligosaccharides having intricate patterns of sulfation and fucosylation. The resulting compounds will provide opportunities to establish binding selectivities of KS binding proteins, which in turn may uncover possible sulfation and fucosylation codes. It will also make it possible to determine ligand requirements of KS-binding antibodies that are used to determine the presence of specific structural motifs on cells and tissues. Collections of KS oligosaccharides will make it possible to determine substrate specificities of keratinases that are used for partial degradation for subsequent structure elucidation. These molecules will also provide analytical standards to develop methods for structure determination. It has been realized that KS is involved in many disease processes,^2^ and the ability to prepare well-defined KS oligosaccharide is expected to provide leads compound for drug discovery.

## Supporting Information Available

Schemes S1–S7, experimental procedures, and product characterization data for all new compounds (PDF)
