# Supplementary material for: A Biomimetic Synthetic Strategy Can Provide Keratan Sulfate I and II Oligosaccharides with Diverse Fucosylation and Sulfation Patterns
Source: J Am Chem Soc. 2024 Mar 18;146(13):9230–40. doi: 10.1021/jacs.4c00363 (PMC10996015; doi:10.1021/jacs.4c00363)
Supplement: Supplementary file 1 — ja4c00363_si_001.pdf [file ja4c00363_si_001.pdf]

Supplementary Information

A Biomimetic Synthetic Strategy can Provide Keratan Sulfate I and II Oligosaccharides with Diverse Fucosylation and Sulfation Patterns

Yunfei Wu,[1] Gerlof P. Bosman,[1] Digantkumar Chapla,[2] Chin Huang,[2,3] Kelley W. Moremen,[2,3] Robert P. de Vries,[1] and Geert-Jan Boons[1,2,4,*]

[1]Department of Chemical Biology and Drug Discovery, Utrecht Institute for Pharmaceutical Sciences, and Bijvoet Center for Biomolecular Research, Utrecht University, Universiteitsweg 99, 3584 CG Utrecht, The Netherlands

[2]Complex Carbohydrate Research Center, University of Georgia, 315 Riverbend Road, Athens, GA 30602, USA

[3]Department of Biochemistry, University of Georgia, Athens, GA 30602, USA

[4]Department of Chemistry, University of Georgia, Athens, GA 30602, USA

*Corresponding author. E-mail: gjboons@ccrc.uga.edu or g.j.p.h.boons@uu.nl

Table of Contents

1) Supplementary Schemes …………………………………………...………………. S3

2) Materials and Methods ……………………………………...…………..………. S5

3) General Protocols for HILIC-HPLC Purification ……………...……………………. S11

4) Analytical Data ………………………………………………...…………………. S12

5) Experimental Procedures and Analysis …………………………...…………...……. S28

6) References ……………………………………………………...……………..…. S138

7) NMR Spectra ……………………………………………...…………….……. S139

1) Supplementary Schemes

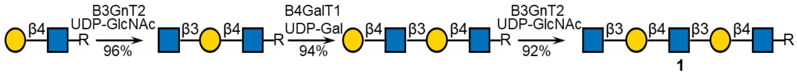

Scheme S1. Enzymatic synthesis of intermediate **1**. R = $O(CH_2)_5NHCbz$.

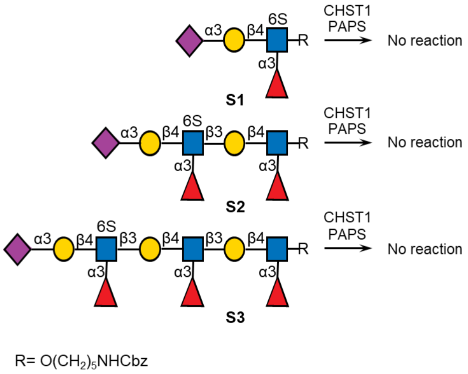

Scheme S2. Blocking sulfation at galactose by 1,3-fucosylation of GlcNAc.

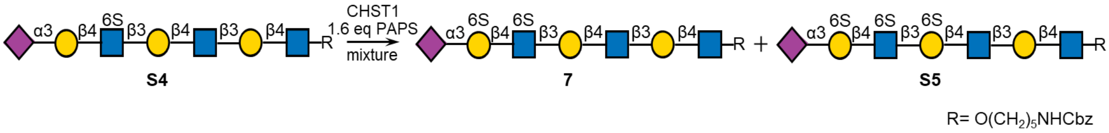

Scheme S3. CHST1 modification of compounds having a sialoside and sulfate.

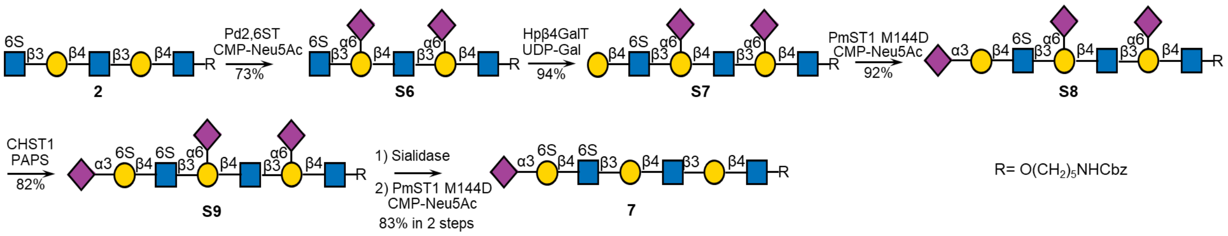

Scheme S4. Blocking sulfation at galactose by 2,6-sialylation. Enzymatic synthesis of oligosaccharides **S8** to examine the substrate specificity of CHST1.

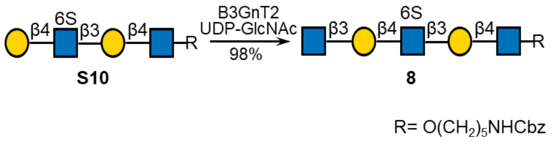

Scheme S5. Enzymatic synthesis of intermediate **8**.

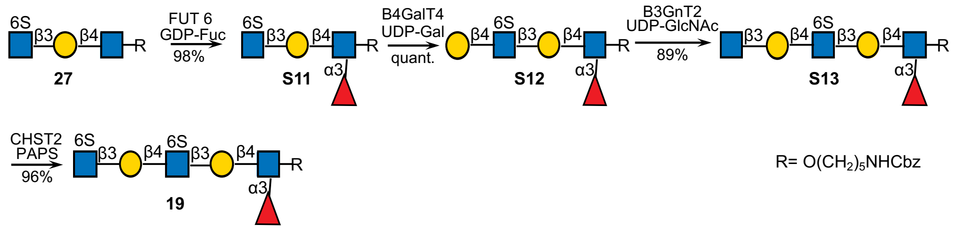

Scheme S6. Enzymatic synthesis of intermediate **19**.

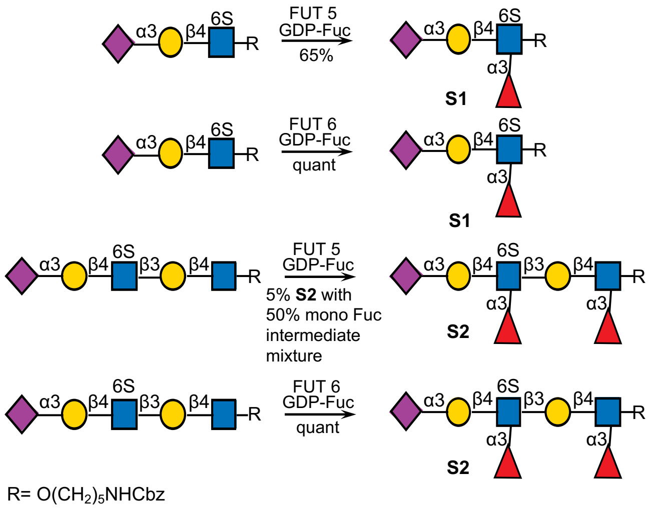

Scheme S7. To compare the activity of FUT5 and FUT6 for Lewis^x and 6-sulfated sialyl Lewis^x.

2) Materials and Methods

Glycosyltransferases Pd2,6ST, Hpβ3GlcNAcT, Hpβ4GalT, PmST1 M144D were expressed and purified according to reported protocols.[1-5] The synthesis of advanced intermediates **1**, **27**, **44**, **S4**，**S10**, and **S18** are described previously.[6] Reagents were purchased from Sigma-Aldrich. Neuraminidase (Sialidase) was obtained from Merck [Cat# 11585886001]. Uridine 5'-diphosphogalactose (UDP-Gal), uridine 5'-diphospho-N-acetyl-glucosamine (UDP-GlcNAc) and cytidine-5'-monophospho-N-acetylneuraminic acid (CMP-Neu5Ac) were obtained from Roche Diagnostics [UDP-Gal: Cat# 07703562103; UDP-GlcNAc: Cat# 06369855103; CMPNeu5Ac: Cat# 05974003103]. Adenosine 3′-phosphate 5′-phosphosulfate (PAPS) was obtained from Merck [Cat# 118410, Purity ≥ 80% by HPLC]. GDP-Fucose was prepared using L-fucokinase/GDP-fucose pyrophosphorylase.[7] Progress of reactions was monitored by liquid chromatography mass spectrometry system (LCMS) from Shimadzu (system controller: SCL10A-VP; HPLC pumps: LC10AD-VP; injector: SIL10AD-VP) using a ZIC HILIC column (ZeQuant, PEEK coated guard HPLC column, 3.5 μm particle size, 20x 2.1 mm). The LC system was attached to a Bruker Daltonics micro TOF-Q mass spectrometer. Mass spectra were recorded on an Applied Biosystems SCIEX MALDI TOF/TOF 5800 mass spectrometer, a Shimadzu Biotech Axima-CFR MALDI-TOF, or a high resolution Shimadzu LCMS-IT-TOF mass spectrometer. Reaction mixtures were purified using a size exclusion Biogel (P2) or Biogel (P6) resins from BioRad in Econo glass columns (0.7 x 30 cm / 1.5 x 30 cm / 1.5 x 50 cm/ 1.5 x 120 cm) coupled to a BioFrac fraction collector (BioRad). Carbohydrate-containing fractions were detected by thin layer chromatography and an appropriate staining reagent (15 mL AcOH and 3.5 mL p-Anisaldehyde in 350 mL EtOH and 50 mL H_2SO_4). If needed, further purification was performed by HPLC-MS using a ZIC HILIC column.

Expression and purification of recombinant human glycosyltransferases and sulfotransferases

Expression constructs were generated encoding the truncated catalytic domains of human glycosyltransferases (B4GALT1, B4GALT4, B3GNT2, FUT6, ST6GAL1, and ST3GAL4) and sulfotransferases (CHST1 and CHST2) as NH_2-terminal fusion proteins in the pGEn2 expression vector essentially as described in prior studies.[6, 8-9] Briefly, the fusion protein coding regions were comprised of a 25-amino acid signal sequence, an His_8 tag, AviTag, the "superfolder" GFP coding region, the 7-amino acid recognition sequence of the tobacco etch virus (TEV) protease followed by the respective catalytic domain regions (for human CHST1 (Uniprot ID: O43916) and CHST2 (Uniprot ID: Q9Y4C5) catalytic domain region comprising of 388 and 454 amino acid residues, respectively). The recombinant human glycosyltransferases and sulfotransferases were expressed as a soluble secreted proteins by transient transfection of suspension culture HEK293-F cells (FreeStyle™ 293-F cells, Thermo Fisher Scientific, Waltham MA) and purified by Ni^{2+}-NTA chromatography as previously described.[8-9] Each protein was concentrated to approximately 3 mg/mL using an ultrafiltration pressure cell (Millipore, Billerica, MA) with a 10-kDa molecular mass cutoff membrane. The enzymes were further purified by gel filtration on a Superdex G-75 column (GE Healthcare) preconditioned with a buffer containing 20 mM HEPES, 150 mM NaCl, 0.05% sodium azide, pH 7.0. Peak fractions of recombinant human enzymes were pooled, respectively, concentrated

at 1 mg/mL and buffer exchanged with 20 mM HEPES, 100 mM NaCl, 0.05% sodium azide, pH 7.0, 10% glycerol. The final protein preparations were aliquoted and stored at -80 °C until use.

Fucosidase production

The protein sequence of fucosidase E1_10125 from *Ruminococcus gnavus* E1[10] was obtained from the RCSB Protein Data Bank under accession number 6TR3. The nucleotide sequence was obtained from the closest hit in a protein-protein search in BLAST, of which the nucleotide sequence was corrected to obtain the exact same amino acid sequence. This open reading frame was ordered at GenScript and cloned into backbone pET23A in frame with a His-tag on the C-terminus of the open reading frame of the fucosidase. Cloning was performed in JM109 *Escherichia coli* (Promega). The plasmid also contains an ampicillin resistance gene. The plasmid is deposited at Addgene under the name: pET23A-Fucosidase-E1_10125-Ruminococcus_gnavus-His (#207665). Expression of the fucosidase was performed in BL21 (DE3) *E. coli* (New England Biolabs). An overnight culture (90 mL per liter culture) was inoculated in 2xYT medium (Serva) supplemented with 50 µg/mL ampicillin (13398.02, Serva). Bacteria were grown at 37 °C while shaking at 200 rpm until OD 0.8-1.0, after which fucosidase production was induced with 1 mM isopropyl β-D-1-thiogalactopyranoside (R0309, Invitrogen). The bacteria were grown for 21 h at room temperature while shaking at 200 rpm. Cell pellets were obtained by centrifugation in a swing-out centrifuge for 30 min at 4 °C at 629 rcf. The pellets were resuspended in 50 mL lysis buffer (100 mM Tris-HCl, pH 8.0, 0.1%TritonX-100) per liter bacterial culture, which was supplemented with 1 gram per liter bacterial culture lysozyme (62971, Merck), 25 µL per liter bacterial culture DNAse (EN0521, Thermo Fisher Scientific), and 25 µL per liter bacterial culture DNAse buffer. The mixtures were incubated for 50 min at 37°C while shaking at 200 rpm. The cells were further lysed by sonication (Bandelin, Sonopuls, needle MS73) at 50% amplitude, three times for one minute at 10 second intervals. The lysates were centrifuged for 1.5 h at 4 °C at 629 rcf until the supernatant was clear. The supernatant was filtered through a 0.45 µm filter (431220, Corning) and incubated for 16 h with Ni-NTA beads at 4 °C while rotating. The beads were washed using washing buffer (0.5 M NaCl, 20 mM Tris-HCl. pH 7.5) after which the fucosidase was eluted using the same buffer supplemented with 10-200 mM imidazole. The eluates were concentrated and buffer exchanged to fucosidase buffer (50 mM citrate buffer with 5 mM $CaCl_2$, pH 6.0) using a centrifugal concentrator with a molecular weight cutoff of 10 kDa (Vivaspin 6, VS0602, Sartorius). The presence of the fucosidase (62 kDa) was evaluated by SDS-PAGE gel (after denaturing for 15 min at 95 °C with the addition of denaturing buffer (NP0009, Invitrogen)) with staining using Coomassie blue dye.

General protocols for enzymatic reactions

General procedure for the installation of β1,3 GlcNAc using B3GnT2

Glycosyl acceptor (1.0 eq) and UDP-GlcNAc (1.5 eq) were dissolved to provide a final acceptor concentration of 2-5 mM in a HEPES buffer (50 mM, pH 7.3) containing KCl (25 mM), $MgCl_2$ (2 mM) and DTT (1 mM). Calf intestine alkaline phosphotase (CIAP, 1% total volume, 1 kU/mL) and B3GnT2 (1% wt/wt relative to acceptor substrate) were added, and the reaction mixture was incubated overnight at 37 °C with gentle shaking. The progress of the reaction was monitored by MALDI-TOF MS or ESI-TOF MS, and if starting material remained after 18 h, another portion of B3GnT2 was added until no starting material could be detected. The reaction mixture was centrifuged over a Nanosep® Omega ultrafiltration device (10 kDa MWCO) to remove proteins and the filtrate was lyophilized. The residue was applicated to P2 or P6 size-exclusion column chromatography using Milli–Q water as eluent to provide the desired product after lyophilization of appropriate fractions. High performance liquid chromatography (HPLC) using a HILIC column (see materials) was employed when impurities were detected.

General procedure for the installation of β1,3 GlcNAc using Hpβ3GlcNAcT

Glycosyl acceptor (1.0 eq) and UDP-GlcNAc (1.5 eq) were dissolved to a final acceptor concentration of 2-5 mM in a HEPES buffer (50 mM, pH 7.3) containing KCl (25 mM), MgCl2 (2 mM) and DTT (1 mM). Calf intestine alkaline phosphotase (CIAP, 1% total volume, 1 kU/mL) and Hpβ3GlcNAcT (1% wt/wt relative to acceptor substrate) were added, and the reaction mixture was incubated overnight at 37 °C with gentle shaking. The progress of the reaction was monitored by MALDI-TOF MS or ESI-TOF MS, and if starting material remained after 18 h, another portion of Hpβ3GlcNAcT was added until no starting material could be detected. The reaction mixture was centrifuged over a Nanosep® Omega ultrafiltration device (10 kDa MWCO) to remove proteins and the filtrate was lyophilized. The residue was applied to P2 or P6 size-exclusion column chromatography using Milli–Q water as eluent to provide the desired product after lyophilization of appropriate fractions. Semi-preparative HPLC using a HILIC column (see materials) was employed when impurities were detected.

General procedure for the installation of β1,4 Gal using B4GalT1

Glycosyl acceptor (1.0 eq) and UDP-Gal (1.5 eq per Gal) were dissolved to provide a final acceptor concentration of 2-5 mM in a Tris buffer buffer (100 mM, pH 7.5) containing $MnCl_2$ (10 mM) and BSA (1% total volume). CIAP (1% volume total) and B4GalT1 (1% wt/wt relative to acceptor substrate) were added, and the reaction mixture was incubated overnight at 37 °C with gentle shaking. The progress of the reaction was monitored by MALDI-TOF MS or ESI-TOF MS, and if starting material remained after 18 h, another portion of B4GalT1 was added until no starting material was detected. The reaction mixture was centrifuged over a Nanosep® Omega ultrafiltration device (10 kDa MWCO) to remove proteins and the filtrate was lyophilized. The residue was applied to P2 or P6 size-exclusion column chromatography using Milli–Q water as eluent to provide the desired product after lyophilization of appropriate

fractions. Semi-preparative HPLC using a HILIC column (see materials) was employed when the impurities were founded after size exclusion.

General procedure for the installation of β1,4 Gal using B4GalT4

Glycosyl acceptor (1.0 eq) and UDP-Gal (1.5 eq per Gal) were dissolved to provide a final acceptor concentration of 2-5 mM in a Tris buffer buffer (100 mM, pH 7.5) containing $MnCl_2$ (10 mM) and BSA (1% total volume). CIAP (1% volume total) and B4GalT4 (1% wt/wt relative to acceptor substrate) were added, and the reaction mixture was incubated overnight at 37 °C with gentle shaking. The progress of the reaction was monitored by MALDI-TOF MS or ESI-TOF MS, and if starting material remained, another portion of B4GalT4 was added until no starting material could be detected. The reaction mixture was centrifuged over a Nanosep® Omega ultrafiltration device (10 kDa MWCO) to remove proteins and the filtrate was lyophilized. The residue was applied to P2 or P6 size-exclusion column chromatography using Milli–Q water as eluent to provide the desired product after lyophilization of appropriate fractions. Semi-preparative HPLC using a HILIC column (see materials) was employed when impurities were detected after size exclusion column chromatography.

General procedure for the installation of β1,4 Gal using Hpβ4GalT

Glycosyl acceptor (1.0 eq) and UDP-Gal (1.5 eq per Gal) were dissolved to provide a final acceptor concentration of 2-5 mM in a Tris buffer buffer (100 mM, pH 7.5) containing $MnCl_2$ (10 mM) and BSA (1% total volume). CIAP (1% volume total) and Hpβ4GalT (1% wt/wt relative to acceptor substrate) were added, and the reaction mixture was incubated overnight at 37 °C with gentle shaking. The progress of the reaction was monitored by MALDI-TOF MS or ESI-TOF MS, and if starting material remained, another portion of Hpβ4GalT was added until no starting material could be detected. The reaction mixture was centrifuged over a Nanosep® Omega ultrafiltration device (10 kDa MWCO) to remove proteins and the filtrate was lyophilized. The residue was applied to P2 or P6 size-exclusion column chromatography using Milli–Q water as eluent to provide the product after lyophilization of the appropriate fractions. Semi-preparative HPLC using a HILIC column (see materials) was employed when the impurities were detected after size exclusion column chromatography.

General procedure for the installation of α2,3 Neu5Ac using PmST1 M144D

Glycosyl acceptor (1.0 eq) and CMP-Neu5Ac (1.5 eq) were dissolved at a final acceptor concentration of 2-5 mM in a MOPS buffer (50 mM, pH 7.2) containing BSA (1% total volume). CIAP (1% volume total) and PmST1 M144D (1% wt/wt relative to acceptor substrate) were added, and the reaction mixture was incubated overnight at 37 °C with gentle shaking. The progress of the reaction was monitored by ESI-TOF MS, and if starting material remained, another portion of PmST1 M144D was added until no starting material could be detected. The reaction mixture was centrifuged over a Nanosep® Omega ultrafiltration device (10 kDa MWCO) to remove proteins, and the filtrate was lyophilized. The residue was applied to P2 or P6 size-exclusion column chromatography using Milli–Q water as eluent to provide the desired product after lyophilization of the appropriate fractions. Semi-preparative HPLC using a HILIC

column (see materials) was employed when the impurities were detected after size exclusion column chromatography.

General procedure for the installation of α2,6 Neu5Ac using PT2,6ST

Glycosyl acceptor (1.0 eq) and CMP-Neu5Ac (2.0 eq per Gal to be added) were dissolved at a final acceptor concentration of 2-5 mM in a MOPS buffer (50 mM, pH 7.2) containing BSA (1% volume total). CIAP (1% volume total) and PT2,6ST (1% wt/wt relative to acceptor substrate) were added, and the reaction mixture was incubated overnight at 37 °C with gentle shaking. The reaction mixture was centrifuged over a Nanosep® Omega ultrafiltration device (10 kDa MWCO) to remove proteins, and the filtrate was lyophilized. The residue was applied to P2 or P6 size-exclusion column chromatography using Milli–Q water as eluent to provide the desired product after lyophilization of the appropriate fractions. Semi-preparative HPLC using a HILIC column (see materials) was employed when the impurities were detected after size exclusion.

General procedure for the installation of α1,3 Fuc using FUT6

Glycosyl acceptor (1.0 eq) and GDP-Fuc (1.5 eq per GlcNAc to be added) were dissolved at a final acceptor concentration of 2-5 mM in a Tris buffered solution (50 mM, pH 7.3) containing $MnCl_2$ (10 mM). CIAP (1% total volume) and FUT6 (1% wt/wt) were added, and the reaction mixture was incubated overnight at 37 °C with gentle shaking. The reaction mixture was centrifuged over a Nanosep® Omega ultrafiltration device (10 kDa MWCO) to remove proteins, and the filtrate was lyophilized. The residue was applied to P2 or P6 size-exclusion column chromatography using Milli–Q water as eluent to provide the desired product after lyophilization of the appropriate fractions. Semi-preparative HPLC using a HILIC column (see materials) was employed when the impurities were detected after size exclusion.

General procedure for the 6-*O*-sulfate installation of terminal GlcNAc using CHST2

Glycosyl acceptor (1.0 eq) and PAPS (1.6 eq) were dissolved at a final acceptor concentration of 2-5 mM in a Tris buffered solution (100 mM, pH 7.5) containing $MgCl_2$ (10 mM). CHST2 (10-20% wt/wt relative to acceptor substrate) was added, and the reaction mixture was incubated overnight at 37 °C with gentle shaking. The reaction mixture was centrifuged over a Nanosep® Omega ultrafiltration device (10 kDa MWCO) to remove proteins, and the filtrate was lyophilized. The residue was applied to P2 or P6 size-exclusion column chromatography using NH_4HCO_3 buffer (50 mM) as eluent to providing the desired product after lyophilization of the appropriate fractions. Semi-preparative HPLC using a HILIC column (see materials) or DEAE ion exchange column chromatography was employed when impurities were detected.

General procedure for the 6-O-sulfate installation of internal Galactose using CHST1

Glycosyl acceptor (1.0 eq) and PAPS (1.6 eq per galactose) were dissolved at a final acceptor concentration of 2–5 mM in a Tris buffered solution (100 mM, pH 7.5) containing $MgCl_2$ (10 mM). CHST1 (10% wt/wt relative to acceptor substrate) were added, and the reaction mixture was incubated overnight at 37 °C with gentle shaking. The reaction mixture was centrifuged over a Nanosep® Omega ultrafiltration device (10 kDa MWCO) to remove proteins, and the

filtrate was lyophilized. The residue was applied to P2 or P6 size-exclusion column chromatography using NH_4HCO_3 buffer (50 mM) as eluent to provide the desired product after lyophilization of the appropriate fractions. Semi-preparative HPLC using a HILIC column (see materials) was employed when the impurities were detected.

General procedure for the removal of sialic acid using sialidase from *Clostridium perfringens* (*C. perfringens* neuraminidase)

The sialoside (starting material) was dissolved with PBS buffer (pH 6.5) in a 15 mL centrifuge tube. After the addition of appropriate amount (0.1 unit per mg substrate) of sialidase, the reaction mixture was incubated in an incubator shaker at 37 °C overnight with shaking at 300 rpm. The reaction mixture was centrifuged over a Nanosep® Omega ultrafiltration device (10 kDa MWCO) to remove proteins, and the filtrate was lyophilized. The residue was applied to P2 or P6 size-exclusion column chromatography using NH_4HCO_3 buffer (50 mM) as eluent, to provide the desired product after lyophilization of the appropriate fractions.

General procedure for the removal of fucose using fucosidase from *Ruminococcus gnavus* E1 (*R. gnavus* E1)

The substrate (starting material) was dissolved with PBS buffer (pH 6.0) in a 15 mL centrifuge tube. After the addition of appropriate amount (30 μg fucosidase protein per mg substrate) of fucosidase, the reaction mixture was incubated in an incubator shaker at 37 °C overnight with shaking at 300 rpm. The reaction mixture was centrifuged over a Nanosep® Omega ultrafiltration device (10 kDa MWCO) to remove proteins, and the filtrate was lyophilized. The residue was applied to P2 or P6 size-exclusion column chromatography using NH_4HCO_3 buffer (50 mM) as eluent to providing the desired product after lyophilization of the appropriate fractions.

General procedure for Cbz deprotection from the linker with $Pd(OH)_2$ reduction

Palladium hydroxide on carbon (Degussa type, 20%, 1.5 times the weight of starting material) was added to a solution of starting material in H_2O (0.1% AcOH as additive). The mixture was placed under an atmosphere of hydrogen until ESI-LC-MS indicated completion of the reaction. The mixture was filtered through a spin filter and the residue was washed with H_2O. The filtrate was lyophilized to give the final product. P6 size-exclusion column chromatography was used for purification using 50 mM ammonium bicarbonate as eluent. Fractions containing compound were lyophilized to give the desired product.

3) General Protocols for HILIC-HPLC Purification

Semi-preparative HILIC-HPLC was applied on a Shimadzu (LC-20AT, SIL-20A, CBM-20A, SPD-20A, FRC-10A) LC-ESI-IT-TOF with a XBridge HILIC column, 5 µm, 10 x 250 mm at a flow rate of 3.6 mL/min, injection volume of 100 µL (10-20 mg/mL), with 0.2% of the flow is diverted to the ESI-MS detector using a splitter. The purification was performed using 10% 10 mM NH_4HCO_3 in MeCN (buffer B) and MeCN in 80% 10 mM NH_4HCO_3 (buffer A).

General conditions for a linear gradient were used as the eluent:

Linear glycan **1-43, S1-S26**

Time (min)	A (%)	B (%)
0	10	90
90	50	50

N-glycan **44-54**

Time (min)	A (%)	B (%)
0	20	80
60	45	55
70	45	55

4) Analytical Data

NMR nomenclature

NMR data was obtained at room temperature on a 600 MHz instrument from Bruker. The chemical shift δ is given in parts per million (ppm) and refers to tetramethyl silane and the residual solvent peak [^{1}H-NMR: δ(D_2O) =4.79 ppm]. NMR data is given as follows: ^{1}H-NMR: chemical shift (multiplicity, coupling constants, relative integral, functional group); ^{13}C data are extracted from HSQC spectra and given as follows: chemical shift. Multiplicity is defined as follows: s = singlet; d = doublet; t = triplet; m= multiplet. Signals were assigned by numbering the monosaccharide units starting at the reducing end of the oligosaccharide. Monosaccharides attached to the mannose-3 branch are indicated by a“ ’ “ (prime) and those attached to the mannose-2 branch without any mark. The assignment was performed by using 2D-NMR spectra (COSY, HSQC, TOCSY, NOESY). The yields of the final products were determined gravimetrically and confirmed by NMR spectroscopy using n-propanol as an internal standard. High resolution masses were measured on an Agilent 6560 Ion Mobility Q-TOF LC-MS system.

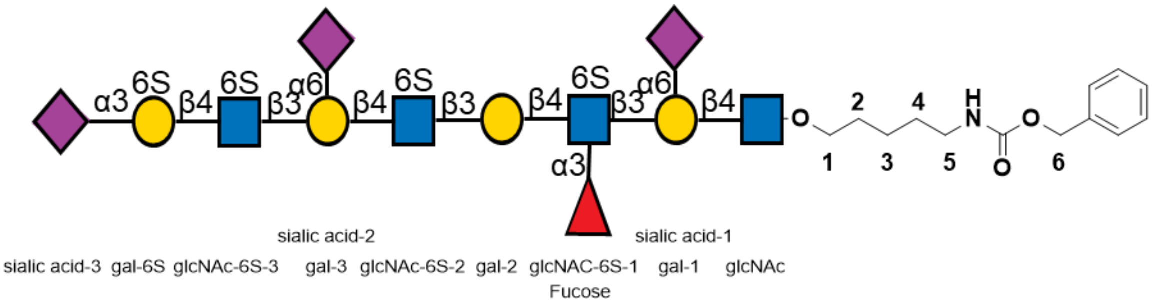

and

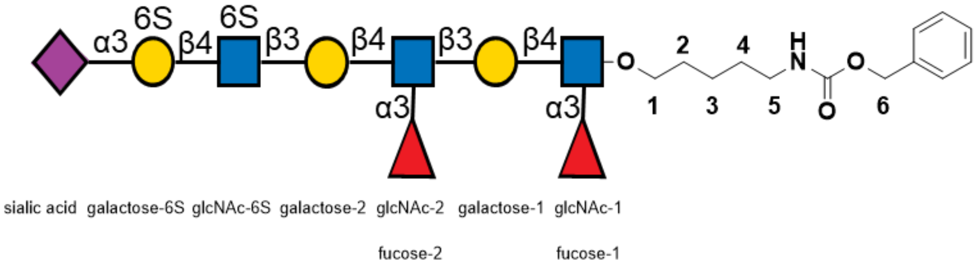

Labelling of linear glycans for NMR peak assignment

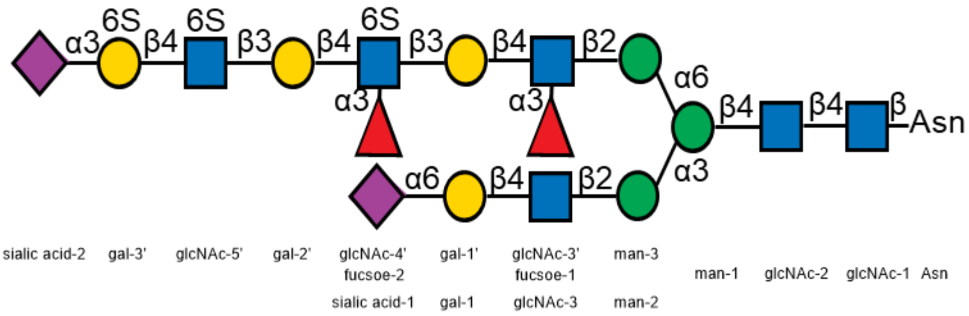

Labelling of *N*-glycans for NMR peak assignment

Determination of GlcNAc-6-sulfation, galactose-6-sulfation, α2,3-sialylation, α2,6-sialylation and α1,3-fucosylation position

Characterization of Compound 7

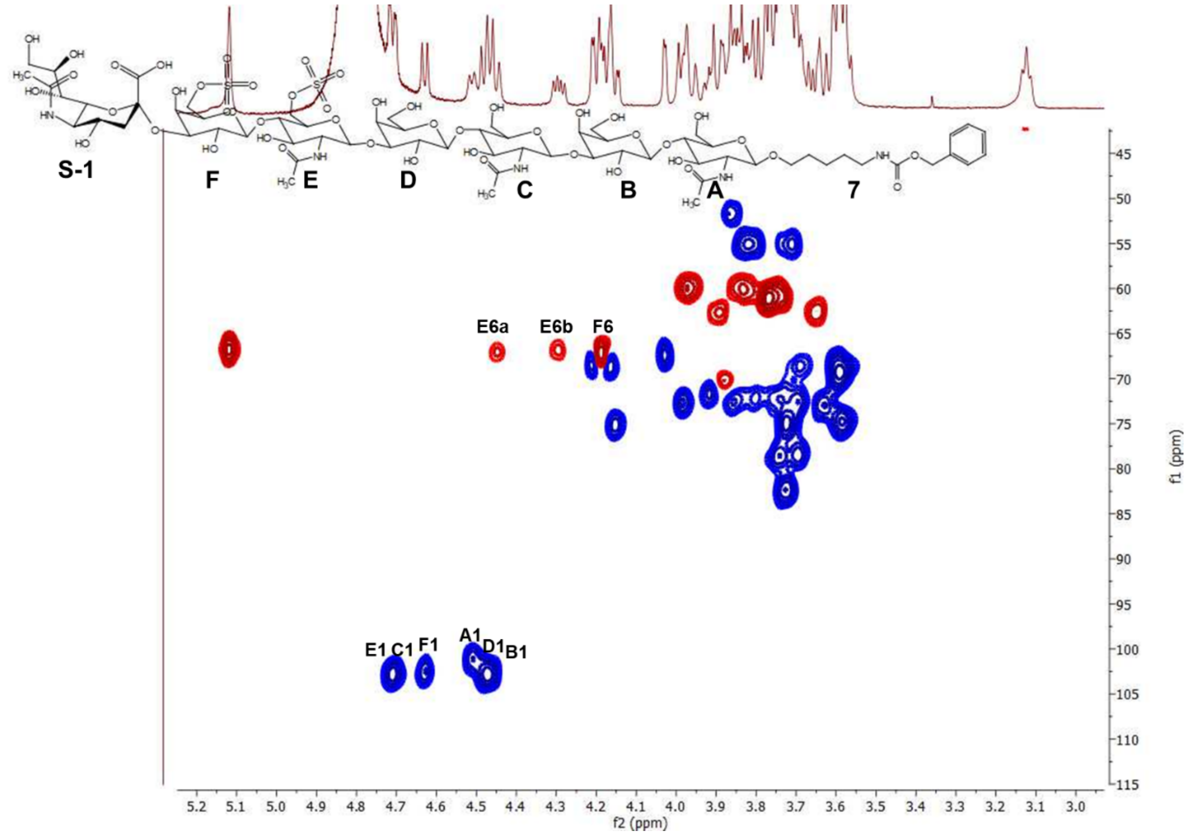

Figure S1a. 600 MHz 1D ^{1}H NMR and 2D ^{13}C-^{1}H HSQC spectra of **7**, recorded at 298K in D_2O.

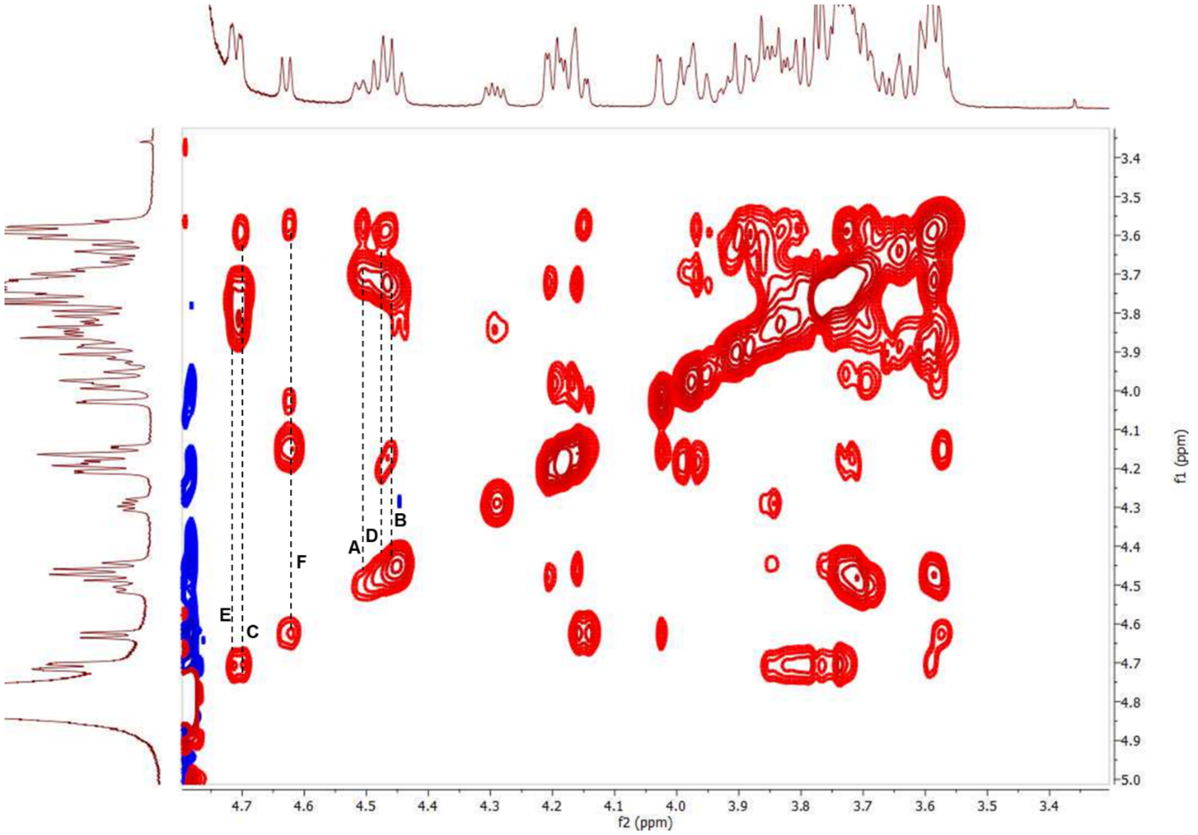

Figure S1b. 2D TOCSY (80 ms) spectrum of **7**.

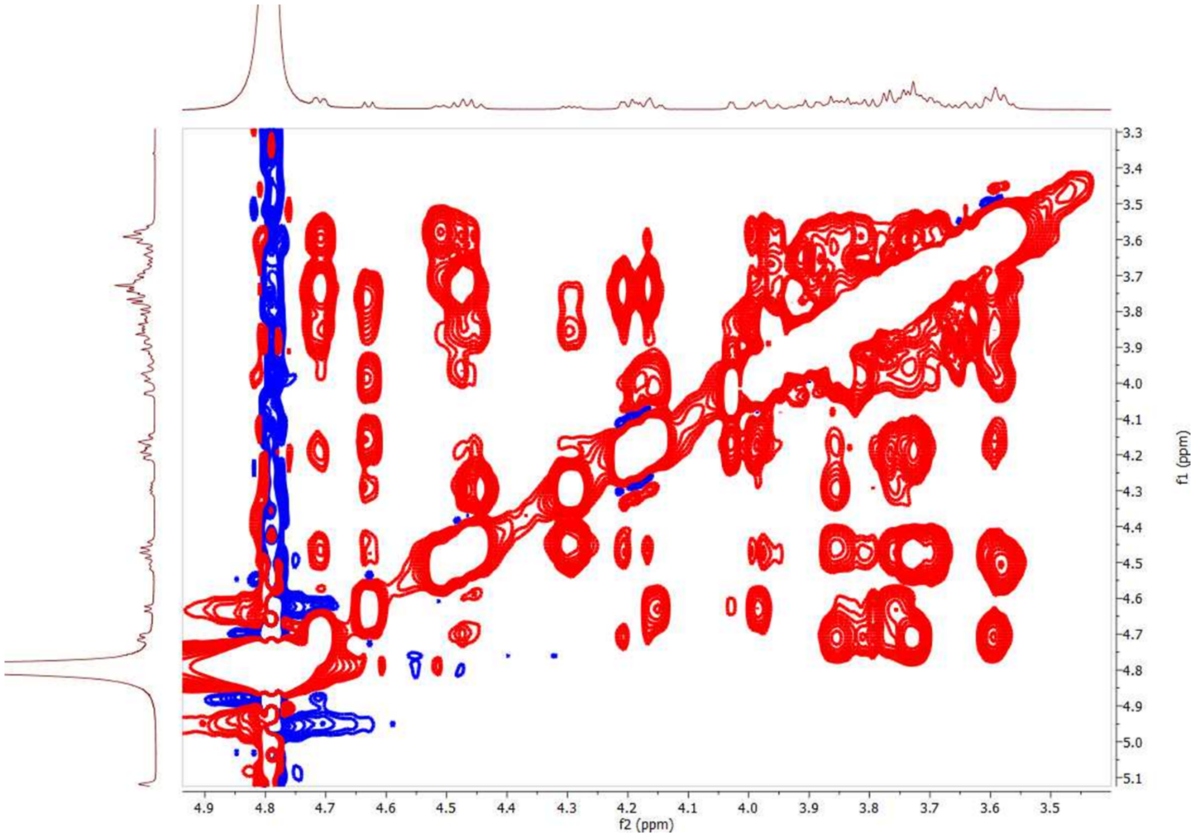

Figure S1c. 600 MHz 1D ^{1}H NMR and 2D NOESY (300 ms) spectra of **7**.

Detailed NMR analysis confirmed the GlcNAc and galactose sulfation position of compound **7**. For example, 1D ^{1}H NMR and 2D ^{13}C–^{1}H HSQC spectra of compound **7** made it possible to assign all proton and carbon signals. The 6-carbon of the internal sulfated GlcNAc moiety had substantially shifted downfield (δ 60.1 → δ 66.9) and the corresponding protons also exhibited a chemical shift difference (H6a δ 3.97 → 4.45, H6b 3.84 → 4.29), which confirmed the regioselectivity of GlcNAc sulfation. The 6-carbon of the internal sulfated galactose moiety had substantially shifted downfield (δ 61.3 → δ 67.3) and the corresponding protons also exhibited a chemical shift difference (H6 δ 3.76 → 4.19), which confirmed the regioselectivity of galactose sulfation. The inter-residue connectivity was confirmed by a NOESY spectrum, the inter-residue connectivities Gal-F H-1, GlcNAc-E H-4, GlcNAc-E H-1, Gal-D H-3, Gal-D H-1, GlcNAc-C H-4, GlcNAc-C H-1, Gal-B H-3 and Gal-B H-1, GlcNAc-A H-4 are in accordance with F(1→4)E, E(1→3)D, D(1→4)C, C(1→3)B, B(1→4)A linkages, respectively.

Characterization of Compound 14

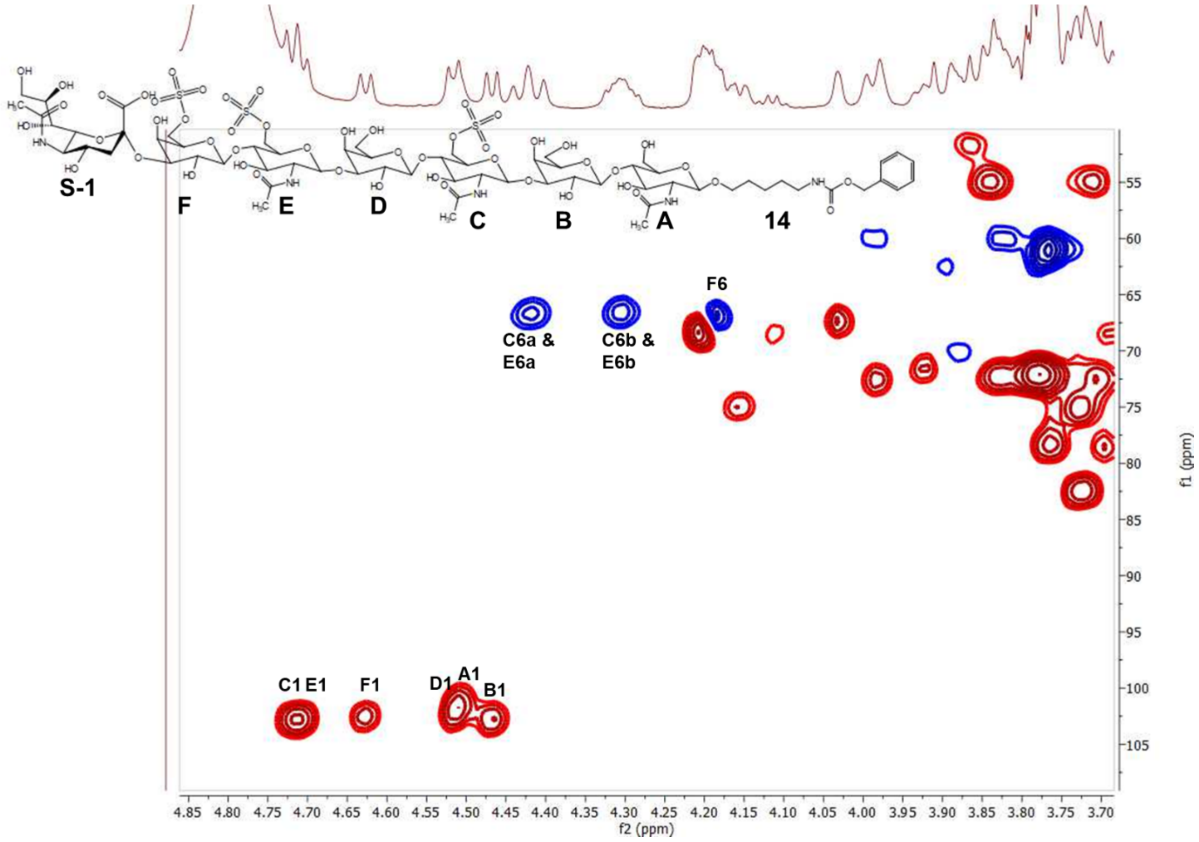

Figure S2a. 600 MHz 1D ^{1}H NMR and 2D ^{13}C-^{1}H HSQC spectra of **14**, recorded at 298K in D_2O.

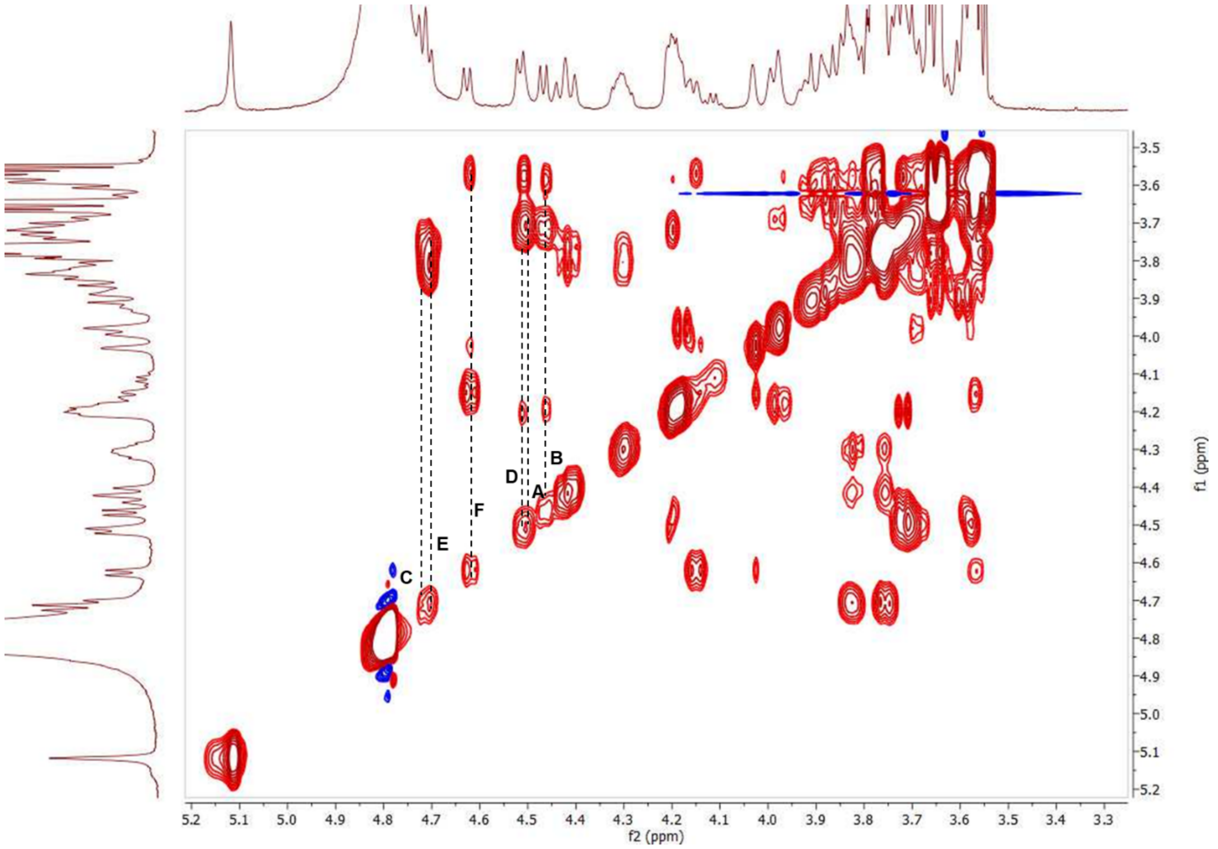

Figure S2b. 2D TOCSY (80 ms) spectrum of **14**.

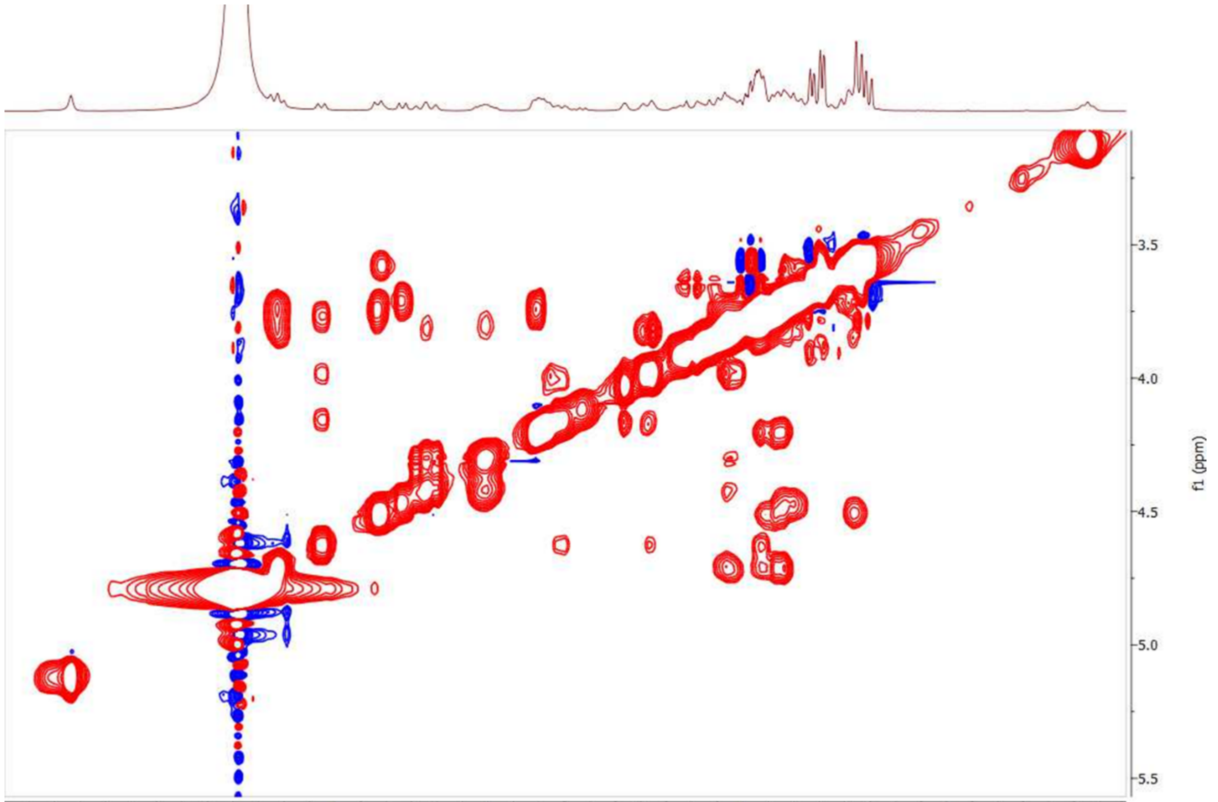

Figure S2c. 600 MHz 1D ^{1}H NMR and 2D NOESY (300 ms) spectra of **14**.

Detailed NMR analysis confirmed the GlcNAc and galactose sulfation position of compound **14**. For example, 1D ^{1}H NMR and 2D ^{13}C–^{1}H HSQC spectra of compound **14** made it possible to assign all proton and carbon signals. The 6-carbon of the two internal sulfated GlcNAc moieties had substantially shifted downfield (δ 60.1 → δ 66.7) and the corresponding protons also exhibited a chemical shift difference (H6a δ 3.97 → 4.42, H6b 3.84 → 4.30), which confirmed the regioselectivity of GlcNAc sulfation. The 6-carbon of the internal sulfated galactose moiety had substantially shifted downfield (δ 61.3 → δ 66.8) and the corresponding protons also exhibited a chemical shift difference (H6 δ 3.76 → 4.18), which confirmed the regioselectivity of galactose sulfation. The inter-residue connectivity was confirmed by a NOESY spectrum, the inter-residue connectivities Gal-F H-1, GlcNAc-E H-4, GlcNAc-E H-1, Gal-D H-3, Gal-D H-1, GlcNAc-C H-4, GlcNAc-C H-1, Gal-B H-3 and Gal-B H-1, GlcNAc-A H-4 are in accordance with F(1→4)E, E(1→3)D, D(1→4)C, C(1→3)B, B(1→4)A linkages, respectively.

Characterization of Compound 39

The assignments of GlcNAc-6-sulfation, Galactose-6-sulfation, α2,3-sialylation, α2,6-sialylation and fucosylation were confirmed by a combination of ^{1}H, COSY, NOESY, TOCSY and HSQC NMR experiments. Compound **39** was shown as a standard example.

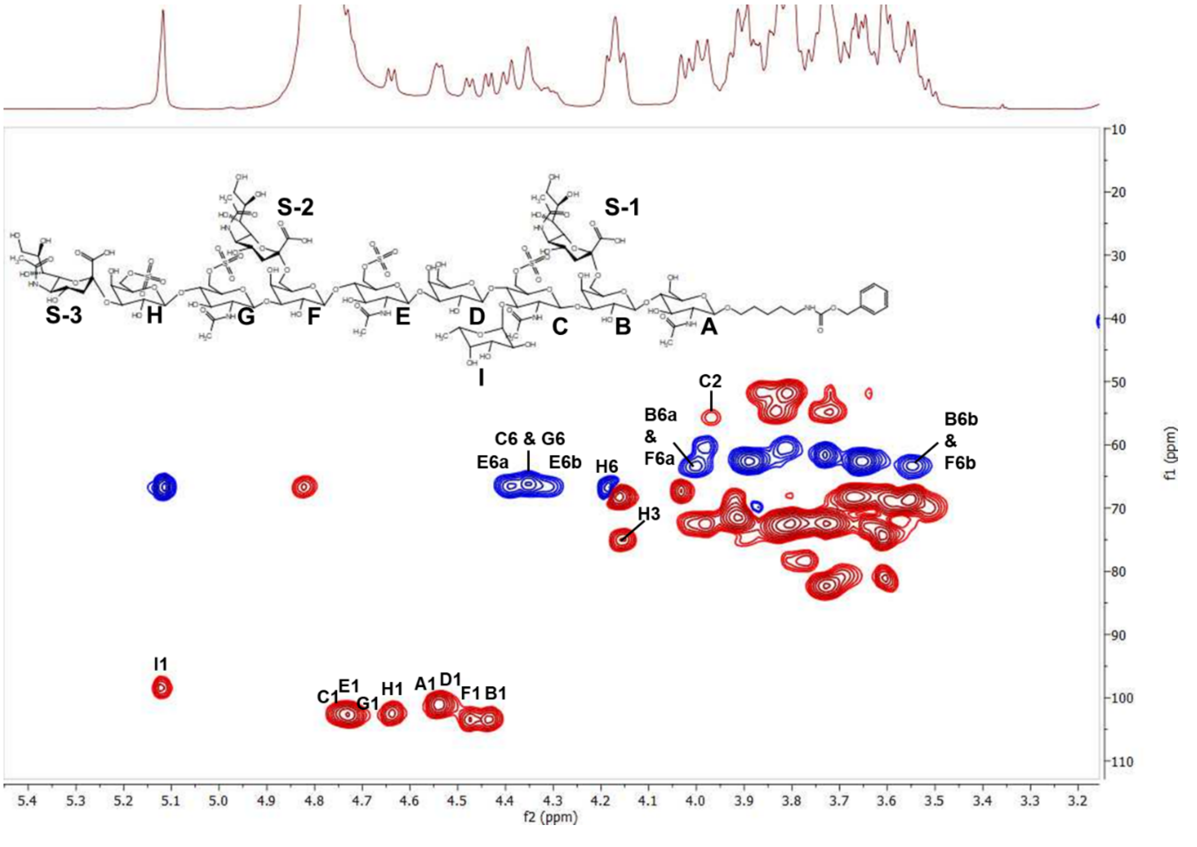

Figure S3a. 600 MHz 1D ^{1}H NMR and 2D ^{13}C-^{1}H HSQC spectra of **39**, recorded at 298K in D_2O.

The 1D ^{1}H NMR and 2D ^{13}C–^{1}H HSQC spectra of **39** are depicted in Figure S1a. The assignments of all ^{1}H and ^{13}C resonances are presented in table for compound **39**. The 1D ^{1}H NMR spectrum of **39** shows nine anomeric signals, correlated with residues **A**, **B**, **C**, **D**, **E**, **F**, **G**, **H**, and **I**. The H-1 signal at δH 4.55 (**A**) is stemming from a reducing-end GlcNAc residue. whereas the H-1 signal at δH 4.44 (**B**) belongs to non-reducing βGal, the anomeric signal at δH 4.75 (**C**) belongs to non-reducing βGlcNAc, respectively. The two H-1 signals at δH 4.54 (**D**) and δH 4.73 (**E**) are stemming from a non-reducing βGal and non-reducing βGlcNAc residue, whereas the H-1 signal at δH 4.48 (**F**) belong to non-reducing βGal. The anomeric signals at δH 4.72 (**G**), δH 4.64 (**H**), δH 5.12 (**I**), belong to βGlcNAc, βGal, αFucose, respectively.

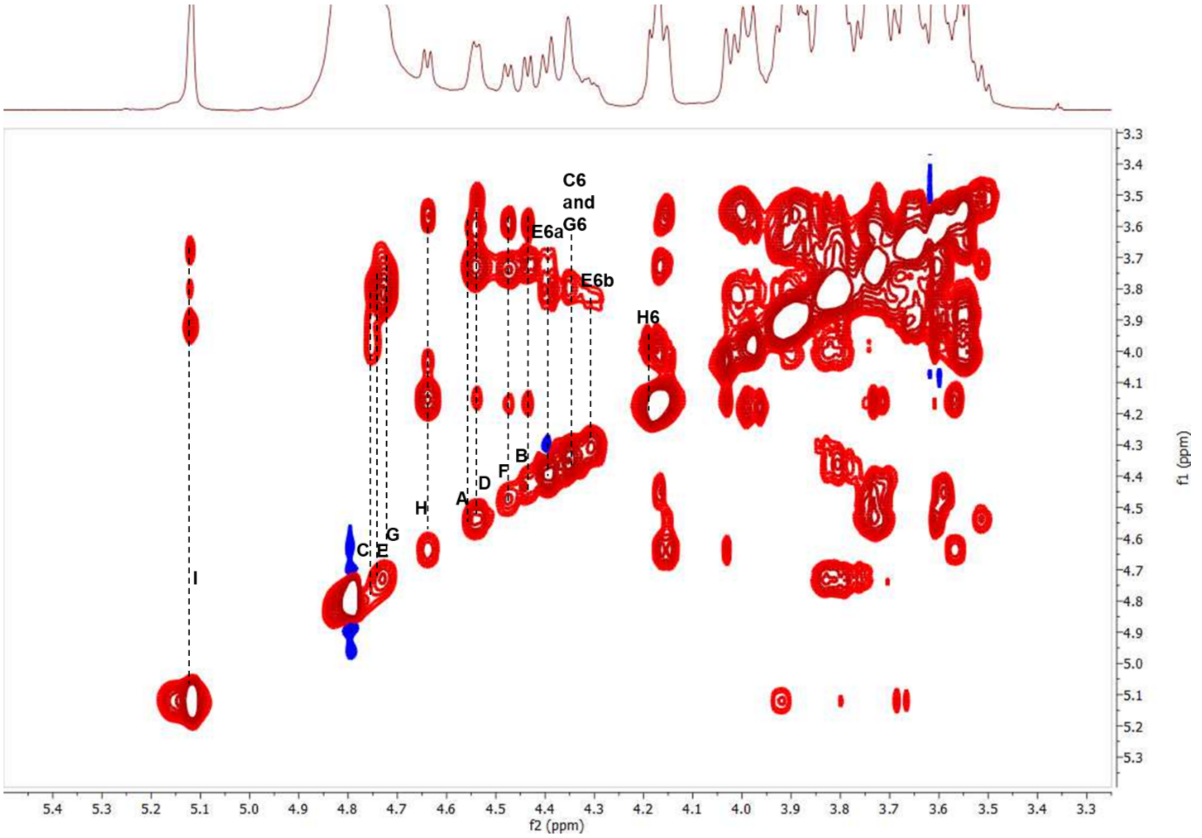

Figure S3b. 2D TOCSY (80 ms) spectrum of **39**.

In the TOCSY spectrum (80 ms, Figure S1b), the H-1 tracks of **A**, **C**, **E**, **G** show spin systems H-1,2,3,4,5 typical for βGlcNAc residues. the H-1 tracks of **B**, **D**, **F**, **H** show spin systems H-1,2,3,4, typical for βGal residues. the H-1 track of **I** shows spin systems H-1,2,3,4,5 typical for αFucose residues. In combination with HSQC data, the H-6 from **A** to **I** signals were identified. Finally, the H-6 tracks of **A**, **C**, **E**, **F**, **G**, **H**, **I** allowed the observation of cross-peaks with H-5 whereas the cross-peaks for H-1 were also detected on the H-6a,6b tracks. In combination with HSQC data, the remaining signals were identified.

For Gal-**B**/**F**-α2,6 Neu5Ac the HSQC spectrum (Figure S1a) containing proton-carbon correlations and the substitution information for the sialylation, showed $–CH_2–$ type group and downfield shifts for Gal-**B**6 and Gal-**F**6 [δC-6 63.5] from (δ 61.1), the corresponding protons shifted from (H6 δ 3.76) to (H6a δ 4.00) and (H6b δ 3.55), indicating the involvement of this carbon and proton in sialic acid linkages.

For Gal-**H**-α2,3-Neu5Ac, the HSQC spectrum (Figure S1a) containing proton-carbon correlations and the substitution information for the sialylation, showed –CH– type group and the corresponding protons shifted from (H3 δ 3.72) to (H3 δ 4.16), indicating the involvement of this proton in sialic acid linkages. The nearby Gal-**H** H-1 also shifts from (H1 δ 4.55) to (H1 δ 4.64), which indicative of the involvement of this proton in nearby sialic acid linkages.

For GlcNAc-**C**-α1,3-Fucose, the HSQC spectrum (Figure S1a) containing proton-carbon correlations and the substitution information for the fucosylation, showed –CH– type group and the corresponding protons shifted from (H3 δ 3.77) to (H3 δ 3.90) indicating the involvement of this proton in fucosyl linkages. The nearby GlcNAc-**C** H-2 also shifted from (H2 δ 3.83) to (H2 δ 3.97), which indicative of the involvement of this proton in nearby fucosyl linkages. The nearby GlcNAc-**C** H-4 also shifts from (H4 δ 3.78) to (H4 δ 4.01), which indicates the involvement of this proton in nearby fucosylated linkages.

For GlcNAc-**C**/**E**/**G**-6S, the HSQC spectrum (Figure S1a) containing proton-carbon correlations and the substitution information for the sulfation, showed –CH_2– type group and downfield shifts for GlcNAc-**C**6 [δC-6 66.3], GlcNAc-**E**6 [δC-6 66.6], GlcNAc-**G**6 [δC-6 66.3] from (δ 60.5), the corresponding protons shifted from (H6a δ 3.98) and (H6b δ 3.82) to (H6 δ 4.35) (GlcNAc-**C**), (H6 δ 4.35) (GlcNAc-**G**) and (H6a δ 4.40), (H6b δ 4.30) (GlcNAc-**E**), indicating the involvement of this carbon and proton in sulfation.

For Gal-**H**-6S, the HSQC spectrum (Figure S1a) containing proton-carbon correlations and the substitution information for the sulfation, showed –CH_2– type group and downfield shifts for Gal-**H**6 [δC-6 66.7] from (δ 60.9), the corresponding protons shifted from (H6 δ 3.74) to (H6 δ 4.18), indicating the involvement of this carbon and proton in sulfated linkages. The nearby Gal-**H** H-3 also shifts from (H3 δ 4.13) to (H3 δ 4.16), which indicating the involvement of this proton in nearby sulfated linkages. The nearby Gal-**H** H-4 also shifts from (H4 δ 3.98) to (H4 δ 4.03), which indicating the involvement of this proton in nearby sulfation.

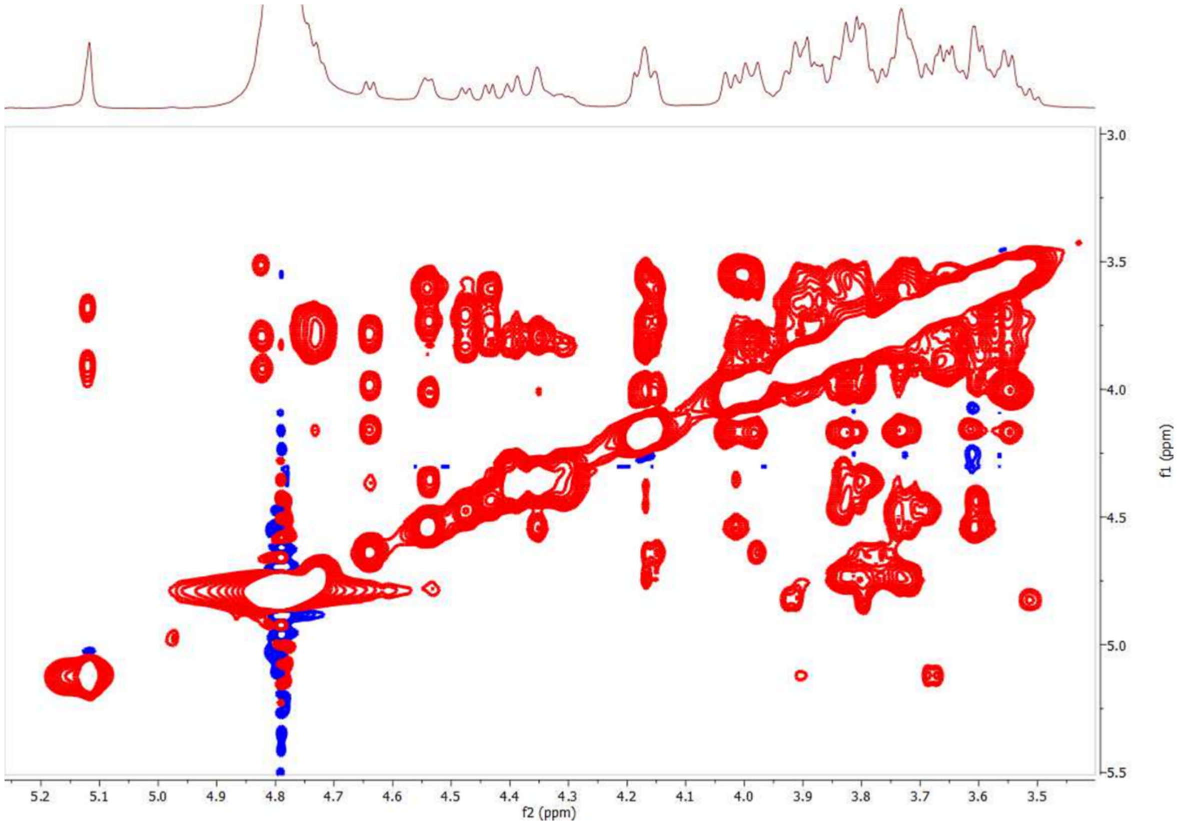

Figure S3c. 600 MHz 1D ^{1}H NMR and 2D NOESY (300 ms) spectra of **39**.

In the 2D NOESY spectrum (300 ms, Figure S1c), the inter-residue connectivities Gal-**H** H-1 to GlcNAc-**G** H-4, GlcNAc-**G** H-1 to Gal-**F** H-3, Gal-**F** H-1 to GlcNAc-**E** H-4, GlcNAc-**E** H-1 to Gal-**D** H-3, Gal-**D** H-1 to GlcNAc-**C** H-4, GlcNAc-**C** H-1 to Gal-**B** H-3 and Gal-**B** H-1 to GlcNAc-**A** H-4 are overlapped but based on overlapped spots still in accordance with **H**(1→4)**G**, **G**(1→3)**F**, **F**(1→4)**E**, **E**(1→3)**D**, **D**(1→4)**C**, **C**(1→3)**B** and **B**(1→4)**A** linkages, respectively.

Characterization of Compound 40

The assignments of GlcNAc-6-sulfation, Galactose-6-sulfation, α2,3-sialylation and fucosylation were confirmed by a combination of ^{1}H, COSY, NOESY, TOCSY and HSQC NMR experiments. Data analysis of compound **40** is shown as an example.

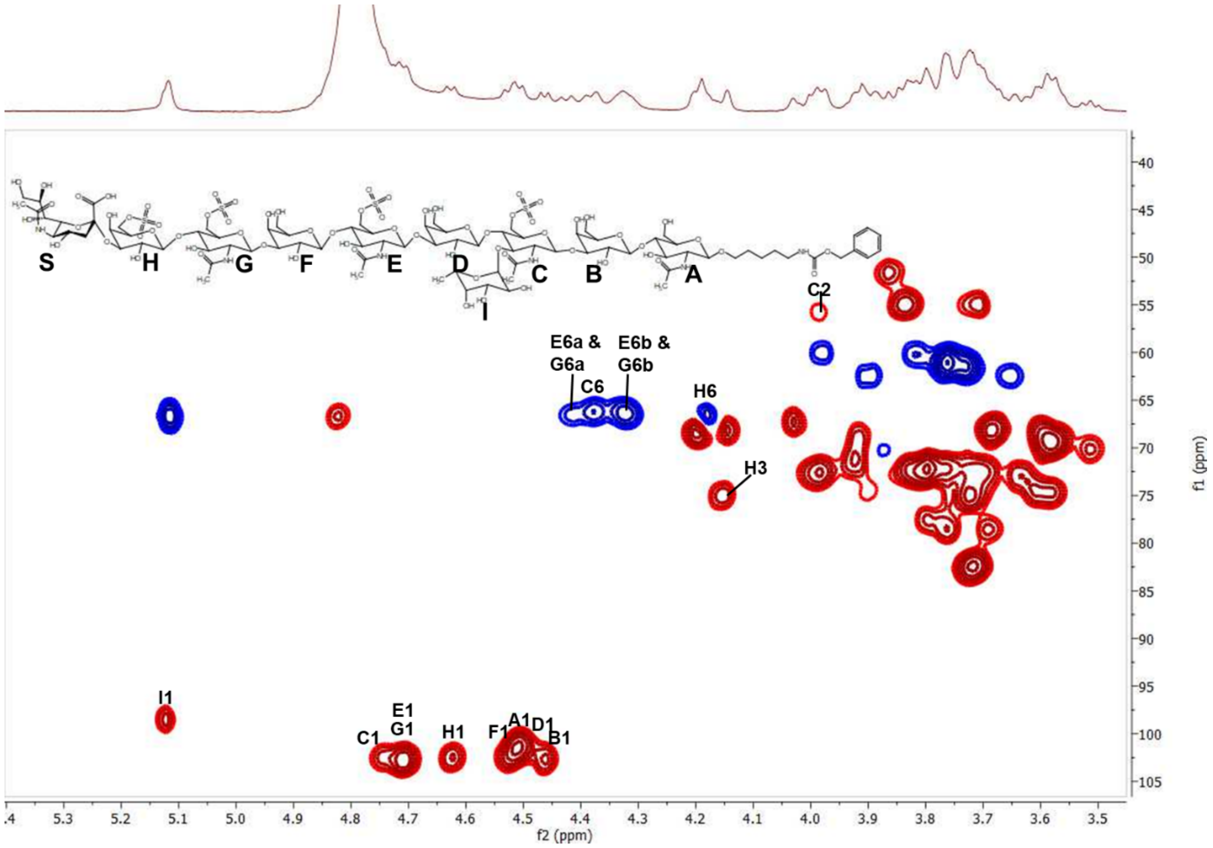

Figure S4a. 600 MHz 1D ^{1}H NMR and 2D ^{13}C-^{1}H HSQC spectra of **40**, recorded at 298K in D_2O.

The 1D ^{1}H NMR and 2D ^{13}C–^{1}H HSQC spectra of **40** are depicted in Figure S2a. The assignments of all ^{1}H and ^{13}C resonances are presented in the table for compound **40**. The 1D ^{1}H NMR spectrum of **40** shows nine anomeric signals, correlating with residues **A**, **B**, **C**, **D**, **E**, **F**, **G**, **H**, and **I**. The H-1 signal at δH 4.51 (**A**) is stemming from the reducing-end GlcNAc residue whereas the H-1 signal at δH 4.46 (**B**) belongs to non-reducing βGal. The anomeric signal at δH 4.75 (**C**) belongs to non-reducing βGlcNAc, respectively. The two H-1 signals at δH 4.50 (**D**) and δH 4.71 (**E**) are stemming from the non-reducing βGal and non-reducing βGlcNAc residue, whereas the H-1 signal at δH 4.53 (**F**) belongs to non-reducing βGal. The anomeric signals at δH 4.71 (**G**), δH 4.63 (**H**), δH 5.12 (**I**) belong to βGlcNAc, βGal, αFucose, respectively.

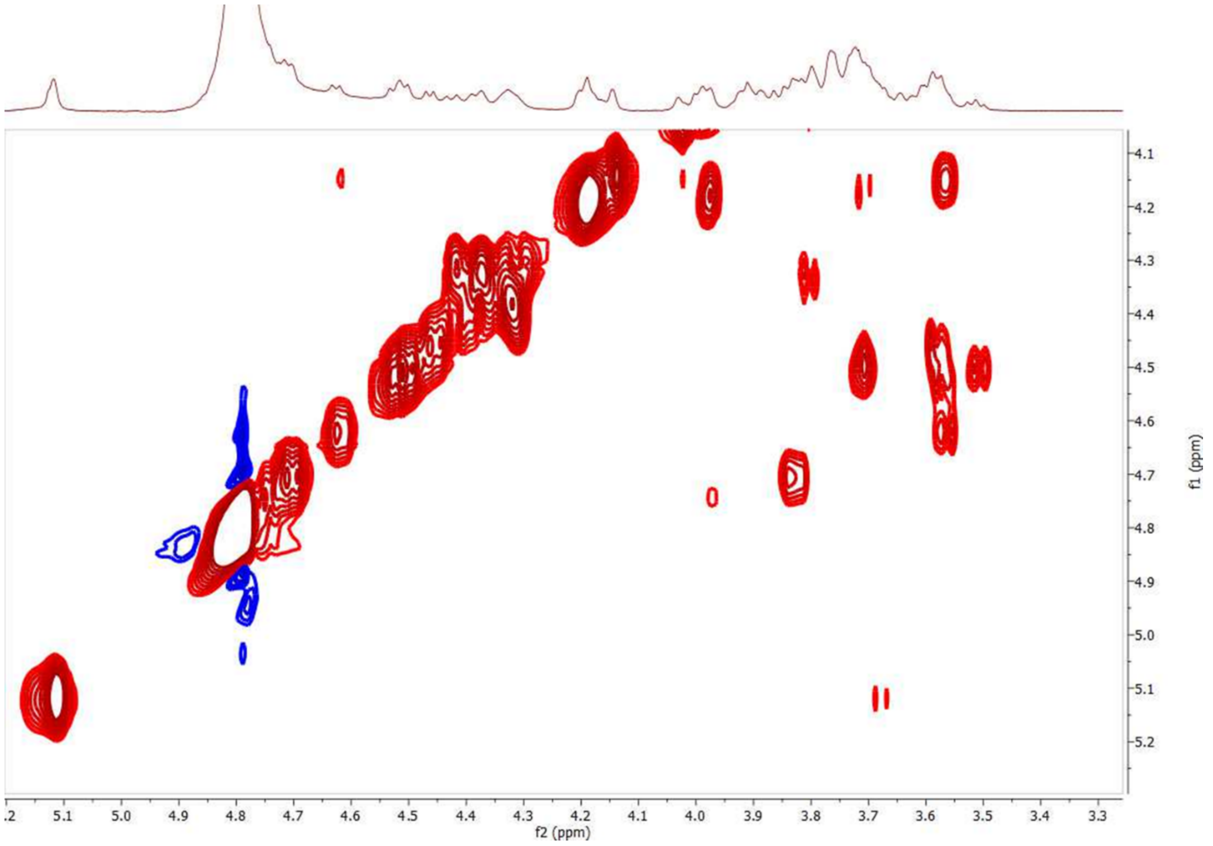

Figure S4b. 2D TOCSY (20 ms) spectrum of **40**.

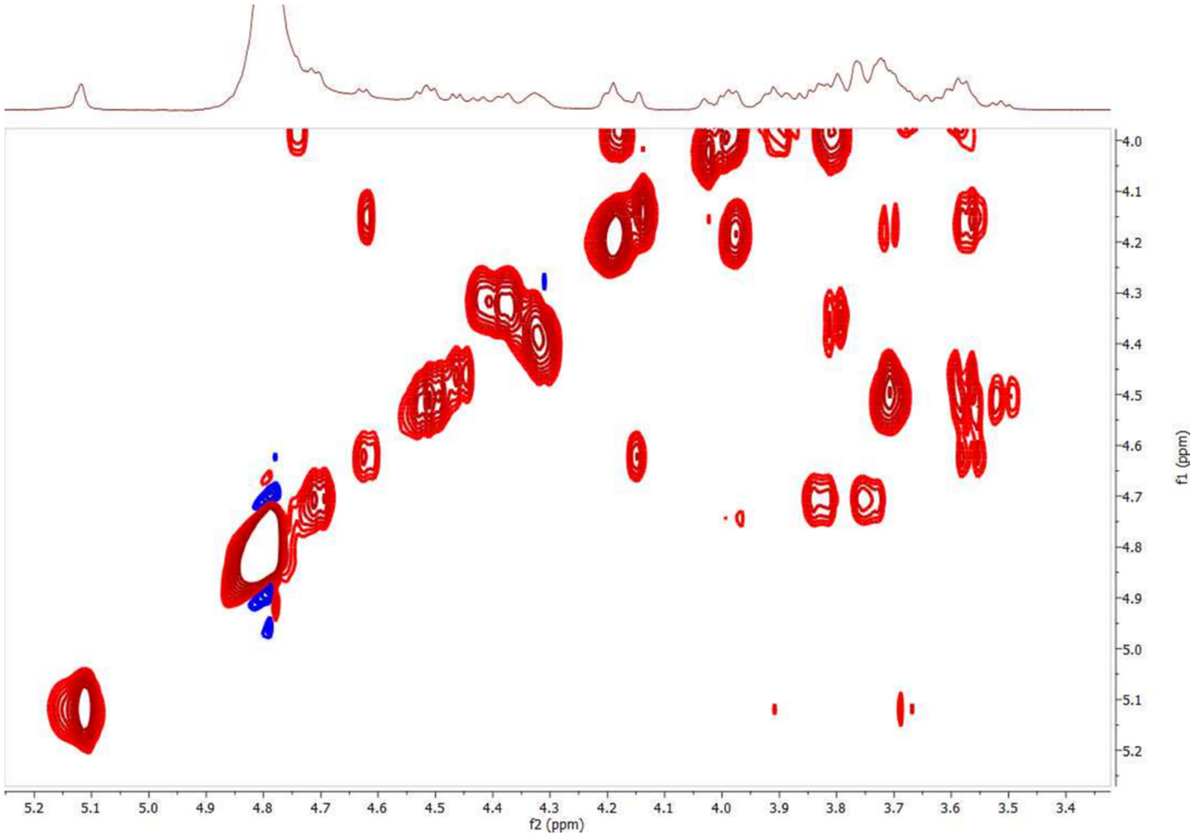

Figure S4c. 2D TOCSY (40 ms) spectrum of **40**.

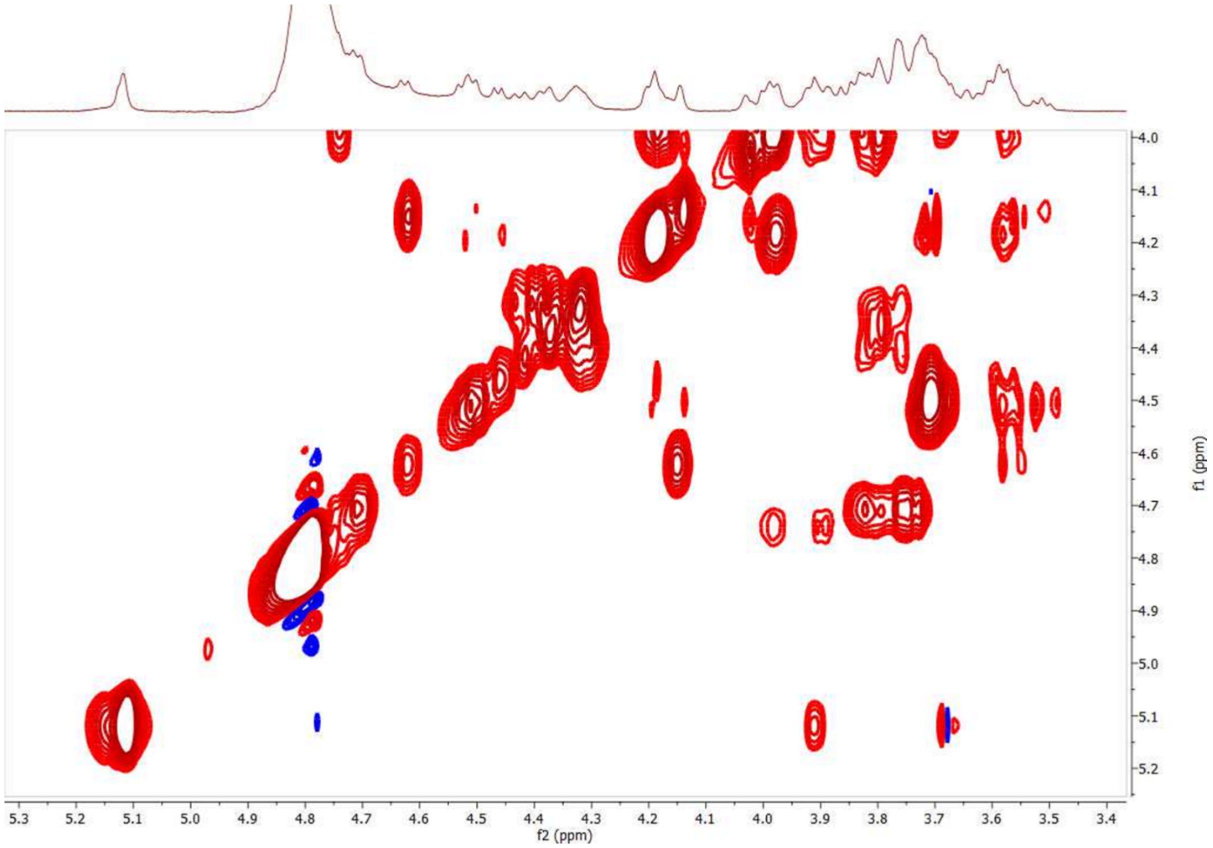

Figure S4d. 2D TOCSY (60 ms) spectrum of **40**.

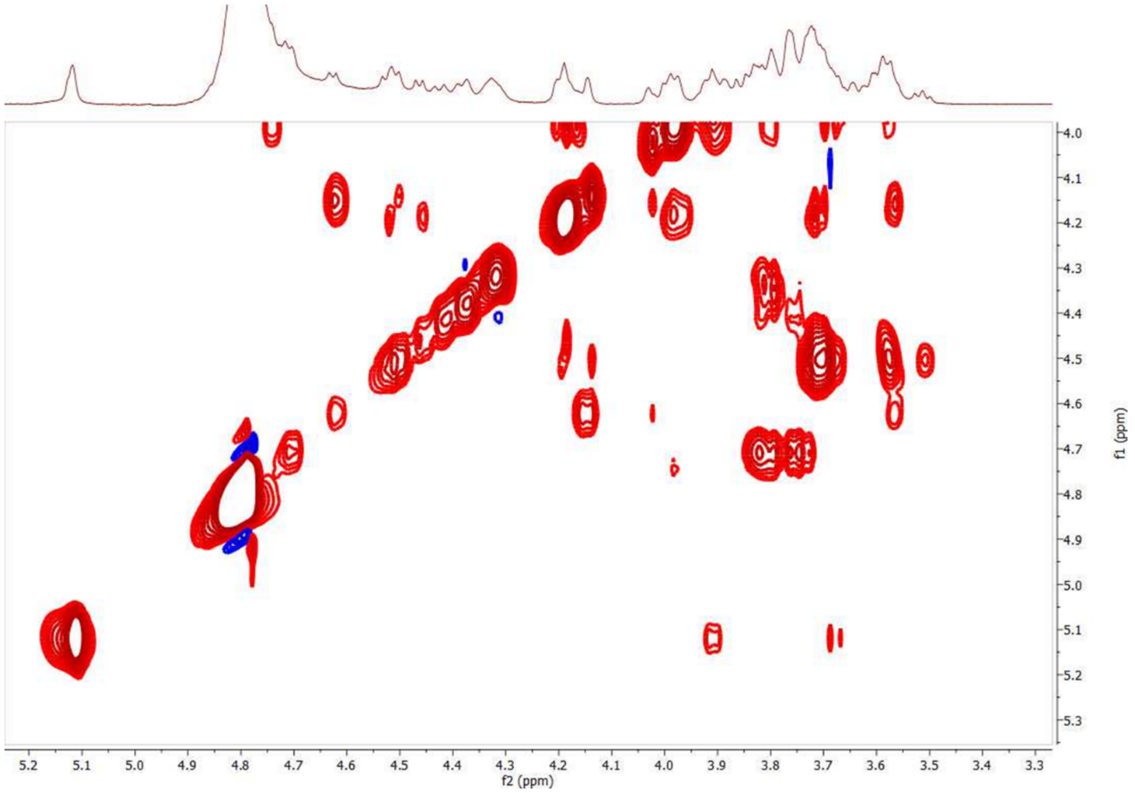

Figure S4e. 2D TOCSY (80 ms) spectrum of **40**.

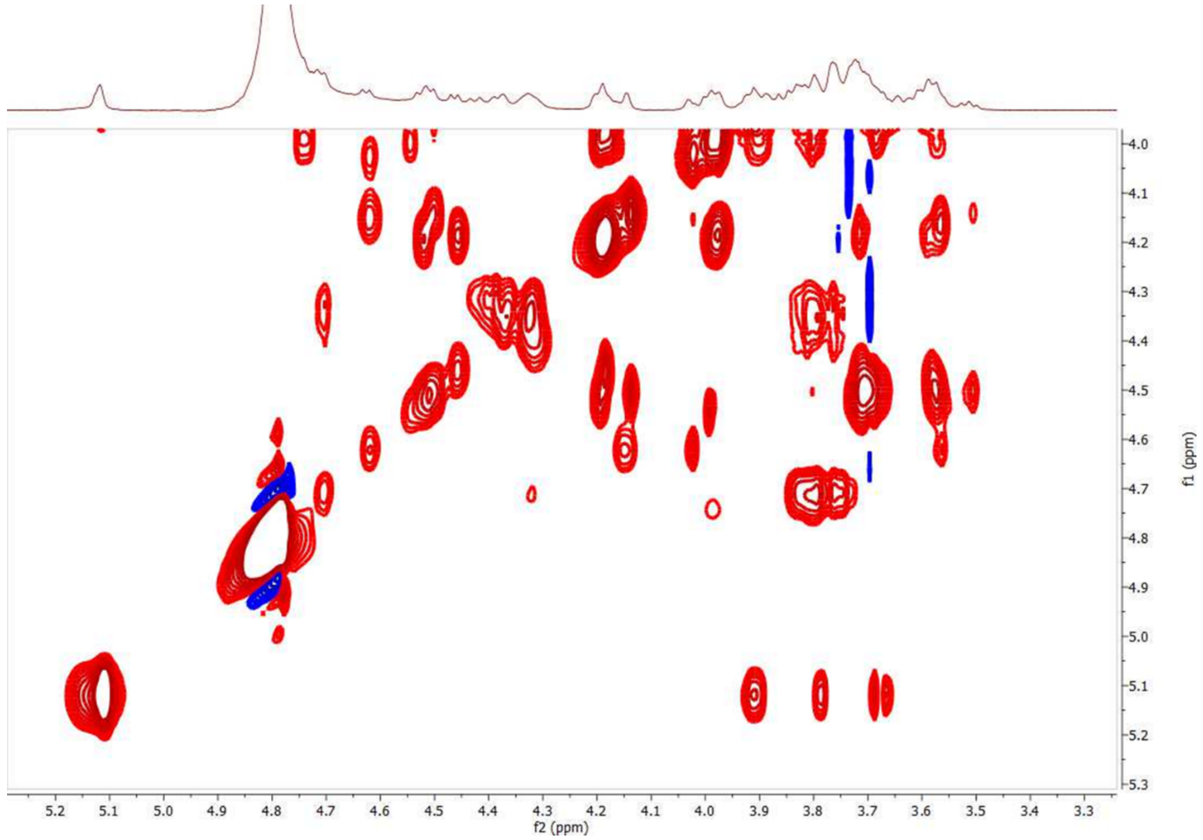

Figure S4f. 2D TOCSY (150 ms) spectrum of **40**.

From the TOCSY spectrum (20 ms to 150 ms, Figure 2b-f), the H-1 tracks of **A**, **C**, **E**, **G** show spin systems H-1,2,3,4,5,6 typical for βGlcNAc residues. The H-1 tracks of **B**, **D**, **F**, **H** show spin systems H-1,2,3,4, typical for βGal residues. The H-1 track of I shows spin systems H-1,2,3,4,5,6 typical for αFucosyl residues. In combination with HSQC data, the H-6 from **A** to **I** signals were fully identified. Finally, the H-6 tracks of H allowed the observation of cross-peaks with H-5. In combination with HSQC data, the remaining signals were identified.

For Gal-**H**-α2,3-Neu5Ac, the HSQC spectrum (Figure S2a) containing proton-carbon correlations and the substitution information for the sialylation, showed –CH– type group and the corresponding protons shifted from (H3 δ 3.72) to (H3 δ 4.16), indicating the involvement of this proton in the sialic acid linkages. The nearby Gal **H** H-1 also shifts from (H1 δ 4.55) to (H1 δ 4.63), which indicates the involvement of this proton in nearby sialic acid linkages. For Gal-**H**-6S, the HSQC spectrum (Figure 3b) containing proton-carbon correlations and the substitution information for the sulfation, showed –CH_2– type group and downfield shifts for Gal-**H**6 [δC-6 66.5] from (δ 60.9), the corresponding protons shifted from (H6 δ 3.74) to (H6 δ 4.18), indicating the involvement of this carbon and proton in sulfated linkages.

For GlcNAc-**C**-α1,3-Fucose, the HSQC spectrum (Figure S2a) containing proton-carbon correlations and the substitution information for the fucosylation, showed –CH– type group and the corresponding protons shifted from (H3 δ 3.77) to (H3 δ 3.90) indicating the involvement of this proton in fucosylated linkages. The nearby GlcNAc **C** H-2 also shifts from (H2 δ 3.83) to (H2 δ 3.99), which indicating the involvement of this proton in nearby

fucosylated linkages. The nearby GlcNAc **C** H-4 also shifts from (H4 δ 3.78) to (H4 δ 4.01), which indicating the involvement of this proton in nearby fucosylated linkages.

For GlcNAc-**C**/**E**/**G**-6S, the HSQC spectrum (Figure S2a) containing proton-carbon correlations and the substitution information for the sulfation, showed –CH_2– type group and downfield shifts for GlcNAc-**C**6 [δC-6 66.4], GlcNAc-**E**6 [δC-6 66.5], GlcNAc-**G**6 [δC-6 66.5] from (δ 60.2), the corresponding protons shifted from (H6a δ 3.98) and (H6b δ 3.81) to (H6 δ 4.36) (GlcNAc **C**), (H6a δ 4.41), (H6b δ 4.32) (GlcNAc **E**) and (H6a δ 4.41) (H6b δ 4.32) (GlcNAc **G**), indicating the involvement of this carbon and proton in sulfated linkages.

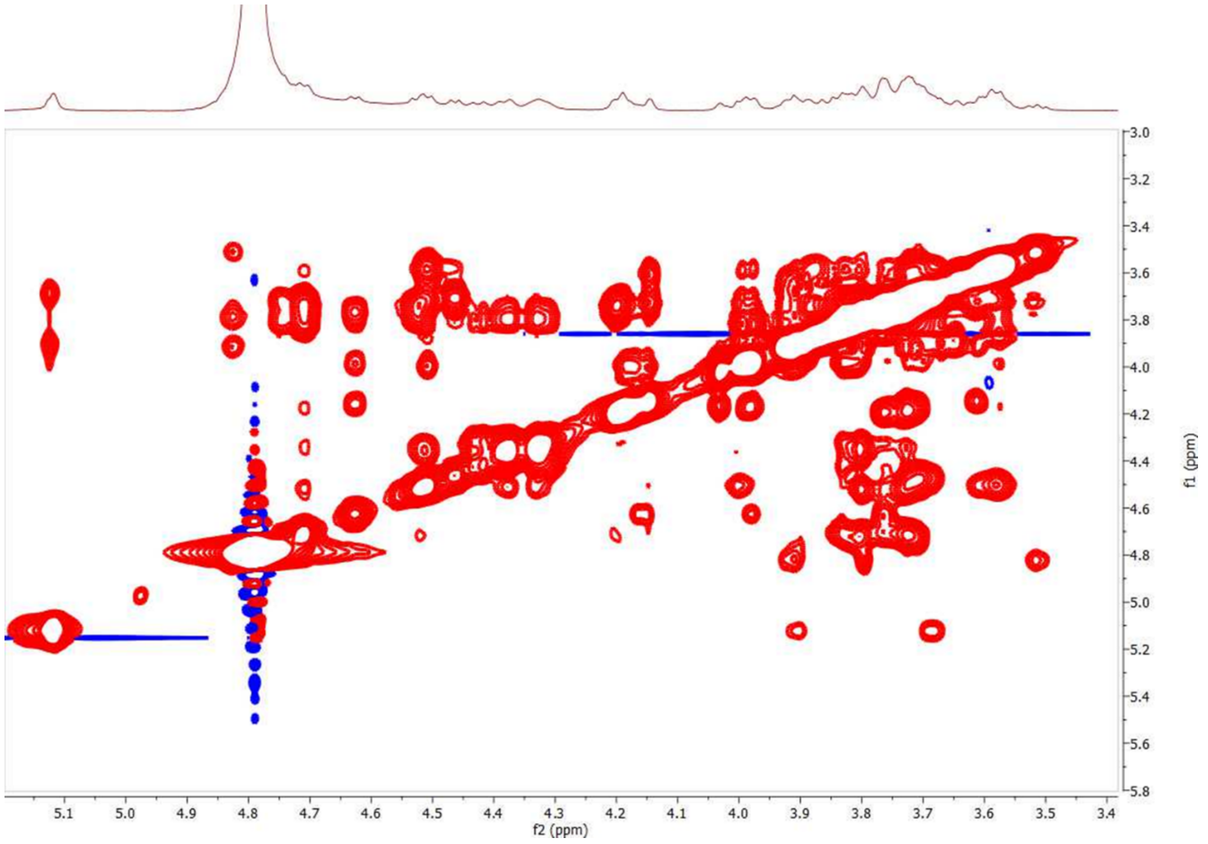

Figure S4g. 600 MHz 1D ^{1}H NMR and 2D NOESY (300 ms) spectra of **40**.

In the 2D NOESY spectrum (300 ms, Figure S2g), the inter-residue connectivities Gal-**H** H-1 to GlcNAc-**G** H-4, GlcNAc-**G** H-1 to Gal-**F** H-3, Gal-**F** H-1 to GlcNAc-**E** H-4, GlcNAc-**E** H-1 to Gal-**D** H-3, Gal-**D** H-1 to GlcNAc-**C** H-4, GlcNAc-**C** H-1 to Gal-**B** H-3 and Gal-**B** H-1 to GlcNAc-**A** H-4 are overlapped but based on overlapped spots still in accordance with **H**(1→4)**G**, **G**(1→3)**F**, **F**(1→4)**E**, **E**(1→3)**D**, **D**(1→4)**C**, **C**(1→3)**B** and **B**(1→4)**A** linkages, respectively.

Determination NMR of multiple negative charged compounds 26 and S23

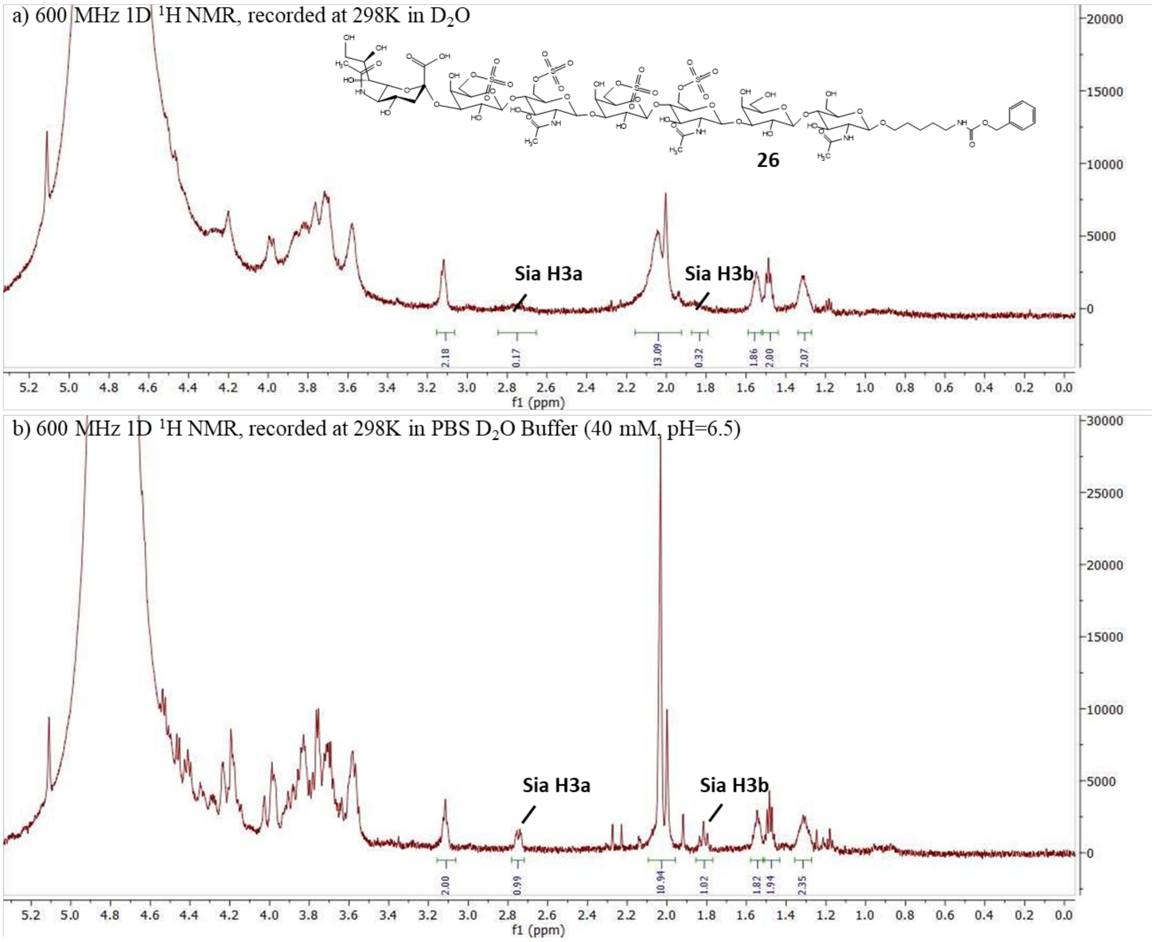

Figure S5. a) 600 MHz 1D ^{1}H NMR spectra (δ 0.0 to 5.3) of **26**, recorded at 298K in D_2O. b) 600 MHz 1D ^{1}H NMR spectra (δ 0.0 to 5.3) of **26**, recorded at 298K in PBS D2O buffer (40 mM, pH 6.5). full NMR in NMR spectra.

26 failed to provide well resolved NMR signals in D_2O. Well resolved NMR spectra were obtained in PBS D_2O buffer (40 mM, pH 6.5).

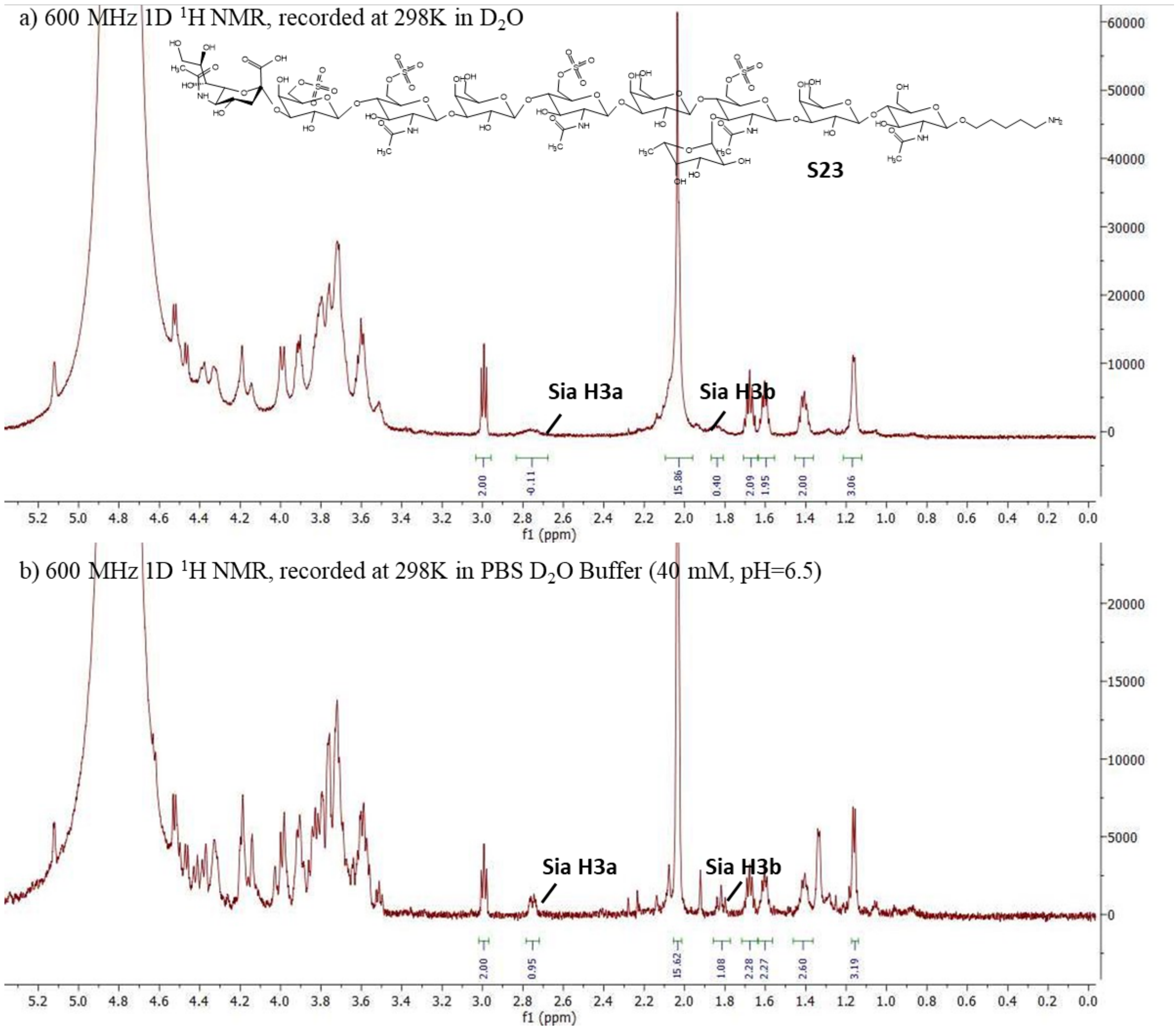

Figure S6. a) 600 MHz 1D ^{1}H NMR spectra (δ 0.0 to 5.3) of **S23**, recorded at 298K in D_2O. b) 600 MHz 1D ^{1}H NMR spectra (δ 0.0 to 5.3) of **S23**, recorded at 298K in PBS D2O buffer (40 mM, pH 6.5).

5) Experimental Procedures and Analysis

Compound S1

S1 (0.45 mg, 0.40 μmol) was tested as a substrate using the general procedure for the 6-O-sulfate installation of internal galactose with CHST1. After several days of incubation, no additionally sulfated product was detected by LC-MS, indicating that **S1** it is not a substrate for CHST1.

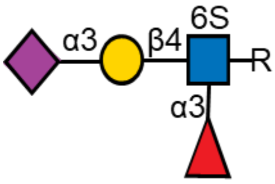

^{1}H (600 MHz, D_2O): δ (ppm)

	H-1	H-2	H-3	H-4	H-5	H-6	H-7	H-8	H-9	NHAc
GlcNAc-6S	4.55 (d, J = 7.5 Hz, 1H)	3.89	n/a	4.02	3.78	4.39 – 4.34 (m, 2H)	-	-	-	2.06 – 1.99 (m, 6H)
Galactose	4.62 (d, J = 7.8 Hz, 1H)	3.52	4.11 (dd, J = 9.6, 2.9 Hz, 1H)	3.96	n/a	3.71 (2H)	-	-	-	-
Fucose	5.10 (d, J = 4.2 Hz, 1H)	3.68	n/a	n/a	4.82	1.18 (d, J = 6.6 Hz, 3H)	-	-	-	-
Sialic acid	-	-	2.76 (dd, J = 12.4, 4.6 Hz, 1H), 1.81 (t, J = 12.1 Hz, 1H)	3.69	3.87	n/a	n/a	n/a	3.90, 3.66	2.06 – 1.99 (m, 6H)

^{13}C (150 MHz, D_2O): δ (ppm)

	C-1	C-2	C-3	C-4	C-5	C-6	C-7	C-8	C-9	NHAc
GlcNAc-6S	100.99	55.79	n/a	72.92	72.57	66.01	-	-	-	22.34
Galactose	102.27	69.54	75.63	67.28	n/a	61.50	-	-	-	-
Fucose	98.58	n/a	n/a	n/a	66.86	15.41	-	-	-	-
Sialic acid	n/a	n/a	39.73	n/a	51.88	n/a	n/a	n/a	62.55	22.34

Linker	1	2	3	4	5	6
H	3.87, 3.57	1.61 – 1.52 (m, 2H)	1.36 – 1.27 (m, 2H)	1.49 (p, J = 7.3 Hz, 2H)	3.12 (t, J = 6.8 Hz, 2H)	5.12 (s, 2H)
C	70.41	28.33	22.42	28.56	40.48	66.88

HRMS (ESI-MS): m/z calculated for $C_{44}H_{67}N_3O_{28}S$ $[M-2H]^{2-}$: 558.6821; found: 558.6805.

Compound S2

S2 (0.65 mg, 0.40 µmol) was tested as a substrate using the general procedure for the 6-O-sulfate installation of internal galactose with CHST1. After several days of incubation, no additional sulfated product was detected by LC-MS indicating that **S2** it is not a substrate for CHST1.

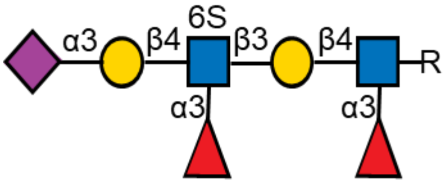

^{1}H (600 MHz, D_2O): δ (ppm)

	H-1	H-2	H-3	H-4	H-5	H-6	H-7	H-8	H-9	NHAc
GlcNAc	4.52 (d, J = 7.3 Hz, 1H)	3.87	n/a	n/a	3.58	3.99, 3.84	-	-	-	2.10 – 1.98 (m, 9H)
Galactose-1	4.44 (d, J = 7.7 Hz, 1H)	3.53	3.71	4.13	n/a	3.71 (4H)	-	-	-	-
GlcNAc-6S	4.74	4.00	3.91	4.02	3.80	4.41 – 4.33 (m, 2H)	-	-	-	2.10 – 1.98 (m, 9H)
Galactose-2	4.61 (d, J = 8.3 Hz, 1H)	3.52	4.11	3.96	n/a	3.71 (4H)	-	-	-	-
Fucose-1	5.09	3.69	n/a	n/a	4.82	1.22 – 1.13 (m, 6H)	-	-	-	-
Fucose-2	5.13	3.69	n/a	n/a	4.82	1.22 – 1.13 (m, 6H)	-	-	-	-
Sialic acid	-	-	2.76 (dd, J = 12.9, 4.7 Hz, 1H), 1.81 (t, J = 12.2 Hz, 1H)	3.69	3.86	n/a	n/a	n/a	3.90, 3.66	2.10 – 1.98 (m, 9H)

^{13}C (150 MHz, D_2O): δ (ppm)

	C-1	C-2	C-3	C-4	C-5	C-6	C-7	C-8	C-9	NHAc
GlcNAc	101.15	55.92	n/a	n/a	75.30	59.94	-	-	-	22.33

Galactose-1	101.91	69.87	82.22	68.31	n/a	61.84	-	-	-	-
GlcNAc-6S	102.67	55.96	n/a	72.80	72.24	66.18	-	-	-	22.33
Galactose-2	101.46	70.60	75.55	67.32	n/a	61.84	-	-	-	-
Fucose-1	98.74	n/a	n/a	n/a	66.98	15.51	-	-	-	-
Fucose-2	98.54	n/a	n/a	n/a	66.98	15.41	-	-	-	-
Sialic acid	n/a	n/a	39.75	n/a	51.78	n/a	n/a	n/a	62.76	22.33

Linker	1	2	3	4	5	6
H	3.88, 3.57	1.61 – 1.52 (m, 2H)	1.36 – 1.27 (m, 2H)	1.53 – 1.46 (m, 2H)	3.19 – 3.04 (m, 2H)	5.12 (s, 2H)
C	70.50	28.42	22.42	28.50	40.48	66.75

HRMS (ESI-MS): m/z calculated for $C_{64}H_{100}N_4O_{42}S$ $[M-2H]^{2-}$: 814.2772; found: 814.2717.

Compound S3

S3 (0.85 mg, 0.40 µmol) was tested as a substrate using the general procedure for the 6-O-sulfate installation of internal galactose with CHST1. After several days of incubation, no additionally sulfated product was detected by LC-MS, indicating that **S3** it is not a substrate for CHST1.

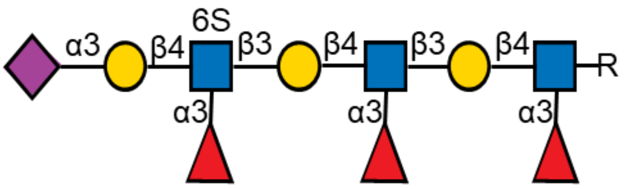

^{1}H (600 MHz, D_2O): δ (ppm)

	H-1	H-2	H-3	H-4	H-5	H-6	H-7	H-8	H-9	NHAc
GlcNAc-1	4.52 (d, J = 8.0 Hz, 1H)	3.87	n/a	n/a	3.58	3.99, 3.84	-	-	-	2.06 – 1.99 (m, 12H)
Galactose-1	4.44 (d, J = 8.0 Hz, 1H)	3.52	3.70	4.11	n/a	3.71 (6H)	-	-	-	-
GlcNAc-2	4.72	3.97	n/a	n/a	3.59	3.97, 3.86	-	-	-	2.06 – 1.99 (m, 12H)
Galactose-2	4.46 (d, J = 8.0 Hz, 1H)	3.52	3.72	4.13 (d, J = 3.7 Hz, 1H)	n/a	3.71 (6H)	-	-	-	-

GlcNAc-6S	4.74	4.00	3.91	4.03	3.80	4.38 – 4.36 (m, 2H)	-	-	-	2.06 – 1.99 (m, 12H)
Galactose-3	4.61 (d, J = 7.8 Hz, 1H)	3.52	4.11	3.96	n/a	3.71 (6H)	-	-	-	-
Fucose-1	5.10 (d, J = 4.0 Hz, 1H)	3.70	n/a	n/a	4.82	1.16 (d, J = 6.6 Hz, 6H)	-	-	-	-
Fucose-2	5.12 (d, J = 5.1 Hz, 1H)	3.70	n/a	n/a	4.82	1.16 (d, J = 6.6 Hz, 6H)	-	-	-	-
Fucose-3	5.14 (d, J = 4.6 Hz, 1H)	3.70	n/a	n/a	4.82	1.18 (d, J = 6.6 Hz, 3H)	-	-	-	-
Sialic acid	-	-	2.76 (dd, J = 12.4, 4.6 Hz, 1H), 1.81 (t, J = 12.2 Hz, 1H)	3.70	3.86	n/a	n/a	n/a	3.90, 3.66	2.06 – 1.99 (m, 12H)

^{13}C (150 MHz, D_2O): δ (ppm)

	C-1	C-2	C-3	C-4	C-5	C-6	H-7	H-8	H-9	NHAc
GlcNAc-1	100.99	56.04	n/a	n/a	75.14	59.88	-	-	-	22.29
Galactose-1	101.71	69.66	81.87	68.34	n/a	61.56	-	-	-	-
GlcNAc-2	102.32	55.91	n/a	n/a	74.99	59.88	-	-	-	22.29
Galactose-2	101.86	69.66	82.19	68.44	n/a	61.56	-	-	-	-
GlcNAc-6S	102.77	55.92	n/a	72.76	72.39	66.09	-	-	-	22.29
Galactose-3	101.21	70.83	75.64	67.34	n/a	61.56	-	-	-	-
Fucose-1	98.72	n/a	n/a	n/a	66.82	15.36	-	-	-	-
Fucose-2	98.67	n/a	n/a	n/a	66.82	15.36	-	-	-	-
Fucose-3	98.41	n/a	n/a	n/a	66.82	15.36	-	-	-	-
Sialic acid	n/a	n/a	39.80	n/a	51.79	n/a	n/a	n/a	62.74	22.29

Linker	1	2	3	4	5	6
H	3.87, 3.57	1.58 – 1.52 (m, 2H)	1.35 – 1.27 (m, 2H)	1.49 (p, J = 7.2 Hz, 2H)	3.12 (t, J = 6.7 Hz, 2H)	5.12 (s, 2H)
C	70.60	28.40	22.45	28.51	40.48	66.87

HRMS (ESI-MS): m/z calculated for $C_{84}H_{133}N_5O_{56}S$ $[M-2H]^{2-}$: 1069.8722; found: 1069.8543.

Compounds S4, S5, and 7

A mixture of compounds **7** and **S5** was prepared from S**4** (1.0 mg, 0.59 µmol) using the general procedure for the 6-O-sulfate installation of internal galactose with CHST1. The NMR data is in agreement with previous reported data.[6]

Compound 2

2 was prepared from **1** (3.0 mg, 2.5 µmol) using the general procedure for the installation of 6-O-sulfate installation of terminal GlcNAc with CHST2. After P6 and HILIC HPLC purification, **2** was obtained as a white solid (2.4 mg, 74%). NMR data is in agreement with previous reported data.[6]

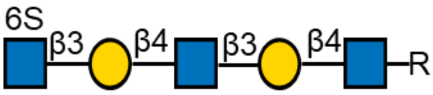

Compound 3

3 was prepared from **2** (2.0 mg, 1.6 µmol) using the general procedure for installation of α1,3 Fuc using FUT6. After P6 purification, **3** was obtained as a white solid (2.4 mg, 96%).

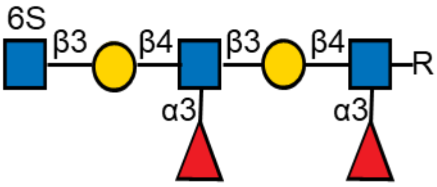

^{1}H (600 MHz, D_2O): δ (ppm)

	H-1	H-2	H-3	H-4	H-5	H-6	NHAc
GlcNAc-1	4.52 (d, J = 7.9 Hz, 1H)	3.87	n/a	n/a	3.59	3.98, 3.85	2.06 – 1.97 (m, 9H)
Galactose-1	4.44 (d, J = 7.9 Hz, 1H)	3.52	3.71	4.11 (d, J = 3.3 Hz, 1H)	n/a	3.72 (4H)	-
GlcNAc-2	4.72	3.98	n/a	n/a	3.59	3.98, 3.85	2.06 – 1.97 (m, 9H)
Galactose-2	4.46 (d, J = 7.9 Hz, 1H)	3.53	3.71	4.13 (d, J = 3.3 Hz, 1H)	n/a	3.72 (4H)	-

GlcNAc-6S	4.70	3.79	3.59	3.53	3.67	4.36 – 4.31 (m, 1H), 4.23 (dd, J = 11.2, 5.5 Hz, 1H)	2.06 – 1.97 (m, 9H)
Fucose-1	5.10 (d, J = 4.0 Hz, 1H)	3.70	n/a	n/a	4.83	1.18 – 1.14 (m, 6H)	-
Fucose-2	5.13 (d, J = 4.1 Hz, 1H)	3.71	n/a	n/a	4.83	1.18 – 1.14 (m, 6H)	-

^{13}C (150 MHz, D_2O): δ (ppm)

	C-1	C-2	C-3	C-4	C-5	C-6	NHAc
GlcNAc-1	100.94	55.82	n/a	n/a	75.02	59.71	22.22
Galactose-1	101.80	70.19	81.82	68.23	n/a	61.55	-
GlcNAc-2	102.67	55.99	n/a	n/a	75.02	59.71	22.22
Galactose-2	101.80	70.19	81.82	68.23	n/a	61.55	-
GlcNAc-6S	102.71	55.60	73.71	69.68	73.64	67.17	22.22
Fucose-1	98.76	n/a	n/a	n/a	66.70	15.25	-
Fucose-2	98.71	n/a	n/a	n/a	66.70	15.25	-

Linker	1	2	3	4	5	6
H	3.88, 3.57	1.59 – 1.52 (m, 2H)	1.35 – 1.26 (m, 2H)	1.49 (p, J = 7.3 Hz, 2H)	3.12 (t, J = 6.8 Hz, 2H)	5.12 (s, 2H)
C	70.44	28.31	22.38	28.51	40.40	66.76

HRMS (ESI-MS): m/z calculated for $C_{61}H_{97}N_4O_{39}S$ $[M-H]^-$: 1541.5456; found: 1541.5505.

Compound 4

4 was prepared from **3** (2.4 mg, 1.5 µmol) using the general procedure for installation of β1,4 Gal using B4GalT4. After P6 purification, **4** was obtained as a white solid (2.6 mg, quant).

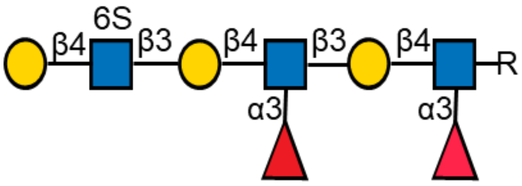

^1H (600 MHz, D_2O): δ (ppm)

	H-1	H-2	H-3	H-4	H-5	H-6	NHAc
GlcNAc-1	4.52	3.87	n/a	n/a	3.59	3.98, 3.85	2.05 – 1.96 (m, 9H)
Galactose-1	4.44 (d, J = 7.9 Hz, 1H)	3.52	3.71	4.11 (d, J = 3.3 Hz, 1H)	n/a	3.72 (4H)	-

GlcNAc-2	4.71	3.97	n/a	n/a	3.59	3.98, 3.85	2.05 – 1.96 (m, 9H)
Galactose-2	4.46 (d, J = 7.9 Hz, 1H)	3.54	3.71	4.13 (d, J = 3.3 Hz, 1H)	n/a	3.72 (4H)	-
GlcNAc-6S	4.73	3.83	3.75	3.80	3.81	4.41 – 4.38 (m, 1H), 4.35 – 4.31 (m, 1H)	2.05 – 1.96 (m, 9H)
Galactose-3	4.54 (d, J = 7.9 Hz, 1H)	3.56	3.69	3.94 (d, J = 3.5 Hz, 1H)	n/a	3.77 (2H)	-
Fucose-1	5.10 (d, J = 4.0 Hz, 1H)	3.70	n/a	n/a	4.82	1.18 – 1.14 (m, 6H)	-
Fucose-2	5.13 (d, J = 4.2 Hz, 1H)	3.70	n/a	n/a	4.82	1.18 – 1.14 (m, 6H)	-

^{13}C (150 MHz, D_2O): δ (ppm)

	C-1	C-2	C-3	C-4	C-5	C-6	NHAc
GlcNAc-1	100.85	55.73	n/a	n/a	74.88	59.71	22.19
Galactose-1	101.69	70.37	81.95	68.21	n/a	61.55	-
GlcNAc-2	102.60	55.93	n/a	n/a	74.88	59.71	22.19
Galactose-2	101.69	70.81	81.95	68.17	n/a	61.55	-
GlcNAc-6S	102.67	55.18	72.23	77.43	72.23	66.35	22.19
Galactose-3	102.34	71.79	72.40	68.71	n/a	61.18	-
Fucose-1	98.65	n/a	n/a	n/a	66.65	15.25	-
Fucose-2	98.55	n/a	n/a	n/a	66.65	15.25	-

Linker	1	2	3	4	5	6
H	3.87, 3.57	1.59 – 1.52 (m, 2H)	1.35 – 1.26 (m, 2H)	1.49 (p, J = 7.3 Hz, 2H)	3.12 (t, J = 6.8 Hz, 2H)	5.12 (s, 2H)
C	70.76	28.26	22.38	28.51	40.40	66.68

HRMS (ESI-MS): m/z calculated for $C_{67}H_{107}N_4O_{44}S$ $[M-H]^-$: 1703.5984; found: 1703.5696.

Compound 5

5 was prepared from **4** (2.6 mg, 1.5 μmol) using the general procedure for installation of α2,3 Neu5Ac using PmST1 M144D. After P6 purification, **5** was obtained as a white solid (2.9 mg, 96%).

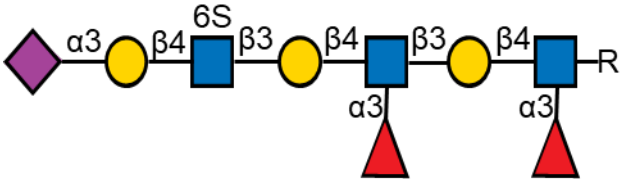

^{1}H (600 MHz, D_2O): δ (ppm)

	H-1	H-2	H-3	H-4	H-5	H-6	H-7	H-8	H-9	NHAc
GlcNAc-1	4.52 (d, J = 7.8 Hz, 1H)	3.88	n/a	n/a	3.58	3.98, 3.86	-	-	-	2.06 – 1.97 (m, 12H)
Galactose-1	4.44 (d, J = 7.9 Hz, 1H)	3.52	3.71	4.11 (d, J = 3.3 Hz, 1H)	n/a	3.73 (4H)	-	-	-	-
GlcNAc-2	4.71 (d, *J* = 8.3 Hz, 1H)	3.98	n/a	n/a	3.58	3.98, 3.86	-	-	-	2.06 – 1.97 (m, 12H)
Galactose-2	4.46 (d, J = 7.9 Hz, 1H)	3.53	3.71	4.14	n/a	3.73 (4H)	-	-	-	-
GlcNAc-6S	4.72 (d, *J* = 8.3 Hz, 1H)	3.83	3.74	3.81	3.81	4.41 (d, J = 11.1 Hz, 1H), 4.33 (d, J = 11.0 Hz, 1H)	-	-	-	2.06 – 1.97 (m, 12H)
Galactose-3	4.61 (d, *J* = 7.9 Hz, 1H)	3.58	4.13	3.98	n/a	3.77 (2H)	-	-	-	-
Sialic acid	-	-	2.76 (dd, J = 12.4, 4.7 Hz, 1H), 1.81 (t, J = 12.1 Hz, 1H)	3.71	3.86	n/a	n/a	n/a	3.90, 3.66	2.06 – 1.97 (m, 12H)
Fucose-1	5.09 (d, J = 3.9 Hz, 1H)	3.70	n/a	n/a	4.82	1.18 – 1.14 (m, 6H)	-	-	-	-
Fucose-2	5.13 (d, J = 4.0 Hz, 1H)	3.70	n/a	n/a	4.82	1.18 – 1.14 (m, 6H)	-	-	-	-

^{13}C (150 MHz, D_2O): δ (ppm)

	C-1	C-2	C-3	C-4	C-5	C-6	C-7	C-8	C-9	NHAc
GlcNAc-1	100.85	55.76	n/a	n/a	74.97	59.71	-	-	-	22.23
Galactose-1	101.81	70.35	82.00	68.21	n/a	61.39	-	-	-	-
GlcNAc-2	102.64	55.97	n/a	n/a	74.97	59.71	-	-	-	22.23
Galactose-2	101.81	70.35	82.00	68.17	n/a	61.39	-	-	-	-
GlcNAc-6S	102.64	55.18	72.26	77.25	72.49	66.43	-	-	-	22.23
Galactose-3	102.13	69.03	75.36	67.51	n/a	61.40	-	-	-	-

Sialic acid	n/a	n/a	39.60	n/a	51.65	n/a	n/a	n/a	62.48	22.23
Fucose-1	98.63	n/a	n/a	n/a	66.65	15.33	-	-	-	-
Fucose-2	98.50	n/a	n/a	n/a	66.65	15.33	-	-	-	-

Linker	1	2	3	4	5	6
H	3.87, 3.57	1.59 – 1.52 (m, 2H)	1.35 – 1.26 (m, 2H)	1.49 (p, J = 7.2 Hz, 2H)	3.12 (t, J = 5.7 Hz, 2H)	5.12 (s, 2H)
C	70.67	28.26	22.38	28.51	40.40	66.68

HRMS (ESI-MS): m/z calculated for $C_{78}H_{123}N_5O_{52}S$ $[M-2H]^{2-}$: 996.8433; found: 996.8371.

Compound 6

Compound **6** was prepared from **5** (2.6 mg, 1.3 μmol) using the general procedure for the 6-O-sulfate installation of internal Galactose using CHST1. After P6 purification, **6** was obtained as a white solid (2.5 mg, 95%).

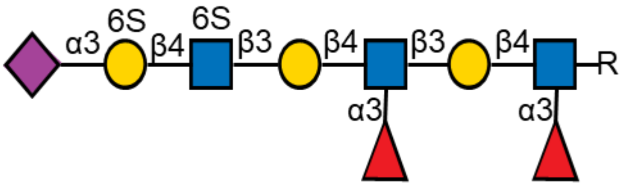

^1H (600 MHz, D_2O): δ (ppm)

	H-1	H-2	H-3	H-4	H-5	H-6	H-7	H-8	H-9	NHAc
GlcNAc-1	4.52 (d, J = 8.0 Hz, 1H)	3.88	n/a	n/a	3.59	3.98, 3.86	-	-	-	2.05 – 1.98 (m, 12H)
Galactose-1	4.44	3.52	3.71	4.11 (d, J = 3.3 Hz, 1H)	n/a	3.73 (4H)	-	-	-	-
GlcNAc-2	4.71 (d, *J* = 8.0 Hz, 2H)	3.98	n/a	n/a	3.59	3.98, 3.86	-	-	-	2.05 – 1.98 (m, 12H)
Galactose-2	4.46 (d, J = 8.0 Hz, 1H)	3.54	3.71	4.14 (d, J = 3.5 Hz, 1H)	n/a	3.73 (4H)	-	-	-	-
GlcNAc-6S	4.71 (d, *J* = 8.0 Hz, 2H)	3.83	n/a	n/a	3.86	4.44, 4.30 (dd, J = 11.1, 5.6 Hz, 1H)	-	-	-	2.05 – 1.98 (m, 12H)
Galactose-6S	4.63 (d, *J* =	3.58	4.15	4.03	3.99	4.19 (2H)	-	-	-	-

	7.9 Hz, 1H)									
Sialic acid	-	-	2.76 (dd, J = 12.5, 4.6 Hz, 1H), 1.83 (t, J = 12.1 Hz, 1H)	3.67	3.87	n/a	n/a	n/a	3.90, 3.66	2.05 – 1.98 (m, 12H)
Fucose-1	5.09 (d, J = 4.0 Hz, 1H)	3.70	n/a	n/a	4.82	1.18 – 1.14 (m, 6H)	-	-	-	-
Fucose-2	5.13 (d, J = 4.1 Hz, 1H)	3.70	n/a	n/a	4.82	1.18 – 1.14 (m, 6H)	-	-	-	-

^{13}C (150 MHz, D_2O): δ (ppm)

	C-1	C-2	C-3	C-4	C-5	C-6	C-7	C-8	C-9	NHAc
GlcNAc-1	100.97	55.79	n/a	n/a	74.97	59.71	-	-	-	22.15
Galactose-1	101.74	70.46	82.00	68.20	n/a	61.61	-	-	-	-
GlcNAc-2	102.59	55.97	n/a	n/a	74.97	59.71	-	-	-	22.15
Galactose-2	101.74	70.46	82.00	68.20	n/a	61.61	-	-	-	-
GlcNAc-6S	102.59	55.18	n/a	n/a	72.61	66.77	-	-	-	22.15
Galactose-6S	102.38	69.38	75.31	67.44	72.93	67.33	-	-	-	-
Sialic acid	n/a	n/a	39.41	n/a	51.65	n/a	n/a	n/a	62.44	22.15
Fucose-1	98.55	n/a	n/a	n/a	66.71	15.33	-	-	-	-
Fucose-2	98.54	n/a	n/a	n/a	66.71	15.33	-	-	-	-

Linker	1	2	3	4	5	6
H	3.87, 3.57	1.59 – 1.52 (m, 2H)	1.35 – 1.26 (m, 2H)	1.49 (p, J = 7.3 Hz, 2H)	3.12 (t, J = 6.7 Hz, 2H)	5.12 (s, 2H)
C	70.42	28.26	22.38	28.51	40.40	66.74

HRMS (ESI-MS): m/z calculated for $C_{78}H_{123}N_5O_{55}S_2$ $[M-2H]^{2-}$: 1036.8217; found: 1036.8112.

Compound 7

7 was prepared from **6** (1.2 mg, 0.6 μmol) using the general procedure for removal of fucose using fucosidase from *R. gnavus* E1. After P6 purification, **7** was obtained as a white solid (1.0 mg, quant). The NMR data are in agreement with previously reported data.[6]

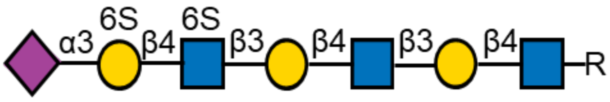

Compound 8

8 was prepared from **S10** (7.0 mg, 6.7 μmol) using the general procedure for installation of β1,3GlcNAc using B3GnT2. After P6 purification, **8** was obtained as a white solid (8.2 mg, 98%).

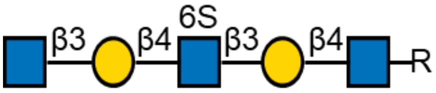

^{1}H (600 MHz, D_2O): δ (ppm)

	H-1	H-2	H-3	H-4	H-5	H-6	NHAc
GlcNAc-1	4.51	3.72	3.69	3.70	3.58	3.99 (d, J = 12.3 Hz, 1H), 3.82	2.07 – 2.00 (m, 9H)
Galactose-1	4.47 (d, J = 7.8 Hz, 1H)	3.60	3.73	4.19 (d, J = 3.2 Hz, 1H)	n/a	3.76 (4H)	-
GlcNAc-6S	4.73 (d, J = 8.3 Hz, 1H)	3.83	3.75	3.80	3.82	4.40 (d, J = 10.9 Hz, 1H), 4.32 (dd, J = 11.2, 4.4 Hz, 1H)	2.07 – 2.00 (m, 9H)
Galactose-2	4.52	3.58	3.73	4.16 (d, J = 3.3 Hz, 1H)	n/a	3.76 (4H)	-
GlcNAc-2	4.70 (d, J = 8.4 Hz, 1H)	3.77	n/a	n/a	3.47	3.90, 3.79	2.07 – 2.00 (m, 9H)

^{13}C (150 MHz, D_2O): δ (ppm)

	C-1	C-2	C-3	C-4	C-5	C-6	NHAc
GlcNAc-1	100.62	55.01	72.27	78.61	74.52	60.13	22.12
Galactose-1	102.86	69.79	82.42	68.32	n/a	60.86	-
GlcNAc-6S	102.83	55.14	72.27	77.91	72.57	66.51	22.12

Galactose-2	102.66	70.62	82.42	68.43	n/a	60.86	-
GlcNAc-2	102.93	55.63	n/a	n/a	n/a	60.41	22.12

Linker	1	2	3	4	5	6
H	3.88(2H)	1.59 – 1.52 (m, 2H)	1.35 – 1.26 (m, 2H)	1.49 (p, J = 7.3 Hz, 2H)	3.12 (t, J = 6.8 Hz, 2H)	5.12 (s, 2H)
C	70.44	28.21	22.33	28.37	40.30	66.76

HRMS (ESI-MS): m/z calculated for $C_{49}H_{77}N_4O_{31}S$ $[M-H]^-$: 1249.4297; found: 1249.4096.

Compound 9

9 was prepared from **8** (8.2 mg, 6.6 μmol) using the general procedure for the 6-O-sulfate installation of terminal GlcNAc using CHST2. After P6 purification, **9** was obtained as a white solid (7.7 mg, 89%).

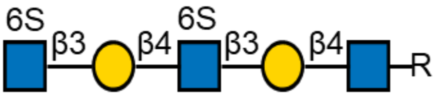

^{1}H (600 MHz, D_2O): δ (ppm)

	H-1	H-2	H-3	H-4	H-5	H-6	NHAc
GlcNAc	4.51	3.72	3.69	3.70	3.58	3.99 (dd, J = 12.3, 2.2 Hz, 1H), 3.82	2.07 – 1.96 (m, 9H)
Galactose-1	4.47 (d, J = 7.9 Hz, 1H)	3.59	3.73	4.20 (d, J = 3.2 Hz, 2H)	n/a	3.76 (4H)	-
GlcNAc-6S-1	4.72 (d, J = 8.4 Hz, 1H)	3.84	3.74	3.78	3.82	4.41 (d, J = 10.8 Hz, 1H), 4.32	2.07 – 1.96 (m, 9H)
Galactose-2	4.52	3.60	3.73	4.20 (d, J = 3.2 Hz, 2H)	n/a	3.76 (4H)	-
GlcNAc-6S-2	4.70 (d, J = 8.5 Hz, 1H)	3.81	3.59	3.53	3.68	4.33 (d, J = 11.5 Hz, 1H), 4.24 (dd, J = 11.2, 5.3 Hz, 1H)	2.07 – 1.96 (m, 9H)

^{13}C (150 MHz, D_2O): δ (ppm)

	C-1	C-2	C-3	C-4	C-5	C-6	NHAc
GlcNAc	100.68	55.05	72.47	78.65	74.36	60.26	22.21
Galactose-1	102.86	69.55	82.75	68.30	n/a	61.20	-
GlcNAc-6S-1	102.92	55.17	72.77	77.99	72.58	66.62	22.21

Galactose-2	102.23	70.48	82.75	68.30	n/a	61.20	-
GlcNAc-6S-2	103.43	55.82	73.31	69.55	73.62	67.06	22.21

Linker	1	2	3	4	5	6
H	3.88(2H)	1.59 – 1.52 (m, 2H)	1.35 – 1.26 (m, 2H)	1.49 (p, J = 7.3 Hz, 2H)	3.12 (t, J = 6.7 Hz, 2H)	5.12 (s, 2H)
C	70.44	28.21	22.33	28.37	40.30	66.76

HRMS (ESI-MS): m/z calculated for $C_{49}H_{76}N_4O_{34}S_2$ $[M-2H]^{2-}$: 664.1896; found: 664.1861.

Compound 10

10 was prepared from **9** (4.0 mg, 3.0 μmol) using the general procedure for installation of α1,3Fuc using FUT6. After P6 purification, **10** was obtained as a white solid (4.5 mg, 92%).

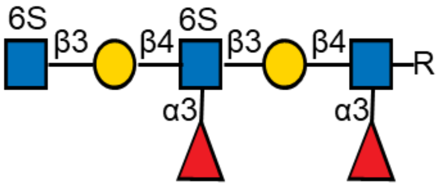

^{1}H (600 MHz, D_2O): δ (ppm)

	H-1	H-2	H-3	H-4	H-5	H-6	NHAc
GlcNAc	4.52	3.88	n/a	n/a	3.59	3.99, 3.85	2.06 – 1.98 (m, 9H)
Galactose-1	4.44 (d, J = 7.8 Hz, 1H)	3.52	3.71	4.12 (d, J = 3.3 Hz, 1H)	n/a	3.73 (4H)	-
GlcNAc-6S-1	4.74 (d, J = 8.6 Hz, 1H)	3.99	3.90	4.01	3.81	4.40 – 4.34 (m, 2H)	2.06 – 1.98 (m, 9H)
Galactose-2	4.51	3.52	3.72	4.14 (d, J = 3.3 Hz, 1H)	n/a	3.73 (4H)	-
GlcNAc-6S-2	4.69 (d, J = 8.4 Hz, 1H)	3.79	3.57	3.55	3.67	4.32 (d, J = 11.2 Hz, 1H), 4.26 (dd, J = 11.1, 4.8 Hz, 1H)	2.06 – 1.98 (m, 9H)
Fucose-1	5.09 (d, J = 4.0 Hz, 1H)	3.70	n/a	n/a	4.83	1.18 – 1.14 (m, 6H)	-
Fucose-2	5.13 (d, J = 4.2 Hz, 1H	3.70	n/a	n/a	4.83	1.18 – 1.14 (m, 6H)	-

^{13}C (150 MHz, D_2O): δ (ppm)

	C-1	C-2	C-3	C-4	C-5	C-6	NHAc
GlcNAc	100.94	55.88	n/a	n/a	74.68	59.88	22.17
Galactose-1	101.75	70.36	82.27	68.19	n/a	61.50	-
GlcNAc-6S-1	102.44	55.88	n/a	72.79	72.28	66.16	22.17
Galactose-2	101.84	70.36	82.27	68.19	n/a	61.50	-
GlcNAc-6S-2	102.82	55.55	73.24	69.25	73.68	66.91	22.17
Fucose-1	98.56	n/a	n/a	n/a	66.70	15.25	-
Fucose-2	98.56	n/a	n/a	n/a	66.70	15.25	-

Linker	1	2	3	4	5	6
H	3.88, 3.57	1.59 – 1.52 (m, 2H)	1.35 – 1.26 (m, 2H)	1.49 (p, J = 7.3 Hz, 2H)	3.12 (t, J = 6.8 Hz, 2H)	5.12 (s, 2H)
C	70.44	28.31	22.38	28.51	40.40	66.76

HRMS (ESI-MS): m/z calculated for $C_{61}H_{96}N_4O_{42}S_2$ $[M-2H]^{2-}$: 810.2476; found: 810.2208.

Compound 11

11 was prepared from **10** (4.5 mg, 2.8 μmol) using the general procedure for installation of β1,4Gal using B4GalT4. After P6 purification, **11** was obtained as a white solid (4.5 mg, 91%).

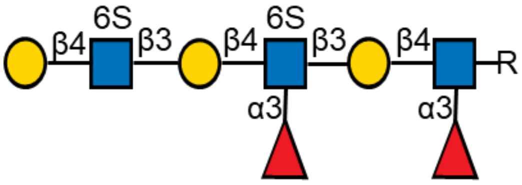

^{1}H (600 MHz, D_2O): δ (ppm)

	H-1	H-2	H-3	H-4	H-5	H-6	NHAc
GlcNAc	4.52	3.88	n/a	n/a	3.59	3.99, 3.85	2.05 – 1.99 (m, 9H)
Galactose-1	4.44 (d, J = 7.8 Hz, 1H)	3.53	3.71	4.12 (d, J = 2.8 Hz, 1H)	n/a	3.73 (4H)	-
GlcNAc-6S-1	4.74 (d, J = 8.9 Hz, 1H)	3.99	3.90	4.00	3.82	4.40 – 4.29 (m, 4H)	2.05 – 1.99 (m, 9H)
Galactose-2	4.52	3.53	3.71	4.14	n/a	3.73 (4H)	-
GlcNAc-6S-2	4.72 (d, J = 8.5 Hz, 1H)	3.84	3.73	3.81	3.82	4.40 – 4.29 (m, 4H)	2.05 – 1.99 (m, 9H)
Galactose-3	4.55 (d, J = 7.5 Hz, 1H)	3.55	3.69	3.94	n/a	3.77 (2H)	-

Fucose-1	5.09 (d, J = 4.0 Hz, 1H)	3.70	n/a	n/a	4.83	1.19 – 1.13 (m, 6H)	-
Fucose-2	5.13 (d, J = 4.0 Hz, 1H	3.70	n/a	n/a	4.83	1.19 – 1.13 (m, 6H)	-

^{13}C (150 MHz, D_2O): δ (ppm)

	C-1	C-2	C-3	C-4	C-5	C-6	NHAc
GlcNAc	100.94	55.88	n/a	n/a	74.87	59.70	22.17
Galactose-1	101.69	70.74	82.22	68.12	n/a	61.50	-
GlcNAc-6S-1	102.44	55.84	n/a	72.83	72.28	66.09	22.17
Galactose-2	101.84	70.74	82.22	68.12	n/a	61.50	-
GlcNAc-6S-2	102.82	55.22	72.15	77.16	72.28	66.09	22.17
Galactose-3	102.61	71.61	72.28	68.90	n/a	61.07	-
Fucose-1	98.56	n/a	n/a	n/a	66.63	15.20	-
Fucose-2	98.56	n/a	n/a	n/a	66.63	15.20	-

Linker	1	2	3	4	5	6
H	3.88, 3.57	1.59 – 1.52 (m, 2H)	1.35 – 1.26 (m, 2H)	1.49 (p, J = 7.3 Hz, 2H)	3.12 (t, J = 6.8 Hz, 2H)	5.12 (s, 2H)
C	70.44	28.31	22.38	28.51	40.40	66.76

HRMS (ESI-MS): m/z calculated for $C_{67}H_{106}N_4O_{47}S_2$ $[M-2H]^{2-}$: 891.2740; found: 891.2546.

Compound 12

12 was prepared from **11** (2.6 mg, 1.5 μmol) using the general procedure for installation of α2,3Neu5Ac using PmST1 M144D. After P6 purification, **12** was obtained as a white solid (2.8 mg, 93%).

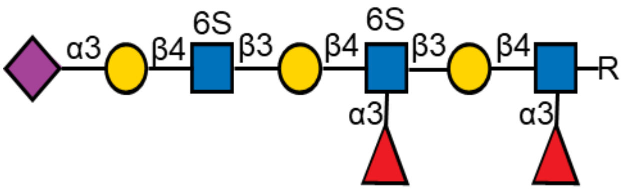

^{1}H (600 MHz, D_2O): δ (ppm)

	H-1	H-2	H-3	H-4	H-5	H-6	H-7	H-8	H-9	NHAc
GlcNAc	4.52	3.88	n/a	n/a	3.58	3.99, 3.85	-	-	-	2.06 – 1.97 (m, 12H)
Galactose-1	4.44 (d, J = 7.8 Hz, 1H)	3.52	3.71	4.13	n/a	3.73 (4H)	-	-	-	-

GlcNAc-6S-1	4.74 (d, *J* = 8.7 Hz, 1H)	3.99	3.90	4.00	3.81	4.40 – 4.30 (m, 4H)	-	-	-	2.06 – 1.97 (m, 12H)
Galactose-2	4.51	3.52	3.71	4.15 (d, J = 3.3 Hz, 1H)	n/a	3.73 (4H)	-	-	-	-
GlcNAc-6S-2	4.71 (d, *J* = 8.4 Hz, 1H)	3.84	3.73	3.81	3.81	4.40 – 4.30 (m, 4H)	-	-	-	2.06 – 1.97 (m, 12H)
Galactose-3	4.62 (d, *J* = 7.8 Hz, 1H)	3.57	4.13	3.98	n/a	3.77 (2H)	-	-	-	-
Sialic acid	-	-	2.76 (dd, J = 12.4, 4.6 Hz, 1H), 1.82 (t, J = 12.1 Hz, 1H)	3.71	3.86	n/a	n/a	n/a	3.90, 3.66	2.06 – 1.97 (m, 12H)
Fucose-1	5.09 (d, J = 4.0 Hz, 1H)	3.70	n/a	n/a	4.82	1.18 – 1.14 (m, 6H)	-	-	-	-
Fucose-2	5.13 (d, J = 4.2 Hz, 1H)	3.70	n/a	n/a	4.82	1.18 – 1.14 (m, 6H)	-	-	-	-

^{13}C (150 MHz, D_2O): δ (ppm)

	C-1	C-2	C-3	C-4	C-5	C-6	C-7	C-8	C-9	NHAc
GlcNAc	100.85	55.93	n/a	n/a	74.97	59.71	-	-	-	22.23
Galactose-1	101.72	70.42	82.17	68.13	n/a	61.52	-	-	-	-
GlcNAc-6S-1	102.54	55.81	n/a	72.95	72.29	66.23	-	-	-	22.23
Galactose-2	101.81	70.42	82.17	68.13	n/a	61.52	-	-	-	-
GlcNAc-6S-2	102.74	55.41	72.51	76.94	72.29	66.23	-	-	-	22.23
Galactose-3	102.13	68.97	75.46	67.59	n/a	61.30	-	-	-	-
Sialic acid	n/a	n/a	39.64	n/a	51.71	n/a	n/a	n/a	62.48	22.23
Fucose-1	98.57	n/a	n/a	n/a	66.65	15.30	-	-	-	-
Fucose-2	98.55	n/a	n/a	n/a	66.65	15.30	-	-	-	-

Linker	1	2	3	4	5	6
H	3.87, 3.57	1.59 – 1.52 (m, 2H)	1.35 – 1.26 (m, 2H)	1.49 (p, J = 7.2 Hz, 2H)	3.12 (t, J = 5.7 Hz, 2H)	5.12 (s, 2H)
C	70.67	28.26	22.38	28.51	40.40	66.68

HRMS (ESI-MS): m/z calculated for $C_{78}H_{123}N_5O_{55}S_2$ $[M-2H]^{2-}$: 1036.8217; found: 1036.7798.

Compound 13

13 was prepared from **12** (2.1 mg, 1.0 μmol) using the general procedure for the 6-O-sulfate installation of internal Galactose using CHST1. After P6 purification, **13** was obtained as a white solid (1.9 mg, 87%).

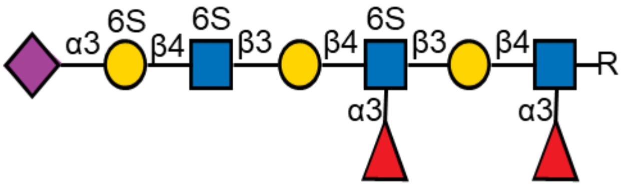

^{1}H (600 MHz, D_2O): δ (ppm)

	H-1	H-2	H-3	H-4	H-5	H-6	H-7	H-8	H-9	NHAc
GlcNAc	4.52	3.88	n/a	n/a	3.59	3.99, 3.85	-	-	-	2.05 – 1.98 (m, 12H)
Galactose-1	4.44 (d, J = 7.8 Hz, 1H)	3.52	3.71	4.13 (d, J = 3.3 Hz, 1H)	n/a	3.73 (4H)	-	-	-	-
GlcNAc-6S-1	4.74 (d, *J* = 8.7 Hz, 1H)	3.99	3.90	4.00	3.82	4.43 – 4.30 (m, 4H)	-	-	-	2.05 – 1.98 (m, 12H)
Galactose-2	4.51	3.52	3.71	4.15	n/a	3.73 (4H)	-	-	-	-
GlcNAc-6S-2	4.71 (d, *J* = 8.4 Hz, 1H)	3.84	n/a	n/a	3.82	4.43 – 4.30 (m, 4H)	-	-	-	2.05 – 1.98 (m, 12H)
Galactose-6S	4.63 (d, *J* = 7.8 Hz, 1H)	3.57	4.16	4.03 (d, J = 3.2 Hz, 1H)	3.99	4.18 (2H)	-	-	-	-
Sialic acid	-	-	2.76 (dd, J = 12.5, 4.6 Hz, 1H), 1.84 (t, J = 12.1 Hz, 1H)	3.67	3.87	n/a	n/a	n/a	3.90, 3.66	2.05 – 1.98 (m, 12H)
Fucose-1	5.09 (d, J = 4.0 Hz, 1H)	3.70	n/a	n/a	4.82	1.18 – 1.14 (m, 6H)	-	-	-	-
Fucose-2	5.13 (d, J = 4.2 Hz, 1H)	3.70	n/a	n/a	4.82	1.18 – 1.14 (m, 6H)	-	-	-	-

^{13}C (150 MHz, D_2O): δ (ppm)

	C-1	C-2	C-3	C-4	C-5	C-6	C-7	C-8	C-9	NHAc
GlcNAc	100.97	55.79	n/a	n/a	74.97	59.71	-	-	-	22.20
Galactose-1	101.74	70.46	82.35	68.30	n/a	61.61	-	-	-	-
GlcNAc-6S-1	102.41	55.97	n/a	72.70	72.59	66.44	-	-	-	22.20
Galactose-2	101.74	70.46	82.35	68.20	n/a	61.61	-	-	-	-
GlcNAc-6S-2	102.72	55.18	n/a	n/a	72.59	66.44	-	-	-	22.20
Galactose-6S	102.63	68.74	75.03	67.44	72.93	67.08	-	-	-	-
Sialic acid	n/a	n/a	39.41	n/a	51.60	n/a	n/a	n/a	62.62	22.20
Fucose-1	98.61	n/a	n/a	n/a	66.82	15.33	-	-	-	-
Fucose-2	98.50	n/a	n/a	n/a	66.82	15.33	-	-	-	-

Linker	1	2	3	4	5	6
H	3.87, 3.57	1.59 – 1.52 (m, 2H)	1.35 – 1.26 (m, 2H)	1.49 (p, J = 7.3 Hz, 2H)	3.12 (t, J = 6.7 Hz, 2H)	5.12 (s, 2H)
C	70.42	28.26	22.38	28.51	40.40	66.74

HRMS (ESI-MS): m/z calculated for $C_{78}H_{122}N_5O_{58}S_3$ $[M-3H]^{3-}$: 717.5310; found: 717.5153.

Compound 14

14 was prepared from **13** (1.4 mg, 0.7 μmol) using the general procedure for removal of fucose using fucosidase from *R. gnavus* E1. After P6 purification, **14** was obtained as a white solid (1.2 mg, quant).

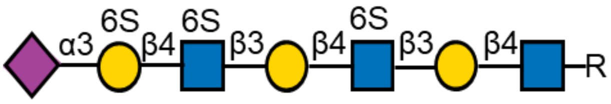

^1H (600 MHz, D_2O): δ (ppm)

	H-1	H-2	H-3	H-4	H-5	H-6	H-7	H-8	H-9	NHAc
GlcNAc	4.51	3.71	3.70	3.70	3.58	3.99, 3.83	-	-	-	2.09 – 1.98 (m, 12H)
Galactose-1	4.47 (d, J = 7.9 Hz, 1H)	3.60	3.73	4.20	n/a	3.77 (4H)	-	-	-	-
GlcNAc-6S-1	4.72	3.84	n/a	n/a	3.83	4.44 – 4.39 (m, 2H), 4.35 – 4.28	-	-	-	2.09 – 1.98 (m, 12H)

						(m, 2H)				
Galactose-2	4.52	3.60	3.73	4.21	n/a	3.77 (4H)	-	-	-	-
GlcNAc-6S-2	4.71	3.86	n/a	n/a	3.83	4.44 – 4.39 (m, 2H), 4.35 – 4.28 (m, 2H)	-	-	-	2.09 – 1.98 (m, 12H)
Galactose-6S	4.63 (d, J = 7.9 Hz, 1H)	3.59	4.16	4.03	3.99	4.18 (2H)	-	-	-	-
Sialic acid	-	-	2.75 (dd, J = 12.7, 4.4 Hz, 1H), 1.82 (t, J = 12.1 Hz, 1H)	3.67	3.87	n/a	n/a	n/a	3.90, 3.66	2.09 – 1.98 (m, 12H)

^{13}C (150 MHz, D_2O): δ (ppm)

	C-1	C-2	C-3	C-4	C-5	C-6	C-7	C-8	C-9	NHAc
GlcNAc	101.21	55.09	72.64	78.69	74.73	60.11	-	-	-	22.21
Galactose-1	102.82	69.31	82.44	68.49	n/a	61.16	-	-	-	-
GlcNAc-6S-1	102.91	55.02	n/a	n/a	72.41	66.68	-	-	-	22.21
Galactose-2	102.72	69.31	82.44	68.49	n/a	61.16	-	-	-	-
GlcNAc-6S-2	102.87	55.02	n/a	n/a	72.41	66.68	-	-	-	22.21
Galactose-6S	102.52	69.31	75.02	67.48	72.68	66.82	-	-	-	-
Sialic acid	n/a	n/a	39.60	n/a	51.73	n/a	n/a	n/a	62.50	22.21

Linker	1	2	3	4	5	6
H	3.88,3.57	1.59 – 1.52 (m, 2H)	1.35 – 1.27 (m, 2H)	1.49 (p, J = 7.3 Hz, 2H)	3.12 (t, J = 6.7 Hz, 2H)	5.12 (s, 2H)
C	70.25	28.34	22.56	28.63	40.47	66.89

HRMS (ESI-MS): m/z calculated for $C_{66}H_{102}N_5O_{50}S_3$ $[M-3H]^{3-}$: 620.1590; found: 620.1464.

Compound S6

S6 was prepared from **2** (6.0 mg, 4.8 μmol) using the general procedure for installation of α2,6Neu5Ac using PT2,6ST. After P6 purification, **S6** was obtained as a white solid (6.4 mg, 73%).

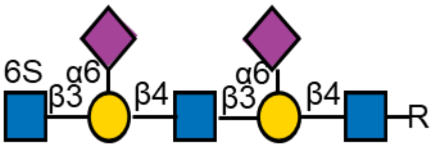

^{1}H (600 MHz, D_2O): δ (ppm)

	H-1	H-2	H-3	H-4	H-5	H-6	H-7	H-8	H-9	NHAc
GlcNAc-1	4.54 (d, J = 7.2 Hz, 1H)	3.72	n/a	n/a	3.58	3.99, 3.82	-	-	-	2.09 – 2.00 (m, 15H)
Galactose-1	4.44 (d, J = 8.0 Hz, 2H)	3.61	3.73	4.17	n/a	4.00, 3.55	-	-	-	-
GlcNAc-2	4.72	3.82	n/a	n/a	n/a	3.97, 3.85	-	-	-	2.09 – 2.00 (m, 15H)
Galactose-2	4.44 (d, J = 8.0 Hz, 2H)	3.61	3.73	4.18	n/a	4.00, 3.55	-	-	-	-
GlcNAc-6S	4.70	3.79	3.58	3.54	3.66	4.32 (d, J = 11.0 Hz, 1H), 4.28 – 4.23 (m, 1H)	-	-	-	2.09 – 2.00 (m, 15H)
Sialic acid-1	-	-	2.67 (dd, J = 11.1, 6.0 Hz, 2H), 1.78 – 1.67 (m, 2H)	3.68	3.81	n/a	3.57	3.91	3.89, 3.65	2.09 – 2.00 (m, 15H)
Sialic acid-2	-	-	2.67 (dd, J = 11.1, 6.0 Hz, 2H), 1.78 – 1.67 (m, 2H)	3.68	3.81	n/a	3.57	3.91	3.89, 3.65	2.09 – 2.00 (m, 15H)

^{13}C (150 MHz, D_2O): δ (ppm)

	C-1	C-2	C-3	C-4	C-5	C-6	C-7	C-8	C-9	NHAc

GlcNAc-1	100.90	54.89	n/a	n/a	74.06	60.41	-	-	-	22.11
Galactose-1	103.56	69.66	82.32	68.15	n/a	63.44	-	-	-	-
GlcNAc-2	102.68	54.84	n/a	n/a	n/a	60.41	-	-	-	22.11
Galactose-2	103.56	69.66	82.32	68.15	n/a	63.44	-	-	-	-
GlcNAc-6S	102.88	55.50	73.91	68.72	73.64	66.92	-	-	-	22.11
Sialic acid-1	n/a	n/a	40.12	n/a	51.89	n/a	68.81	71.64	62.74	22.11
Sialic acid-2	n/a	n/a	40.12	n/a	51.89	n/a	68.81	71.64	62.74	22.11

Linker	1	2	3	4	5	6
H	3.88,3.58	1.61 – 1.52 (m, 2H)	1.37 – 1.24 (m, 2H)	1.50 (p, J = 7.2 Hz, 2H)	3.13 (t, J = 6.5 Hz, 2H)	5.12 (s, 2H)
C	70.74	28.25	22.48	28.44	40.37	66.73

HRMS (ESI-MS): m/z calculated for $C_{71}H_{110}N_6O_{47}S$ $[M-2H]^{2-}$: 915.3067; found: 915.2843.

Compound S7

S7 was prepared from S**6** (6.4 mg, 3.5 µmol) using the general procedure for installation of β1,4Gal using Hpβ4GalT. After P6 purification, **S7** was obtained as a white solid (6.6 mg, 94%).

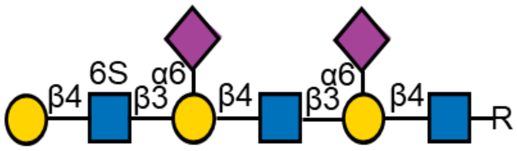

^{1}H (600 MHz, D_2O): δ (ppm)

	H-1	H-2	H-3	H-4	H-5	H-6	H-7	H-8	H-9	NHAc
GlcNAc-1	4.54	3.73	n/a	n/a	n/a	3.99, 3.82	-	-	-	2.08 – 2.01 (m, 15H)
Galactose-1	4.44 (d, J = 8.0 Hz, 2H)	3.61	3.74	4.18	n/a	4.00, 3.55	-	-	-	-
GlcNAc-2	4.72	3.81	n/a	n/a	n/a	3.97, 3.85	-	-	-	2.08 – 2.01 (m, 15H)
Galactose-2	4.44 (d, J = 8.0 Hz, 2H)	3.61	3.74	4.18	n/a	4.00, 3.55	-	-	-	-

GlcNAc-6S	4.73	3.85	n/a	n/a	3.81	4.39 – 4.32 (m, 2H)	-	-	-	2.08 – 2.01 (m, 15H)
Galactose-3	4.55	3.54	3.72	3.94	n/a	3.76 (2H)	-	-	-	-
Sialic acid-1	-	-	2.69 – 2.61 (m, 2H), 1.77 – 1.67 (m, 2H)	3.69	3.81	n/a	3.57	3.90	3.88, 3.65	2.08 – 2.01 (m, 15H)
Sialic acid-2	-	-	2.69 – 2.61 (m, 2H), 1.77 – 1.67 (m, 2H)	3.69	3.81	n/a	3.57	3.90	3.88, 3.65	2.08 – 2.01 (m, 15H)

^{13}C (150 MHz, D_2O): δ (ppm)

	C-1	C-2	C-3	C-4	C-5	C-6	C-7	C-8	C-9	NHAc
GlcNAc-1	100.90	54.89	n/a	n/a	n/a	60.34	-	-	-	22.20
Galactose-1	103.56	69.59	82.38	68.06	n/a	63.41	-	-	-	-
GlcNAc-2	102.68	54.91	n/a	n/a	n/a	60.34	-	-	-	22.20
Galactose-2	103.56	69.59	82.38	68.06	n/a	63.41	-	-	-	-
GlcNAc-6S	102.88	55.13	n/a	n/a	72.47	66.23	-	-	-	22.20
Galactose-3	102.45	71.27	72.53	68.68	n/a	60.99	-	-	-	-
Sialic acid-1	n/a	n/a	40.12	n/a	51.89	n/a	68.68	71.72	62.77	22.20
Sialic acid-2	n/a	n/a	40.12	n/a	51.89	n/a	68.68	71.72	62.77	22.20

Linker	1	2	3	4	5	6
H	3.88,3.58	1.61 – 1.52 (m, 2H)	1.37 – 1.24 (m, 2H)	1.49 (p, J = 7.5 Hz, 2H)	3.13 (t, J = 6.2 Hz, 2H)	5.12 (s, 2H)
C	70.22	28.25	22.38	28.44	40.37	66.73

HRMS (ESI-MS): m/z calculated for $C_{77}H_{120}N_6O_{52}S$ $[M-2H]^{2-}$: 996.3331; found: 996.3305.

Compound S8

S8 was prepared from **S7** (6.6 mg, 3.3 μmol) using the general procedure for installation of α2,3Neu5Ac using PmST1 M144D. After P6 purification, **S8** was obtained as a white solid (6.9 mg, 92%).

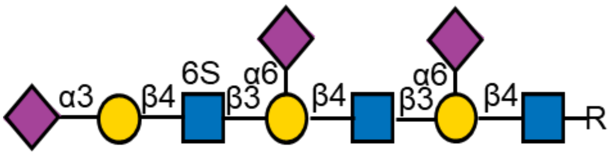

^{1}H (600 MHz, D_2O): δ (ppm)

	H-1	H-2	H-3	H-4	H-5	H-6	H-7	H-8	H-9	NHAc
GlcNAc-1	4.54 (d, J = 6.9 Hz, 1H)	3.73	n/a	n/a	3.59	3.99, 3.82	-	-	-	2.08 – 1.99 (m, 18H)
Galactose-1	4.44 (d, J = 7.9 Hz, 2H)	3.61	3.74	4.18	n/a	4.01, 3.56	-	-	-	-
GlcNAc-2	4.72 (d, J = 7.6 Hz, 2H)	3.81	n/a	n/a	n/a	3.97, 3.85	-	-	-	2.08 – 1.99 (m, 18H)
Galactose-2	4.44 (d, J = 7.9 Hz, 2H)	3.61	3.74	4.18	n/a	4.01, 3.56	-	-	-	-
GlcNAc-6S	4.72 (d, J = 7.6 Hz, 2H)	3.84	n/a	n/a	3.81	4.39 – 4.32 (m, 2H)	-	-	-	2.08 – 1.99 (m, 18H)
Galactose-3	4.62 (d, J = 7.7 Hz, 1H)	3.57	4.14 (dd, J = 9.9, 3.1 Hz, 1H)	3.98	n/a	3.74 (2H)	-	-	-	-
Sialic acid-1	-	-	2.70 – 2.63 (m, 2H), 1.78 – 1.66 (m, 2H)	3.68	3.82	n/a	n/a	3.91	3.89, 3.66	2.08 – 1.99 (m, 18H)
Sialic acid-2	-	-	2.70 – 2.63 (m, 2H), 1.78 – 1.66 (m, 2H)	3.68	3.82	n/a	n/a	3.91	3.89, 3.66	2.08 – 1.99 (m, 18H)
Sialic acid-3	-	-	2.76 (dd, J =	3.70	3.87	n/a	n/a	3.91	3.89, 3.66	2.08 – 1.99

			12.4, 4.6 Hz, 1H), 1.82 (t, J = 12.2 Hz, 1H)							(m, 18H)

^{13}C (150 MHz, D_2O): δ (ppm)

	C-1	C-2	C-3	C-4	C-5	C-6	C-7	C-8	C-9	NHAc
GlcNAc-1	100.90	54.89	n/a	n/a	74.59	60.30	-	-	-	22.20
Galactose-1	103.56	69.86	82.38	68.06	n/a	63.52	-	-	-	-
GlcNAc-2	102.86	54.87	n/a	n/a	n/a	60.19	-	-	-	22.20
Galactose-2	103.56	69.86	82.38	68.06	n/a	63.52	-	-	-	-
GlcNAc-6S	102.86	55.29	n/a	n/a	72.84	66.37	-	-	-	22.20
Galactose-3	102.14	69.49	75.37	67.51	n/a	61.11	-	-	-	-
Sialic acid-1	n/a	n/a	40.04	n/a	51.86	n/a	n/a	71.63	62.78	22.20
Sialic acid-2	n/a	n/a	40.04	n/a	51.86	n/a	n/a	71.63	62.78	22.20
Sialic acid-3	n/a	n/a	39.60	n/a	51.71	n/a	n/a	71.63	62.78	22.20

Linker	1	2	3	4	5	6
H	3.88,3.58	1.61 – 1.52 (m, 2H)	1.37 – 1.24 (m, 2H)	1.50 (p, J = 7.2 Hz, 2H)	3.13 (t, J = 6.8 Hz, 2H)	5.12 (s, 2H)
C	70.22	28.28	22.44	28.49	40.43	66.77

HRMS (ESI-MS): m/z calculated for $C_{88}H_{137}N_7O_{60}S$ $[M-2H]^{2-}$: 1141.8808; found: 1141.9083.

Compound S9

S9 was prepared from **S8** (2.3 mg, 1.0 μmol) using the general procedure for the 6-O-sulfate installation of internal Galactose using CHST1. After P6 purification, **S9** was obtained as a white solid (1.9 mg, 82%).

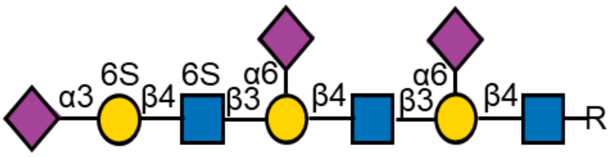

^{1}H (600 MHz, D_2O): δ (ppm)

	H-1	H-2	H-3	H-4	H-5	H-6	H-7	H-8	H-9	NHAc
GlcNAc-1	4.54	3.73	n/a	n/a	3.59	3.99，3.82	-	-	-	2.08 – 1.98 (m, 18H)
Galactose-1	4.44	3.61	3.74	4.18	n/a	4.00, 3.56	-	-	-	-
GlcNAc-2	4.72	n/a	n/a	n/a	n/a	3.97，3.85	-	-	-	2.08 – 1.98 (m, 18H)
Galactose-2	4.44	3.61	3.74	4.18	n/a	4.00, 3.56	-	-	-	-
GlcNAc-6S	4.72	n/a	n/a	n/a	3.82	4.39 – 4.32 (m, 2H)	-	-	-	2.08 – 1.98 (m, 18H)
Galactose-6S	4.63	3.59	n/a	n/a	3.99	4.19 (2H)	-	-	-	-
Sialic acid-1	-	-	2.70 – 2.63 (m, 2H), 1.78 – 1.66 (m, 2H)	3.68	3.82	n/a	n/a	3.90	3.88, 3.65	2.08 – 1.98 (m, 18H)
Sialic acid-2	-	-	2.70 – 2.63 (m, 2H), 1.78 – 1.66 (m, 2H)	3.68	3.82	n/a	n/a	3.90	3.88, 3.65	2.08 – 1.98 (m, 18H)
Sialic acid-3	-	-	2.80 – 2.71 (m, 1H)，1.87 – 1.80 (m, 1H)	3.70	3.86	n/a	n/a	3.90	3.88, 3.65	2.08 – 1.98 (m, 18H)

^{13}C (150 MHz, D_2O): δ (ppm)

	C-1	C-2	C-3	C-4	C-5	C-6	C-7	C-8	C-9	NHAc
GlcNAc-1	100.90	55.14	n/a	n/a	74.66	60.43	-	-	-	22.14
Galactose-1	103.56	69.42	82.25	68.25	n/a	63.39	-	-	-	-
GlcNAc-2	102.86	n/a	n/a	n/a	n/a	60.43	-	-	-	22.14
Galactose-2	103.56	69.42	82.25	68.25	n/a	63.39	-	-	-	-
GlcNAc-6S	102.86	n/a	n/a	n/a	72.90	n/a	-	-	-	22.14

Galactose-6S	102.89	69.58	n/a	n/a	72.50	67.03	-	-	-	-
Sialic acid-1	n/a	n/a	40.12	n/a	51.97	n/a	n/a	71.82	62.78	22.14
Sialic acid-2	n/a	n/a	40.12	n/a	51.97	n/a	n/a	71.82	62.78	22.14
Sialic acid-3	n/a	n/a	n/a	n/a	51.88	n/a	n/a	71.82	62.78	22.14

Linker	1	2	3	4	5	6
H	3.88,3.58	1.61 – 1.52 (m, 2H)	1.37 – 1.24 (m, 2H)	1.52 – 1.46 (m, 1H)	3.19 – 3.07 (m, 1H)	5.12 (s, 2H)
C	70.22	28.28	22.44	28.49	40.43	66.77

HRMS (ESI-MS): m/z calculated for $C_{88}H_{137}N_7O_{63}S_2$ $[M-2H]^{2-}$: 1181.8592; found: 1181.8729.

Compound S14

S14 was prepared from **S9** (1.9 mg, 0.8 μmol) using the general procedure for the removal of sialic acid using *C. perfringens* neuraminidase. After P6 purification, **S14** was obtained as a white solid (1.2 mg, quant).

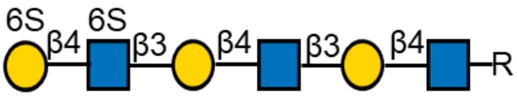

^1H (600 MHz, D_2O): δ (ppm)

	H-1	H-2	H-3	H-4	H-5	H-6	NHAc
GlcNAc-1	4.51 (d, J = 7.5 Hz, 1H)	3.70	3.70	3.69	3.58	3.99，3.83	2.06 – 1.99 (m, 9H)
Galactose-1	4.46	3.60	3.72	4.16 (d, J = 3.3 Hz, 1H)	n/a	3.76 (4H)	-
GlcNAc-2	4.71	3.82	3.74	3.74	3.59	3.97, 3.85	2.06 – 1.99 (m, 9H)
Galactose-2	4.48	3.60	3.73	4.20	n/a	3.76 (4H)	-
GlcNAc-6S	4.72	3.83	n/a	n/a	3.84	4.42 (d, J = 9.2 Hz, 1H), 4.31 (dd, J = 11.1, 5.3 Hz, 1H)	2.06 – 1.99 (m, 9H)
Galactose-6S	4.55 (d, J = 7.8 Hz, 1H)	3.56	3.75	4.00	3.99	4.21 (2H)	-

^{13}C (150 MHz, D_2O): δ (ppm)

	C-1	C-2	C-3	C-4	C-5	C-6	NHAc
GlcNAc-1	101.12	55.06	72.57	78.56	74.87	59.95	22.19
Galactose-1	102.73	69.99	82.07	68.29	n/a	61.01	-
GlcNAc-2	102.88	55.06	72.29	78.49	74.87	59.95	22.19
Galactose-2	103.13	69.99	82.25	68.73	n/a	61.01	-
GlcNAc-6S	103.06	55.06	n/a	n/a	72.36	67.01	22.19
Galactose-6S	102.73	71.20	n/a	68.37	72.84	66.77	-

Linker	1	2	3	4	5	6
H	3.88, 3.55	1.59 – 1.52 (m, 2H)	1.35 – 1.26 (m, 2H)	1.49 (p, J = 7.2 Hz, 2H)	3.12 (t, J = 6.8 Hz, 1H)	5.12 (s, 2H)
C	70.33	28.24	22.42	28.53	40.53	66.56

HRMS (ESI-MS): m/z calculated for $C_{55}H_{86}N_4O_{39}S_2$ $[M-2H]^{2-}$: 745.2161; found: 745.2246.

Compound 7

7 was prepared from **S14** (1.2 mg, 0.8 µmol) using the general procedure for installation of α2,3 Neu5Ac using PmST1 M144D. After P6 purification, **7** was obtained as a white solid (1.2 mg, 83%). NMR data is in agreement with previously reported data.[6]

Compound 15

15 was prepared from **9** (6.0 mg, 4.5 µmol) using the general procedure for installation of α2,6Neu5Ac using PT2,6ST. After P6 purification, **15** was obtained as a white solid (6.1 mg, 71%).

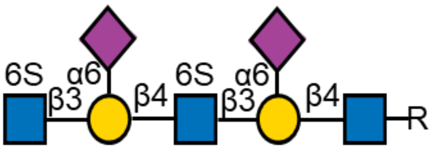

^{1}H (600 MHz, D_2O): δ (ppm)

	H-1	H-2	H-3	H-4	H-5	H-6	H-7	H-8	H-9	NHAc
GlcNAc	4.54 (d, J = 7.8 Hz, 1H)	3.72	n/a	n/a	3.60	3.99, 3.82	-	-	-	2.09 – 2.00 (m, 15H)
Galactose-1	4.44 (d, J = 8.0 Hz, 1H)	3.61	3.74	4.18	n/a	4.01, 3.55	-	-	-	-

GlcNAc-6S-1	4.75 (d, J = 7.2 Hz, 1H)	3.82	n/a	n/a	3.85	4.40 (d, J = 10.8 Hz, 1H), 4.30	-	-	-	2.09 – 2.00 (m, 15H)
Galactose-2	4.48 (d, J = 7.9 Hz, 1H)	3.60	3.74	4.17	n/a	4.01, 3.55	-	-	-	-
GlcNAc-6S-2	4.71 (d, J = 8.5 Hz, 1H)	3.79	3.58	3.54	3.66	4.30 – 4.24 (m, 2H)	-	-	-	2.09 – 2.00 (m, 15H)
Sialic acid-1	-	-	2.66 (dd, J = 12.4, 4.6 Hz, 2H), 1.74 (td, J = 12.2, 3.5 Hz, 2H)	3.68	3.81	n/a	n/a	3.91	3.88, 3.65	2.09 – 2.00 (m, 15H)
Sialic acid-2	-	-	2.66 (dd, J = 12.4, 4.6 Hz, 2H), 1.74 (td, J = 12.2, 3.5 Hz, 2H)	3.68	3.81	n/a	n/a	3.91	3.88, 3.65	2.09 – 2.00 (m, 15H)

^{13}C (150 MHz, D_2O): δ (ppm)

	C-1	C-2	C-3	C-4	C-5	C-6	C-7	C-8	C-9	NHAc
GlcNAc	100.90	54.89	n/a	n/a	74.59	60.65	-	-	-	22.11
Galactose-1	103.56	69.67	82.23	68.15	n/a	63.59	-	-	-	-
GlcNAc-6S-1	102.48	55.05	n/a	n/a	73.80	66.90	-	-	-	22.11
Galactose-2	103.56	69.39	82.23	68.15	n/a	63.59	-	-	-	-
GlcNAc-6S-2	103.09	55.53	73.80	68.72	73.40	66.90	-	-	-	22.11
Sialic acid-1	n/a	n/a	40.12	n/a	51.89	n/a	n/a	71.64	62.84	22.11
Sialic acid-2	n/a	n/a	40.12	n/a	51.89	n/a	n/a	71.64	62.84	22.11

Linker	1	2	3	4	5	6
H	3.88,3.58	1.61 – 1.52 (m, 2H)	1.37 – 1.24 (m, 2H)	1.50 (p, J = 7.2 Hz, 2H)	3.13 (t, J = 6.5 Hz, 2H)	5.12 (s, 2H)
C	70.74	28.25	22.48	28.44	40.37	66.73

HRMS (ESI-MS): m/z calculated for $C_{71}H_{110}N_6O_{50}S_2$ $[M-2H]^{2-}$: 955.2851; found: 955.2877.

Compound 16

16 was prepared from **15** (6.1 mg, 3.2 µmol) using the general procedure for installation of β1,4Gal using Hpβ4GalT. After P6 purification, **16** was obtained as a white solid (6.4 mg, 97%).

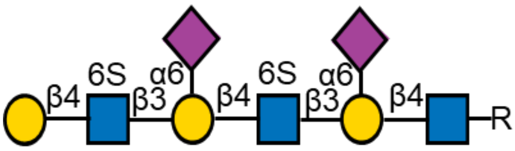

^{1}H (600 MHz, D_2O): δ (ppm)

	H-1	H-2	H-3	H-4	H-5	H-6	H-7	H-8	H-9	NHAc
GlcNAc	4.54	3.73	n/a	n/a	3.61	3.99, 3.82	-	-	-	2.10 – 1.99 (m, 15H)
Galactose-1	4.44 (d, J = 7.9 Hz, 1H)	3.61	3.74	4.17 (d, J = 3.7 Hz, 1H)	n/a	4.01, 3.54	-	-	-	-
GlcNAc-6S-1	4.75	3.82	n/a	n/a	3.84	4.40 (d, J = 10.6 Hz, 1H), 4.31 (dd, J = 11.1, 5.0 Hz, 1H)	-	-	-	2.10 – 1.99 (m, 15H)
Galactose-2	4.48 (d, J = 7.9 Hz, 1H)	3.60	3.74	4.16	n/a	4.01, 3.54	-	-	-	-
GlcNAc-6S-2	4.73	3.85	n/a	n/a	3.81	4.36 (2H)	-	-	-	2.10 – 1.99 (m, 15H)
Galactose-3	4.55	3.54	3.69	3.93 (d, J = 3.5 Hz, 1H)	n/a	3.76 (2H)	-	-	-	-
Sialic acid-1	-	-	2.67 (dd, J = 12.5, 4.6 Hz, 2H), 1.73 (dd, J = 13.4, 10.9 Hz, 2H)	3.68	3.81	n/a	3.57	3.91	3.88, 3.65	2.10 – 1.99 (m, 15H)
Sialic acid-2	-	-	2.67 (dd, J = 12.5, 4.6 Hz, 2H),	3.68	3.81	n/a	3.57	3.91	3.88, 3.65	2.10 – 1.99 (m, 15H)

			1.73 (dd, J = 13.4, 10.9 Hz, 2H)							

^{13}C (150 MHz, D_2O): δ (ppm)

	C-1	C-2	C-3	C-4	C-5	C-6	C-7	C-8	C-9	NHAc
GlcNAc	100.74	54.84	n/a	n/a	74.57	60.57	-	-	-	22.20
Galactose-1	103.35	69.82	82.54	68.13	n/a	63.41	-	-	-	-
GlcNAc-6S-1	102.44	54.95	n/a	n/a	73.77	66.63	-	-	-	22.20
Galactose-2	103.54	69.53	82.54	68.13	n/a	63.41	-	-	-	-
GlcNAc-6S-2	103.15	55.00	n/a	n/a	72.71	66.19	-	-	-	22.20
Galactose-3	102.54	71.65	72.53	68.68	n/a	61.15	-	-	-	-
Sialic acid-1	n/a	n/a	40.12	n/a	51.89	n/a	68.68	71.72	62.64	22.20
Sialic acid-2	n/a	n/a	40.12	n/a	51.89	n/a	68.68	71.72	62.64	22.20

Linker	1	2	3	4	5	6
H	3.88,3.58	1.61 – 1.52 (m, 2H)	1.37 – 1.24 (m, 2H)	1.49 (p, J = 7.5 Hz, 2H)	3.13 (t, J = 6.2 Hz, 2H)	5.12 (s, 2H)
C	70.22	28.25	22.38	28.44	40.37	66.73

HRMS (ESI-MS): m/z calculated for $C_{77}H_{120}N_6O_{55}S_2$ $[M-2H]^{2-}$: 1036.3115; found: 1036.2769.

Compound 17

17 was prepared from **16** (6.4 mg, 3.1 µmol) using the general procedure for installation of α2,3Neu5Ac using PmST1 M144D. After P6 purification, **17** was obtained as a white solid (6.8 mg, 93%).

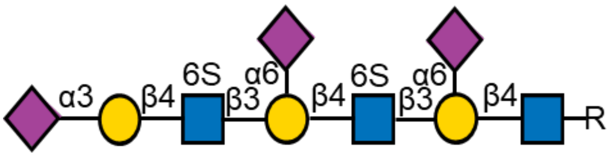

^{1}H (600 MHz, D_2O): δ (ppm)

	H-1	H-2	H-3	H-4	H-5	H-6	H-7	H-8	H-9	NHAc
GlcNAc	4.55 (d, J = 7.9 Hz, 1H)	3.73	n/a	n/a	3.59	3.99, 3.82	-	-	-	2.08 – 1.99 (m, 18H)
Galactose-1	4.44 (d, J = 7.8 Hz, 1H)	3.61	3.75	4.17	n/a	4.01, 3.54	-	-	-	-
GlcNAc-6S-1	4.76 (d, J = 8.0 Hz, 1H)	3.83	n/a	n/a	3.84	4.40 (d, J = 10.6 Hz, 1H), 4.30 (dd, J = 11.4, 5.0 Hz, 1H)	-	-	-	2.08 – 1.99 (m, 18H)
Galactose-2	4.48 (d, J = 8.3 Hz, 1H)	3.60	3.75	4.17	n/a	4.01, 3.54	-	-	-	-
GlcNAc-6S-2	4.72 (d, J = 8.7 Hz, 1H)	3.83	n/a	n/a	3.81	4.36 (2H)	-	-	-	2.08 – 1.99 (m, 18H)
Galactose-3	4.63 (d, J = 7.3 Hz, 1H)	3.57	4.13 (dd, J = 10.2, 3.0 Hz, 1H)	3.98 (d, J = 3.6 Hz, 1H)	n/a	3.74 (2H)	-	-	-	-
Sialic acid-1	-	-	2.70 – 2.63 (m, 2H), 1.78 – 1.66 (m, 2H)	3.68	3.81	n/a	n/a	3.91	3.89, 3.66	2.08 – 1.99 (m, 18H)
Sialic acid-2	-	-	2.70 – 2.63 (m, 2H), 1.78 – 1.66 (m, 2H)	3.68	3.81	n/a	n/a	3.91	3.89, 3.66	2.08 – 1.99 (m, 18H)
Sialic acid-3	-	-	2.76 (dd, J = 11.9, 4.8 Hz, 1H), 1.82 (t, J = 12.1 Hz, 1H)	3.70	3.87	n/a	n/a	3.91	3.89, 3.66	2.08 – 1.99 (m, 18H)

^{13}C (150 MHz, D_2O): δ (ppm)

	C-1	C-2	C-3	C-4	C-5	C-6	C-7	C-8	C-9	NHAc
GlcNAc	100.80	54.89	n/a	n/a	74.38	60.52	-	-	-	22.20
Galactose-1	103.51	69.33	82.48	67.99	n/a	63.52	-	-	-	-
GlcNAc-6S-1	102.54	54.92	n/a	n/a	73.47	66.70	-	-	-	22.20
Galactose-2	103.47	68.96	82.48	67.99	n/a	63.52	-	-	-	-
GlcNAc-6S-2	102.97	54.92	n/a	n/a	72.54	66.13	-	-	-	22.20
Galactose-3	102.04	69.79	75.29	67.78	n/a	60.97	-	-	-	-
Sialic acid-1	n/a	n/a	40.24	n/a	51.79	n/a	n/a	71.63	62.53	22.20
Sialic acid-2	n/a	n/a	40.24	n/a	51.79	n/a	n/a	71.63	62.53	22.20
Sialic acid-3	n/a	n/a	39.77	n/a	51.88	n/a	n/a	71.63	62.53	22.20

Linker	1	2	3	4	5	6
H	3.88,3.58	1.61 – 1.52 (m, 2H)	1.37 – 1.24 (m, 2H)	1.50 (p, J = 7.2 Hz, 2H)	3.13 (t, J = 6.8 Hz, 2H)	5.12 (s, 2H)
C	70.22	28.28	22.44	28.49	40.43	66.77

HRMS (ESI-MS): m/z calculated for $C_{88}H_{137}N_7O_{63}S_2$ $[M-2H]^{2-}$: 1181.8592; found: 1181.8421.

Compound 18

18 was prepared from **17** (3.0 mg, 1.3 μmol) using the general procedure for the 6-O-sulfate installation of internal Galactose using CHST1. After P6 purification, **18** was obtained as a white solid (2.7 mg, 88%).

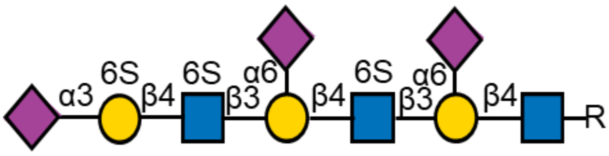

^{1}H (600 MHz, D_2O): δ (ppm)

	H-1	H-2	H-3	H-4	H-5	H-6	H-7	H-8	H-9	NHAc
GlcNAc	4.54 (d, J = 7.9 Hz, 1H)	3.73	n/a	n/a	3.59	3.99, 3.82	-	-	-	2.08 – 1.98 (m, 18H)
Galactose-1	4.44 (d, J = 7.9 Hz, 1H)	3.61	3.74	4.18	n/a	4.01, 3.56	-	-	-	-

GlcNAc-6S-1	4.75 (d, J = 7.3 Hz, 1H)	3.82	n/a	n/a	3.83	4.40 (d, J = 11.6 Hz, 1H), 4.31 – 4.27 (m, 1H)	-	-	-	2.08 – 1.98 (m, 18H)
Galactose-2	4.48 (d, J = 7.8 Hz, 1H)	3.60	3.74	4.17	n/a	4.01, 3.56	-	-	-	-
GlcNAc-6S-2	4.71 (d, J = 7.9 Hz, 1H)	3.83	n/a	n/a	3.83	4.36 – 4.32 (m, 2H)	-	-	-	2.08 – 1.98 (m, 18H)
Galactose-6S	4.64 (d, J = 7.8 Hz, 1H)	3.57	4.16	4.03	3.99	4.18 (2H)	-	-	-	-
Sialic acid-1	-	-	2.66 (dd, J = 12.5, 4.6 Hz, 2H), 1.75 (td, J = 12.2, 7.4 Hz, 2H)	3.68	3.82	n/a	n/a	3.91	3.88, 3.65	2.08 – 1.98 (m, 18H)
Sialic acid-2	-	-	2.66 (dd, J = 12.5, 4.6 Hz, 2H), 1.75 (td, J = 12.2, 7.4 Hz, 2H)	3.68	3.82	n/a	n/a	3.91	3.88, 3.65	2.08 – 1.98 (m, 18H)
Sialic acid-3	-	-	2.75 (dd, J = 12.4, 4.7 Hz, 1H), 1.83	3.70	3.87	n/a	n/a	3.91	3.88, 3.65	2.08 – 1.98 (m, 18H)

^{13}C (150 MHz, D_2O): δ (ppm)

	C-1	C-2	C-3	C-4	C-5	C-6	C-7	C-8	C-9	NHAc
GlcNAc	100.91	54.94	n/a	n/a	74.56	60.45	-	-	-	22.16
Galactose-1	103.46	69.42	82.48	68.65	n/a	63.39	-	-	-	-
GlcNAc-6S-1	102.55	54.93	n/a	n/a	73.39	66.70	-	-	-	22.16
Galactose-2	103.39	69.28	82.48	68.65	n/a	63.39	-	-	-	-
GlcNAc-6S-2	102.86	54.93	n/a	n/a	73.39	66.47	-	-	-	22.16
Galactose-6S	102.55	69.54	75.21	67.33	72.50	66.50	-	-	-	-

Sialic acid-1	n/a	n/a	40.12	n/a	51.89	n/a	n/a	71.48	62.78	22.16
Sialic acid-2	n/a	n/a	40.12	n/a	51.89	n/a	n/a	71.48	62.78	22.16
Sialic acid-3	n/a	n/a	39.52	n/a	51.79	n/a	n/a	71.48	62.78	22.16

Linker	1	2	3	4	5	6
H	3.88,3.58	1.61 – 1.52 (m, 2H)	1.37 – 1.24 (m, 2H)	1.52 – 1.46 (m, 1H)	3.19 – 3.07 (m, 1H)	5.12 (s, 2H)
C	70.22	28.28	22.44	28.49	40.43	66.77

HRMS (ESI-MS): m/z calculated for $C_{88}H_{136}N_7O_{66}S_3$ $[M-3H]^{3-}$: 814.2226; found: 814.1956.

Compound S15

S15 was prepared from **18** (2.7 mg, 1.1 µmol) using the general procedure for installation of α2,3Neu5Ac using PmST1 M144D. After P6 purification, **S15** was obtained as a white solid (1.7 mg, quant).

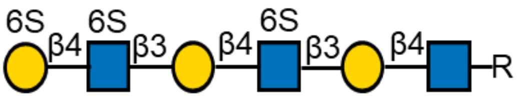

^{1}H (600 MHz, D_2O): δ (ppm)

	H-1	H-2	H-3	H-4	H-5	H-6	NHAc
GlcNAc	4.51	3.71	3.70	3.69	3.58	3.99，3.82	2.07 – 2.00 (m, 9H)
Galactose-1	4.47 (d, J = 7.9 Hz, 1H)	3.60	3.73	4.20	n/a	3.76 (4H)	-
GlcNAc-6S-1	4.72 (d, J = 8.4 Hz, 2H)	3.84	3.77	3.78	3.83	4.41 (d, *J* = 10.8 Hz, 2H), 4.31 (td, *J* = 10.6, 4.9 Hz, 2H)	2.07 – 2.00 (m, 9H)
Galactose-2	4.52	3.60	3.73	4.20	n/a	3.76 (4H)	-
GlcNAc-6S-2	4.72 (d, J = 8.4 Hz, 2H)	3.84	3.77	3.78	3.83	4.41 (d, *J* = 10.8 Hz, 2H), 4.31 (td, *J* = 10.6, 4.9 Hz, 2H)	2.07 – 2.00 (m, 9H)

Galactose-6S	4.55 (d, J = 7.9 Hz, 1H)	3.55	3.72	4.00 (d, J = 3.6 Hz, 1H)	3.99	4.21 (2H)	-

^{13}C (150 MHz, D_2O): δ (ppm)

	C-1	C-2	C-3	C-4	C-5	C-6	NHAc
GlcNAc	100.78	55.09	72.61	78.65	74.81	60.10	22.19
Galactose-1	102.73	69.85	82.57	68.75	n/a	61.06	-
GlcNAc-6S-1	102.84	55.01	n/a	n/a	72.53	66.66	22.19
Galactose-2	102.87	69.85	82.57	68.75	n/a	61.06	-
GlcNAc-6S-2	102.84	55.01	n/a	n/a	72.53	66.66	22.19
Galactose-6S	102.91	71.02	n/a	68.28	72.86	66.42	-

Linker	1	2	3	4	5	6
H	3.88, 3.55	1.59 – 1.52 (m, 2H)	1.35 – 1.26 (m, 2H)	1.49 (p, J = 7.2 Hz, 2H)	3.12 (t, J = 6.8 Hz, 1H)	5.12 (s, 2H)
C	70.33	28.24	22.42	28.53	40.53	66.56

HRMS (ESI-MS): m/z calculated for $C_{55}H_{85}N_4O_{42}S_3$ $[M-3H]^{3-}$: 523.1272; found: 523.1110.

Compound 14

14 was prepared from **S15** (0.6 mg, 0.4 µmol) using the general procedure for installation of α2,3Neu5Ac using PmST1 M144D. After P6 purification, **14** was obtained as a white solid (0.6 mg, 80%). NMR data are in agreement with previous reported data for compound **14**.

Compound S11

S11 was prepared from **27** (3.0 mg, 3.4 µmol) using the general procedure for installation of α1,3Fuc using FUT6. After P6 purification, **S11** was obtained as a white solid (3.4 mg, 98%).

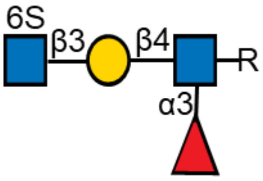

^{1}H (600 MHz, D_2O): δ (ppm)

	H-1	H-2	H-3	H-4	H-5	H-6	NHAc
GlcNAc	4.52 (d, J = 7.9 Hz, 1H)	3.87	n/a	n/a	3.58	3.99，3.85	2.05 – 1.96 (m, 6H)
Galactose	4.45 (d, J = 7.9 Hz, 1H)	3.53	3.71	4.13 (d, J = 3.2 Hz, 1H)	n/a	3.73 (2H)	-
GlcNAc-6S	4.70 (d, J = 8.5 Hz, 1H)	3.79	3.59	3.54	3.67	4.34 (d, J = 11.1 Hz, 1H), 4.23 (dd, J = 11.2, 5.5 Hz, 1H)	2.05 – 1.96 (m, 6H)
Fucose	5.09 (d, J = 4.0 Hz, 1H)	3.70	n/a	n/a	4.84	1.16 (d, J = 6.6 Hz, 3H)	-

^{13}C (150 MHz, D_2O): δ (ppm)

	C-1	C-2	C-3	C-4	C-5	C-6	NHAc
GlcNAc	100.93	55.83	n/a	n/a	74.61	60.00	22.18
Galactose	101.71	69.70	82.13	68.30	n/a	61.70	-
GlcNAc-6S	102.88	55.54	73.83	69.91	73.73	67.15	22.18
Fucose	98.61	n/a	n/a	n/a	66.69	15.26	-

Linker	1	2	3	4	5	6
H	3.87,3.57	1.61 – 1.52 (m, 2H)	1.37 – 1.24 (m, 2H)	1.49 (p, J = 7.3 Hz, 2H)	3.16 – 3.10 (m, 2H)	5.12 (s, 2H)
C	70.35	28.22	22.45	28.46	40.38	66.71

HRMS (ESI-MS): m/z calculated for $C_{41}H_{64}N_3O_{25}S$ $[M-H]^-$: 1030.3555; found: 1030.3237.

Compound S12

S12 was prepared from **S11** (3.4 mg, 3.3 μmol) using the general procedure for installation of β1,4Gal using B4GalT4. After P6 purification, **S12** was obtained as a white solid (3.9 mg, quant).

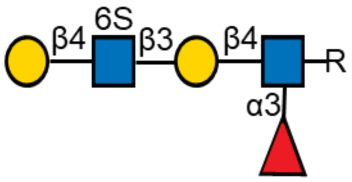

^{1}H (600 MHz, D_2O): δ (ppm)

	H-1	H-2	H-3	H-4	H-5	H-6	NHAc
GlcNAc	4.52	3.87	n/a	n/a	3.58	3.99 (d, J = 12.0 Hz, 1H), 3.85	2.06 – 1.98 (m, 6H)
Galactose-1	4.45 (d, J = 8.2 Hz, 1H)	3.53	3.71	4.13 (d, J = 2.9 Hz, 1H)	n/a	3.73 (2H)	-
GlcNAc-6S	4.73 (d, J = 8.5 Hz, 1H)	3.83	3.75	3.80	3.81	4.40 (d, J = 11.1 Hz, 1H), 4.33 (d, J = 10.9 Hz, 1H)	2.06 – 1.98 (m, 6H)
Galactose-2	4.54 (d, J = 8.3 Hz, 1H)	3.54	3.69	3.94 (d, J = 3.2 Hz, 1H)	n/a	3.76 (2H)	-
Fucose	5.09 (d, J = 3.9 Hz, 1H)	3.70	n/a	n/a	4.83	1.16 (d, J = 6.7 Hz, 3H)	-

^{13}C (150 MHz, D_2O): δ (ppm)

	C-1	C-2	C-3	C-4	C-5	C-6	NHAc
GlcNAc	100.78	55.98	n/a	n/a	74.84	59.87	22.21
Galactose-1	101.82	69.72	82.13	68.14	n/a	61.52	-
GlcNAc-6S	102.88	55.35	72.24	77.60	72.58	66.44	22.21
Galactose-2	102.85	71.78	72.27	68.81	n/a	61.03	-
Fucose	98.61	n/a	n/a	n/a	66.83	15.39	-

Linker	1	2	3	4	5	6
H	3.87,3.57	1.61 – 1.52 (m, 2H)	1.37 – 1.24 (m, 2H)	1.49 (p, J = 7.3 Hz, 2H)	3.16 – 3.10 (m, 2H)	5.12 (s, 2H)
C	70.35	28.22	22.45	28.46	40.38	66.71

HRMS (ESI-MS): m/z calculated for $C_{47}H_{74}N_3O_{30}S$ $[M-H]^-$: 1192.4083; found: 1192.3881.

Compound S13

S13 was prepared from **S12** (3.0 mg, 2.5 μmol) using the general procedure for installation of β1,3GlcNAc using B3GnT2. After P6 purification, **S13** was obtained as a white solid (3.1 mg, 89%).

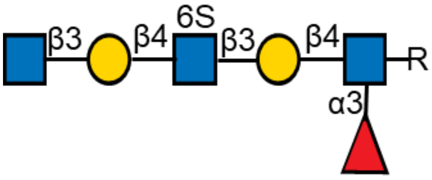

^{1}H (600 MHz, D_2O): δ (ppm)

	H-1	H-2	H-3	H-4	H-5	H-6	NHAc
GlcNAc-1	4.52	3.87	n/a	n/a	3.58	3.99 (d, J = 10.9 Hz, 1H)， 3.84	2.06 – 1.98 (m, 9H)
Galactose-1	4.44 (d, J = 7.8 Hz, 1H)	3.53	3.71	4.13 (d, J = 3.3 Hz, 1H)	n/a	3.73 (2H)	-
GlcNAc-6S	4.72 (d, J = 8.4 Hz, 1H)	3.83	3.74	3.80	3.81	4.39 (d, J = 10.8 Hz, 1H), 4.32 (dd, J = 11.0, 3.9 Hz, 1H)	2.06 – 1.98 (m, 9H)
Galactose-2	4.53	3.58	3.74	4.16 (d, J = 3.3 Hz, 1H)	n/a	3.77 (2H)	-
GlcNAc-2	4.70 (d, J = 8.4 Hz, 1H)	3.77	n/a	n/a	3.47	3.90, 3.78	2.06 – 1.98 (m, 9H)
Fucose	5.09 (d, J = 4.0 Hz, 1H)	3.70	n/a	n/a	4.83	1.16 (d, J = 6.6 Hz, 3H)	-

^{13}C (150 MHz, D_2O): δ (ppm)

	C-1	C-2	C-3	C-4	C-5	C-6	NHAc
GlcNAc-1	100.86	55.90	n/a	n/a	74.82	59.80	22.13
Galactose-1	101.95	70.46	82.14	68.14	n/a	61.45	-
GlcNAc-6S	102.66	55.27	72.13	77.69	72.68	66.36	22.13
Galactose-2	103.11	69.89	82.01	68.35	n/a	60.77	-
GlcNAc-2	102.90	55.77	n/a	n/a	n/a	60.35	22.13
Fucose	98.71	n/a	n/a	n/a	66.67	15.39	-

Linker	1	2	3	4	5	6
H	3.87,3.57	1.61 – 1.52 (m, 2H)	1.37 – 1.24 (m, 2H)	1.49 (p, J = 7.3 Hz, 2H)	3.16 – 3.10 (m, 2H)	5.12 (s, 2H)
C	70.35	28.22	22.45	28.46	40.38	66.71

HRMS (ESI-MS): m/z calculated for $C_{55}H_{87}N_4O_{35}S$ $[M-H]^-$: 1395.4877; found: 1395.4539.

Compound 19

19 was prepared from **S13** (3.1 mg, 2.2 μmol) using the general procedure for 6-O-sulfate installation of terminal GlcNAc using CHST2. After P6 purification, **19** was obtained as a white solid (3.2 mg, 96%).

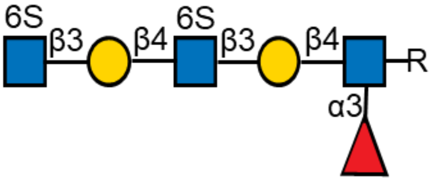

^1H (600 MHz, D_2O): δ (ppm)

	H-1	H-2	H-3	H-4	H-5	H-6	NHAc
GlcNAc	4.52	3.87	n/a	n/a	3.58	3.99 (d, J = 10.1 Hz, 1H)，3.85	2.06 – 1.96 (m, 9H)
Galactose-1	4.45 (d, J = 7.9 Hz, 1H)	3.53	3.71	4.13 (d, J = 3.3 Hz, 1H)	n/a	3.73 (2H)	-
GlcNAc-6S-1	4.72 (d, J = 8.4 Hz, 1H)	3.83	3.74	3.79	3.80	4.42 – 4.38 (m, 1H), 4.31	2.06 – 1.96 (m, 9H)
Galactose-2	4.52	3.58	3.73	4.20 (d, J = 3.3 Hz, 1H)	n/a	3.76 (2H)	-
GlcNAc-6S -2	4.70 (d, J = 8.5 Hz, 1H)	3.80	3.59	3.53	3.67	4.33, 4.24 (dd, J = 11.2, 5.3 Hz, 1H)	2.06 – 1.96 (m, 9H)
Fucose	5.09 (d, J = 4.0 Hz, 1H)	3.70	n/a	n/a	4.83	1.16 (d, J = 6.6 Hz, 3H)	-

^{13}C (150 MHz, D_2O): δ (ppm)

	C-1	C-2	C-3	C-4	C-5	C-6	NHAc
GlcNAc	100.86	55.75	n/a	n/a	74.82	59.80	22.19
Galactose-1	101.70	70.07	81.91	68.18	n/a	61.53	-
GlcNAc-6S-1	102.71	55.27	72.21	77.94	72.68	66.49	22.19
Galactose-2	103.11	69.82	82.73	68.35	n/a	61.12	-
GlcNAc-6S -2	102.90	56.02	73.42	69.62	73.60	66.89	22.19
Fucose	98.66	n/a	n/a	n/a	66.73	15.37	-

Linker	1	2	3	4	5	6
H	3.87,3.57	1.61 – 1.52 (m, 2H)	1.37 – 1.24 (m, 2H)	1.49 (p, J = 7.3 Hz, 2H)	3.16 – 3.10 (m, 2H)	5.12 (s, 2H)
C	70.35	28.22	22.45	28.46	40.38	66.71

HRMS (ESI-MS): m/z calculated for $C_{55}H_{86}N_4O_{38}S_2$ $[M-2H]^{2-}$: 737.2186; found: 737.1830.

Compound 20

20 was prepared from **19** (3.2 mg, 2.2 μmol) using the general procedure for installation of α2,6Neu5Ac using PT2,6ST. After P6 purification, **20** was obtained as a white solid (1.9 mg, 51%). Unreacted **19** was recovered.

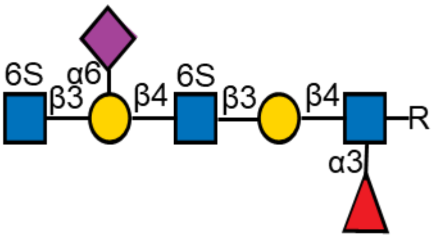

^{1}H (600 MHz, D_2O): δ (ppm)

	H-1	H-2	H-3	H-4	H-5	H-6	H-7	H-8	H-9	NHAc
GlcNAc	4.52 (d, J = 8.2 Hz, 1H)	3.89	n/a	n/a	3.58	3.99, 3.85	-	-	-	2.08 – 1.98 (m, 12H)
Galactose-1	4.45 (d, J = 8.0 Hz, 1H)	3.53	3.72	4.13 (d, J = 3.3 Hz, 1H)	n/a	3.73 (2H)	-	-	-	-
GlcNAc-6S-1	4.74	3.83	n/a	n/a	3.85	4.43 (d, J = 10.8 Hz, 1H), 4.30	-	-	-	2.08 – 1.98 (m, 12H)
Galactose-2	4.47 (d, J = 8.6 Hz, 1H)	3.60	3.74	4.17	n/a	4.01, 3.53	-	-	-	-
GlcNAc-6S -2	4.71	3.79	3.57	3.53	3.66	4.34 – 4.24 (m, 2H)	-	-	-	2.08 – 1.98 (m, 12H)
Fucose	5.10 (d, J = 3.8 Hz, 1H)	3.70	n/a	n/a	4.83	1.16 (d, J = 6.3 Hz, 3H)	-	-	-	-
Sialic acid	-	-	2.67 (dd, J = 12.4, 4.6 Hz, 1H), 1.73 (t, J = 12.2 Hz, 1H)	3.67	3.82	n/a	n/a	3.91	3.88, 3.65	2.08 – 1.98 (m, 12H)

^{13}C (150 MHz, D_2O): δ (ppm)

	C-1	C-2	C-3	C-4	C-5	C-6	C-7	C-8	C-9	NHAc
GlcNAc	100.89	56.23	n/a	n/a	74.82	59.86	-	-	-	22.24
Galactose-1	102.04	70.47	81.89	68.29	n/a	61.61	-	-	-	-
GlcNAc-6S-1	102.43	54.64	n/a	n/a	73.75	67.13	-	-	-	22.24
Galactose-2	103.63	69.53	82.57	68.29	n/a	63.56	-	-	-	-
GlcNAc-6S -2	102.93	55.70	73.84	69.79	73.35	66.89	-	-	-	22.24
Fucose	98.66	n/a	n/a	n/a	66.68	15.27	-	-	-	-
Sialic acid	n/a	n/a	40.08	n/a	51.97	n/a	n/a	71.79	62.63	22.24

Linker	1	2	3	4	5	6
H	3.87,3.57	1.61 – 1.52 (m, 2H)	1.37 – 1.24 (m, 2H)	1.49 (p, J = 7.3 Hz, 2H)	3.16 – 3.10 (m, 2H)	5.12 (s, 2H)
C	70.35	28.22	22.45	28.46	40.38	66.71

HRMS (ESI-MS): m/z calculated for $C_{66}H_{103}N_5O_{46}S_2$ $[M-2H]^{2-}$: 882.7663; found: 882.7527.

Compound 21

21 was prepared from **20** (2.8 mg, 1.6 μmol) using the general procedure for β1,4Gal using Hpβ4GalT. After P6 purification, **21** was obtained as a white solid (3.0 mg, quant).

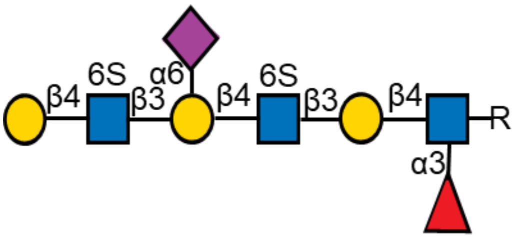

^1H (600 MHz, D_2O): δ (ppm)

	H-1	H-2	H-3	H-4	H-5	H-6	H-7	H-8	H-9	NHAc
GlcNAc	4.52 (d, J = 8.4 Hz, 1H)	3.88	n/a	n/a	3.59	3.99, 3.85	-	-	-	2.08 – 1.98 (m, 12H)
Galactose-1	4.45 (d, J = 8.0 Hz, 1H)	3.54	3.72	4.14 (d, J = 3.3 Hz, 1H)	n/a	3.73 (2H)	-	-	-	-
GlcNAc-6S-1	4.74	3.83	n/a	n/a	3.85	4.42 (d, J = 10.9 Hz, 1H),	-	-	-	2.08 – 1.98 (m, 12H)

						4.28 (dd, J = 11.1, 5.7 Hz, 1H)				
Galactose-2	4.47 (d, J = 8.1 Hz, 1H)	3.60	3.74	4.17 (d, J = 3.1 Hz, 1H)	n/a	4.01, 3.54	-	-	-	-
GlcNAc-6S -2	4.74	3.83	n/a	n/a	3.81	4.36 (2H)	-	-	-	2.08 – 1.98 (m, 12H)
Galactose-3	4.55 (d, J = 7.6 Hz, 1H)	3.54	3.69	3.93	n/a	3.77 (2H)	-	-	-	-
Fucose	5.10 (d, J = 4.1 Hz, 1H)	3.70	n/a	n/a	4.83	1.16 (d, J = 6.4 Hz, 3H)	-	-	-	-
Sialic acid	-	-	2.67 (dd, J = 12.5, 2.8 Hz, 1H), 1.73 (t, J = 12.1 Hz, 1H)	3.67	3.82	n/a	n/a	3.91	3.88, 3.65	2.08 – 1.98 (m, 12H)

^{13}C (150 MHz, D_2O): δ (ppm)

	C-1	C-2	C-3	C-4	C-5	C-6	C-7	C-8	C-9	NHAc
GlcNAc	101.08	55.75	n/a	n/a	74.72	59.81	-	-	-	22.15
Galactose-1	101.83	70.55	82.01	68.16	n/a	61.46	-	-	-	-
GlcNAc-6S-1	102.75	54.88	n/a	n/a	73.88	66.87	-	-	-	22.15
Galactose-2	103.55	69.41	82.59	68.09	n/a	63.52	-	-	-	-
GlcNAc-6S -2	102.75	54.88	n/a	n/a	72.43	66.27	-	-	-	22.15
Galactose-3	102.39	71.64	72.57	68.89	n/a	60.87	-	-	-	-
Fucose	98.62	n/a	n/a	n/a	66.68	15.25	-	-	-	-
Sialic acid	n/a	n/a	40.19	n/a	51.94	n/a	n/a	71.91	62.66	22.15

Linker	1	2	3	4	5	6
H	3.87,3.57	1.61 – 1.52 (m, 2H)	1.37 – 1.24 (m, 2H)	1.49 (p, J = 7.3 Hz, 2H)	3.16 – 3.10 (m, 2H)	5.12 (s, 2H)
C	70.35	28.22	22.45	28.46	40.38	66.71

HRMS (ESI-MS): m/z calculated for $C_{72}H_{113}N_5O_{51}S_2$ $[M-2H]^{2-}$: 963.7927; found: 963.7478.

Compound 22

22 was prepared from **21** (3.0 mg, 1.6 μmol) using the general procedure installation of α2,3Neu5Ac using PmST1 M144D. After P6 purification, **22** was obtained as a white solid (3.1 mg, 90%).

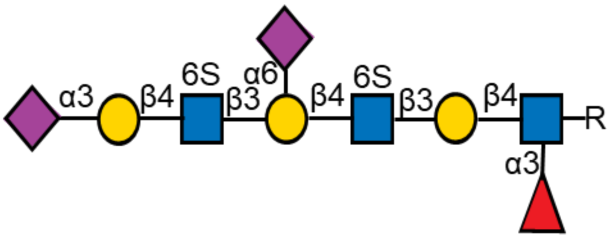

^{1}H (600 MHz, D_2O): δ (ppm)

	H-1	H-2	H-3	H-4	H-5	H-6	H-7	H-8	H-9	NHAc
GlcNAc	4.52 (d, J = 7.8 Hz, 1H)	3.88	n/a	n/a	3.59	3.99, 3.85	-	-	-	2.08 – 1.98 (m, 15H)
Galactose-1	4.45 (d, J = 8.1 Hz, 1H)	3.54	3.73	4.14 (d, J = 3.1 Hz, 1H)	n/a	3.73 (2H)	-	-	-	-
GlcNAc-6S-1	4.74	3.83	n/a	n/a	3.85	4.43 (d, J = 11.6 Hz, 1H), 4.28 (dd, J = 11.1, 5.7 Hz, 1H)	-	-	-	2.08 – 1.98 (m, 15H)
Galactose-2	4.47 (d, J = 8.3 Hz, 1H)	3.60	3.74	4.17	n/a	4.01, 3.54	-	-	-	-
GlcNAc-6S -2	4.73	3.83	n/a	n/a	3.81	4.36 (2H)	-	-	-	2.08 – 1.98 (m, 15H)
Galactose-3	4.63 (d, J = 7.8 Hz, 1H)	3.57	4.13	3.98	n/a	3.74 (2H)	-	-	-	-
Fucose	5.10 (d, J = 4.0 Hz, 1H)	3.70	n/a	n/a	4.83	1.16 (d, J = 6.6 Hz, 3H)	-	-	-	-
Sialic acid-1	-	-	2.67 (d, J = 11.9 Hz, 1H), 1.74 (t, J = 12.0	3.70	3.82	n/a	n/a	3.91	3.89, 3.66	2.08 – 1.98 (m, 15H)

			Hz, 1H)							
Sialic acid-2	-	-	2.76 (dd, J = 12.6, 3.9 Hz, 1H), 1.82 (t, J = 12.1 Hz, 1H)	3.68	3.86	n/a	n/a	3.91	3.89, 3.66	2.08 – 1.98 (m, 15H)

^{13}C (150 MHz, D_2O): δ (ppm)

	C-1	C-2	C-3	C-4	C-5	C-6	C-7	C-8	C-9	NHAc
GlcNAc	100.93	55.88	n/a	n/a	74.72	59.81	-	-	-	22.21
Galactose-1	101.94	70.33	82.15	68.27	n/a	61.53	-	-	-	-
GlcNAc-6S-1	102.70	54.97	n/a	n/a	73.63	67.02	-	-	-	22.21
Galactose-2	103.55	69.08	82.66	68.13	n/a	63.56	-	-	-	-
GlcNAc-6S-2	103.08	54.97	n/a	n/a	72.16	66.35	-	-	-	22.21
Galactose-3	102.18	69.52	75.40	67.67	n/a	60.78	-	-	-	-
Fucose	98.53	n/a	n/a	n/a	66.86	15.40	-	-	-	-
Sialic acid-1	n/a	n/a	40.21	n/a	51.89	n/a	n/a	71.69	62.47	22.21
Sialic acid-2	n/a	n/a	39.75	n/a	51.76	n/a	n/a	71.69	62.47	22.21

Linker	1	2	3	4	5	6
H	3.87,3.57	1.61 – 1.52 (m, 2H)	1.37 – 1.24 (m, 2H)	1.49 (p, J = 7.3 Hz, 2H)	3.16 – 3.10 (m, 2H)	5.12 (s, 2H)
C	70.35	28.22	22.45	28.46	40.38	66.71

HRMS (ESI-MS): m/z calculated for $C_{83}H_{130}N_6O_{59}S_2$ $[M-2H]^{2-}$: 1109.3404; found: 1109.3257.

Compound 23

23 was prepared from **22** (3.1 mg, 1.4 μmol) using the general procedure for 6-O-sulfate installation of internal Galactose using CHST1. After P6 purification, **23** was obtained as a white solid (2.9 mg, 88%).

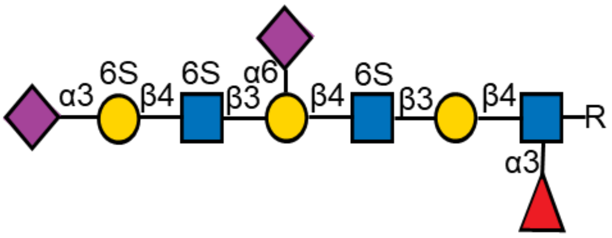

^{1}H (600 MHz, D_2O): δ (ppm)

	H-1	H-2	H-3	H-4	H-5	H-6	H-7	H-8	H-9	NHAc
GlcNAc	4.52 (d, J = 8.2 Hz, 1H)	3.88	n/a	n/a	3.59	3.99, 3.85	-	-	-	2.08 – 1.98 (m, 15H)
Galactose-1	4.45	3.54	3.72	4.14	n/a	3.73 (2H)	-	-	-	-
GlcNAc-6S-1	4.74	3.85	n/a	n/a	3.85	4.42, 4.30 – 4.25 (m, 1H)	-	-	-	2.08 – 1.98 (m, 15H)
Galactose-2	4.47	3.60	3.73	4.17	n/a	4.02, 3.54	-	-	-	-
GlcNAc-6S-2	4.73	3.84	n/a	n/a	3.82	4.39, 4.36 – 4.31 (m, 1H)	-	-	-	2.08 – 1.98 (m, 15H)
Galactose-6S	4.64 (d, J = 7.9 Hz, 1H)	3.57	4.16	4.03	3.99	4.19 (2H)	-	-	-	-
Fucose	5.10 (d, J = 4.0 Hz, 1H)	3.70	n/a	n/a	4.83	1.16 (d, J = 6.3 Hz, 3H)	-	-	-	-
Sialic acid-1	-	-	2.66 (d, J = 12.1 Hz, 1H), 1.74 (t, J = 12.1 Hz, 1H)	3.67	3.83	n/a	n/a	3.91	3.90, 3.66	2.08 – 1.98 (m, 15H)
Sialic acid-2	-	-	2.75 (d, J = 10.9 Hz, 1H), 1.82 (t, J =	3.70	3.87	n/a	n/a	3.91	3.90, 3.66	2.08 – 1.98 (m, 15H)

			12.0 Hz, 1H)							

^{13}C (150 MHz, D_2O): δ (ppm)

	C-1	C-2	C-3	C-4	C-5	C-6	C-7	C-8	C-9	NHAc
GlcNAc	100.88	55.63	n/a	n/a	74.72	59.88	-	-	-	22.23
Galactose-1	101.94	70.48	82.15	68.27	n/a	61.64	-	-	-	-
GlcNAc-6S-1	102.70	55.03	n/a	n/a	73.66	67.02	-	-	-	22.23
Galactose-2	103.55	69.21	82.66	68.17	n/a	63.63	-	-	-	-
GlcNAc-6S-2	103.08	54.99	n/a	n/a	72.69	66.60	-	-	-	22.23
Galactose-6S	102.57	69.04	75.14	67.56	72.89	66.76	-	-	-	-
Fucose	98.61	n/a	n/a	n/a	66.78	15.52	-	-	-	-
Sialic acid-1	n/a	n/a	40.09	n/a	51.88	n/a	n/a	71.69	62.72	22.23
Sialic acid-2	n/a	n/a	39.57	n/a	51.81	n/a	n/a	71.69	62.72	22.23

Linker	1	2	3	4	5	6
H	3.87,3.57	1.61 – 1.52 (m, 2H)	1.37 – 1.24 (m, 2H)	1.49 (p, J = 7.3 Hz, 2H)	3.16 – 3.10 (m, 2H)	5.12 (s, 2H)
C	70.35	28.22	22.45	28.46	40.38	66.71

HRMS (ESI-MS): m/z calculated for $C_{83}H_{129}N_6O_{62}S_3$ $[M-3H]^{3-}$: 765.8768; found: 765.8531.

Compound S16

S16 was prepared from **23** (1.9 mg, 0.8 µmol) using the general procedure for the removal of sialic acid using *C. perfringens* neuraminidase. After P6 purification, **S16** was obtained as a white solid (1.4 mg, quant).

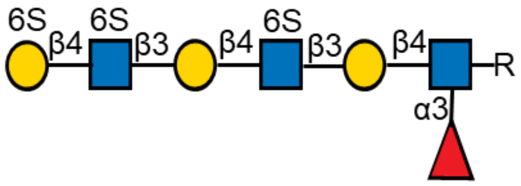

^{1}H (600 MHz, D_2O): δ (ppm)

	H-1	H-2	H-3	H-4	H-5	H-6	NHAc
GlcNAc	4.52 (d, J = 7.8 Hz, 2H)	3.87	n/a	n/a	3.58	3.99, 3.85	2.06 – 1.97 (m, 9H)

Galactose-1	4.45 (d, J = 7.9 Hz, 1H)	3.53	3.72	4.13 (d, J = 3.3 Hz, 1H)	n/a	3.73 (2H)	-
GlcNAc-6S-1	4.72	3.83	n/a	n/a	3.83	4.42 – 4.38 (m, 2H), 4.36 – 4.29 (m, 2H)	2.06 – 1.97 (m, 9H)
Galactose-2	4.52 (d, J = 7.8 Hz, 2H)	3.59	3.73	4.20	n/a	3.77 (2H)	-
GlcNAc-6S -2	4.72	3.83	n/a	n/a	3.83	4.42 – 4.38 (m, 2H), 4.36 – 4.29 (m, 2H)	2.06 – 1.97 (m, 9H)
Galactose-6S	4.55 (d, J = 7.7 Hz, 1H)	3.55	3.72	4.00	3.99	4.21 (2H)	-
Fucose	5.09 (d, J = 3.9 Hz, 1H)	3.69	n/a	n/a	4.82	1.16 (d, J = 6.6 Hz, 3H)	-

^{13}C (150 MHz, D_2O): δ (ppm)

	C-1	C-2	C-3	C-4	C-5	C-6	NHAc
GlcNAc	100.76	55.94	n/a	n/a	74.59	59.78	22.23
Galactose-1	101.79	70.55	82.44	68.28	n/a	61.64	-
GlcNAc-6S-1	102.72	55.09	n/a	n/a	72.62	66.58	22.23
Galactose-2	102.03	69.87	82.47	69.17	n/a	61.09	-
GlcNAc-6S -2	102.72	55.09	n/a	n/a	72.62	66.58	22.23
Galactose-6S	102.69	71.48	n/a	68.38	72.90	66.51	-
Fucose	98.67	n/a	n/a	n/a	66.73	15.31	-

Linker	1	2	3	4	5	6
H	3.87,3.57	1.61 – 1.52 (m, 2H)	1.37 – 1.24 (m, 2H)	1.49 (p, J = 7.3 Hz, 2H)	3.16 – 3.10 (m, 2H)	5.12 (s, 2H)
C	70.35	28.22	22.45	28.46	40.38	66.71

HRMS (ESI-MS): m/z calculated for $C_{61}H_{95}N_4O_{46}S_3$ $[M-3H]^{3-}$: 571.8132; found: 571.7962.

Compound 24

24 was prepared from **S16** (1.4 mg, 0.8 μmol) using the general procedure for installation of α2,3Neu5Ac using PmST1 M144D. After P6 purification, **24** was obtained as a white solid (1.6 mg, 97%).

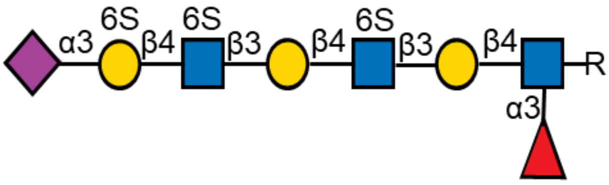

^{1}H (600 MHz, D_2O): δ (ppm)

	H-1	H-2	H-3	H-4	H-5	H-6	H-7	H-8	H-9	NHAc
GlcNAc	4.52 (d, J = 7.8 Hz, 2H)	3.88	3.58	n/a	3.59	3.99, 3.85	-	-	-	2.06 – 1.98 (m, 12H)
Galactose-1	4.45 (d, *J* = 7.8 Hz, 1H)	3.53	3.71	4.13	n/a	3.73 (2H)	-	-	-	-
GlcNAc-6S-1	4.72	3.82	n/a	n/a	3.81	4.42, 4.31	-	-	-	2.06 – 1.98 (m, 12H)
Galactose-2	4.52 (d, J = 7.8 Hz, 2H)	3.58	3.72	4.21	n/a	3.77 (2H)	-	-	-	-
GlcNAc-6S -2	4.71	3.86	n/a	n/a	3.81	4.42, 4.31	-	-	-	2.06 – 1.98 (m, 12H)
Galactose-6S	4.63 (d, *J* = 7.9 Hz, 1H)	3.58	4.16	4.03	3.98	4.19 (2H)	-	-	-	-
Fucose	5.09 (d, J = 4.0 Hz, 1H)	3.70	n/a	n/a	4.83	1.16 (d, J = 6.4 Hz, 3H)	-	-	-	-
Sialic acid	-	-	2.75 (dd, J = 12.8, 4.3 Hz, 1H), 1.82 (t, J = 12.1 Hz, 1H)	3.70	3.87	n/a	n/a	3.91	3.90, 3.66	2.06 – 1.98 (m, 12H)

^{13}C (150 MHz, D_2O): δ (ppm)

	C-1	C-2	C-3	C-4	C-5	C-6	C-7	C-8	C-9	NHAc
GlcNAc	100.82	55.86	75.87	n/a	74.72	59.88	-	-	-	22.16

Galactose-1	101.83	70.46	81.85	68.20	n/a	61.73	-	-	-	-
GlcNAc-6S-1	102.85	55.15	n/a	n/a	72.57	66.70	-	-	-	22.16
Galactose-2	102.21	69.24	82.66	68.61	n/a	61.23	-	-	-	-
GlcNAc-6S-2	102.85	55.30	n/a	n/a	72.57	66.70	-	-	-	22.16
Galactose-6S	102.43	69.24	75.43	67.26	72.61	66.93	-	-	-	-
Fucose	98.40	n/a	n/a	n/a	66.81	15.29	-	-	-	-
Sialic acid	n/a	n/a	39.54	n/a	51.81	n/a	n/a	71.54	62.47	22.16

Linker	1	2	3	4	5	6
H	3.87,3.57	1.61 – 1.52 (m, 2H)	1.37 – 1.24 (m, 2H)	1.49 (p, J = 7.3 Hz, 2H)	3.16 – 3.10 (m, 2H)	5.12 (s, 2H)
C	70.35	28.22	22.45	28.46	40.38	66.71

HRMS (ESI-MS): m/z calculated for $C_{72}H_{112}N_5O_{54}S_3$ $[M-3H]^{3-}$: 668.8450; found: 668.8175.

Compound 14

14 was prepared from **24** (0.6 mg, 0.3 μmol) using the general procedure for removal of fucose using fucosidase from *R. gnavus* E1. After P6 purification, **14** was obtained as a white solid (0.6 mg, quant). NMR data is confirmed with previous made compound **14**.

Compound 25

25 was prepared from **24** (1.0 mg, 0.5 µmol) using the general procedure for 6-O-sulfate installation of internal Galactose using CHST1. After P6 and DEAE purification, **25** was obtained as a white solid (0.7 mg, 68%). Unreacted **24** (0.2 mg, 20%) was recovered.

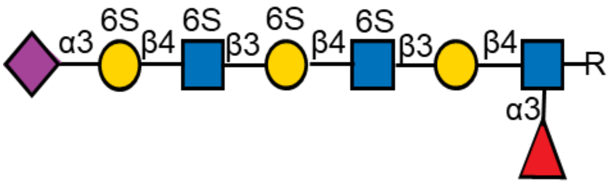

^{1}H (600 MHz, D_2O): δ (ppm)

	H-1	H-2	H-3	H-4	H-5	H-6	H-7	H-8	H-9	NHAc
GlcNAc	4.52 (d, *J* = 7.9 Hz, 1H)	3.89	n/a	n/a	3.59	3.99, 3.85	-	-	-	2.06 – 1.98 (m, 12H)
Galactose	4.45 (d, *J* = 7.9 Hz, 1H)	3.53	3.71	4.13 (d, J = 3.6 Hz, 1H)	n/a	3.73 (2H)	-	-	-	-
GlcNAc-6S-1	4.73	3.82	n/a	n/a	3.81	4.39, 4.32	-	-	-	2.06 – 1.98 (m, 12H)
Galactose-6S-1	4.54 (d, J = 8.0 Hz, 1H)	3.59	3.75	4.25	n/a	4.19 (4H)	-	-	-	-
GlcNAc-6S -2	4.72	3.82	n/a	n/a	3.81	4.42, 4.29	-	-	-	2.06 – 1.98 (m, 12H)
Galactose-6S-2	4.64 (d, *J* = 7.8 Hz, 1H)	3.58	4.16	4.03	3.99	4.19 (4H)	-	-	-	-
Fucose	5.09 (d, J = 4.0 Hz, 1H)	3.69	n/a	n/a	4.83	1.16 (d, J = 6.6 Hz, 3H)	-	-	-	-
Sialic acid	-	-	2.75 (dd, J = 12.5, 4.7 Hz, 1H), 1.82 (t, J = 12.0 Hz, 1H)	3.70	3.87	n/a	n/a	3.91	3.90, 3.66	2.06 – 1.98 (m, 12H)

^{13}C (150 MHz, D_2O): δ (ppm)

	C-1	C-2	C-3	C-4	C-5	C-6	C-7	C-8	C-9	NHAc
GlcNAc	100.82	55.56	n/a	n/a	74.72	59.95	-	-	-	22.16
Galactose	101.75	70.46	82.17	68.24	n/a	61.73	-	-	-	-
GlcNAc-6S-1	102.85	55.03	n/a	n/a	72.57	66.52	-	-	-	22.16
Galactose-6S-1	102.69	69.24	82.35	68.41	n/a	67.24	-	-	-	-
GlcNAc-6S 6S-2	102.85	55.03	n/a	n/a	72.57	66.78	-	-	-	22.16
Galactose-6S-2	102.63	69.24	75.08	67.48	72.69	67.24	-	-	-	-
Fucose	98.64	n/a	n/a	n/a	66.81	15.29	-	-	-	-
Sialic acid	n/a	n/a	39.54	n/a	51.73	n/a	n/a	71.54	62.47	22.16

Linker	1	2	3	4	5	6
H	3.87,3.57	1.61 – 1.52 (m, 2H)	1.37 – 1.24 (m, 2H)	1.49 (p, J = 7.2 Hz, 2H)	3.12 (t, *J* = 6.8 Hz, 2H)	5.12 (s, 2H)
C	70.29	28.29	22.45	28.42	40.34	66.63

HRMS (ESI-MS): m/z calculated for $C_{72}H_{113}N_5O_{57}S_4$ $[M-2H]^{2-}$: 1043.7495; found: 1043.7372.

Compound 26

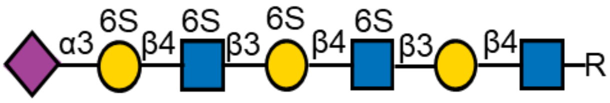

26 was prepared from **25** (0.6 mg, 0.29 µmol) using the general procedure for removal of fucose using fucosidase from *R. gnavus* E1. After P6 purification, **26** was obtained as a white solid (0.6 mg, quant).

In PBS D_2O buffer (40 mM, pH 6.5) well resolved NMR spectra were obtained.

^1H (600 MHz, D_2O): δ (ppm)

	H-1	H-2	H-3	H-4	H-5	H-6	H-7	H-8	H-9	NHAc
GlcNAc	4.50 (d, J = 7.5 Hz, 1H)	3.71	3.70	3.69	3.58	3.98, 3.82	-	-	-	2.09 – 1.96 (m, 12H)
Galactose	4.46 (d, *J* = 7.6 Hz, 1H)	3.59	3.71	4.20	n/a	3.76 (2H)	-	-	-	-

GlcNAc-6S-1	4.72	3.83	n/a	n/a	3.82	4.41, 4.33	-	-	-	2.09 – 1.96 (m, 12H)
Galactose-6S-1	4.53 (d, J = 7.7 Hz, 1H)	3.58	3.75	4.24	n/a	4.18 (4H)	-	-	-	-
GlcNAc-6S -2	4.71	3.83	n/a	n/a	3.82	4.41, 4.29	-	-	-	2.09 – 1.96 (m, 12H)
Galactose-6S-2	4.63 (d, *J* = 7.8 Hz, 1H)	3.56	4.15	4.03	3.99	4.18 (4H)	-	-	-	-
Sialic acid	-	-	2.75 (d, J = 12.3 Hz, 1H), 1.82 (t, J = 12.0 Hz, 1H)	3.70	3.86	n/a	n/a	3.91	3.90, 3.65	2.09 – 1.96 (m, 12H)

^{13}C (150 MHz, D_2O): δ (ppm)

	C-1	C-2	C-3	C-4	C-5	C-6	C-7	C-8	C-9	NHAc
GlcNAc	100.94	55.05	72.49	78.59	74.93	60.14	-	-	-	22.21
Galactose	102.54	69.56	82.73	69.26	n/a	61.36	-	-	-	-
GlcNAc-6S-1	102.76	55.11	n/a	n/a	72.37	66.68	-	-	-	22.21
Galactose-6S-1	102.66	69.44	82.55	68.58	n/a	67.02	-	-	-	-
GlcNAc-6S 6S-2	102.76	55.11	n/a	n/a	72.37	66.85	-	-	-	22.21
Galactose-6S-2	102.54	69.44	75.08	67.38	72.91	67.02	-	-	-	-
Sialic acid	n/a	n/a	39.82	n/a	51.86	n/a	n/a	71.92	62.47	22.21

Linker	1	2	3	4	5	6
H	3.87,3.57	1.58 – 1.51 (m, 2H)	1.36 – 1.25 (m, 2H)	1.51 – 1.43 (m, 2H)	3.15 – 3.08 (m, 2H)	5.11 (s, 2H)
C	70.13	28.27	22.42	28.69	40.61	66.83

HRMS (ESI-MS): m/z calculated for $C_{66}H_{103}N_5O_{53}S_4$ $[M-2H]^{2-}$: 970.7206; found: 970.6880.

Compound 13

13 was prepared from **14** (1.8 mg, 1.0 µmol) using the general procedure for installation of α1,3Fuc using FUT6. After P6 purification, **13** was obtained as a white solid (2.0 mg, 92%). NMR data was confirmed with the previous made compound **13**.

Compound 28

28 was prepared from **27** (6.2 mg, 7.0 µmol) using the general procedure for installation of α2,6 Neu5Ac using PT2,6ST. After P6 purification, **28** was obtained as a white solid (7.9 mg, 96%).

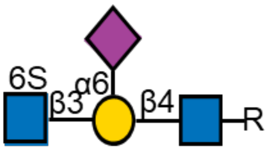

^{1}H (600 MHz, D_2O): δ (ppm)

	H-1	H-2	H-3	H-4	H-5	H-6	H-7	H-8	H-9	NHAc
GlcNAc	4.55 (d, J = 8.0 Hz, 1H)	3.72	n/a	n/a	3.58	3.99, 3.82	-	-	-	2.06 – 2.02 (m, 9H)
Galactose	4.44 (d, J = 7.9 Hz, 1H)	3.61	3.73	4.17 (d, J = 3.3 Hz, 1H)	n/a	4.00, 3.55	-	-	-	-
GlcNAc-6S	4.71 (d, J = 8.5 Hz, 1H)	3.78	3.58	3.54	3.66	4.32 (dd, J = 11.3, 2.1 Hz, 1H), 4.25 (dd, J = 11.3, 4.8 Hz, 1H)	-	-	-	2.06 – 2.02 (m, 9H)
Sialic acid	-	-	2.67 (dd, J = 12.4, 4.6 Hz, 1H), 1.72 (t, J = 12.1 Hz, 1H)	3.67	3.81	n/a	3.57	3.90	3.88, 3.65	2.06 – 2.02 (m, 9H)

^{13}C (150 MHz, D_2O): δ (ppm)

	C-1	C-2	C-3	C-4	C-5	C-6	C-7	C-8	C-9	NHAc
GlcNAc	100.86	54.96	n/a	n/a	74.59	60.65	-	-	-	22.16
Galactose	103.63	69.29	82.22	68.13	n/a	63.49	-	-	-	-
GlcNAc-6S	102.88	55.65	73.75	69.89	73.52	66.89	-	-	-	22.16
Sialic acid	n/a	n/a	40.12	n/a	51.89	n/a	68.70	71.94	62.90	22.16

Linker	1	2	3	4	5	6
H	3.87,3.58	1.61 – 1.52 (m, 2H)	1.37 – 1.24 (m, 2H)	1.50 (p, J = 7.2 Hz, 2H)	3.13 (t, J = 6.8 Hz, 2H)	5.12 (s, 2H)
C	69.84	28.25	22.48	28.44	40.50	66.73

HRMS (ESI-MS): m/z calculated for $C_{46}H_{71}N_4O_{29}S$ $[M-H]^-$: 1175.3930; found: 1175.3966.

Compound 29

29 was prepared from **28** (7.9 mg, 6.7 µmol) using the general procedure for installation of β1,4Gal using Hpβ4GalT. After P6 purification, **29** was obtained as a white solid (8.4 mg, 93%).

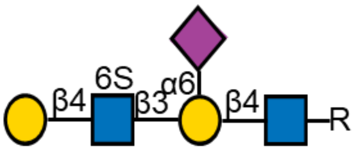

^1H (600 MHz, D_2O): δ (ppm)

	H-1	H-2	H-3	H-4	H-5	H-6	H-7	H-8	H-9	NHAc
GlcNAc	4.54	3.73	n/a	n/a	3.60	3.99, 3.82	-	-	-	2.06 – 2.01 (m, 9H)
Galactose-1	4.44 (d, J = 8.4 Hz, 1H)	3.61	3.73	4.17 (d, J = 3.3 Hz, 1H)	n/a	4.00, 3.56	-	-	-	-
GlcNAc-6S	4.73 (d, J = 8.4 Hz, 1H)	3.83	n/a	n/a	3.81	4.40 – 4.31 (m, 2H)	-	-	-	2.06 – 2.01 (m, 9H)
Galactose-2	4.55	3.55	3.69	3.94	n/a	3.79 – 3.74 (m, 2H)	-	-	-	-
Sialic acid	-	-	2.67 (dd, J = 12.4, 4.5 Hz, 1H),	3.68	3.81	n/a	n/a	3.91	3.88, 3.65	2.06 – 2.01 (m, 9H)

			1.73 (t, J = 12.1 Hz, 1H)							

^{13}C (150 MHz, D_2O): δ (ppm)

	C-1	C-2	C-3	C-4	C-5	C-6	C-7	C-8	C-9	NHAc
GlcNAc	101.01	54.93	n/a	n/a	74.31	60.58	-	-	-	22.22
Galactose-1	103.42	69.41	82.44	68.04	n/a	63.42	-	-	-	-
GlcNAc-6S	102.88	55.23	n/a	n/a	72.89	66.26	-	-	-	22.22
Galactose-2	102.68	71.41	72.47	68.54	n/a	61.10	-	-	-	-
Sialic acid	n/a	n/a	40.42	n/a	51.85	n/a	n/a	71.86	62.82	22.22

Linker	1	2	3	4	5	6
H	3.88,3.58	1.61 – 1.52 (m, 2H)	1.37 – 1.24 (m, 2H)	1.50 (p, J = 7.3 Hz, 2H)	3.13 (t, J = 6.8 Hz, 2H)	5.12 (s, 2H)
C	70.02	28.25	22.31	28.55	40.39	66.79

HRMS (ESI-MS): m/z calculated for $C_{52}H_{81}N_4O_{34}S$ $[M-H]^-$: 1337.4458; found: 1337.4225.

Compound 30

30 was prepared from **29** (8.4 mg, 6.2 µmol) using the general procedure for installation of β1,3GlcNAc using Hpβ3GlcNAcT. After P6 purification, **30** was obtained as a white solid (8.9 mg, 92%).

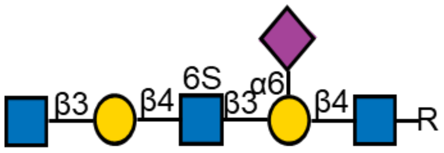

^1H (600 MHz, D_2O): δ (ppm)

	H-1	H-2	H-3	H-4	H-5	H-6	H-7	H-8	H-9	NHAc
GlcNAc-1	4.55	3.73	n/a	n/a	3.60	3.99, 3.82	-	-	-	2.07 – 2.01 (m, 12H)
Galactose-1	4.44 (d, J = 8.0 Hz, 1H)	3.61	3.73	4.17 (d, J = 3.3 Hz, 1H)	n/a	4.00, 3.55	-	-	-	-
GlcNAc-6S	4.73 (d, J = 8.4 Hz, 1H)	3.82	n/a	n/a	3.81	4.39 – 4.32 (m, 2H)	-	-	-	2.07 – 2.01 (m, 12H)

Galactose-2	4.54	3.58	3.75	4.16 (d, J = 3.0 Hz, 1H)	n/a	3.77 (2H)	-	-	-	-
GlcNAc-2	4.70 (d, J = 8.3 Hz, 1H)	3.77	n/a	n/a	3.47	3.91, 3.77	-	-	-	2.07 – 2.01 (m, 12H)
Sialic acid	-	-	2.67 (dd, J = 12.5, 4.5 Hz, 1H), 1.72 (t, J = 12.2 Hz, 1H)	3.67	3.81	n/a	n/a	3.91	3.88, 3.65	2.07 – 2.01 (m, 12H)

^{13}C (150 MHz, D_2O): δ (ppm)

	C-1	C-2	C-3	C-4	C-5	C-6	C-7	C-8	C-9	NHAc
GlcNAc-1	100.78	54.98	n/a	n/a	74.31	60.51	-	-	-	22.17
Galactose-1	103.42	69.93	82.44	68.04	n/a	63.42	-	-	-	-
GlcNAc-6S	102.68	55.18	n/a	n/a	72.54	66.38	-	-	-	22.17
Galactose-2	102.15	69.61	82.20	68.68	n/a	61.02	-	-	-	-
GlcNAc-2	102.96	55.60	n/a	n/a	n/a	60.56	-	-	-	22.17
Sialic acid	n/a	n/a	40.02	n/a	51.85	n/a	n/a	71.86	62.73	22.17

Linker	1	2	3	4	5	6
H	3.88,3.58	1.61 – 1.52 (m, 2H)	1.37 – 1.24 (m, 2H)	1.50 (p, J = 7.3 Hz, 2H)	3.13 (t, J = 6.8 Hz, 2H)	5.12 (s, 2H)
C	70.02	28.25	22.31	28.55	40.39	66.79

HRMS (ESI-MS): m/z calculated for $C_{60}H_{94}N_5O_{39}S$ $[M-H]^-$: 1540.5252; found: 1540.5051.

Compound 31

31 was prepared from **30** (8.9 mg, 5.8 μmol) using the general procedure for 6-O-sulfate installation of terminal GlcNAc using CHST2. After P6 purification, **31** was obtained as a white solid (6.7 mg, 72%).

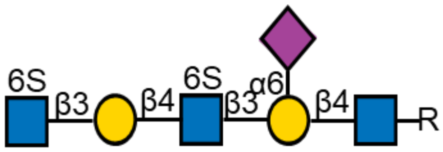

^1H (600 MHz, D_2O): δ (ppm)

	H-1	H-2	H-3	H-4	H-5	H-6	H-7	H-8	H-9	NHAc
GlcNAc	4.55	3.73	n/a	n/a	3.60	3.99, 3.82	-	-	-	2.07 – 2.01 (m, 12H)
Galactose-1	4.44 (d, J = 7.8 Hz, 1H)	3.61	3.73	4.17 (d, J = 3.2 Hz, 1H)	n/a	4.00, 3.55	-	-	-	-
GlcNAc-6S-1	4.73 (d, J = 8.3 Hz, 1H)	3.83	n/a	n/a	3.83	4.38 (d, J = 10.9 Hz, 1H), 4.33	-	-	-	2.07 – 2.01 (m, 12H)
Galactose-2	4.54	3.56	3.74	4.19 (d, J = 3.3 Hz, 1H)	n/a	3.76 (2H)	-	-	-	-
GlcNAc-6S-2	4.70 (d, J = 8.5 Hz, 1H)	3.81	3.59	3.53	3.67	4.33, 4.25 (dd, J = 11.2, 5.0 Hz, 1H)	-	-	-	2.07 – 2.01 (m, 12H)
Sialic acid	-	-	2.67 (dd, J = 12.4, 4.6 Hz, 1H), 1.72 (t, J = 12.2 Hz, 1H)	3.67	3.81	n/a	n/a	3.91	3.88, 3.65	2.07 – 2.01 (m, 12H)

^{13}C (150 MHz, D_2O): δ (ppm)

	C-1	C-2	C-3	C-4	C-5	C-6	C-7	C-8	C-9	NHAc
GlcNAc	100.74	54.90	n/a	n/a	74.44	60.51	-	-	-	22.17
Galactose-1	103.51	69.53	82.57	68.04	n/a	63.42	-	-	-	-
GlcNAc-6S-1	102.85	55.32	n/a	n/a	72.61	66.29	-	-	-	22.17
Galactose-2	102.51	69.20	82.65	68.29	n/a	61.13	-	-	-	-
GlcNAc-6S-2	102.99	55.82	73.86	69.55	73.51	66.94	-	-	-	22.17
Sialic acid	n/a	n/a	40.09	n/a	51.76	n/a	n/a	71.86	62.77	22.17

Linker	1	2	3	4	5	6
H	3.88,3.58	1.61 – 1.52 (m, 2H)	1.37 – 1.24 (m, 2H)	1.50 (p, J = 7.3 Hz, 2H)	3.13 (t, J = 6.8 Hz, 2H)	5.12 (s, 2H)
C	70.02	28.25	22.31	28.55	40.39	66.79

HRMS (ESI-MS): m/z calculated for $C_{60}H_{93}N_5O_{42}S_2$ $[M-2H]^{2-}$: 809.7374; found: 809.7224.

Compound 32

32 was prepared from **31** (5.0 mg, 3.1 µmol) using the general procedure for installation of α1,3Fuc using FUT6. After P6 purification, **32** was obtained as a white solid (5.4 mg, quant).

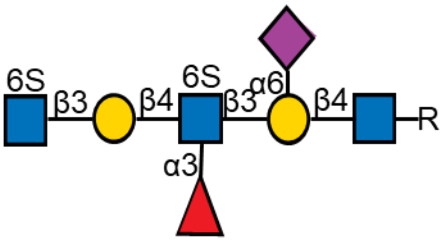

^{1}H (600 MHz, D_2O): δ (ppm)

	H-1	H-2	H-3	H-4	H-5	H-6	H-7	H-8	H-9	NHAc
GlcNAc	4.54	3.73	n/a	n/a	3.60	3.98, 3.81	-	-	-	2.07 – 2.01 (m, 12H)
Galactose-1	4.43 (d, J = 8.0 Hz, 1H)	3.61	3.72	4.17 (d, J = 3.3 Hz, 1H)	n/a	4.00, 3.55	-	-	-	-
GlcNAc-6S-1	4.75	3.97	3.90	4.01	3.80	4.37 – 4.34 (m, 2H)	-	-	-	2.07 – 2.01 (m, 12H)
Galactose-2	4.53	3.52	3.72	4.14 (d, J = 3.3 Hz, 1H)	n/a	3.73 (2H)	-	-	-	-
GlcNAc-6S-2	4.69 (d, J = 8.5 Hz, 1H)	3.79	3.57	3.53	3.66	4.33 – 4.30 (m, 1H), 4.26 (dd, J = 11.1, 4.6 Hz, 1H)	-	-	-	2.07 – 2.01 (m, 12H)
Fucose	5.12	3.68	n/a	n/a	4.83	1.16 (d, J = 6.5 Hz, 3H)	-	-	-	-
Sialic acid	-	-	2.66 (dd, J = 12.4, 4.6 Hz, 1H), 1.74 (t, J = 12.2 Hz, 1H)	3.67	3.81	n/a	n/a	3.91	3.88, 3.65	2.07 – 2.01 (m, 12H)

^{13}C (150 MHz, D_2O): δ (ppm)

	C-1	C-2	C-3	C-4	C-5	C-6	C-7	C-8	C-9	NHAc
GlcNAc	100.82	54.90	n/a	n/a	74.54	60.51	-	-	-	22.17
Galactose-1	103.55	69.38	82.58	68.21	n/a	63.42	-	-	-	-

GlcNAc-6S-1	102.48	55.83	n/a	72.72	72.61	66.09	-	-	-	22.17
Galactose-2	101.70	70.10	82.65	68.50	n/a	61.56	-	-	-	-
GlcNAc-6S-2	102.99	55.60	73.64	69.63	73.46	66.83	-	-	-	22.17
Fucose	98.46	n/a	n/a	n/a	66.73	15.29	-	-	-	-
Sialic acid	n/a	n/a	40.02	n/a	51.76	n/a	n/a	71.70	62.66	22.17

Linker	1	2	3	4	5	6
H	3.88,3.58	1.61 – 1.52 (m, 2H)	1.37 – 1.24 (m, 2H)	1.50 (p, J = 7.3 Hz, 2H)	3.13 (t, J = 6.8 Hz, 2H)	5.12 (s, 2H)
C	70.02	28.25	22.31	28.55	40.39	66.79

HRMS (ESI-MS): m/z calculated for $C_{66}H_{103}N_5O_{46}S_2$ $[M-2H]^{2-}$: 882.7663; found: 882.7471.

Compound 33

33 was prepared from **32** (5.4 mg, 3.1 µmol) using the general procedure for installation of β1,4Gal using B4GalT4. After P6 purification, **33** was obtained as a white solid (5.6 mg, 94%).

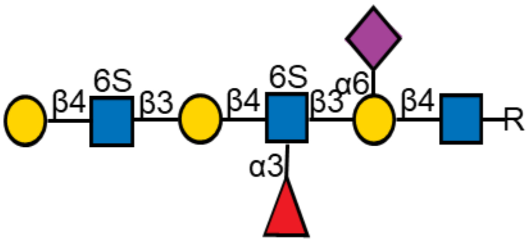

^{1}H (600 MHz, D_2O): δ (ppm)

	H-1	H-2	H-3	H-4	H-5	H-6	H-7	H-8	H-9	NHAc
GlcNAc	4.54	3.73	n/a	n/a	3.60	3.98, 3.81	-	-	-	2.07 – 2.01 (m, 12H)
Galactose-1	4.43 (d, J = 7.9 Hz, 1H)	3.60	3.72	4.18 – 4.16 (m, 1H)	n/a	4.00, 3.55	-	-	-	-
GlcNAc-6S-1	4.75	3.97	3.90	4.01	3.80	4.36 (4H)	-	-	-	2.07 – 2.01 (m, 12H)
Galactose-2	4.53	3.52	3.72	4.14 (d, J = 3.3 Hz, 1H)	n/a	3.73 (2H)	-	-	-	-
GlcNAc-6S-2	4.72 (d, J =	3.84	3.73	3.81	3.80	4.36 (4H)	-	-	-	2.07 – 2.01

	8.5 Hz, 1H)									(m, 12H)
Galactose-3	4.55	3.55	3.69	3.93 (d, J = 3.3 Hz, 1H)	n/a	3.77 (2H)	-	-	-	-
Fucose	5.12	3.68	n/a	n/a	4.83	1.16 (d, J = 6.5 Hz, 3H)	-	-	-	-
Sialic acid	-	-	2.67 (dd, J = 12.5, 4.5 Hz, 1H), 1.72 (t, J = 12.1 Hz, 1H)	3.68	3.81	n/a	n/a	3.90	3.88, 3.65	2.07 – 2.01 (m, 12H)

^{13}C (150 MHz, D_2O): δ (ppm)

	C-1	C-2	C-3	C-4	C-5	C-6	C-7	C-8	C-9	NHAc
GlcNAc	100.82	54.90	n/a	n/a	74.68	60.72	-	-	-	22.17
Galactose-1	103.49	69.14	82.60	67.99	n/a	63.46	-	-	-	-
GlcNAc-6S-1	102.54	55.83	n/a	72.88	72.71	66.79	-	-	-	22.17
Galactose-2	101.70	70.66	82.60	68.21	n/a	61.48	-	-	-	-
GlcNAc-6S-2	102.88	55.17	72.79	77.12	72.71	66.79	-	-	-	22.17
Galactose-3	102.60	71.62	72.80	68.78	n/a	61.00	-	-	-	-
Fucose	98.57	n/a	n/a	n/a	66.73	15.35	-	-	-	-
Sialic acid	n/a	n/a	40.09	n/a	51.97	n/a	n/a	71.70	62.88	22.17

Linker	1	2	3	4	5	6
H	3.88,3.58	1.61 – 1.52 (m, 2H)	1.37 – 1.24 (m, 2H)	1.50 (p, J = 7.3 Hz, 2H)	3.13 (t, J = 6.8 Hz, 2H)	5.12 (s, 2H)
C	70.02	28.25	22.31	28.55	40.39	66.79

HRMS (ESI-MS): m/z calculated for $C_{72}H_{113}N_5O_{51}S_2$ $[M-2H]^{2-}$: 963.7927; found: 963.7632.

Compound 34

34 was prepared from **33** (5.6 mg, 2.9 µmol) using the general procedure for installation of β1,3GlcNAc using B3GnT2. After P6 purification, **34** was obtained as a white solid (5.5 mg, 89%).

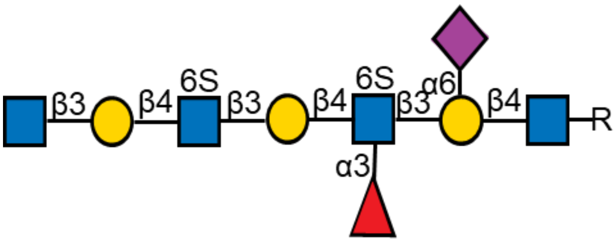

^{1}H (600 MHz, D_2O): δ (ppm)

	H-1	H-2	H-3	H-4	H-5	H-6	H-7	H-8	H-9	NHAc
GlcNAc-1	4.54	3.73	n/a	n/a	3.60	3.98, 3.82	-	-	-	2.08 – 1.98 (m, 15H)
Galactose-1	4.43 (d, J = 7.9 Hz, 1H)	3.60	3.72	4.17 (d, J = 3.3 Hz, 1H)	n/a	4.00, 3.55	-	-	-	-
GlcNAc-6S-1	4.75	3.97	3.90	4.01	3.80	4.36 (4H)	-	-	-	2.08 – 1.98 (m, 15H)
Galactose-2	4.53	3.52	3.73	4.14 (d, J = 3.3 Hz, 1H)	n/a	3.73 (2H)	-	-	-	-
GlcNAc-6S-2	4.72	3.84	3.73	3.81	3.80	4.36 (4H)	-	-	-	2.08 – 1.98 (m, 15H)
Galactose-3	4.54	3.60	3.74	4.15 (d, J = 3.1 Hz, 1H)	n/a	3.77 (2H)	-	-	-	-
GlcNAc-2	4.70	3.78	n/a	n/a	3.46	3.90, 3.78	-	-	-	2.08 – 1.98 (m, 15H)
Fucose	5.12	3.68	n/a	n/a	4.83	1.16 (d, J = 6.5 Hz, 3H)	-	-	-	-
Sialic acid	-	-	2.67 (dd, J = 12.4, 4.6 Hz, 1H), 1.72 (t, J = 12.2 Hz, 1H)	3.68	3.81	n/a	n/a	3.91	3.88, 3.65	2.08 – 1.98 (m, 15H)

^{13}C (150 MHz, D_2O): δ (ppm)

	C-1	C-2	C-3	C-4	C-5	C-6	C-7	C-8	C-9	NHAc
GlcNAc-1	100.82	54.94	n/a	n/a	74.47	60.48	-	-	-	22.17
Galactose-1	103.43	69.17	82.67	67.99	n/a	63.46	-	-	-	-
GlcNAc-6S-1	102.54	55.83	n/a	72.51	72.71	66.11	-	-	-	22.17
Galactose-2	101.70	70.34	82.60	68.28	n/a	61.48	-	-	-	-
GlcNAc-6S-2	102.88	55.17	72.72	77.15	72.71	66.11	-	-	-	22.17
Galactose-3	102.28	70.01	82.09	68.36	n/a	61.00	-	-	-	-
GlcNAc-2	102.88	55.79	n/a	n/a	n/a	60.62	-	-	-	22.17
Fucose	98.44	n/a	n/a	n/a	66.73	15.29	-	-	-	-
Sialic acid	n/a	n/a	40.18	n/a	51.79	n/a	n/a	71.75	62.64	22.17

Linker	1	2	3	4	5	6
H	3.88,3.58	1.61 – 1.52 (m, 2H)	1.37 – 1.24 (m, 2H)	1.50 (p, J = 7.3 Hz, 2H)	3.13 (t, J = 6.8 Hz, 2H)	5.12 (s, 2H)
C	70.02	28.25	22.31	28.55	40.39	66.79

HRMS (ESI-MS): m/z calculated for $C_{80}H_{126}N_6O_{56}S_2$ $[M-2H]^{2-}$: 1065.3324; found: 1065.2949.

Compound 35

35 was prepared from **34** (5.5 mg, 2.6 μmol) using the general procedure for 6-O-sulfate installation of terminal GlcNAc using CHST2. After P6 purification, **35** was obtained as a white solid (5.6 mg, 98%).

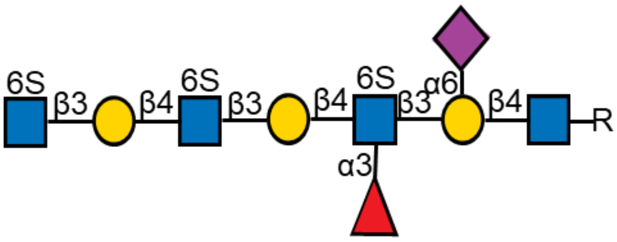

^{1}H (600 MHz, D_2O): δ (ppm)

	H-1	H-2	H-3	H-4	H-5	H-6	H-7	H-8	H-9	NHAc
GlcNAc	4.55	3.73	n/a	n/a	3.60	3.98, 3.82	-	-	-	2.08 – 1.98 (m, 15H)
Galactose-1	4.43 (d, J = 7.8 Hz, 1H)	3.60	3.72	4.17	n/a	4.00, 3.55	-	-	-	-

GlcNAc-6S-1	4.75	3.98	3.90	4.01	3.80	4.35 (4H)	-	-	-	2.08 – 1.98 (m, 15H)
Galactose-2	4.54	3.52	3.72	4.14	n/a	3.73 (2H)	-	-	-	-
GlcNAc-6S-2	4.72	3.83	3.73	3.80	3.80	4.35 (4H)	-	-	-	2.08 – 1.98 (m, 15H)
Galactose-3	4.53	3.59	3.74	4.19	n/a	3.76 (2H)	-	-	-	-
GlcNAc-6S-3	4.70	3.80	3.59	3.53	3.67	4.33 (d, *J* = 11.4 Hz, 1H), 4.27 – 4.23 (m, 1H)	-	-	-	2.08 – 1.98 (m, 15H)
Fucose	5.12	3.68	n/a	n/a	4.83	1.16 (d, J = 6.4 Hz, 3H)	-	-	-	-
Sialic acid	-	-	2.67 (dd, J = 12.8, 3.8 Hz, 1H), 1.72 (t, J = 12.1 Hz, 1H)	3.68	3.81	n/a	n/a	3.91	3.88, 3.65	2.08 – 1.98 (m, 15H)

^{13}C (150 MHz, D_2O): δ (ppm)

	C-1	C-2	C-3	C-4	C-5	C-6	C-7	C-8	C-9	NHAc
GlcNAc	100.82	54.94	n/a	n/a	74.47	60.48	-	-	-	22.17
Galactose-1	103.43	69.72	82.59	68.11	n/a	63.46	-	-	-	-
GlcNAc-6S-1	102.64	55.83	n/a	72.60	72.71	66.19	-	-	-	22.17
Galactose-2	101.70	70.05	82.59	68.36	n/a	61.48	-	-	-	-
GlcNAc-6S-2	102.69	55.17	72.72	77.52	72.71	66.19	-	-	-	22.17
Galactose-3	102.28	69.55	82.59	68.78	n/a	61.17	-	-	-	-
GlcNAc-6S-3	102.86	55.62	73.37	69.55	73.53	66.84	-	-	-	22.17
Fucose	98.51	n/a	n/a	n/a	66.73	15.29	-	-	-	-
Sialic acid	n/a	n/a	40.18	n/a	51.76	n/a	n/a	71.75	62.64	22.17

Linker	1	2	3	4	5	6
H	3.88,3.58	1.61 – 1.52 (m, 2H)	1.37 – 1.24 (m, 2H)	1.50 (p, J = 7.3 Hz, 2H)	3.13 (t, J = 6.8 Hz, 2H)	5.12 (s, 2H)
C	70.02	28.25	22.31	28.55	40.39	66.79

HRMS (ESI-MS): m/z calculated for $C_{80}H_{125}N_6O_{59}S_3$ $[M-3H]^{3-}$: 736.5381; found: 736.5203.

Compound 36

36 was prepared from **35** (5.6 mg, 2.5 µmol) using the general procedure for installation of α2,6Neu5Ac using PT2,6ST. After P6 purification, **36** was obtained as a white solid (5.7 mg, 90%).

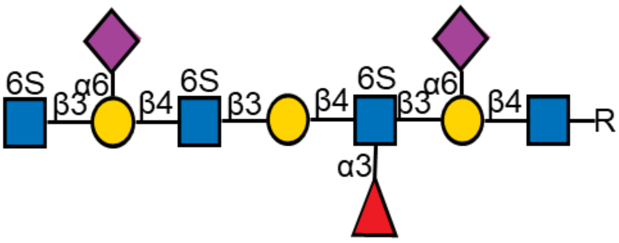

^{1}H (600 MHz, D_2O): δ (ppm)

	H-1	H-2	H-3	H-4	H-5	H-6	H-7	H-8	H-9	NHAc
GlcNAc	4.55	3.73	n/a	n/a	3.60	3.98, 3.81	-	-	-	2.08 – 1.98 (m, 18H)
Galactose-1	4.44 (d, J = 7.9 Hz, 1H)	3.59	3.73	4.17	n/a	4.00, 3.55	-	-	-	-
GlcNAc-6S-1	4.75	3.97	3.90	4.01	3.80	4.35 (2H)	-	-	-	2.08 – 1.98 (m, 18H)
Galactose-2	4.54	3.52	3.73	4.15	n/a	3.73 (2H)	-	-	-	-
GlcNAc-6S-2	4.73	3.84	n/a	n/a	3.83	4.39 (d, J = 10.5 Hz, 1H), 4.32	-	-	-	2.08 – 1.98 (m, 18H)
Galactose-3	4.48 (d, J = 8.0 Hz, 1H)	3.59	3.74	4.16	n/a	4.00, 3.55	-	-	-	-
GlcNAc-6S-3	4.71 (d, J = 7.7 Hz, 1H)	3.79	3.58	3.53	3.66	4.31, 4.27 (dd, J = 11.3, 4.3 Hz, 1H)	-	-	-	2.08 – 1.98 (m, 18H)
Fucose	5.12	3.68	n/a	n/a	4.83	1.17 (3H)	-	-	-	-
Sialic acid-1	-	-	2.67, 1.72	3.68	3.81	n/a	n/a	3.91	3.88, 3.65	2.08 – 1.98 (m, 18H)
Sialic acid-2	-	-	2.67, 1.72	3.68	3.81	n/a	n/a	3.91	3.88, 3.65	2.08 – 1.98 (m, 18H)

^{13}C (150 MHz, D_2O): δ (ppm)

	C-1	C-2	C-3	C-4	C-5	C-6	C-7	C-8	C-9	NHAc
GlcNAc	100.82	54.94	n/a	n/a	74.47	60.48	-	-	-	22.17
Galactose-1	103.49	69.92	82.44	68.18	n/a	63.53	-	-	-	-
GlcNAc-6S-1	102.42	55.95	n/a	72.38	72.71	66.19	-	-	-	22.17
Galactose-2	101.50	70.15	82.44	68.18	n/a	61.63	-	-	-	-
GlcNAc-6S-2	102.69	54.92	n/a	n/a	72.71	66.57	-	-	-	22.17
Galactose-3	103.49	69.92	82.44	68.18	n/a	63.53	-	-	-	-
GlcNAc-6S-3	103.34	55.69	73.88	69.63	73.71	66.74	-	-	-	22.17
Fucose	98.54	n/a	n/a	n/a	66.73	15.58	-	-	-	-
Sialic acid-1	n/a	n/a	40.05	n/a	51.76	n/a	n/a	71.59	62.74	22.17
Sialic acid-2	n/a	n/a	40.05	n/a	51.76	n/a	n/a	71.59	62.74	22.17

Linker	1	2	3	4	5	6
H	3.88,3.58	1.61 – 1.52 (m, 2H)	1.37 – 1.24 (m, 2H)	1.50 (p, J = 7.3 Hz, 2H)	3.13 (t, J = 6.8 Hz, 2H)	5.12 (s, 2H)
C	70.02	28.25	22.31	28.55	40.39	66.79

HRMS (ESI-MS): m/z calculated for $C_{91}H_{142}N_7O_{67}S_3$ $[M-3H]^{3-}$: 833.5699; found: 833.5462.

Compound 37

37 was prepared from **36** (5.7 mg, 2.3 µmol) using the general procedure for installation of β1,4Gal using Hpβ4GalT. After P6 purification, **37** was obtained as a white solid (6.0 mg, quant).

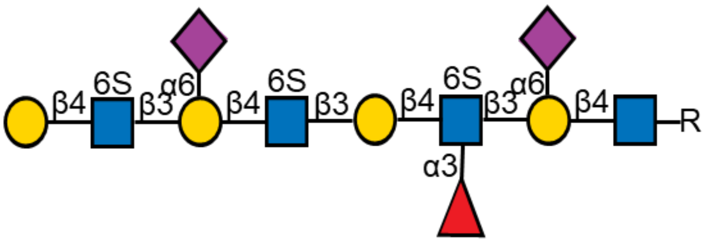

^{1}H (600 MHz, D_2O): δ (ppm)

	H-1	H-2	H-3	H-4	H-5	H-6	H-7	H-8	H-9	NHAc
GlcNAc	4.55	3.73	n/a	n/a	3.60	3.98, 3.81	-	-	-	2.08 – 1.98 (m, 18H)

Galactose-1	4.44 (d, J = 7.9 Hz, 1H)	3.59	3.72	4.17	n/a	4.00, 3.55	-	-	-	-
GlcNAc-6S-1	4.75	3.97	3.90	4.01	3.80	4.36 (4H)	-	-	-	2.08 – 1.98 (m, 18H)
Galactose-2	4.53	3.52	3.73	4.15	n/a	3.73 (2H)	-	-	-	-
GlcNAc-6S-2	4.74	3.83	n/a	n/a	3.84	4.39 (d, J = 10.5 Hz, 1H), 4.35 – 4.29 (m, 1H)	-	-	-	2.08 – 1.98 (m, 18H)
Galactose-3	4.48 (d, J = 7.8 Hz, 1H)	3.59	3.74	4.16	n/a	4.00, 3.55	-	-	-	-
GlcNAc-6S-3	4.74	3.83	n/a	n/a	3.80	4.36 (4H)	-	-	-	2.08 – 1.98 (m, 18H)
Galactose-4	4.56	3.53	3.69	3.93	n/a	3.76 (2H)	-	-	-	-
Fucose	5.12	3.68	n/a	n/a	4.82	1.17 (3H)	-	-	-	-
Sialic acid-1	-	-	2.67, 1.72	3.68	3.81	n/a	n/a	3.91	3.88, 3.65	2.08 – 1.98 (m, 18H)
Sialic acid-2	-	-	2.67, 1.72	3.68	3.81	n/a	n/a	3.91	3.88, 3.65	2.08 – 1.98 (m, 18H)

^{13}C (150 MHz, D_2O): δ (ppm)

	C-1	C-2	C-3	C-4	C-5	C-6	C-7	C-8	C-9	NHAc
GlcNAc	100.82	54.94	n/a	n/a	74.69	60.48	-	-	-	22.17
Galactose-1	103.49	69.48	82.44	68.32	n/a	63.53	-	-	-	-
GlcNAc-6S-1	102.42	55.71	n/a	72.53	72.71	66.15	-	-	-	22.17
Galactose-2	101.50	70.15	82.44	68.32	n/a	61.63	-	-	-	-
GlcNAc-6S-2	102.69	55.02	n/a	n/a	72.84	66.57	-	-	-	22.17
Galactose-3	103.49	69.48	82.44	68.32	n/a	63.53	-	-	-	-
GlcNAc-6S-3	102.69	55.02	n/a	n/a	72.71	66.15	-	-	-	22.17
Galactose-4	102.33	71.29	72.61	68.91	n/a	61.22	-	-	-	-
Fucose	98.48	n/a	n/a	n/a	66.73	15.74	-	-	-	-
Sialic acid-1	n/a	n/a	40.12	n/a	51.84	n/a	n/a	71.59	62.74	22.17
Sialic acid-2	n/a	n/a	40.12	n/a	51.84	n/a	n/a	71.59	62.74	22.17

Linker	1	2	3	4	5	6
H	3.88,3.58	1.61 – 1.52 (m, 2H)	1.37 – 1.24 (m, 2H)	1.50 (p, J = 7.3 Hz, 2H)	3.13 (t, J = 6.8 Hz, 2H)	5.12 (s, 2H)
C	70.02	28.25	22.31	28.55	40.39	66.79

HRMS (ESI-MS): m/z calculated for $C_{97}H_{152}N_7O_{72}S_3$ $[M-3H]^{3-}$: 887.9220; found: 887.8949.

Compound 38

38 was prepared from **37** (6.0 mg, 2.3 µmol) using the general procedure for installation of α2,3Neu5Ac using PmST1 M144D. After P6 purification, **38** was obtained as a white solid (6.0 mg, 91%).

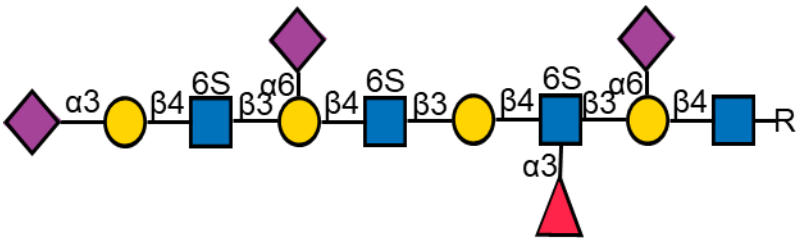

^{1}H (600 MHz, D_2O): δ (ppm)

	H-1	H-2	H-3	H-4	H-5	H-6	H-7	H-8	H-9	NHAc
GlcNAc	4.55	3.73	n/a	n/a	3.60	3.98, 3.81	-	-	-	2.08 – 1.98 (m, 21H)
Galactose-1	4.44 (d, J = 7.7 Hz, 1H)	3.59	3.73	4.17	n/a	4.00, 3.55	-	-	-	-
GlcNAc-6S-1	4.75	3.97	3.90	4.01	3.81	4.36 (4H)	-	-	-	2.08 – 1.98 (m, 21H)
Galactose-2	4.54	3.52	3.73	4.15	n/a	3.73 (2H)	-	-	-	-
GlcNAc-6S-2	4.73	3.83	n/a	n/a	3.85	4.39 (d, J = 10.3 Hz, 1H), 4.33 – 4.28 (m, 1H)	-	-	-	2.08 – 1.98 (m, 21H)
Galactose-3	4.47 (d, J = 7.3 Hz, 1H)	3.59	3.74	4.16	n/a	4.00, 3.55	-	-	-	-
GlcNAc-6S-3	4.73	3.83	n/a	n/a	3.81	4.36 (4H)	-	-	-	2.08 – 1.98

										(m, 21H)
Galactose-4	4.63 (d, J = 7.5 Hz, 1H)	3.55	4.13	3.98	n/a	3.74 (2H)	-	-	-	-
Fucose	5.12	3.68	n/a	n/a	4.82	1.17 (3H)	-	-	-	-
Sialic acid-1	-	-	2.67, 1.71	3.68	3.81	n/a	n/a	3.91	3.88, 3.65	2.08 – 1.98 (m, 21H)
Sialic acid-2	-	-	2.67, 1.71	3.68	3.81	n/a	n/a	3.91	3.88, 3.65	2.08 – 1.98 (m, 21H)
Sialic acid-3	-	-	2.76 (d, J = 11.8 Hz, 1H), 1.82 (t, J = 12.0 Hz, 1H)	3.67	3.87	n/a	n/a	3.91	3.88, 3.65	2.08 – 1.98 (m, 21H)

^{13}C (150 MHz, D_2O): δ (ppm)

	C-1	C-2	C-3	C-4	C-5	C-6	C-7	C-8	C-9	NHAc
GlcNAc	100.82	54.94	n/a	n/a	74.69	60.48	-	-	-	22.17
Galactose-1	103.49	69.48	82.24	68.14	n/a	63.53	-	-	-	-
GlcNAc-6S-1	102.42	55.71	n/a	72.53	72.48	66.22	-	-	-	22.17
Galactose-2	101.50	70.15	82.44	68.14	n/a	61.63	-	-	-	-
GlcNAc-6S-2	102.69	55.02	n/a	n/a	72.91	66.55	-	-	-	22.17
Galactose-3	103.49	69.48	82.44	68.14	n/a	63.53	-	-	-	-
GlcNAc-6S-3	102.69	55.02	n/a	n/a	72.48	66.22	-	-	-	22.17
Galactose-4	102.20	69.60	75.41	67.46	n/a	60.93	-	-	-	-
Fucose	98.48	n/a	n/a	n/a	66.65	15.74	-	-	-	-
Sialic acid-1	n/a	n/a	40.12	n/a	51.84	n/a	n/a	71.59	62.74	22.17
Sialic acid-2	n/a	n/a	40.12	n/a	51.84	n/a	n/a	71.59	62.74	22.17
Sialic acid-3	n/a	n/a	39.64	n/a	51.96	n/a	n/a	71.59	62.74	22.17

Linker	1	2	3	4	5	6
H	3.88,3.58	1.61 – 1.52 (m, 2H)	1.37 – 1.24 (m, 2H)	1.50 (p, J = 7.3 Hz, 2H)	3.13 (t, J = 6.8 Hz, 2H)	5.12 (s, 2H)
C	70.02	28.25	22.31	28.55	40.39	66.79

HRMS (ESI-MS): m/z calculated for $C_{108}H_{170}N_8O_{80}S_3$ $[M-3H]^{3-}$: 985.2897; found: 985.2680.

Compound 39

39 was prepared from **38** (6.0 mg, 2.0 μmol) using the general procedure for 6-O-sulfate installation of internal Galactose using CHST1. After P6 purification, **39** was obtained as a white solid (5.1 mg, 82%).

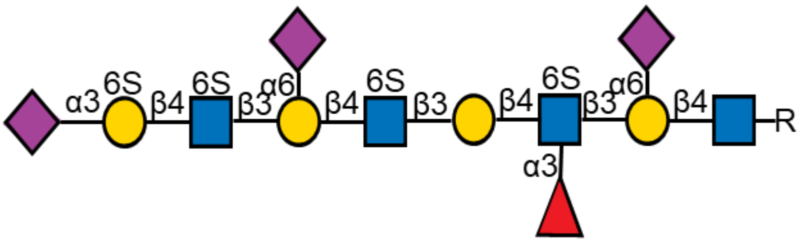

^{1}H (600 MHz, D_2O): δ (ppm)

	H-1	H-2	H-3	H-4	H-5	H-6	H-7	H-8	H-9	NHAc
GlcNAc	4.55	3.73	n/a	n/a	3.60	3.98, 3.81	-	-	-	2.08 – 2.01 (m, 21H)
Galactose-1	4.44 (d, J = 7.2 Hz, 1H)	3.60	3.73	4.17	n/a	4.00, 3.55	-	-	-	-
GlcNAc-6S-1	4.75	3.97	3.90	4.01	3.81	4.35 (4H)	-	-	-	2.08 – 2.01 (m, 21H)
Galactose-2	4.54	3.54	3.73	4.16	n/a	3.73 (2H)	-	-	-	-
GlcNAc-6S-2	4.73	3.83	n/a	n/a	3.85	4.40 (d, J = 10.4 Hz, 1H), 4.33 – 4.28 (m, 1H)	-	-	-	2.08 – 2.01 (m, 21H)
Galactose-3	4.48 (d, J = 7.8 Hz, 1H)	3.60	3.74	4.17	n/a	4.00, 3.55	-	-	-	-
GlcNAc-6S-3	4.72	3.83	n/a	n/a	3.81	4.35 (4H)	-	-	-	2.08 – 2.01 (m, 21H)
Galactose-6S	4.64 (d, J = 7.9 Hz, 1H)	3.57	4.16	4.03	3.99	4.18 (2H)	-	-	-	-
Fucose	5.12	3.68	n/a	n/a	4.82	1.17 (d, J = 6.4 Hz, 3H)	-	-	-	-
Sialic acid-1	-	-	2.67, 1.73	3.68	3.81	n/a	n/a	3.91	3.89, 3.65	2.08 – 2.01 (m, 21H)

Sialic acid-2	-	-	2.67, 1.73	3.68	3.81	n/a	n/a	3.91	3.89, 3.65	2.08 – 2.01 (m, 21H)
Sialic acid-3	-	-	2.76, 1.82 (t, J = 12.1 Hz, 1H)	3.67	3.87	n/a	n/a	3.91	3.89, 3.65	2.08 – 2.01 (m, 21H)

^{13}C (150 MHz, D_2O): δ (ppm)

	C-1	C-2	C-3	C-4	C-5	C-6	C-7	C-8	C-9	NHAc
GlcNAc	100.82	54.94	n/a	n/a	74.69	60.48	-	-	-	22.17
Galactose-1	103.49	69.48	82.24	68.39	n/a	63.53	-	-	-	-
GlcNAc-6S-1	102.42	55.84	n/a	72.53	72.48	66.28	-	-	-	22.17
Galactose-2	101.50	69.59	82.44	68.39	n/a	61.71	-	-	-	-
GlcNAc-6S-2	102.69	55.02	n/a	n/a	72.91	66.62	-	-	-	22.17
Galactose-3	103.49	69.48	82.44	68.39	n/a	63.53	-	-	-	-
GlcNAc-6S-3	102.69	55.02	n/a	n/a	72.48	66.28	-	-	-	22.17
Galactose-6S	102.59	69.48	75.27	67.46	72.68	66.69	-	-	-	-
Fucose	98.48	n/a	n/a	n/a	66.68	15.37	-	-	-	-
Sialic acid-1	n/a	n/a	40.12	n/a	51.78	n/a	n/a	71.59	62.74	22.17
Sialic acid-2	n/a	n/a	40.12	n/a	51.78	n/a	n/a	71.59	62.74	22.17
Sialic acid-3	n/a	n/a	39.64	n/a	51.88	n/a	n/a	71.59	62.74	22.17

Linker	1	2	3	4	5	6
H	3.88,3.58	1.61 – 1.52 (m, 2H)	1.37 – 1.24 (m, 2H)	1.50 (p, J = 7.3 Hz, 2H)	3.13 (t, J = 6.8 Hz, 2H)	5.12 (s, 2H)
C	70.02	28.25	22.31	28.55	40.39	66.79

HRMS (ESI-MS): m/z calculated for $C_{108}H_{169}N_8O_{83}S_4$ $[M-4H]^{4-}$: 758.7047; found: 758.6606.

Compound S17

S17 was prepared from **39** (3.6 mg, 1.2 μmol) using the general procedure for the removal of sialic acid using *C. perfringens* neuraminidase. After P6 purification, **S17** was obtained as a white solid (2.5 mg, quant).

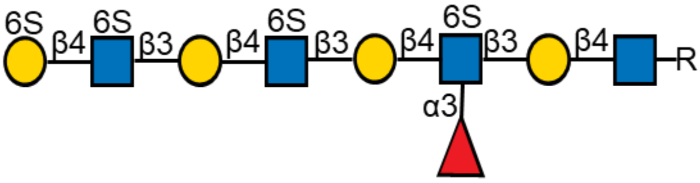

^{1}H (600 MHz, D_2O): δ (ppm)

	H-1	H-2	H-3	H-4	H-5	H-6	NHAc
GlcNAc	4.51	3.71	3.70	3.69	3.58	3.98, 3.82	2.08 – 1.98 (m, 12H)
Galactose-1	4.46 (d, J = 7.8 Hz, 1H)	3.59	3.72	4.19	n/a	3.76 (4H)	-
GlcNAc-6S-1	4.75	3.98	3.90	4.01	3.81	4.38, 4.33	2.08 – 1.98 (m, 12H)
Galactose-2	4.50	3.52	3.72	4.14 (d, J = 3.2 Hz, 1H)	n/a	3.73 (2H)	-
GlcNAc-6S-2	4.72	3.83	3.77	3.78	3.85	4.38, 4.33	2.08 – 1.98 (m, 12H)
Galactose-3	4.53	3.59	3.73	4.19	n/a	3.76 (4H)	-
GlcNAc-6S-3	4.72	3.83	3.77	3.78	3.81	4.38, 4.33	2.08 – 1.98 (m, 12H)
Galactose-6S	4.55 (d, J = 8.2 Hz, 1H)	3.56	3.72	4.00	3.99	4.20 (2H)	-
Fucose	5.12	3.69	3.92	3.80	4.83	1.16 (d, J = 6.5 Hz, 3H)	-

^{13}C (150 MHz, D_2O): δ (ppm)

	C-1	C-2	C-3	C-4	C-5	C-6	NHAc
GlcNAc	100.74	54.94	72.66	78.70	74.89	60.11	22.17
Galactose-1	102.69	69.90	82.44	68.72	n/a	61.08	-
GlcNAc-6S-1	102.60	55.91	74.67	72.48	72.30	66.40	22.17
Galactose-2	102.78	70.50	82.44	68.36	n/a	61.49	-

GlcNAc-6S-2	102.74	55.02	n/a	n/a	72.75	66.40	22.17
Galactose-3	102.88	69.90	82.44	68.72	n/a	61.08	-
GlcNAc-6S-3	102.74	55.02	n/a	n/a	72.39	66.40	22.17
Galactose-6S	103.15	71.32	n/a	68.46	72.78	66.38	-
Fucose	98.54	n/a	69.04	n/a	66.84	15.33	-

Linker	1	2	3	4	5	6
H	3.88,3.58	1.61 – 1.52 (m, 2H)	1.37 – 1.24 (m, 2H)	1.50 (p, J = 7.3 Hz, 2H)	3.13 (t, J = 6.8 Hz, 2H)	5.12 (s, 2H)
C	70.02	28.25	22.31	28.55	40.39	66.79

HRMS (ESI-MS): m/z calculated for $C_{75}H_{118}N_5O_{59}S_4$ $[M-3H]^{3-}$: 720.1762; found: 720.1662.

Compound 40

40 was prepared from **S17** (1.8 mg, 0.8 µmol) using the general procedure for installation of α2,3Neu5Ac using PmST1 M144D. After P6 purification, **40** was obtained as a white solid (1.9 mg, 96%).

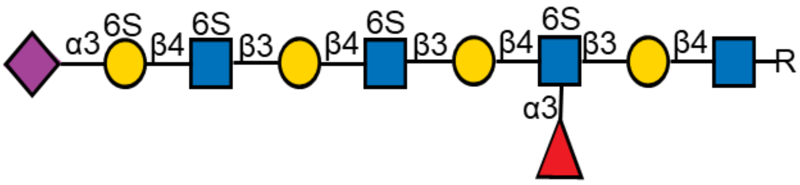

^{1}H (600 MHz, D_2O): δ (ppm)

	H-1	H-2	H-3	H-4	H-5	H-6	H-7	H-8	H-9	NHAc
GlcNAc	4.51	3.71	3.70	3.69	3.58	3.98, 3.81	-	-	-	2.07 – 1.96 (m, 15H)
Galactose-1	4.46 (d, J = 7.7 Hz, 1H)	3.59	3.72	4.19	n/a	3.76 (4H)	-	-	-	-
GlcNAc-6S-1	4.75	3.99	3.90	4.01	3.82	4.36 (2H)	-	-	-	2.07 – 1.96 (m, 15H)
Galactose-2	4.50	3.52	3.72	4.14	n/a	3.73 (2H)	-	-	-	-
GlcNAc-6S-2	4.71 (d, *J* = 7.8 Hz, 2H)	3.84	3.77	3.78	3.82	4.41, 4.32	-	-	-	2.07 – 1.96 (m, 15H)

Galactose-3	4.53	3.58	3.72	4.20	n/a	3.76 (4H)	-	-	-	-
GlcNAc-6S-3	4.71 (d, *J* = 7.8 Hz, 2H)	3.84	3.77	3.78	3.82	4.41, 4.32	-	-	-	2.07 – 1.96 (m, 15H)
Galactose-6S	4.63 (d, J = 7.8 Hz, 1H)	3.58	4.16	4.03	3.99	4.18 (2H)	-	-	-	-
Fucose	5.12	3.69	3.92	3.80	4.83	1.16 (d, J = 6.4 Hz, 3H)	-	-	-	-
Sialic acid	-	-	2.75 (d, J = 11.8 Hz, 1H), 1.82 (t, J = 12.0 Hz, 1H)	3.70	3.87	n/a	n/a	3.92	3.90, 3.65	2.07 – 1.96 (m, 15H)

^{13}C (150 MHz, D_2O): δ (ppm)

	C-1	C-2	C-3	C-4	C-5	C-6	C-7	C-8	C-9	NHAc
GlcNAc	100.74	55.06	72.66	78.70	74.89	60.21	-	-	-	22.17
Galactose-1	102.69	69.59	82.53	68.83	n/a	61.08	-	-	-	-
GlcNAc-6S-1	102.66	55.82	74.78	72.90	72.68	66.40	-	-	-	22.17
Galactose-2	102.11	70.20	82.53	68.42	n/a	61.49	-	-	-	-
GlcNAc-6S-2	102.82	55.02	n/a	n/a	72.68	66.48	-	-	-	22.17
Galactose-3	102.65	69.59	82.53	68.78	n/a	61.08	-	-	-	-
GlcNAc-6S-3	102.82	55.02	n/a	n/a	72.68	66.48	-	-	-	22.17
Galactose-6S	102.58	69.80	75.18	67.44	72.78	66.48	-	-	-	-
Fucose	98.51	n/a	69.04	n/a	66.73	15.33	-	-	-	-
Sialic acid	n/a	n/a	39.57	n/a	51.69	n/a	n/a	71.85	62.58	22.17

Linker	1	2	3	4	5	6
H	3.88,3.58	1.61 – 1.52 (m, 2H)	1.37 – 1.24 (m, 2H)	1.50 (p, J = 7.3 Hz, 2H)	3.13 (t, J = 6.8 Hz, 2H)	5.12 (s, 2H)
C	70.02	28.25	22.31	28.55	40.39	66.79

HRMS (ESI-MS): m/z calculated for $C_{86}H_{134}N_6O_{67}S_4$ $[M-4H]^{4-}$: 612.6542; found: 612.6484.

Compound 41

41 was prepared from **39** (1.5 mg, 0.5 μmol) using the general procedure for removal of fucose using fucosidase from *R. gnavus* E1. After P6 purification, **41** was obtained as a white solid (1.4 mg, quant).

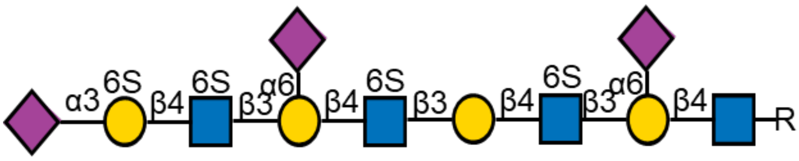

^{1}H (600 MHz, D_2O): δ (ppm)

	H-1	H-2	H-3	H-4	H-5	H-6	H-7	H-8	H-9	NHAc
GlcNAc	4.55	3.73	n/a	n/a	3.60	3.99, 3.82	-	-	-	2.08 – 2.01 (m, 21H)
Galactose-1	4.44 (d, J = 8.0 Hz, 1H)	3.60	3.74	4.17	n/a	4.01, 3.56	-	-	-	-
GlcNAc-6S-1	4.75	3.84	n/a	n/a	3.83	4.43 – 4.26 (m, 6H)	-	-	-	2.08 – 2.01 (m, 21H)
Galactose-2	4.54	3.59	3.73	4.22	n/a	3.77 (2H)	-	-	-	-
GlcNAc-6S-2	4.73	3.83	n/a	n/a	3.83	4.43 – 4.26 (m, 6H)	-	-	-	2.08 – 2.01 (m, 21H)
Galactose-3	4.47 (d, J = 7.3 Hz, 1H)	3.60	3.74	4.17	n/a	4.01, 3.56	-	-	-	-
GlcNAc-6S-3	4.72	3.83	n/a	n/a	3.83	4.43 – 4.26 (m, 6H)	-	-	-	2.08 – 2.01 (m, 21H)
Galactose-6S	4.64 (d, J = 7.6 Hz, 1H)	3.57	4.16	4.03	3.99	4.18 (2H)	-	-	-	-
Sialic acid-1	-	-	2.67 (dd, *J* = 12.4, 3.8 Hz, 1H), 1.72 (t, *J* = 11.1 Hz, 1H)	3.68	3.81	n/a	n/a	3.91	3.89, 3.65	2.08 – 2.01 (m, 21H)
Sialic acid-2	-	-	2.67 (dd, *J* = 12.4,	3.68	3.81	n/a	n/a	3.91	3.89, 3.65	2.08 – 2.01

			3.8 Hz, 1H), 1.72 (t, *J* = 11.1 Hz, 1H)							(m, 21H)
Sialic acid-3	-	-	2.75 (dd, *J* = 11.5, 3.7 Hz, 1H), 1.83 (t, *J* = 12.0 Hz, 1H)	3.67	3.87	n/a	n/a	3.91	3.89, 3.65	2.08 – 2.01 (m, 21H)

^{13}C (150 MHz, D_2O): δ (ppm)

	C-1	C-2	C-3	C-4	C-5	C-6	C-7	C-8	C-9	NHAc
GlcNAc	100.82	54.94	n/a	n/a	74.69	60.48	-	-	-	22.17
Galactose-1	103.56	69.24	82.66	68.41	n/a	63.69	-	-	-	-
GlcNAc-6S-1	102.69	55.04	n/a	n/a	72.88	66.65	-	-	-	22.17
Galactose-2	101.90	69.36	82.66	68.25	n/a	61.25	-	-	-	-
GlcNAc-6S-2	102.69	55.02	n/a	n/a	72.88	66.65	-	-	-	22.17
Galactose-3	103.59	69.24	82.66	68.41	n/a	63.69	-	-	-	-
GlcNAc-6S-3	102.69	55.02	n/a	n/a	72.88	66.65	-	-	-	22.17
Galactose-6S	102.59	69.48	75.18	67.19	72.52	66.59	-	-	-	-
Sialic acid-1	n/a	n/a	40.17	n/a	51.78	n/a	n/a	71.59	62.74	22.17
Sialic acid-2	n/a	n/a	40.17	n/a	51.78	n/a	n/a	71.59	62.74	22.17
Sialic acid-3	n/a	n/a	39.44	n/a	51.88	n/a	n/a	71.59	62.74	22.17

Linker	1	2	3	4	5	6
H	3.88,3.58	1.61 – 1.52 (m, 2H)	1.37 – 1.24 (m, 2H)	1.53 – 1.45 (m, 2H)	3.13 (t, J = 6.8 Hz, 2H)	5.12 (s, 2H)
C	70.02	28.25	22.31	28.55	40.29	66.67

HRMS (ESI-MS): m/z calculated for $C_{102}H_{158}N_8O_{79}S_4$ $[M-4H]^{4-}$: 721.9382; found: 721.9211.

Compound 42

42 was prepared from **41** (1.4 mg, 0.5 μmol) using the general procedure for 6-O-sulfate installation of internal galactose using CHST1. After P6 purification, **42** was obtained as a white solid (1.0 mg, 71%).

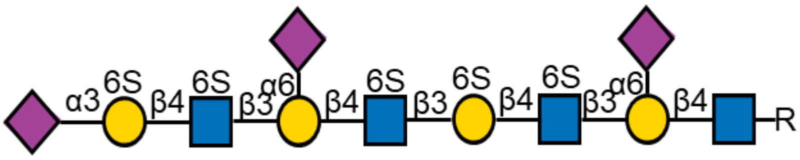

^{1}H (600 MHz, D_2O): δ (ppm)

	H-1	H-2	H-3	H-4	H-5	H-6	H-7	H-8	H-9	NHAc
GlcNAc	4.54	3.72	n/a	n/a	3.60	3.98, 3.82	-	-	-	2.08 – 2.01 (m, 21H)
Galactose-1	4.44 (d, J = 7.9 Hz, 1H)	3.60	3.74	4.17	n/a	4.02, 3.56	-	-	-	-
GlcNAc-6S-1	4.73	3.81	n/a	n/a	n/a	4.43 – 4.27 (m, 6H)	-	-	-	2.08 – 2.01 (m, 21H)
Galactose-6S-1	4.55	3.60	3.74	n/a	n/a	4.18 (4H)	-	-	-	-
GlcNAc-6S-2	4.73	3.81	n/a	n/a	n/a	4.43 – 4.27 (m, 6H)	-	-	-	2.08 – 2.01 (m, 21H)
Galactose-2	4.47	3.60	3.74	4.17	n/a	4.02, 3.56	-	-	-	-
GlcNAc-6S-3	4.73	3.81	n/a	n/a	n/a	4.43 – 4.27 (m, 6H)	-	-	-	2.08 – 2.01 (m, 21H)
Galactose-6S-2	4.64	3.58	4.15	4.03	n/a	4.18 (4H)	-	-	-	-
Sialic acid-1	-	-	2.67 (2H), 1.73 (2H)	3.68	3.81	n/a	n/a	3.91	3.89, 3.65	2.08 – 2.01 (m, 21H)
Sialic acid-2	-	-	2.67 (2H), 1.73 (2H)	3.68	3.81	n/a	n/a	3.91	3.89, 3.65	2.08 – 2.01 (m, 21H)
Sialic acid-3	-	-	2.75, 1.83	3.67	3.87	n/a	n/a	3.91	3.89, 3.65	2.08 – 2.01 (m, 21H)

^{13}C (150 MHz, D_2O): δ (ppm)

	C-1	C-2	C-3	C-4	C-5	C-6	C-7	C-8	C-9	NHAc
GlcNAc	100.82	54.97	n/a	n/a	74.69	60.37	-	-	-	22.30

Galactose-1	103.67	69.60	82.47	68.85	n/a	63.80	-	-	-	-
GlcNAc-6S-1	102.95	n/a	n/a	n/a	n/a	66.94	-	-	-	22.30
Galactose-6S-1	102.47	69.60	82.47	n/a	n/a	66.70	-	-	-	-
GlcNAc-6S-2	102.95	n/a	n/a	n/a	n/a	66.94	-	-	-	22.30
Galactose-2	103.41	69.60	82.47	68.85	n/a	63.80	-	-	-	-
GlcNAc-6S-3	102.95	n/a	n/a	n/a	n/a	66.94	-	-	-	22.30
Galactose-6S-2	102.84	69.38	75.30	67.32	n/a	66.70	-	-	-	-
Sialic acid-1	n/a	n/a	40.21	n/a	51.78	n/a	n/a	71.75	62.79	22.30
Sialic acid-2	n/a	n/a	40.21	n/a	51.78	n/a	n/a	71.75	62.79	22.30
Sialic acid-3	n/a	n/a	39.62	n/a	51.85	n/a	n/a	71.75	62.79	22.30

Linker	1	2	3	4	5	6
H	3.88,3.58	1.61 – 1.52 (m, 2H)	1.37 – 1.24 (m, 2H)	1.53 – 1.45 (m, 2H)	3.13 (t, J = 6.3 Hz, 2H)	5.12 (s, 2H)
C	70.02	28.25	22.31	28.55	40.43	66.67

HRMS (ESI-MS): m/z calculated for $C_{102}H_{158}N_8O_{82}S_5$ $[M-3H]^{3-}$: 989.2364; found: 989.2289.

Compound 43

43 was prepared from **42** (1.0 mg, 0.34 μmol) using the general procedure for the removal of sialic acid using *C. perfringens* neuraminidase. After P6 purification, **43** was obtained as a white solid (0.7 mg, quant).

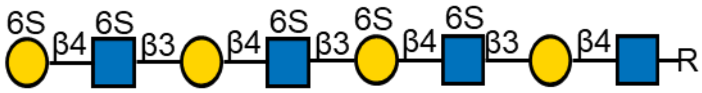

^1H (600 MHz, D_2O): δ (ppm)

	H-1	H-2	H-3	H-4	H-5	H-6	NHAc
GlcNAc	4.51	3.72	3.70	3.69	3.58	3.98, 3.82	2.09 – 2.00 (m, 12H)
Galactose-1	4.47	3.60	3.72	4.19	n/a	3.76 (4H)	-

GlcNAc-6S-1	4.72	3.83	3.77	3.78	3.81	4.43 – 4.27 (m, 6H)	2.09 – 2.00 (m, 12H)
Galactose-6S-1	4.54	3.60	3.74	4.24	3.99	4.20 (4H)	-
GlcNAc-6S-2	4.72	3.83	3.77	3.78	3.81	4.43 – 4.27 (m, 6H)	2.09 – 2.00 (m, 12H)
Galactose-2	4.52	3.60	3.72	4.19	n/a	3.76 (4H)	-
GlcNAc-6S-3	4.72	3.83	3.77	3.78	3.81	4.43 – 4.27 (m, 6H)	2.09 – 2.00 (m, 12H)
Galactose-6S-2	4.55	3.55	3.72	4.00	3.99	4.20 (4H)	-

^{13}C (150 MHz, D_2O): δ (ppm)

	C-1	C-2	C-3	C-4	C-5	C-6	NHAc
GlcNAc	101.12	55.01	72.61	78.52	75.03	60.14	22.16
Galactose-1	102.94	69.75	82.65	68.96	n/a	61.16	-
GlcNAc-6S-1	102.93	54.93	n/a	n/a	72.49	66.40	22.16
Galactose-6S-1	102.86	69.75	82.65	68.18	72.69	66.34	-
GlcNAc-6S-2	102.93	54.93	n/a	n/a	72.49	67.35	22.16
Galactose-2	102.30	69.75	82.65	68.96	n/a	61.16	-
GlcNAc-6S-3	102.93	54.93	n/a	n/a	72.49	66.40	22.16
Galactose-6S-2	103.43	70.46	n/a	67.94	72.69	66.34	-

Linker	1	2	3	4	5	6
H	3.88,3.58	1.61 – 1.52 (m, 2H)	1.37 – 1.24 (m, 2H)	1.53 – 1.45 (m, 2H)	3.14 – 3.10 (m, 2H)	5.12 (s, 2H)
C	70.02	28.25	22.31	28.55	40.43	66.67

HRMS (ESI-MS): m/z calculated for $C_{69}H_{109}N_5O_{58}S_5$ $[M-2H]^{2-}$: 1047.7174; found: 1047.6833.

Compound 45

Compound **45** was prepared from **44** (5.0 mg, 2.4 μmol) using the general procedure for the installation of β1,3GlcNAc with B3GnT2. After P2 purification, **45** was obtained as a white solid (4.4 mg, 82%). The NMR data is agreement with previous reported data.[6]

Compound 46

46 was prepared from **45** (5.0 mg, 2.2 μmol) using the general procedure for 6-O-sulfate installation of terminal GlcNAc using CHST2. After P6 purification, **46** was obtained as a white solid (4.4 mg, 85%).

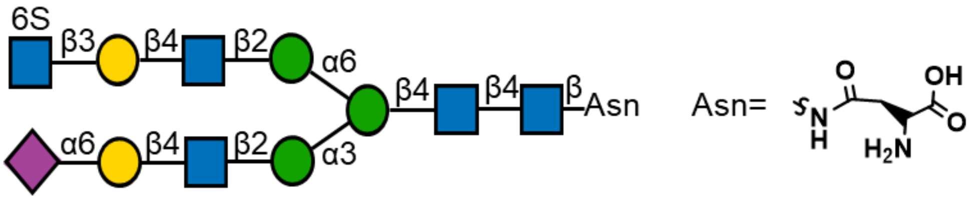

^{1}H (600 MHz, D_2O): δ (ppm)

	H-1	H-2	H-3	H-4	H-5	H-6	H-7	H-8	H-9	NHAc
GlcNAc-1	5.08 (d, J = 9.6 Hz, 1H)	3.87	n/a	3.67	3.60	3.77, 3.65	-	-	-	2.10 – 2.00 (m, 18H)
GlcNAc-2	4.63	3.80	n/a	n/a	n/a	n/a	-	-	-	2.10 – 2.00 (m, 18H)
Man-1	4.78	4.27 (s, 1H)	3.80	3.80	n/a	3.97, 3.81	-	-	-	-
Man-2	5.14 (s, 1H)	4.21	3.93	3.54	n/a	3.92, 3.63	-	-	-	-
Man-3	4.93 (s, 1H)	4.12	3.91	3.51	3.63	3.93, 3.63	-	-	-	-
GlcNAc-3	4.61	3.77	n/a	n/a	3.58	3.99, 3.85	-	-	-	2.10 – 2.00 (m, 18H)
Galactose-1	4.46	3.55	3.69	3.94	n/a	4.01, 3.56	-	-	-	-
Sialic acid	-	-	2.68 (dd, J = 12.3, 4.6 Hz, 1H), 1.73 (t, J = 12.1 Hz, 1H)	3.68	3.81	n/a	3.57	3.90	3.89, 3.66	2.10 – 2.00 (m, 18H)
GlcNAc-3’	4.59 (d, J = 8.2 Hz, 1H)	3.77	n/a	n/a	3.58	3.99, 3.85	-	-	-	2.10 – 2.00 (m, 18H)
Galactose-1’	4.47	3.58	3.74	4.18	n/a	3.76	-	-	-	-
GlcNAc-4’	4.70 (d, J = 8.5 Hz, 1H)	3.79	3.59	3.53	3.67	4.34 (d, J = 11.1 Hz, 1H),	-	-	-	2.10 – 2.00 (m, 18H)

						4.25 – 4.21 (m, 1H)				

^{13}C (150 MHz, D_2O): δ (ppm)

	C-1	C-2	C-3	C-4	C-5	C-6	C-7	C-8	C-9	NHAc
GlcNAc-1	78.09	53.53	n/a	78.73	76.25	59.92	-	-	-	22.28
GlcNAc-2	101.27	54.94	n/a	n/a	n/a	n/a	-	-	-	22.28
Man-1	100.41	70.13	80.55	65.57	n/a	65.80	-	-	-	-
Man-2	99.49	76.40	69.43	67.39	n/a	61.60	-	-	-	-
Man-3	96.92	76.30	69.43	67.28	74.32	61.49	-	-	-	-
GlcNAc-3	99.56	54.84	n/a	n/a	74.39	60.11	-	-	-	22.28
Galactose-1	103.47	70.91	72.61	68.45	n/a	63.41	-	-	-	-
Sialic acid	n/a	n/a	39.95	n/a	51.88	n/a	68.44	71.76	62.49	22.28
GlcNAc-3’	99.30	54.84	n/a	n/a	74.39	60.11	-	-	-	22.28
Galactose-1’	103.40	69.85	82.33	68.84	n/a	60.84	-	-	-	-
GlcNAc-4’	102.91	55.39	73.45	69.59	73.70	67.15	-	-	-	22.28

ASN	βCH2-Asn	αCH-Asn
H	2.95 (dd, J = 16.9, 4.2 Hz, 1H), 2.87 (dd, J = 17.2, 7.1 Hz, 1H)	4.01
C	35.26	n/a

HRMS (ESI-MS): m/z calculated for $C_{85}H_{138}N_8O_{64}S$ $[M-2H]^{2-}$: 1163.3761; found: 1163.3778.

Compound 47

47 was prepared from **46** (4.4 mg, 1.9 µmol) using the general procedure for installation of β1,4Gal using B4GalT4. After P6 purification, **47** was obtained as a white solid (4.1 mg, 88%).

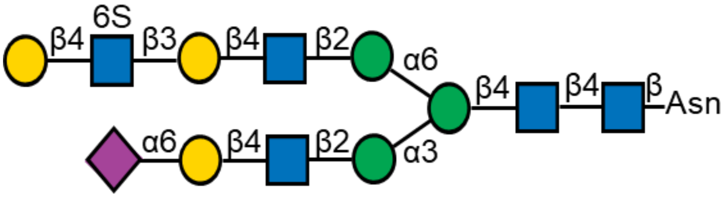

^{1}H (600 MHz, D_2O): δ (ppm)

	H-1	H-2	H-3	H-4	H-5	H-6	H-7	H-8	H-9	NHAc
GlcNAc-1	5.09 (d, J = 9.5 Hz, 1H)	3.88	n/a	3.67	3.60	3.77, 3.66	-	-	-	2.11 – 2.01 (m, 18H)
GlcNAc-2	4.63	3.80	n/a	n/a	n/a	n/a	-	-	-	2.11 – 2.01 (m, 18H)
Man-1	4.78	4.27 (s, 1H)	3.80	3.80	n/a	3.97, 3.81	-	-	-	-
Man-2	5.15 (s, 1H)	4.21	3.93	3.54	n/a	3.92, 3.63	-	-	-	-
Man-3	4.94 (s, 1H)	4.12 (s, 1H)	3.91	3.51	3.63	3.93, 3.63	-	-	-	-
GlcNAc-3	4.62	3.77	n/a	n/a	3.58	3.99, 3.85	-	-	-	2.11 – 2.01 (m, 18H)
Galactose-1	4.46	3.55	3.69	3.94	n/a	4.01, 3.55	-	-	-	-
Sialic acid	-	-	2.68, 1.73 (t, J = 12.2 Hz, 1H)	3.70	3.82	n/a	3.57	3.90	3.89, 3.66	2.11 – 2.01 (m, 18H)
GlcNAc-3’	4.59 (d, J = 8.3 Hz, 1H)	3.77	n/a	n/a	3.58	3.99, 3.85	-	-	-	2.11 – 2.01 (m, 18H)
Galactose-1’	4.47	3.60	3.73	4.20	n/a	3.77 (2H)	-	-	-	-
GlcNAc-4’	4.73	3.84	3.74	3.81	3.82	4.41 (d, J = 10.9 Hz, 1H), 4.33 (d, J = 10.5 Hz, 1H)	-	-	-	2.11 – 2.01 (m, 18H)
Galactose-2’	4.53 (d, J = 7.8 Hz, 1H)	3.55	3.69	3.94	n/a	3.77 (2H)	-	-	-	-

^{13}C (150 MHz, D_2O): δ (ppm)

	C-1	C-2	C-3	C-4	C-5	C-6	C-7	C-8	C-9	NHAc
GlcNAc-1	78.15	53.70	n/a	78.68	76.42	59.93	-	-	-	22.26
GlcNAc-2	101.39	55.03	n/a	n/a	n/a	n/a	-	-	-	22.26
Man-1	100.47	70.22	80.65	65.58	n/a	65.81	-	-	-	-
Man-2	99.66	76.48	69.33	67.45	n/a	61.72	-	-	-	-
Man-3	97.04	76.35	69.56	67.45	74.57	61.60	-	-	-	-
GlcNAc-3	99.56	54.84	n/a	n/a	74.69	60.19	-	-	-	22.26

Galactose-1	103.58	70.98	72.54	68.57	n/a	63.47	-	-	-	-
Sialic acid	n/a	n/a	40.10	n/a	51.88	n/a	68.42	71.74	62.56	22.26
GlcNAc-3’	99.41	54.84	n/a	n/a	74.69	60.19	-	-	-	22.26
Galactose-1’	103.33	70.01	82.26	68.18	n/a	60.80	-	-	-	-
GlcNAc-4’	102.88	55.09	72.54	77.58	72.82	66.36	-	-	-	22.26
Galactose-2’	102.49	71.01	72.65	68.67	n/a	60.80	-	-	-	-

HRMS (ESI-MS): m/z calculated for $C_{91}H_{148}N_8O_{69}S$ $[M-2H]^{2-}$: 1244.4025; found: 1244.3769.

Compound 48

48 was prepared from **47** (4.1 mg, 1.7 µmol) using the general procedure for installation of β1,3GlcNAc using B3GnT2. After P6 purification, **48** was obtained as a white solid (4.5 mg, quant).

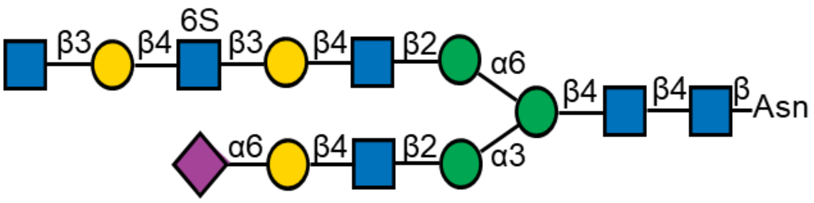

^{1}H (600 MHz, D_2O): δ (ppm)

	H-1	H-2	H-3	H-4	H-5	H-6	H-7	H-8	H-9	NHAc
GlcNAc-1	5.08	3.88	n/a	3.67	3.60	3.77, 3.66	-	-	-	2.10 – 2.01 (m, 21H)
GlcNAc-2	4.63	3.80	n/a	n/a	n/a	n/a	-	-	-	2.10 – 2.01 (m, 21H)
Man-1	4.78	4.26 (s, 1H)	3.80	3.80	n/a	3.97, 3.81	-	-	-	-
Man-2	5.15 (s, 1H)	4.20 (s, 1H)	3.93	3.54	n/a	3.92, 3.63	-	-	-	-
Man-3	4.94 (s, 1H)	4.12 (s, 1H)	3.91	3.51	3.63	3.93, 3.63	-	-	-	-
GlcNAc-3	4.62	3.77	n/a	n/a	3.58	3.99, 3.85	-	-	-	2.10 – 2.01 (m, 21H)
Galactose-1	4.46	3.55	3.69	3.94	n/a	4.00, 3.55	-	-	-	-
Sialic acid	-	-	2.68, 1.73	3.70	3.83	n/a	3.57	3.90	3.89, 3.66	2.10 – 2.01

										(m, 21H)
GlcNAc-3’	4.59	3.77	n/a	n/a	3.58	3.99, 3.85	-	-	-	2.10 – 2.01 (m, 21H)
Galactose-1’	4.47	3.59	3.73	4.20	n/a	3.77 (2H)	-	-	-	-
GlcNAc-4’	4.73	3.83	3.74	3.80	3.82	4.40， 4.32	-	-	-	2.10 – 2.01 (m, 21H)
Galactose-2’	4.52	3.57	3.73	4.16	n/a	3.77 (2H)	-	-	-	-
GlcNAc-5’	4.70	3.77	n/a	n/a	3.47	3.90， 3.77	-	-	-	2.10 – 2.01 (m, 21H)

^{13}C (150 MHz, D_2O): δ (ppm)

	C-1	C-2	C-3	C-4	C-5	C-6	C-7	C-8	C-9	NHAc
GlcNAc-1	78.15	53.70	n/a	78.79	76.30	59.92	-	-	-	22.29
GlcNAc-2	101.39	55.00	n/a	n/a	n/a	n/a	-	-	-	22.29
Man-1	100.47	70.22	80.65	65.58	n/a	65.81	-	-	-	-
Man-2	99.66	76.48	69.33	67.45	n/a	61.72	-	-	-	-
Man-3	97.04	76.35	69.56	67.45	74.57	61.60	-	-	-	-
GlcNAc-3	99.56	55.10	n/a	n/a	74.69	60.19	-	-	-	22.29
Galactose-1	103.58	70.94	72.58	68.43	n/a	63.47	-	-	-	-
Sialic acid	n/a	n/a	40.10	n/a	51.88	n/a	68.56	71.74	62.56	22.29
GlcNAc-3’	99.41	55.10	n/a	n/a	74.69	60.19	-	-	-	22.29
Galactose-1’	103.11	69.53	82.18	68.34	n/a	60.81	-	-	-	-
GlcNAc-4’	102.98	54.93	72.50	77.95	72.44	66.50	-	-	-	22.29
Galactose-2’	102.72	70.62	82.19	68.34	n/a	60.81	-	-	-	-
GlcNAc-5’	102.98	55.05	n/a	n/a	n/a	60.47	-	-	-	22.29

HRMS (ESI-MS): m/z calculated for $C_{99}H_{161}N_9O_{74}S$ $[M-2H]^{2-}$: 1346.4438; found: 1346.4001.

Compound 49

49 was prepared from **48** (4.5 mg, 1.7 μmol) using the general procedure for 6-O-sulfate installation of terminal GlcNAc using CHST2. After P6 purification, **49** was obtained as a white solid (4.5 mg, 97%).

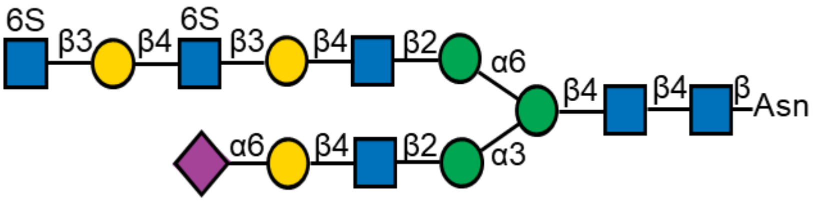

^{1}H (600 MHz, D_2O): δ (ppm)

	H-1	H-2	H-3	H-4	H-5	H-6	H-7	H-8	H-9	NHAc
GlcNAc-1	5.08 (d, J = 9.7 Hz, 1H)	3.87	n/a	3.67	3.60	3.77, 3.66	-	-	-	2.11 – 2.00 (m, 21H)
GlcNAc-2	4.63	3.80	n/a	n/a	n/a	n/a	-	-	-	2.11 – 2.00 (m, 21H)
Man-1	4.78	4.26	3.80	3.80	n/a	3.97, 3.81	-	-	-	-
Man-2	5.14 (s, 1H)	4.21	3.93	3.54	n/a	3.92, 3.63	-	-	-	-
Man-3	4.94 (s, 1H)	4.12 (s, 1H)	3.91	3.51	3.63	3.93, 3.63	-	-	-	-
GlcNAc-3	4.61	3.77	n/a	n/a	3.58	3.99, 3.85	-	-	-	2.11 – 2.00 (m, 21H)
Galactose-1	4.46	3.55	3.69	3.94	n/a	4.00, 3.55	-	-	-	-
Sialic acid	-	-	2.68 (dd, J = 12.4, 4.7 Hz, 1H), 1.73 (t, J = 12.2 Hz, 1H)	3.68	3.82	n/a	3.57	3.90	3.89, 3.65	2.11 – 2.00 (m, 21H)
GlcNAc-3'	4.59 (d, J = 8.2 Hz, 1H)	3.76	3.70	3.70	3.58	3.99, 3.85	-	-	-	2.11 – 2.00 (m, 21H)
Galactose-1'	4.47	3.60	3.73	4.20	n/a	3.76 (4H)	-	-	-	-
GlcNAc-4'	4.72 (d, J = 8.5 Hz, 1H)	3.84	3.74	3.77	3.81	4.41 (d, J = 10.5 Hz, 1H),	-	-	-	2.11 – 2.00 (m, 21H)

						4.32 – 4.28 (m, 1H)				
Galactose-2’	4.52 (d, J = 7.9 Hz, 1H)	3.59	3.73	4.20	n/a	3.76 (4H)	-	-	-	-
GlcNAc-5’	4.70 (d, J = 8.5 Hz, 1H)	3.81	3.59	3.53	3.67	4.35 – 4.32 (m, 1H), 4.26 – 4.23 (m, 1H)	-	-	-	2.11 – 2.00 (m, 21H)

^{13}C (150 MHz, D_2O): δ (ppm)

	C-1	C-2	C-3	C-4	C-5	C-6	C-7	C-8	C-9	NHAc
GlcNAc-1	78.06	53.53	n/a	n/a	n/a	59.92	-	-	-	22.28
GlcNAc-2	101.30	55.03	n/a	n/a	n/a	n/a	-	-	-	22.28
Man-1	100.47	70.22	80.65	65.58	n/a	65.81	-	-	-	-
Man-2	99.66	76.48	69.33	67.45	n/a	61.72	-	-	-	-
Man-3	97.04	76.35	69.56	67.45	74.57	61.60	-	-	-	-
GlcNAc-3	99.48	54.84	n/a	n/a	74.69	60.19	-	-	-	22.28
Galactose-1	103.58	70.94	72.61	68.45	n/a	63.47	-	-	-	-
Sialic acid	n/a	n/a	40.10	68.33	51.88	n/a	68.56	71.74	62.56	22.28
GlcNAc-3’	99.41	55.10	72.63	78.94	74.69	60.19	-	-	-	22.28
Galactose-1’	103.11	70.07	82.60	68.44	n/a	60.99	-	-	-	-
GlcNAc-4’	102.87	55.09	72.55	78.80	72.73	66.51	-	-	-	22.28
Galactose-2’	102.74	70.07	82.60	68.44	n/a	60.99	-	-	-	-
GlcNAc-5’	102.87	55.04	73.58	69.52	73.54	66.95	-	-	-	22.28

HRMS (ESI-MS): m/z calculated for $C_{99}H_{161}N_9O_{77}S_2$ $[M-2H]^{2-}$: 1386.4222; found: 1386.3988.

Compound 50

50 was prepared from **49** (4.5 mg, 1.6 μmol) using the general procedure for installation of α1,3Fuc using FUT6. After P6 purification, **50** was obtained as a white solid (4.8 mg, 96%).

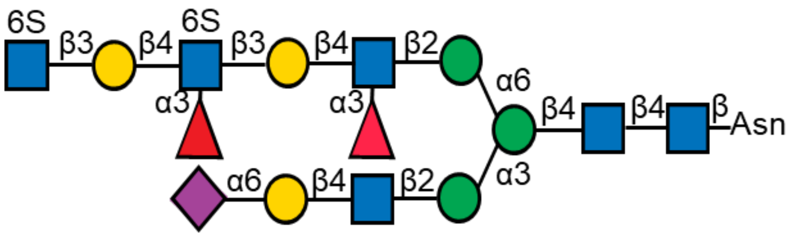

^{1}H (600 MHz, D_2O): δ (ppm)

	H-1	H-2	H-3	H-4	H-5	H-6	H-7	H-8	H-9	NHAc
GlcNAc-1	5.08 (d, J = 9.6 Hz, 1H)	3.87	n/a	3.67	3.60	3.77, 3.66	-	-	-	2.11 – 2.00 (m, 21H)
GlcNAc-2	4.63	3.80	n/a	n/a	n/a	n/a	-	-	-	2.11 – 2.00 (m, 21H)
Man-1	4.78	4.27 (s, 1H)	3.80	3.80	n/a	3.97, 3.81	-	-	-	-
Man-2	5.14 (s, 1H)	4.20	3.93	3.54	n/a	3.92, 3.63	-	-	-	-
Man-3	4.92 (s, 1H)	4.11	3.91	3.51	3.63	3.93, 3.63	-	-	-	-
GlcNAc-3	4.61	3.77	n/a	n/a	3.58	3.99, 3.85	-	-	-	2.11 – 2.00 (m, 21H)
Galactose-1	4.46	3.55	3.69	3.94	n/a	4.00, 3.55	-	-	-	-
Sialic acid	-	-	2.68 (dd, J = 12.4, 4.6 Hz, 1H), 1.73 (t, J = 12.1 Hz, 1H)	3.68	3.82	n/a	3.57	3.90	3.89, 3.65	2.11 – 2.00 (m, 21H)
GlcNAc-3’	4.59	3.95	n/a	n/a	3.59	3.99, 3.85	-	-	-	2.11 – 2.00 (m, 21H)
Galactose-1’	4.44	3.52	3.70	4.13	n/a	3.73 (4H)	-	-	-	-
GlcNAc-4’	4.74 (d, J = 8.7 Hz, 1H)	3.99	3.90	4.01	3.81	4.38, 4.34	-	-	-	2.11 – 2.00 (m, 21H)
Galactose-2’	4.52 (d, J = 7.9 Hz, 1H)	3.52	3.71	4.14	n/a	3.73 (4H)	-	-	-	-
GlcNAc-5’	4.69 (d, J = 8.3 Hz, 1H)	3.79	3.59	3.53	3.67	4.32, 4.26	-	-	-	2.11 – 2.00 (m, 21H)
Fucose-1	5.13	3.70	n/a	n/a	4.83	1.19 – 1.15 (m, 6H)	-	-	-	-
Fucose-2	5.13	3.70	n/a	n/a	4.83	1.19 – 1.15 (m, 6H)	-	-	-	-

^{13}C (150 MHz, D_2O): δ (ppm)

	C-1	C-2	C-3	C-4	C-5	C-6	C-7	C-8	C-9	NHAc
GlcNAc-1	78.06	53.73	n/a	n/a	n/a	59.92	-	-	-	22.28
GlcNAc-2	101.30	55.03	n/a	n/a	n/a	n/a	-	-	-	22.28
Man-1	100.47	70.22	80.65	65.58	n/a	65.81	-	-	-	-
Man-2	99.66	76.48	69.33	67.45	n/a	61.72	-	-	-	-
Man-3	96.84	76.35	69.56	67.45	74.57	61.60	-	-	-	-
GlcNAc-3	99.48	54.84	n/a	n/a	74.69	60.19	-	-	-	22.28
Galactose-1	103.58	70.94	72.61	68.45	n/a	63.47	-	-	-	-
Sialic acid	n/a	n/a	40.10	68.33	51.88	n/a	68.56	71.74	62.56	22.28
GlcNAc-3’	99.41	55.73	n/a	n/a	74.69	60.19	-	-	-	22.28
Galactose-1’	101.91	70.34	82.45	68.19	n/a	61.68	-	-	-	-
GlcNAc-4’	102.47	55.84	n/a	72.83	72.73	66.33	-	-	-	22.28
Galactose-2’	101.74	70.34	82.45	68.19	n/a	61.68	-	-	-	-
GlcNAc-5’	102.87	55.20	73.58	69.52	73.54	66.98	-	-	-	22.28
Fucose-1	98.78	n/a	n/a	n/a	66.68	15.31	-	-	-	-
Fucose-2	98.78	n/a	n/a	n/a	66.68	15.31	-	-	-	-

HRMS (ESI-MS): m/z calculated for $C_{111}H_{181}N_9O_{85}S_2$ $[M-2H]^{2-}$: 1532.4801; found: 1532.4134.

Compound 51

51 was prepared from **50** (4.8 mg, 1.6 µmol) using the general procedure for installation of β1,4Gal using B4GalT4. After P6 purification, **51** was obtained as a white solid (4.8 mg, 95%).

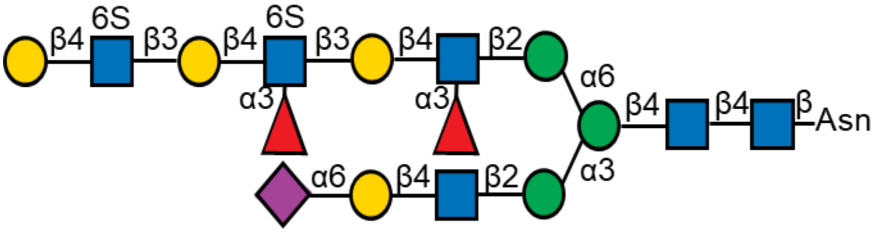

^{1}H (600 MHz, D_2O): δ (ppm)

	H-1	H-2	H-3	H-4	H-5	H-6	H-7	H-8	H-9	NHAc
GlcNAc-1	5.08 (d, J = 9.7 Hz, 1H)	3.88	n/a	3.67	3.60	3.77, 3.66	-	-	-	2.11 – 2.00 (m, 21H)
GlcNAc-2	4.63	3.80	n/a	n/a	n/a	n/a	-	-	-	2.11 – 2.00 (m, 21H)
Man-1	4.79	4.27 (s, 1H)	3.80	3.80	n/a	3.97, 3.81	-	-	-	-

Man-2	5.14 (s, 1H)	4.21 (d, J = 3.3 Hz, 1H)	3.93	3.54	n/a	3.92, 3.63	-	-	-	-
Man-3	4.92 (s, 1H)	4.11 (d, J = 3.4 Hz, 1H)	3.91	3.51	3.63	3.93, 3.63	-	-	-	-
GlcNAc-3	4.61	3.77	n/a	n/a	3.58	3.99, 3.85	-	-	-	2.11 – 2.00 (m, 21H)
Galactose-1	4.46	3.55	3.69	3.94	n/a	4.00, 3.55	-	-	-	-
Sialic acid	-	-	2.68 (dd, J = 12.4, 4.6 Hz, 1H), 1.73 (t, J = 12.1 Hz, 1H)	3.68	3.82	n/a	3.57	3.90	3.89, 3.65	2.11 – 2.00 (m, 21H)
GlcNAc-3’	4.59	3.93	n/a	n/a	3.59	3.99, 3.85	-	-	-	2.11 – 2.00 (m, 21H)
Galactose-1’	4.44	3.52	3.70	4.13	n/a	3.73 (4H)	-	-	-	-
GlcNAc-4’	4.74	3.99	3.90	4.01	3.81	4.40 – 4.31 (m, 4H)	-	-	-	2.11 – 2.00 (m, 21H)
Galactose-2’	4.52 (d, J = 7.7 Hz, 1H)	3.52	3.71	4.14 (d, *J* = 3.2 Hz, 1H)	n/a	3.73 (4H)	-	-	-	-
GlcNAc-5’	4.72 (d, J = 8.7 Hz, 1H)	3.84	3.73	3.81	3.81	4.40 – 4.31 (m, 4H)	-	-	-	2.11 – 2.00 (m, 21H)
Galactose-3’	4.55 (d, J = 7.8 Hz, 1H)	3.54	3.69	3.94	n/a	3.77 (2H)	-	-	-	-
Fucose-1	5.13	3.70	n/a	n/a	4.83	1.16 (dd, *J* = 6.7, 2.6 Hz, 6H)	-	-	-	-
Fucose-2	5.13	3.70	n/a	n/a	4.83	1.16 (dd, *J* = 6.7, 2.6 Hz, 6H)	-	-	-	-

^{13}C (150 MHz, D_2O): δ (ppm)

	C-1	C-2	C-3	C-4	C-5	C-6	C-7	C-8	C-9	NHAc
GlcNAc-1	78.06	53.73	n/a	n/a	n/a	59.92	-	-	-	22.28
GlcNAc-2	101.30	55.03	n/a	n/a	n/a	n/a	-	-	-	22.28
Man-1	100.47	70.22	80.65	65.58	n/a	65.81	-	-	-	-
Man-2	99.66	76.48	69.33	67.45	n/a	61.72	-	-	-	-
Man-3	96.84	76.35	69.56	67.45	74.57	61.60	-	-	-	-
GlcNAc-3	99.48	54.84	n/a	n/a	74.69	60.19	-	-	-	22.28
Galactose-1	103.44	70.94	72.61	68.45	n/a	63.47	-	-	-	-
Sialic acid	n/a	n/a	40.10	68.33	51.88	n/a	68.56	71.74	62.56	22.28
GlcNAc-3’	99.41	55.65	n/a	n/a	74.69	60.19	-	-	-	22.28
Galactose-1’	102.15	70.34	82.25	68.19	n/a	61.51	-	-	-	-
GlcNAc-4’	102.47	55.84	n/a	72.83	72.73	66.20	-	-	-	22.28
Galactose-2’	101.57	70.34	82.25	68.19	n/a	61.51	-	-	-	-
GlcNAc-5’	102.69	55.20	72.58	77.16	72.73	66.20	-	-	-	22.28
Galactose-3’	102.36	71.12	72.74	68.75	n/a	60.83	-	-	-	-
Fucose-1	98.53	n/a	n/a	n/a	66.68	15.31	-	-	-	-
Fucose-2	98.53	n/a	n/a	n/a	66.68	15.31	-	-	-	-

HRMS (ESI-MS): m/z calculated for $C_{117}H_{191}N_9O_{90}S_2$ $[M-2H]^{2-}$: 1613.5066; found: 1613.4448.

Compound 52

52 was prepared from **51** (4.8 mg, 1.5 μmol) using the general procedure for removal of installation of α2,3Neu5Ac using PmST1 M144D. After P6 purification, **52** was obtained as a white solid (4.8 mg, 92%).

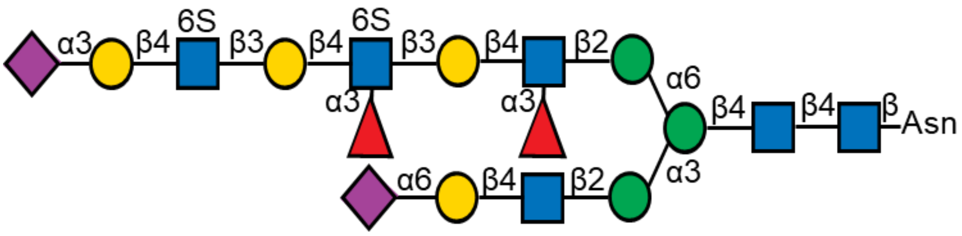

^{1}H (600 MHz, D_2O): δ (ppm)

	H-1	H-2	H-3	H-4	H-5	H-6	H-7	H-8	H-9	NHAc
GlcNAc-1	5.08 (d, J = 9.7 Hz, 1H)	3.88	n/a	3.67	3.60	3.77, 3.66	-	-	-	2.10 – 1.99 (m, 24H)
GlcNAc-2	4.63	3.80	n/a	n/a	n/a	n/a	-	-	-	2.10 – 1.99

										(m, 24H)
Man-1	4.79	4.26 (s, 1H)	3.80	3.80	n/a	3.97, 3.81	-	-	-	-
Man-2	5.14 (s, 1H)	4.20 (d, J = 3.4 Hz, 1H)	3.93	3.54	n/a	3.92, 3.63	-	-	-	-
Man-3	4.92 (s, 1H)	4.11 (s, 1H)	3.91	3.51	3.63	3.93, 3.63	-	-	-	-
GlcNAc-3	4.62	3.77	n/a	n/a	3.58	3.99, 3.85	-	-	-	2.10 – 1.99 (m, 24H)
Galactose-1	4.45	3.55	3.69	3.94	n/a	4.00, 3.55	-	-	-	-
Sialic acid-1	-	-	2.68 (d, J = 11.8 Hz, 1H), 1.73 (t, J = 12.0 Hz, 1H)	3.68	3.82	n/a	3.57	3.90	3.89, 3.65	2.10 – 1.99 (m, 24H)
GlcNAc-3’	4.59	3.94	n/a	n/a	3.58	3.99, 3.85	-	-	-	2.10 – 1.99 (m, 24H)
Galactose-1’	4.44	3.52	3.70	4.13	n/a	3.73 (4H)	-	-	-	-
GlcNAc-4’	4.74	3.99	3.90	4.01	3.82	4.40 – 4.31 (m, 4H)	-	-	-	2.10 – 1.99 (m, 24H)
Galactose-2’	4.51 (d, J = 7.3 Hz, 1H)	3.52	3.72	4.15	n/a	3.73 (4H)	-	-	-	-
GlcNAc-5’	4.71	3.84	3.73	3.81	3.82	4.40 – 4.31 (m, 4H)	-	-	-	2.10 – 1.99 (m, 24H)
Galactose-3’	4.62	3.54	4.13	3.98	n/a	3.77 (2H)	-	-	-	-
Sialic acid-2	-	-	2.76, 1.82	3.69	3.87	n/a	n/a	n/a	3.89, 3.65	2.10 – 1.99 (m, 24H)
Fucose-1	5.13	3.70	n/a	n/a	4.83	1.16 (d, *J* = 6.3 Hz, 6H)	-	-	-	-
Fucose-2	5.13	3.70	n/a	n/a	4.83	1.16 (d, *J* = 6.3 Hz, 6H)	-	-	-	-

^{13}C (150 MHz, D_2O): δ (ppm)

	C-1	C-2	C-3	C-4	C-5	C-6	C-7	C-8	C-9	NHAc
GlcNAc-1	78.06	53.73	n/a	n/a	n/a	59.92	-	-	-	22.28
GlcNAc-2	101.30	55.03	n/a	n/a	n/a	n/a	-	-	-	22.28
Man-1	100.47	70.22	80.65	65.58	n/a	65.81	-	-	-	-
Man-2	99.66	76.48	69.33	67.45	n/a	61.72	-	-	-	-
Man-3	96.84	76.35	69.56	67.45	74.57	61.60	-	-	-	-
GlcNAc-3	99.48	54.84	n/a	n/a	74.69	60.19	-	-	-	22.28
Galactose-1	103.44	70.94	72.61	68.45	n/a	63.47	-	-	-	-
Sialic acid-1	n/a	n/a	40.10	68.33	51.88	n/a	68.56	71.74	62.56	22.28
GlcNAc-3’	99.41	55.51	n/a	n/a	74.69	60.19	-	-	-	22.28
Galactose-1’	102.15	70.34	82.25	68.19	n/a	61.51	-	-	-	-
GlcNAc-4’	102.47	56.07	n/a	72.83	72.73	66.32	-	-	-	22.28
Galactose-2’	101.67	70.34	82.25	68.19	n/a	61.51	-	-	-	-
GlcNAc-5’	102.94	55.20	72.58	77.16	72.73	66.32	-	-	-	22.28
Galactose-3’	102.19	69.29	75.60	68.39	n/a	61.30	-	-	-	-
Sialic acid-2	n/a	n/a	39.80	n/a	51.76	n/a	n/a	n/a	62.56	22.28
Fucose-1	98.92	n/a	n/a	n/a	66.68	15.31	-	-	-	-
Fucose-2	98.92	n/a	n/a	n/a	66.68	15.31	-	-	-	-

HRMS (ESI-MS): m/z calculated for $C_{128}H_{208}N_{10}O_{98}S_2$ $[M-2H]^{2-}$: 1759.0543; found: 1759.0267.

Compound 53

53 was prepared from **52** (3.5 mg, 1.0 μmol) using the general procedure for 6-O-sulfate installation of internal Galactose using CHST1. After P6 purification, **53** was obtained as a white solid (2.9 mg, 81%).

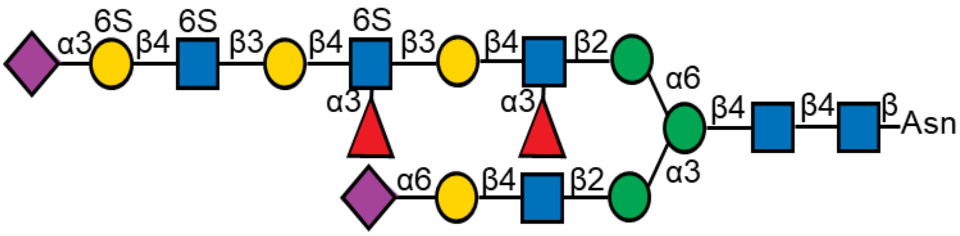

^{1}H (600 MHz, D_2O): δ (ppm)

	H-1	H-2	H-3	H-4	H-5	H-6	H-7	H-8	H-9	NHAc
GlcNAc-1	5.08 (d, J = 9.7 Hz, 1H)	3.88	n/a	3.67	3.60	3.77, 3.66	-	-	-	2.10 – 1.99 (m, 24H)

GlcNAc-2	4.63	3.80	n/a	n/a	n/a	n/a	-	-	-	2.10 – 1.99 (m, 24H)
Man-1	4.79	4.27 (s, 1H)	3.80	3.80	n/a	3.97, 3.81	-	-	-	-
Man-2	5.14 (s, 1H)	4.21 (d, J = 3.5 Hz, 1H)	3.93	3.54	n/a	3.92, 3.63	-	-	-	-
Man-3	4.92 (s, 1H)	4.11 (s, 1H)	3.91	3.51	3.63	3.93, 3.63	-	-	-	-
GlcNAc-3	4.61	3.77	n/a	n/a	3.58	3.99, 3.85	-	-	-	2.10 – 1.99 (m, 24H)
Galactose-1	4.46	3.55	3.69	3.94	n/a	4.00, 3.55	-	-	-	-
Sialic acid-1	-	-	2.68 (dd, J = 12.8, 4.4 Hz, 1H), 1.73 (t, J = 12.1 Hz, 1H)	3.68	3.82	n/a	3.57	3.90	3.89, 3.65	2.10 – 1.99 (m, 24H)
GlcNAc-3’	4.59	3.93	n/a	n/a	3.58	3.99, 3.85	-	-	-	2.10 – 1.99 (m, 24H)
Galactose-1’	4.44	3.52	3.71	4.13	n/a	3.73 (4H)	-	-	-	-
GlcNAc-4’	4.74	3.99	3.90	4.01	3.82	4.42 – 4.30 (m, 4H)	-	-	-	2.10 – 1.99 (m, 24H)
Galactose-2’	4.51 (d, J = 7.6 Hz, 1H)	3.52	3.72	4.15	n/a	3.73 (4H)	-	-	-	-
GlcNAc-5’	4.71	3.84	n/a	n/a	3.82	4.42 – 4.30 (m, 4H)	-	-	-	2.10 – 1.99 (m, 24H)
Galactose-3’	4.63	3.57	4.16	4.03	3.99	4.19 (2H)	-	-	-	-
Sialic acid-2	-	-	2.76, 1.83	3.69	3.87	n/a	n/a	n/a	3.89, 3.65	2.10 – 1.99 (m, 24H)
Fucose-1	5.13	3.70	n/a	n/a	4.83	1.16 (d, *J* = 6.6 Hz, 6H)	-	-	-	-
Fucose-2	5.13	3.70	n/a	n/a	4.83	1.16 (d, *J* =	-	-	-	-

						6.6 Hz, 6H)				

^{13}C (150 MHz, D_2O): δ (ppm)

	C-1	C-2	C-3	C-4	C-5	C-6	C-7	C-8	C-9	NHAc
GlcNAc-1	78.06	53.73	n/a	n/a	n/a	59.92	-	-	-	22.28
GlcNAc-2	101.30	55.03	n/a	n/a	n/a	n/a	-	-	-	22.28
Man-1	100.47	70.22	80.65	65.58	n/a	65.81	-	-	-	-
Man-2	99.66	76.48	69.33	67.45	n/a	61.72	-	-	-	-
Man-3	96.84	76.35	69.56	67.45	74.57	61.60	-	-	-	-
GlcNAc-3	99.48	54.84	n/a	n/a	74.69	60.19	-	-	-	22.28
Galactose-1	103.44	70.94	72.61	68.45	n/a	63.47	-	-	-	-
Sialic acid-1	n/a	n/a	40.10	68.33	51.88	n/a	68.56	71.74	62.56	22.28
GlcNAc-3’	99.41	55.51	n/a	n/a	74.69	60.19	-	-	-	22.28
Galactose-1’	101.88	70.34	82.13	68.19	n/a	61.51	-	-	-	-
GlcNAc-4’	102.19	55.73	n/a	72.83	72.73	66.32	-	-	-	22.28
Galactose-2’	101.45	70.34	82.13	68.19	n/a	61.51	-	-	-	-
GlcNAc-5’	102.68	55.20	n/a	n/a	72.73	66.32	-	-	-	22.28
Galactose-3’	102.33	68.97	75.26	67.01	72.58	66.96	-	-	-	-
Sialic acid-2	n/a	n/a	39.50	n/a	51.76	n/a	n/a	n/a	62.56	22.28
Fucose-1	98.62	n/a	n/a	n/a	66.68	15.31	-	-	-	-
Fucose-2	98.62	n/a	n/a	n/a	66.68	15.31	-	-	-	-

HRMS (ESI-MS): m/z calculated for $C_{128}H_{207}N_{10}O_{101}S_3$ $[M-3H]^{3-}$: 1199.0194; found: 1199.0156.

Compound 54

54 was prepared from **53** (1.8 mg, 0.5 µmol) using the general procedure for removal of fucose using fucosidase from *R. gnavus* E1. After P6 purification, **54** was obtained as a white solid (1.6 mg, quant).

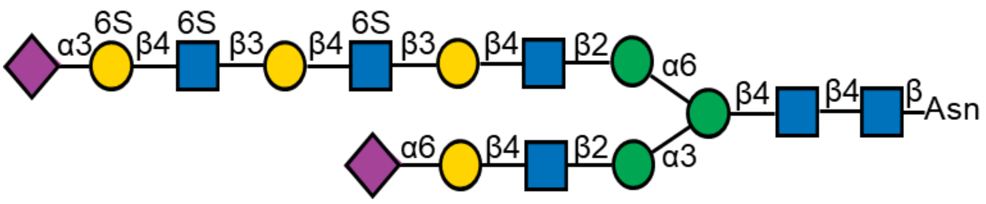

^{1}H (600 MHz, D_2O): δ (ppm)

	H-1	H-2	H-3	H-4	H-5	H-6	H-7	H-8	H-9	NHAc
GlcNAc-1	5.08 (d, J = 9.8 Hz, 1H)	3.88	n/a	3.67	3.60	3.77, 3.66	-	-	-	2.10 – 1.99 (m, 24H)
GlcNAc-2	4.63	3.80	n/a	n/a	n/a	n/a	-	-	-	2.10 – 1.99 (m, 24H)
Man-1	4.78	4.26 (s, 1H)	3.80	3.80	n/a	3.97, 3.81	-	-	-	-
Man-2	5.14 (s, 1H)	4.21	3.93	3.54	n/a	3.92, 3.63	-	-	-	-
Man-3	4.93 (s, 1H)	4.12 (s, 1H)	3.91	3.51	3.63	3.93, 3.63	-	-	-	-
GlcNAc-3	4.61	3.77	n/a	n/a	3.58	3.99, 3.85	-	-	-	2.10 – 1.99 (m, 24H)
Galactose-1	4.45	3.55	3.68	3.94	n/a	4.00, 3.55	-	-	-	-
Sialic acid-1	-	-	2.68 (dd, J = 12.5, 4.3 Hz, 1H), 1.73 (t, J = 12.1 Hz, 1H)	3.68	3.82	n/a	3.57	3.90	3.90, 3.65	2.10 – 1.99 (m, 24H)
GlcNAc-3’	4.59	3.75	n/a	n/a	3.58	3.99, 3.85	-	-	-	2.10 – 1.99 (m, 24H)
Galactose-1’	4.47	3.60	3.72	4.21	n/a	3.77 (4H)	-	-	-	-
GlcNAc-4’	4.72	3.84	n/a	n/a	3.83	4.41 (d, *J* = 11.1 Hz, 2H), 4.35 – 4.28 (m, 2H)	-	-	-	2.10 – 1.99 (m, 24H)
Galactose-2’	4.52 (d, *J* = 7.7 Hz, 1H)	3.59	3.73	4.21	n/a	3.77 (4H)	-	-	-	-
GlcNAc-5’	4.71	3.84	n/a	n/a	3.83	4.41 (d, *J* = 11.1 Hz, 2H), 4.35 – 4.28	-	-	-	2.10 – 1.99 (m, 24H)

						(m, 2H)				
Galactose-3’	4.63	3.58	4.16	4.03	3.99	4.18 (2H)	-	-	-	-
Sialic acid-2	-	-	2.76, 1.82 (t, J = 12.2 Hz, 1H)	3.69	3.87	n/a	n/a	n/a	3.90, 3.65	2.10 – 1.99 (m, 24H)

^{13}C (150 MHz, D_2O): δ (ppm)

	C-1	C-2	C-3	C-4	C-5	C-6	C-7	C-8	C-9	NHAc
GlcNAc-1	78.06	53.73	n/a	n/a	n/a	59.92	-	-	-	22.28
GlcNAc-2	101.30	55.03	n/a	n/a	n/a	n/a	-	-	-	22.28
Man-1	100.47	70.22	80.65	65.58	n/a	65.81	-	-	-	-
Man-2	99.66	76.48	69.33	67.45	n/a	61.72	-	-	-	-
Man-3	96.84	76.35	69.56	67.45	74.57	61.60	-	-	-	-
GlcNAc-3	99.48	54.84	n/a	n/a	74.69	60.19	-	-	-	22.28
Galactose-1	103.44	70.94	72.61	68.45	n/a	63.47	-	-	-	-
Sialic acid-1	n/a	n/a	40.10	68.33	51.88	n/a	68.56	71.74	62.56	22.28
GlcNAc-3’	99.41	54.80	n/a	n/a	74.69	60.19	-	-	-	22.28
Galactose-1’	103.19	69.24	82.64	68.41	n/a	61.11	-	-	-	-
GlcNAc-4’	102.87	55.07	n/a	n/a	72.46	66.71	-	-	-	22.28
Galactose-2’	102.73	69.24	82.64	68.41	n/a	61.11	-	-	-	-
GlcNAc-5’	102.87	55.07	n/a	n/a	72.46	66.71	-	-	-	22.28
Galactose-3’	102.62	69.24	75.16	67.36	72.43	66.96	-	-	-	-
Sialic acid-2	n/a	n/a	39.58	n/a	51.76	n/a	n/a	n/a	62.56	22.28

HRMS (ESI-MS): m/z calculated for $C_{116}H_{187}N_{10}O_{93}S_3$ $[M-3H]^{3-}$: 1101.6474; found: 1101.6221.

Compound S18

S18 was prepared from **7** (0.4 mg, 0.20 μmol) using the general procedure for Cbz deprotection using $Pd(OH)_2$ reduction. After purification, **S18** was obtained as a white solid (150 μg, 45%).

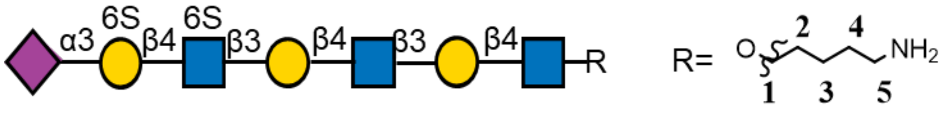

^{1}H (600 MHz, D_2O): δ (ppm)

	H-1	H-2	H-3	H-4	H-5	H-6	H-7	H-8	H-9	NHAc
GlcNAc-1	4.53	3.73	3.70	3.70	3.59	3.97, 3.84	-	-	-	2.06 – 1.99 (m, 12H)
Galactose-1	4.46	3.59	3.73	4.16	3.72	3.76	-	-	-	-
GlcNAc-2	4.71	3.81	3.73	3.72	3.59	3.97, 3.84	-	-	-	2.06 – 1.99 (m, 12H)
Galactose-2	4.48	3.59	3.73	4.21	3.72	3.76	-	-	-	-
GlcNAc-6S	4.71	3.81	n/a	n/a	3.86	4.45, 4.29	-	-	-	2.06 – 1.99 (m, 12H)
Galactose-6S	4.63 (d, J = 7.6 Hz, 1H)	3.59	4.15	4.03	n/a	4.19	-	-	-	-
Sialic acid	-	-	2.76 (dd, J = 12.5, 4.6 Hz, 1H), 1.82 (t, J = 12.1 Hz, 1H)	3.68	3.86	n/a	n/a	n/a	n/a	2.06 – 1.99 (m, 12H)

^{13}C (150 MHz, D_2O): δ (ppm)

	C-1	C-2	C-3	C-4	C-5	C-6	C-7	C-8	C-9	NHAc
GlcNAc-1	101.21	55.00	72.52	78.46	74.87	59.95	-	-	-	2.06 – 1.99 (m, 3H)
Galactose-1	103.07	69.72	82.60	68.72	75.18	61.04	-	-	-	-
GlcNAc-2	102.78	55.11	72.22	78.55	74.87	59.95	-	-	-	2.06 – 1.99 (m, 3H)
Galactose-2	103.07	69.72	82.60	68.57	75.18	61.04	-	-	-	-
GlcNAc-6S	102.78	55.11	n/a	n/a	n/a	n/a	-	-	-	2.06 – 1.99 (m, 3H)
Galactose-6S	102.42	69.55	75.14	67.63	n/a	67.06	-	-	-	-
Sialic acid	n/a	n/a	n/a	n/a	n/a	n/a	n/a	n/a	n/a	2.06 – 1.99

										(m, 3H)

Linker	1	2	3	4	5
H	3.91,3.62	1.61 (p, J = 6.8 Hz, 2H)	1.46 – 1.36 (m, 2H)	1.71 – 1.65 (m, 2H)	3.04 – 2.95 (m, 2H)
C	69.90	28.08	22.25	26.41	39.26

HRMS (ESI-MS): m/z calculated for $C_{58}H_{97}N_5O_{45}S_2$ $[M-2H]^{2-}$: 823.7454; found: 823.7531.

Compound S19

S19 was prepared from **14** (1.2 mg, 0.65 μmol) using the general procedure for Cbz deprotection using $Pd(OH)_2$ reduction. After purification, **S19** was obtained as a white solid (0.6 mg, 53%).

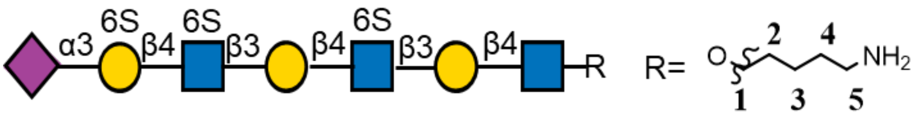

^1H (600 MHz, D_2O): δ (ppm)

	H-1	H-2	H-3	H-4	H-5	H-6	H-7	H-8	H-9	NHAc
GlcNAc	4.53	3.76	3.70	3.70	3.58	3.99, 3.83	-	-	-	2.06 – 2.02 (m, 12H)
Galactose-1	4.47 (d, J = 8.0 Hz, 1H)	3.60	3.73	4.20	n/a	3.77 (4H)	-	-	-	-
GlcNAc-6S-1	4.72	3.84	n/a	n/a	3.84	4.42 (t, *J* = 10.9 Hz, 2H), 4.30 (td, *J* = 10.7, 5.3 Hz, 2H)	-	-	-	2.06 – 2.02 (m, 12H)
Galactose-2	4.51	3.59	3.73	4.21	n/a	3.77 (4H)	-	-	-	-
GlcNAc-6S-2	4.71	3.86	n/a	n/a	3.84	4.42 (t, *J* = 10.9 Hz, 2H), 4.30 (td, *J* = 10.7,	-	-	-	2.06 – 2.02 (m, 12H)

						5.3 Hz, 2H)				
Galactose-6S	4.63 (d, J = 7.8 Hz, 1H)	3.58	4.16	4.03	3.99	4.18 (2H)	-	-	-	-
Sialic acid	-	-	2.76 (dd, J = 12.5, 4.6 Hz, 1H), 1.82 (t, J = 12.1 Hz, 1H)	3.68	3.87	n/a	n/a	n/a	3.91, 3.66	2.06 – 2.02 (m, 12H)

^{13}C (150 MHz, D_2O): δ (ppm)

	C-1	C-2	C-3	C-4	C-5	C-6	C-7	C-8	C-9	NHAc
GlcNAc	101.29	55.25	72.64	78.69	74.43	60.11	-	-	-	22.21
Galactose-1	102.46	69.55	82.44	68.49	n/a	61.16	-	-	-	-
GlcNAc-6S-1	102.91	55.02	n/a	n/a	72.41	66.68	-	-	-	22.21
Galactose-2	102.56	69.55	82.44	68.49	n/a	61.16	-	-	-	-
GlcNAc-6S-2	102.87	55.02	n/a	n/a	72.41	66.68	-	-	-	22.21
Galactose-6S	102.45	69.55	75.45	67.38	72.68	66.82	-	-	-	-
Sialic acid	n/a	n/a	39.60	n/a	51.73	n/a	n/a	n/a	62.50	22.21

Linker	1	2	3	4	5
H	3.91,3.62	1.61 (p, J = 6.7 Hz, 2H)	1.46 – 1.36 (m, 2H)	1.68 (p, J = 7.7 Hz, 2H)	3.02 – 2.96 (m, 2H)
C	69.90	28.08	22.25	26.50	39.30

HRMS (ESI-MS): m/z calculated for $C_{58}H_{96}N_5O_{48}S_3$ $[M-3H]^{3-}$: 575.4801; found: 575.4781.

Compound S20

S20 was prepared from **13** (2.0 mg, 0.93 μmol) using the general procedure for Cbz deprotection using $Pd(OH)_2$ reduction. After purification, **S20** was obtained as a white solid (1.4 mg, 74%).

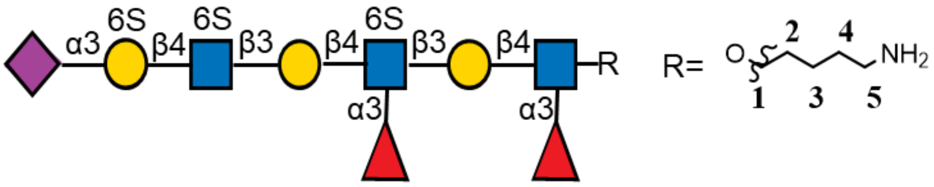

^{1}H (600 MHz, D_2O): δ (ppm)

	H-1	H-2	H-3	H-4	H-5	H-6	H-7	H-8	H-9	NHAc
GlcNAc	4.54 (d, J = 8.2 Hz, 1H)	3.89	n/a	n/a	3.59	4.00, 3.85	-	-	-	2.05 – 2.00 (m, 12H)
Galactose-1	4.45 (d, J = 7.8 Hz, 1H)	3.52	3.71	4.13 (d, J = 3.3 Hz, 1H)	n/a	3.73 (4H)	-	-	-	-
GlcNAc-6S-1	4.74 (d, *J* = 8.4 Hz, 1H)	4.00	3.90	4.00	3.82	4.43 – 4.30 (m, 4H)	-	-	-	2.05 – 2.00 (m, 12H)
Galactose-2	4.51 (d, *J* = 7.9 Hz, 1H)	3.52	3.72	4.15 (d, *J* = 3.1 Hz, 1H)	n/a	3.73 (4H)	-	-	-	-
GlcNAc-6S-2	4.71 (d, *J* = 8.4 Hz, 1H)	3.84	n/a	n/a	3.82	4.43 – 4.30 (m, 4H)	-	-	-	2.05 – 2.00 (m, 12H)
Galactose-6S	4.63 (d, *J* = 7.9 Hz, 1H)	3.57	4.16	4.03	3.99	4.19 (2H)	-	-	-	-
Sialic acid	-	-	2.76 (dd, J = 12.4, 4.6 Hz, 1H), 1.83 (t, J = 12.1 Hz, 1H)	3.70	3.87	n/a	n/a	n/a	3.90, 3.65	2.05 – 2.00 (m, 12H)
Fucose-1	5.10 (d, J = 3.8 Hz, 1H)	3.70	n/a	n/a	4.82	1.18 – 1.14 (m, 6H)	-	-	-	-
Fucose-2	5.13 (d, J =	3.70	n/a	n/a	4.82	1.18 – 1.14 (m, 6H)	-	-	-	-

	4.0 Hz, 1H)									

^{13}C (150 MHz, D_2O): δ (ppm)

	C-1	C-2	C-3	C-4	C-5	C-6	C-7	C-8	C-9	NHAc
GlcNAc	101.07	55.90	n/a	n/a	74.97	59.82	-	-	-	22.20
Galactose-1	101.74	70.46	82.19	68.22	n/a	61.75	-	-	-	-
GlcNAc-6S-1	102.50	55.97	n/a	72.70	72.59	66.51	-	-	-	22.20
Galactose-2	101.46	70.46	82.78	68.22	n/a	61.75	-	-	-	-
GlcNAc-6S-2	102.70	55.22	n/a	n/a	72.59	66.51	-	-	-	22.20
Galactose-6S	102.60	69.08	75.10	67.44	72.65	67.06	-	-	-	-
Sialic acid	n/a	n/a	39.47	n/a	51.60	n/a	n/a	n/a	62.83	22.20
Fucose-1	98.61	n/a	n/a	n/a	66.77	15.33	-	-	-	-
Fucose-2	98.60	n/a	n/a	n/a	66.77	15.33	-	-	-	-

Linker	1	2	3	4	5
H	3.91,3.62	1.60 (p, J = 6.7 Hz, 2H)	1.46 – 1.36 (m, 2H)	1.68 (p, J = 7.7 Hz, 2H)	3.00 (t, J = 8.0 Hz, 2H)
C	70.66	28.08	22.25	26.50	39.23

HRMS (ESI-MS): m/z calculated for $C_{70}H_{116}N_5O_{56}S_3$ $[M-3H]^{3-}$: 672.8520; found: 672.8205.

Compound S21

S21 was prepared from **S20** (0.9 mg, 0.45 μmol) using the general procedure for removal of fucose using fucosidase from *R. gnavus* E1. After P6 purification, **S21** was obtained as a white solid (0.8 mg, quant).

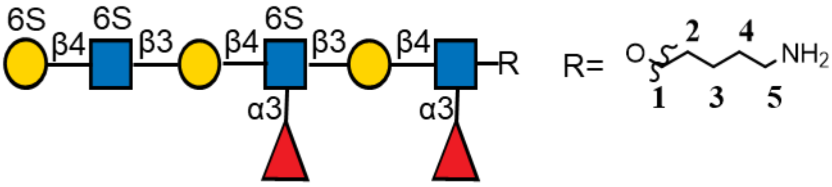

^1H (600 MHz, D_2O): δ (ppm)

	H-1	H-2	H-3	H-4	H-5	H-6	NHAc
GlcNAc	4.53 (d, J = 8.6 Hz, 1H)	3.90	n/a	n/a	3.59	4.00, 3.85	2.07 – 1.99 (m, 9H)
Galactose-1	4.44 (d, J = 7.8 Hz, 1H)	3.52	3.70	4.12 (d, J = 3.2 Hz, 1H)	n/a	3.73 (4H)	-

GlcNAc-6S-1	4.74	4.00	3.90	4.00	3.82	4.41 – 4.36 (m, 2H), 4.33 (d, *J* = 10.8 Hz, 2H)	2.07 – 1.99 (m, 9H)
Galactose-2	4.51 (d, *J* = 7.8 Hz, 1H)	3.52	3.72	4.14	n/a	3.73 (4H)	-
GlcNAc-6S-2	4.71	3.84	n/a	n/a	3.82	4.41 – 4.36 (m, 2H), 4.33 (d, *J* = 10.8 Hz, 2H)	2.07 – 1.99 (m, 9H)
Galactose-6S	4.55 (d, J = 8.0 Hz, 1H)	3.56	3.72	4.00	3.99	4.22 – 4.19 (m, 2H)	-
Fucose-1	5.10 (d, J = 4.0 Hz, 1H)	3.70	n/a	n/a	4.82	1.18 – 1.14 (m, 6H)	-
Fucose-2	5.13 (d, J = 3.8 Hz, 1H)	3.70	n/a	n/a	4.82	1.18 – 1.14 (m, 6H)	-

^{13}C (150 MHz, D_2O): δ (ppm)

	C-1	C-2	C-3	C-4	C-5	C-6	NHAc
GlcNAc	101.07	55.90	n/a	n/a	74.97	59.97	22.20
Galactose-1	101.74	70.58	82.31	68.22	n/a	61.71	-
GlcNAc-6S-1	102.69	55.97	n/a	72.93	72.59	66.35	22.20
Galactose-2	101.56	70.58	82.48	68.22	n/a	61.71	-
GlcNAc-6S-2	102.86	55.36	n/a	n/a	72.59	66.35	22.20
Galactose-6S	103.04	71.58	n/a	68.38	72.84	66.97	-
Fucose-1	98.72	n/a	n/a	n/a	66.83	15.33	-
Fucose-2	98.72	n/a	n/a	n/a	66.83	15.33	-

Linker	1	2	3	4	5
H	3.90, 3.60	1.59 (p, J = 6.9 Hz, 2H)	1.43 – 1.36 (m, 2H)	1.68 (p, J = 7.7 Hz, 2H)	3.02 – 2.96 (m, 2H)
C	70.73	28.18	22.12	26.50	39.23

HRMS (ESI-MS): m/z calculated for $C_{59}H_{100}N_4O_{48}S_3$ $[M-2H]^{2-}$: 864.2340; found: 864.2113.

Compound S22

S22 was prepared from **S21** (0.5 mg, 0.29 μmol) using the general procedure for removal of fucose using fucosidase from *R. gnavus* E1. After P6 purification, **S22** was obtained as a white solid (0.4 mg, quant).

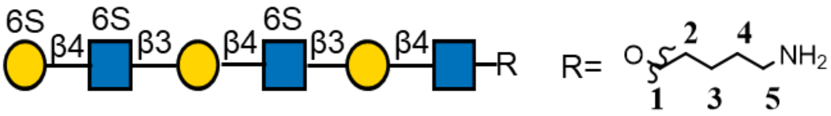

^{1}H (600 MHz, D_2O): δ (ppm)

	H-1	H-2	H-3	H-4	H-5	H-6	NHAc
GlcNAc	4.53	3.74	3.70	3.69	3.59	4.01, 3.83	2.06 – 2.02 (m, 9H)
Galactose-1	4.47 (d, J = 7.8 Hz, 1H)	3.59	3.73	4.20	n/a	3.76 (4H)	-
GlcNAc-6S-1	4.72	4.84	3.77	3.78	3.82	4.43 – 4.39 (m, 2H), 4.34 – 4.26 (m, 2H)	2.06 – 2.02 (m, 9H)
Galactose-2	4.51	3.59	3.73	4.20	n/a	3.76 (4H)	-
GlcNAc-6S-2	4.72	3.84	3.77	3.78	3.82	4.43 – 4.39 (m, 2H), 4.34 – 4.26 (m, 2H)	2.06 – 2.02 (m, 9H)
Galactose-6S	4.55 (d, J = 7.8 Hz, 1H)	3.54	3.72	4.00	3.99	4.21 (2H)	-

^{13}C (150 MHz, D_2O): δ (ppm)

	C-1	C-2	C-3	C-4	C-5	C-6	NHAc
GlcNAc	100.94	54.93	72.43	78.68	74.86	60.10	22.31
Galactose-1	102.82	70.33	82.61	68.92	n/a	61.38	-
GlcNAc-6S-1	102.87	55.07	n/a	n/a	72.59	66.56	22.31
Galactose-2	102.93	70.33	82.61	68.92	n/a	61.38	-
GlcNAc-6S-2	102.87	55.07	n/a	n/a	72.59	66.56	22.31
Galactose-6S	103.03	71.14	n/a	68.40	72.95	66.35	-

Linker	1	2	3	4	5
H	3.91, 3.62	1.61 (p, J = 6.7 Hz, 2H)	1.45 – 1.37 (m, 2H)	1.68 (p, J = 7.6 Hz, 2H)	3.02 – 2.96 (m, 2H)
C	70.20	28.07	22.10	26.52	39.32

HRMS (ESI-MS): m/z calculated for $C_{47}H_{80}N_4O_{40}S_3$ $[M-2H]^{2-}$: 718.1761; found: 718.1498.

Compound S23

S23 was prepared from **40** (1.9 mg, 0.77 μmol) using the general procedure for Cbz deprotection using $Pd(OH)_2$ reduction. After purification, **S23** was obtained as a white solid (1.2 mg, 68%). Proper resolved NMR spectra were obtained in PBS D_2O buffer (40 mM, pH 6.5).

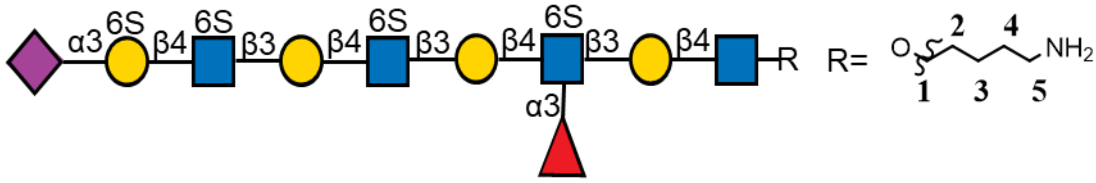

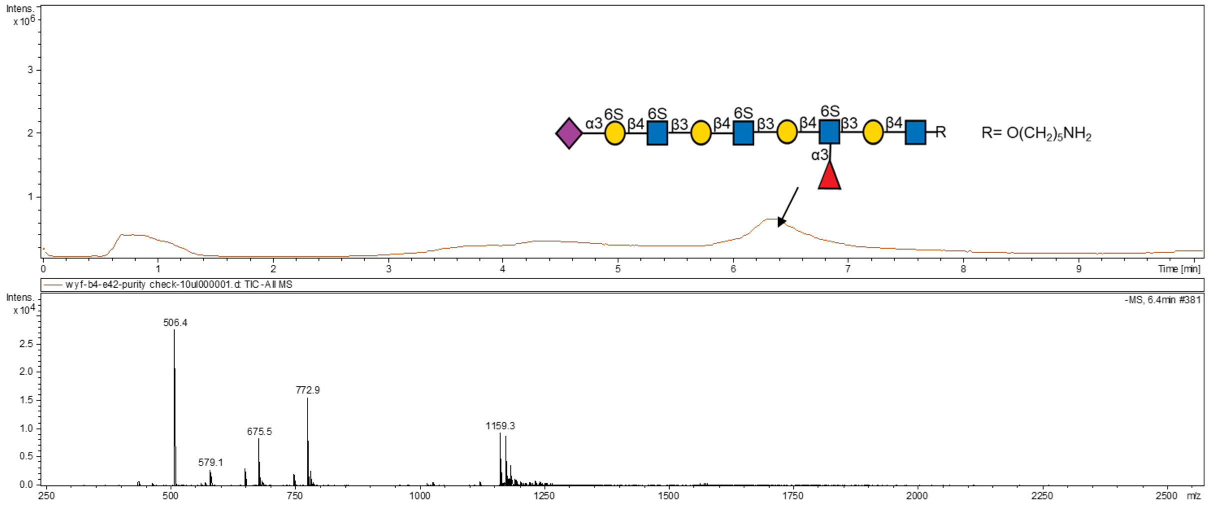

Figure S7a. LC-MS chromatograph of the purified **S23** displaying one peak at 6.4 min showing expected mass of the product.

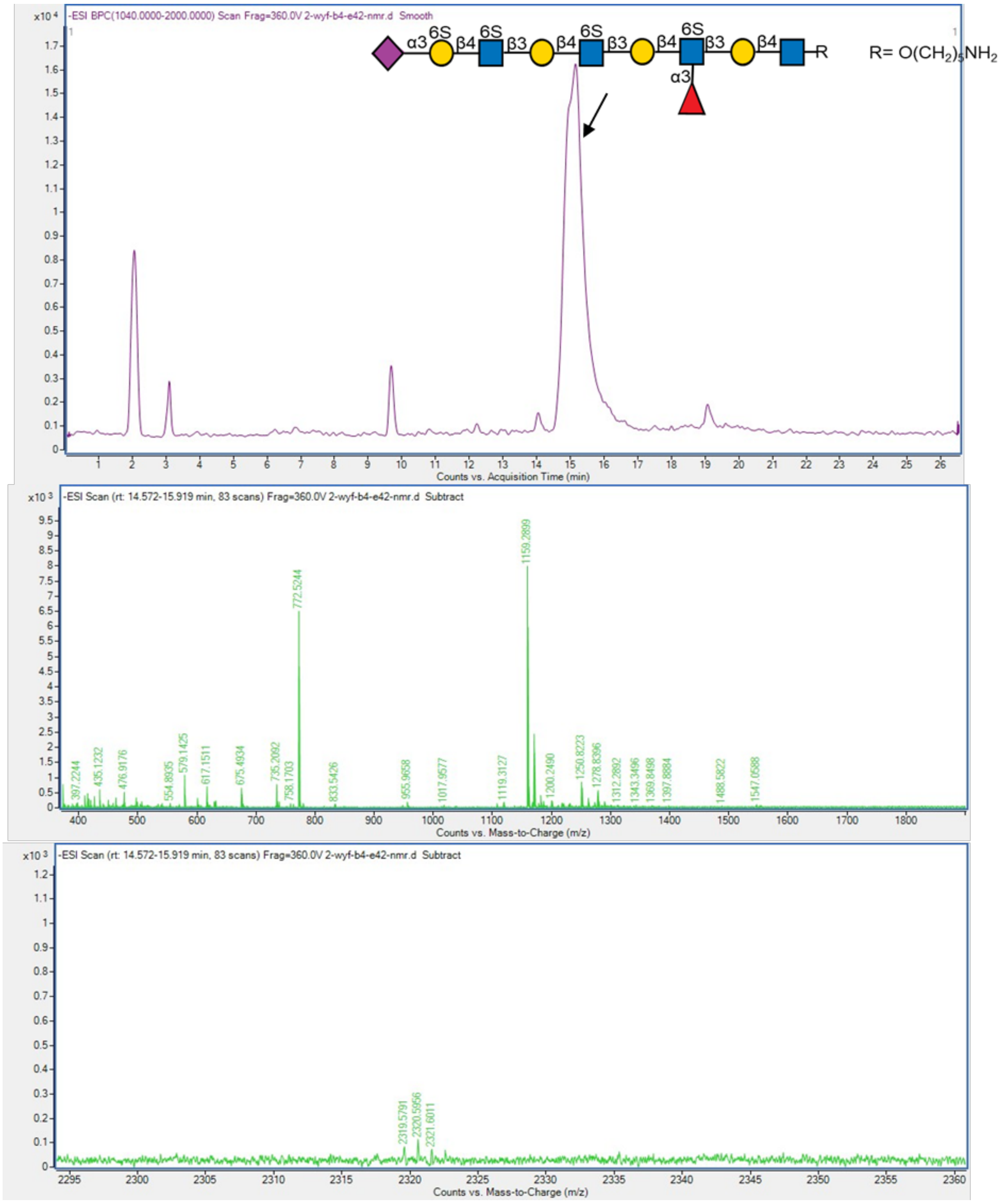

Figure S7b. GC-MS chromatograph of the purified **S23** displaying one peak at 15.4 min showing expected mass of the product.

^{1}H (600 MHz, D_2O): δ (ppm)

	H-1	H-2	H-3	H-4	H-5	H-6	H-7	H-8	H-9	NHAc
GlcNAc	4.52	3.72	3.70	3.69	3.59	3.98, 3.82	-	-	-	2.09 – 1.98 (m, 15H)

Galactose-1	4.46 (d, J = 7.8 Hz, 1H)	3.59	3.72	4.19	n/a	3.76 (4H)	-	-	-	-
GlcNAc-6S-1	4.75	3.99	3.90	4.01	3.82	4.43 – 4.29 (m, 6H)	-	-	-	2.09 – 1.98 (m, 15H)
Galactose-2	4.50	3.52	3.72	4.14	n/a	3.73 (2H)	-	-	-	-
GlcNAc-6S-2	4.71	3.83	3.77	3.78	3.82	4.43 – 4.29 (m, 6H)	-	-	-	2.09 – 1.98 (m, 15H)
Galactose-3	4.53	3.59	3.72	4.20	n/a	3.76 (4H)	-	-	-	-
GlcNAc-6S-3	4.71	3.83	3.77	3.78	3.82	4.43 – 4.29 (m, 6H)	-	-	-	2.09 – 1.98 (m, 15H)
Galactose-6S	4.62 (d, J = 7.2 Hz, 1H)	3.57	4.16	4.03	3.99	4.18 (2H)	-	-	-	-
Fucose	5.12 (d, J = 4.3 Hz, 1H)	3.69	3.92	3.80	4.82	1.16 (d, J = 6.7 Hz, 3H)	-	-	-	-
Sialic acid	-	-	2.75 (dd, J = 12.5, 4.9 Hz, 1H), 1.82 (t, J = 12.0 Hz, 1H)	3.70	3.87	n/a	n/a	3.92	3.90, 3.65	2.09 – 1.98 (m, 15H)

^{13}C (150 MHz, D_2O): δ (ppm)

	C-1	C-2	C-3	C-4	C-5	C-6	C-7	C-8	C-9	NHAc
GlcNAc	100.93	55.18	72.66	78.70	74.91	60.14	-	-	-	22.27
Galactose-1	102.77	69.63	82.63	69.01	n/a	61.21	-	-	-	-
GlcNAc-6S-1	102.70	56.26	74.82	72.94	72.61	66.47	-	-	-	22.27
Galactose-2	101.82	70.46	82.63	68.46	n/a	61.49	-	-	-	-
GlcNAc-6S-2	102.84	55.14	n/a	n/a	72.61	66.47	-	-	-	22.27
Galactose-3	102.50	69.63	82.63	69.01	n/a	61.21	-	-	-	-
GlcNAc-6S-3	102.84	55.14	n/a	n/a	72.61	66.47	-	-	-	22.27
Galactose-6S	102.70	69.84	75.22	67.37	72.78	66.58	-	-	-	-
Fucose	98.62	n/a	68.67	n/a	66.71	15.36	-	-	-	-

Sialic acid	n/a	n/a	39.65	n/a	51.72	n/a	n/a	72.04	62.54	22.27

Linker	1	2	3	4	5
H	3.91, 3.62	1.60 (q, *J* = 6.8 Hz, 2H)	1.46 – 1.36 (m, 2H)	1.68 (p, *J* = 7.6 Hz, 2H)	2.99 (t, *J* = 7.6 Hz, 2H)
C	70.36	28.13	22.18	26.35	39.21

HRMS (ESI-MS): m/z calculated for $C_{78}H_{128}N_6O_{65}S_4$ $[M-4H]^{4-}$: 579.1450; found: 579.1293.

Compound S24

S24 was prepared from **S23** (0.8 mg, 0.34 μmol) using the general procedure for the removal of sialic acid using *C. perfringens* neuraminidase. After P6 purification, **S24** was obtained as a white solid (0.6 mg, 86%).

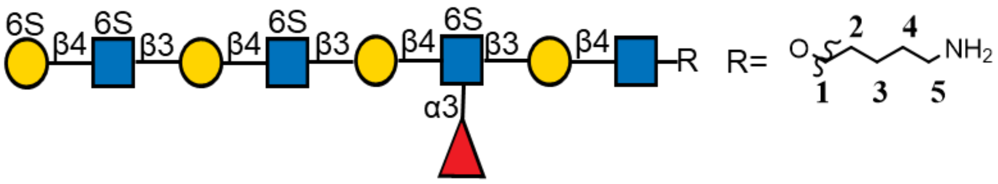

^1H (600 MHz, D_2O): δ (ppm)

	H-1	H-2	H-3	H-4	H-5	H-6	H-7	H-8	H-9	NHAc
GlcNAc	4.53 (d, *J* = 7.7 Hz, 2H)	3.73	3.70	3.70	3.60	3.99, 3.83	-	-	-	2.07 – 2.00 (m, 12H)
Galactose-1	4.47 (d, J = 7.8 Hz, 1H)	3.60	3.73	4.20	n/a	3.76 (4H)	-	-	-	-
GlcNAc-6S-1	4.75	3.98	3.90	4.01	3.83	4.43 – 4.30 (m, 6H)	-	-	-	2.07 – 2.00 (m, 12H)
Galactose-2	4.51 (d, J = 7.9 Hz, 1H)	3.52	3.72	4.14 (d, J = 3.6 Hz, 1H)	n/a	3.73 (2H)	-	-	-	-
GlcNAc-6S-2	4.72	3.84	3.77	3.78	3.83	4.43 – 4.30 (m, 6H)	-	-	-	2.07 – 2.00 (m, 12H)
Galactose-3	4.53 (d, *J* = 7.7 Hz, 2H)	3.59	3.73	4.20	n/a	3.76 (4H)	-	-	-	-
GlcNAc-6S-3	4.72	3.84	3.77	3.78	3.83	4.43 – 4.30 (m, 6H)	-	-	-	2.07 – 2.00

										(m, 12H)
Galactose-6S	4.55 (d, J = 7.8 Hz, 1H)	3.56	3.72	4.00	3.99	4.21 (2H)	-	-	-	-
Fucose	5.12 (d, J = 4.1 Hz, 1H)	3.69	3.93	3.81	4.83	1.16 (d, J = 6.5 Hz, 3H)	-	-	-	-

^{13}C (150 MHz, D_2O): δ (ppm)

	C-1	C-2	C-3	C-4	C-5	C-6	C-7	C-8	C-9	NHAc
GlcNAc	101.08	55.03	72.36	78.72	74.91	60.12	-	-	-	22.23
Galactose-1	102.84	70.01	82.54	68.72	n/a	61.10	-	-	-	-
GlcNAc-6S-1	102.60	55.85	74.54	72.83	72.53	66.36	-	-	-	22.23
Galactose-2	102.17	70.50	82.54	68.14	n/a	61.52	-	-	-	-
GlcNAc-6S-2	102.76	55.08	n/a	n/a	72.53	66.36	-	-	-	22.23
Galactose-3	102.76	70.01	82.54	68.72	n/a	61.10	-	-	-	-
GlcNAc-6S-3	102.76	55.08	n/a	n/a	72.53	66.36	-	-	-	22.23
Galactose-6S	103.18	71.40	n/a	68.46	72.73	66.31	-	-	-	-
Fucose	98.54	n/a	68.52	n/a	66.77	15.33	-	-	-	-

Linker	1	2	3	4	5
H	3.91, 3.63	1.61 (p, J = 6.6 Hz, 2H)	1.43 – 1.37 (m, 2H)	1.68 (p, J = 7.7 Hz, 2H)	3.02 – 2.97 (m, 2H)
C	70.39	28.16	22.12	26.50	39.23

HRMS (ESI-MS): m/z calculated for $C_{67}H_{113}N_5O_{57}S_4$ $[M-2H]^{2-}$: 1013.7495; found: 1013.7217.

Compound S25

S25 was prepared from **43** (0.7 mg, 0.34 μmol) using the general procedure for Cbz deprotection using $Pd(OH)_2$ reduction. After purification, **S25** was obtained as a white solid (0.4 mg, 60%).

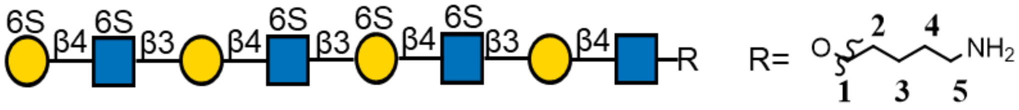

^{1}H (600 MHz, D_2O): δ (ppm)

	H-1	H-2	H-3	H-4	H-5	H-6	NHAc
GlcNAc	4.53	3.72	3.70	3.69	3.60	4.00，3.82	2.06 – 2.01 (m, 12H)
Galactose-1	4.47 (d, J = 7.7 Hz, 1H)	3.60	3.73	4.20	n/a	3.76 (4H)	-
GlcNAc-6S-1	4.72	3.83	3.77	3.78	3.81	4.44 – 4.27 (m, 6H)	2.06 – 2.01 (m, 12H)
Galactose-6S-1	4.54	3.60	3.73	4.24	3.99	4.20 (4H)	-
GlcNAc-6S-2	4.72	3.83	3.77	3.78	3.81	4.44 – 4.27 (m, 6H)	2.06 – 2.01 (m, 12H)
Galactose-2	4.52	3.60	3.73	4.20	n/a	3.76 (4H)	-
GlcNAc-6S-3	4.72	3.83	3.77	3.78	3.81	4.44 – 4.27 (m, 6H)	2.06 – 2.01 (m, 12H)
Galactose-6S-2	4.55	3.56	3.72	4.01	3.99	4.20 (4H)	-

^{13}C (150 MHz, D_2O): δ (ppm)

	C-1	C-2	C-3	C-4	C-5	C-6	NHAc
GlcNAc	100.97	55.11	72.42	78.81	75.10	59.93	22.18
Galactose-1	102.62	70.02	82.52	68.84	n/a	61.17	-
GlcNAc-6S-1	102.77	55.16	n/a	n/a	72.27	66.54	22.18
Galactose-6S-1	102.56	70.02	82.52	68.58	72.86	66.46	-
GlcNAc-6S-2	102.77	55.16	n/a	n/a	72.27	66.54	22.18
Galactose-2	102.28	70.02	82.52	68.84	n/a	61.17	-
GlcNAc-6S-3	102.77	55.16	n/a	n/a	72.27	66.54	22.18
Galactose-6S-2	103.14	70.84	n/a	68.16	72.86	66.46	-

Linker	1	2	3	4	5
H	3.91,3.61	1.64 – 1.57 (m, 2H)	1.45 – 1.38 (m, 2H)	1.68 (p, *J* = 7.7 Hz, 2H)	3.02 – 2.96 (m, 2H)
C	70.23	28.07	22.24	26.37	39.30

HRMS (ESI-MS): m/z calculated for $C_{61}H_{103}N_5O_{56}S_5$ $[M-2H]^{2-}$: 980.6990; found: 980.6858.

Compound S26

S26 was prepared from **26** (0.6 mg, 0.29 μmol) using the general procedure for Cbz deprotection using $Pd(OH)_2$ reduction. After purification, **S26** was obtained as a white solid (0.3 mg, 57%).

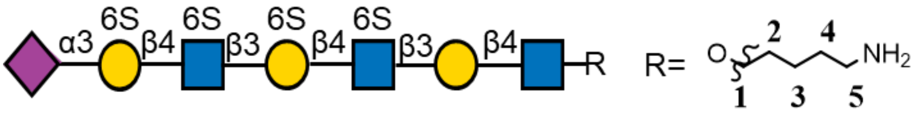

^{1}H (600 MHz, D_2O): δ (ppm)

	H-1	H-2	H-3	H-4	H-5	H-6	H-7	H-8	H-9	NHAc
GlcNAc	4.53	3.72	3.70	3.69	3.58	3.99, 3.83	-	-	-	2.09 – 1.96 (m, 12H)
Galactose	4.47 (d, *J* = 8.0 Hz, 1H)	3.60	3.71	4.20	n/a	3.76 (2H)	-	-	-	-
GlcNAc-6S-1	4.73	3.83	n/a	n/a	3.84	4.42, 4.34	-	-	-	2.09 – 1.96 (m, 12H)
Galactose-6S-1	4.54	3.59	3.75	4.24	n/a	4.19 (4H)	-	-	-	-
GlcNAc-6S -2	4.72	3.83	n/a	n/a	3.84	4.42, 4.28	-	-	-	2.09 – 1.96 (m, 12H)
Galactose-6S-2	4.64 (d, *J* = 7.9 Hz, 1H)	3.58	4.16	4.03	3.99	4.19 (4H)	-	-	-	-
Sialic acid	-	-	2.75 (dd, J = 12.3, 4.7 Hz, 1H), 1.83 (t, J = 12.2 Hz, 1H)	3.70	3.86	n/a	n/a	3.91	3.90, 3.65	2.09 – 1.96 (m, 12H)

^{13}C (150 MHz, D_2O): δ (ppm)

	C-1	C-2	C-3	C-4	C-5	C-6	C-7	C-8	C-9	NHAc
GlcNAc	101.05	55.01	72.64	78.59	74.79	60.30	-	-	-	22.28
Galactose	102.91	70.57	82.63	69.40	n/a	61.18	-	-	-	-
GlcNAc-6S-1	102.76	55.16	n/a	n/a	72.46	66.78	-	-	-	22.28
Galactose-6S-1	102.56	70.20	82.54	68.66	n/a	67.22	-	-	-	-
GlcNAc-6S 6S-2	102.76	55.16	n/a	n/a	72.46	66.78	-	-	-	22.28

Galactose-6S-2	102.62	69.57	75.17	67.40	72.55	67.22	-	-	-	-
Sialic acid	n/a	n/a	39.52	n/a	51.82	n/a	n/a	71.92	62.63	22.28

Linker	1	2	3	4	5
H	3.90, 3.72	1.60 (q, J = 6.9 Hz, 2H)	1.46 – 1.36 (m, 2H)	1.68 (p, J = 7.7 Hz, 2H)	3.00 (t, J = 7.7 Hz, 2H)
C	69.92	28.17	22.42	26.49	39.19

HRMS (ESI-MS): m/z calculated for $C_{58}H_{97}N_5O_{51}S_4$ $[M-2H]^{2-}$: 903.7022; found: 903.6910.

6) References

1. Peng, W. J.; Pranskevich, J.; Nycholat, C.; Gilbert, M.; Wakarchuk, W.; Paulson, J. C.; Razi, N., *Helicobacter pylori* beta 1,3-*N*-acetylglucosaminyltransferase for versatile synthesis of type 1 and type 2 poly-LacNAcs on *N*-linked, *O*-linked and I-antigen glycans. *Glycobiology* **2012**, *22* (11), 1453-1464.

2. Prudden, A. R.; Liu, L.; Capicciotti, C. J.; Wolfert, M. A.; Wang, S.; Gao, Z.; Meng, L.; Moremen, K. W.; Boons, G. J., Synthesis of asymmetrical multiantennary human milk oligosaccharides. *Proc. Natl. Acad. Sci. U. S. A.* **2017**, *114* (27), 6954-6959.

3. Li, Y.; Xue, M.; Sheng, X.; Yu, H.; Zeng, J.; Thon, V.; Chen, Y.; Muthana, M. M.; Wang, P. G.; Chen, X., Donor substrate promiscuity of bacterial β1–3-N-acetylglucosaminyltransferases and acceptor substrate flexibility of β1–4-galactosyltransferases. *Biorg. Med. Chem.* **2016**, *24* (8), 1696-1705.

4. Yu, H.; Huang, S.; Chokhawala, H.; Sun, M.; Zheng, H.; Chen, X., Highly efficient chemoenzymatic synthesis of naturally occurring and non-natural α-2, 6-linked sialosides: A *P. damsela* α-2, 6-sialyltransferase with extremely flexible donor–substrate specificity. *Angew. Chem. Int. Ed.* **2006**, *45* (24), 3938-3944.

5. Sugiarto, G.; Lau, K.; Qu, J.; Li, Y.; Lim, S.; Mu, S.; Ames, J. B.; Fisher, A. J.; Chen, X., A sialyltransferase mutant with decreased donor hydrolysis and reduced sialidase activities for directly sialylating Lewisx. *ACS Chem. Biol.* **2012**, *7* (7), 1232-1240.

6. Wu, Y.; Vos, G. M.; Huang, C.; Chapla, D.; Kimpel, A. L. M.; Moremen, K. W.; de Vries, R. P.; Boons, G.-J., Exploiting substrate specificities of 6-O-sulfotransferases to enzymatically synthesize keratan sulfate oligosaccharides. *JACS Au* **2023**, *3* (11), 3155-3164.

7. Wang, W.; Hu, T.; Frantom, P. A.; Zheng, T.; Gerwe, B.; Del Amo, D. S.; Garret, S.; Seidel III, R. D.; Wu, P., Chemoenzymatic synthesis of GDP-L-fucose and the Lewis X glycan derivatives. *Proc. Natl. Acad. Sci. U. S. A.* **2009**, *106* (38), 16096-16101.

8. Meng, L.; Forouhar, F.; Thieker, D.; Gao, Z.; Ramiah, A.; Moniz, H.; Xiang, Y.; Seetharaman, J.; Milaninia, S.; Su, M., Enzymatic basis for N-glycan sialylation: structure of rat α2, 6-sialyltransferase (ST6GAL1) reveals conserved and unique features for glycan sialylation. *J. Biol. Chem.* **2013,** *288* (48), 34680-34698.

9. Moremen, K. W.; Ramiah, A.; Stuart, M.; Steel, J.; Meng, L.; Forouhar, F.; Moniz, H. A.; Gahlay, G.; Gao, Z.; Chapla, D., ; Wang, S.; Yang, J. Y.; Prabhakar, P. K.; Johnson, R.; Rosa, M. D.; Geisler, C.; Nairn, A. V.; Seetharaman, J.; Wu, S. C.; Tong, L.; Gilbert, H. J.; LaBaer, J.; Jarvis, D. L. Expression system for structural and functional studies of human glycosylation enzymes. *Nat. Chem. Biol.* **2018**, *14* (2), 156-162.

10. Wu, H.; Rebello, O.; Crost, E. H.; Owen, C. D.; Walpole, S.; Bennati-Granier, C.; Ndeh, D.; Monaco, S.; Hicks, T.; Colvile, A., Fucosidases from the human gut symbiont *Ruminococcus gnavus*. *Cell. Mol. Life Sci.* **2021**, *78*, 675-693.

7) NMR spectra

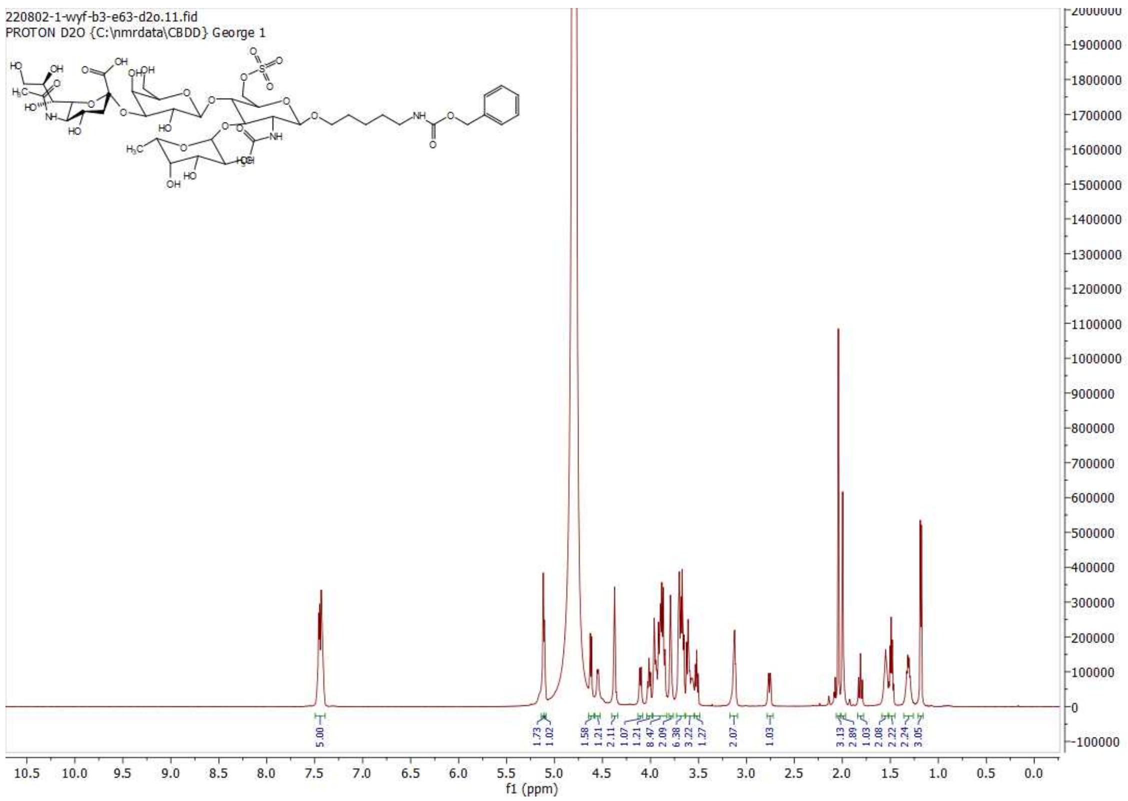

^{1}H NMR of S1; 600MHz; D_2O

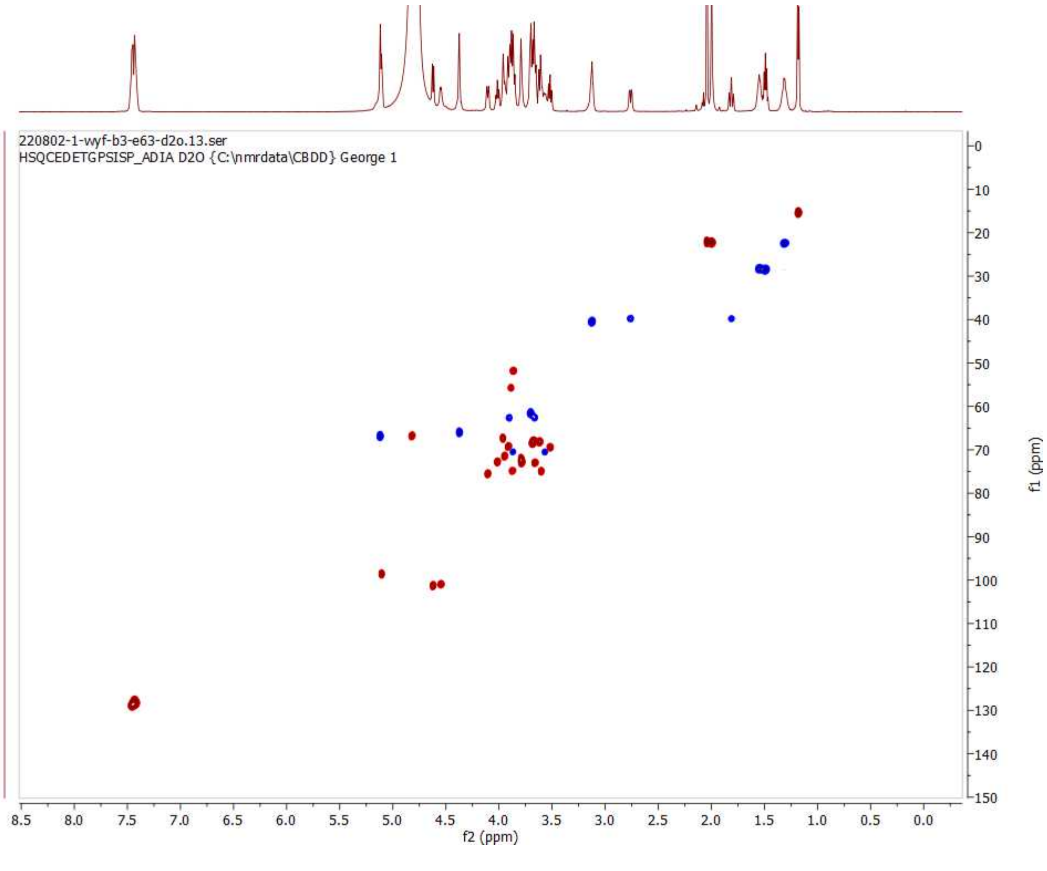

HSQC of S1; 600 MHz/150 MHz, D_2O

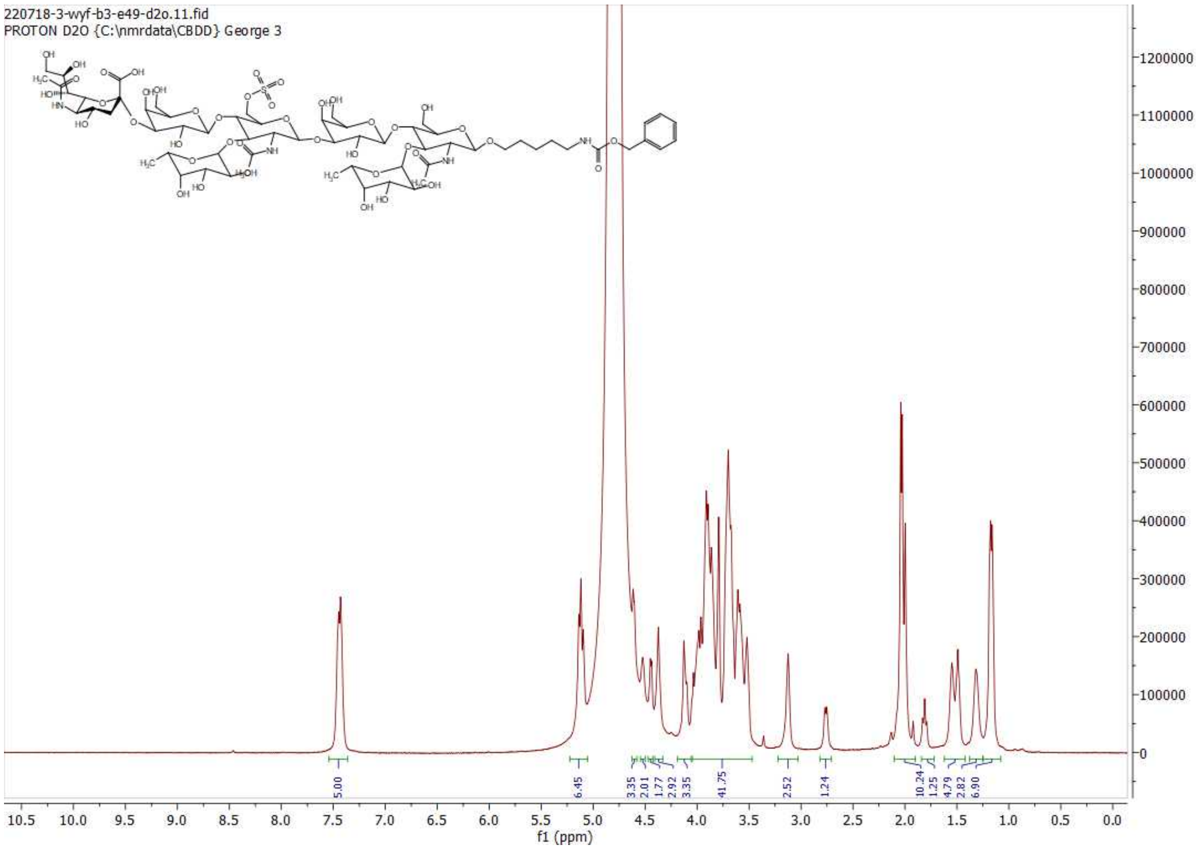

^{1}H NMR of S2; 600MHz; D_2O

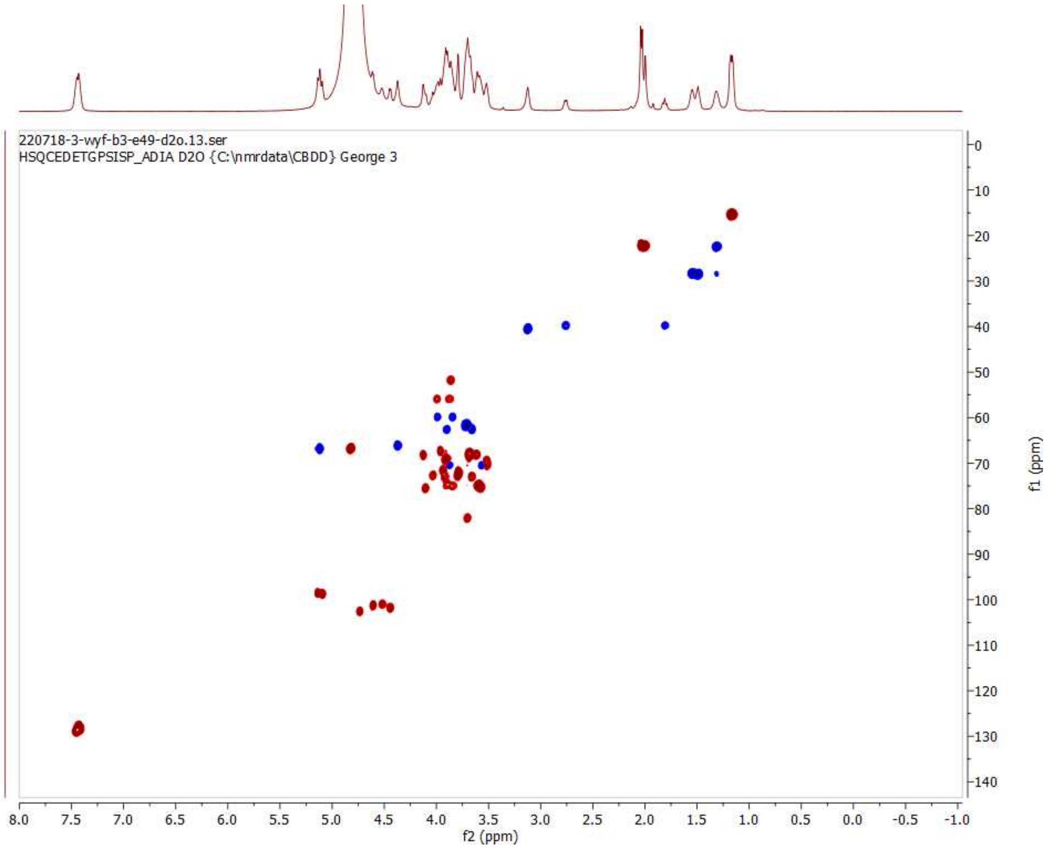

HSQC of S2; 600 MHz/150 MHz, D_2O

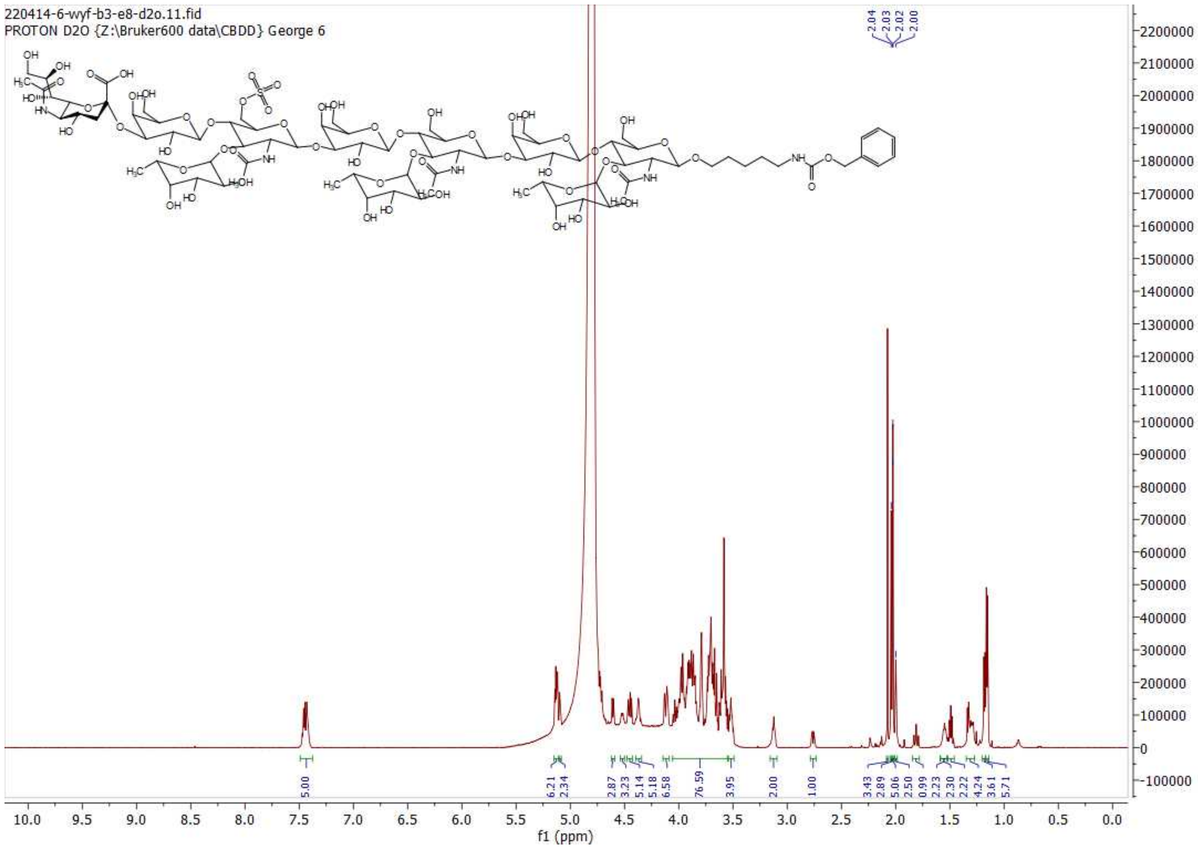

^{1}H NMR of S3; 600MHz; D_2O

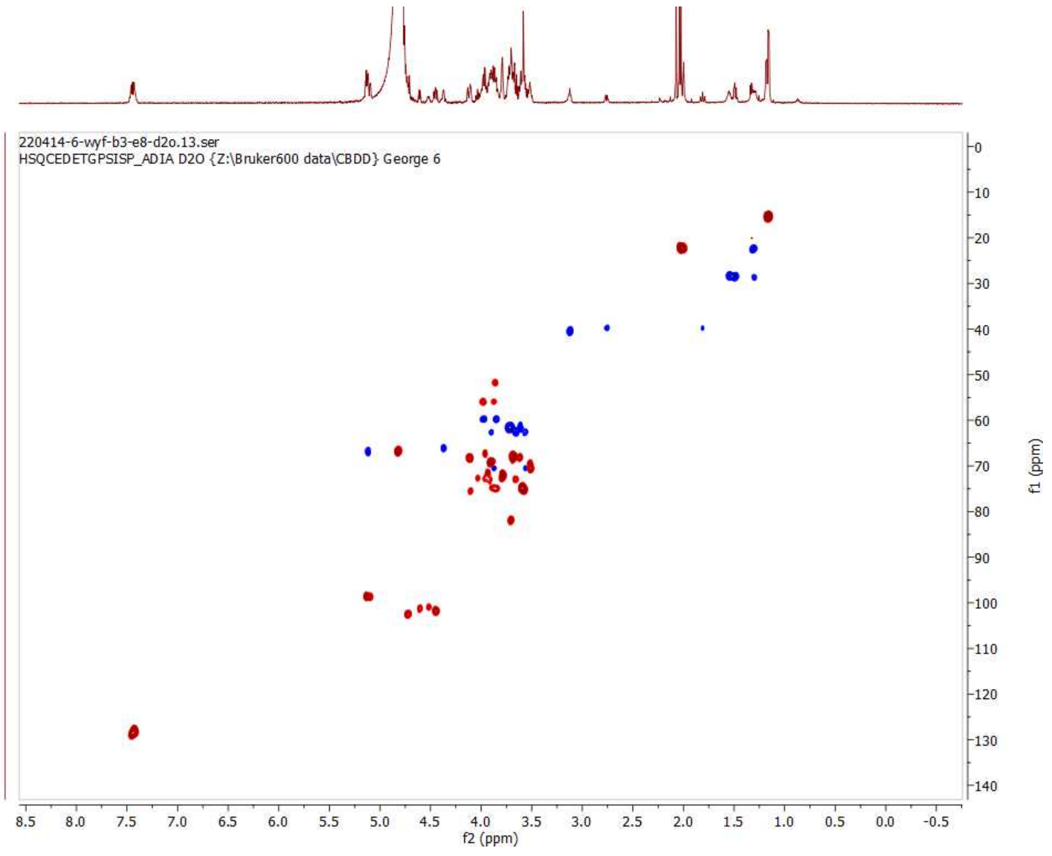

HSQC of S3; 600 MHz/150 MHz, D_2O

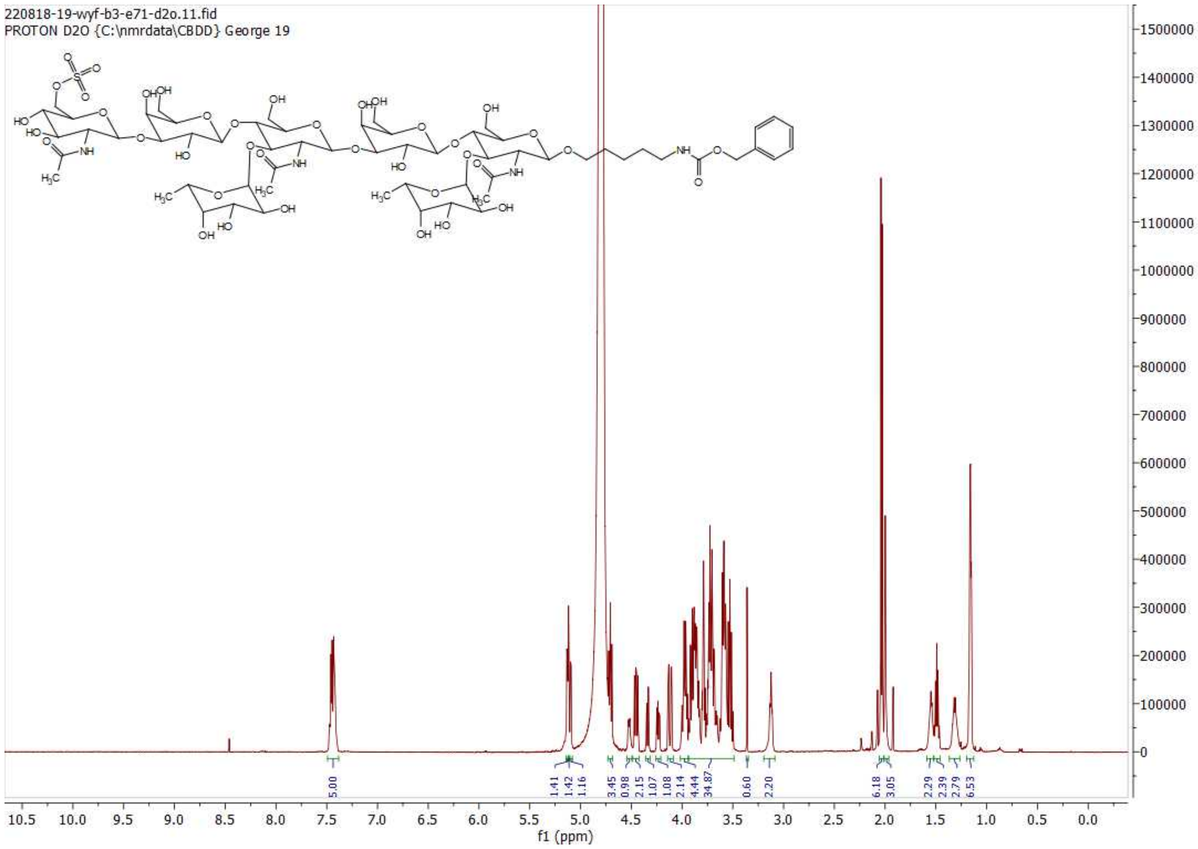

^{1}H NMR of 3; 600MHz; D$_2$O

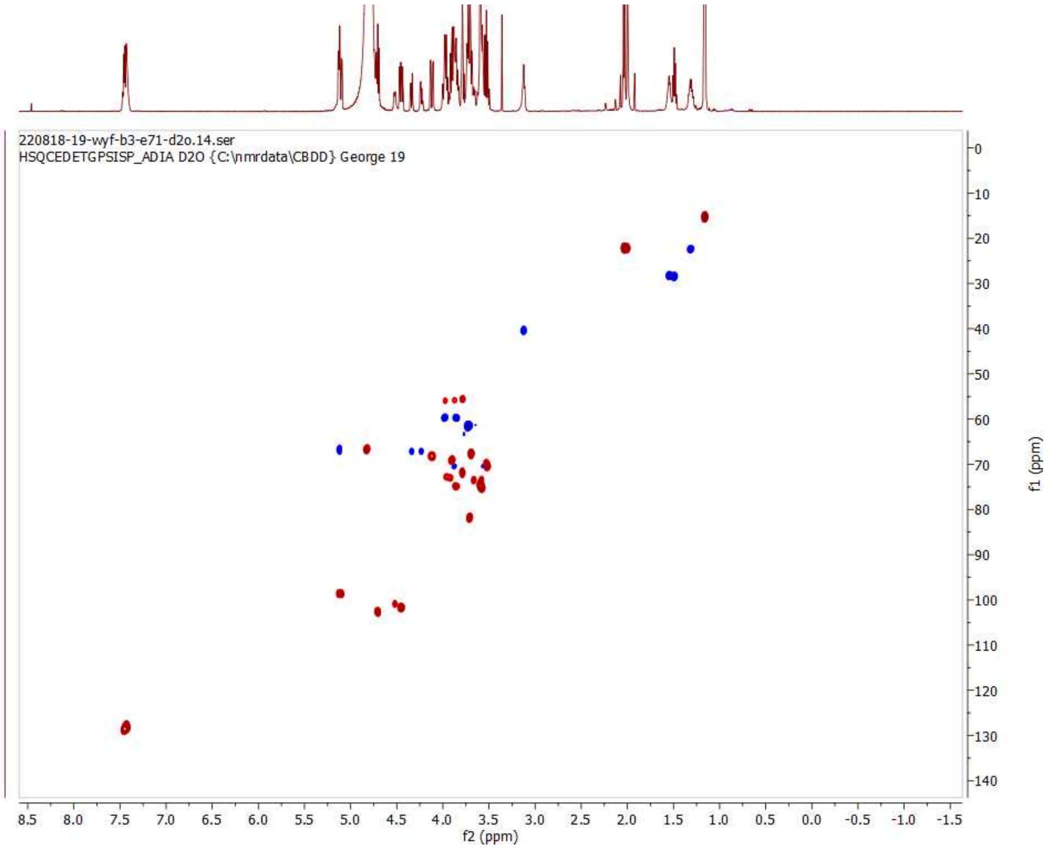

HSQC of 3; 600 MHz/150 MHz, D$_2$O

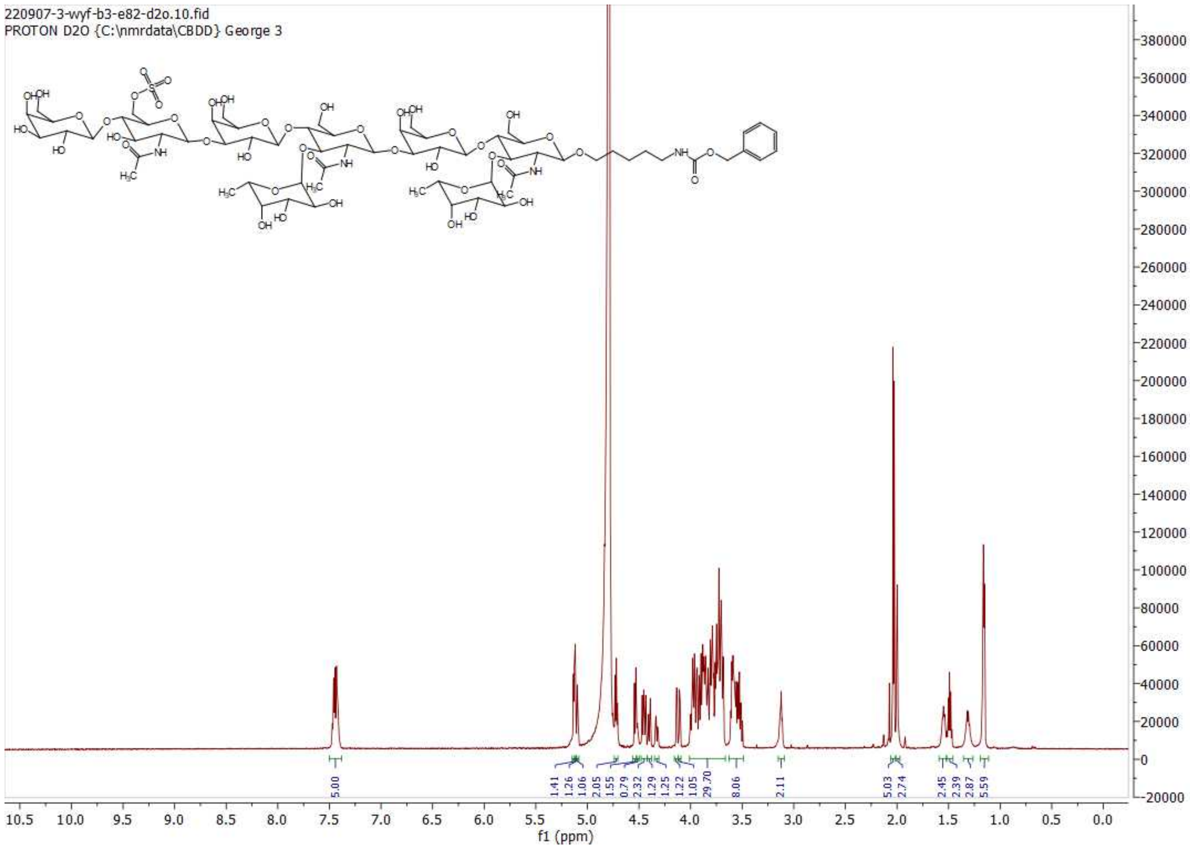

^{1}H NMR of 4; 600MHz; D_2O

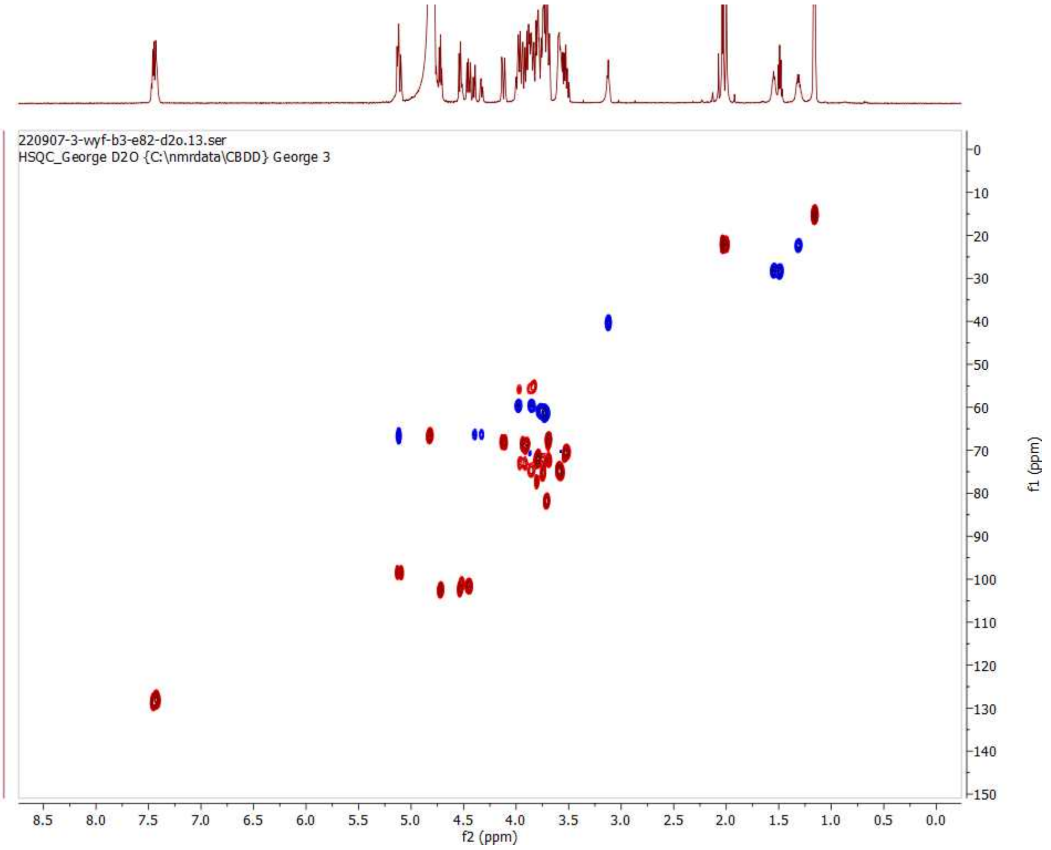

HSQC of 4; 600 MHz/150 MHz, D_2O

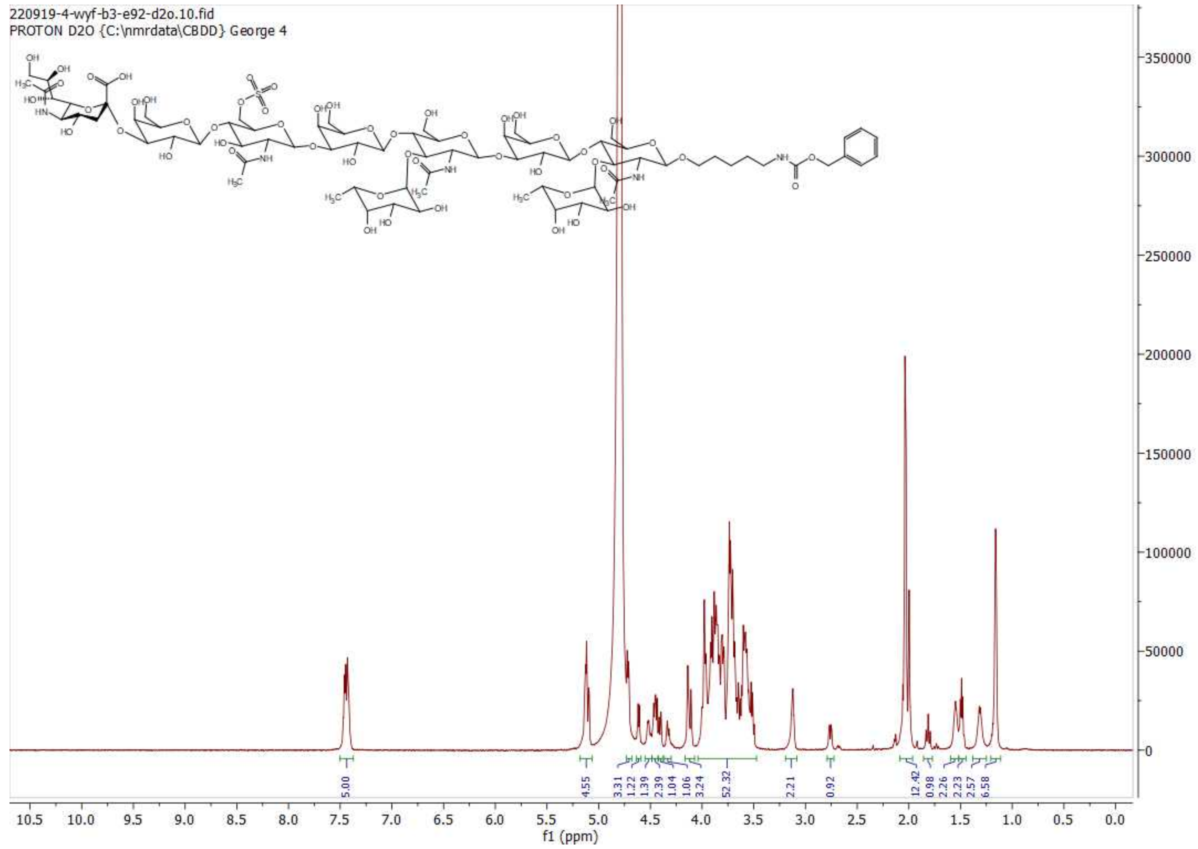

^{1}H NMR of 5; 600MHz; D$_2$O

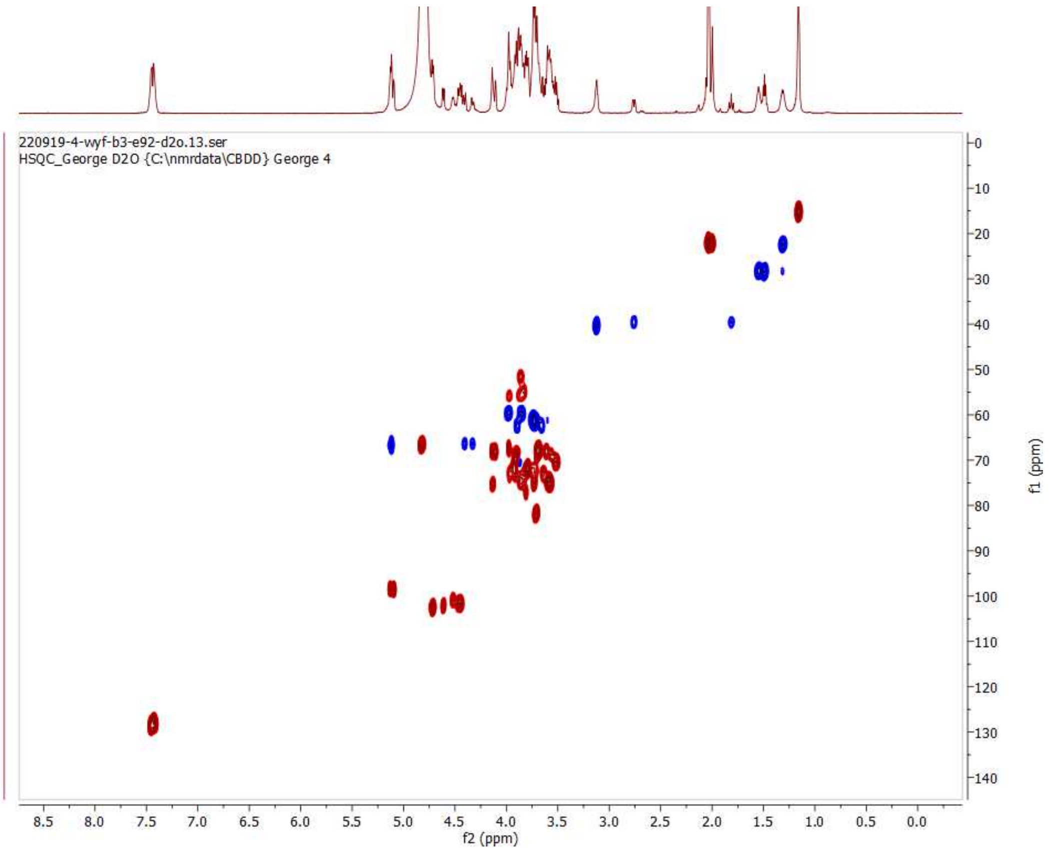

HSQC of 5; 600 MHz/150 MHz, D$_2$O

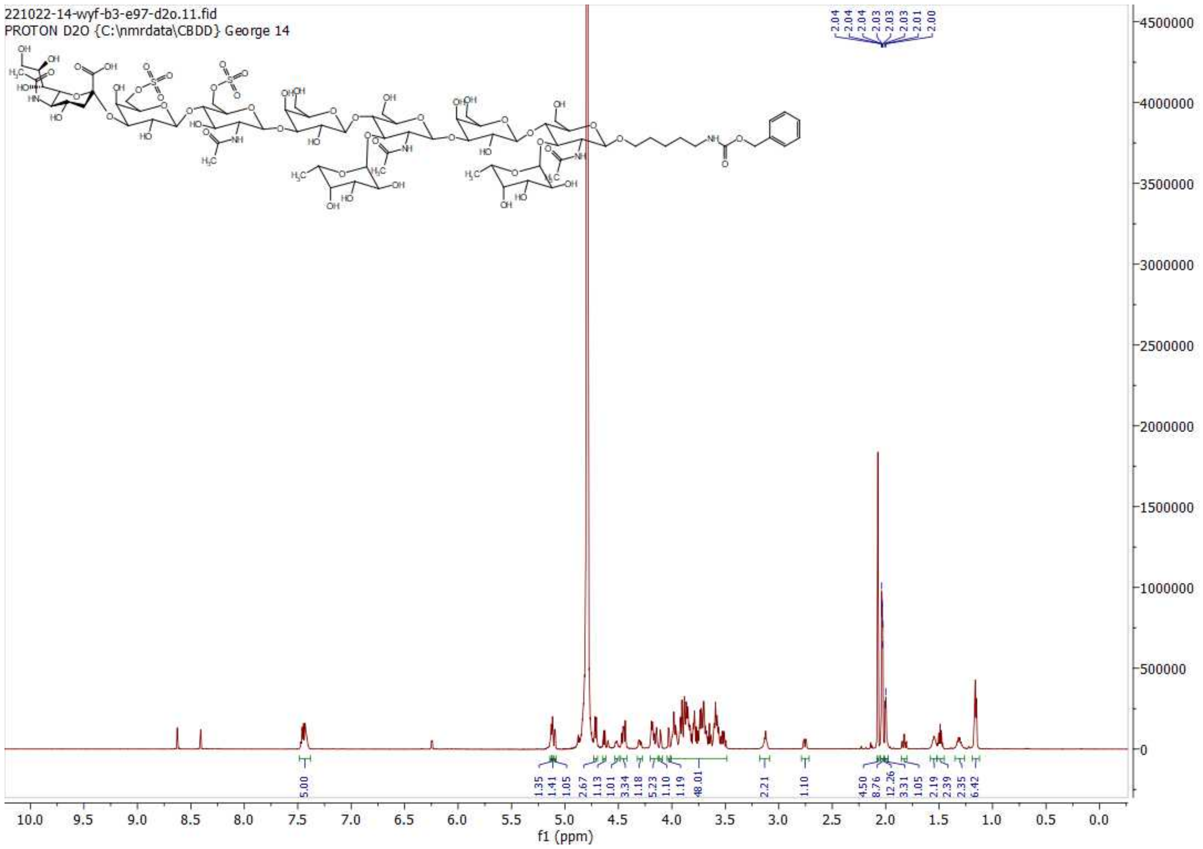

^{1}H NMR of 6; 600MHz; D$_2$O

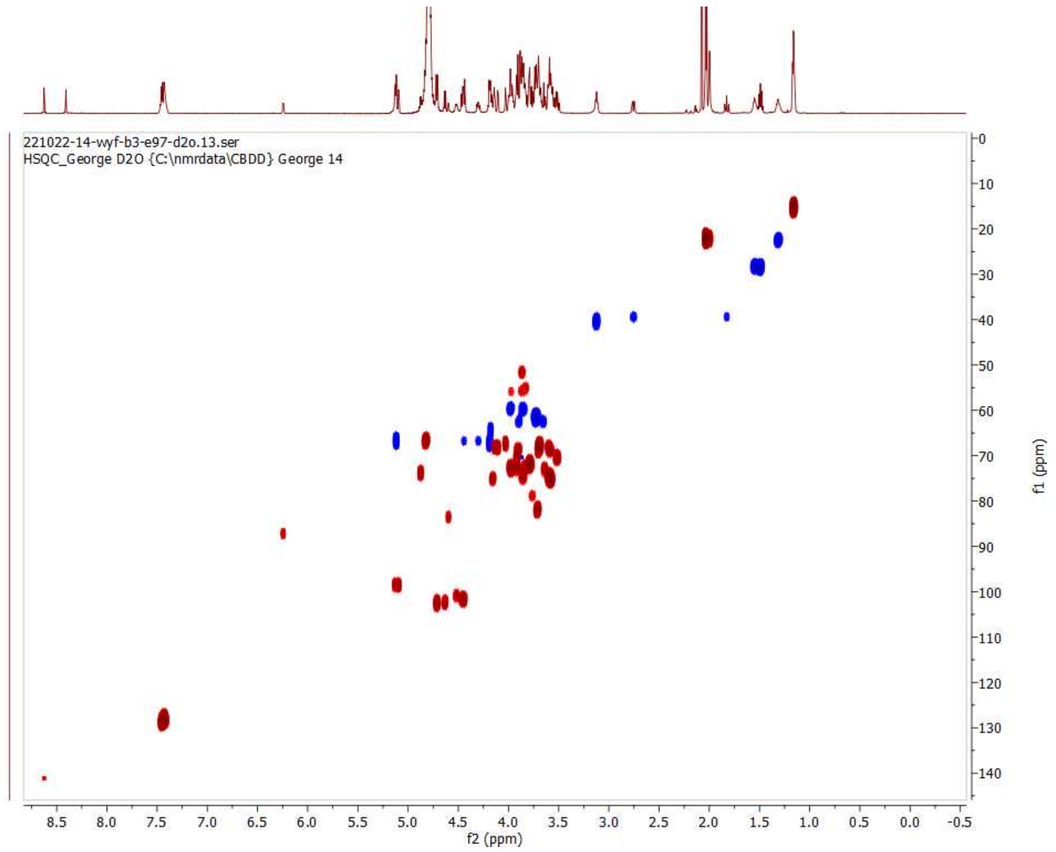

HSQC of 6; 600 MHz/150 MHz, D$_2$O

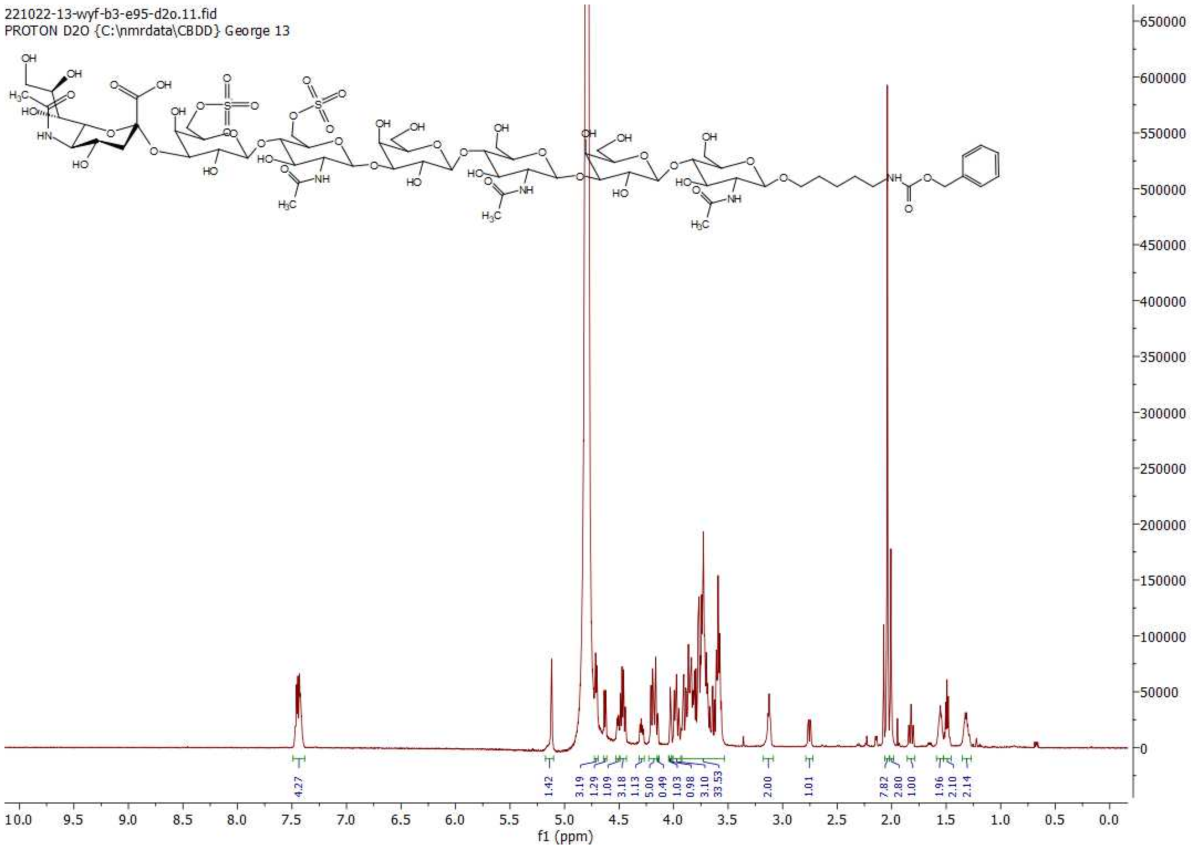

^{1}H NMR of 7; 600MHz; D_2O

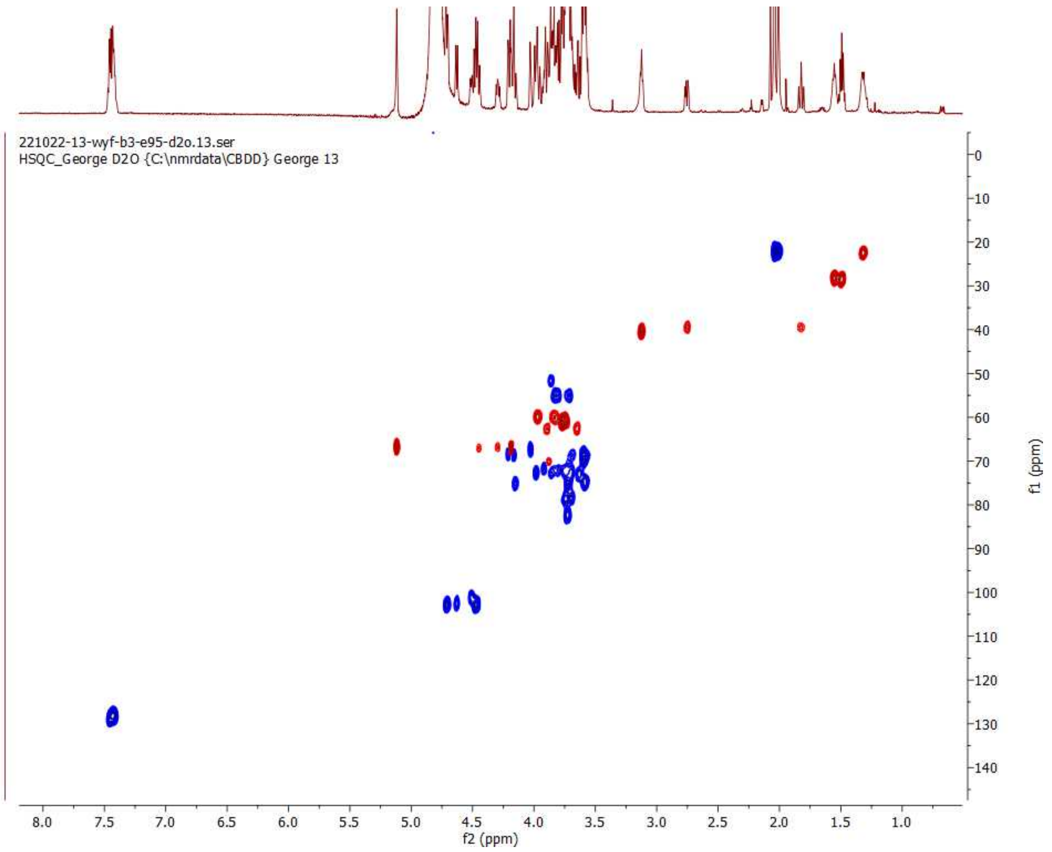

HSQC of 7; 600 MHz/150 MHz, D_2O

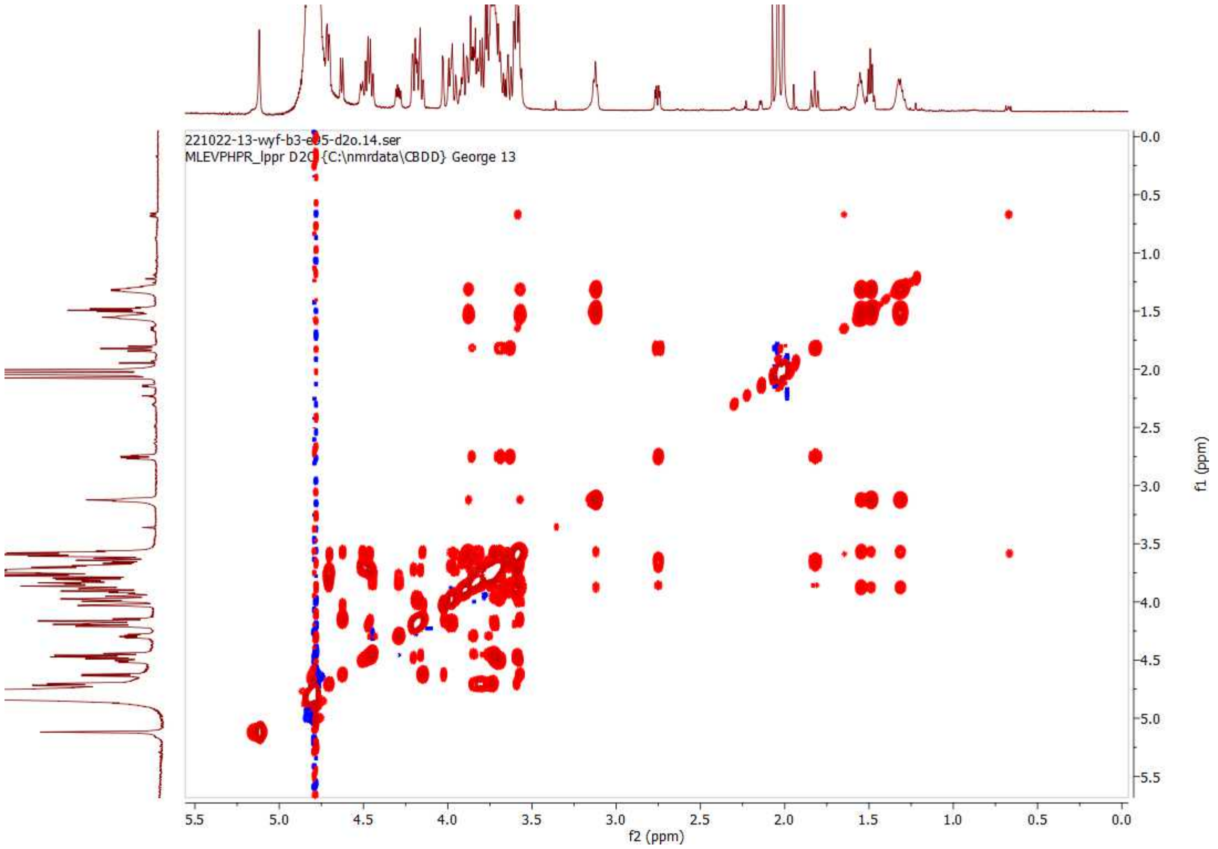

TOCSY (80 ms) of 7; 600MHz; D_2O

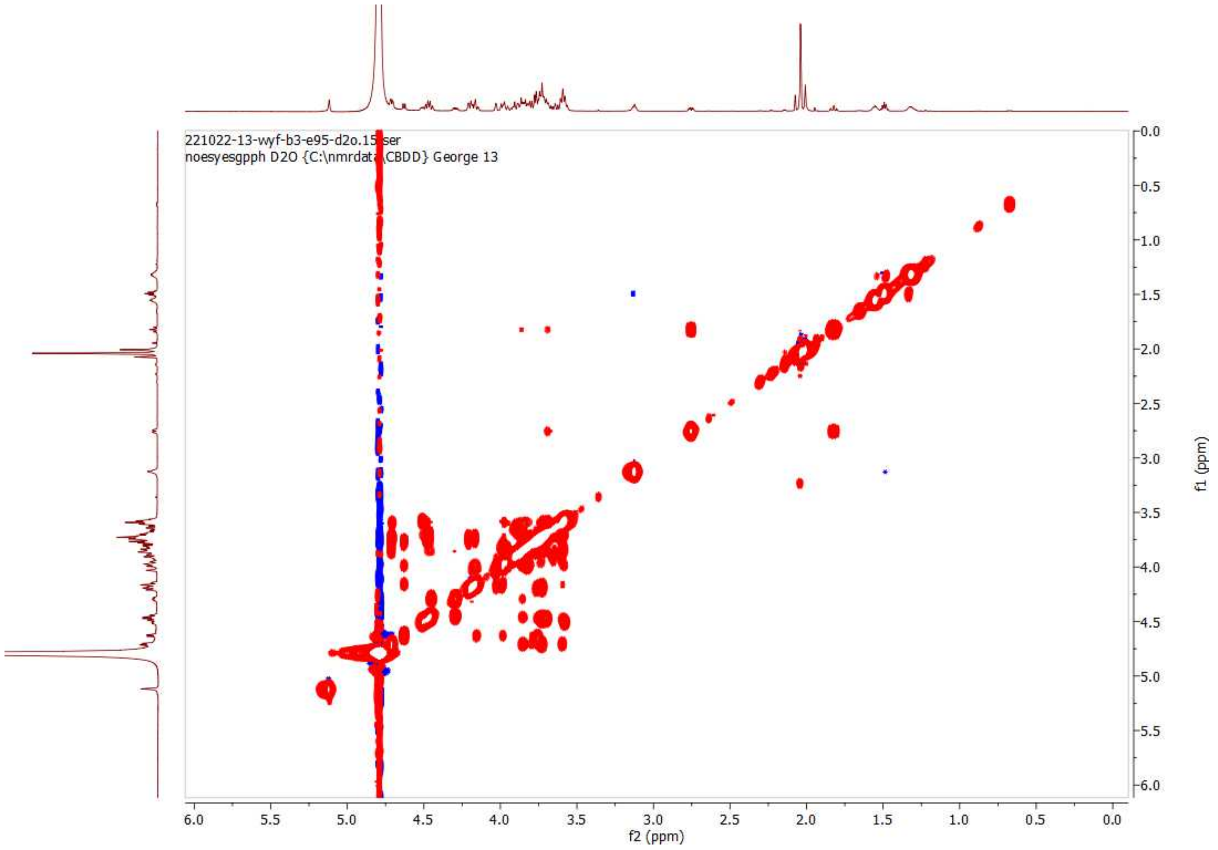

NOESY (300 ms) of 7; 600 MHz, D_2O

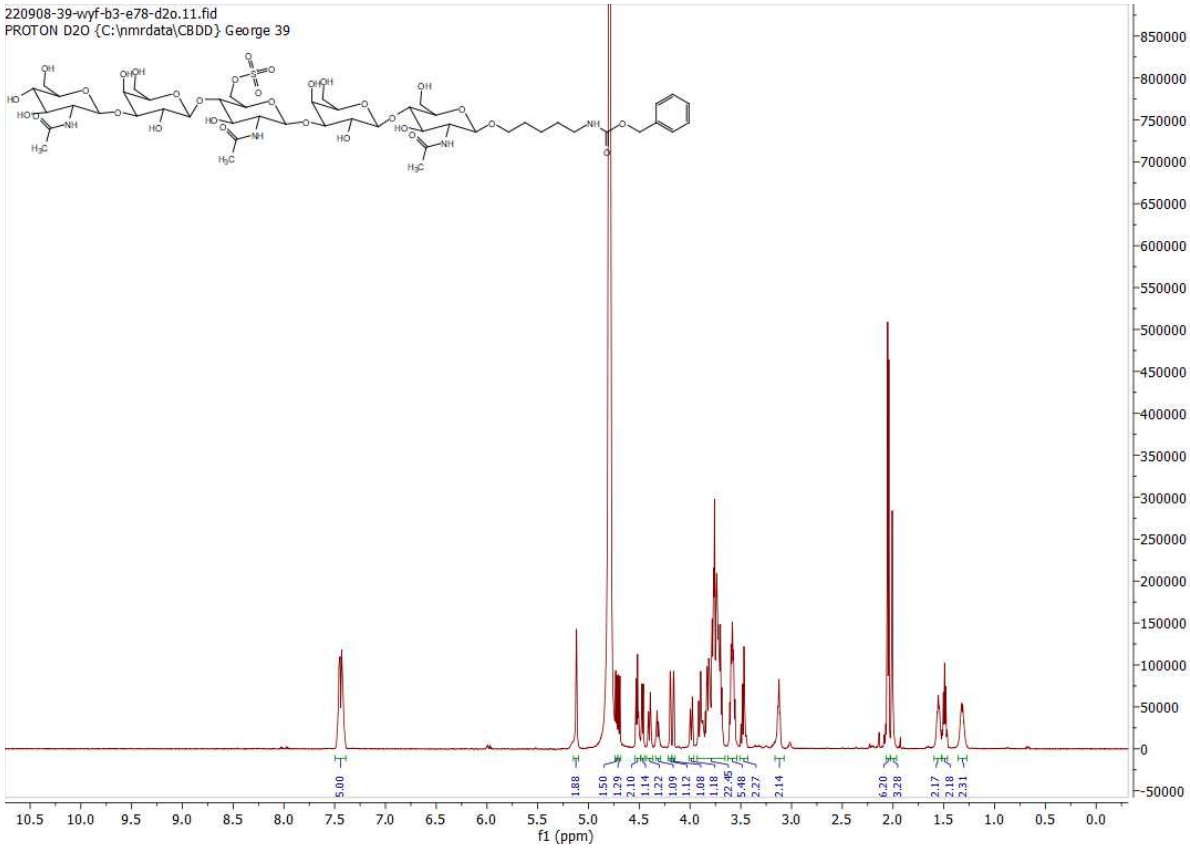

^{1}H NMR of 8; 600MHz; D_2O

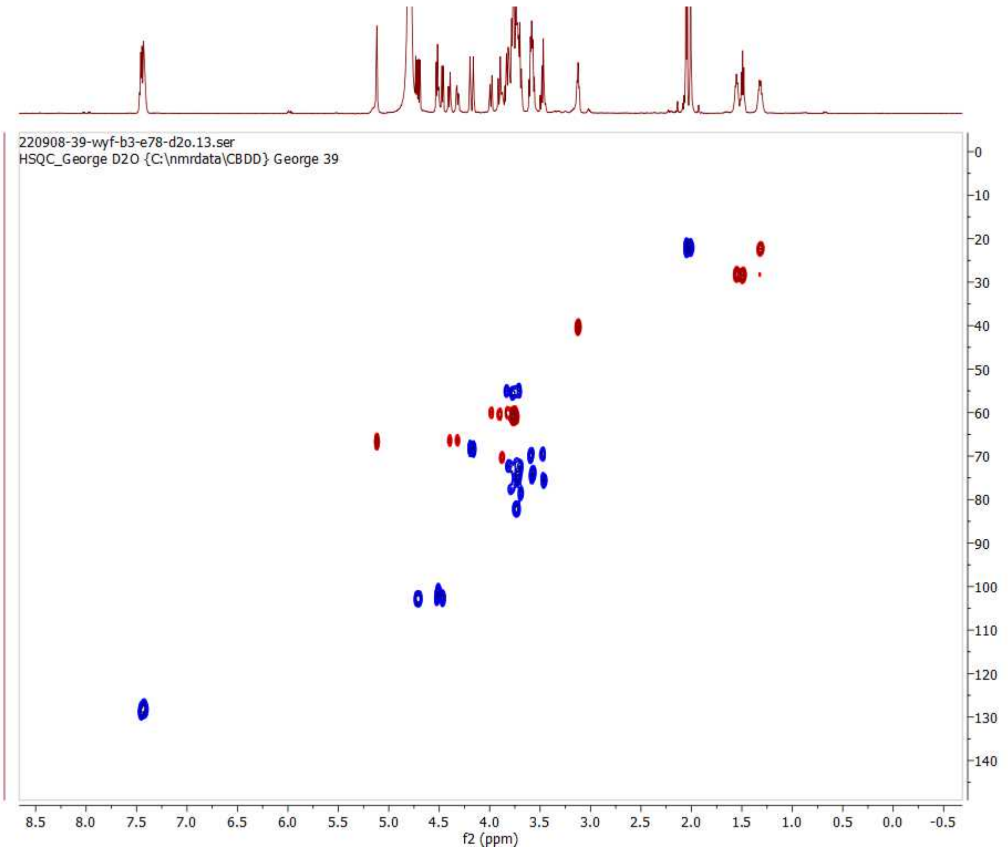

HSQC of 8; 600 MHz/150 MHz, D_2O

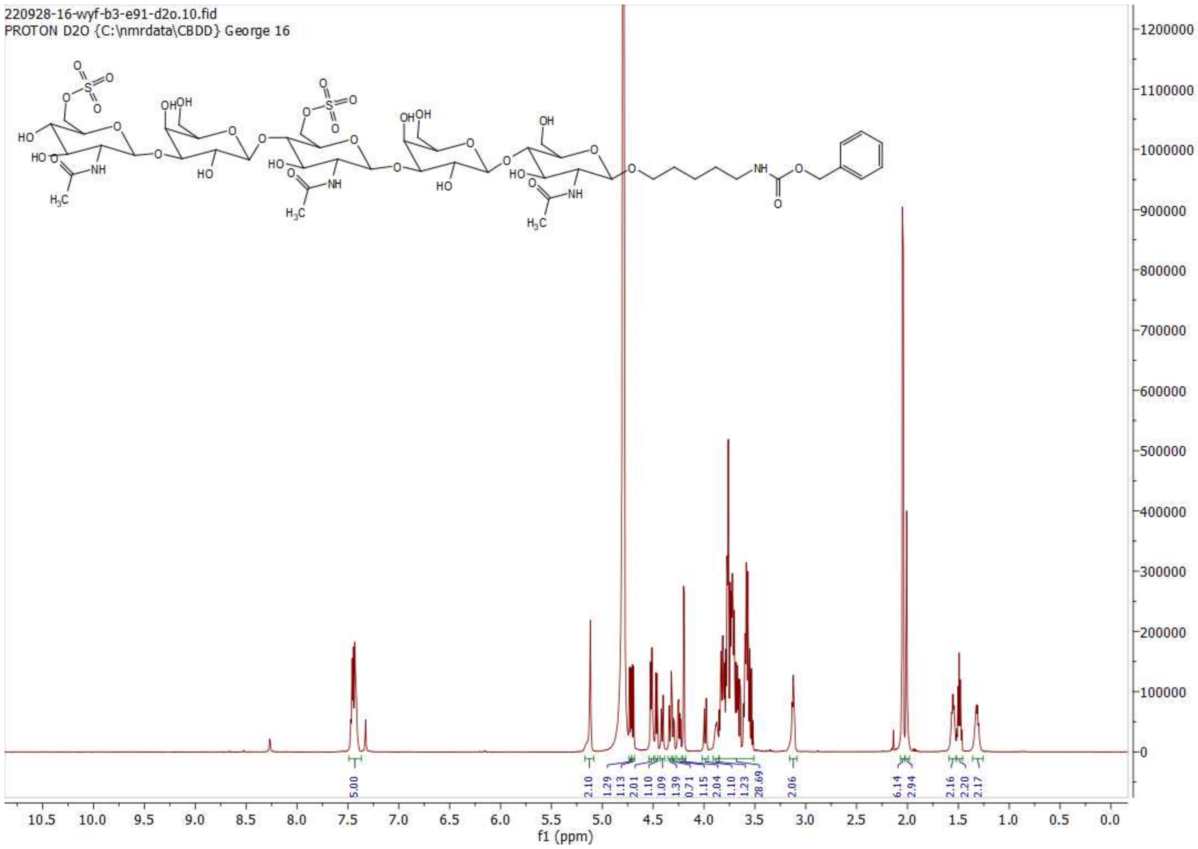

^{1}H NMR of 9; 600MHz; D_2O

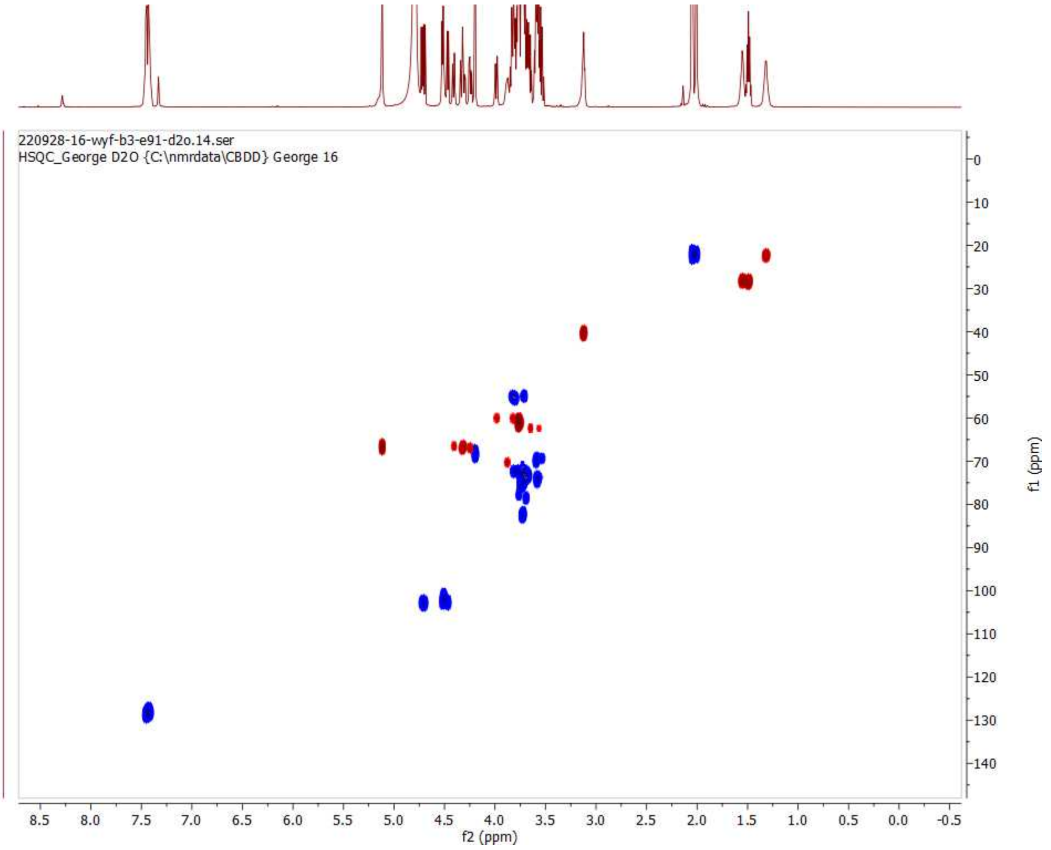

HSQC of 9; 600 MHz/150 MHz, D_2O

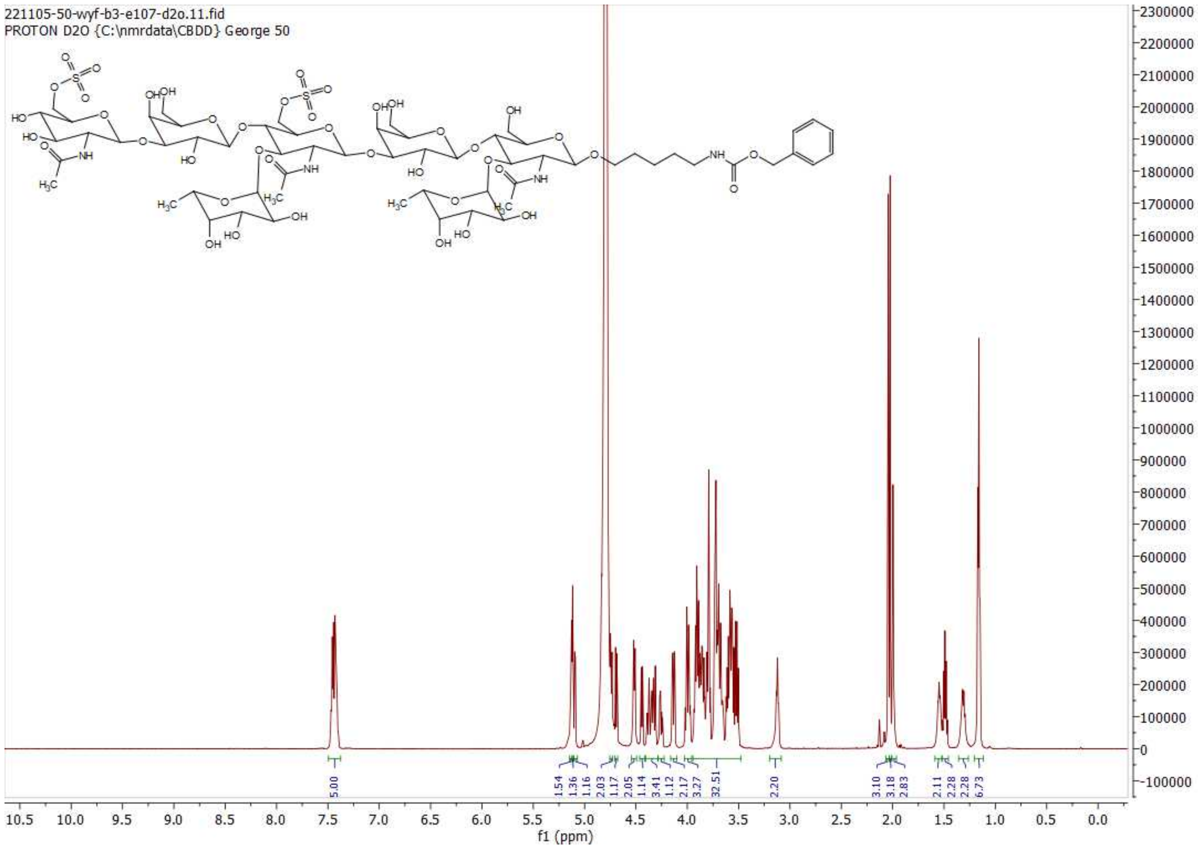

^{1}H NMR of 10; 600MHz; D_2O

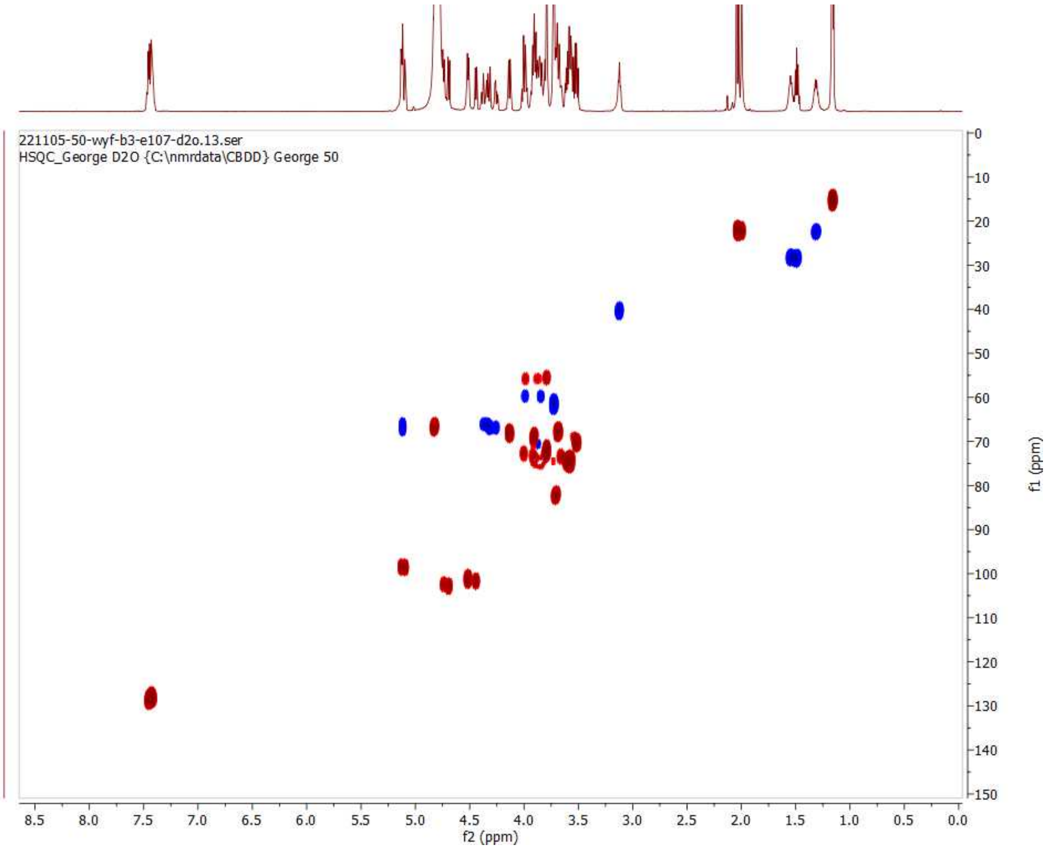

HSQC of 10; 600 MHz/150 MHz, D_2O

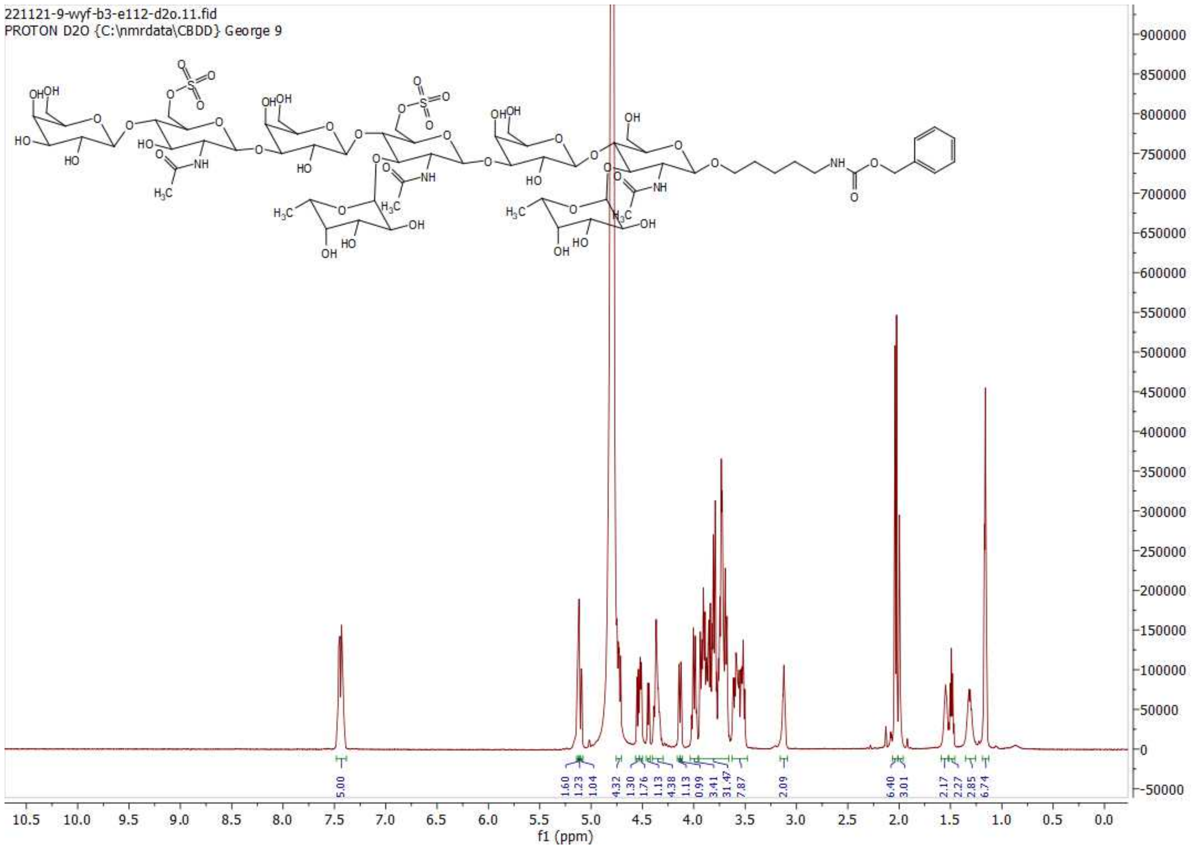

^{1}H NMR of 11; 600MHz; D_2O

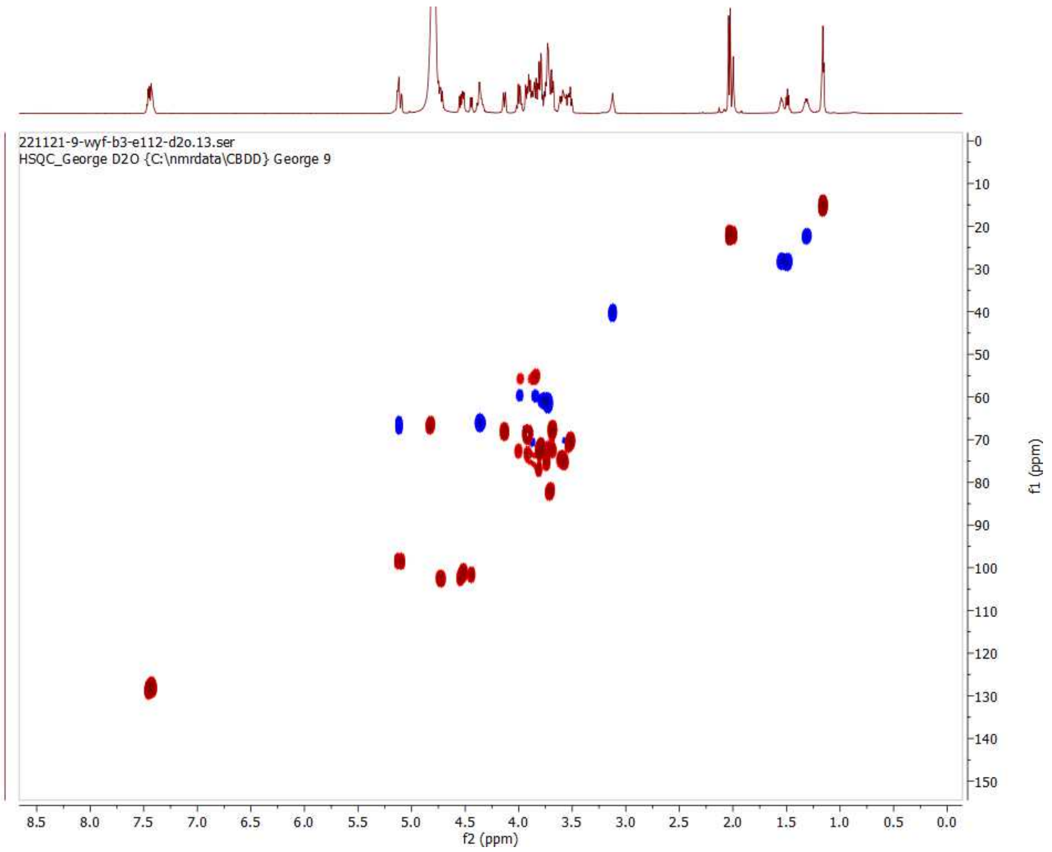

HSQC of 11; 600 MHz/150 MHz, D_2O

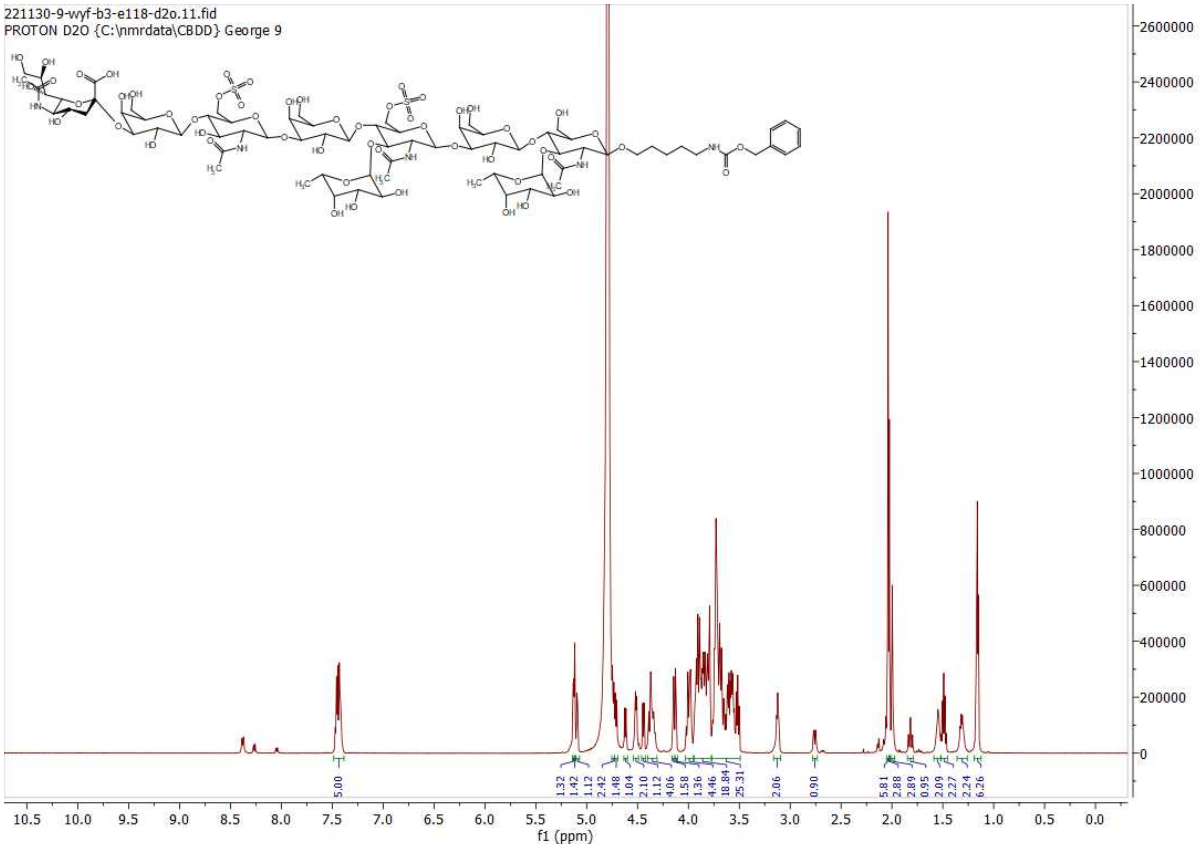

^{1}H NMR of 12; 600MHz; D_2O

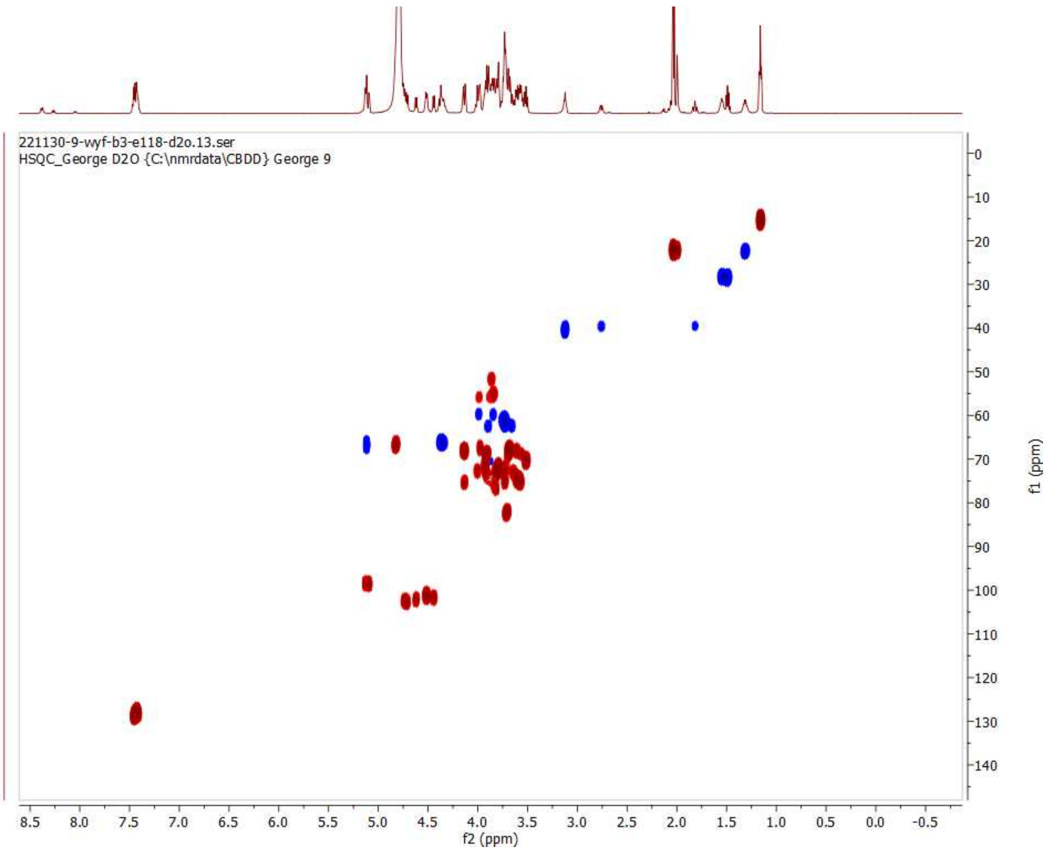

HSQC of 12; 600 MHz/150 MHz, D_2O

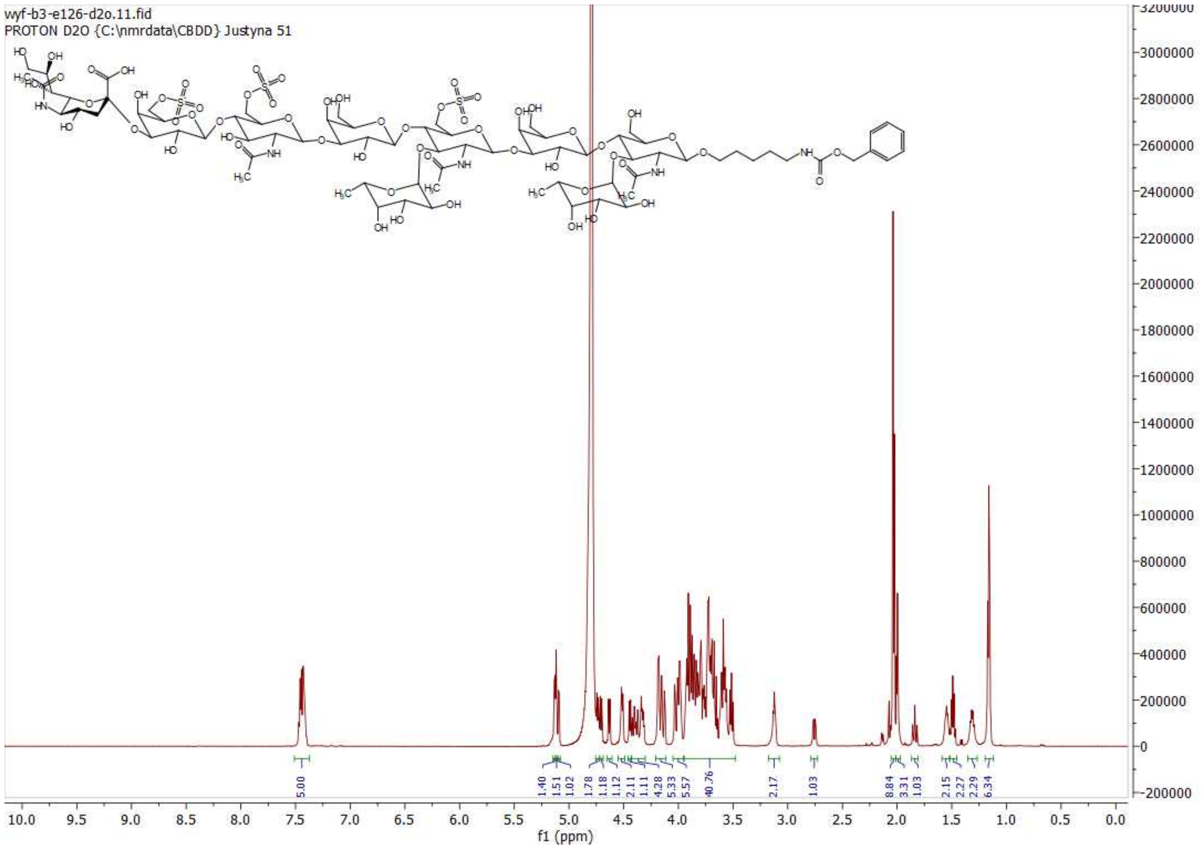

^{1}H NMR of 13; 600MHz; D_2O

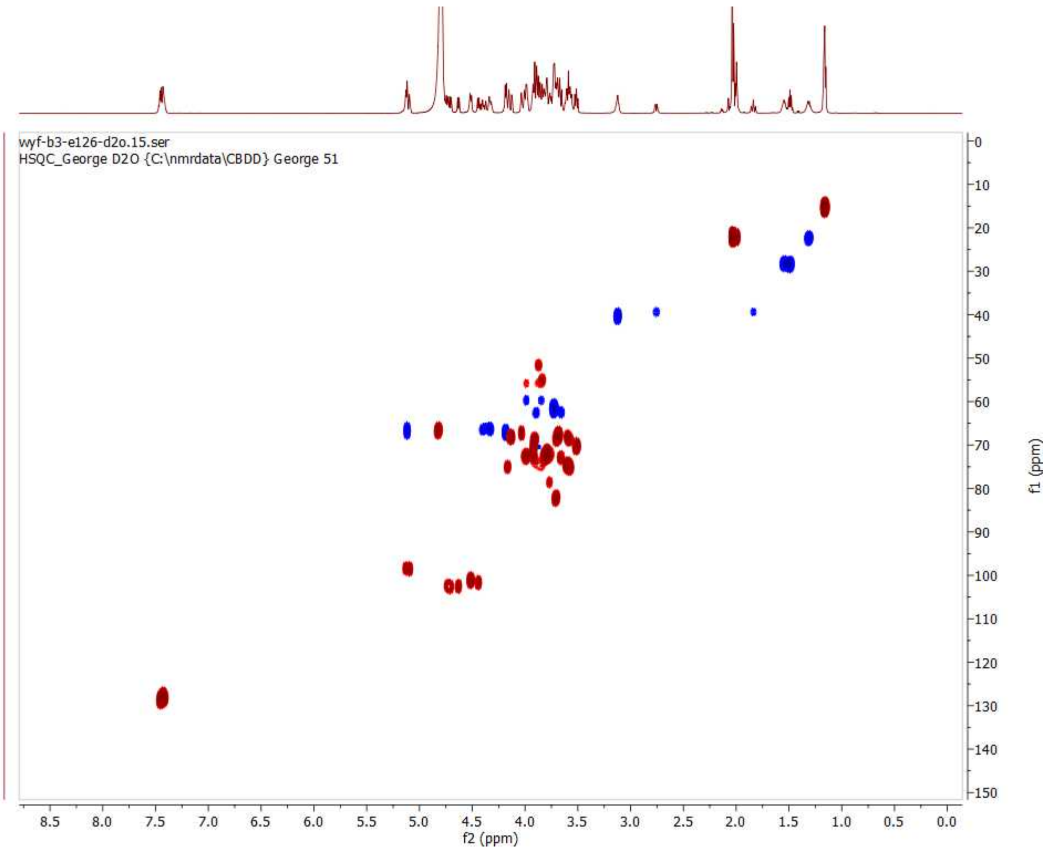

HSQC of 13; 600 MHz/150 MHz, D_2O

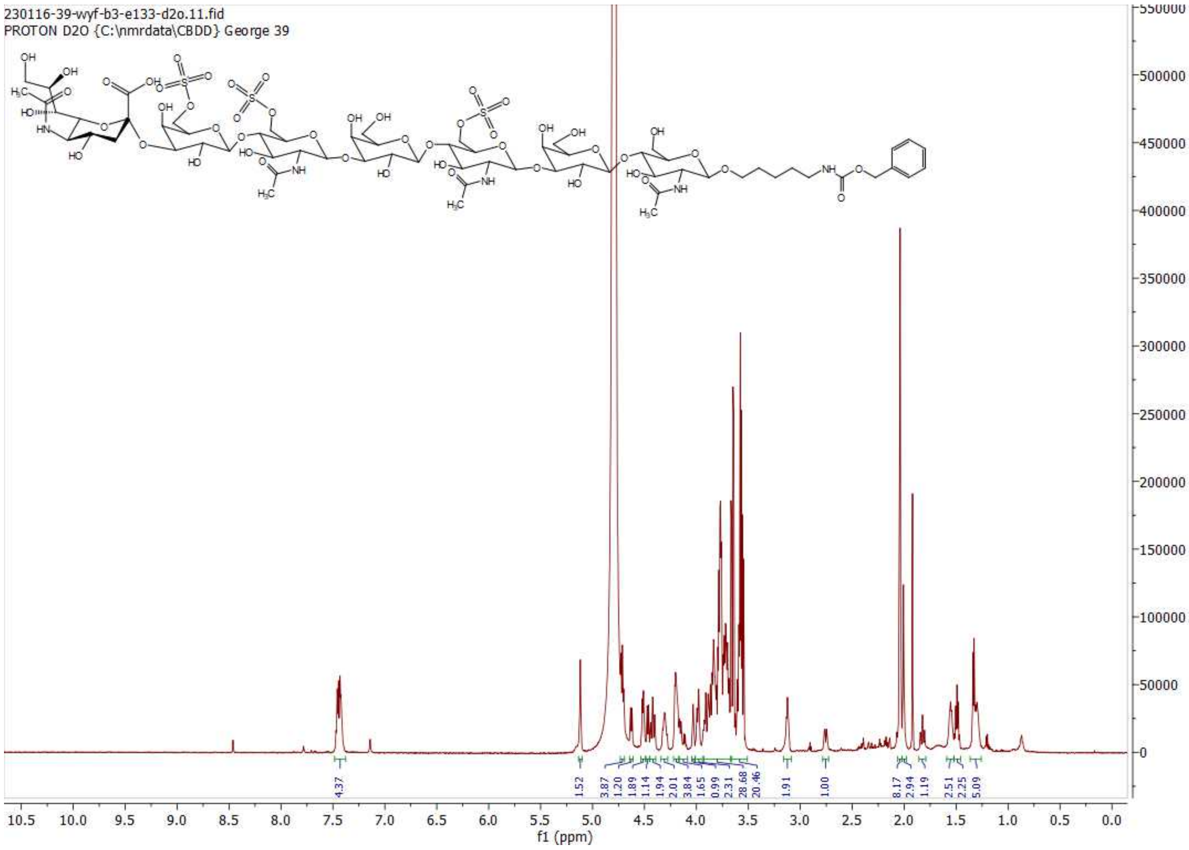

^{1}H NMR of 14; 600MHz; D_2O

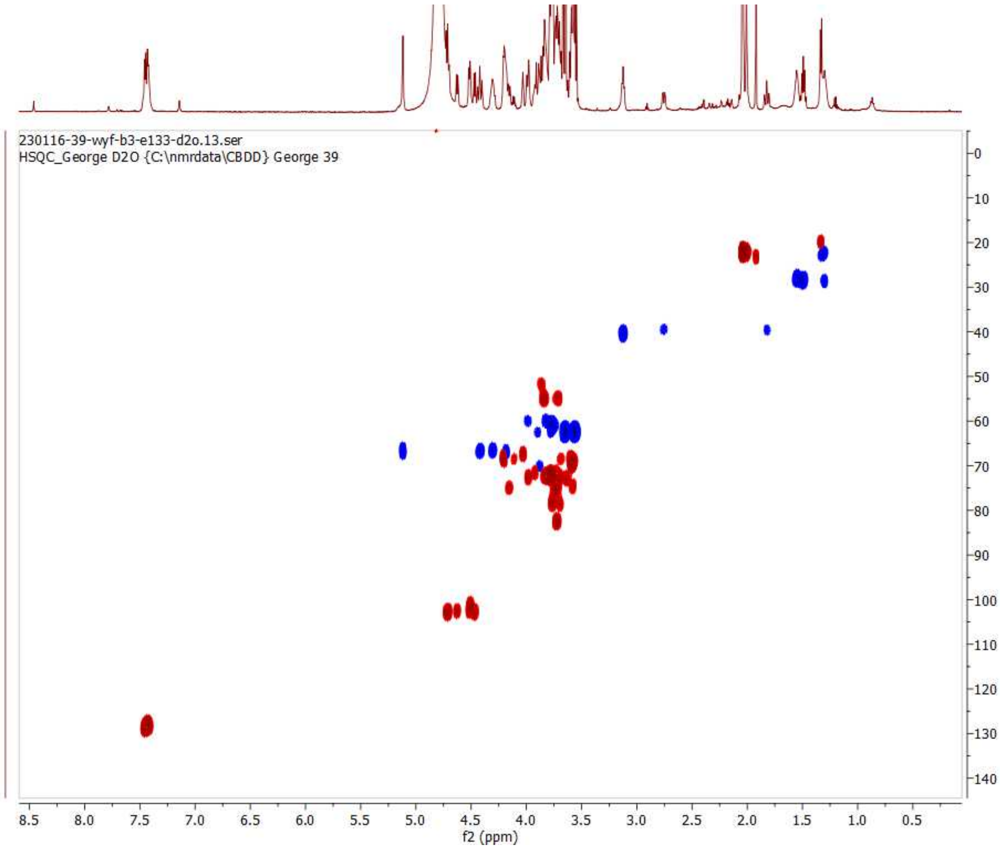

HSQC of 14; 600 MHz/150 MHz, D_2O

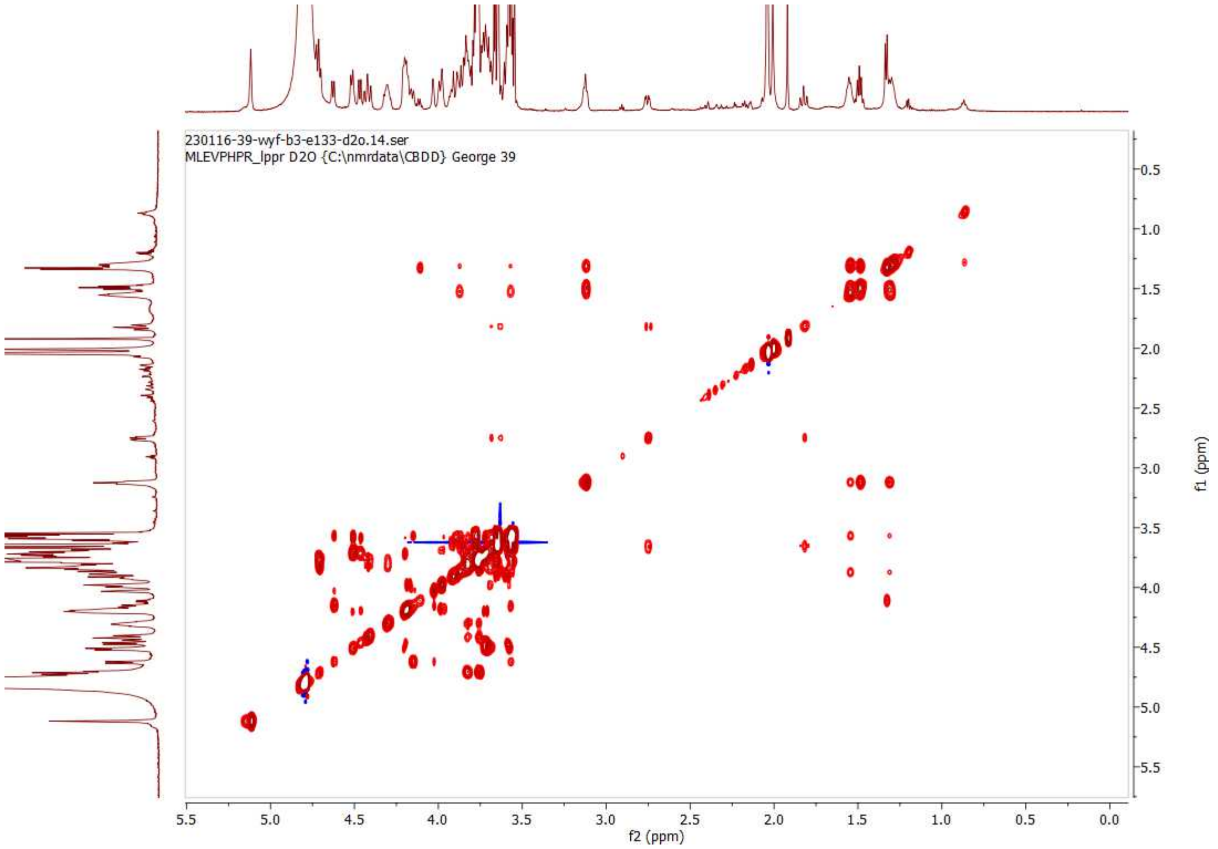

TOCSY (80 ms) of 14; 600MHz; D_2O

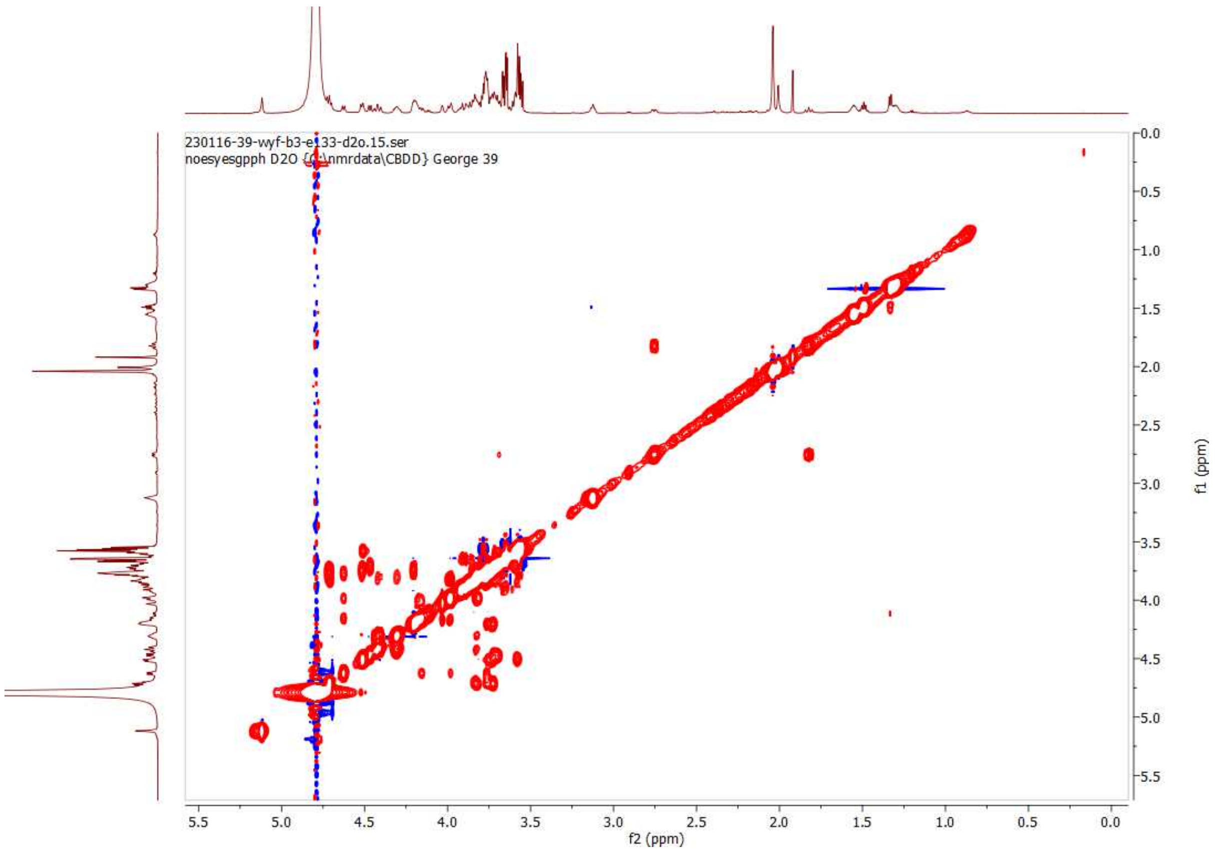

NOESY (300 ms) of 14; 600 MHz, D_2O

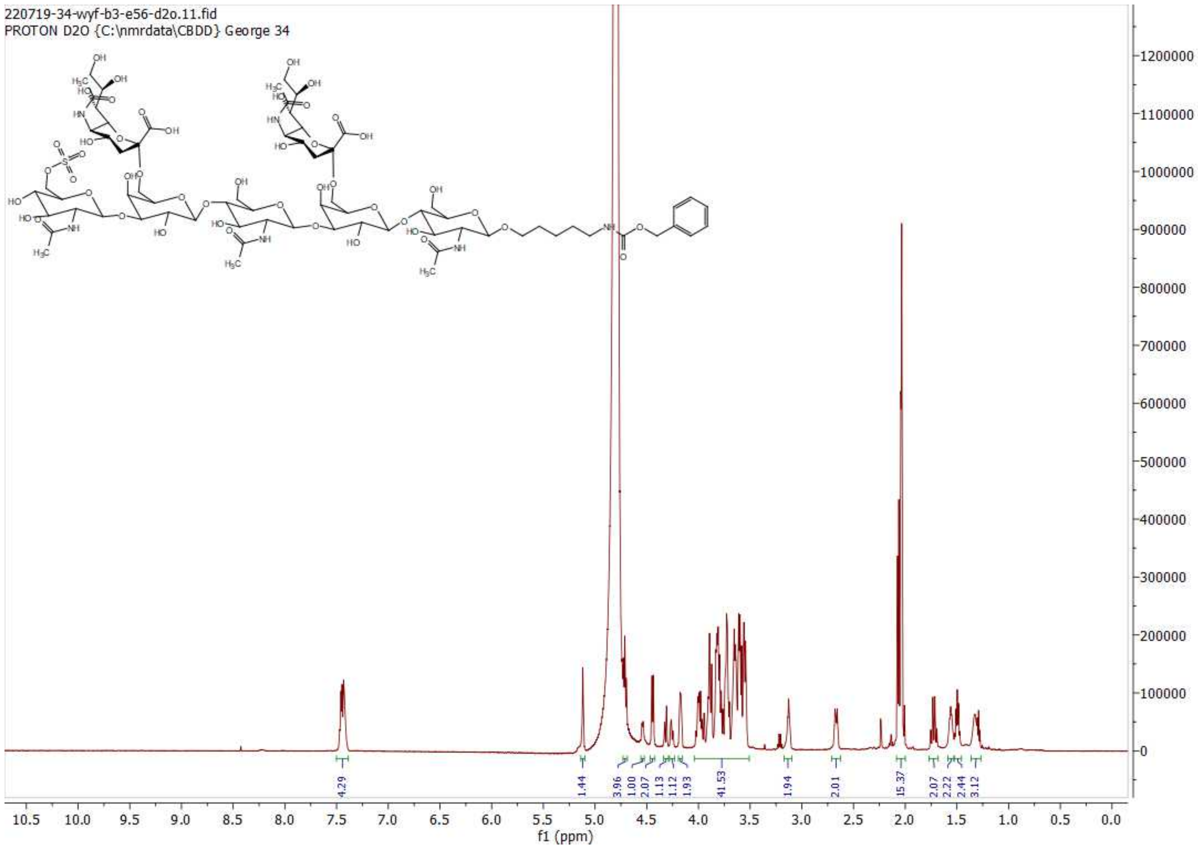

^{1}H NMR of S6; 600MHz; D_2O

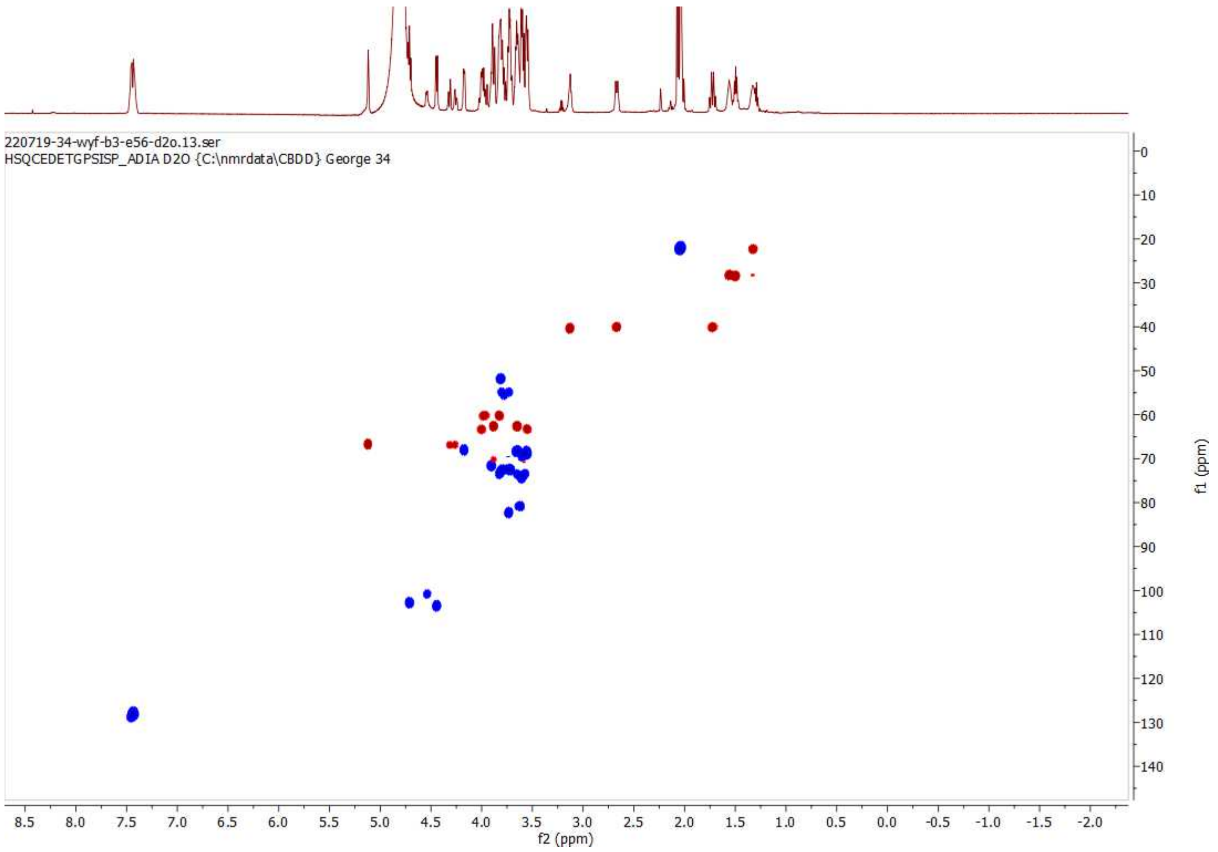

HSQC of S6; 600 MHz/150 MHz, D_2O

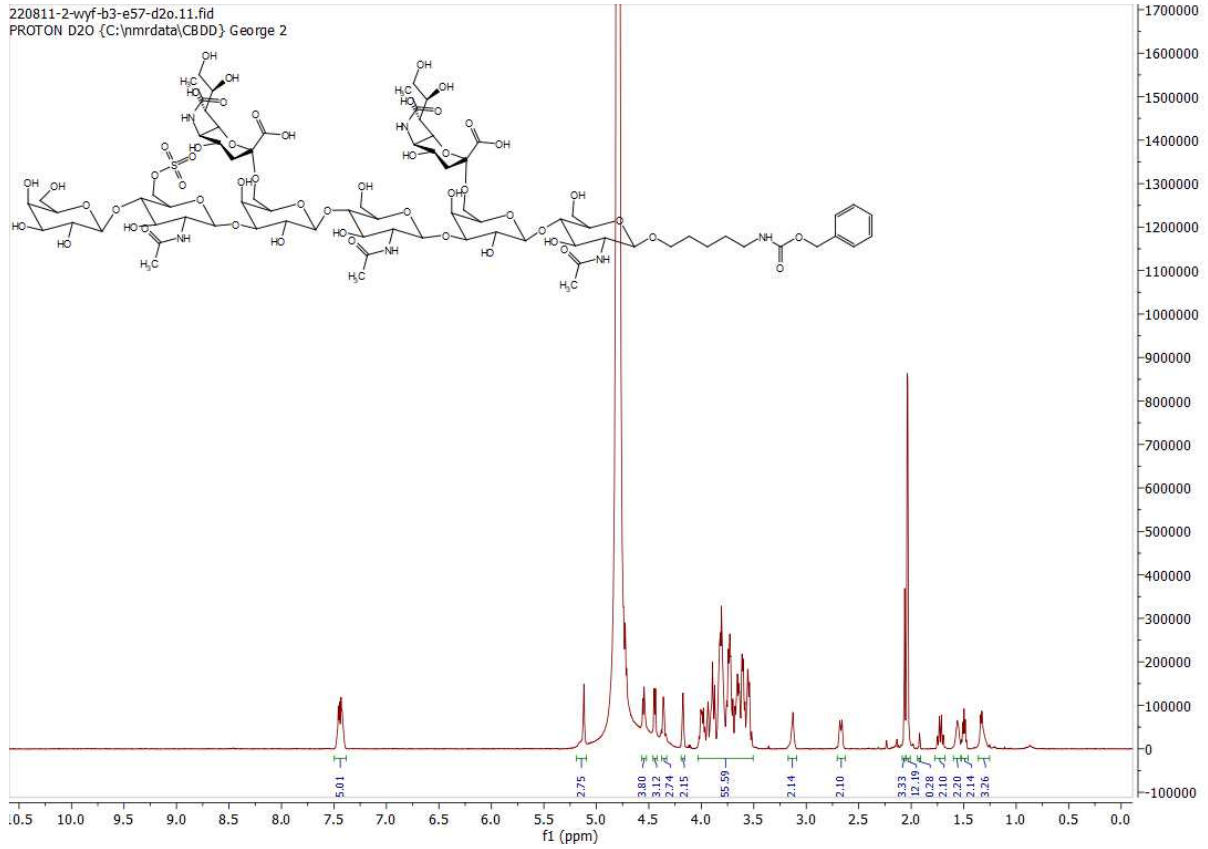

^{1}H NMR of S7; 600MHz; D_2O

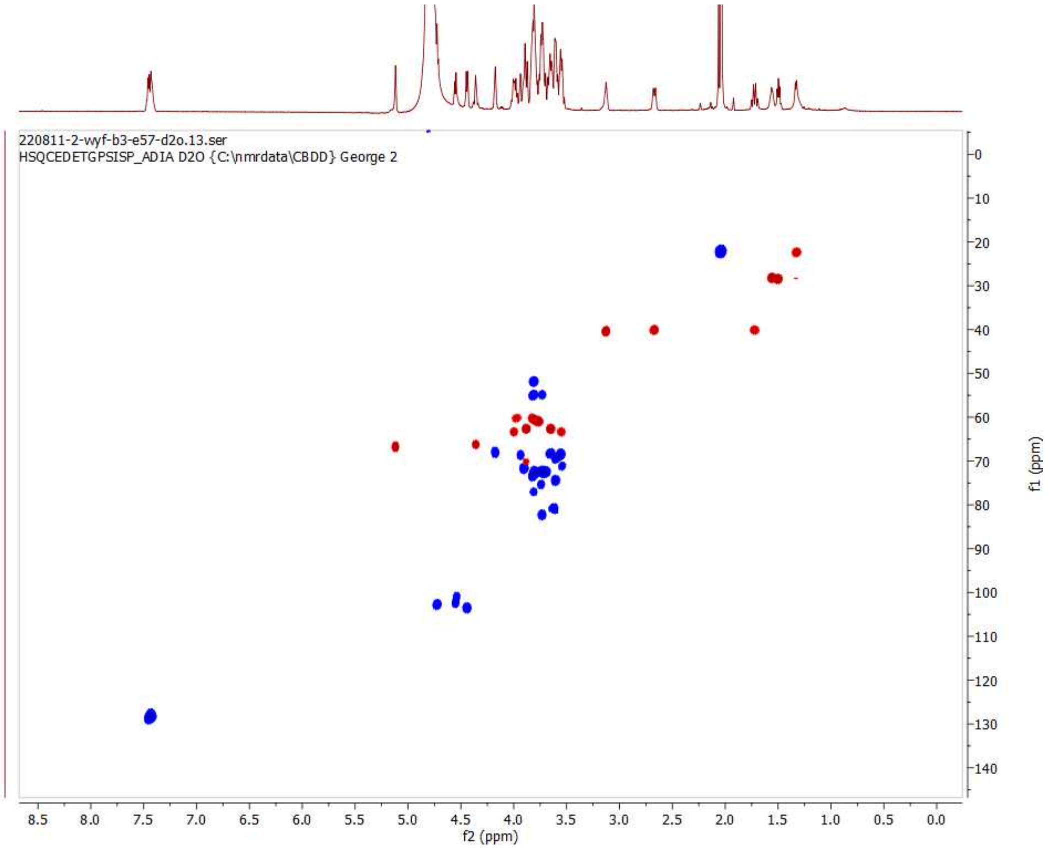

HSQC of S7; 600 MHz/150 MHz, D_2O

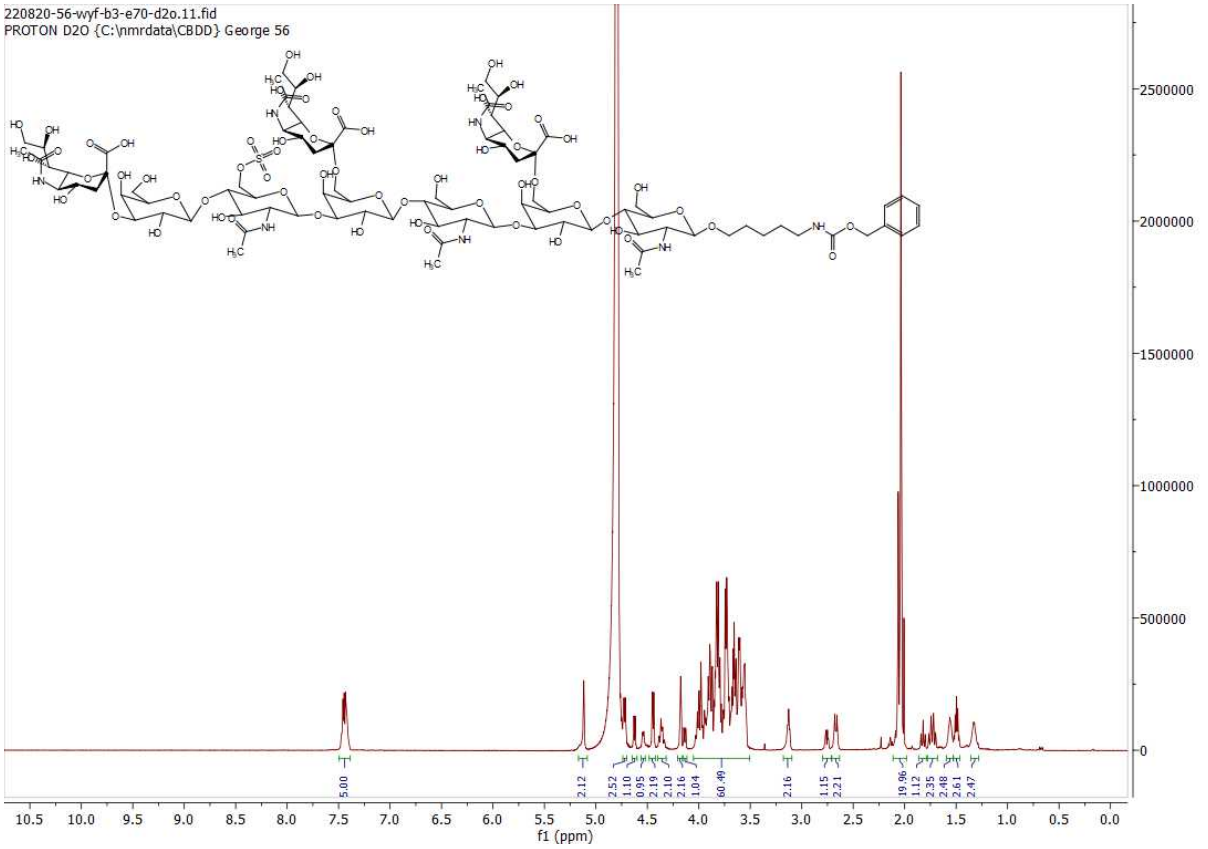

^{1}H NMR of S8; 600MHz; D_2O

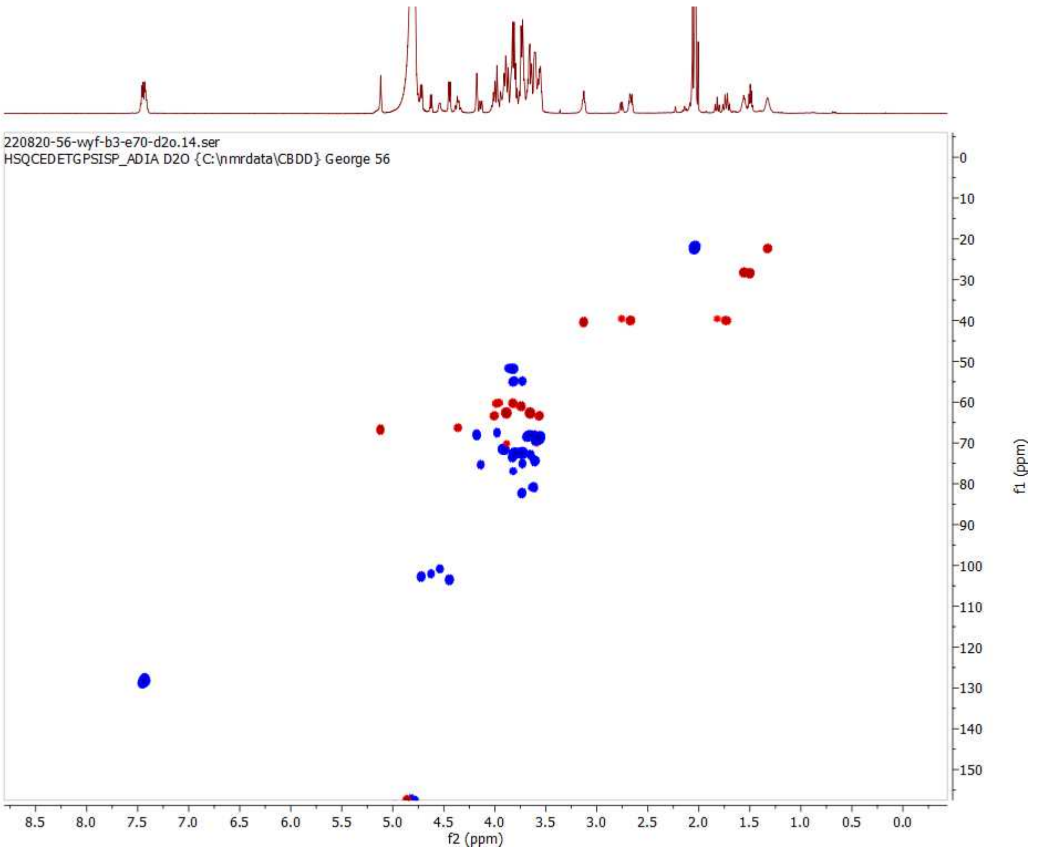

HSQC of S8; 600 MHz/150 MHz, D_2O

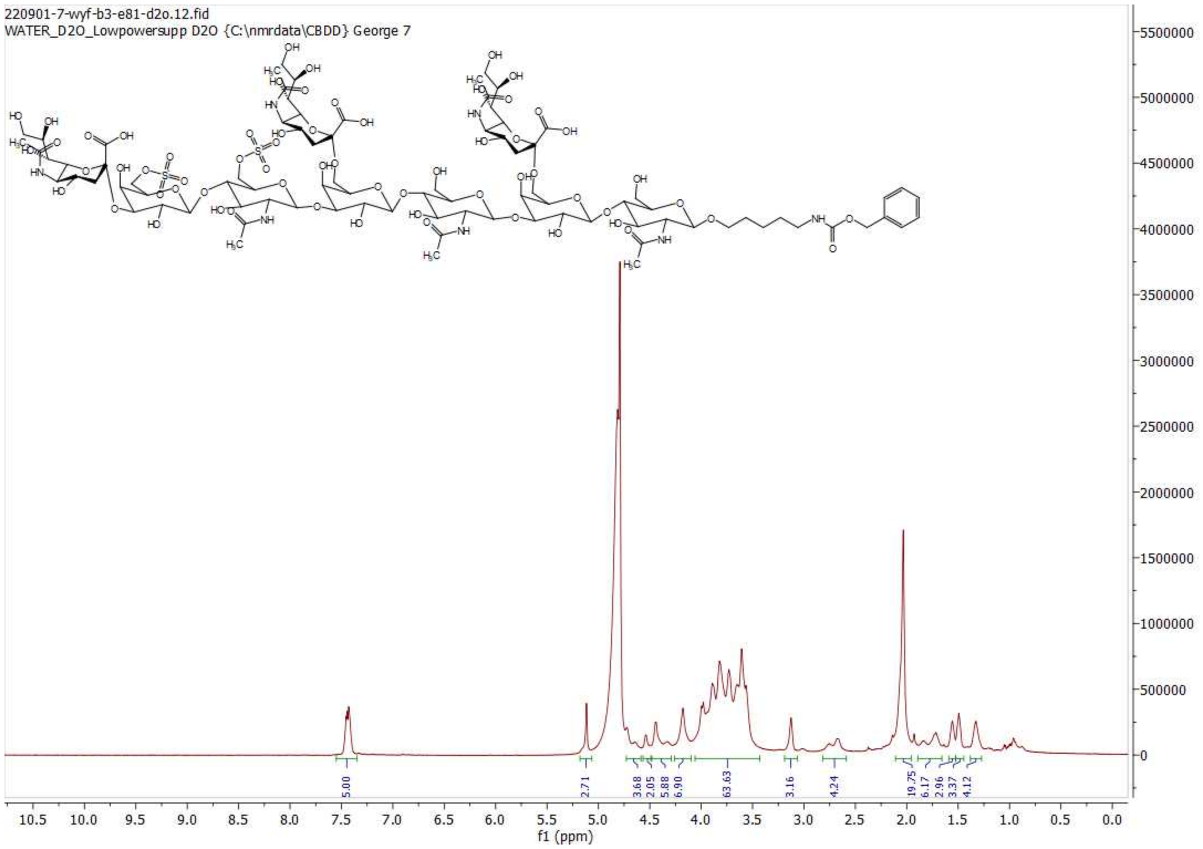

^{1}H NMR of S9; 600MHz; D_2O

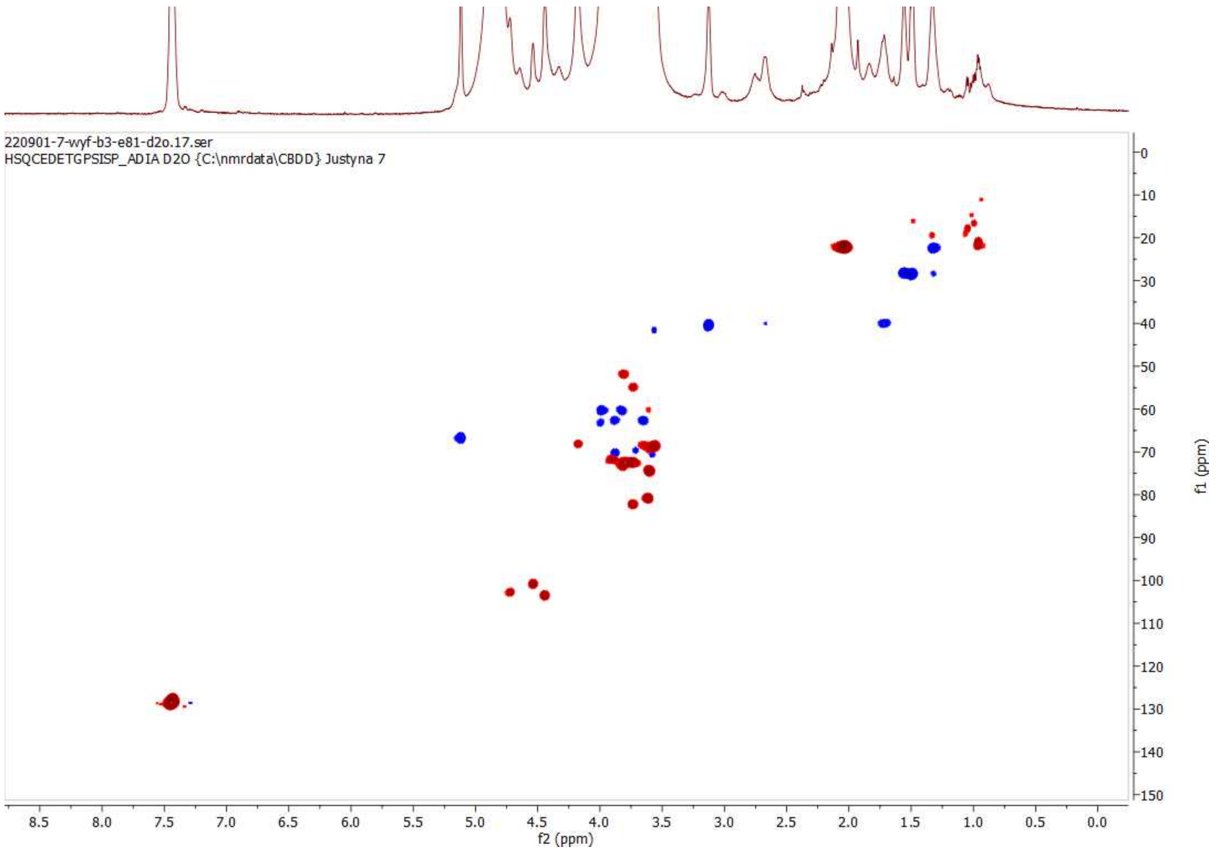

HSQC of S9; 600 MHz/150 MHz, D_2O

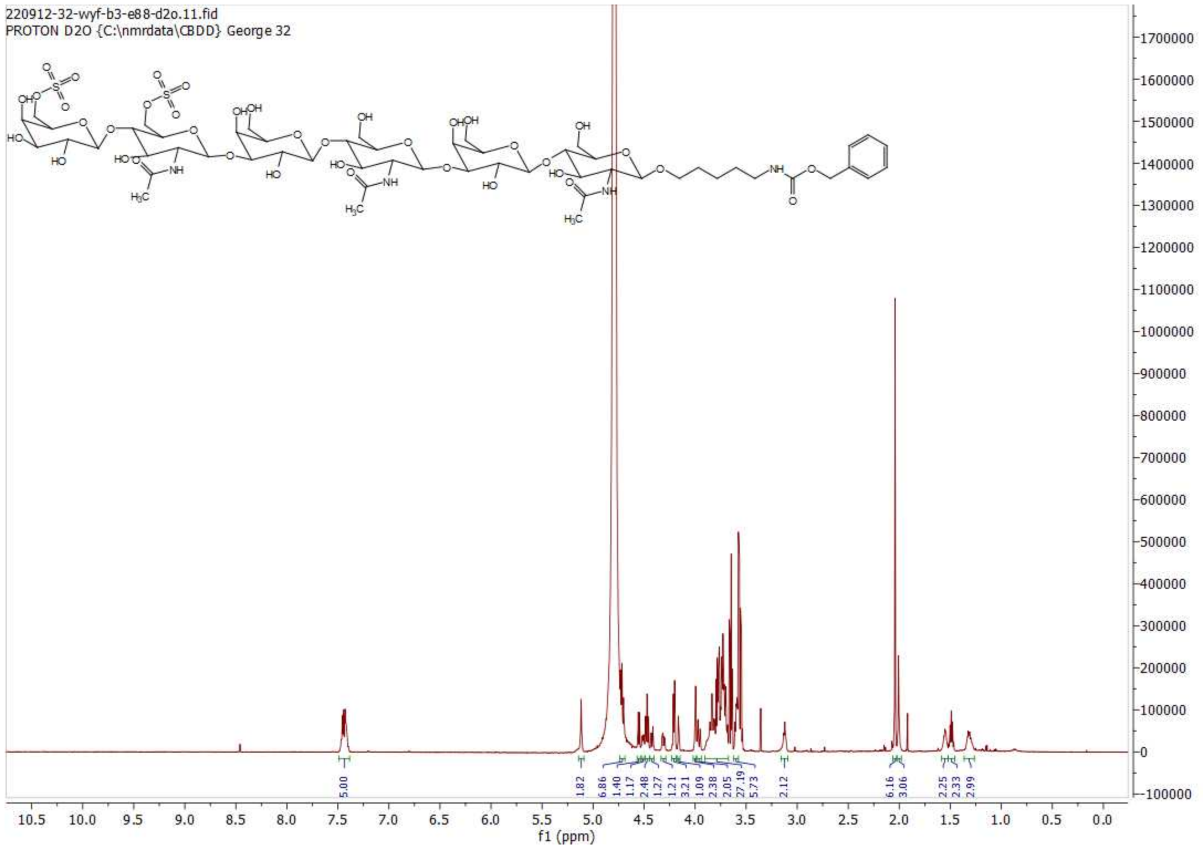

^{1}H NMR of S14; 600MHz; D_2O

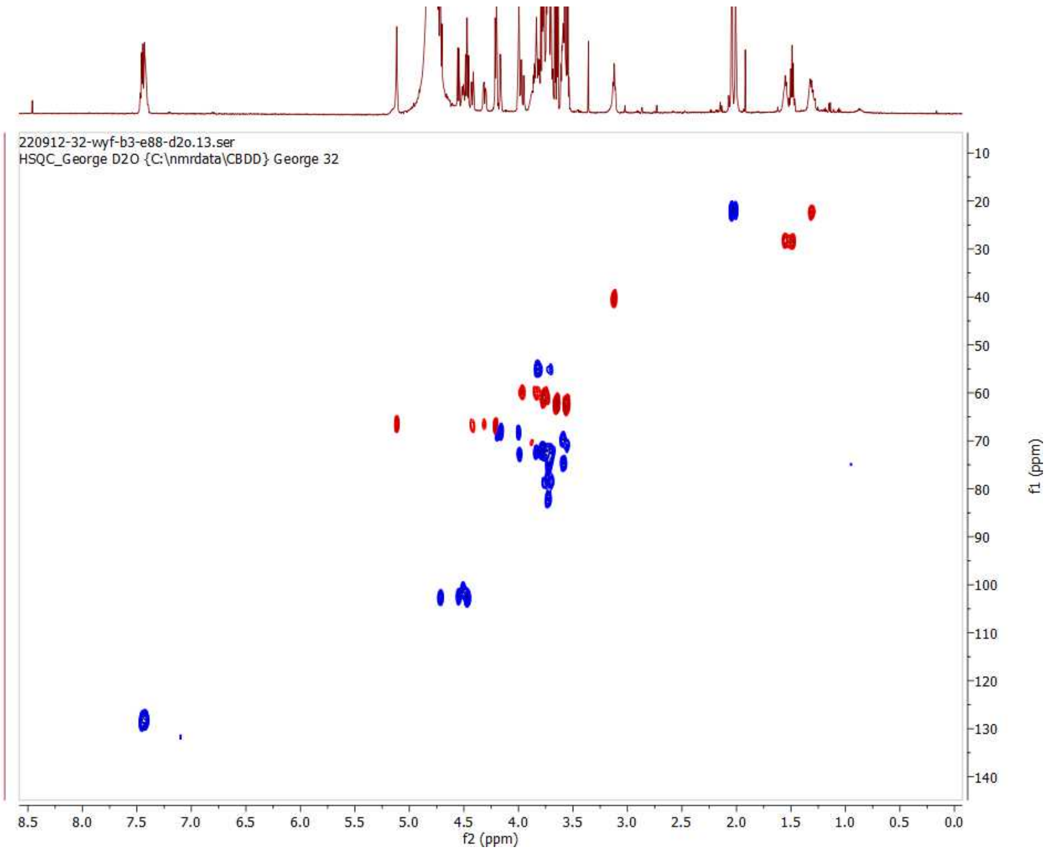

HSQC of S14; 600 MHz/150 MHz, D_2O

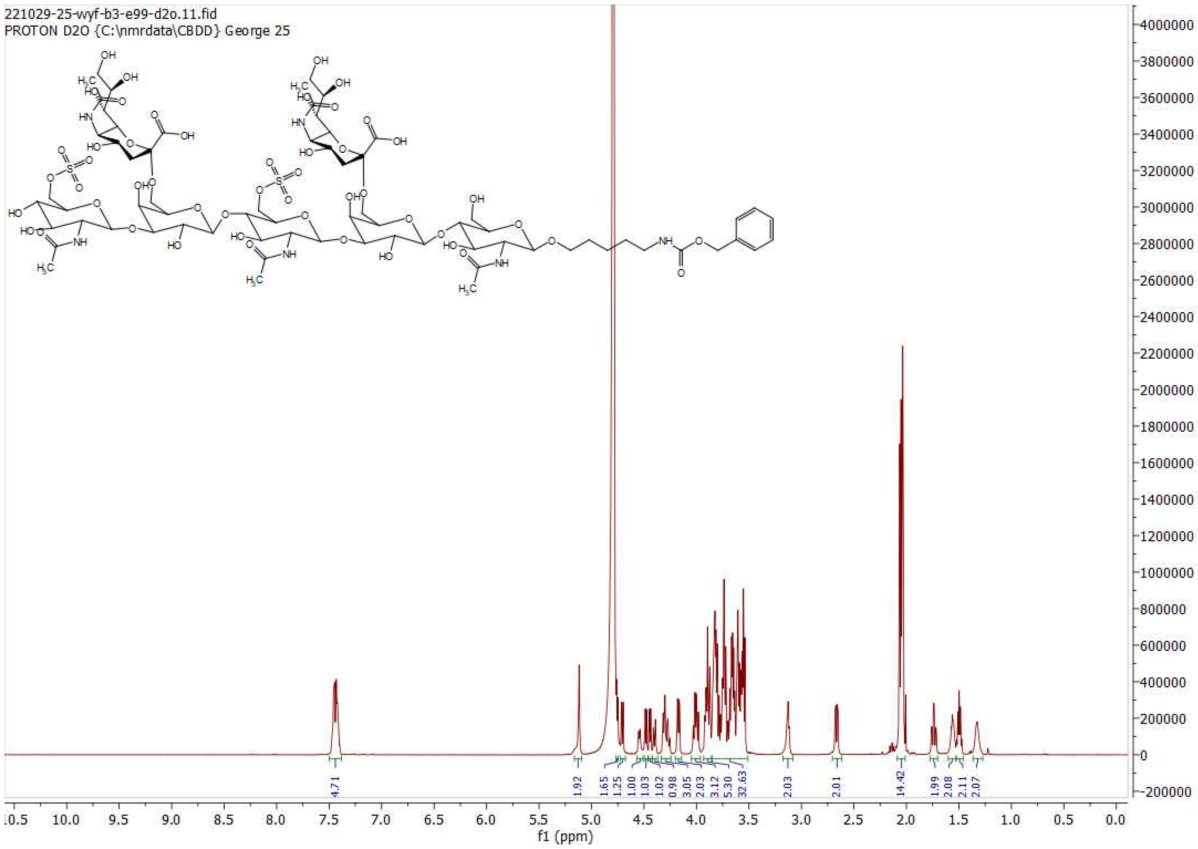

^{1}**H NMR of 15; 600MHz; D$_2$O**

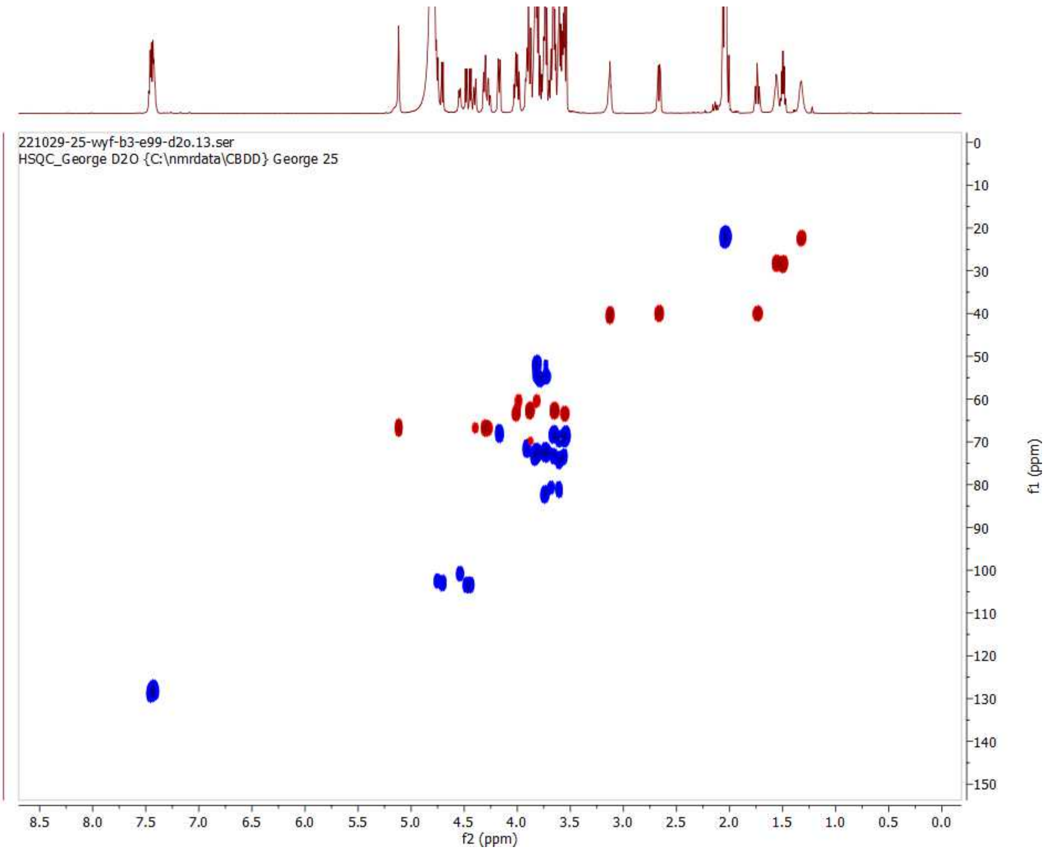

HSQC of 15; 600 MHz/150 MHz, D$_2$O

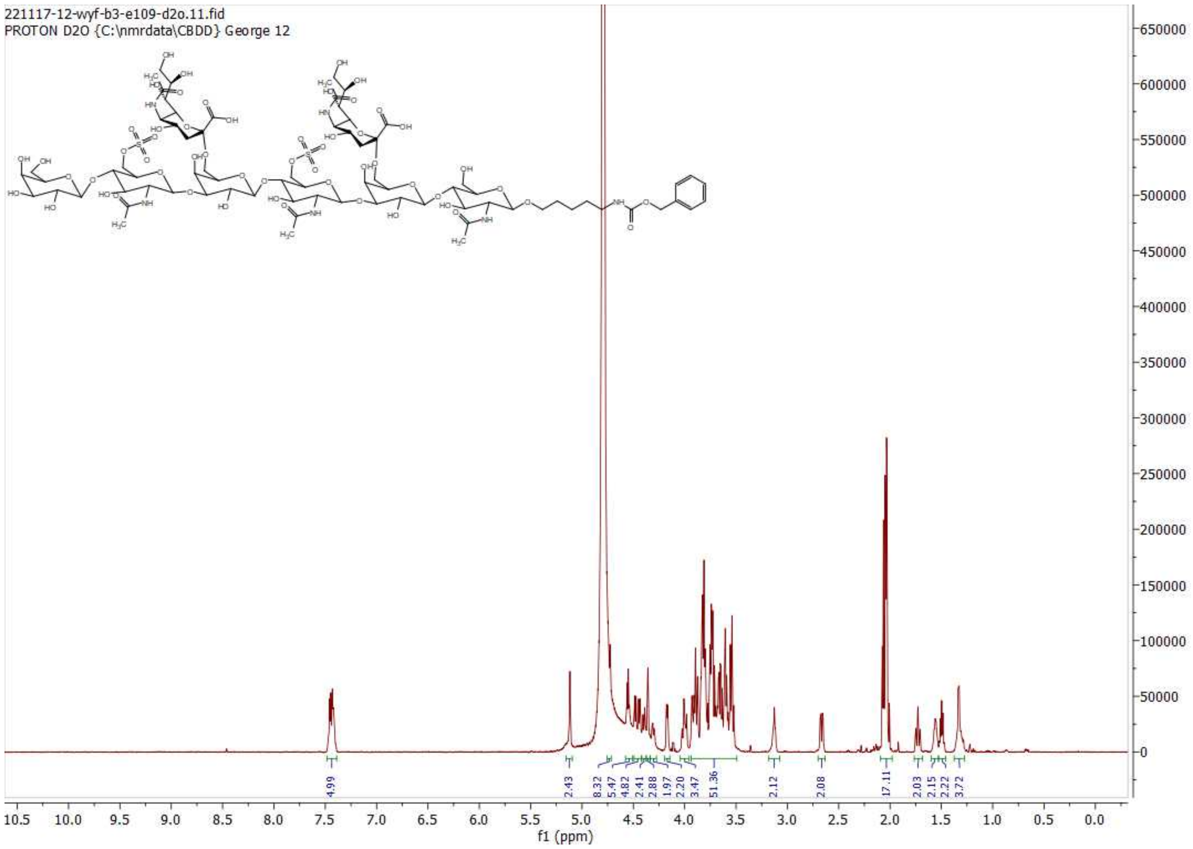

^{1}H NMR of 16; 600MHz; D_2O

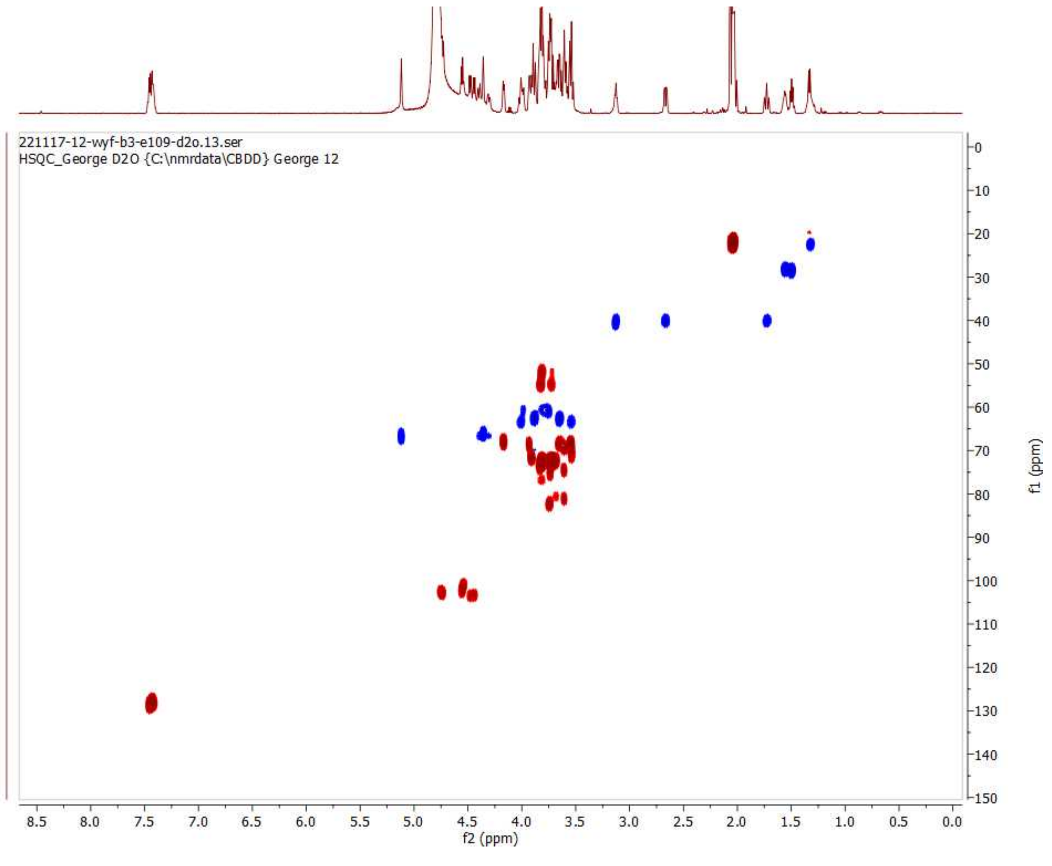

HSQC of 16; 600 MHz/150 MHz, D_2O

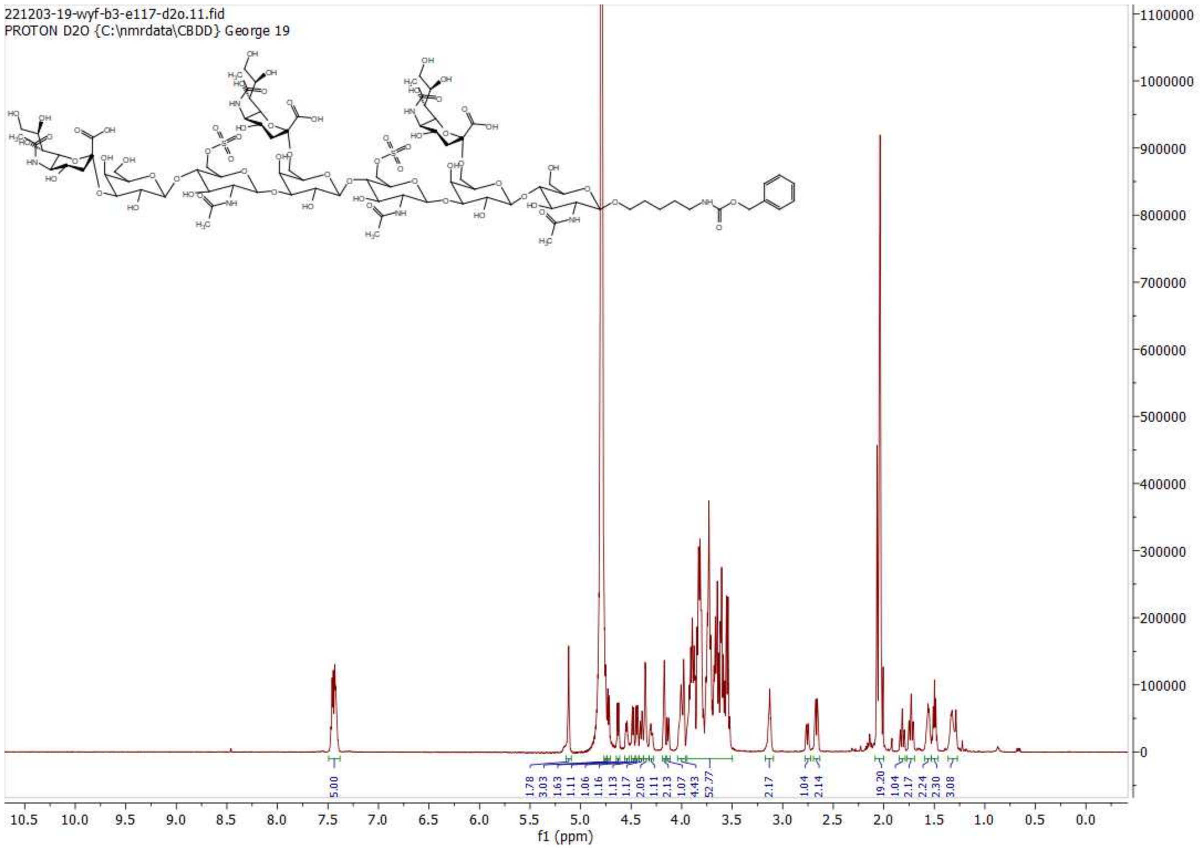

^{1}H NMR of 17; 600MHz; D_2O

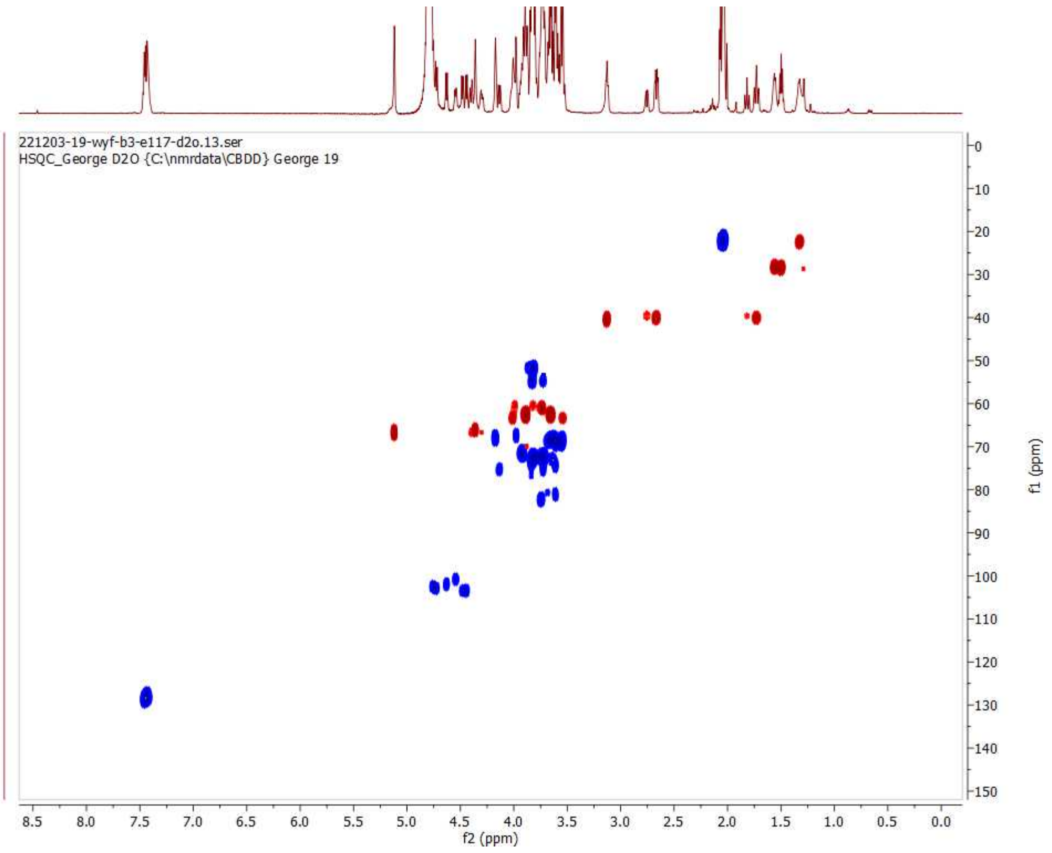

HSQC of 17; 600 MHz/150 MHz, D_2O

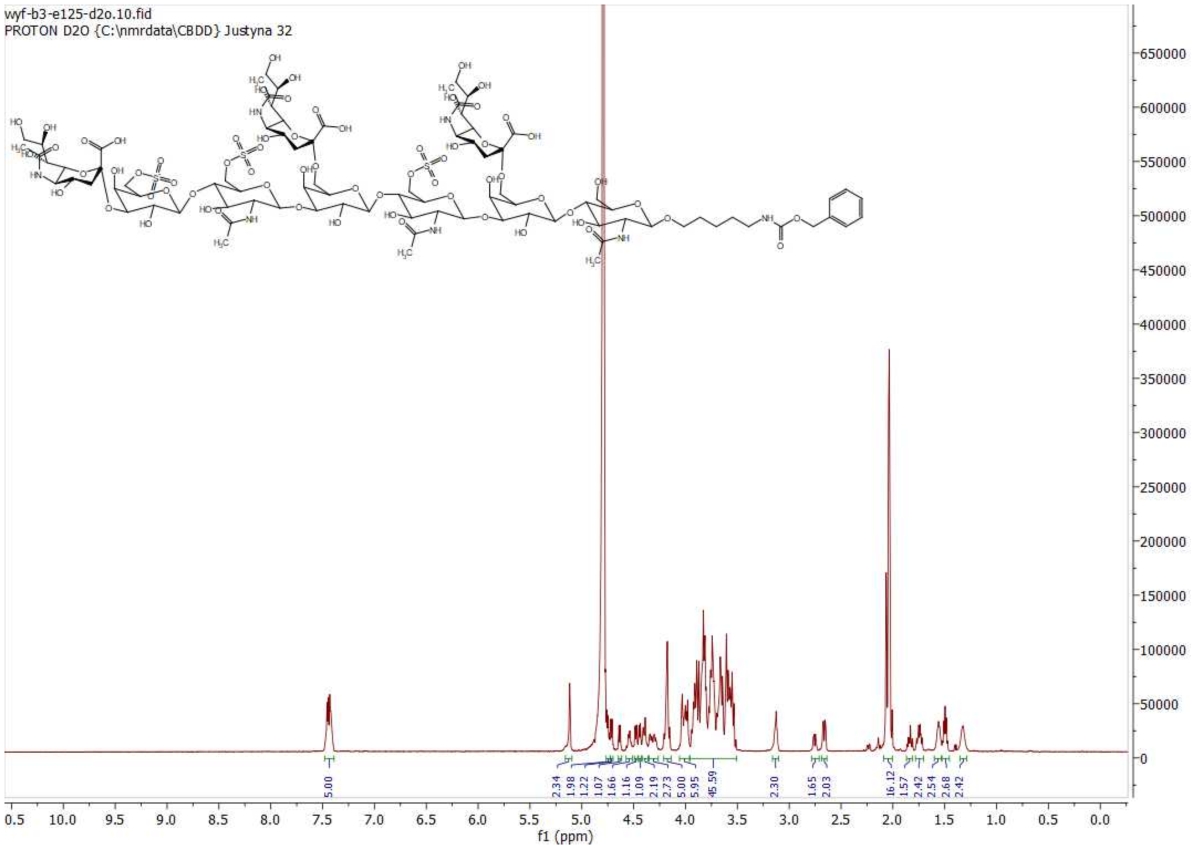

^{1}H NMR of 18; 600MHz; D_2O

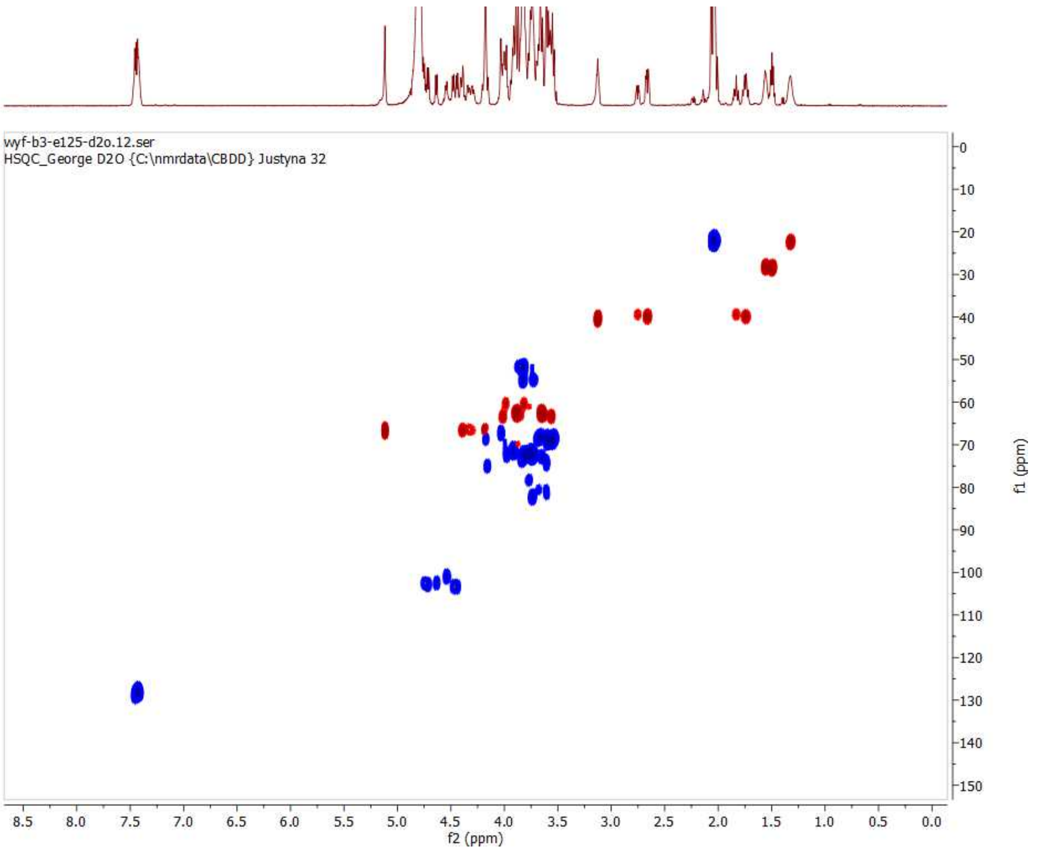

HSQC of 18; 600 MHz/150 MHz, D_2O

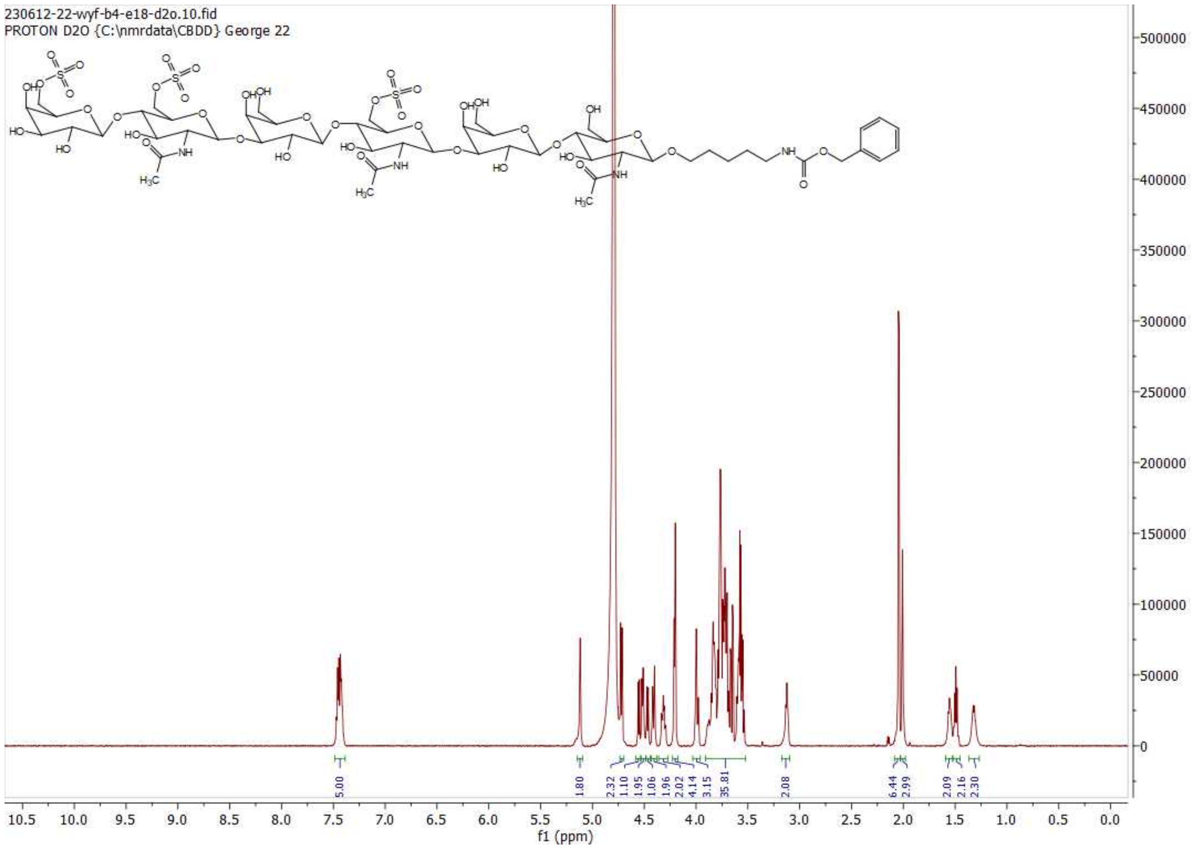

^{1}H NMR of S15; 600MHz; D_2O

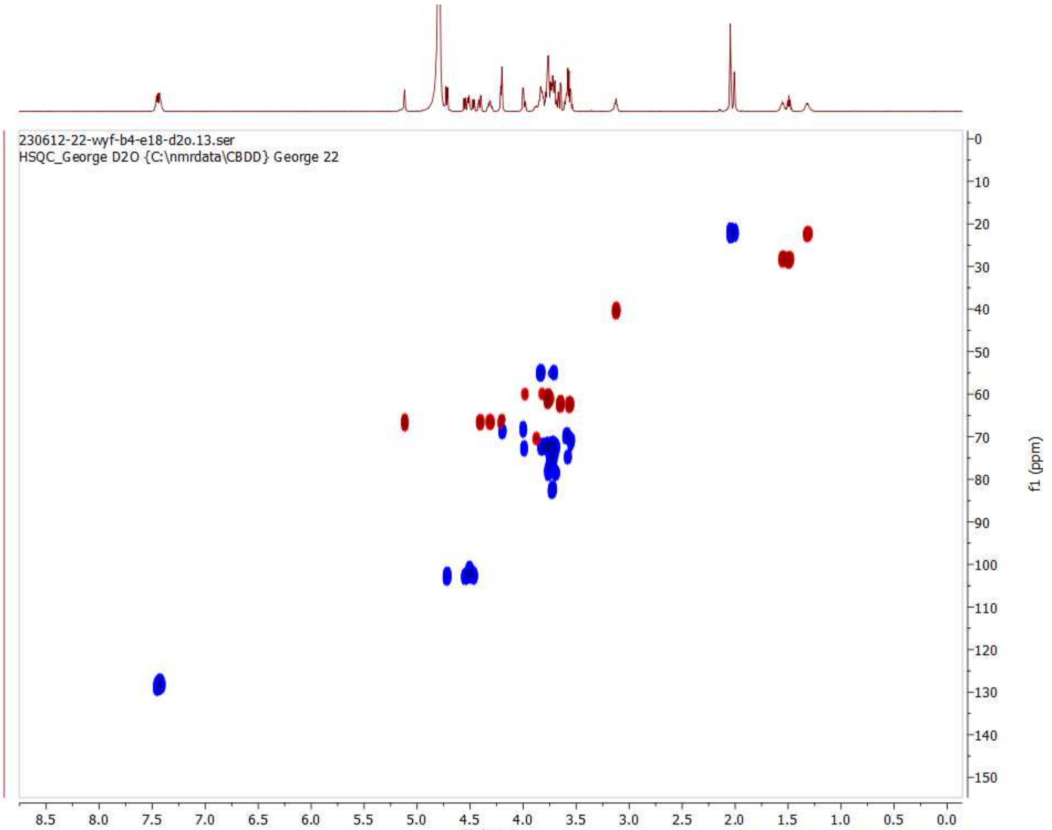

HSQC of S15; 600 MHz/150 MHz, D_2O

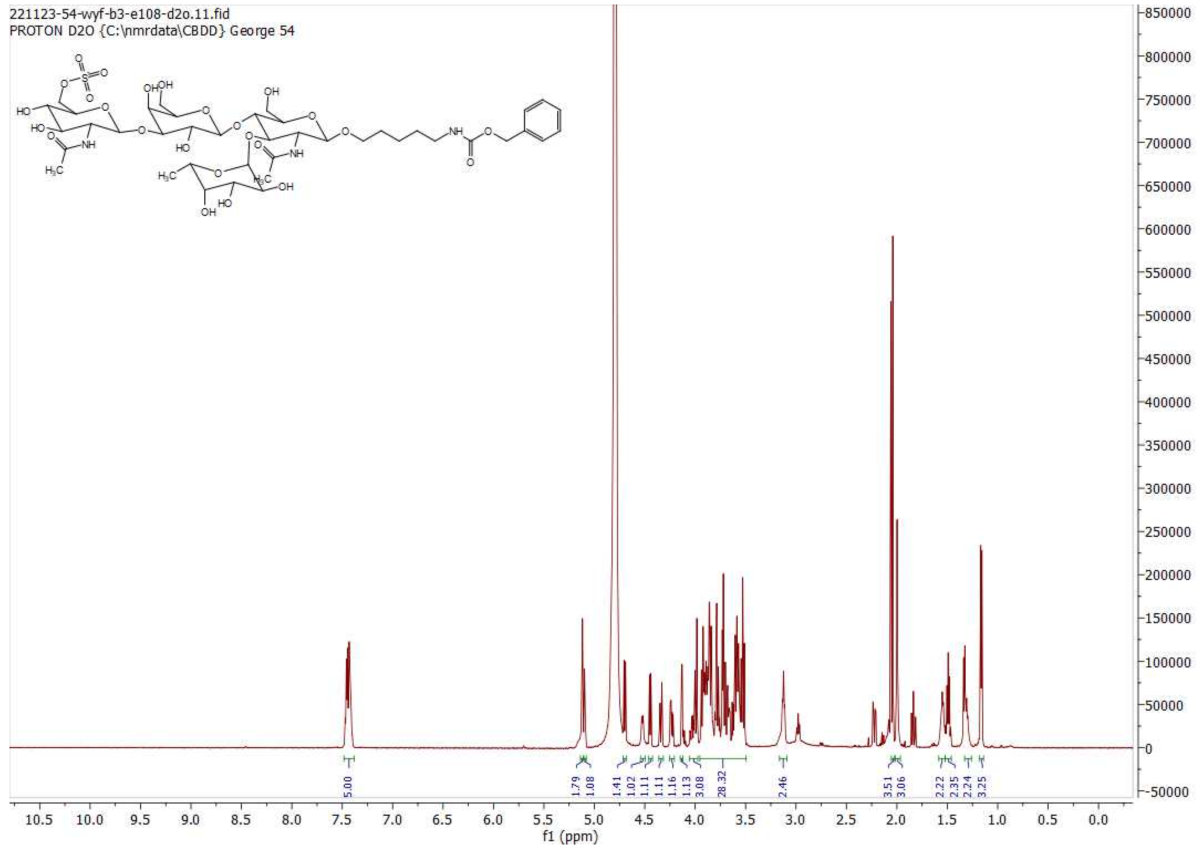

^{1}H NMR of S11; 600MHz; D_2O

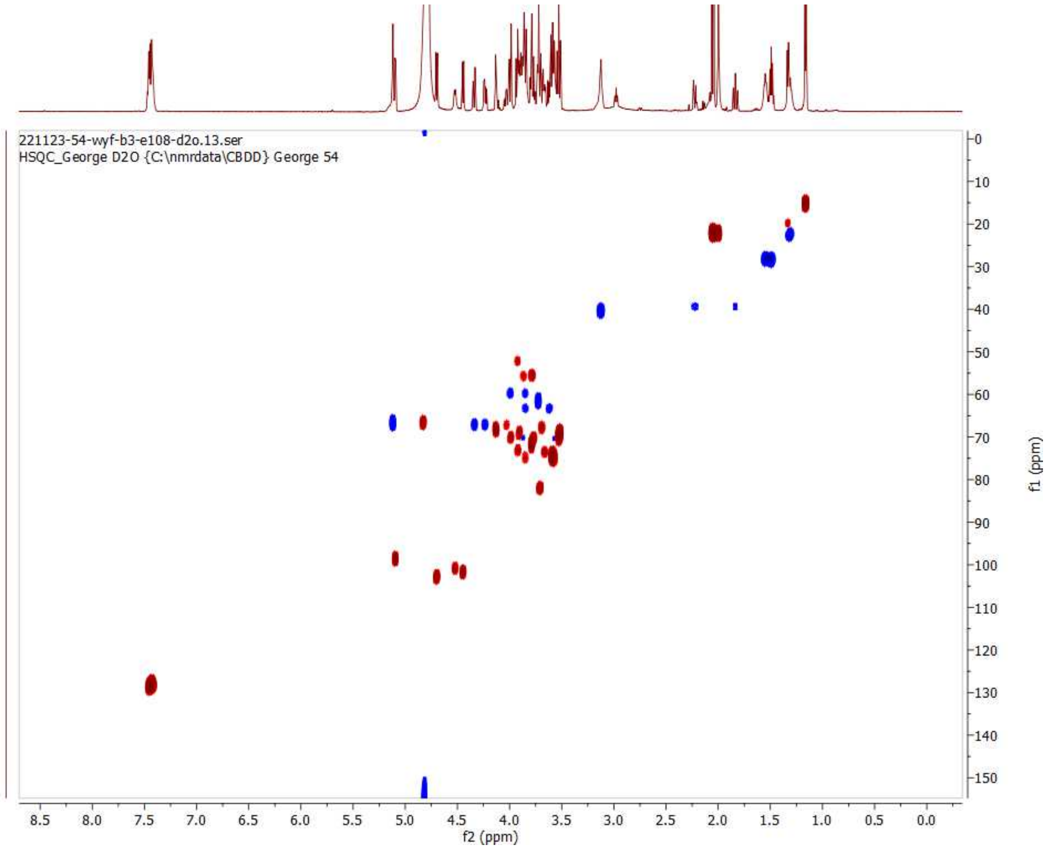

HSQC of S11; 600 MHz/150 MHz, D_2O

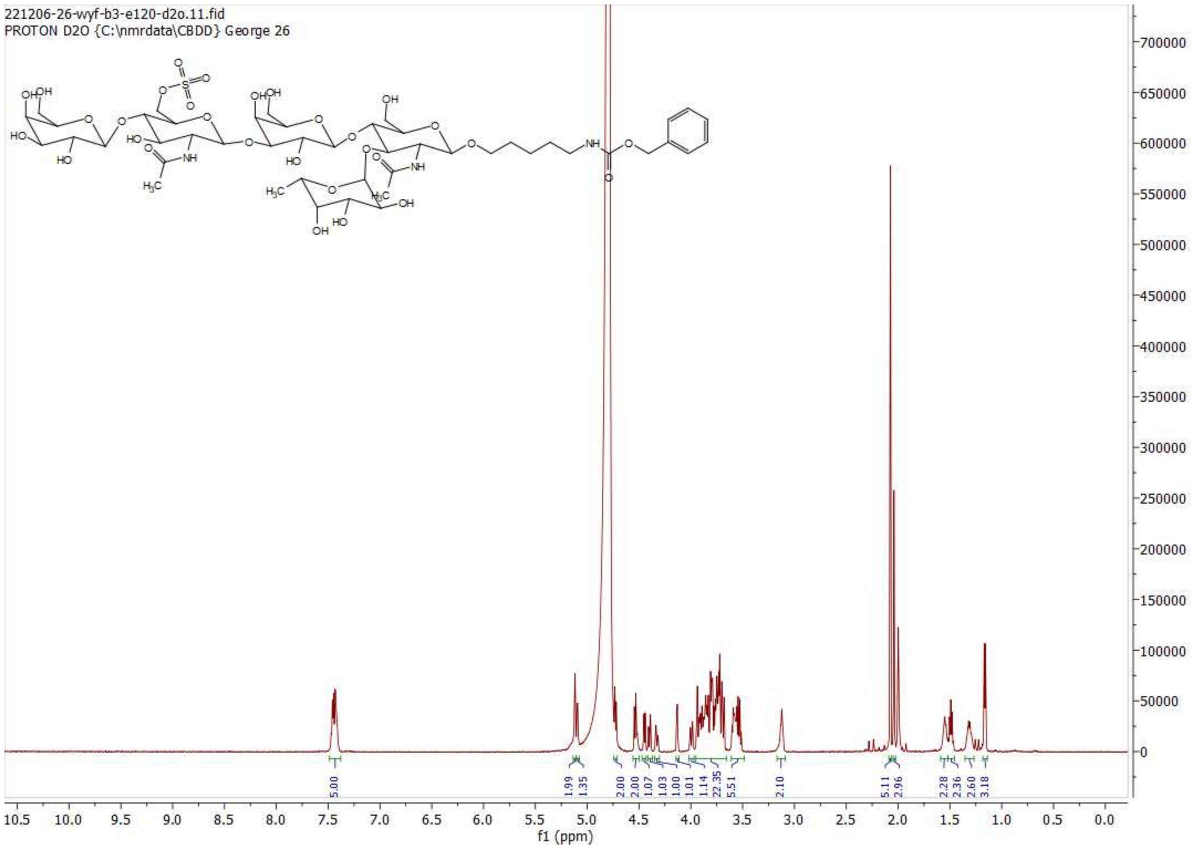

^{1}H NMR of S12; 600MHz; D_2O

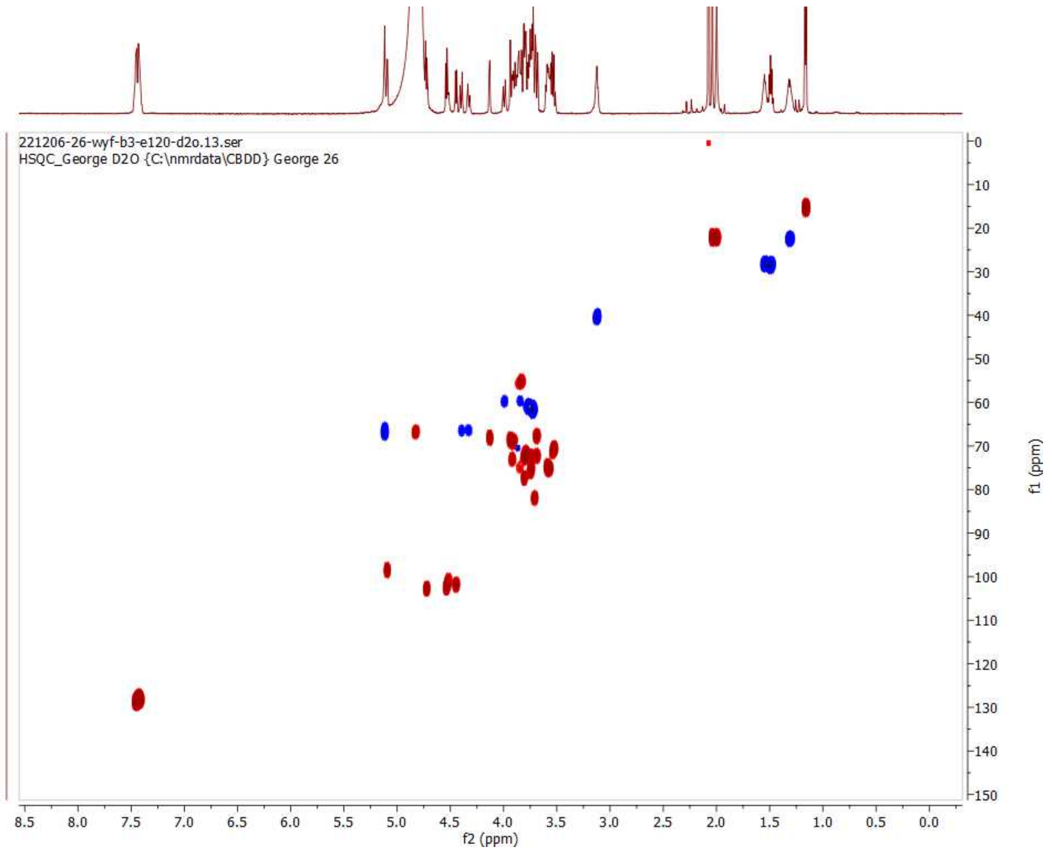

HSQC of S12; 600 MHz/150 MHz, D_2O

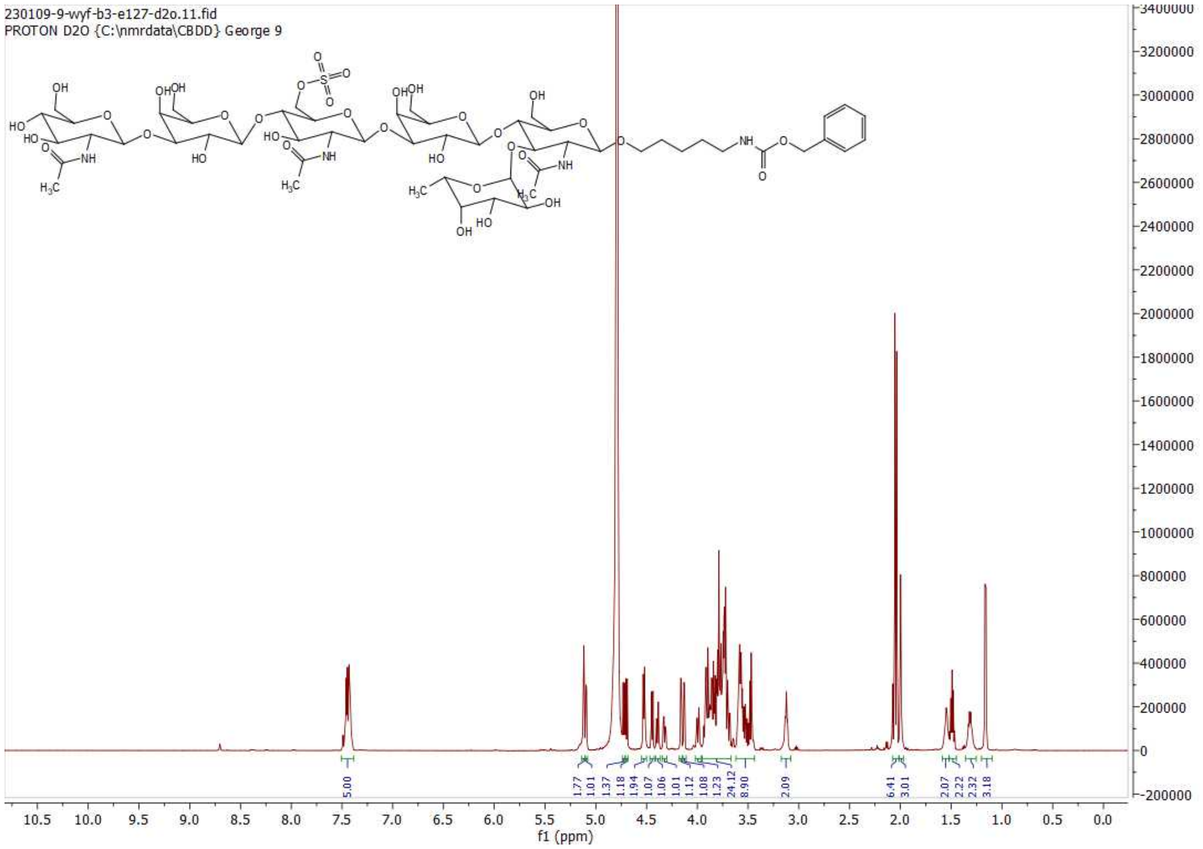

^{1}H NMR of S13; 600MHz; D_2O

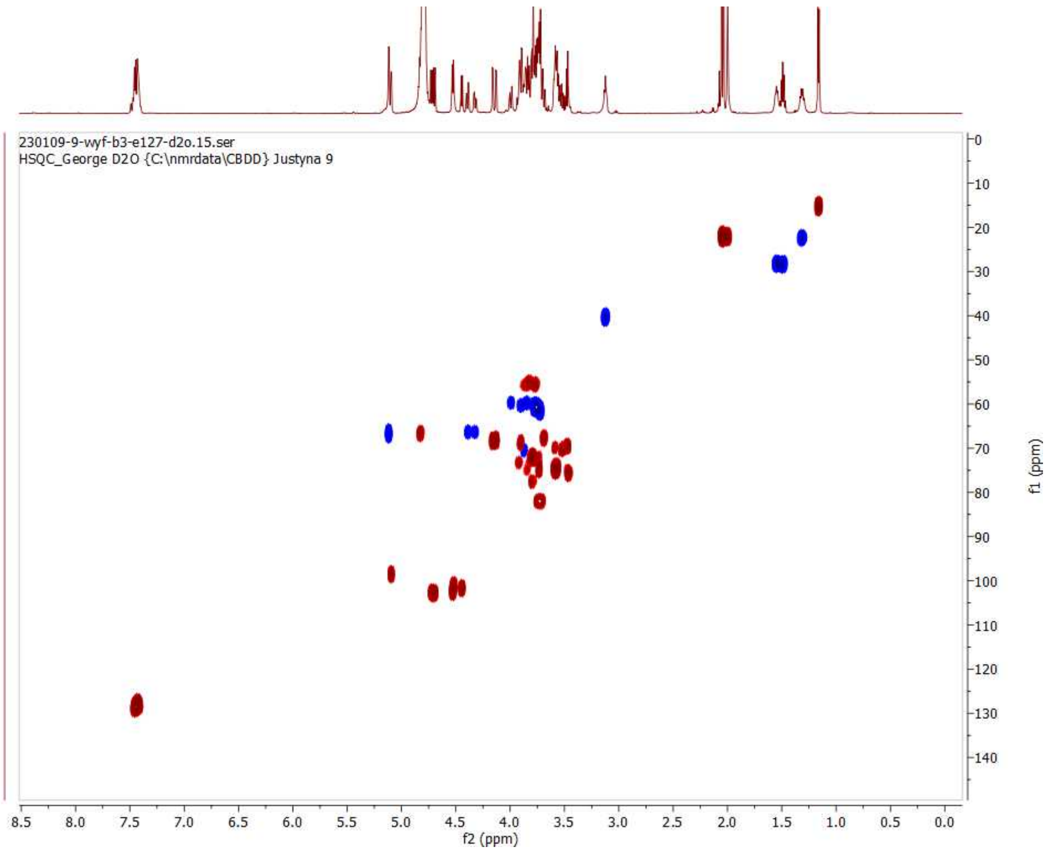

HSQC of S13; 600 MHz/150 MHz, D_2O

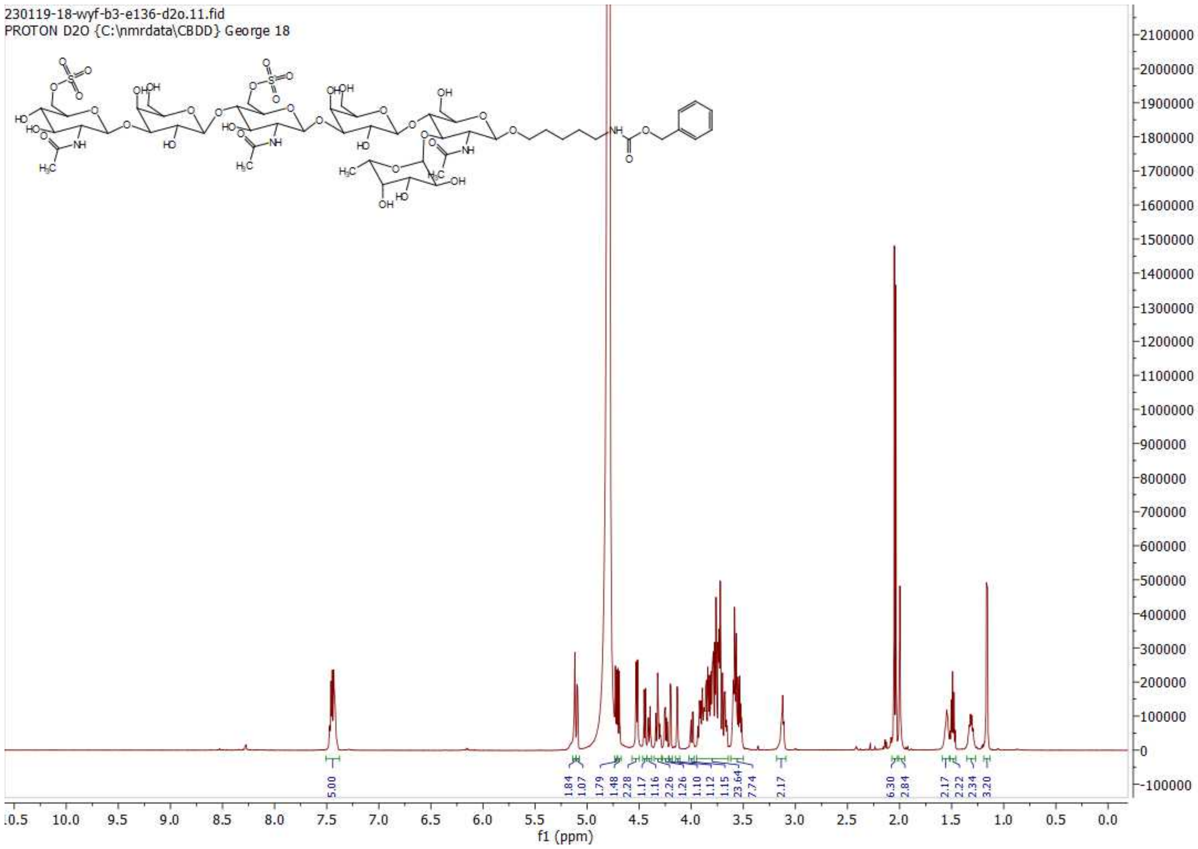

^{1}H NMR of 19; 600MHz; D_2O

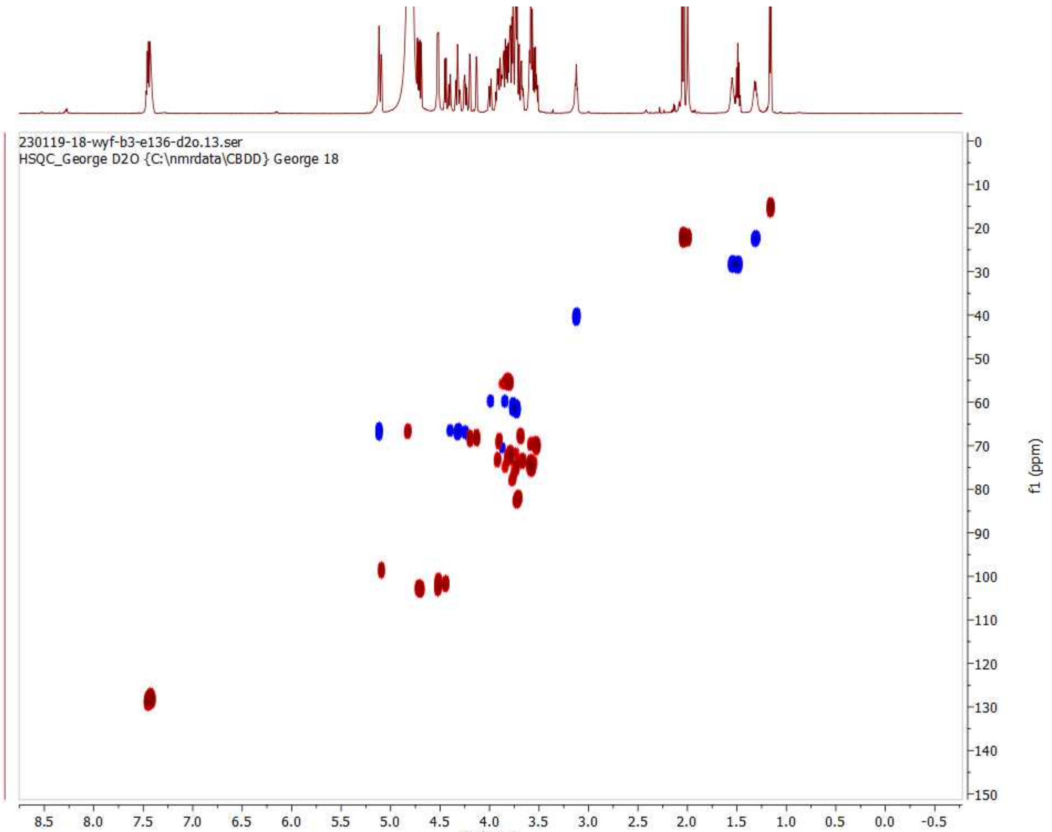

HSQC of 19; 600 MHz/150 MHz, D_2O

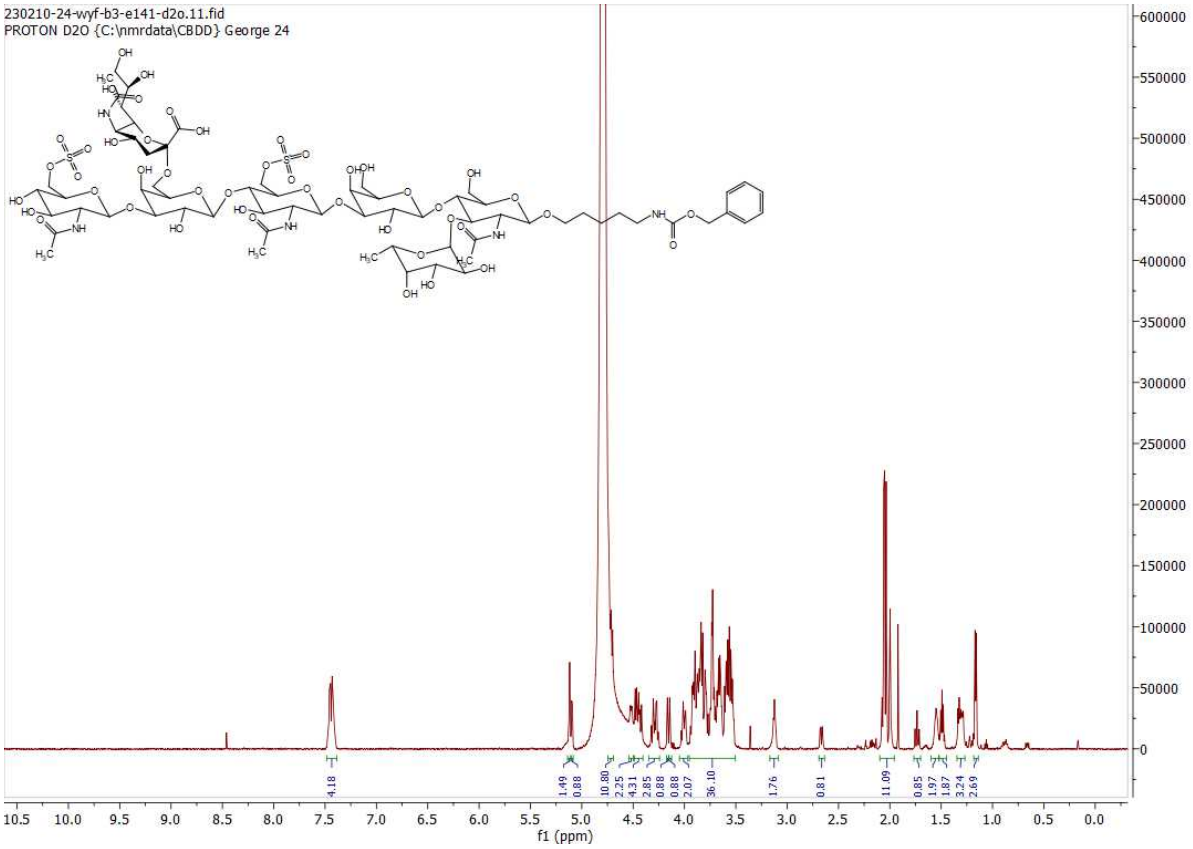

^{1}H NMR of 20; 600MHz; D_2O

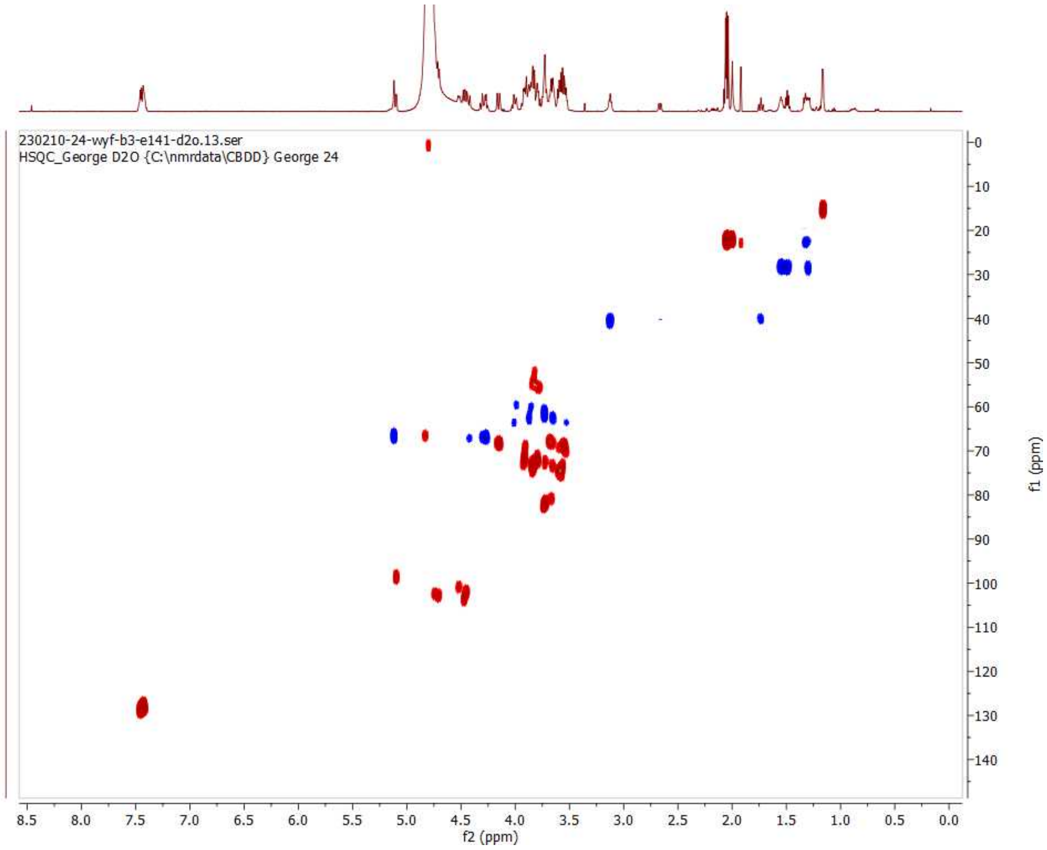

HSQC of 20; 600 MHz/150 MHz, D_2O

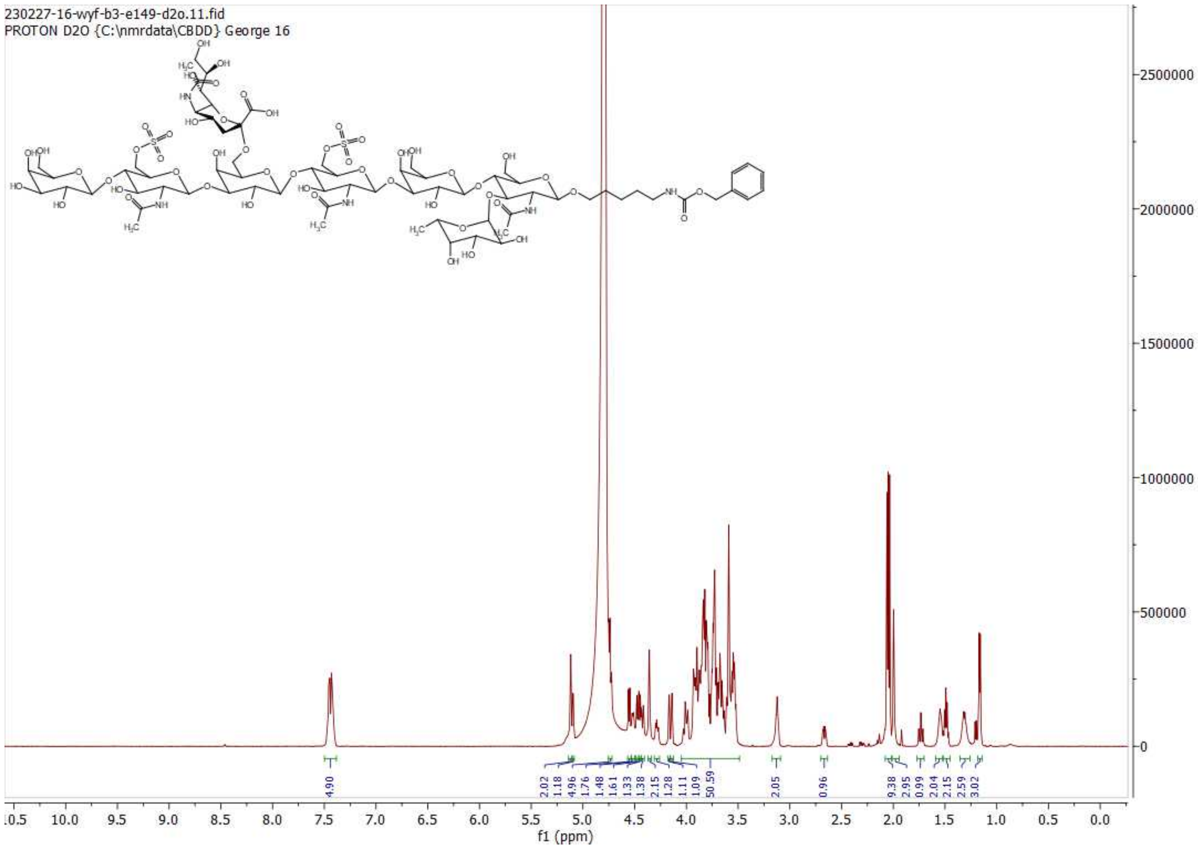

^{1}H NMR of 21; 600MHz; D_2O

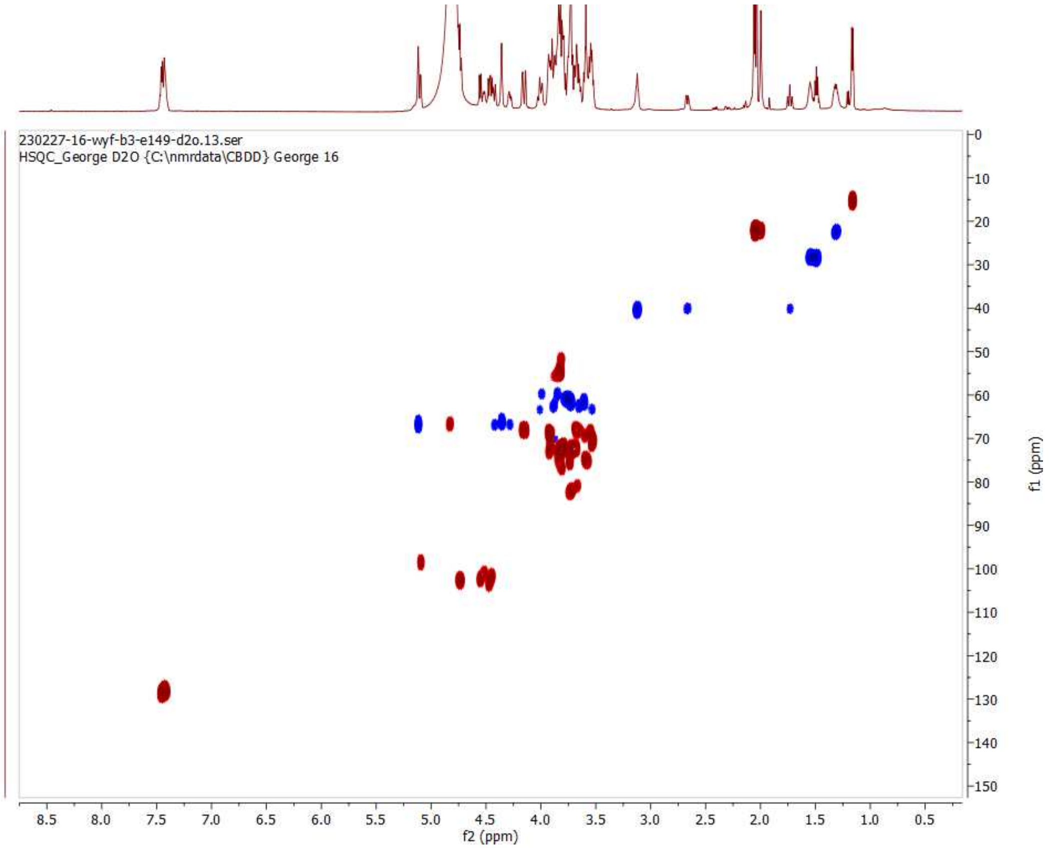

HSQC of 21; 600 MHz/150 MHz, D_2O

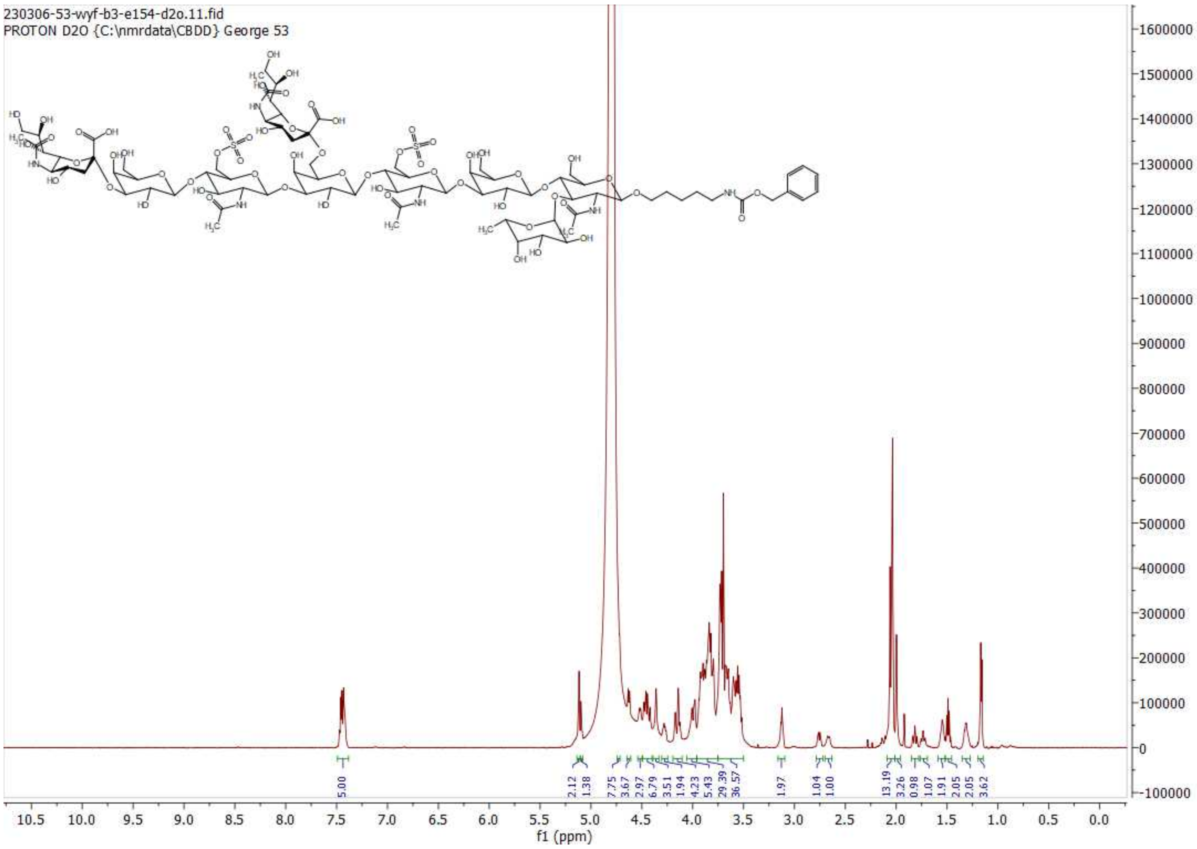

^{1}H NMR of 22; 600MHz; D_2O

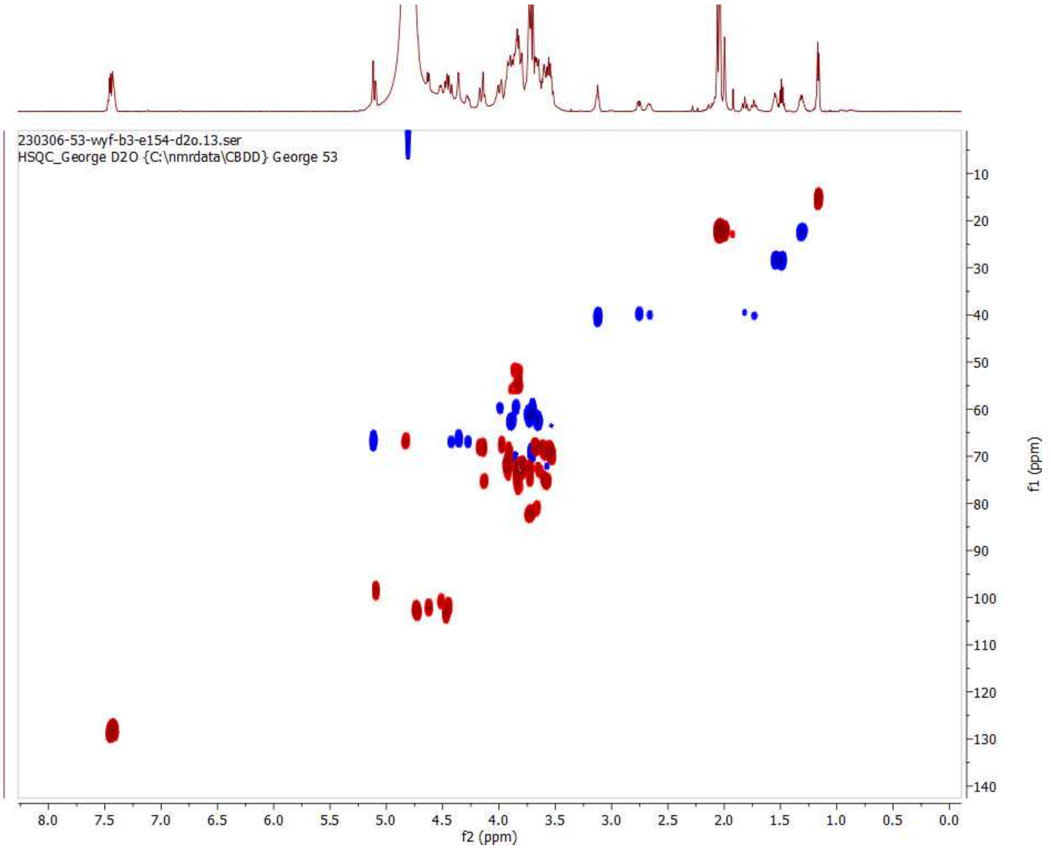

HSQC of 22; 600 MHz/150 MHz, D_2O

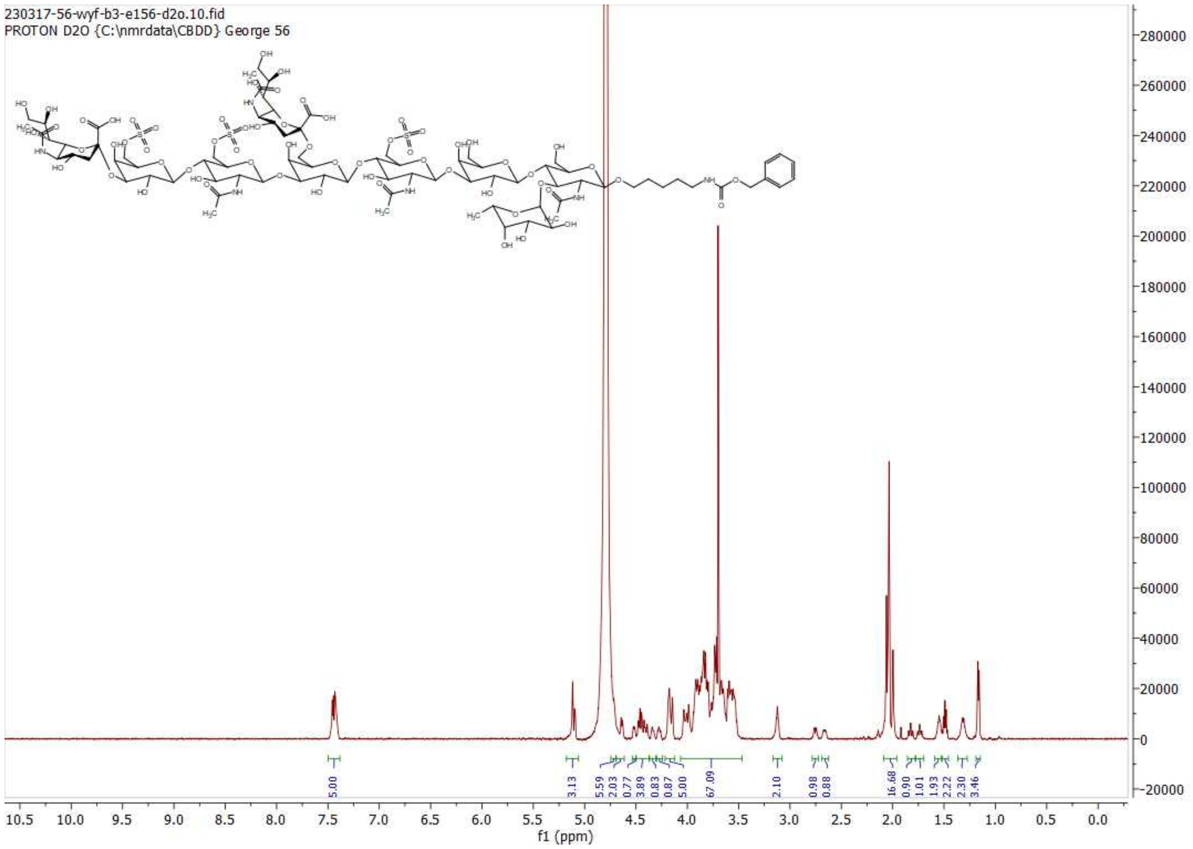

^{1}H NMR of 23; 600MHz; D_2O

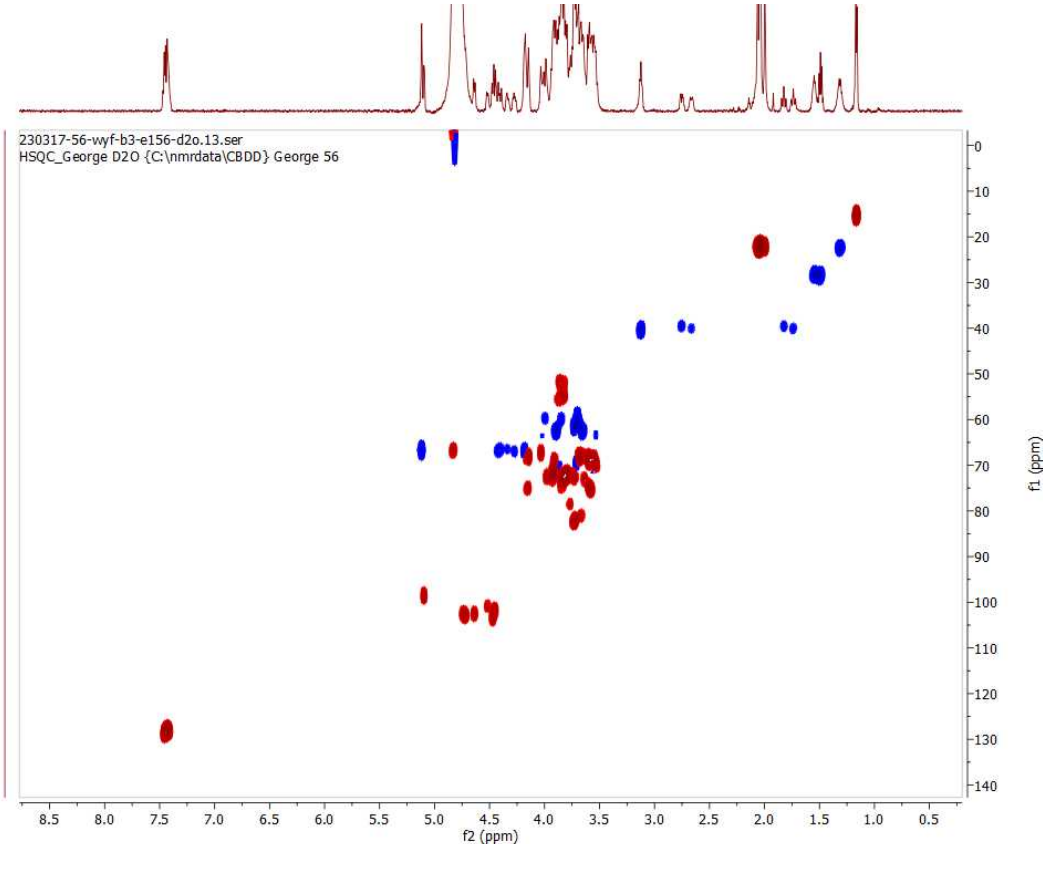

HSQC of 23; 600 MHz/150 MHz, D_2O

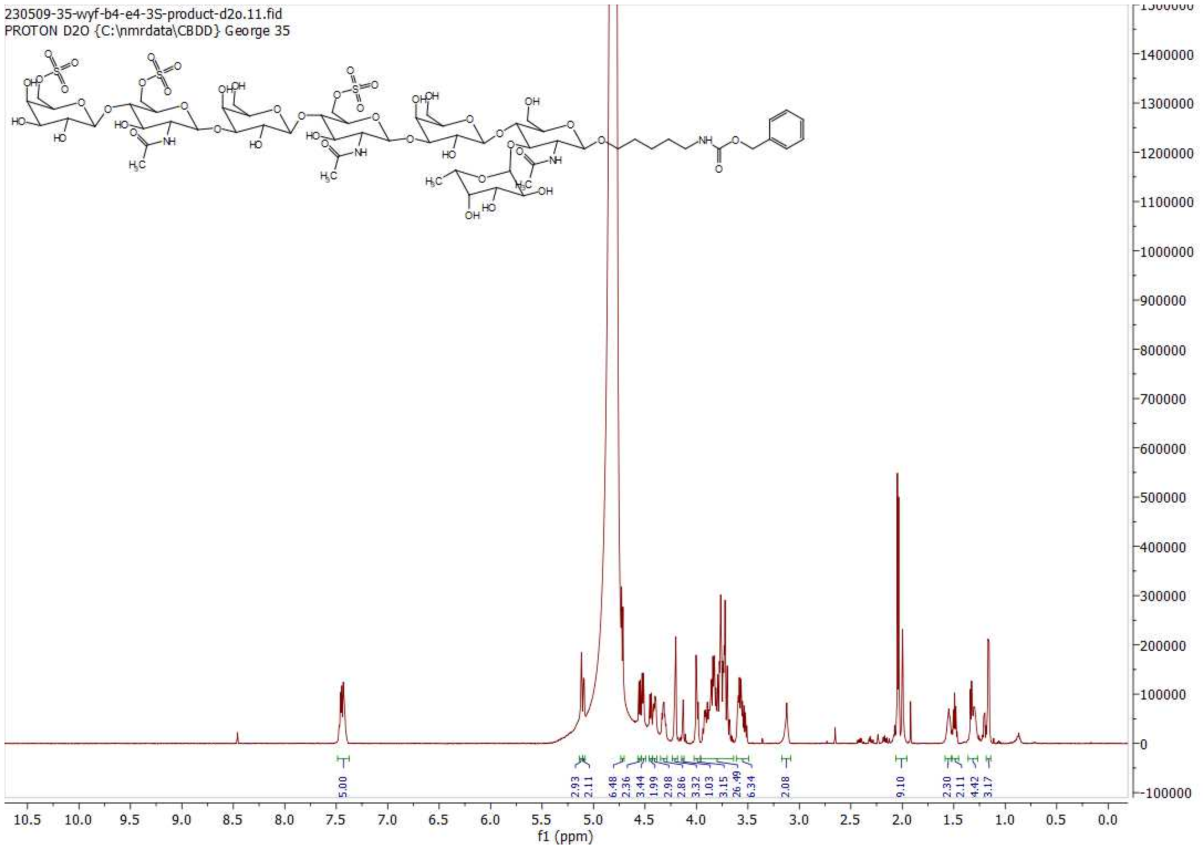

^{1}H NMR of S16; 600MHz; D_2O

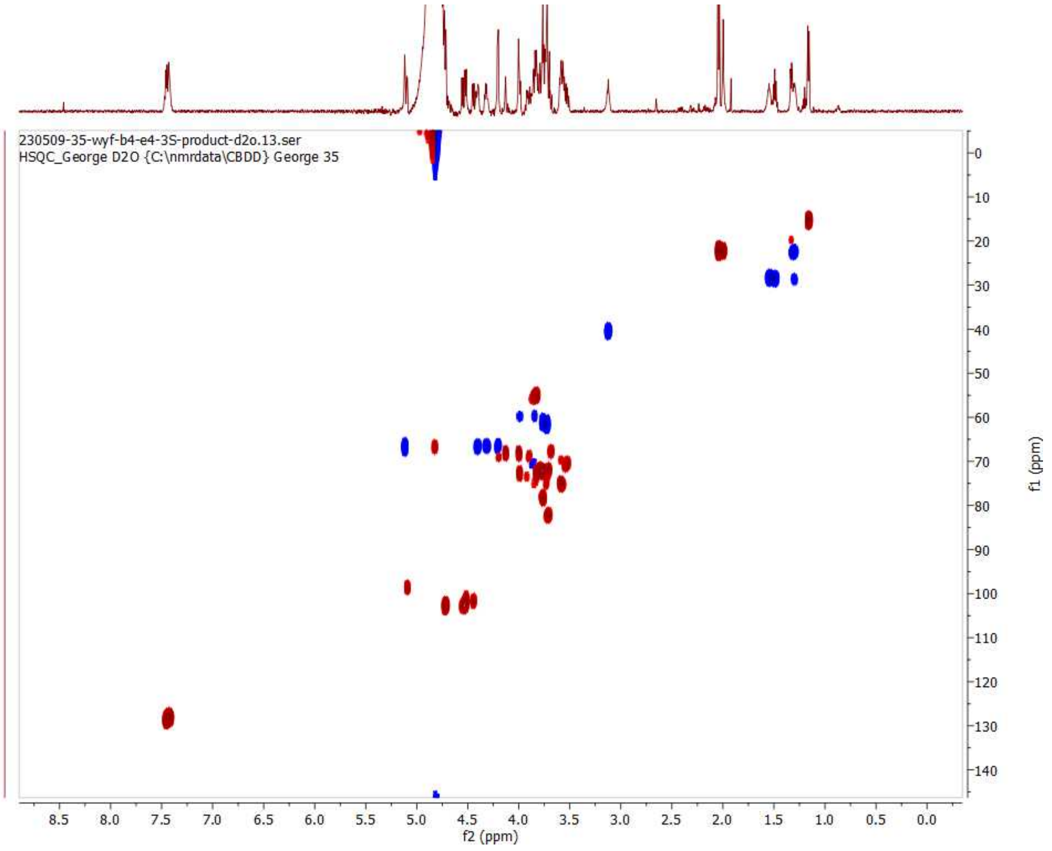

HSQC of S16; 600 MHz/150 MHz, D_2O

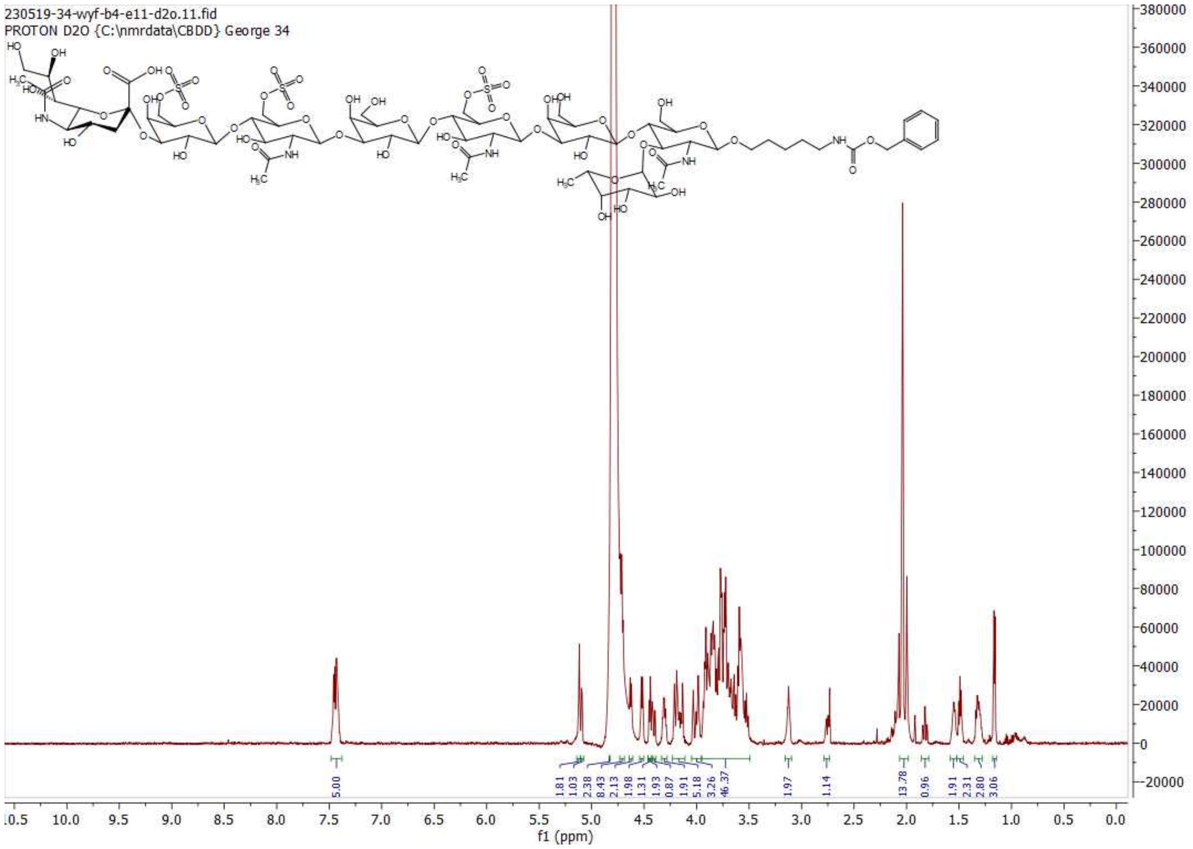

^{1}H NMR of 24; 600MHz; D_2O

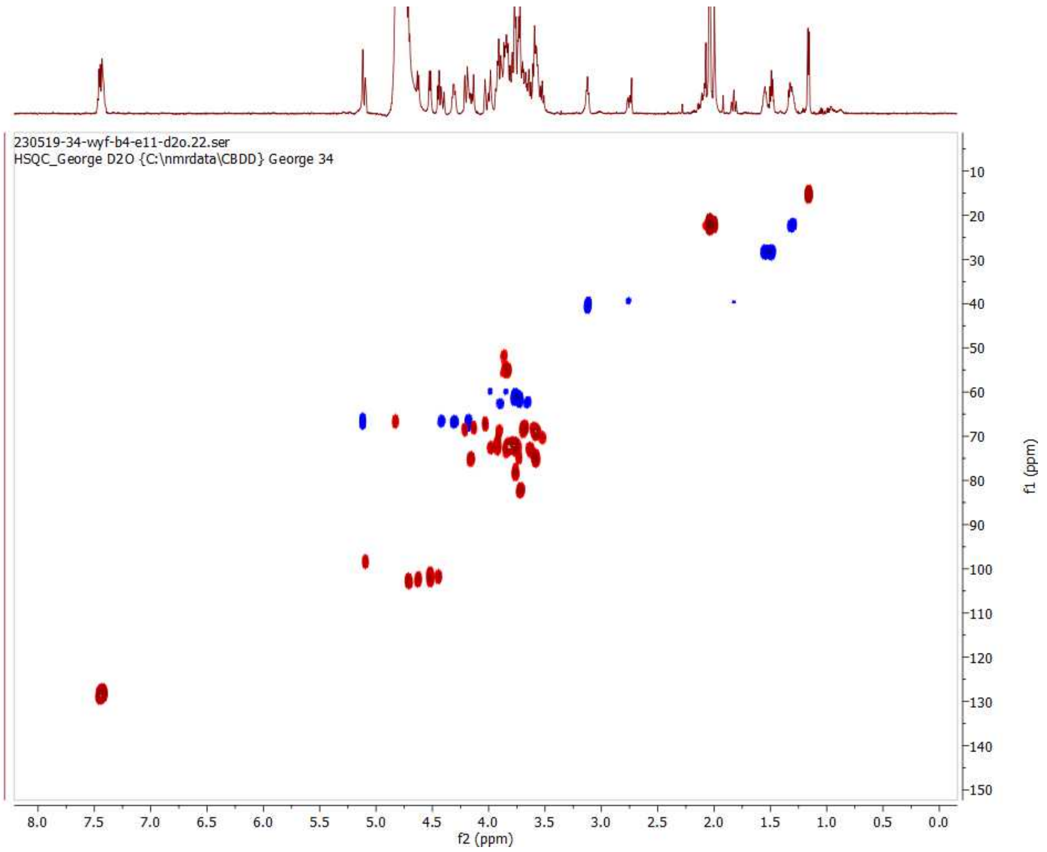

HSQC of 24; 600 MHz/150 MHz, D_2O

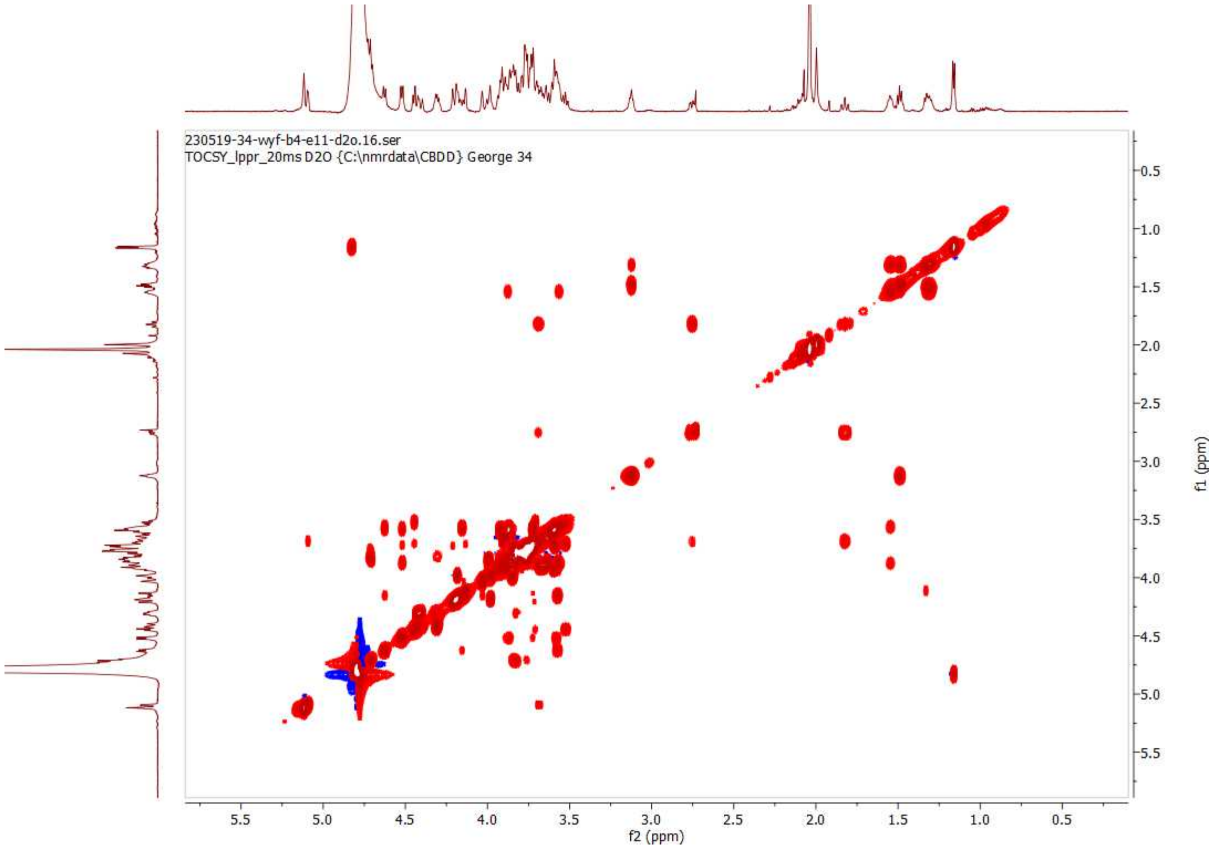

TOCSY (20 ms) of 24; 600MHz; D_2O

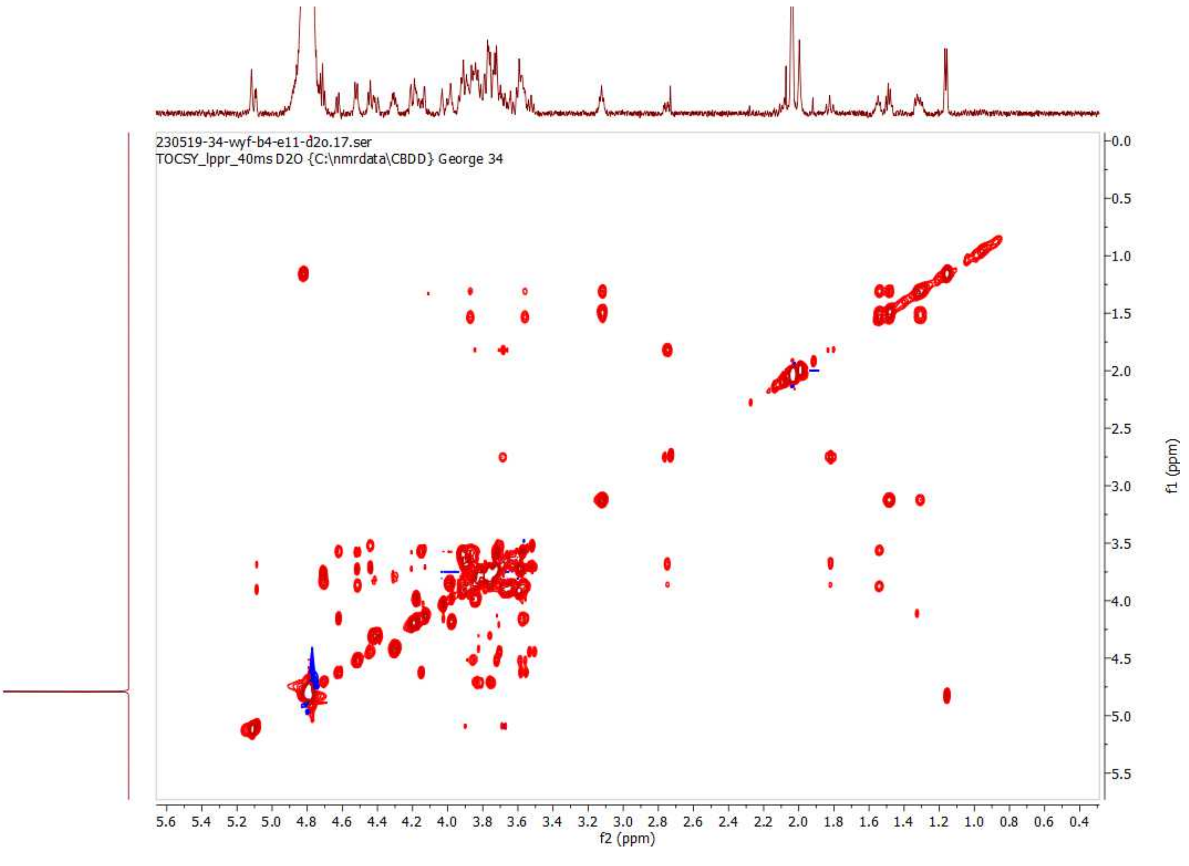

TOCSY (40 ms) of 24; 600MHz; D_2O

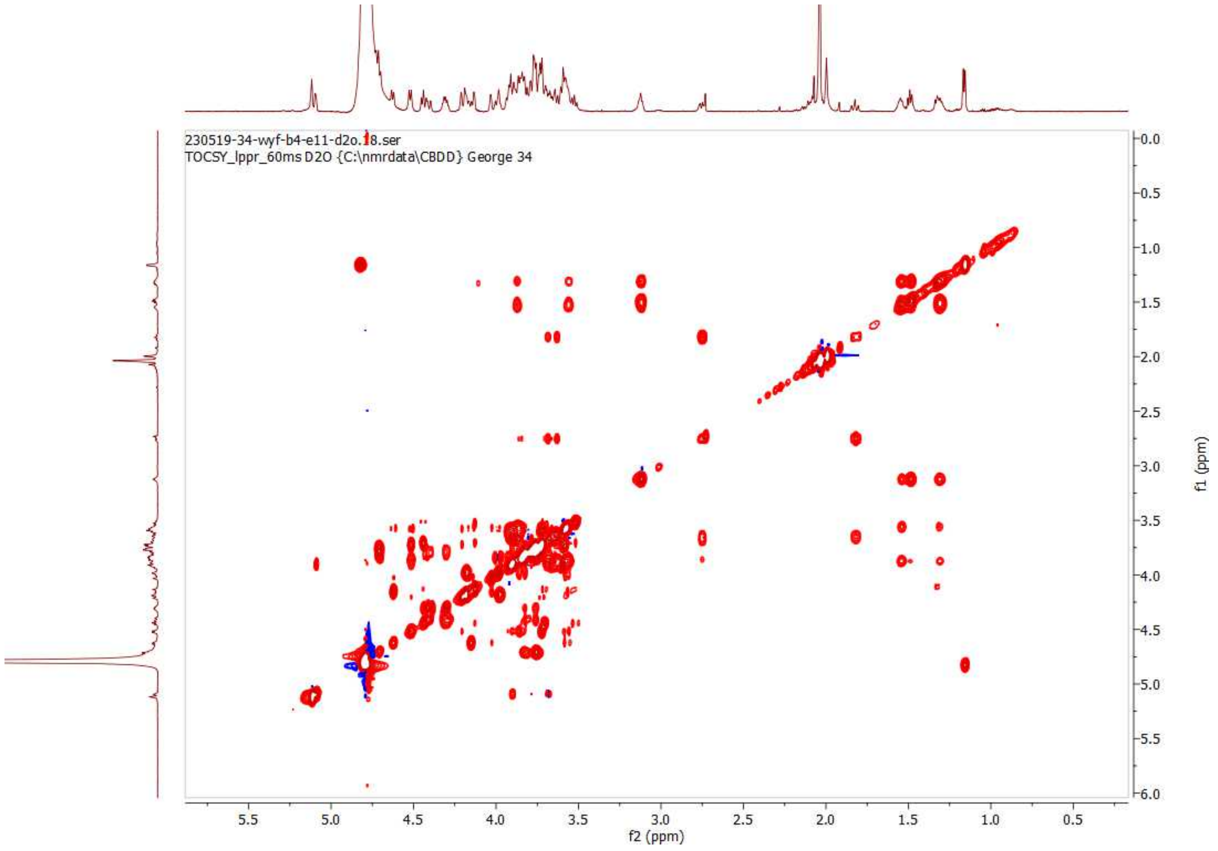

TOCSY (60 ms) of 24; 600MHz; D$_2$O

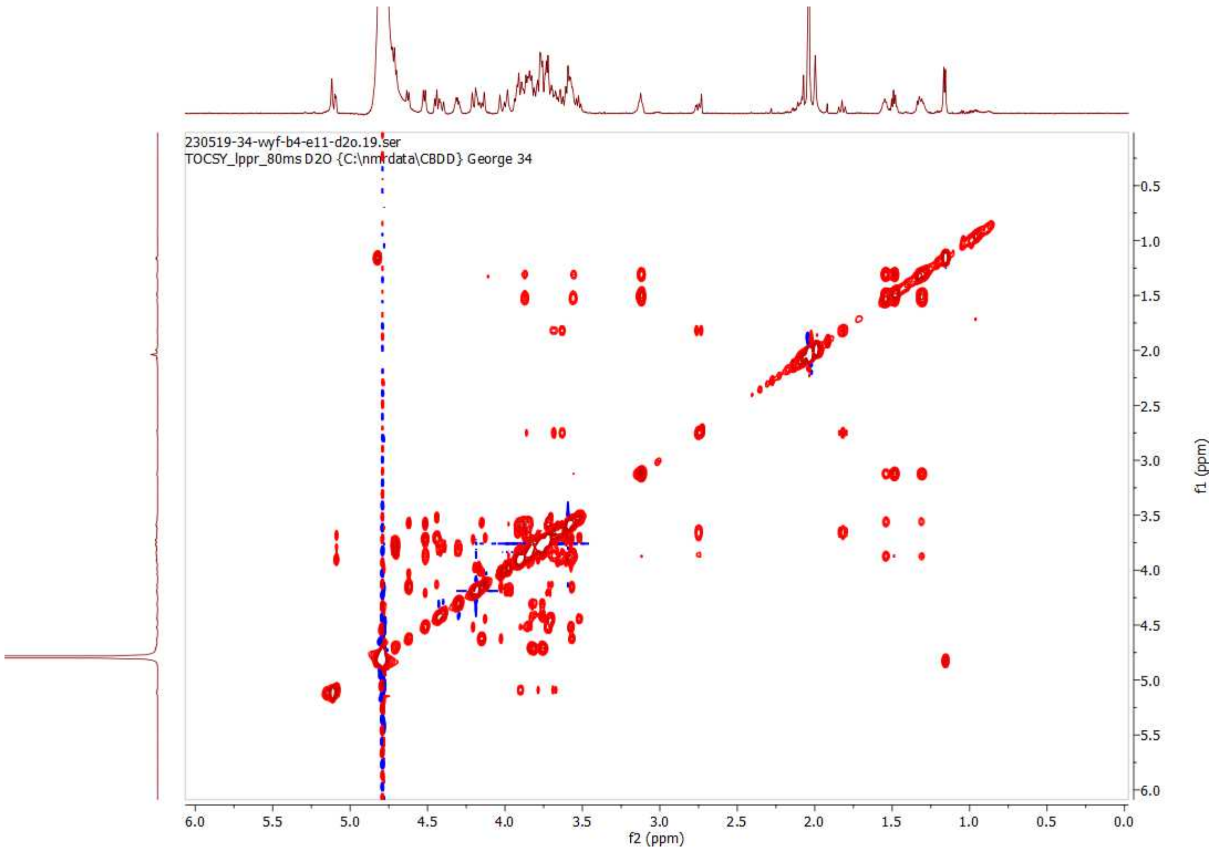

TOCSY (80 ms) of 24; 600MHz; D$_2$O

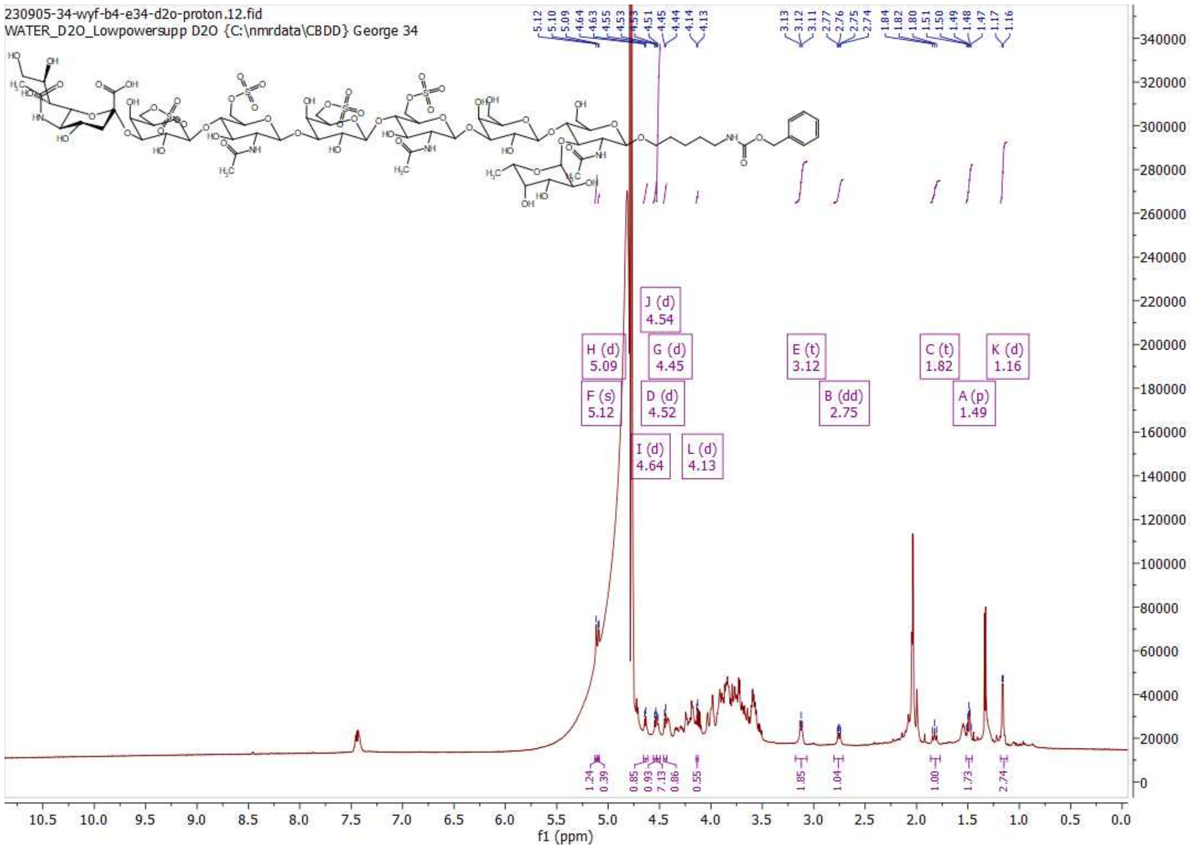

¹H NMR of 25; 600MHz; D₂O

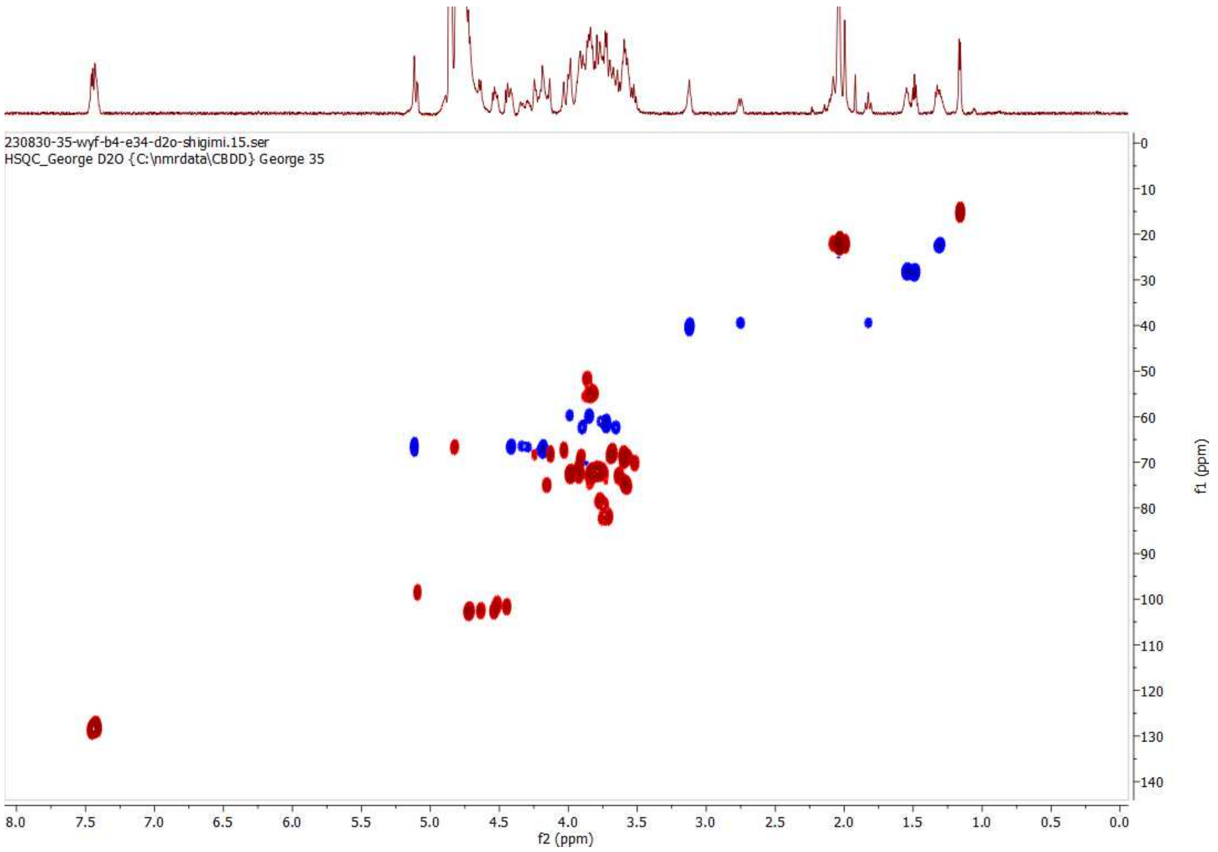

HSQC of 25; 600 MHz/150 MHz, D₂O

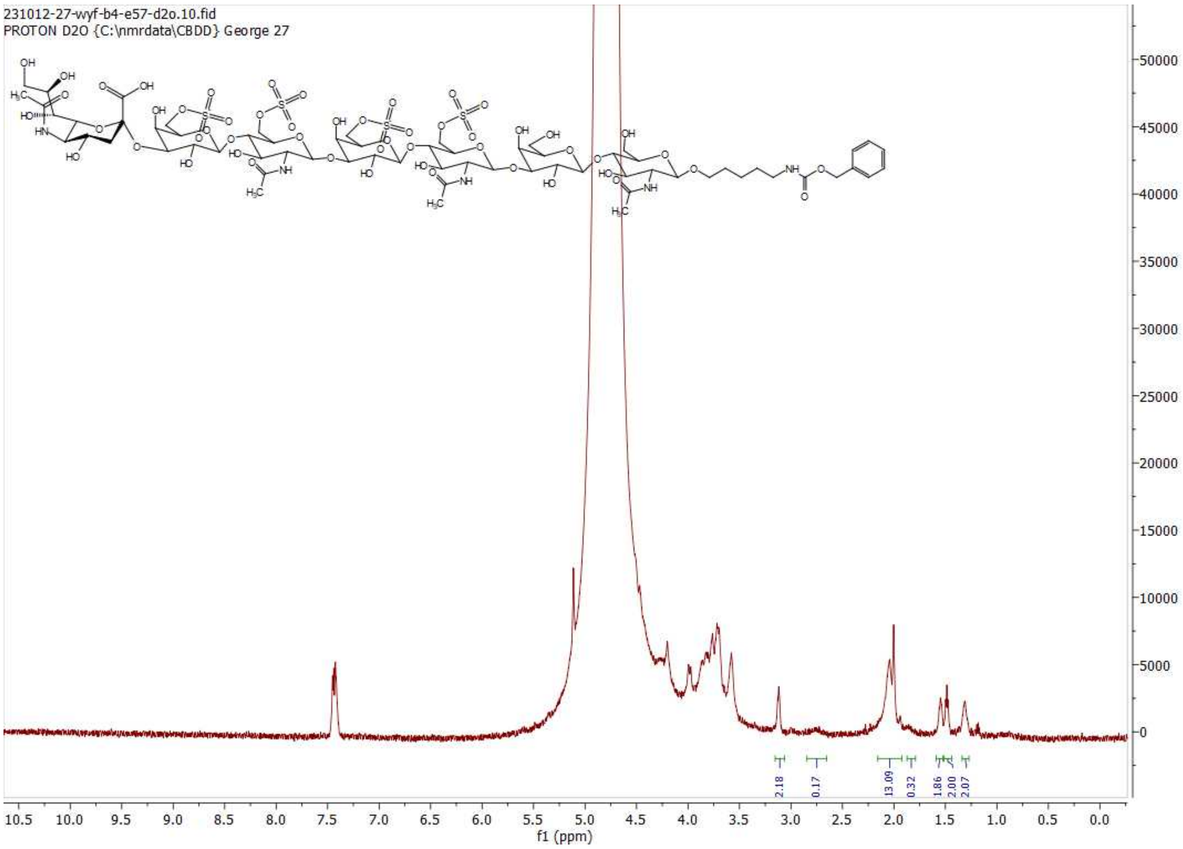

^{1}H NMR of 26; 600MHz; D_2O

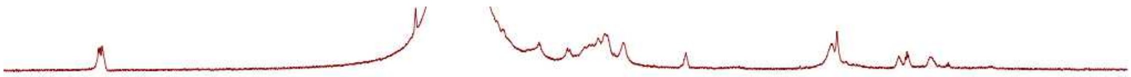

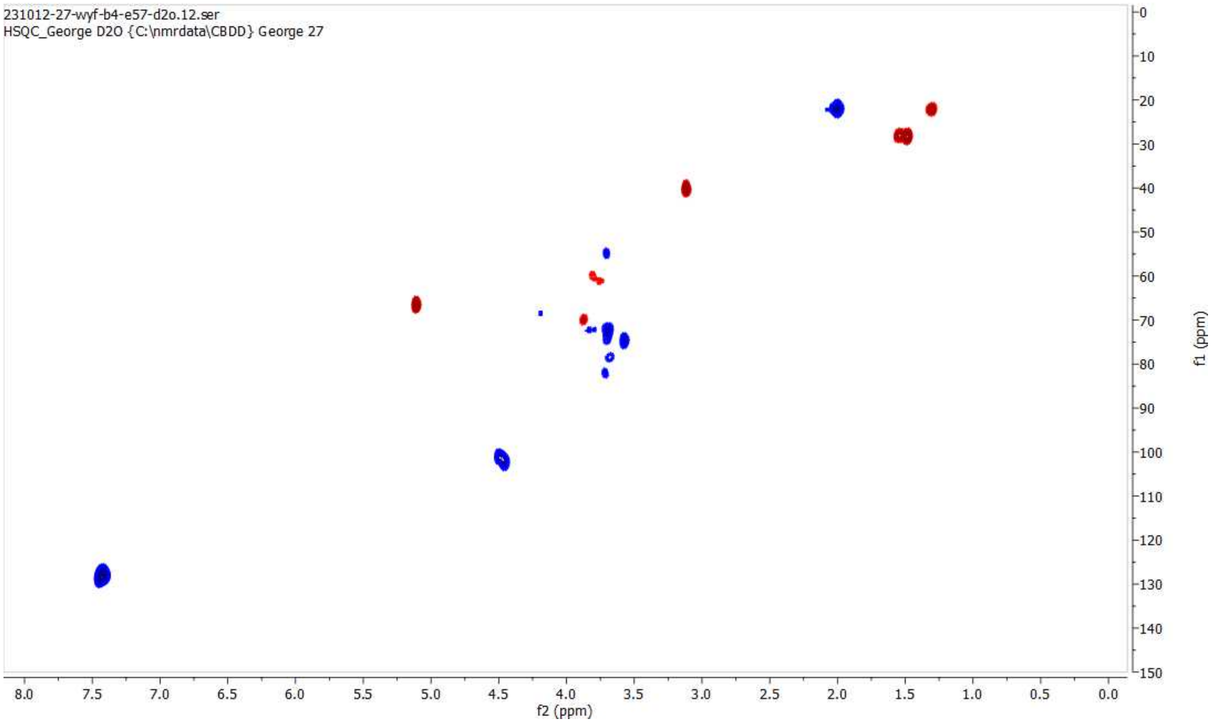

HSQC of 26; 600 MHz/150 MHz, D_2O

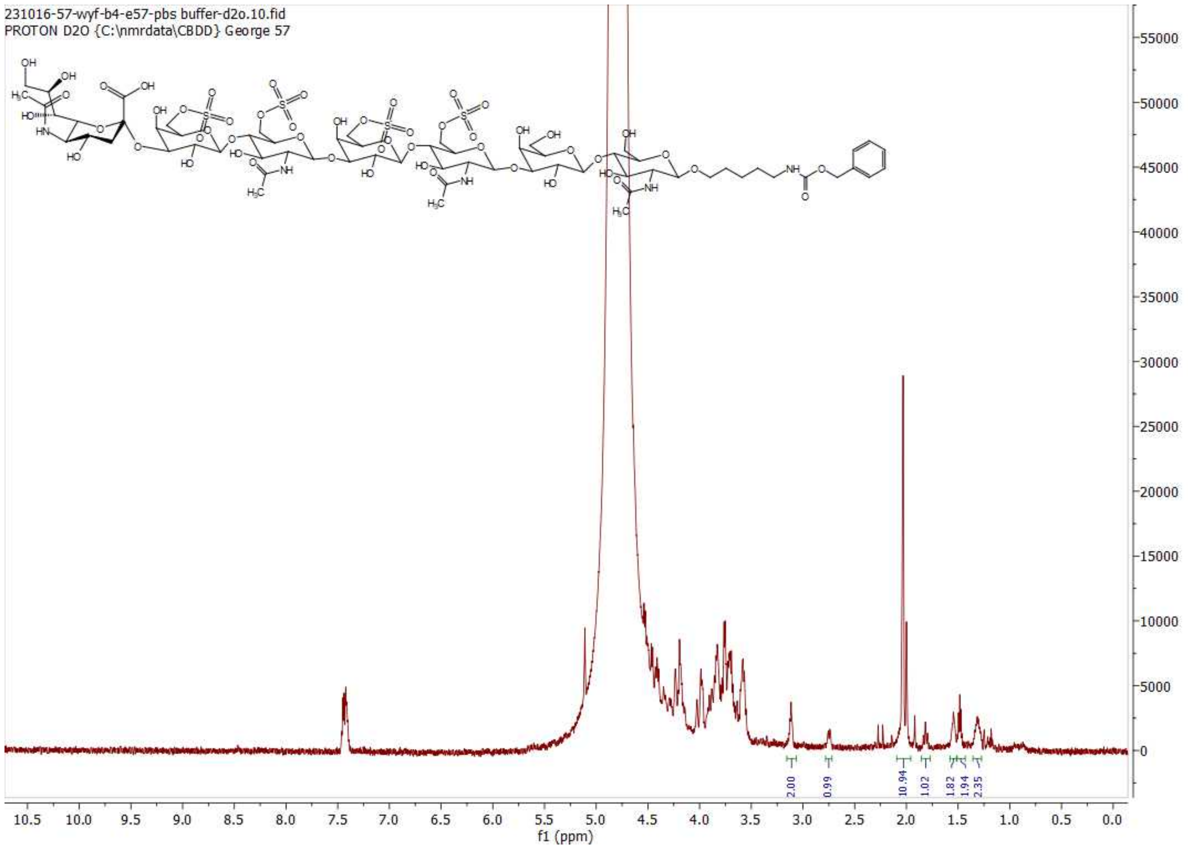

^{1}H NMR of 26; 600MHz; PBS D_2O buffer (40 mM, pH=6.5)

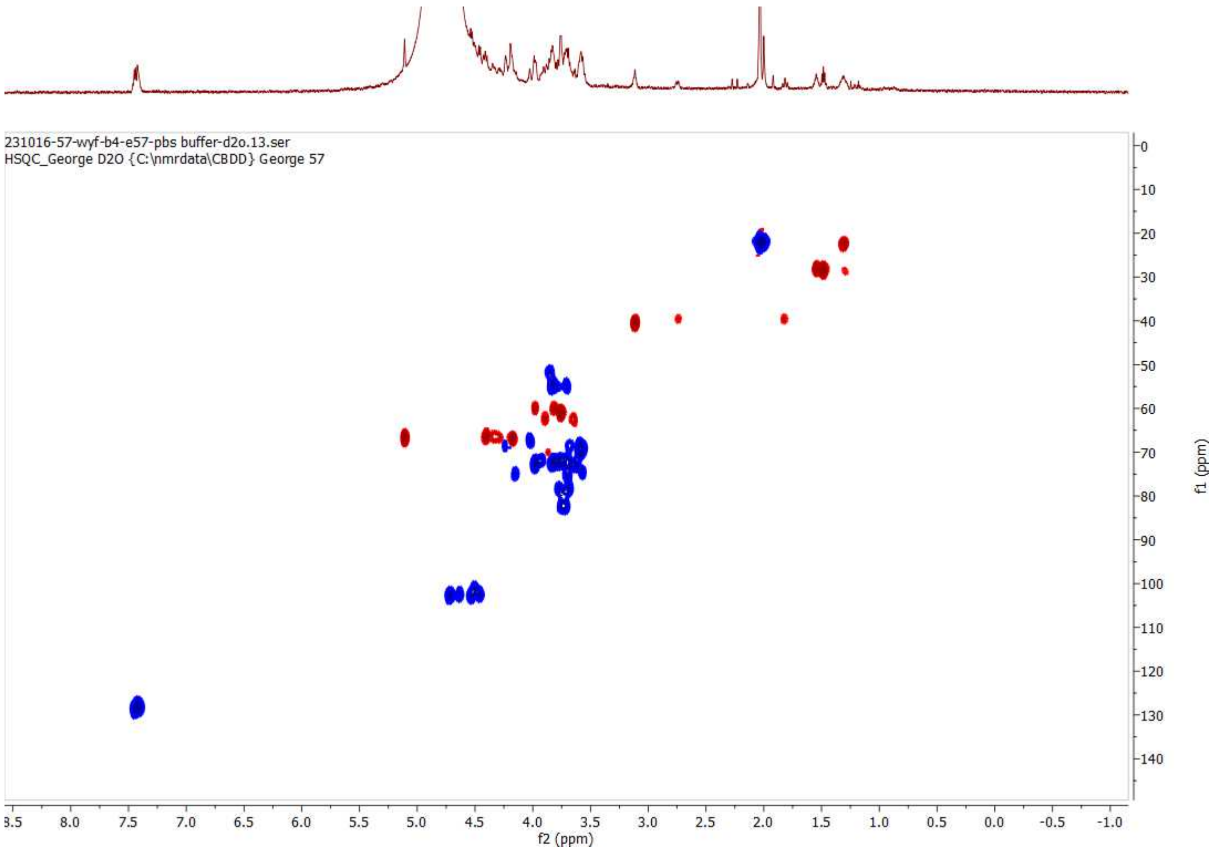

HSQC of 26; 600 MHz/150 MHz, PBS D_2O buffer (40 mM, pH=6.5)

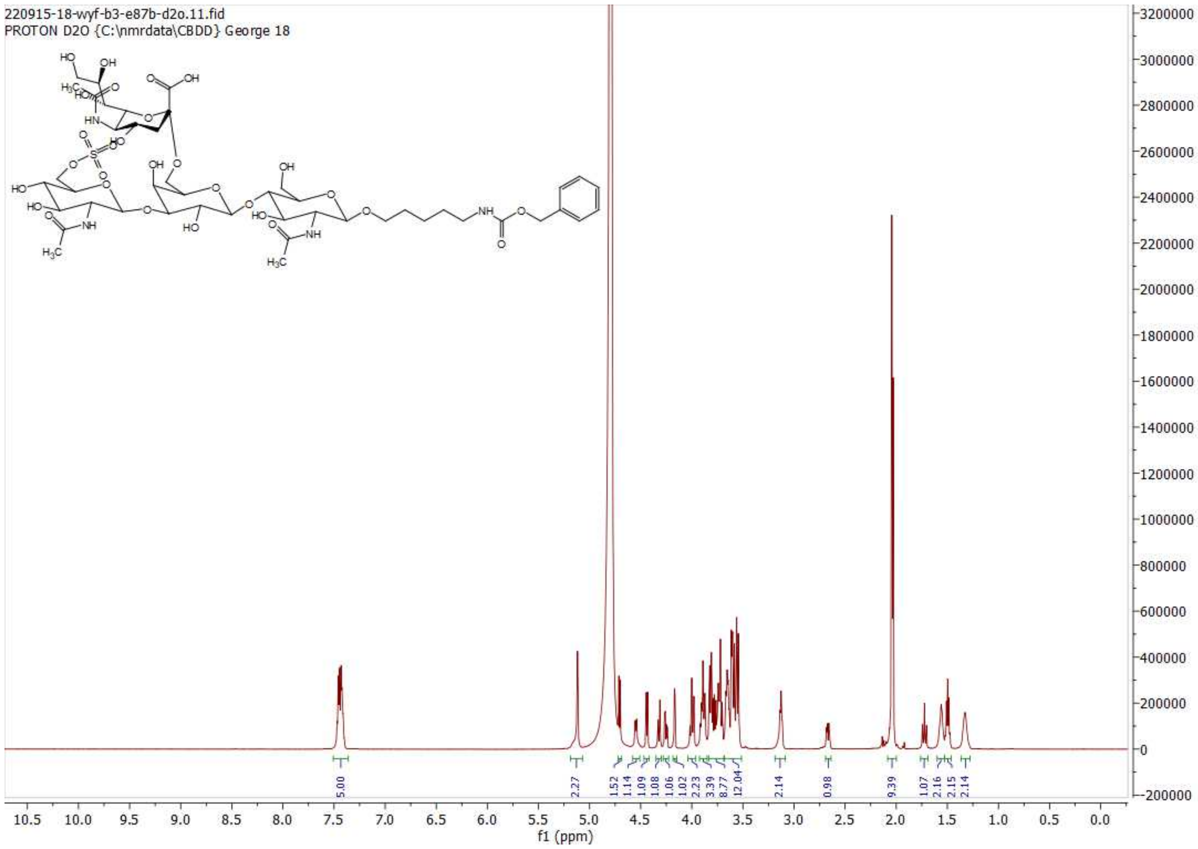

^{1}H NMR of 28; 600MHz; D_2O

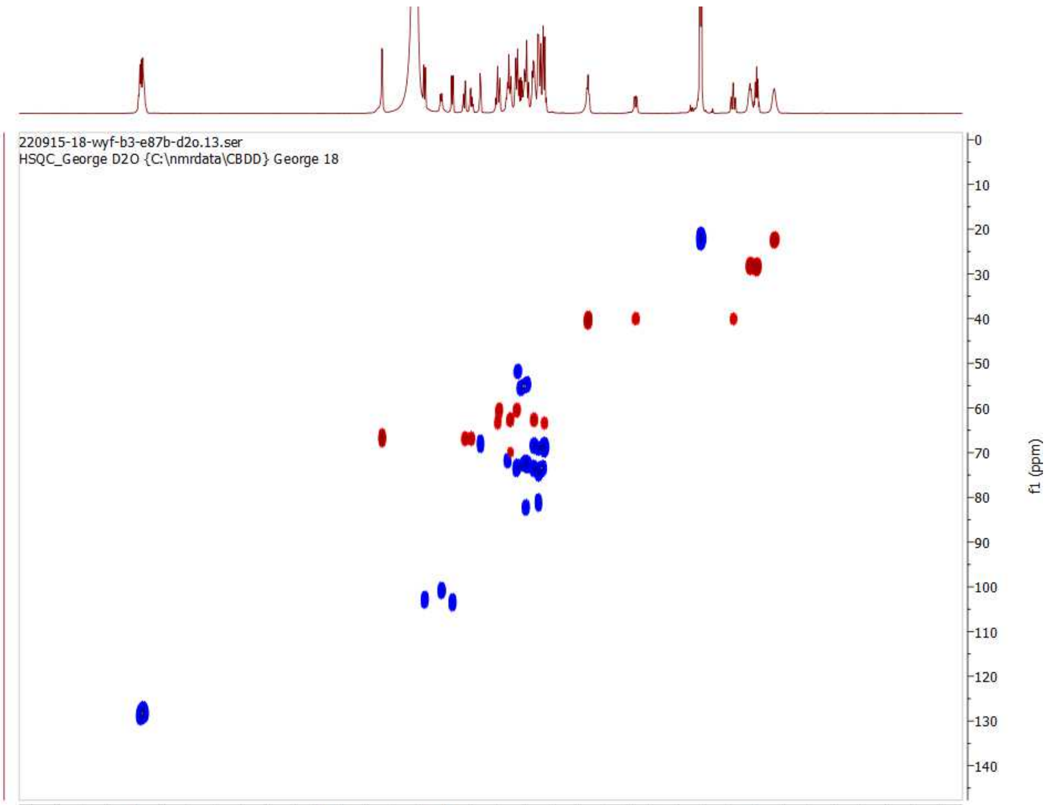

HSQC of 28; 600 MHz/150 MHz, D_2O

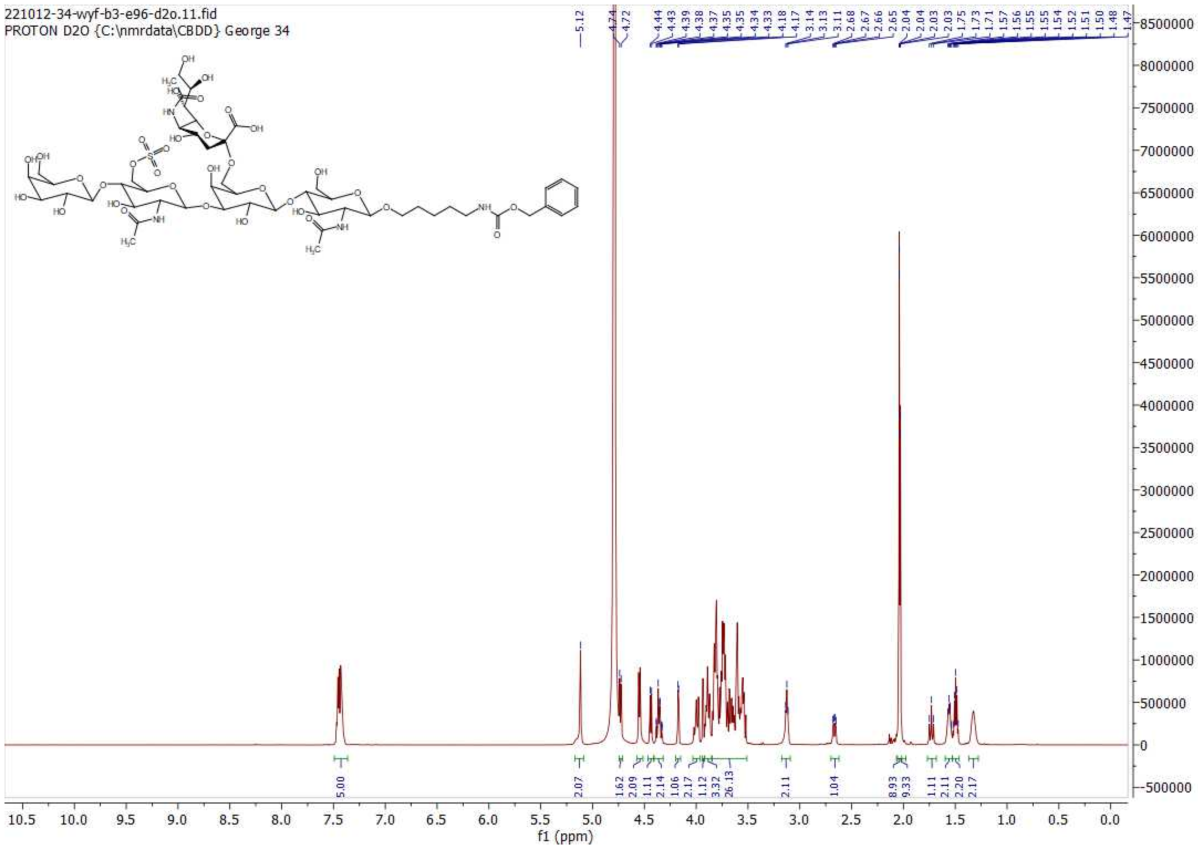

^{1}H NMR of 29; 600MHz; D_2O

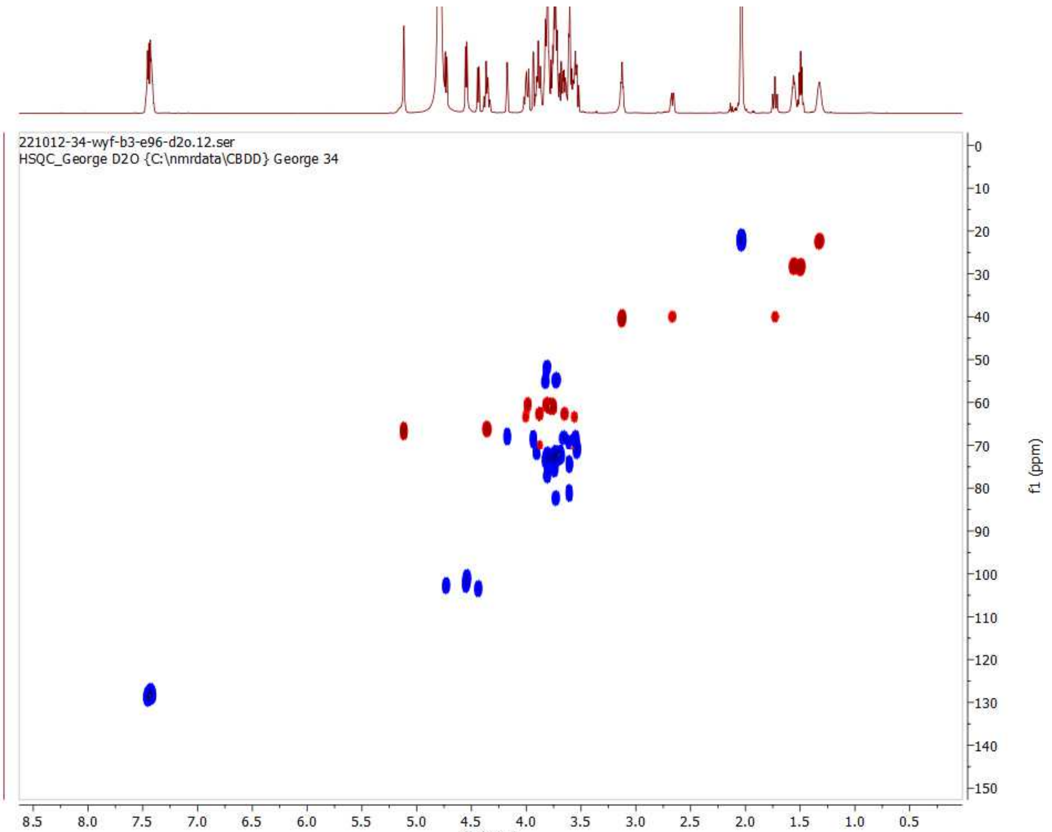

HSQC of 29; 600 MHz/150 MHz, D_2O

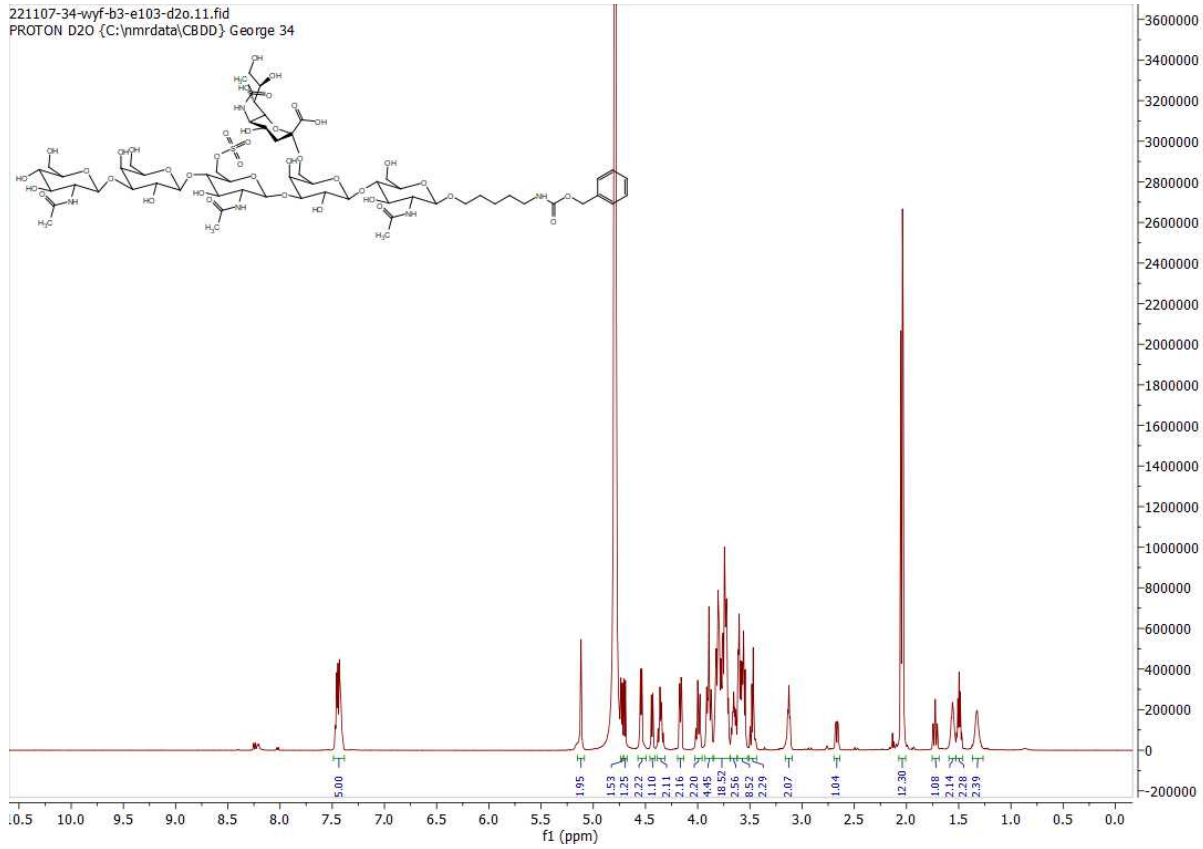

^{1}H NMR of 30; 600MHz; D_2O

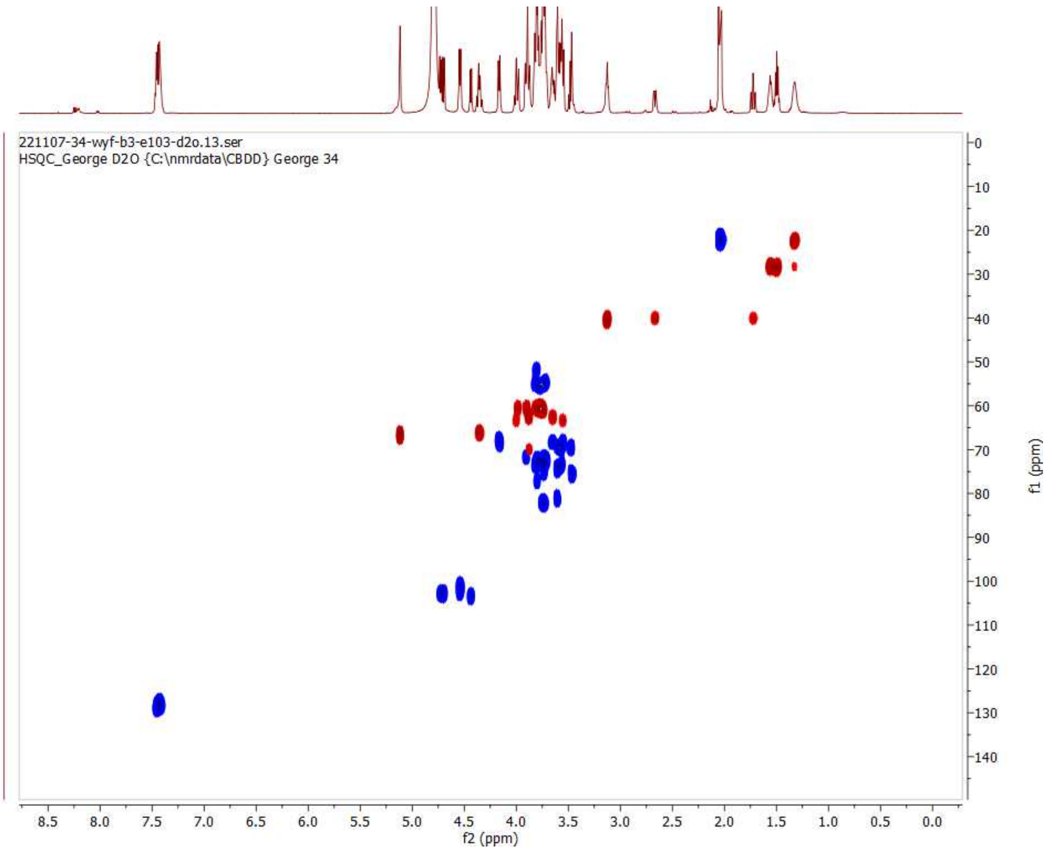

HSQC of 30; 600 MHz/150 MHz, D_2O

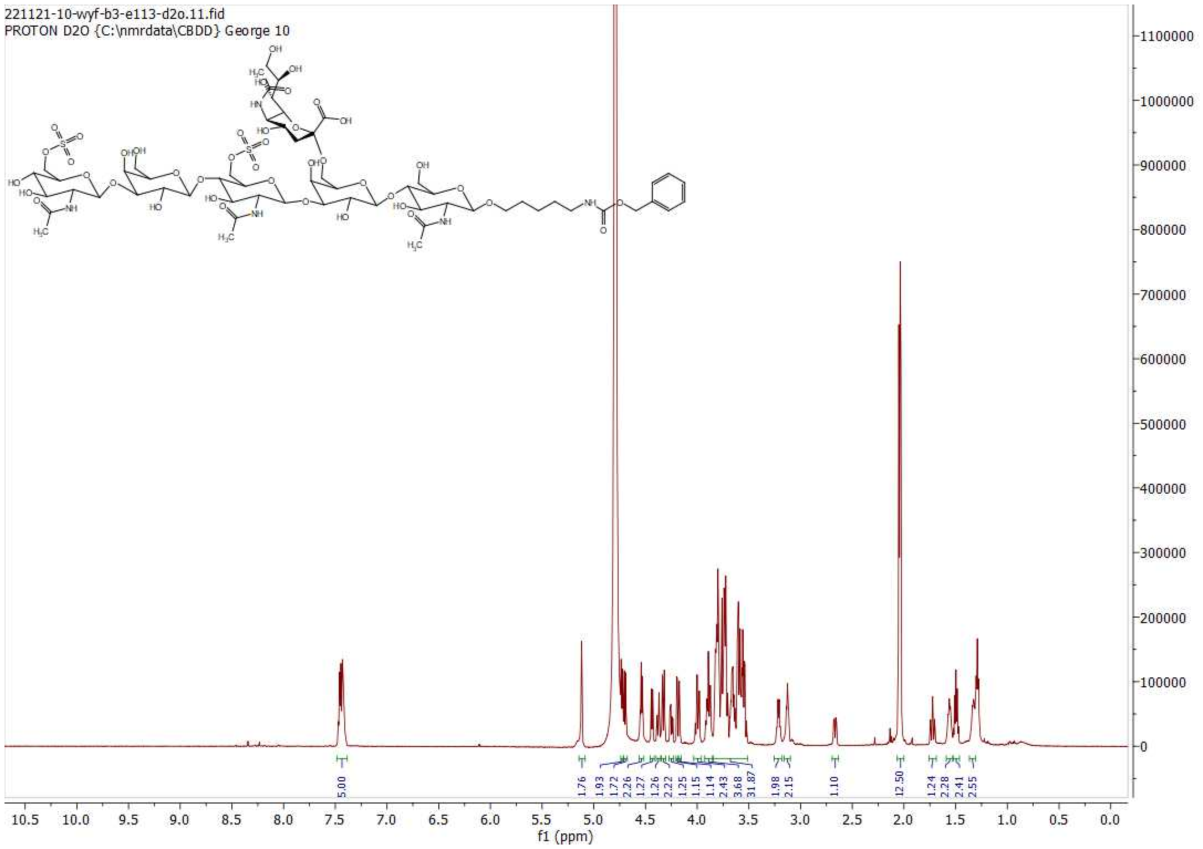

^{1}H NMR of 31; 600MHz; D_2O

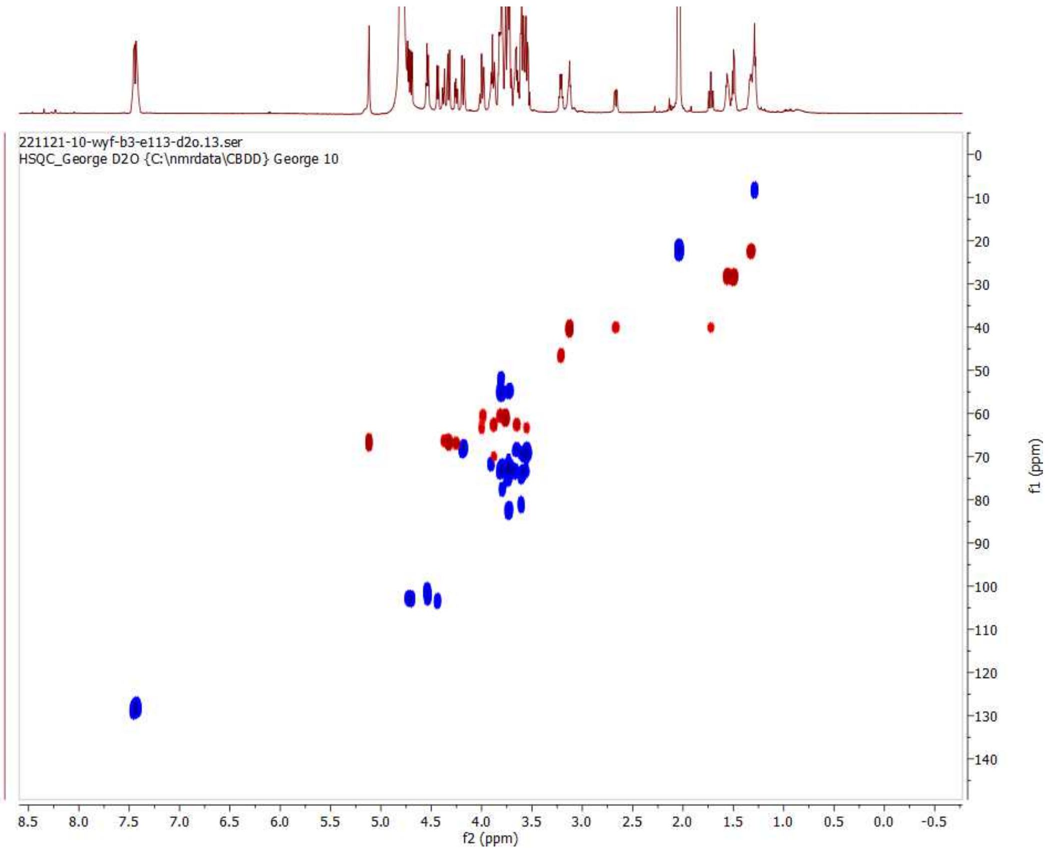

HSQC of 31; 600 MHz/150 MHz, D_2O

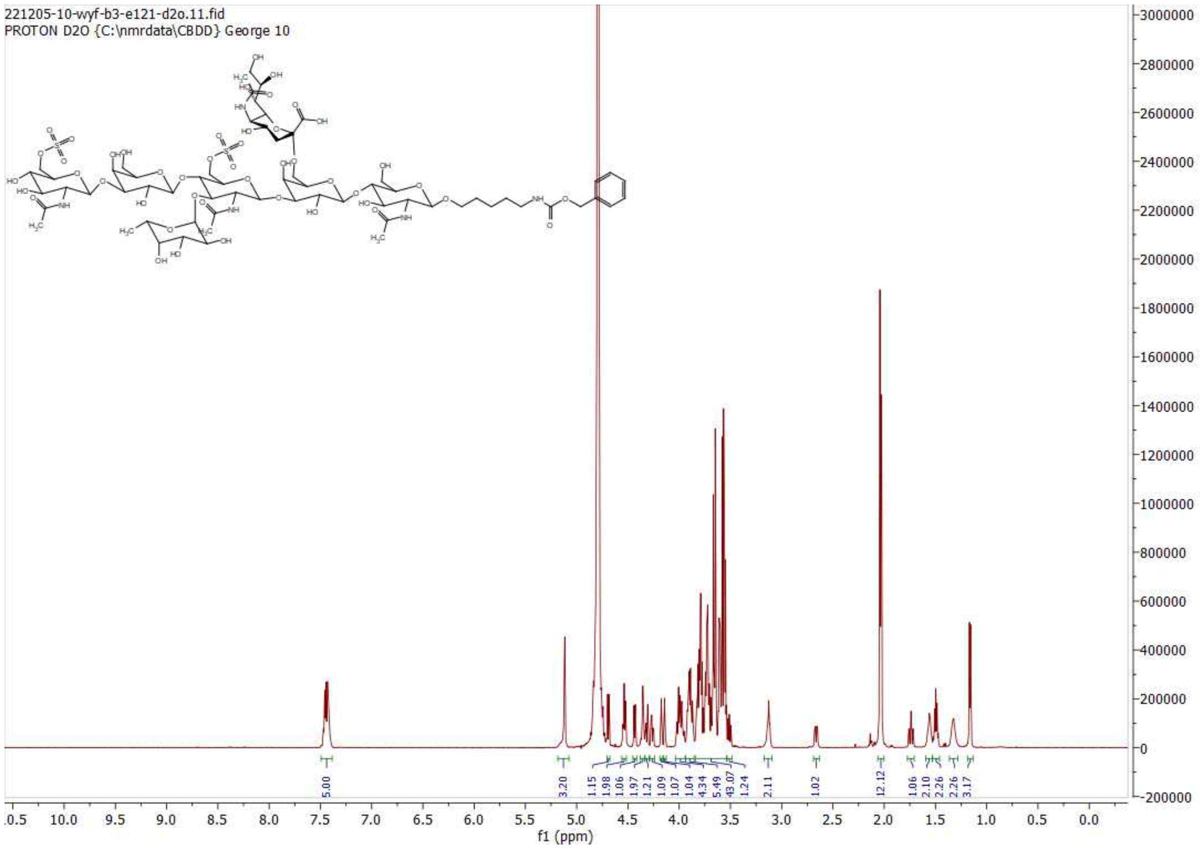

^{1}H NMR of 32; 600MHz; D_2O

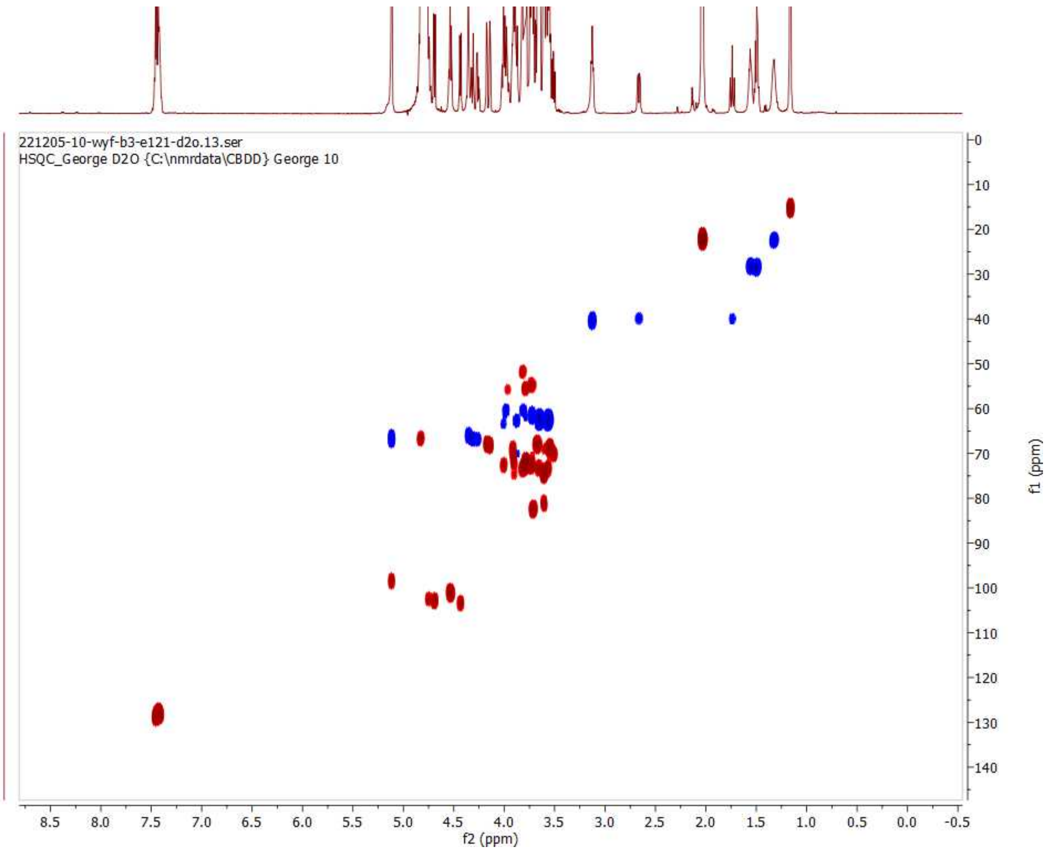

HSQC of 32; 600 MHz/150 MHz, D_2O

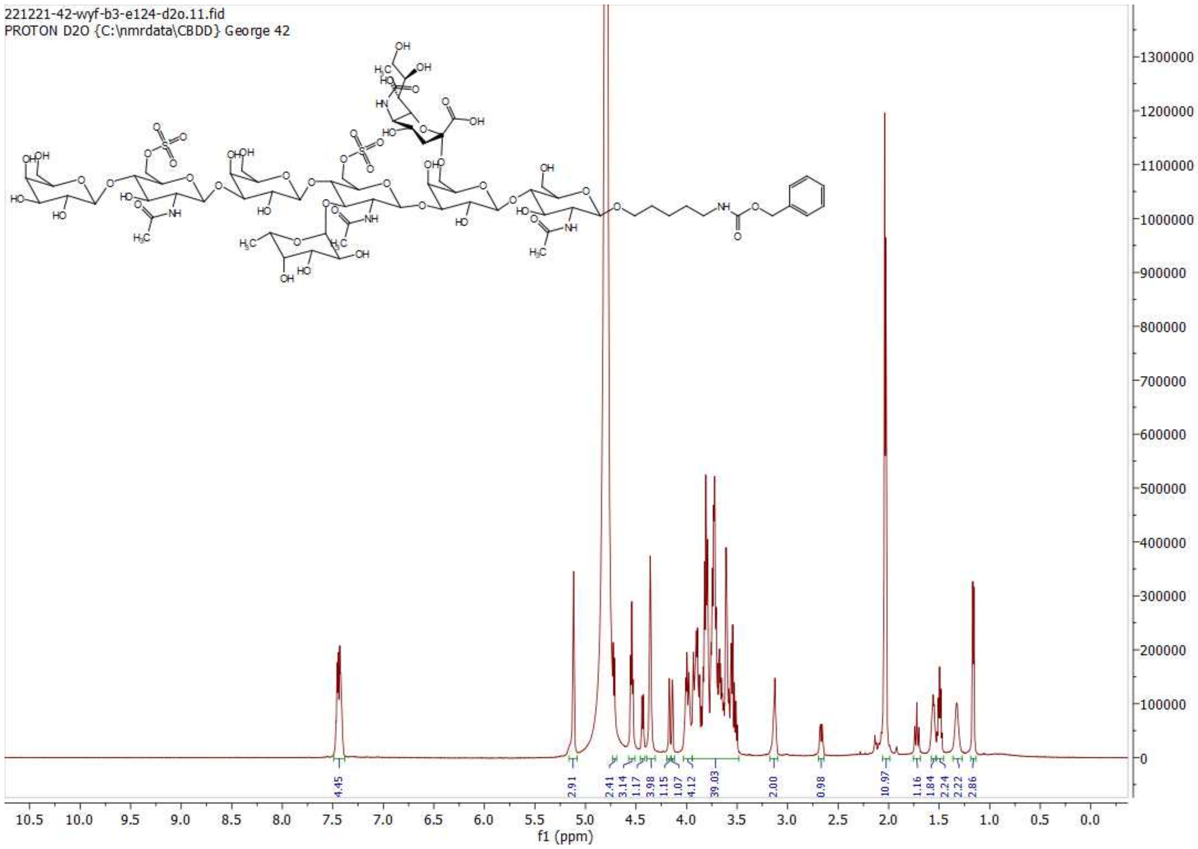

^{1}H NMR of 33; 600MHz; D_2O

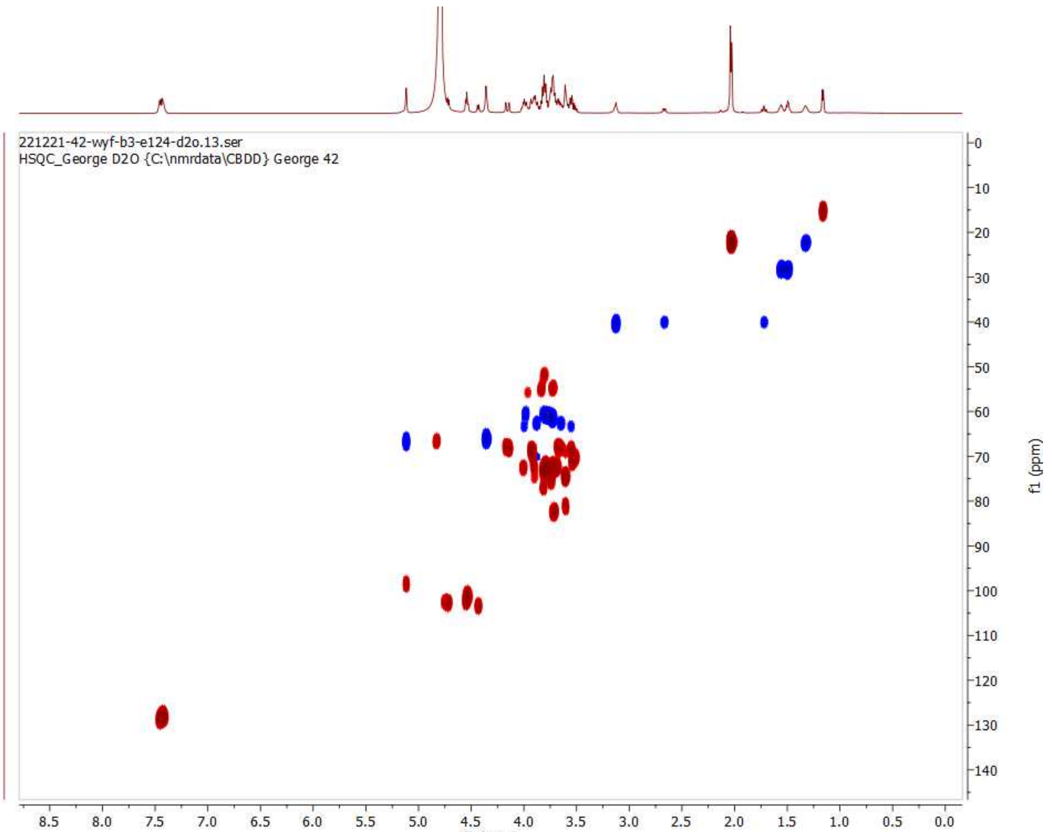

HSQC of 33; 600 MHz/150 MHz, D_2O

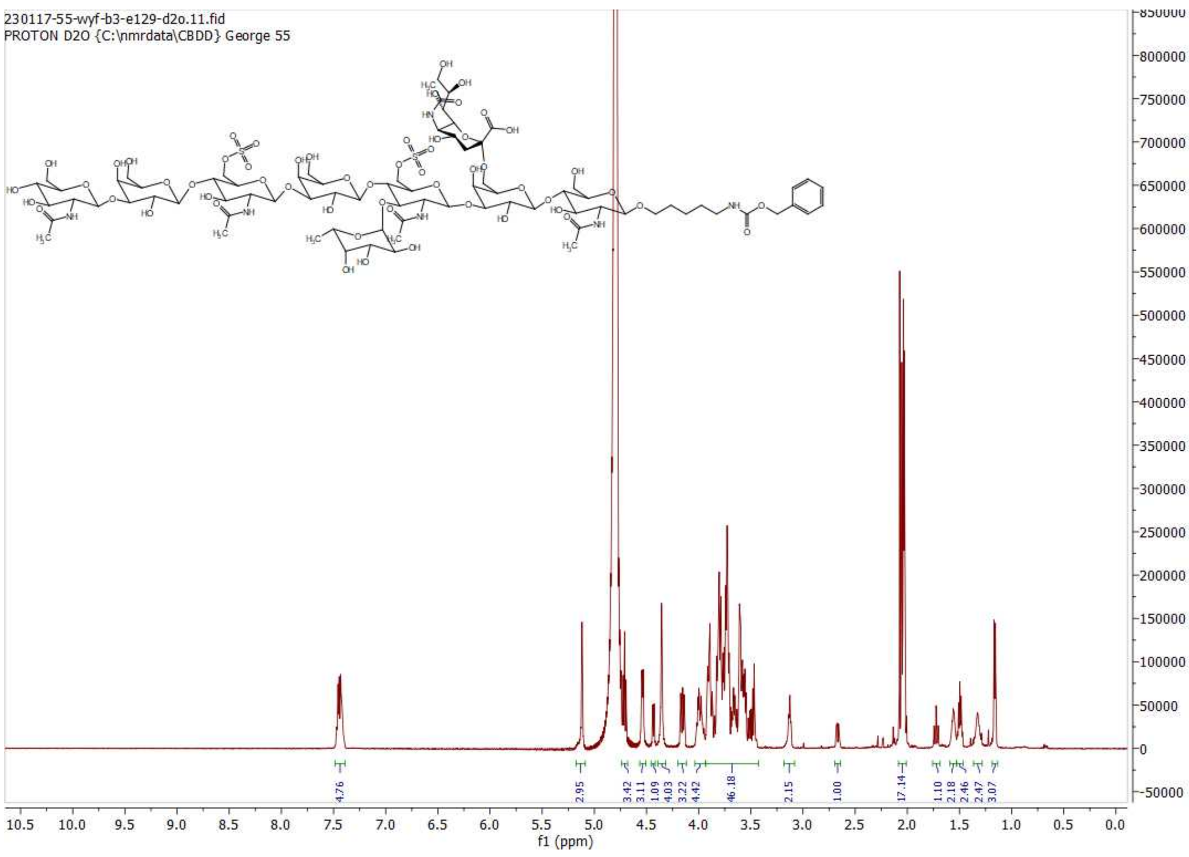

^{1}H NMR of 34; 600MHz; D_2O

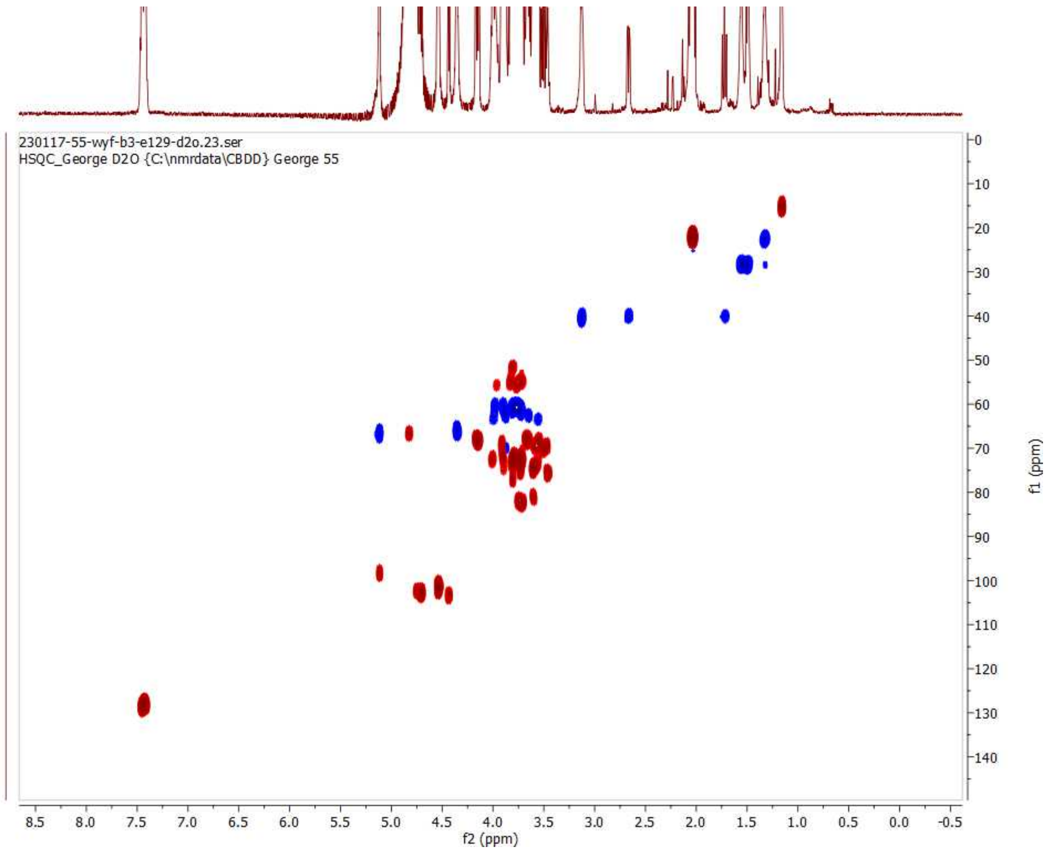

HSQC of 34; 600 MHz/150 MHz, D_2O

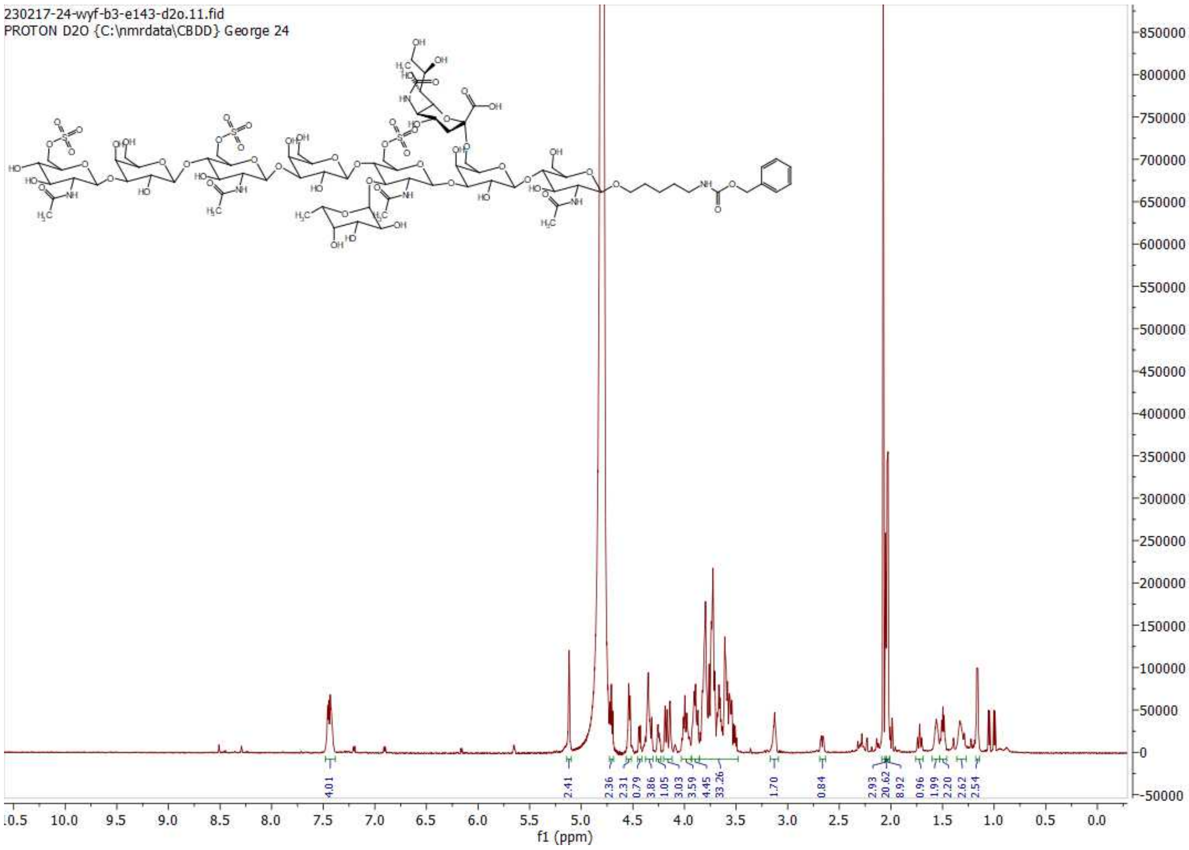

^{1}H NMR of 35; 600MHz; D_2O

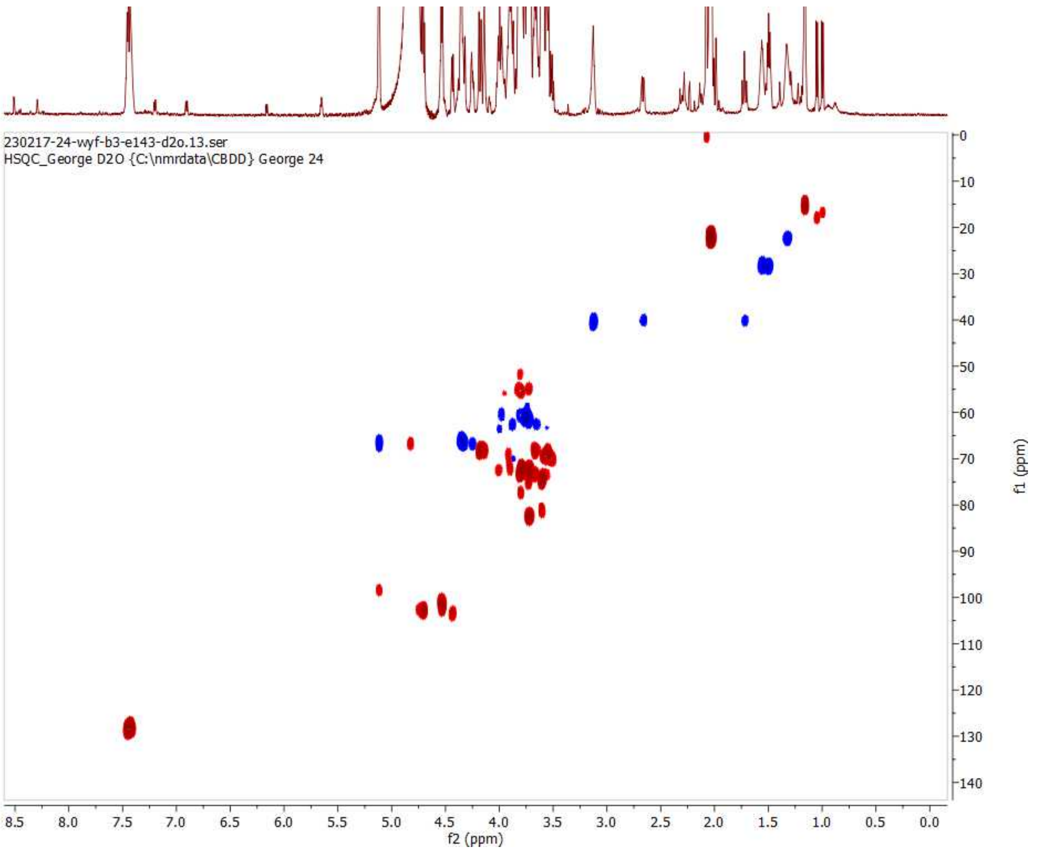

HSQC of 35; 600 MHz/150 MHz, D_2O

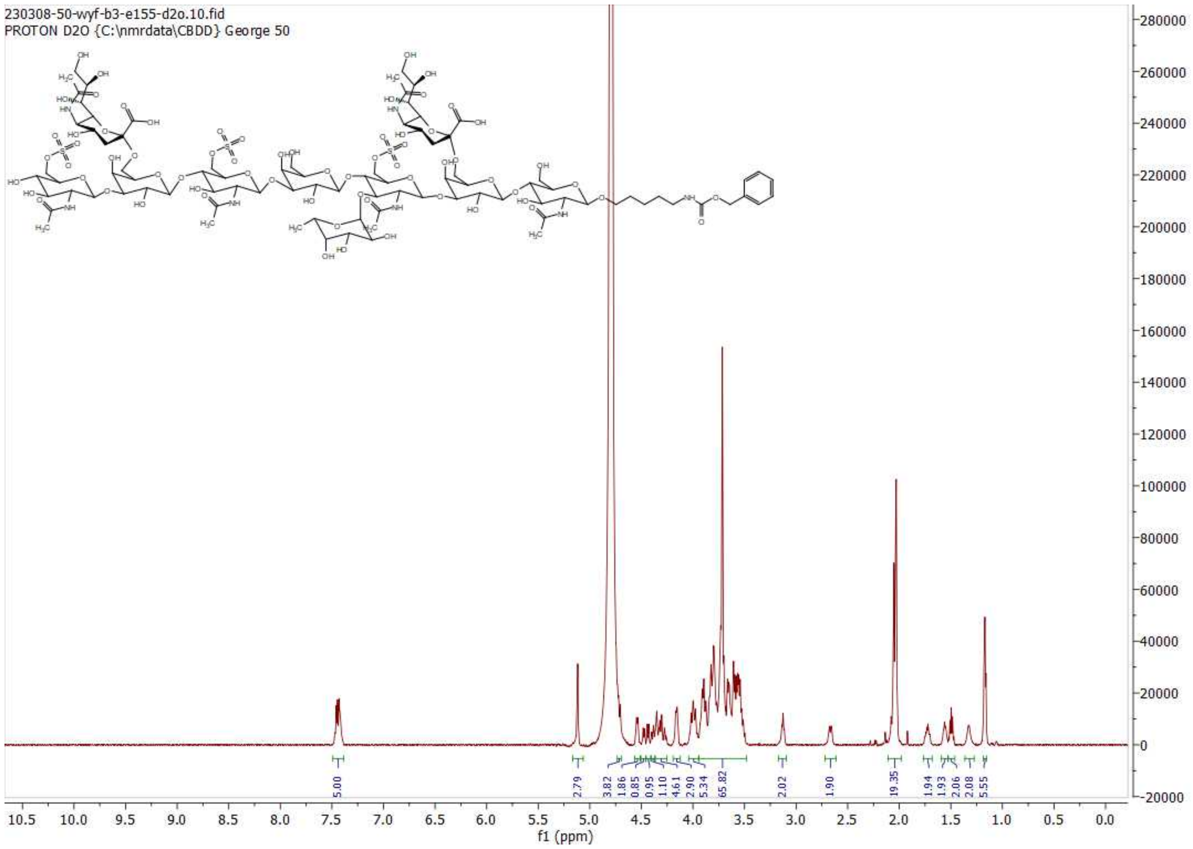

^{1}H NMR of 36; 600MHz; D$_2$O

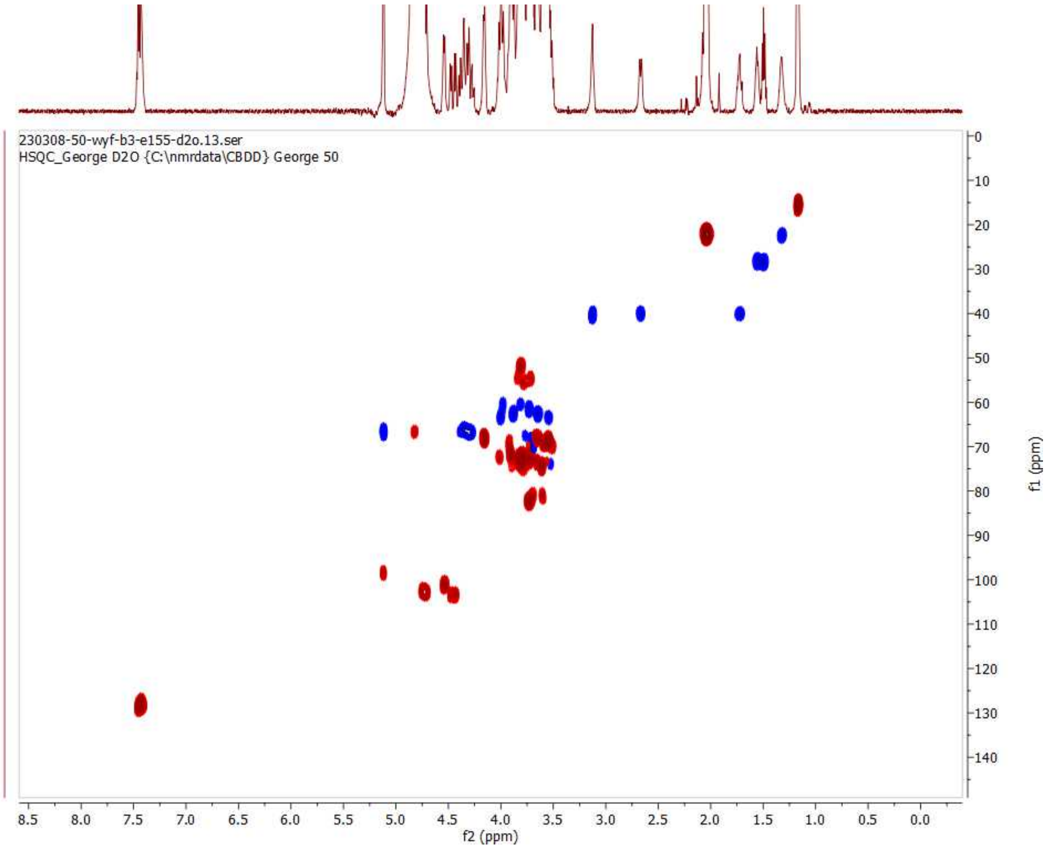

HSQC of 36; 600 MHz/150 MHz, D$_2$O

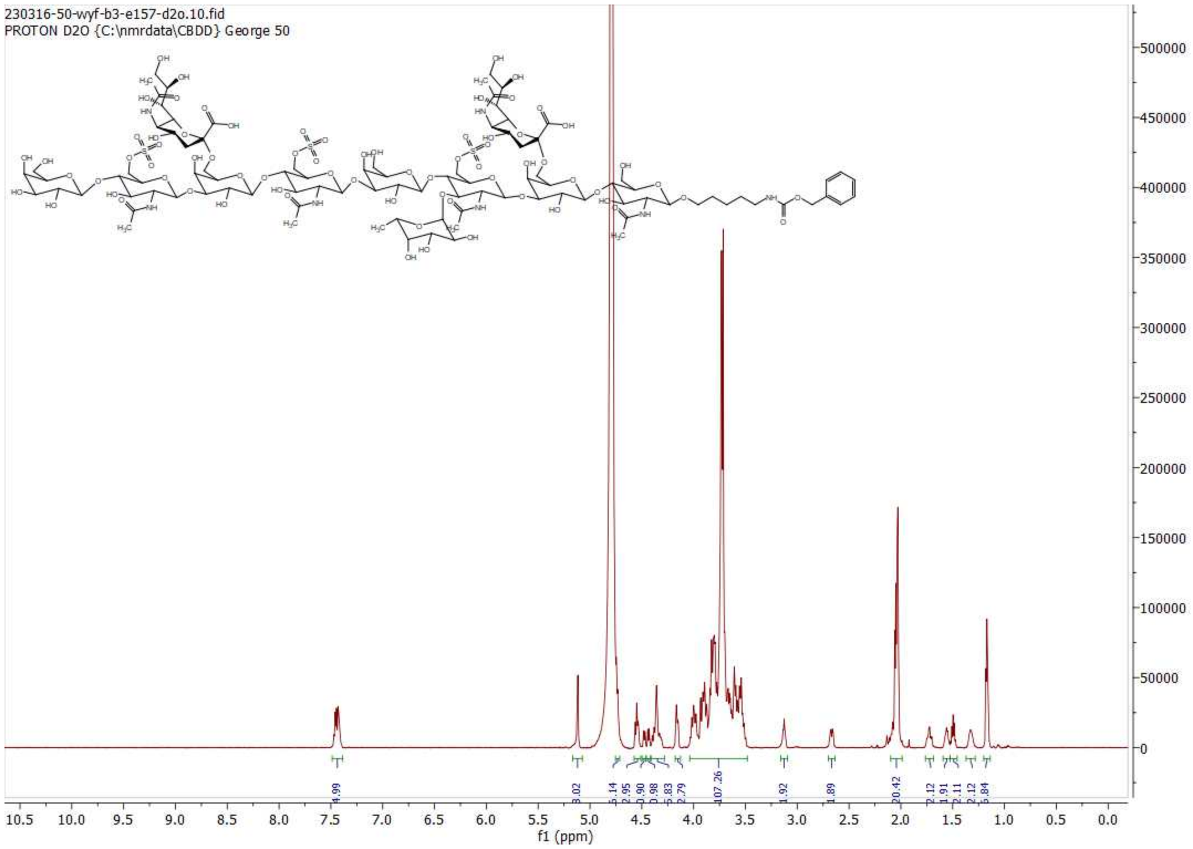

^{1}H NMR of 37; 600MHz; D$_2$O

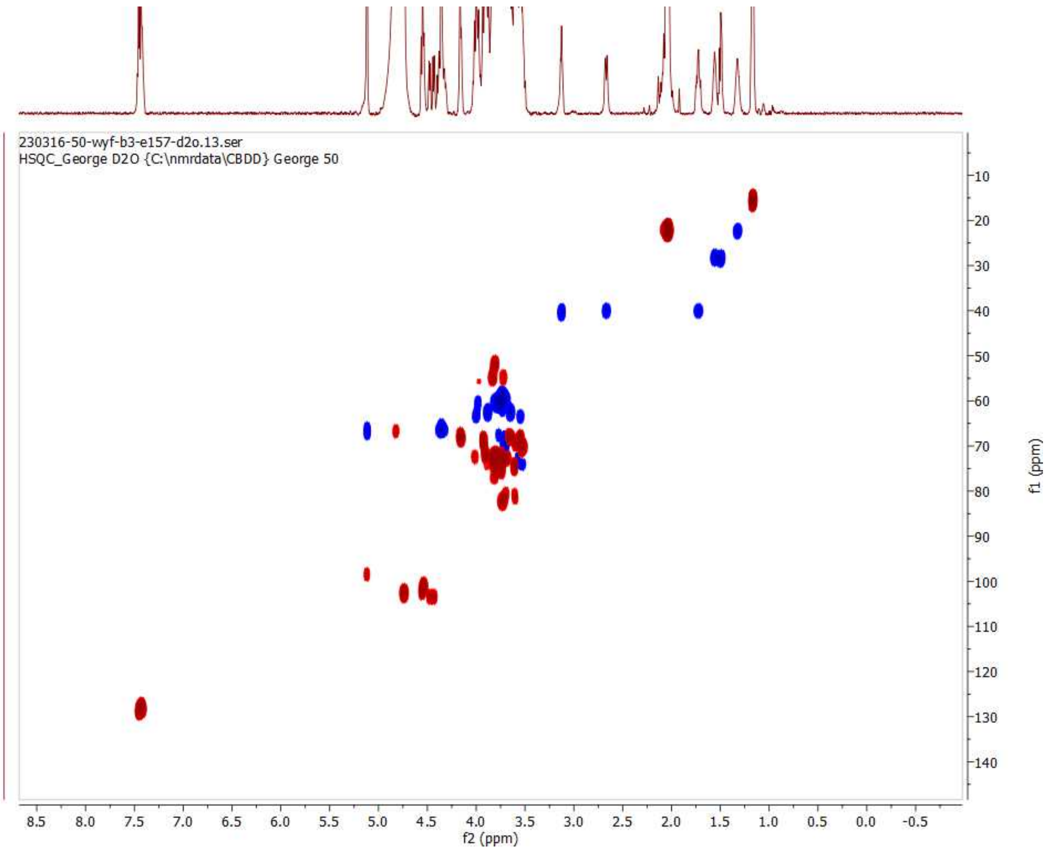

HSQC of 37; 600 MHz/150 MHz, D$_2$O

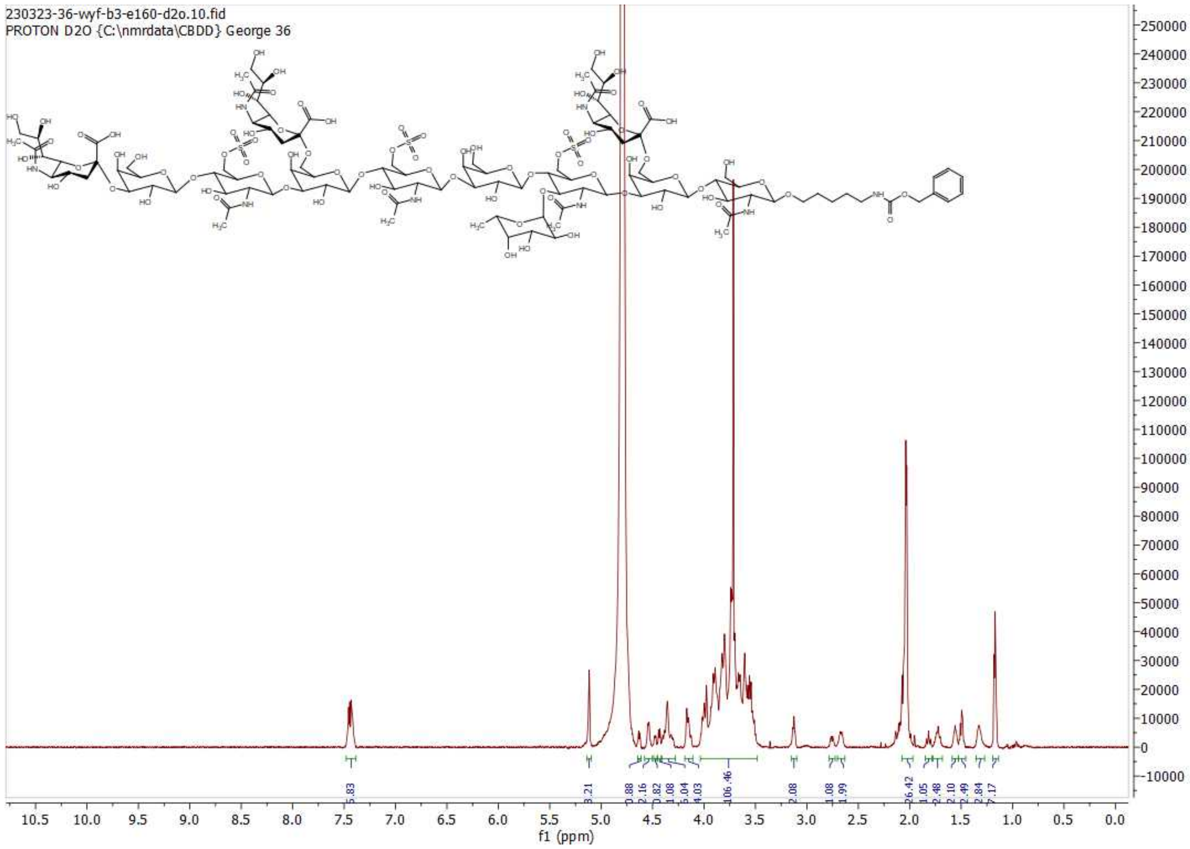

^{1}H NMR of 38; 600MHz; D_2O

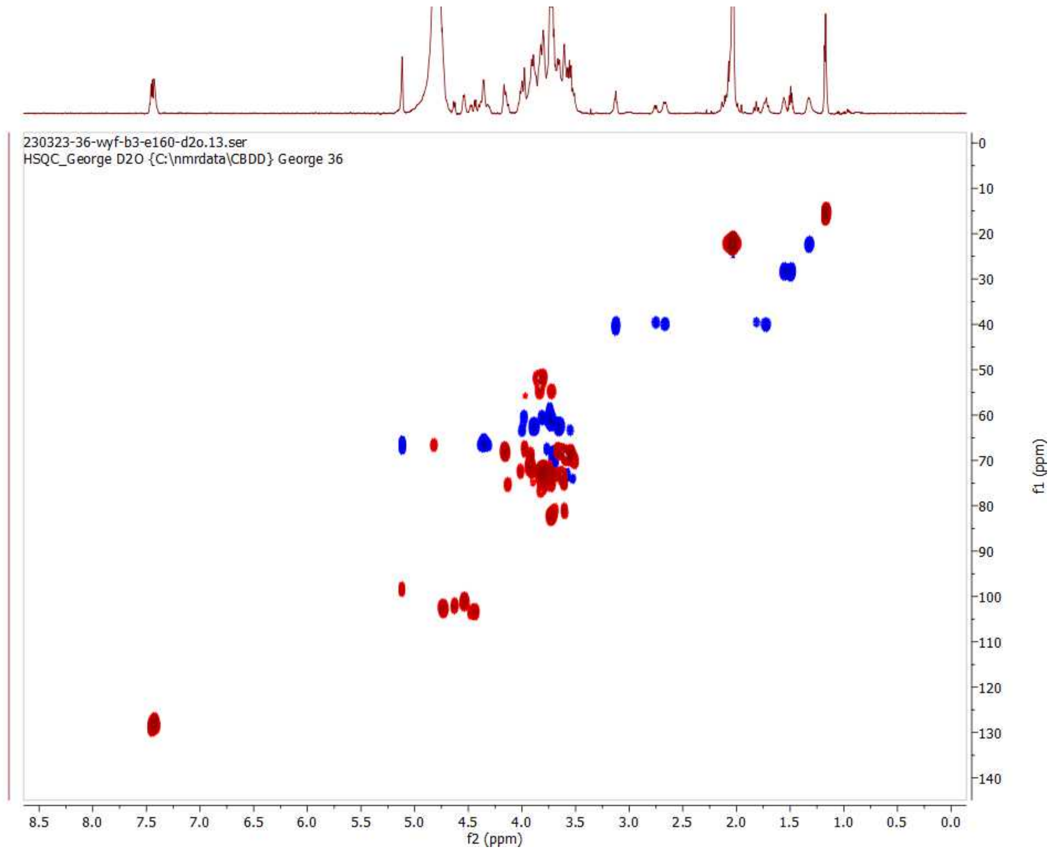

HSQC of 38; 600 MHz/150 MHz, D_2O

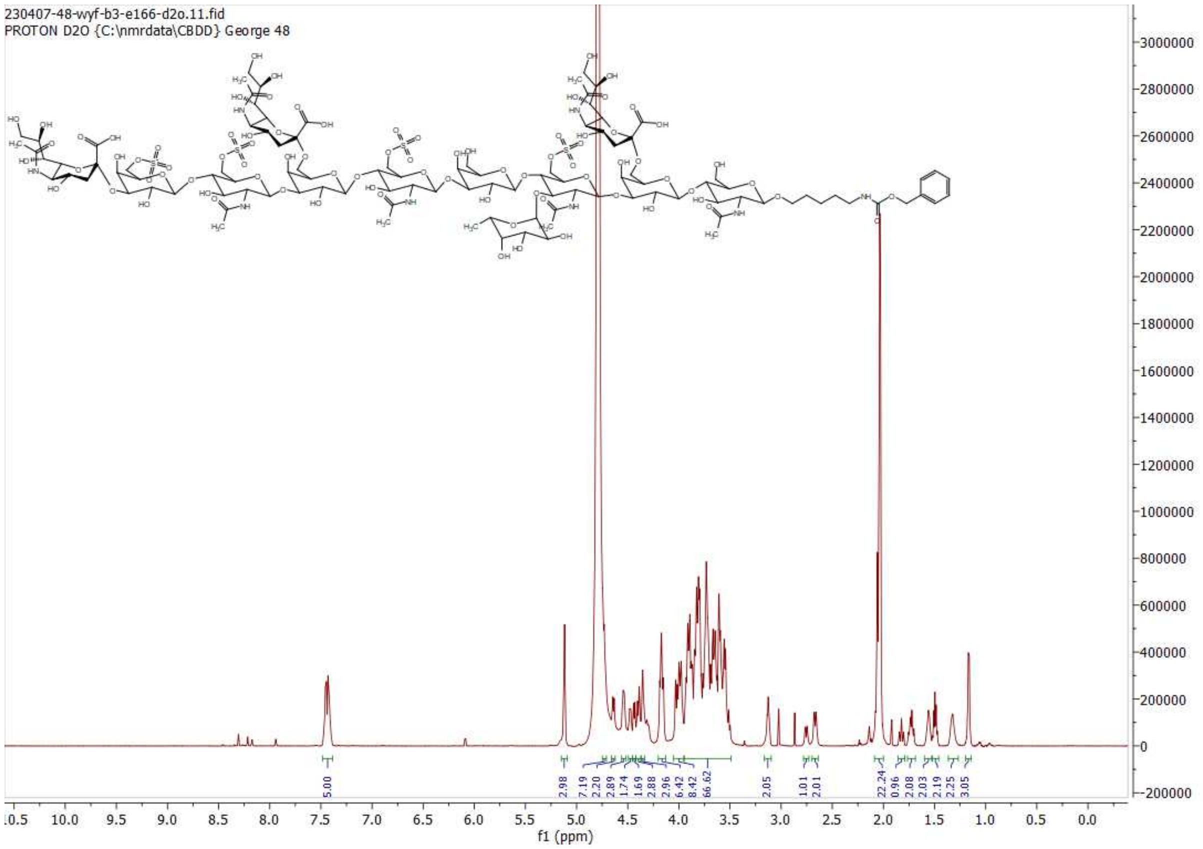

^{1}H NMR of 39; 600MHz; D_2O

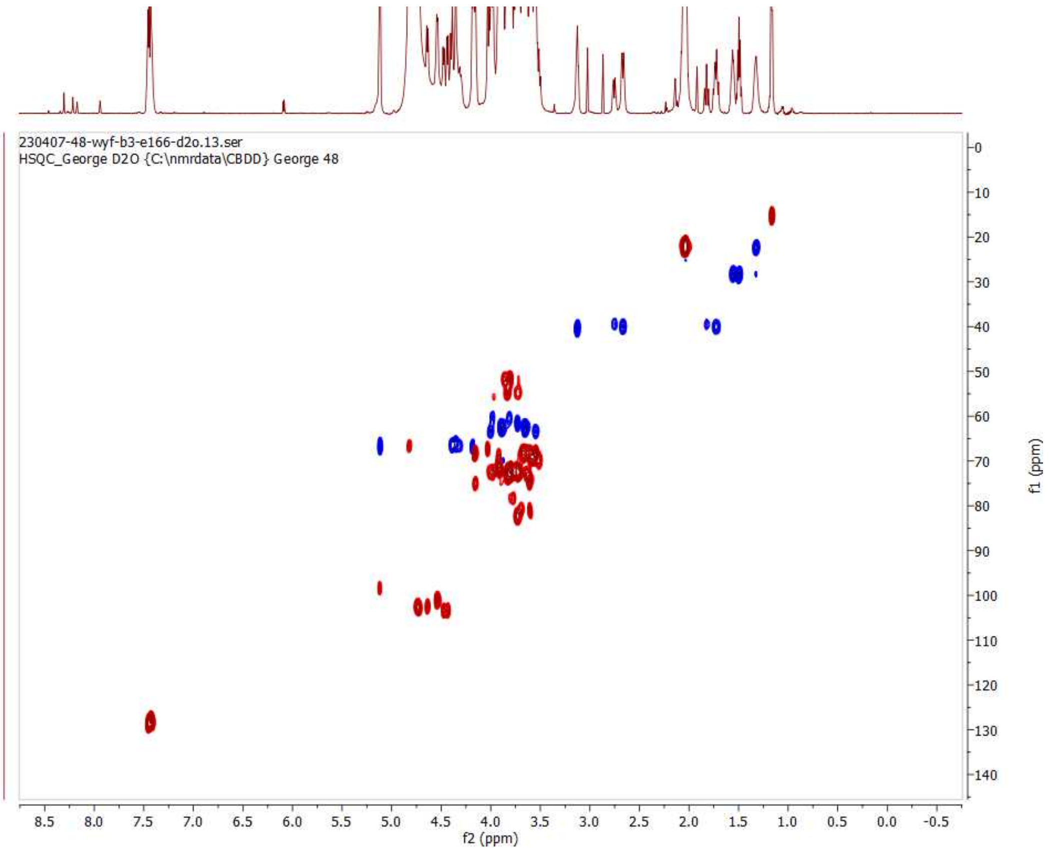

HSQC of 39; 600 MHz/150 MHz, D_2O

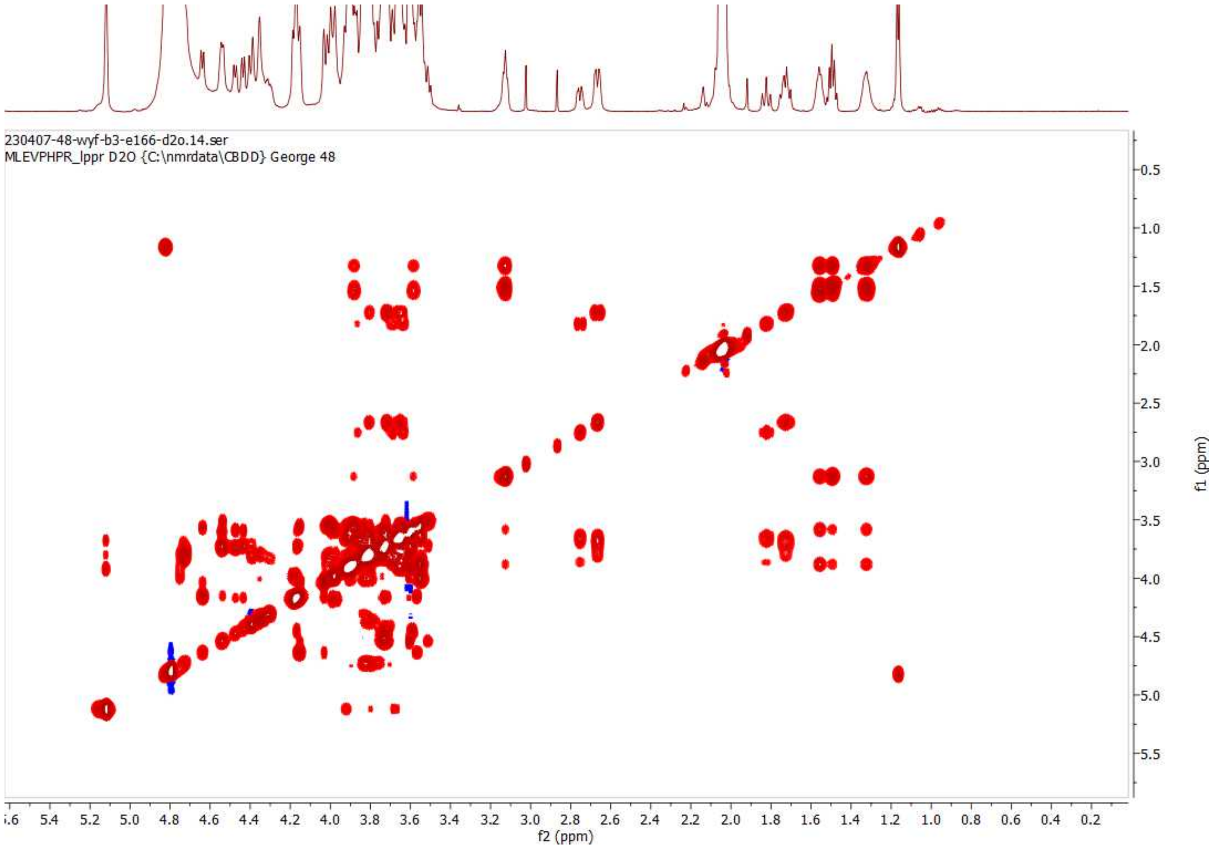

TOCSY (80 ms) of 39; 600MHz; D_2O

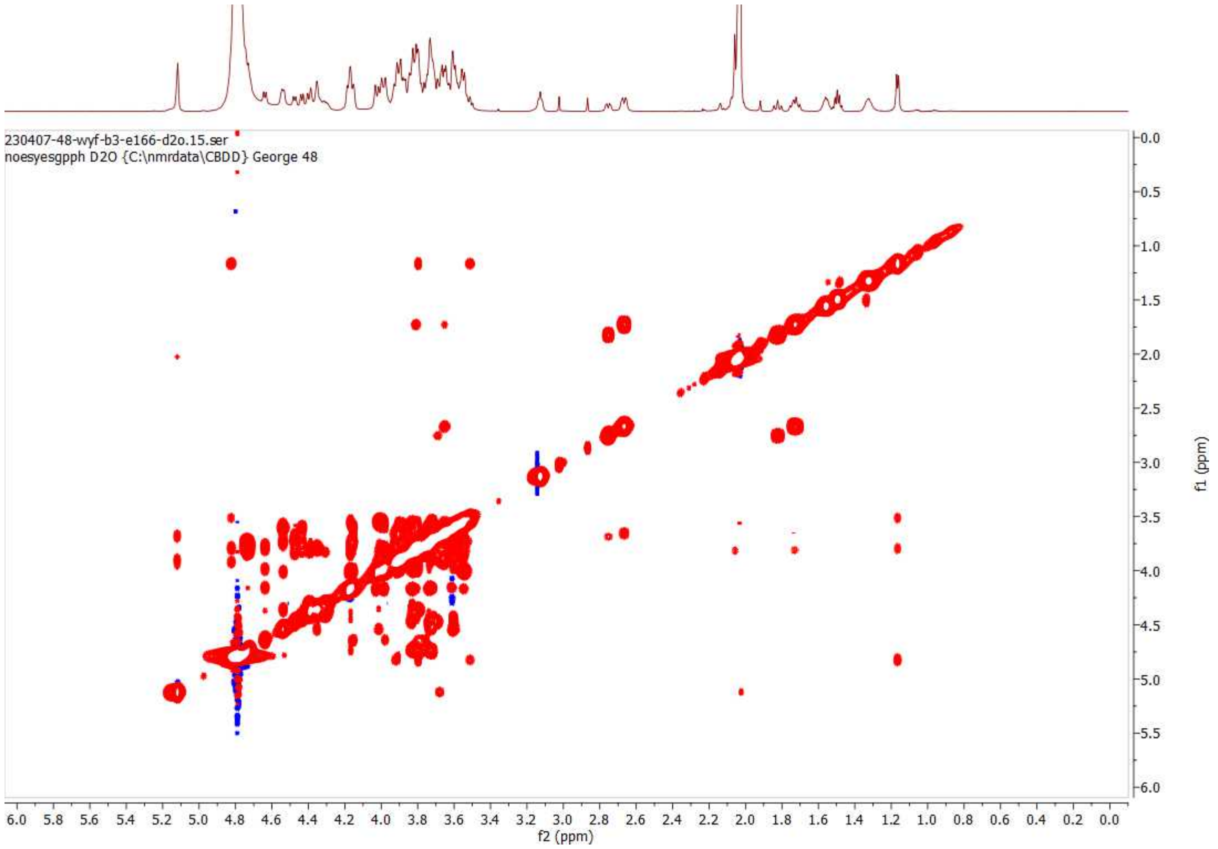

NOESY (300 ms) of 39; 600 MHz, D_2O

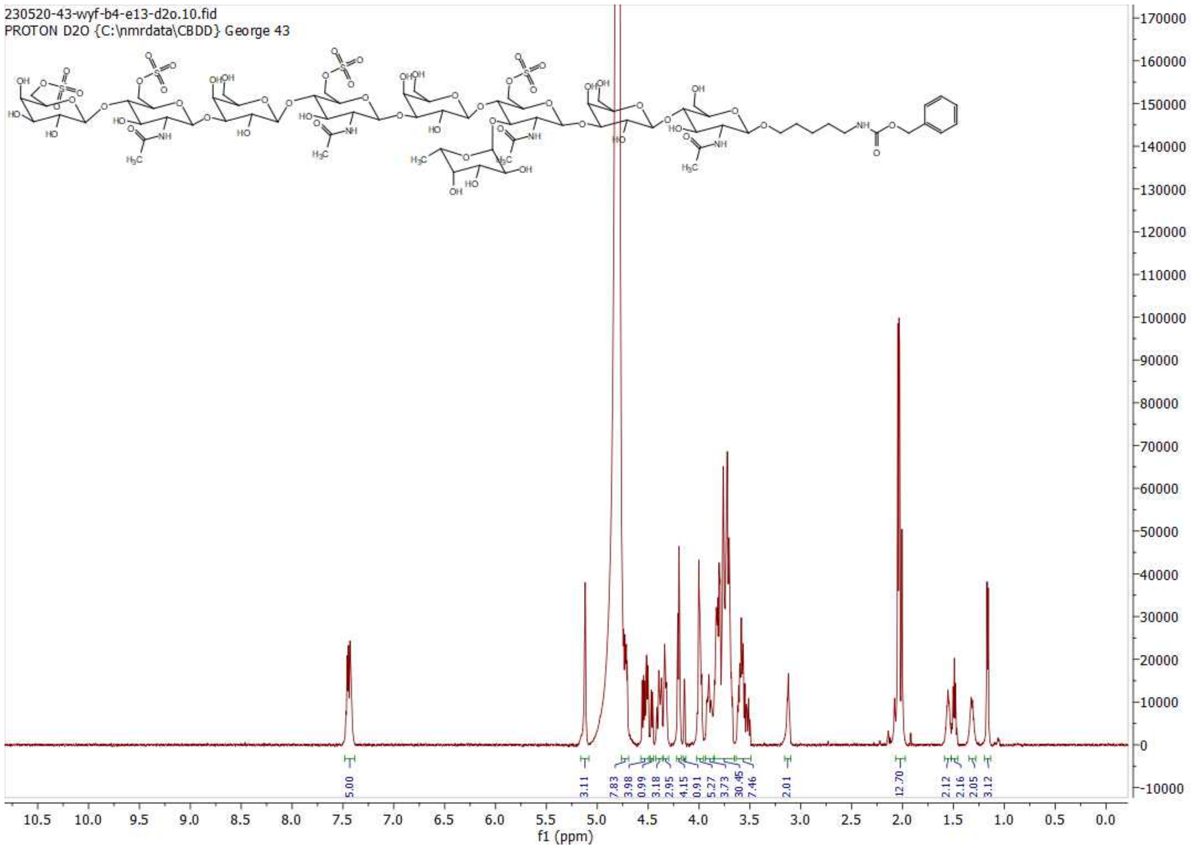

^{1}H NMR of S17; 600MHz; D_2O

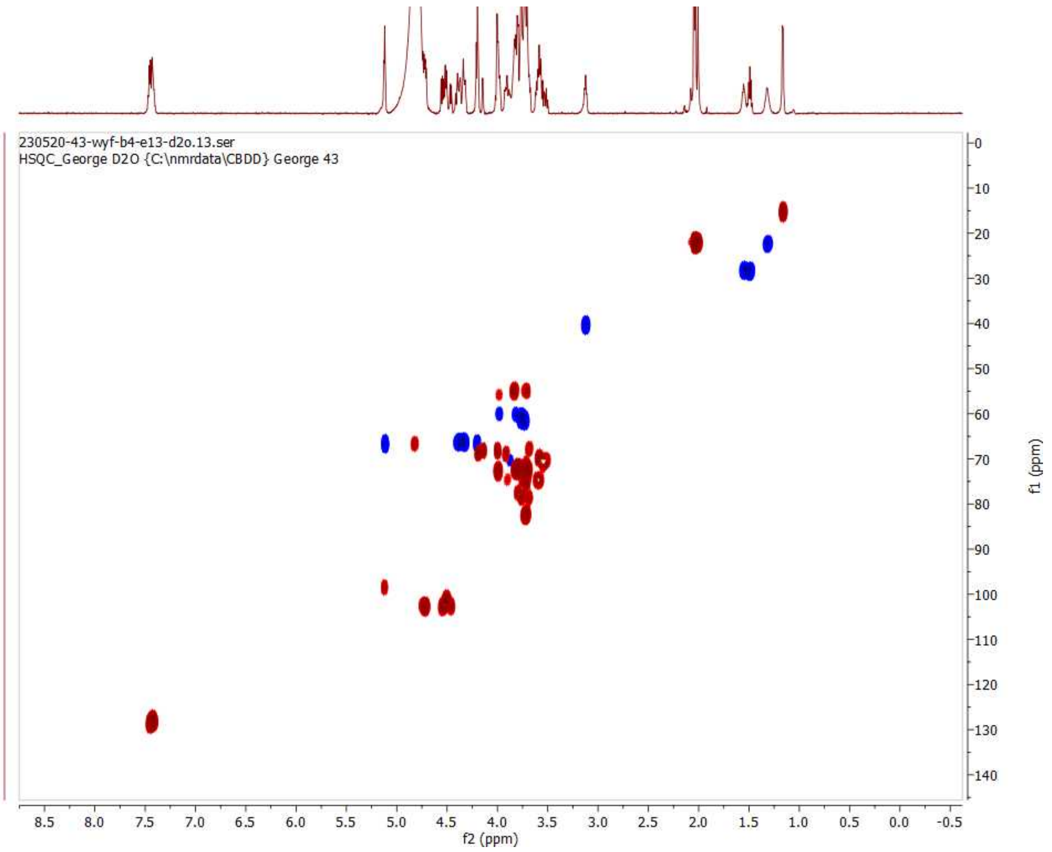

HSQC of S17; 600 MHz/150 MHz, D_2O

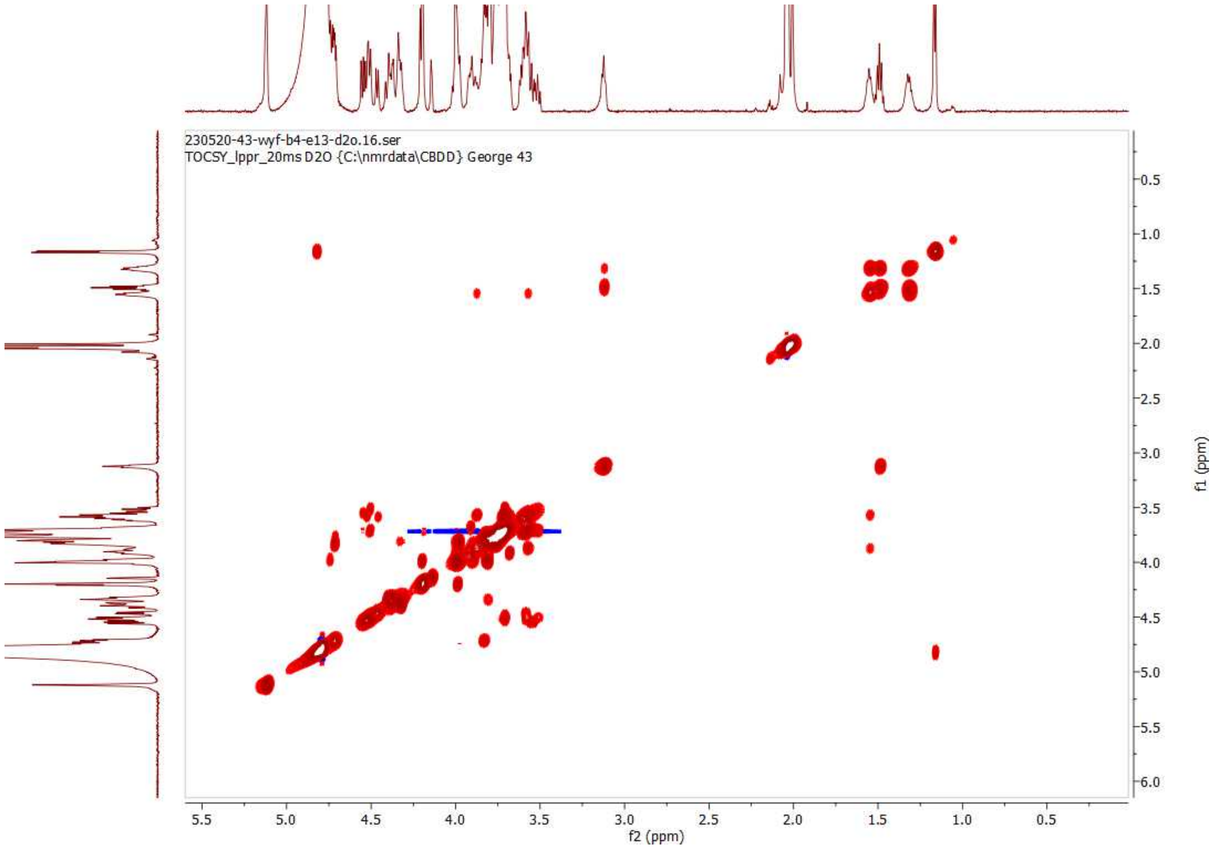

TOCSY (20 ms) of S17; 600MHz; D_2O

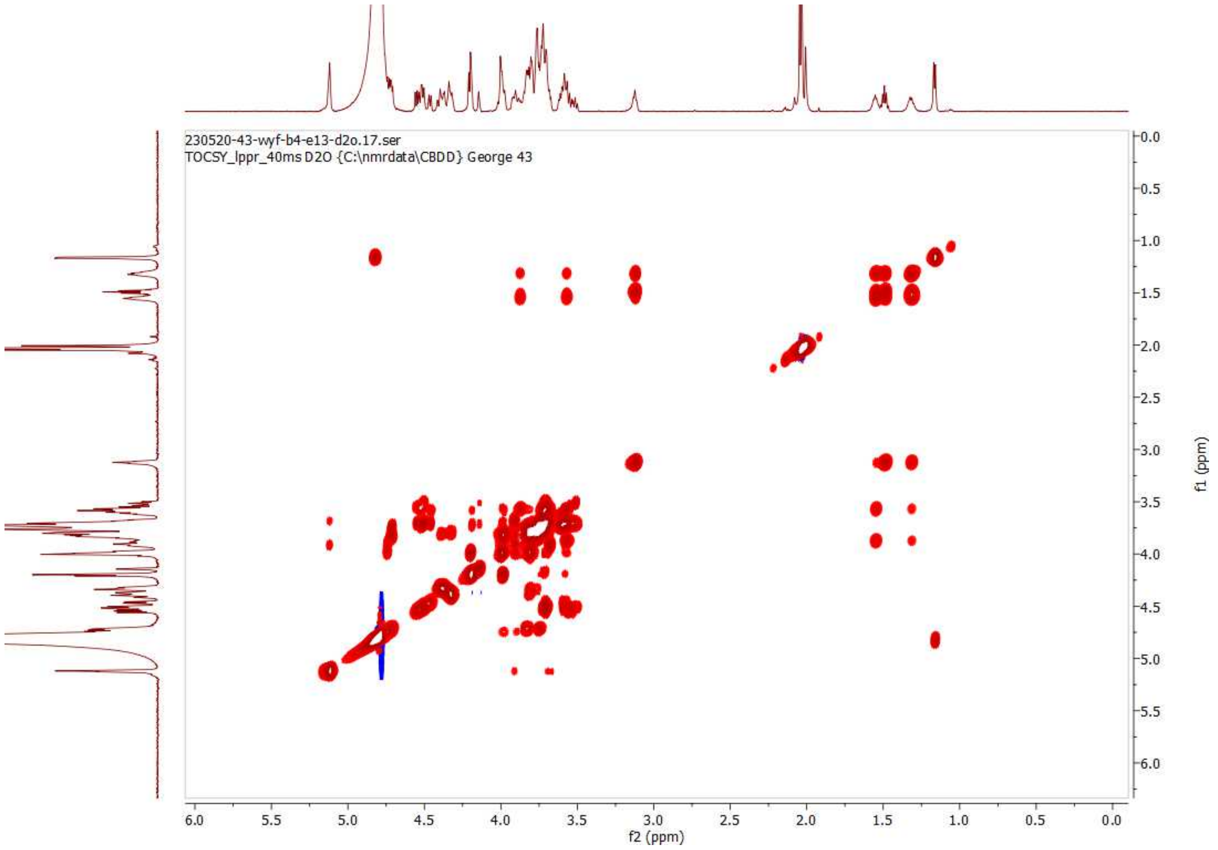

TOCSY (40 ms) of S17; 600MHz; D_2O

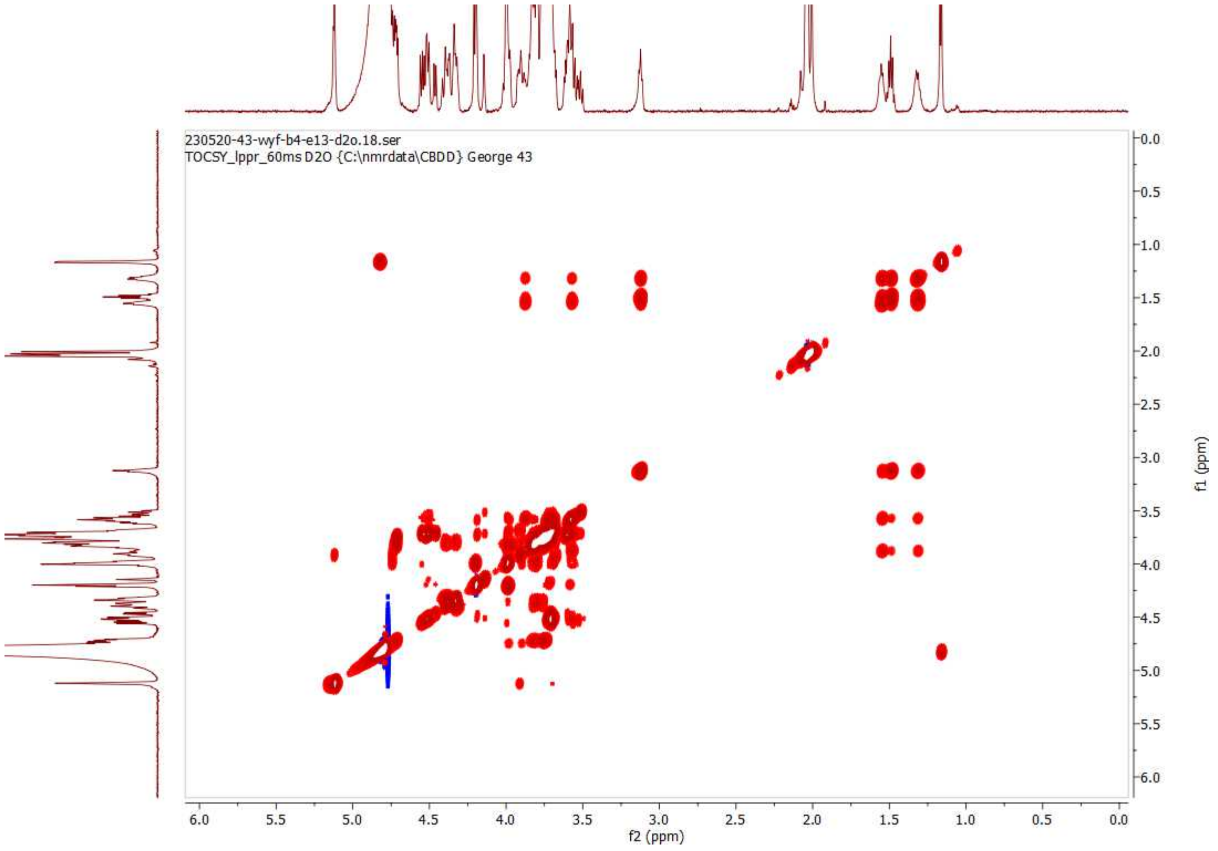

TOCSY (60 ms) of S17; 600MHz; D_2O

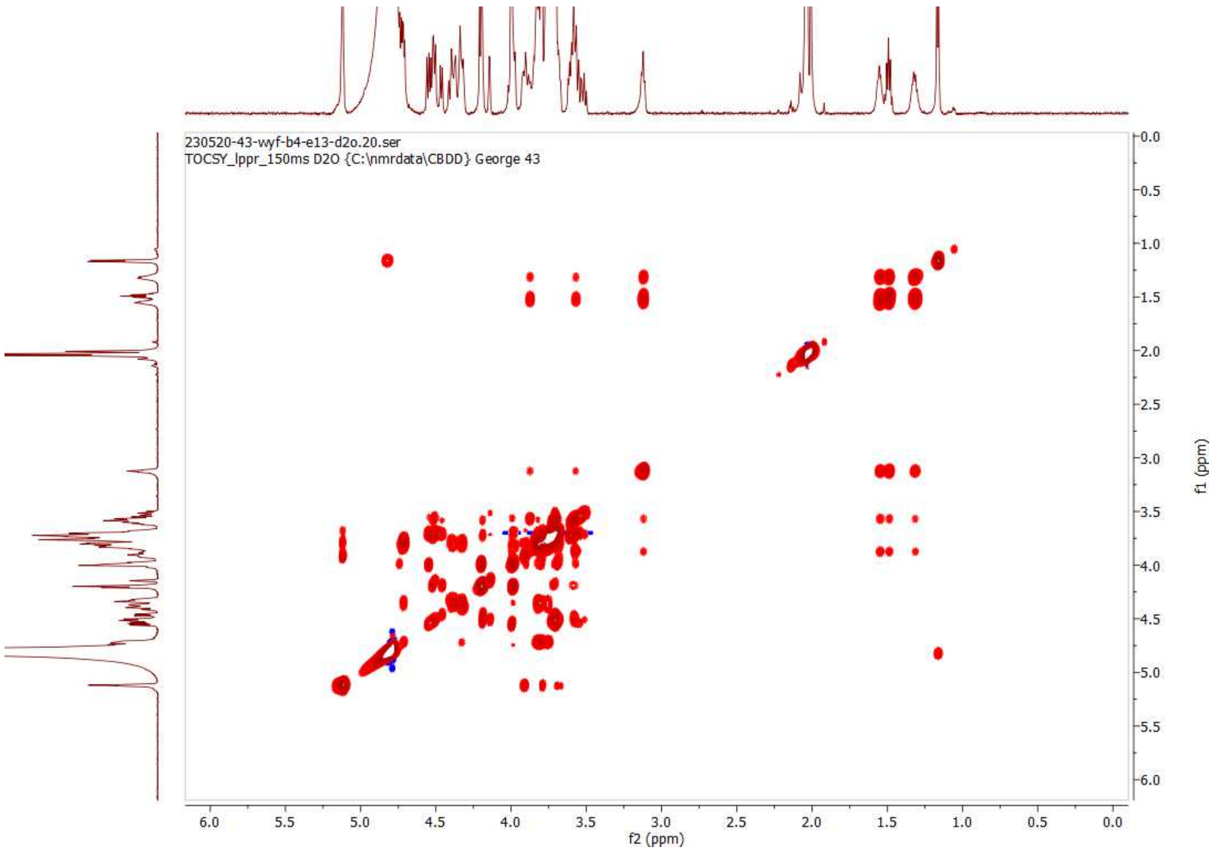

TOCSY (150 ms) of S17; 600MHz; D_2O

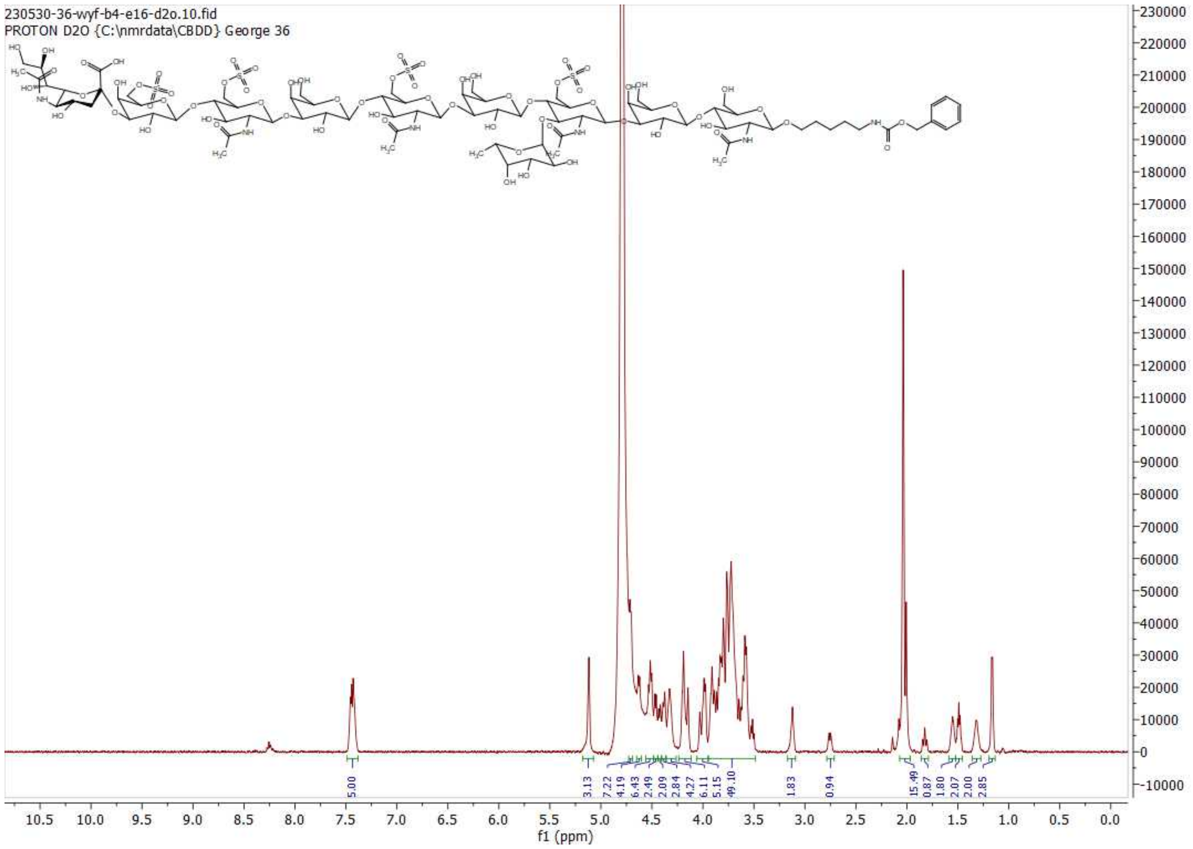

^{1}H NMR of 40; 600MHz; D_2O

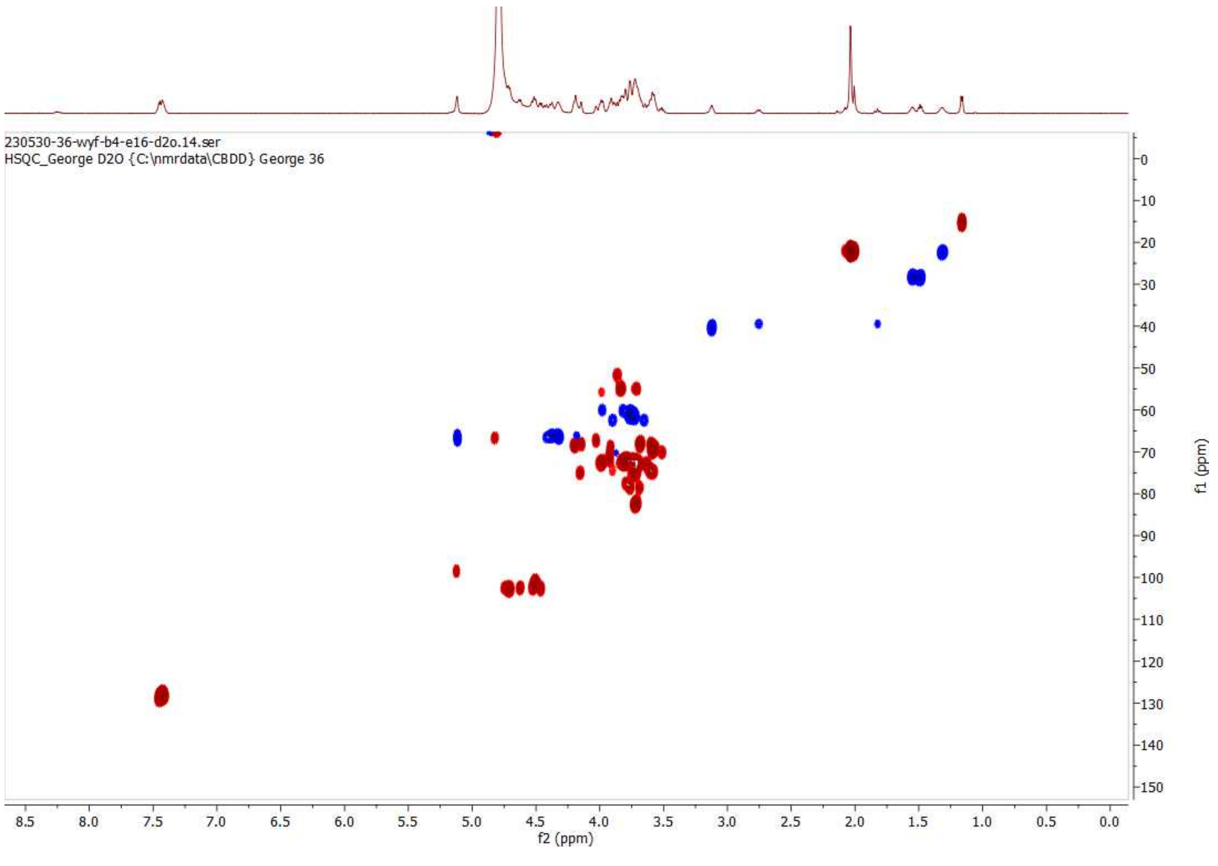

HSQC of 40; 600 MHz/150 MHz, D_2O

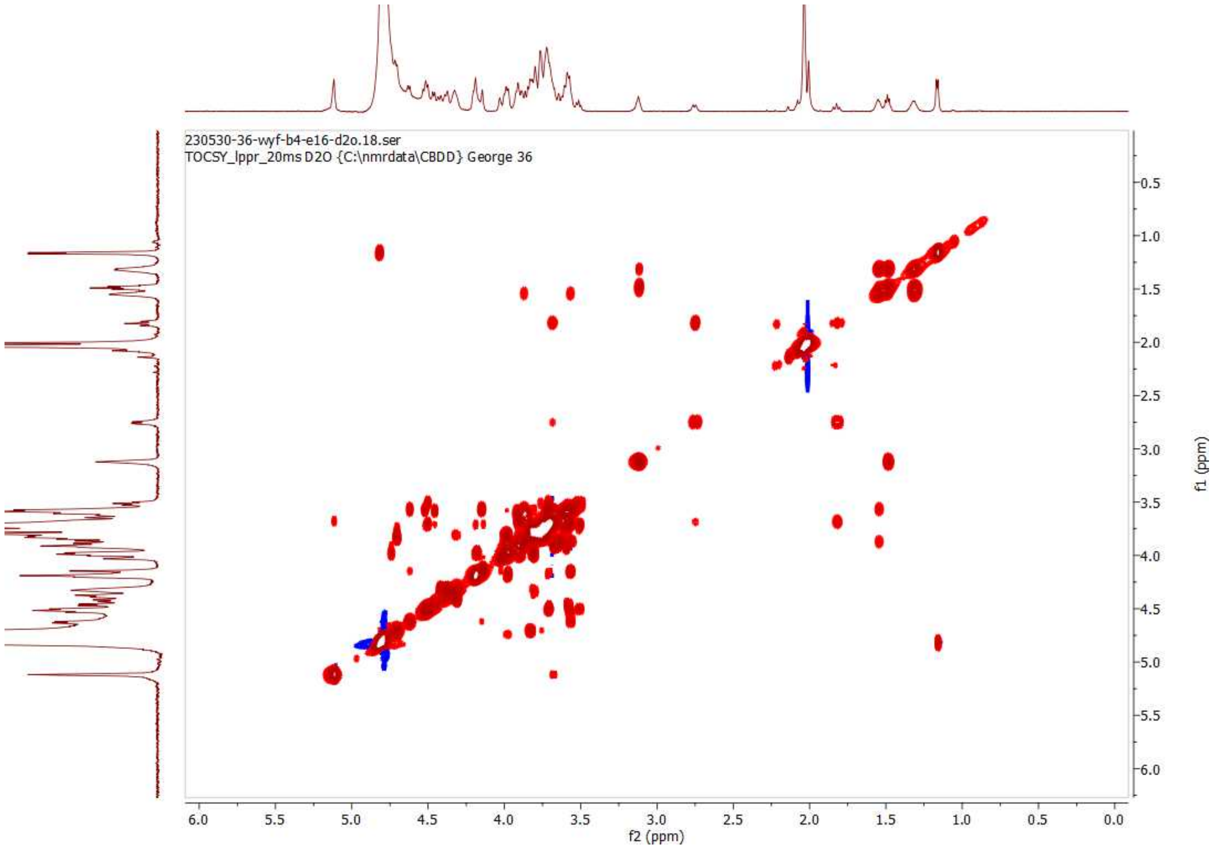

TOCSY (20 ms) of 40; 600MHz; D_2O

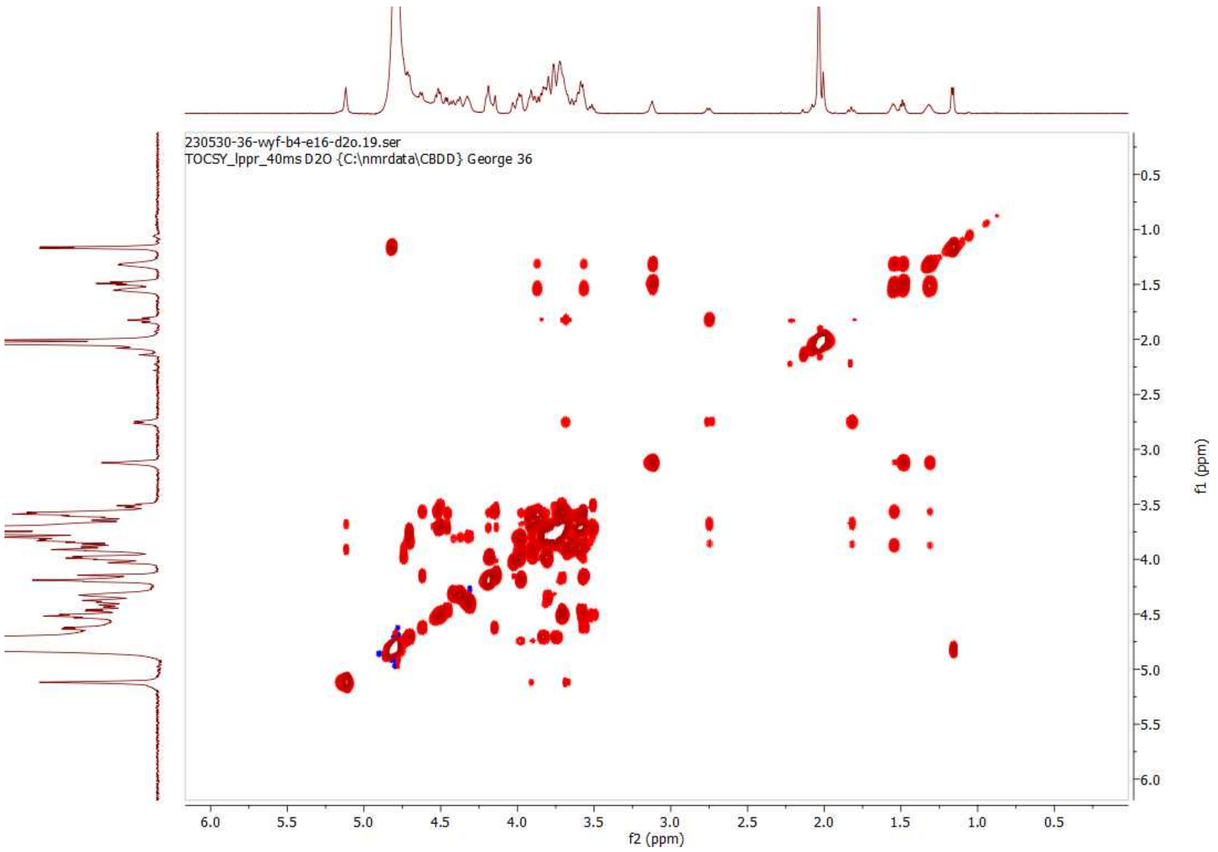

TOCSY (40 ms) of 40; 600MHz; D_2O

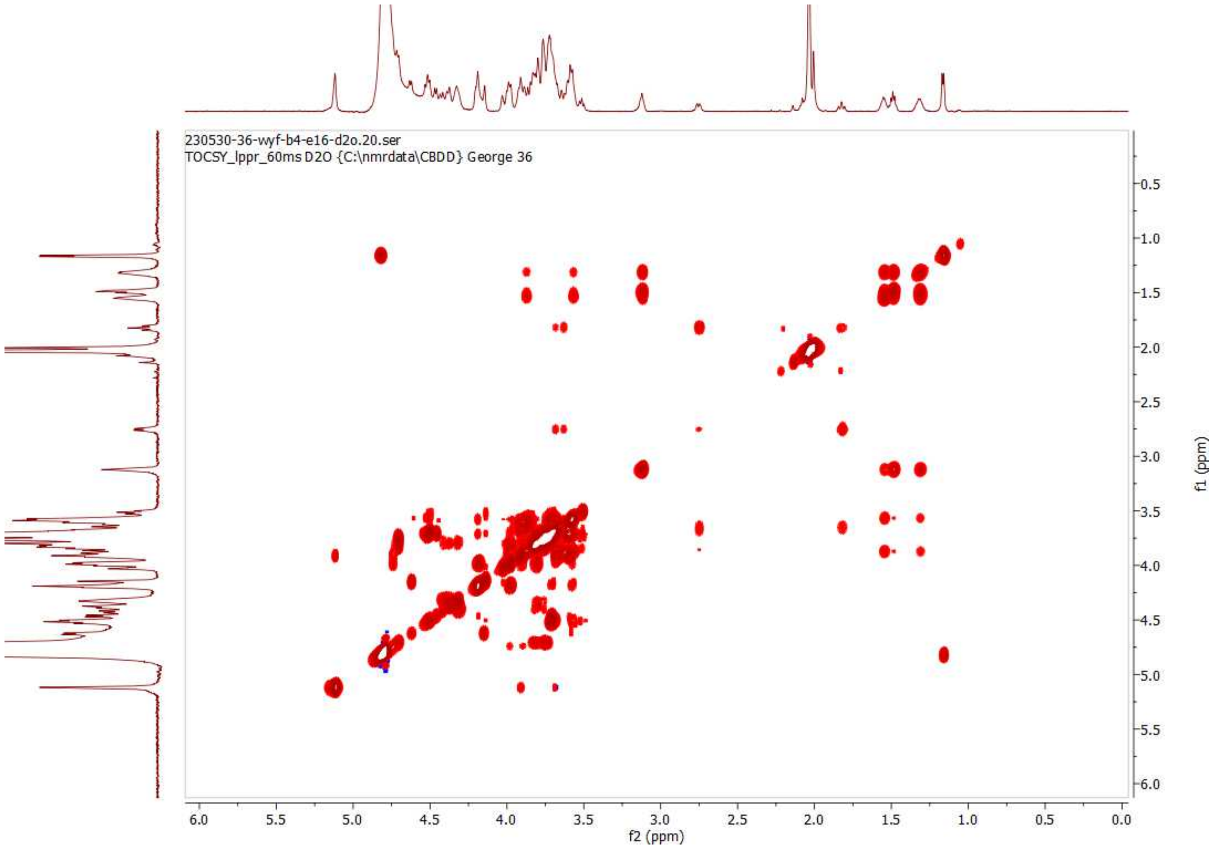

TOCSY (60 ms) of 40; 600MHz; D_2O

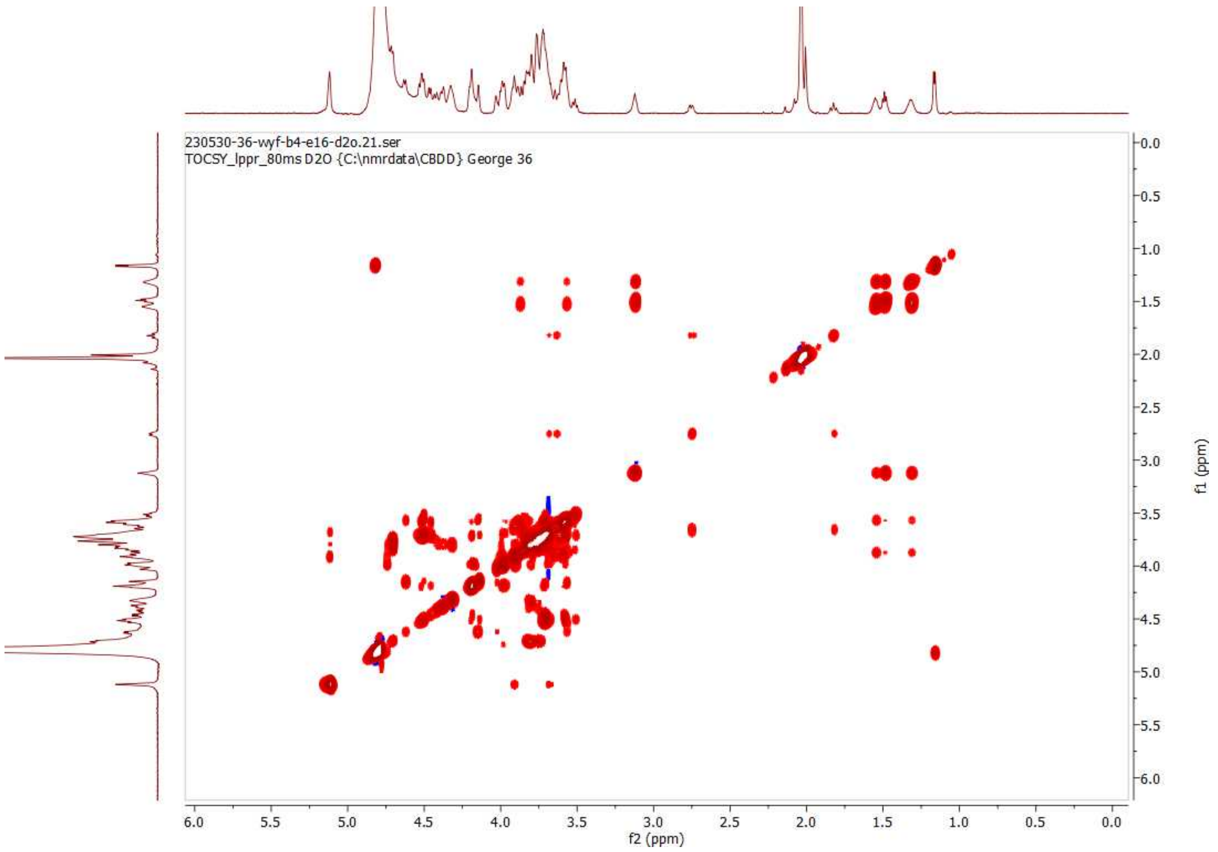

TOCSY (80 ms) of 40; 600MHz; D_2O

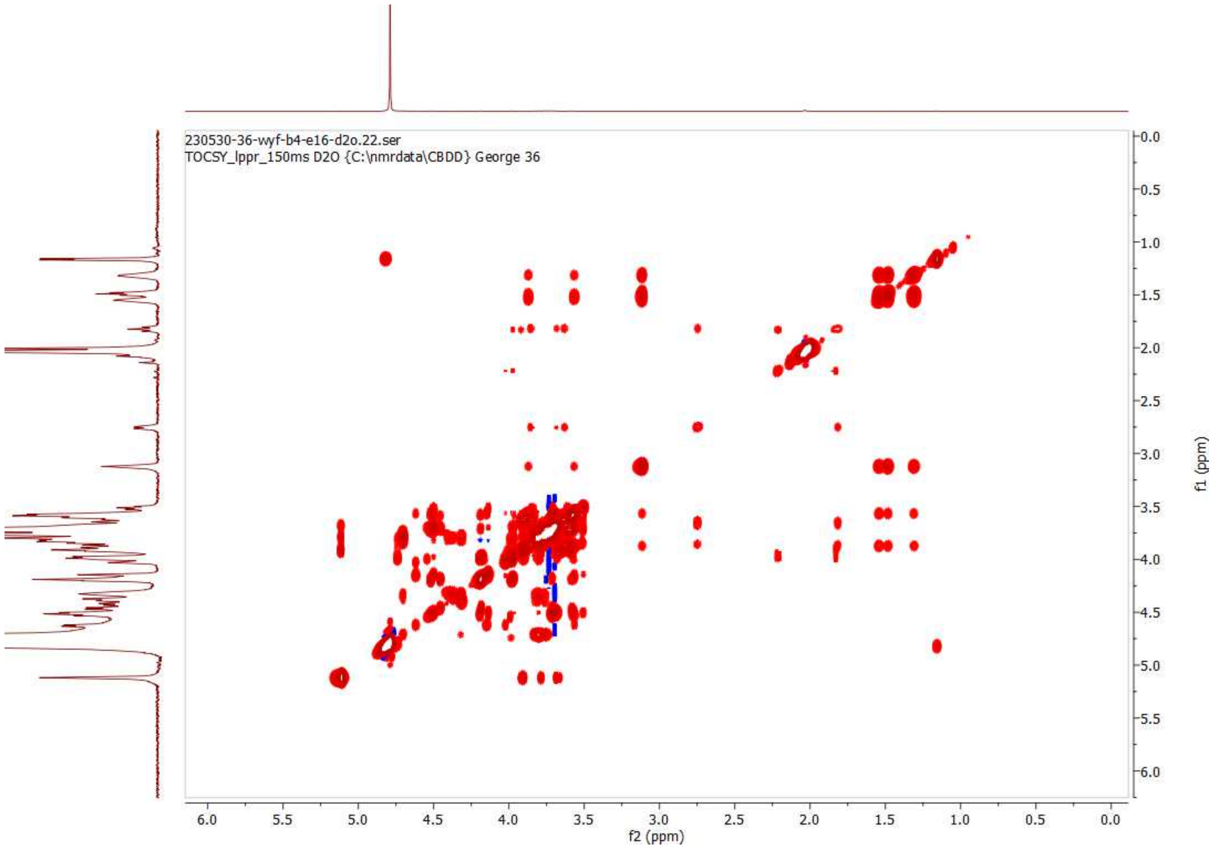

TOCSY (150 ms) of 40; 600MHz; D_2O

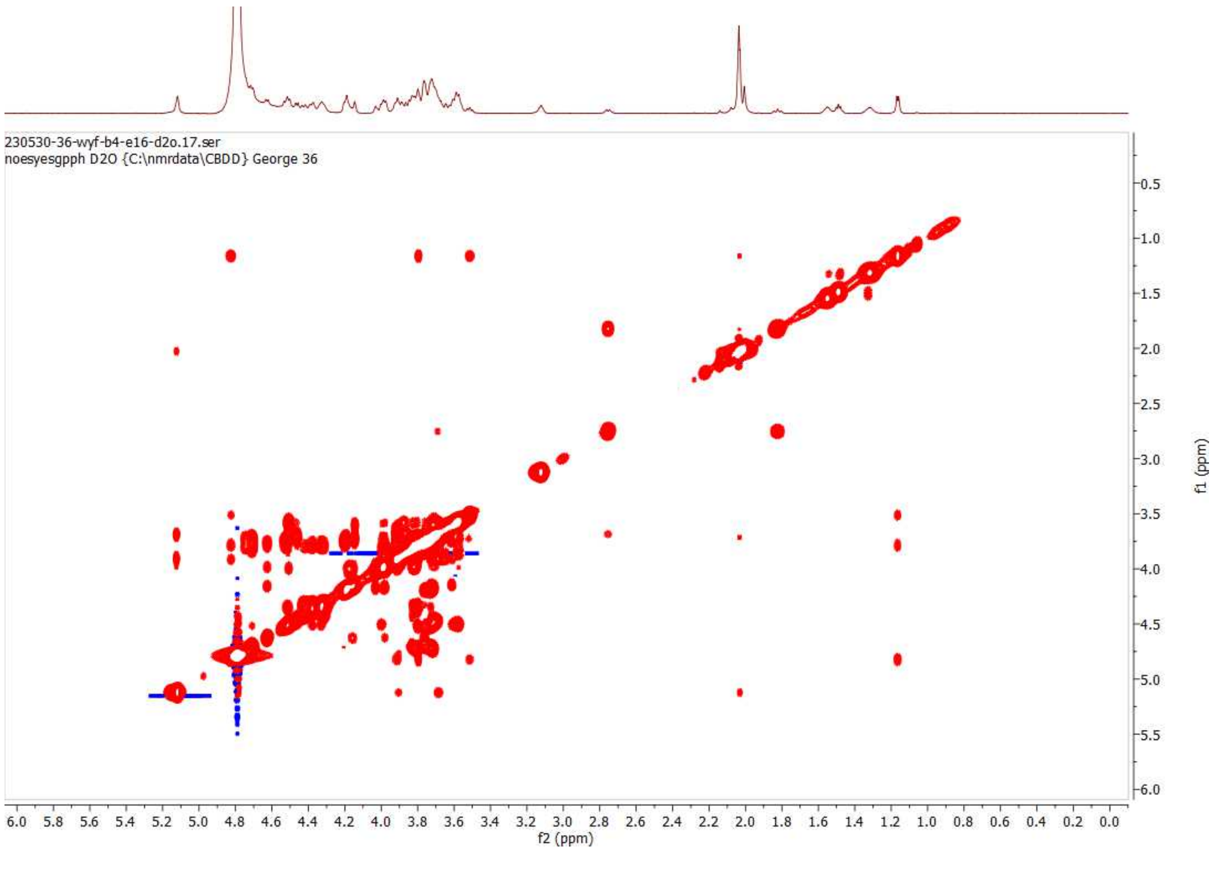

NOESY (300 ms) of 40; 600 MHz, D_2O

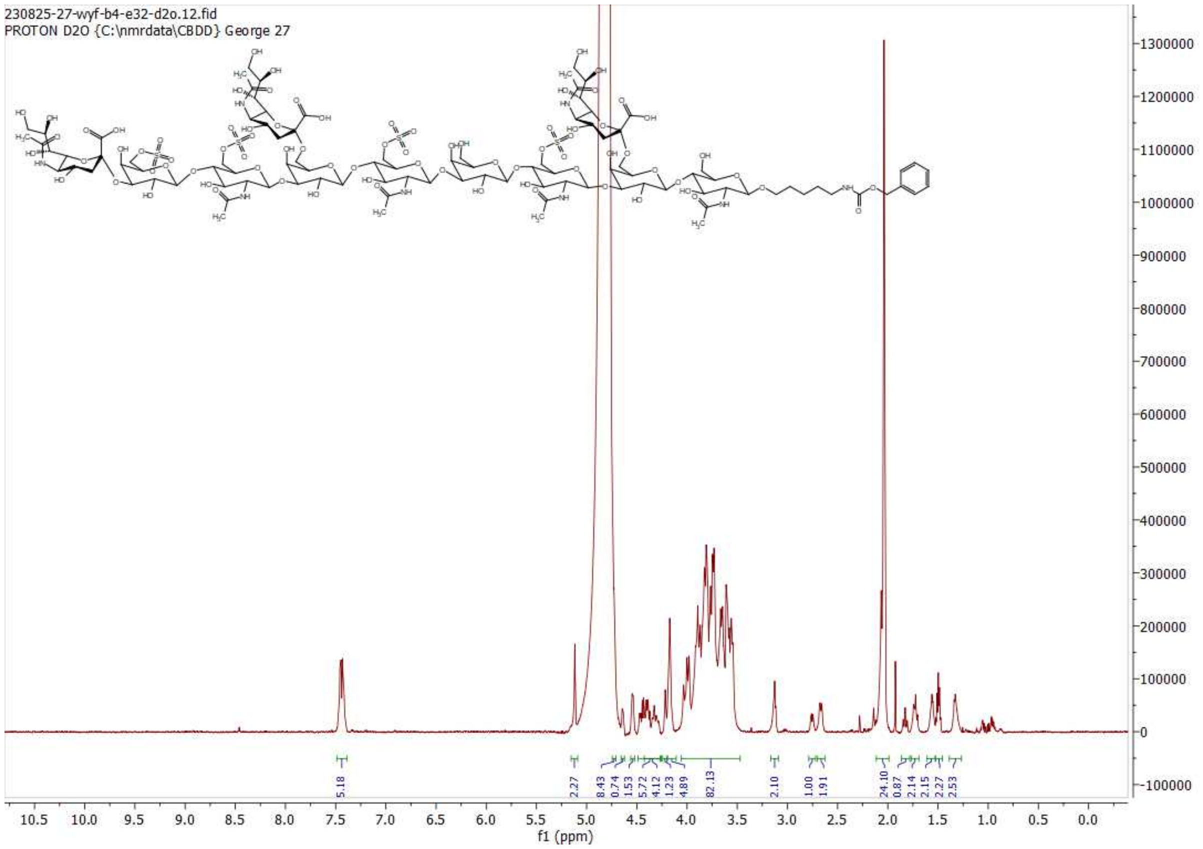

^{1}H NMR of 41; 600MHz; D_2O

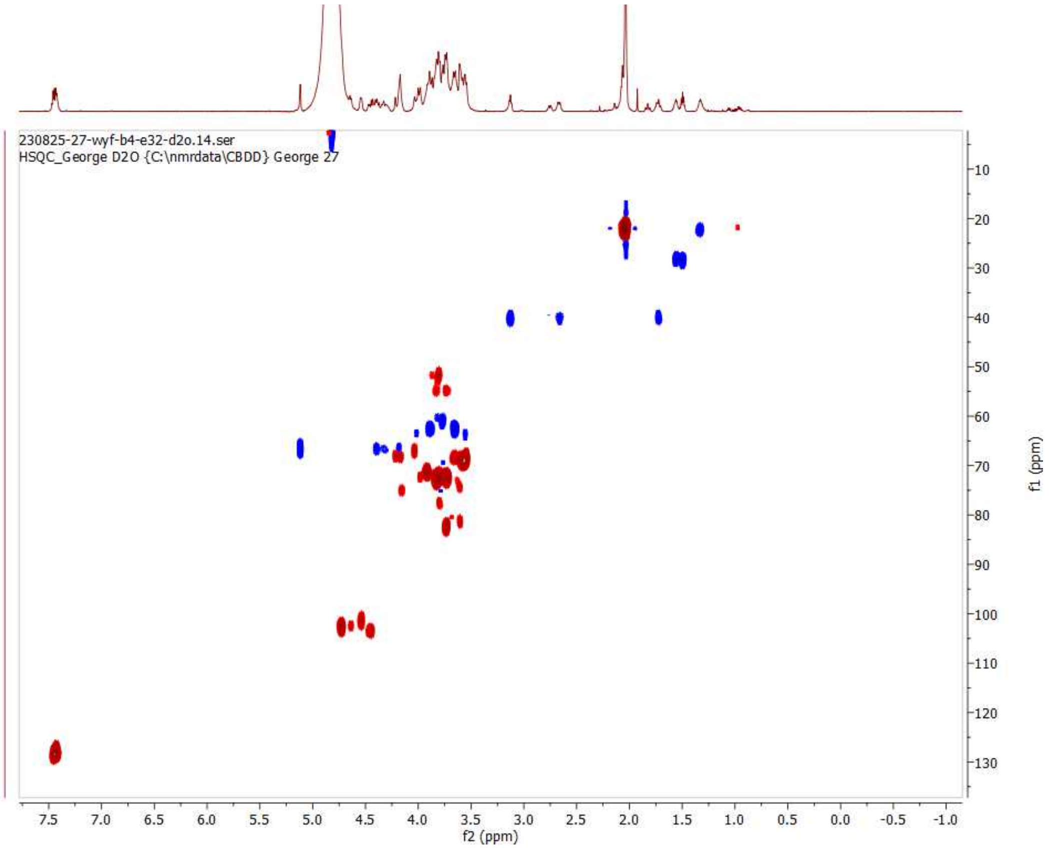

HSQC of 41; 600 MHz/150 MHz, D_2O

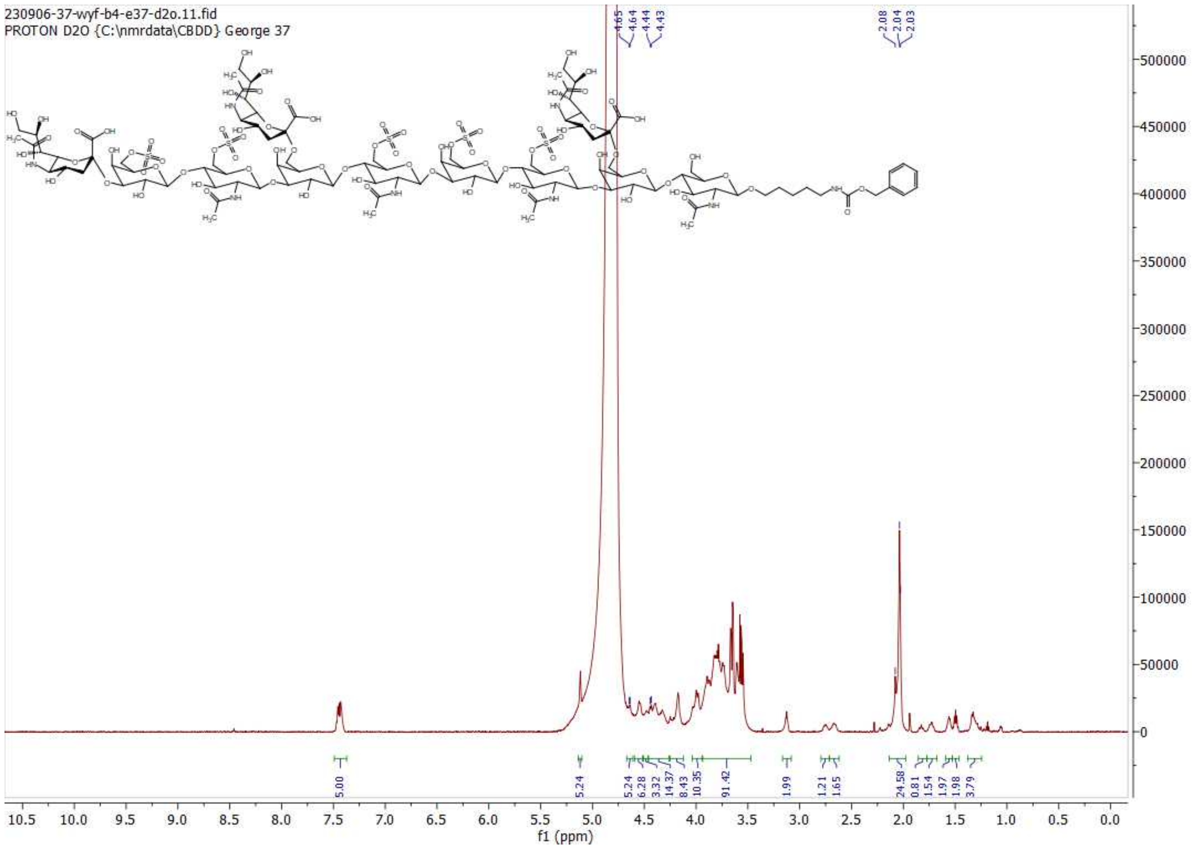

^{1}H NMR of 42; 600MHz; D_2O

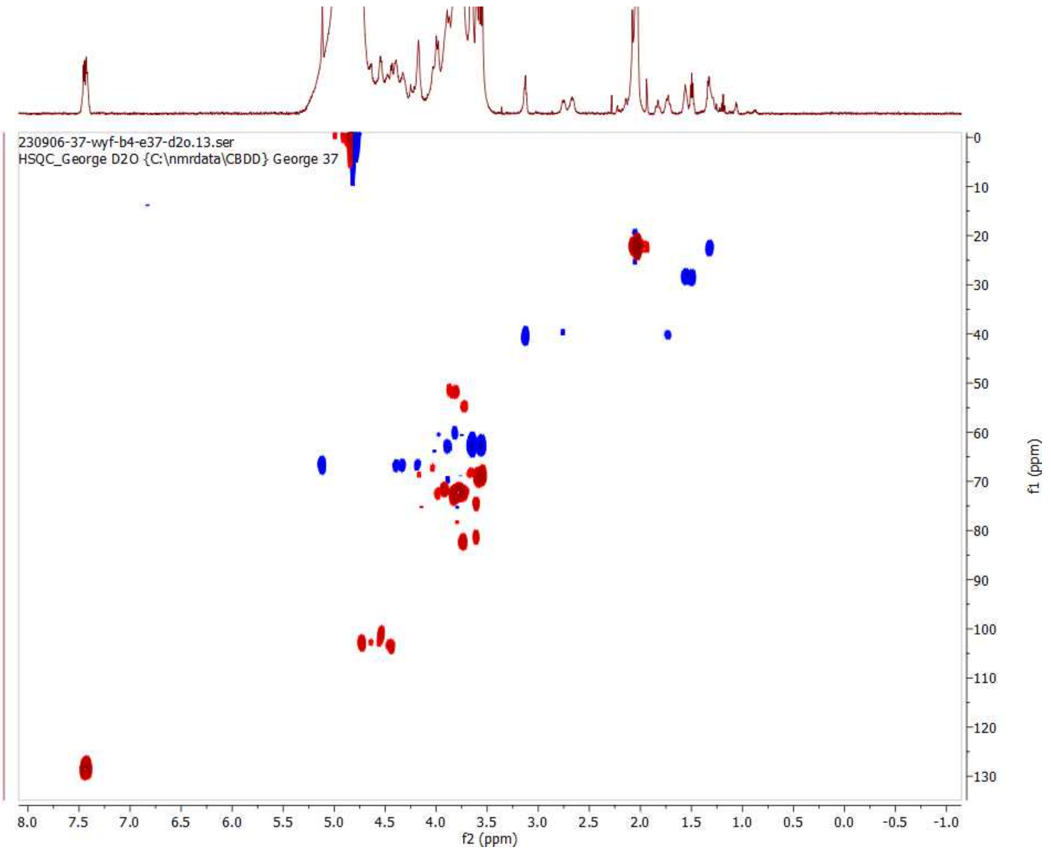

HSQC of 42; 600 MHz/150 MHz, D_2O

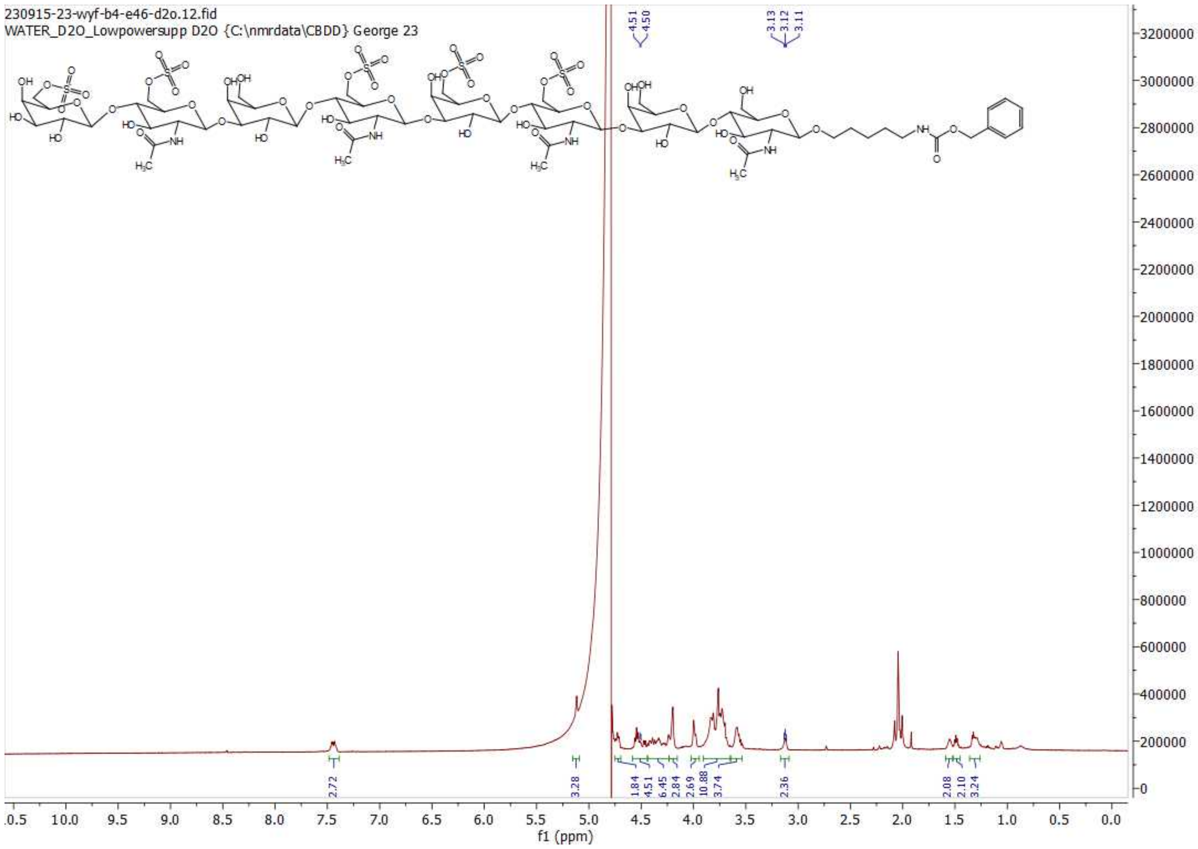

^{1}H NMR of 43; 600MHz; D_2O

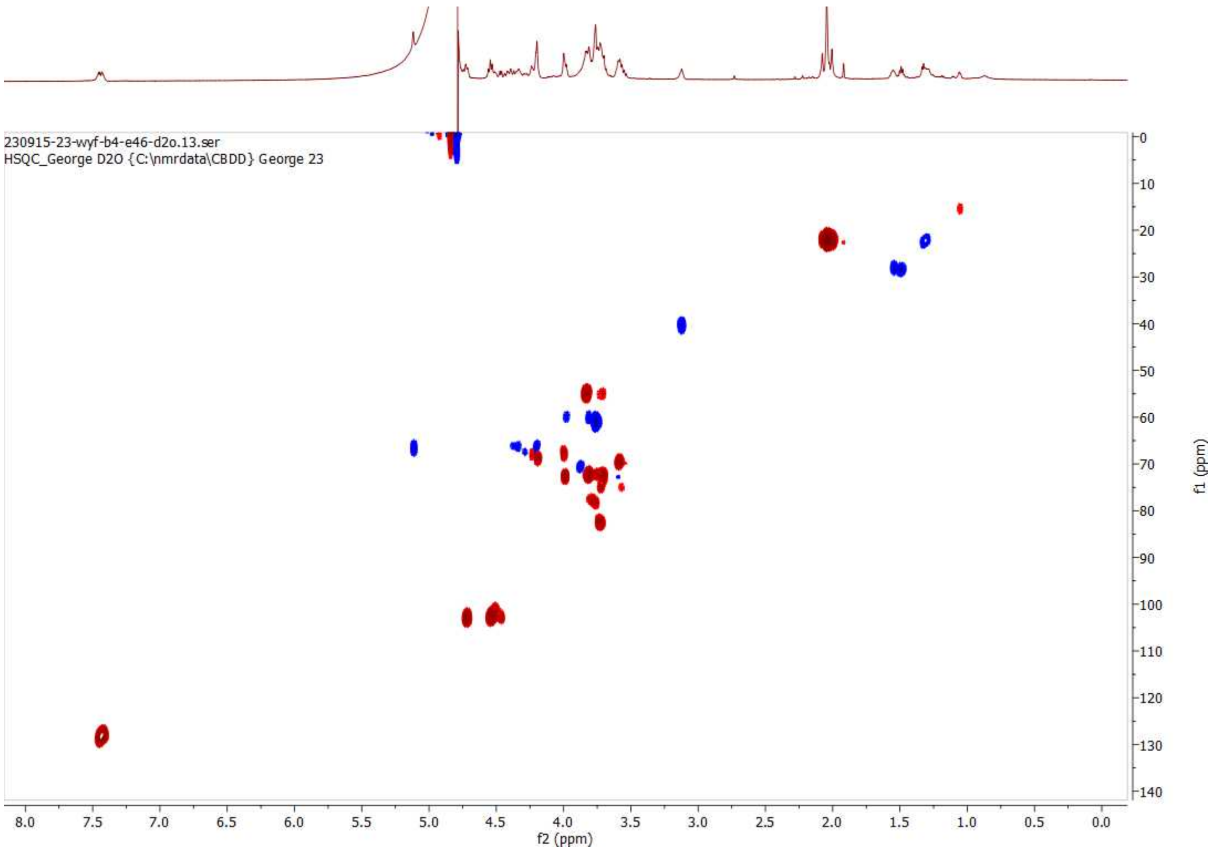

HSQC of 43; 600 MHz/150 MHz, D_2O

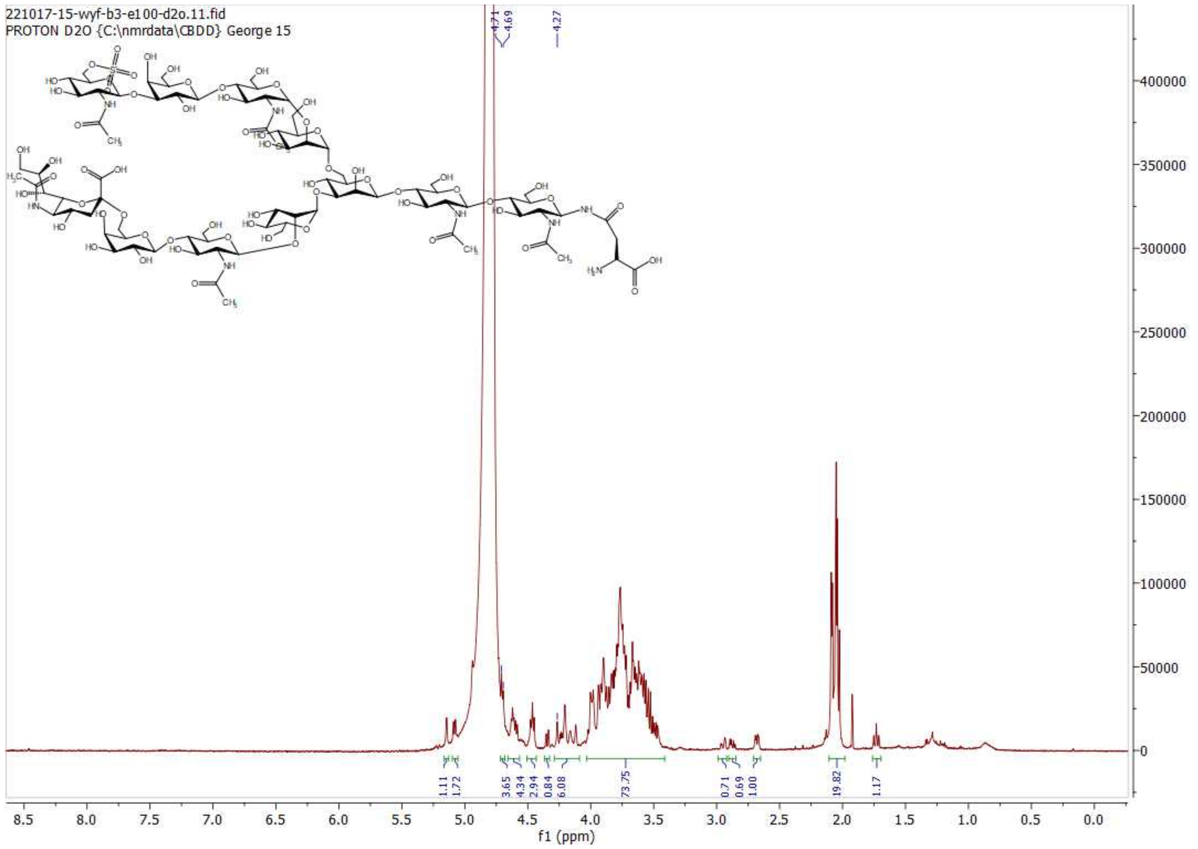

^{1}H NMR of 46; 600MHz; D_2O

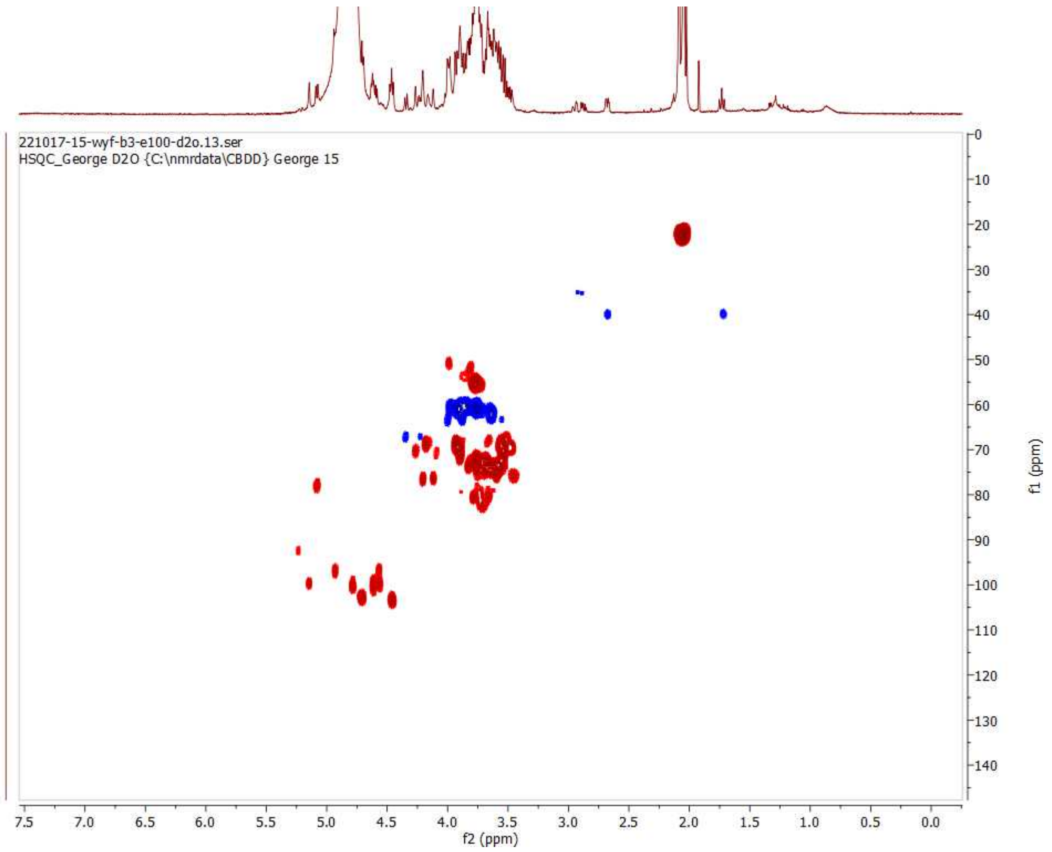

HSQC of 46; 600 MHz/150 MHz, D_2O

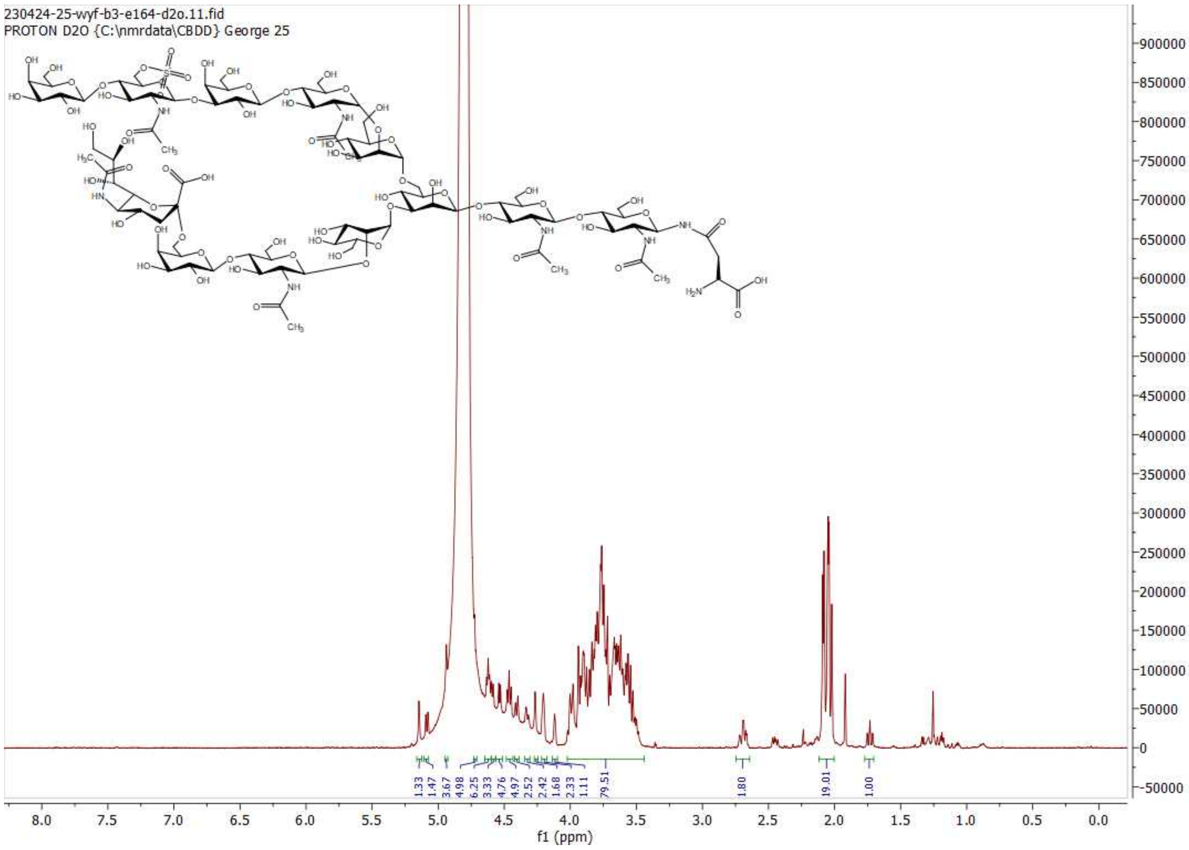

^{1}H NMR of 47; 600MHz; D_2O

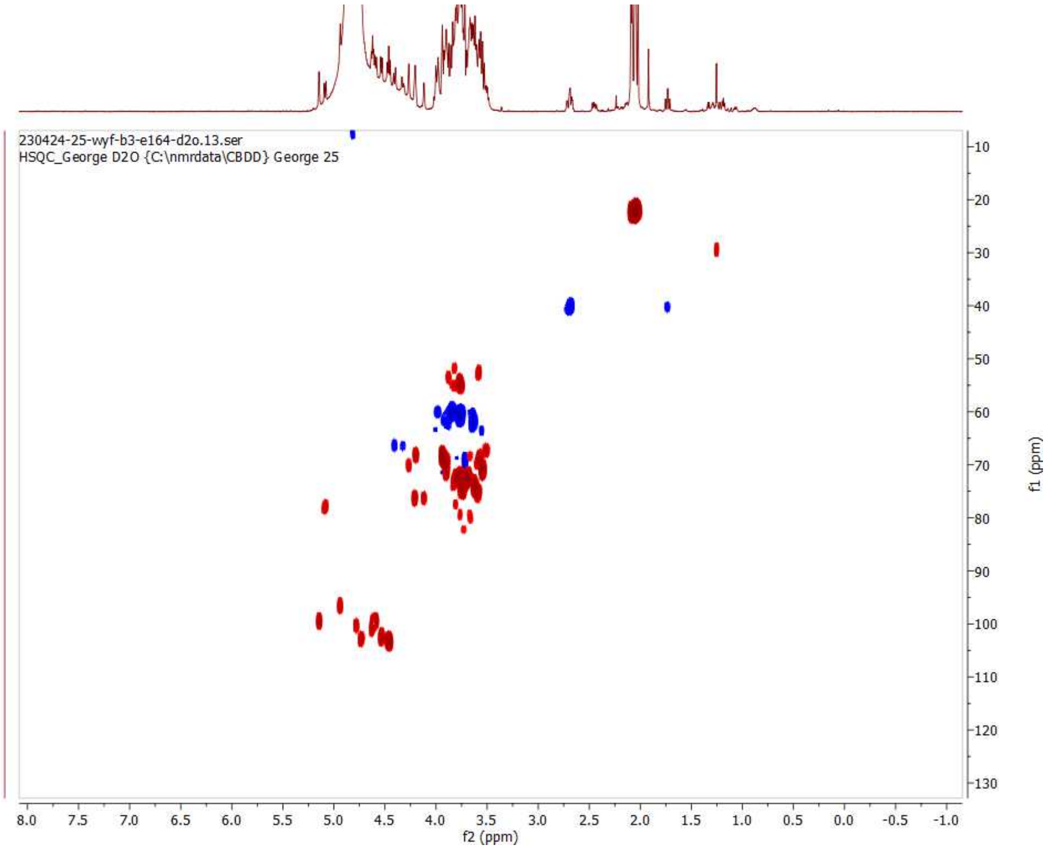

HSQC of 47; 600 MHz/150 MHz, D_2O

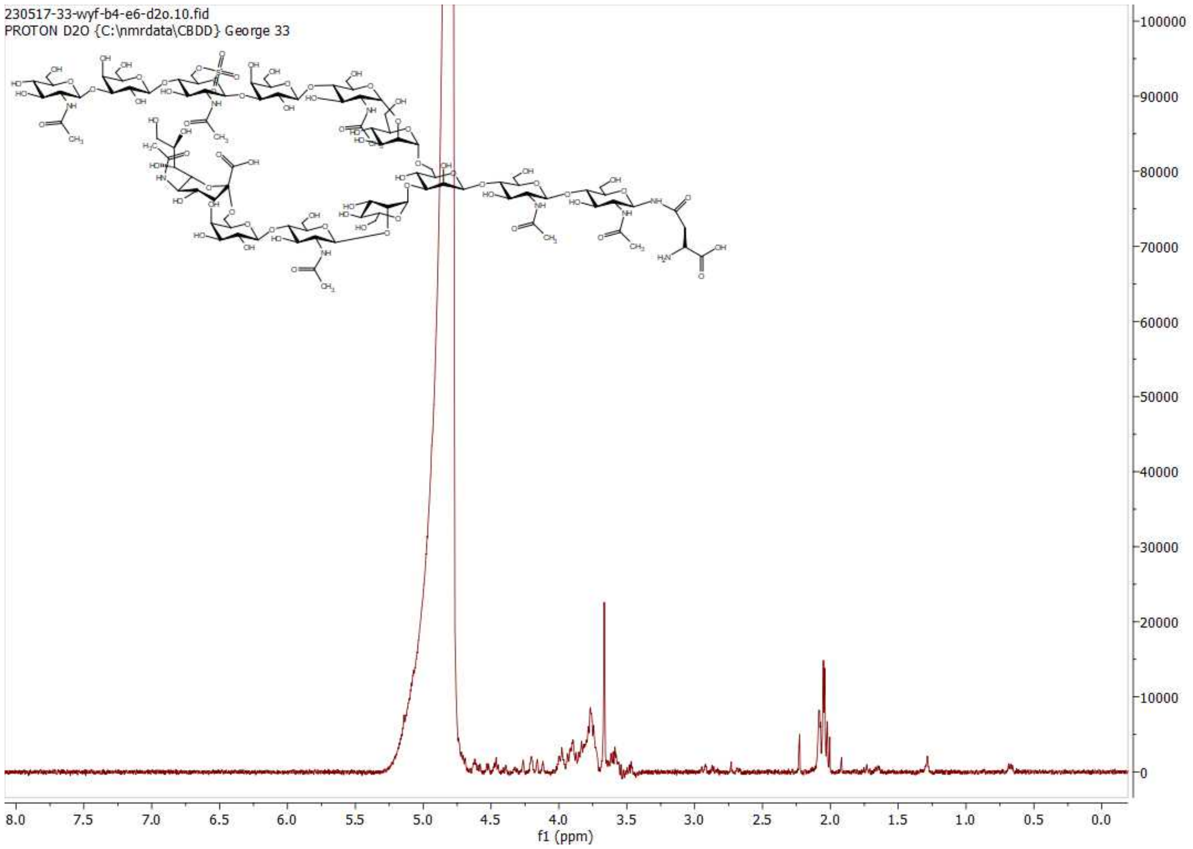

^{1}H NMR of 48; 600MHz; D_2O

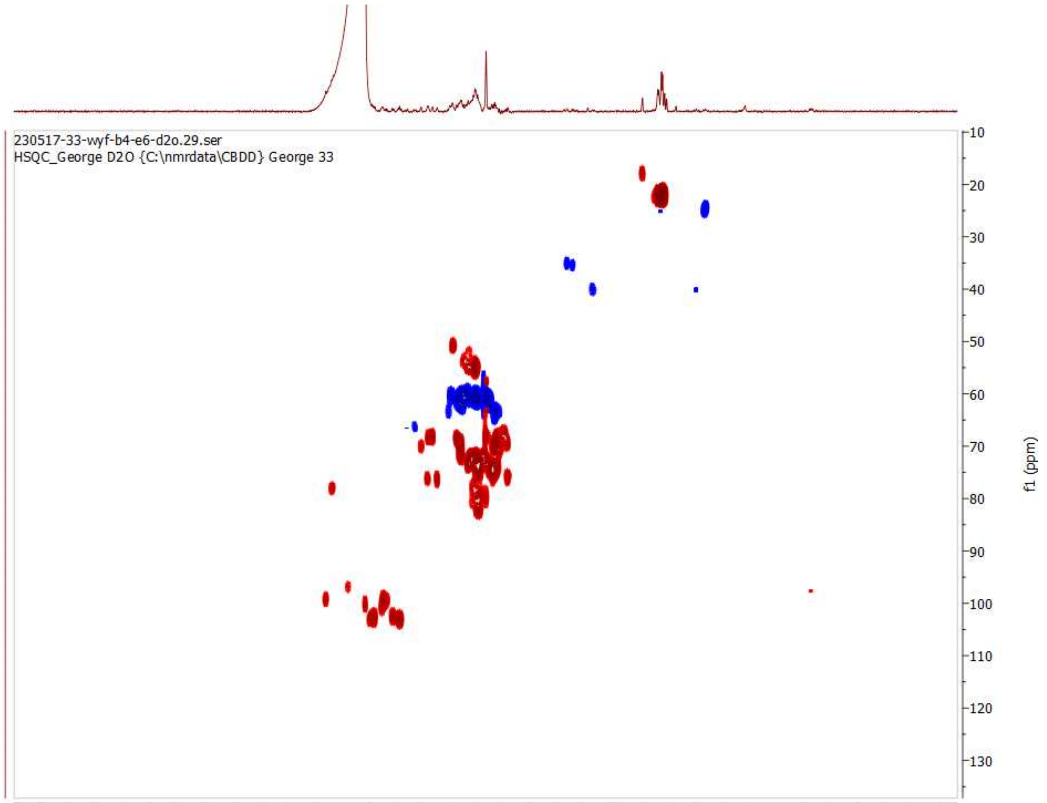

HSQC of 48; 600 MHz/150 MHz, D_2O

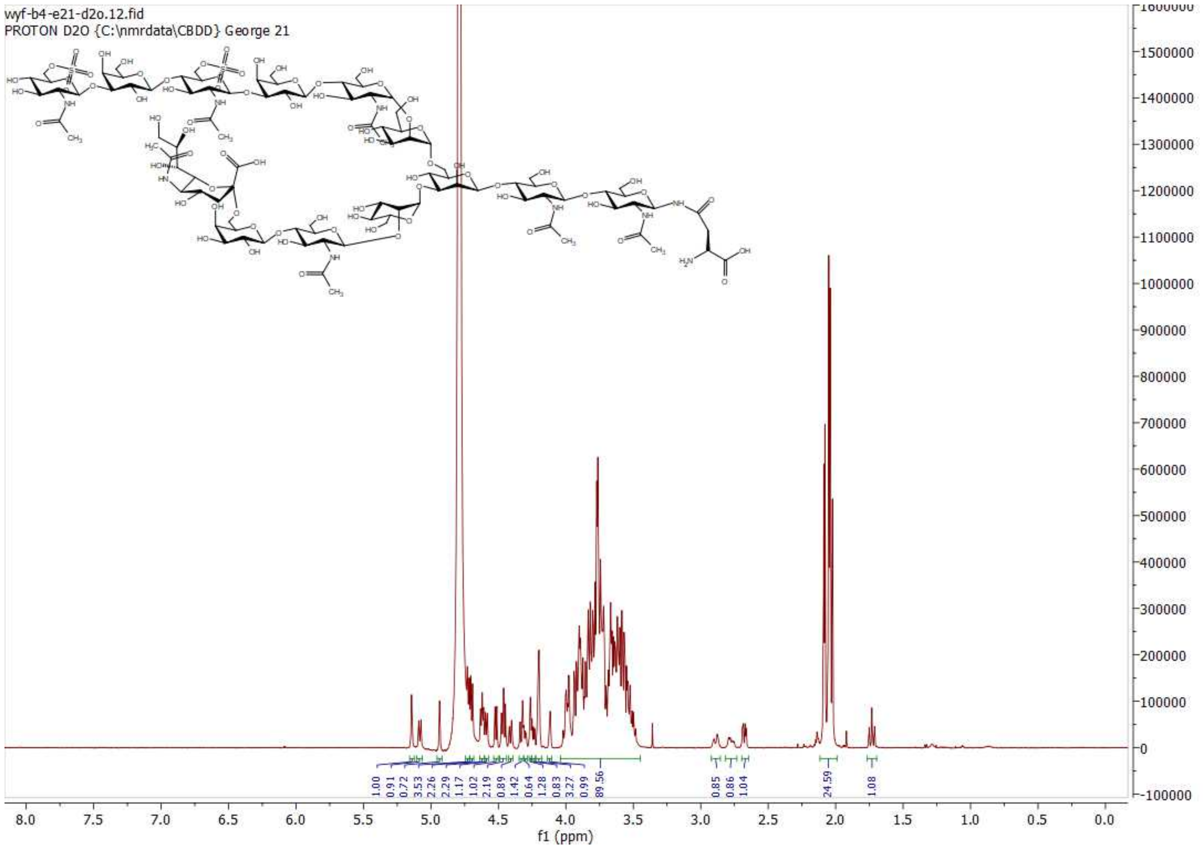

^{1}H NMR of 49; 600MHz; D_2O

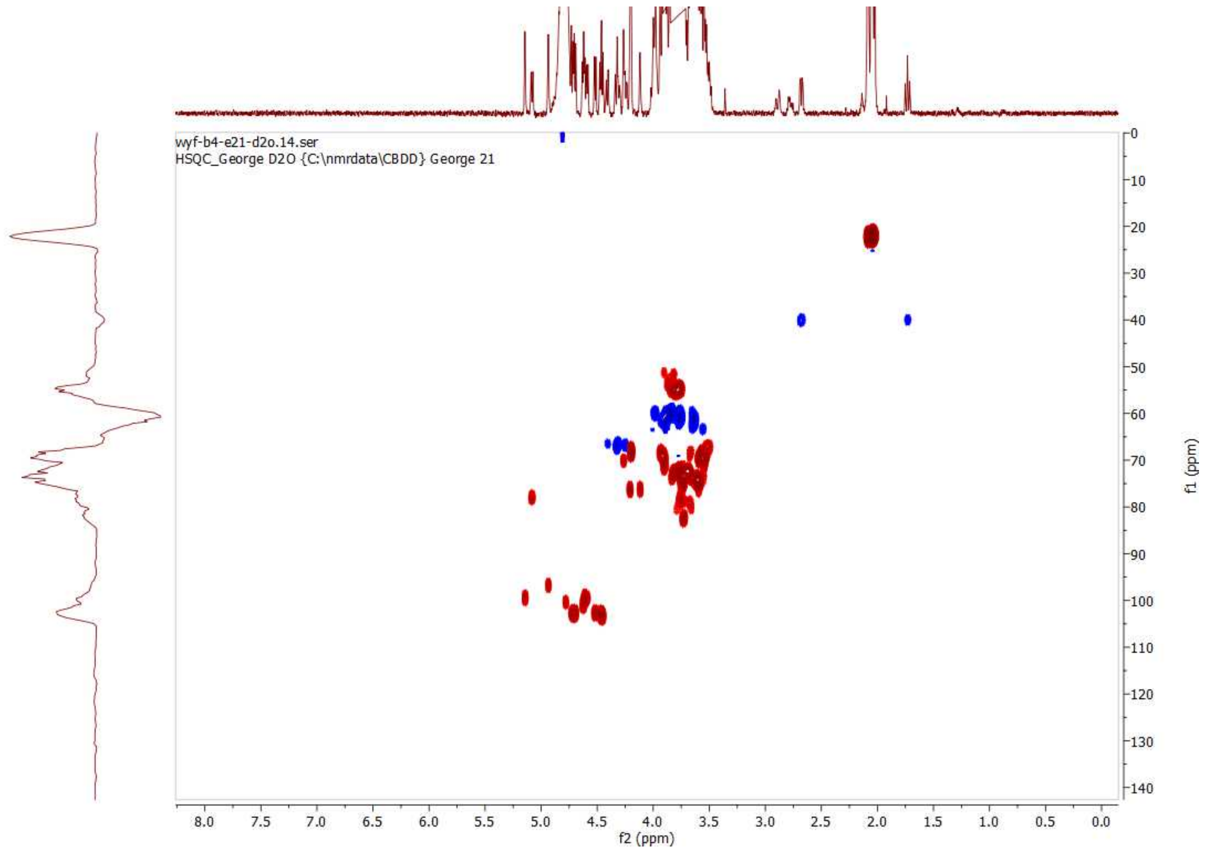

HSQC of 49; 600 MHz/150 MHz, D_2O

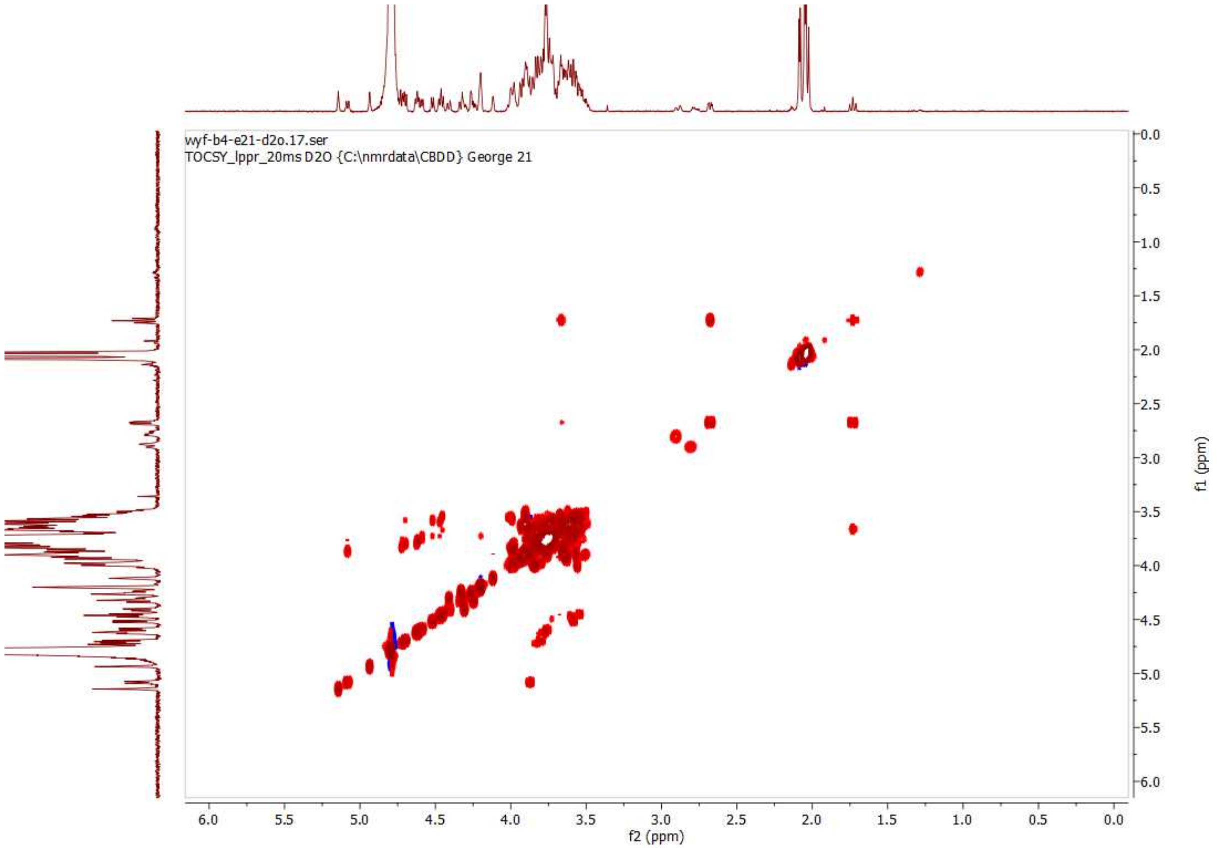

TOCSY (20 ms) of 49; 600MHz; D_2O

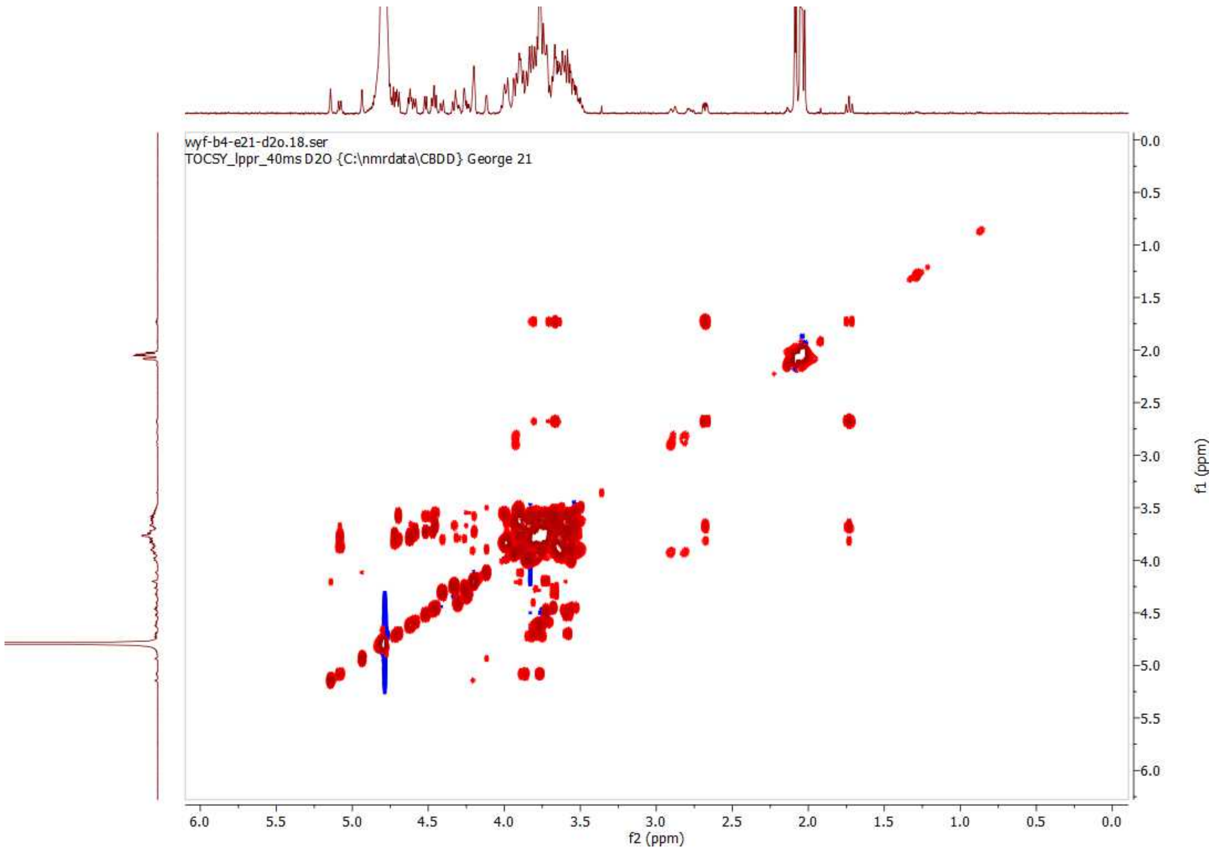

TOCSY (40 ms) of 49; 600MHz; D_2O

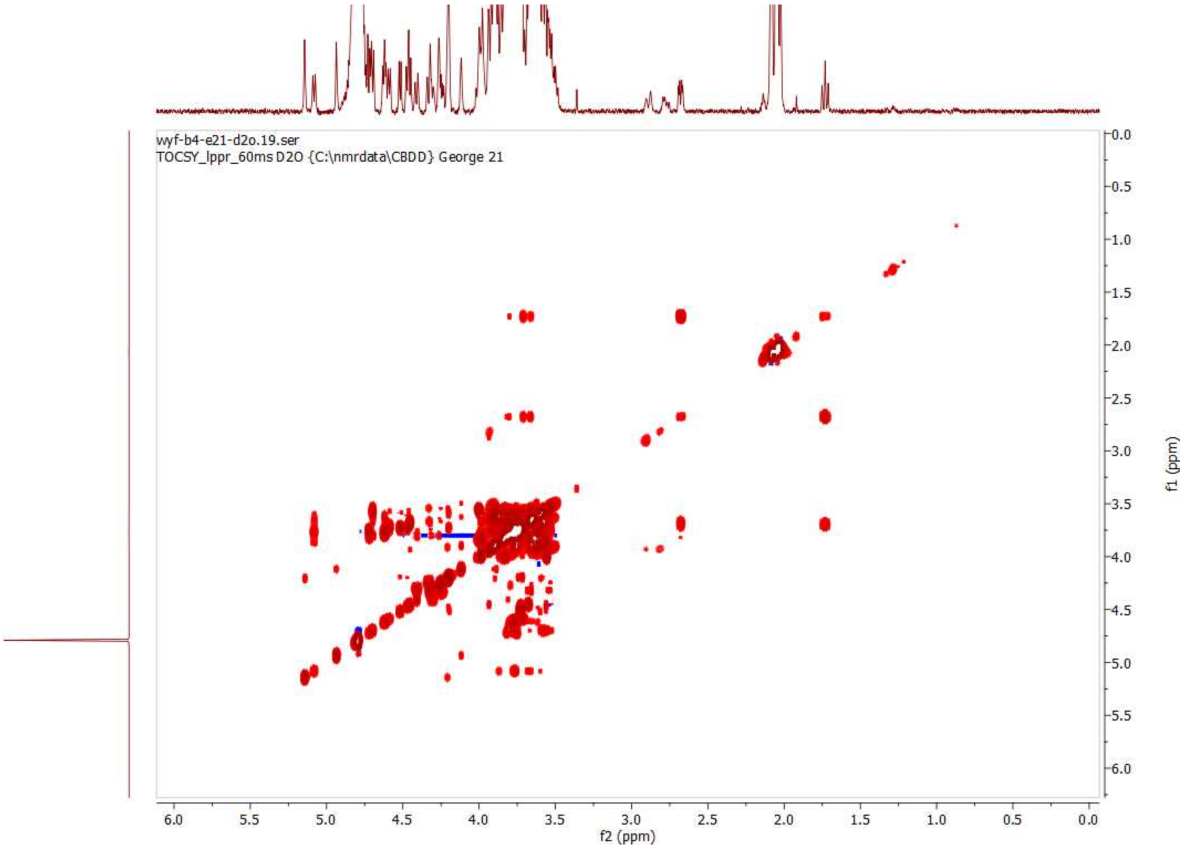

TOCSY (60 ms) of 49; 600MHz; D_2O

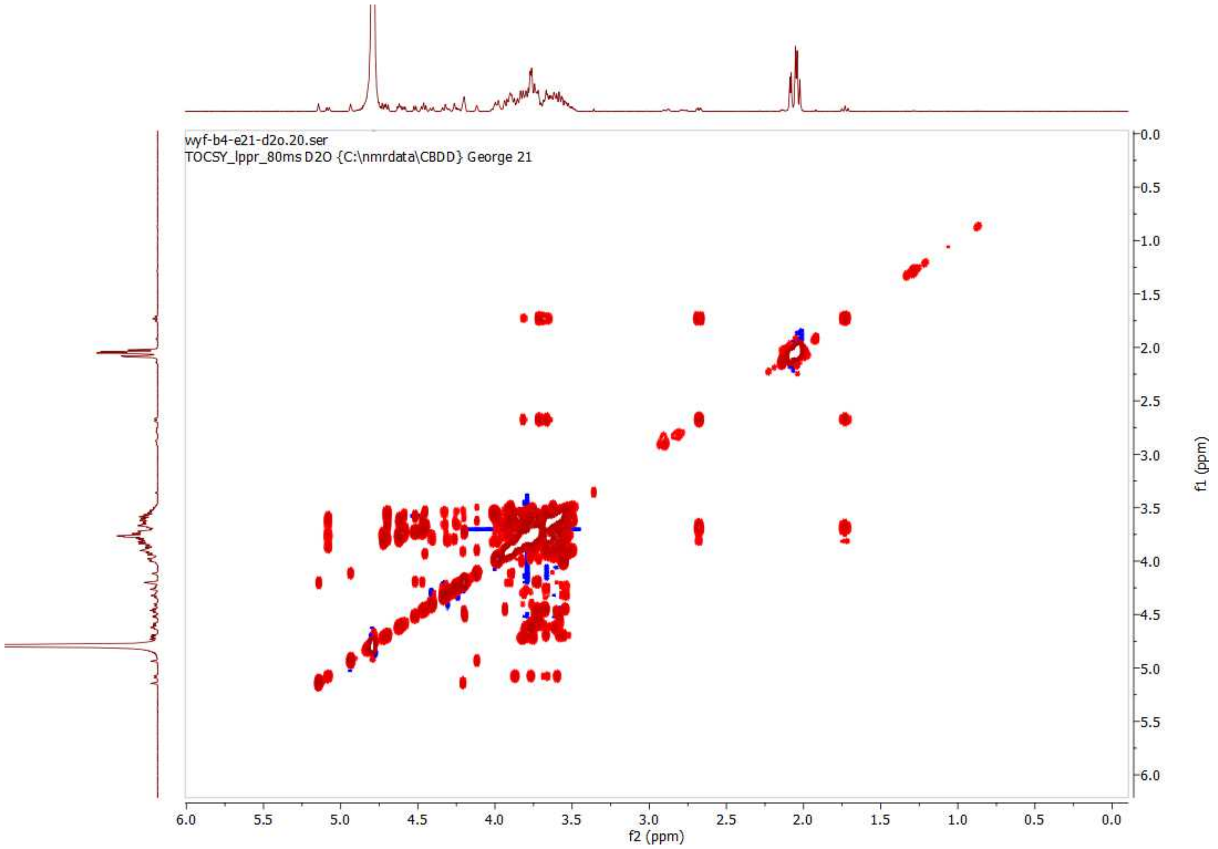

TOCSY (80 ms) of 49; 600MHz; D_2O

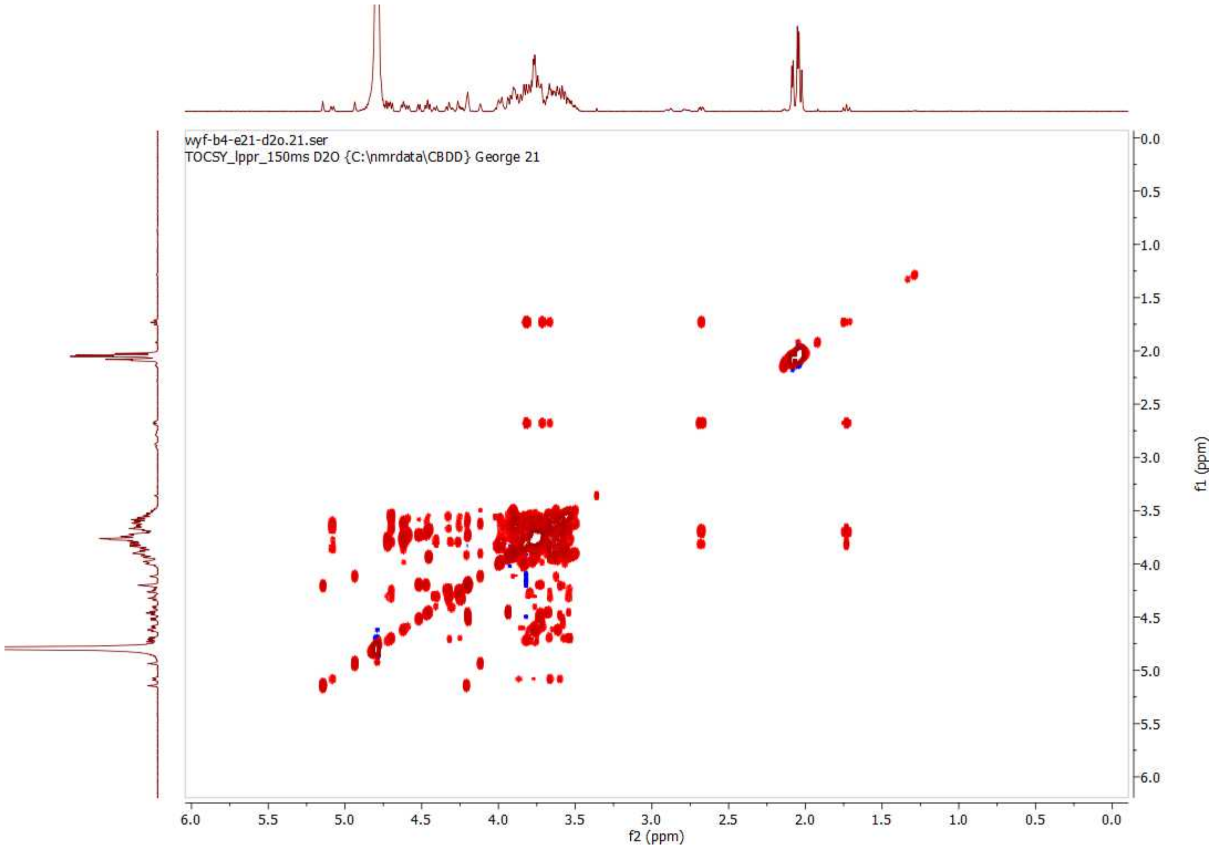

TOCSY (150 ms) of 49; 600MHz; D_2O

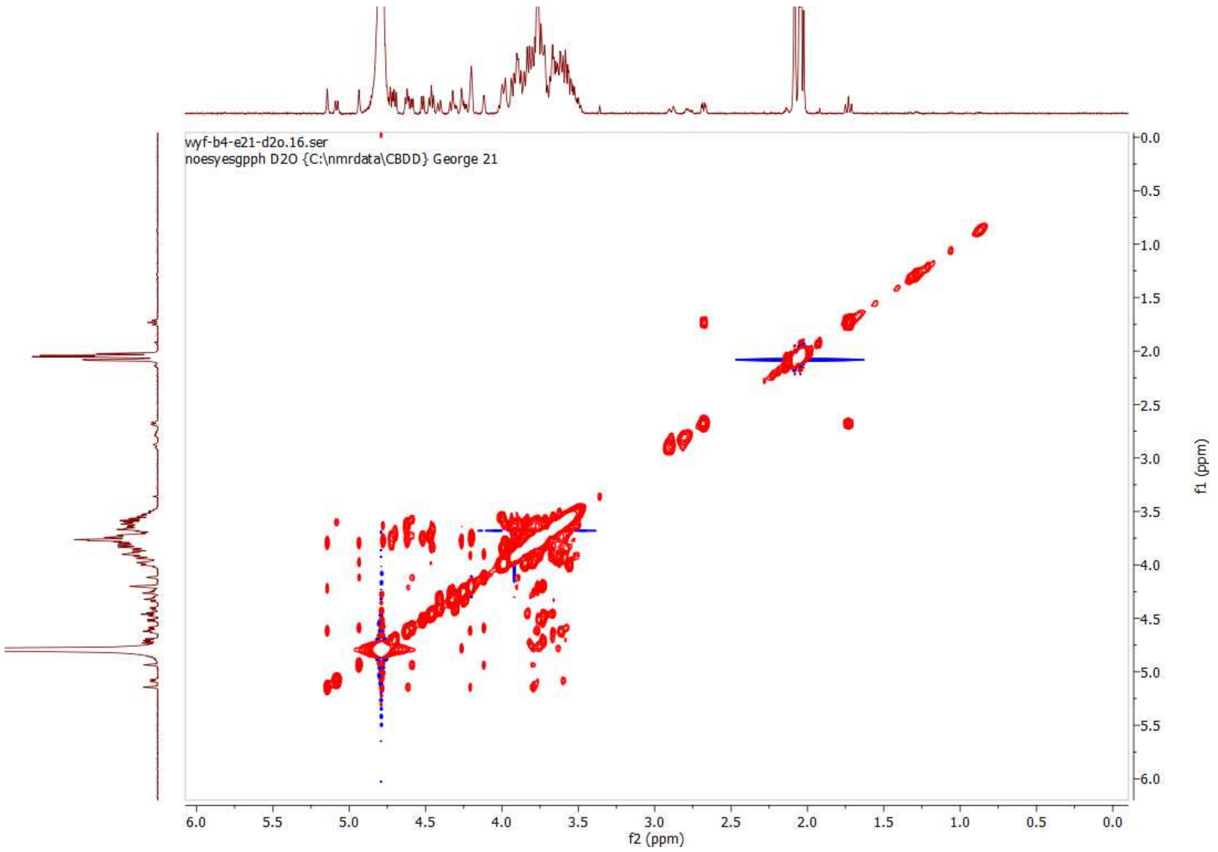

NOESY (300 ms) of 49; 600 MHz, D_2O

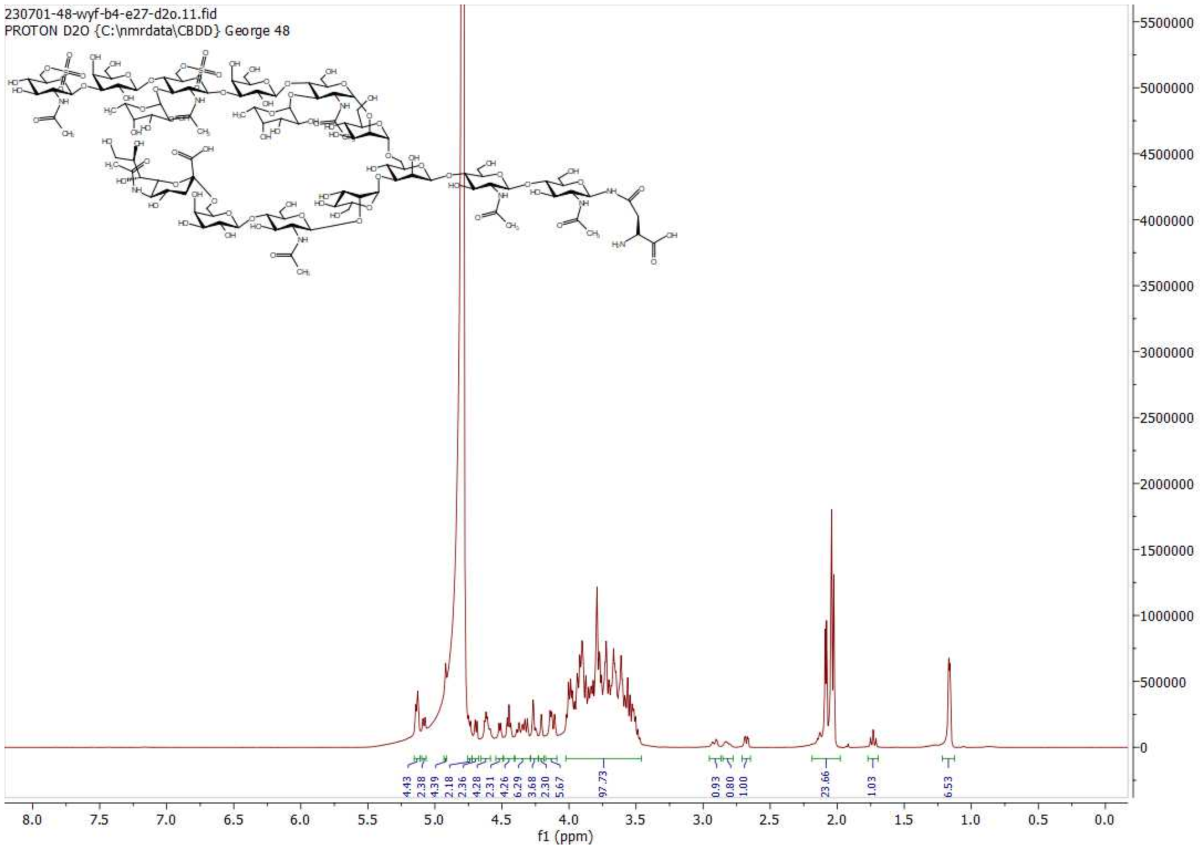

^{1}H NMR of 50; 600MHz; D_2O

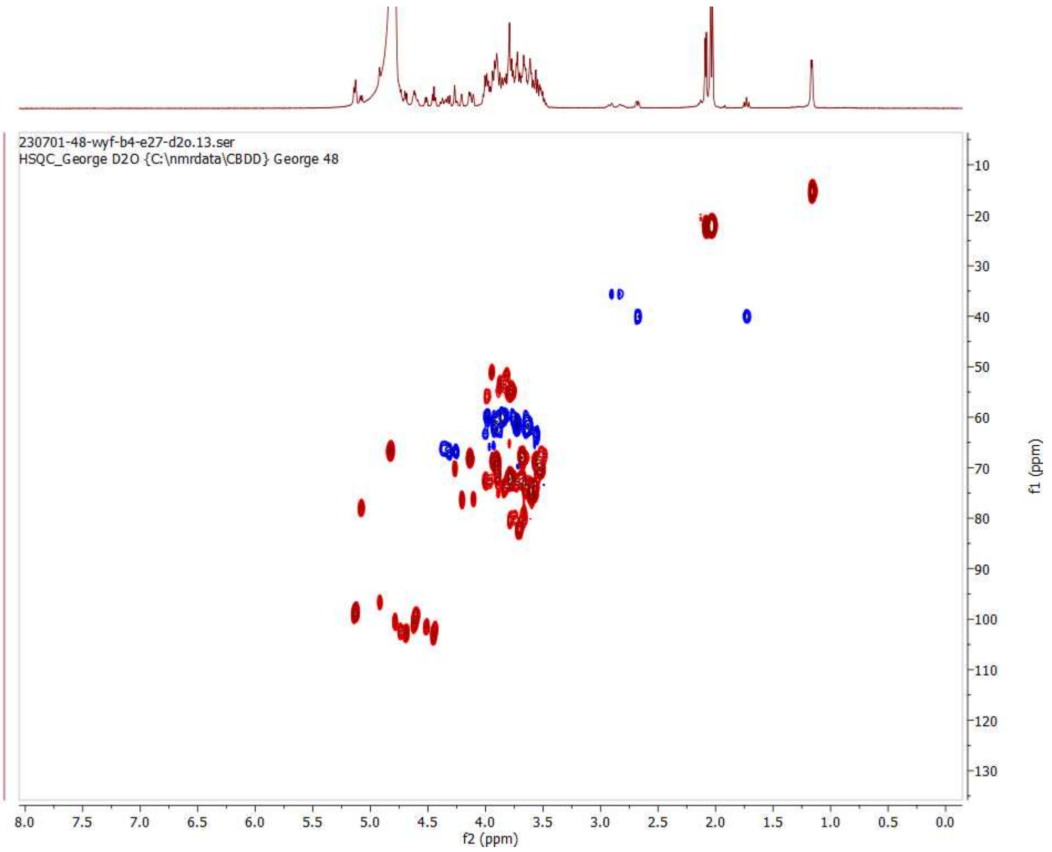

HSQC of 50; 600 MHz/150 MHz, D_2O

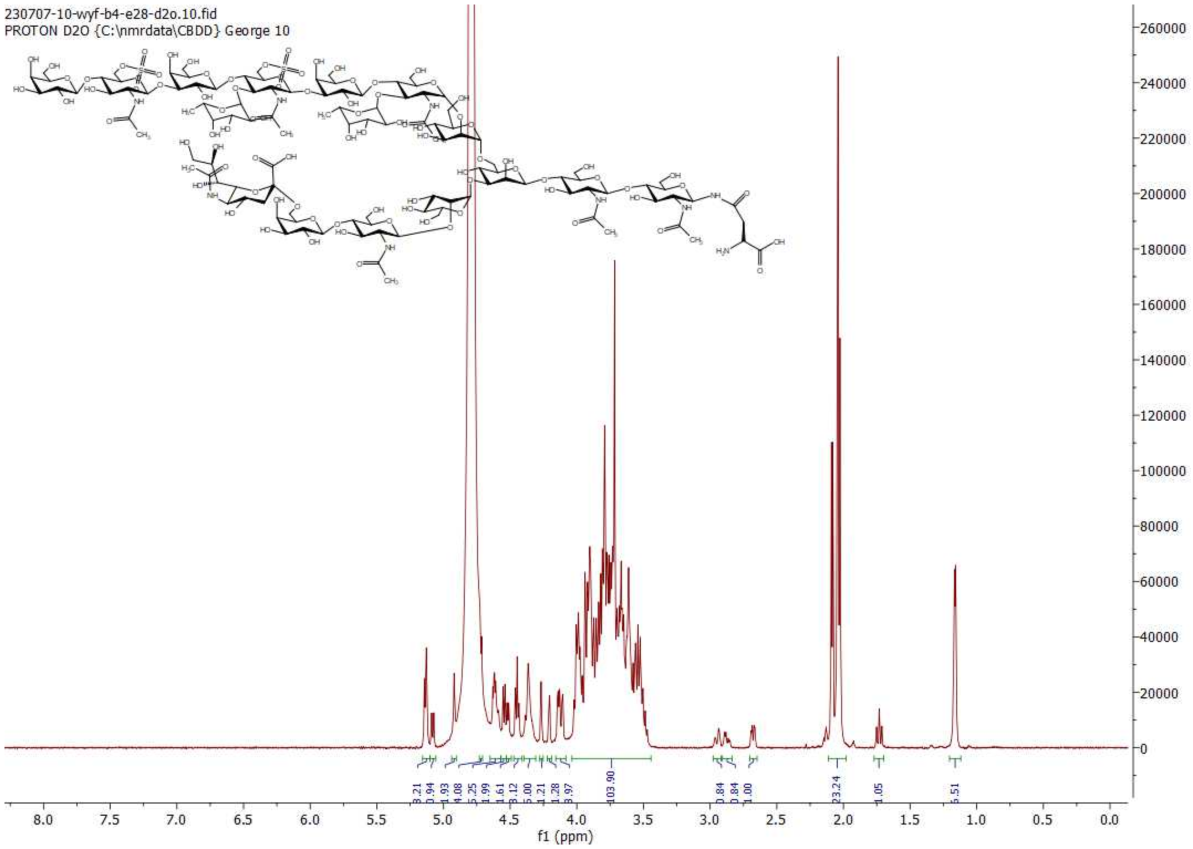

^{1}H NMR of 51; 600MHz; D₂O

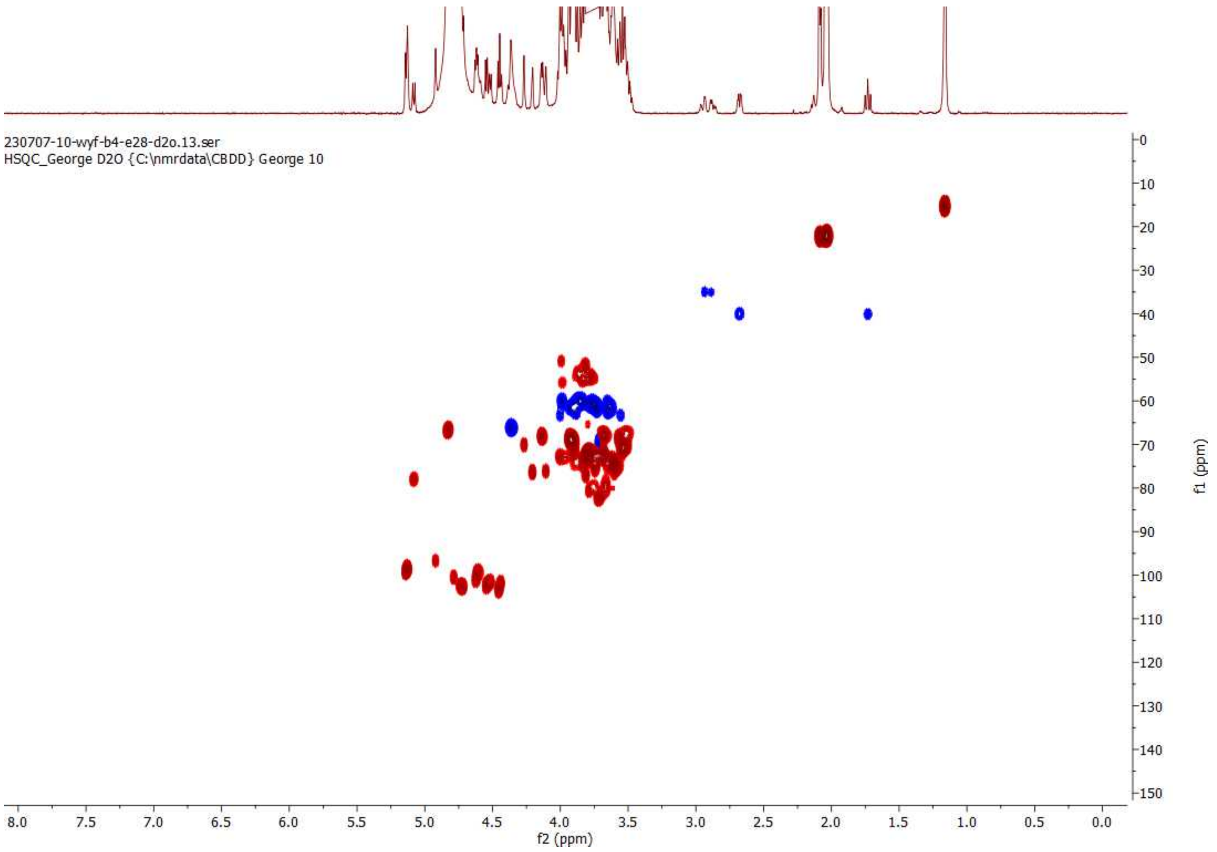

HSQC of 51; 600 MHz/150 MHz, D_2O

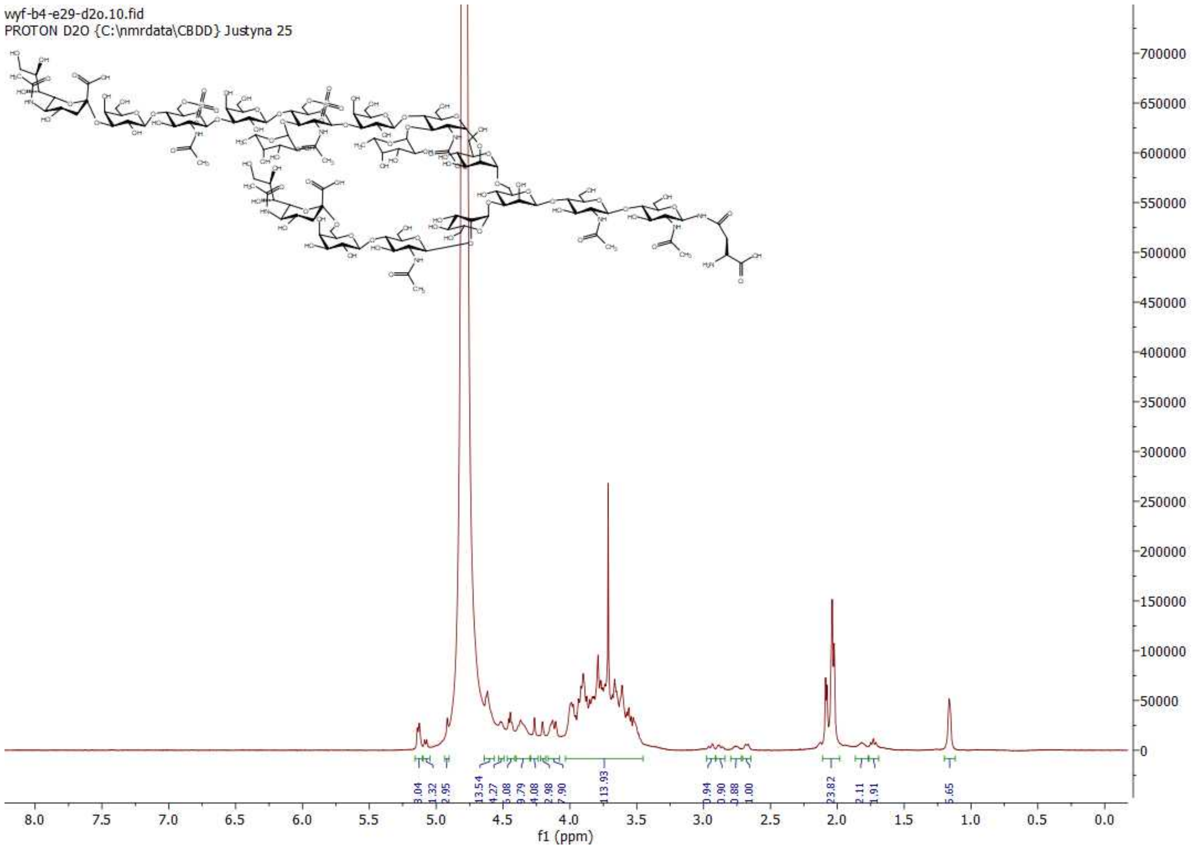

^{1}H NMR of 52; 600MHz; D_2O

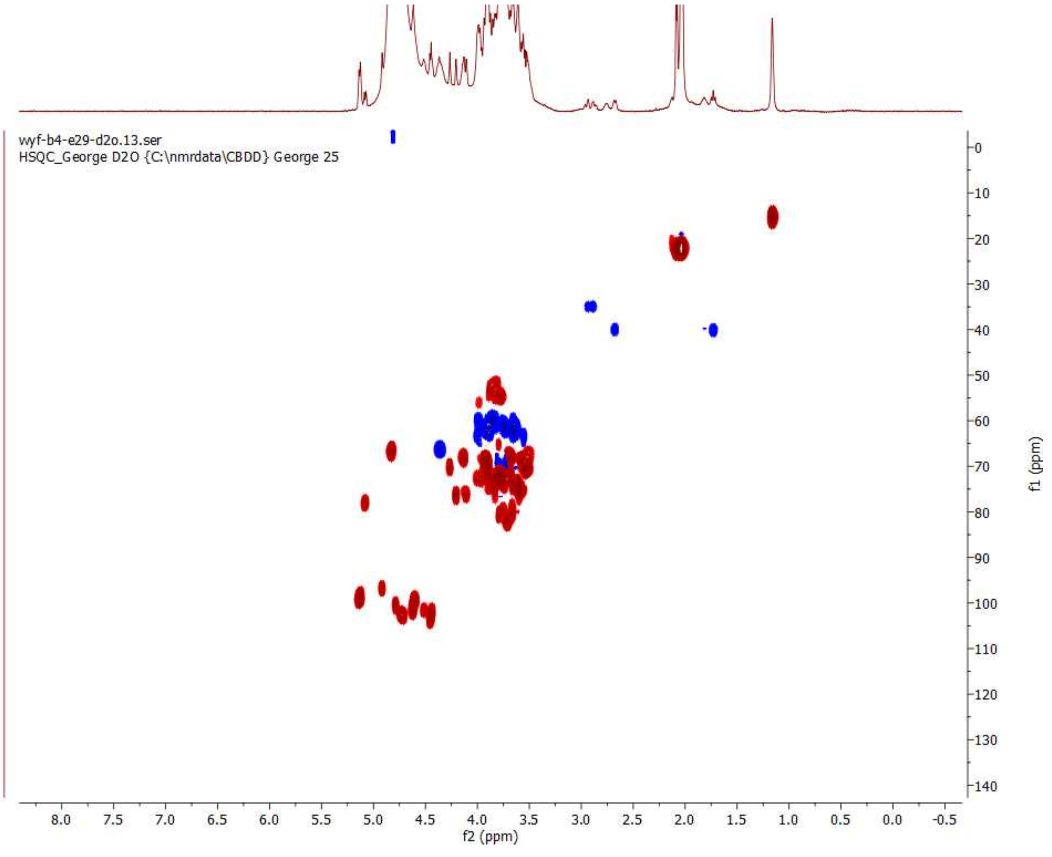

HSQC of 52; 600 MHz/150 MHz, D_2O

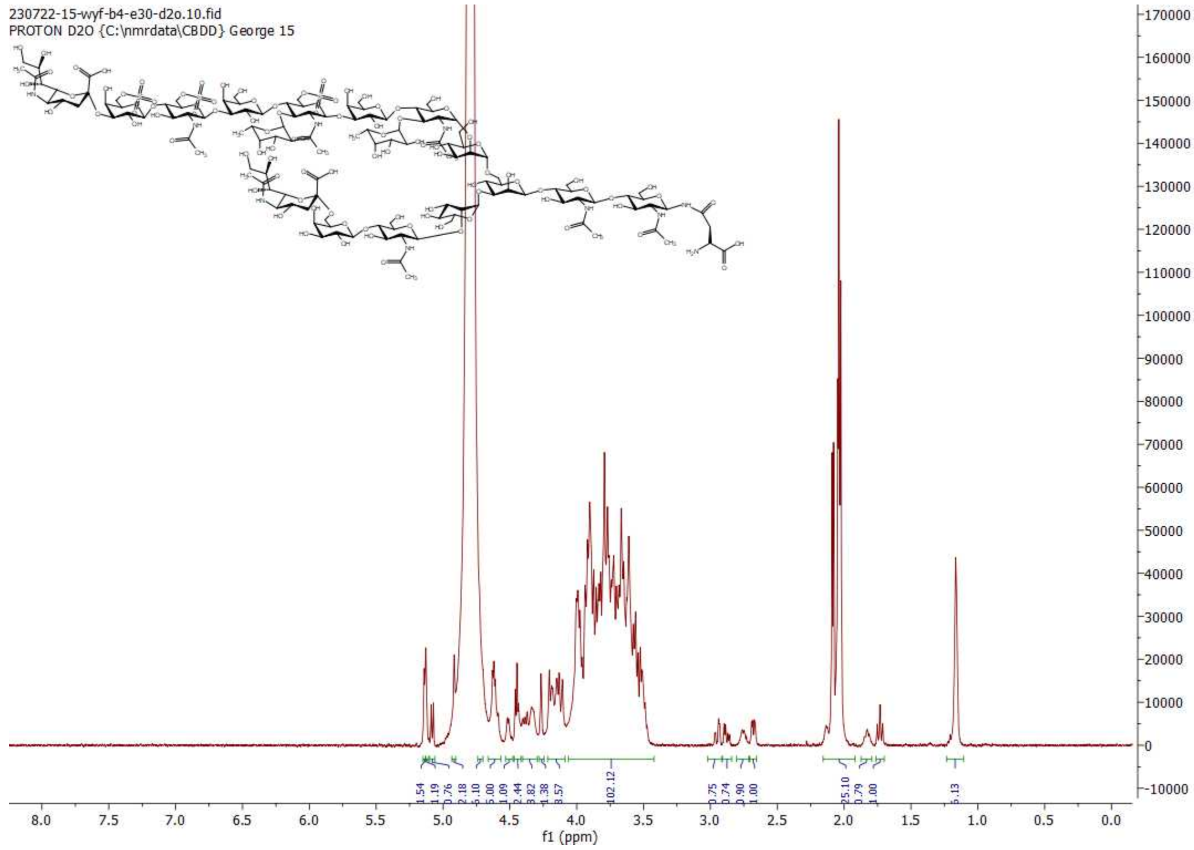

^{1}H NMR of 53; 600MHz; D_2O

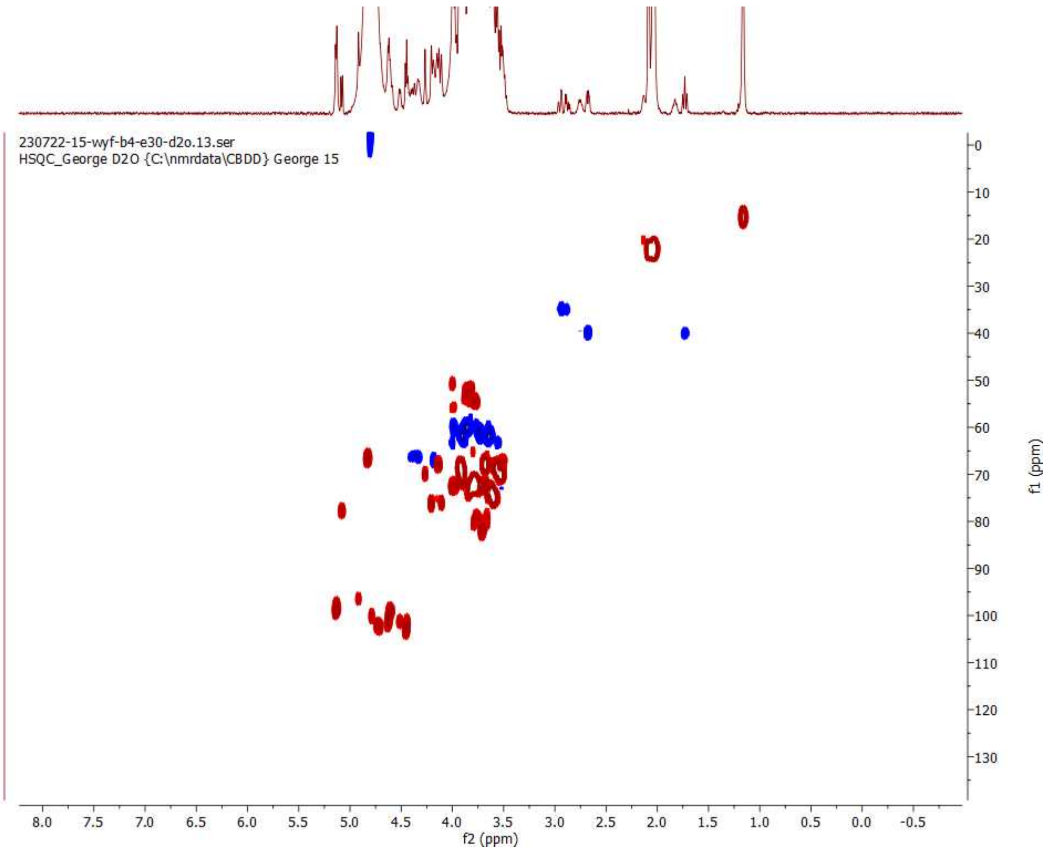

HSQC of 53; 600 MHz/150 MHz, D_2O

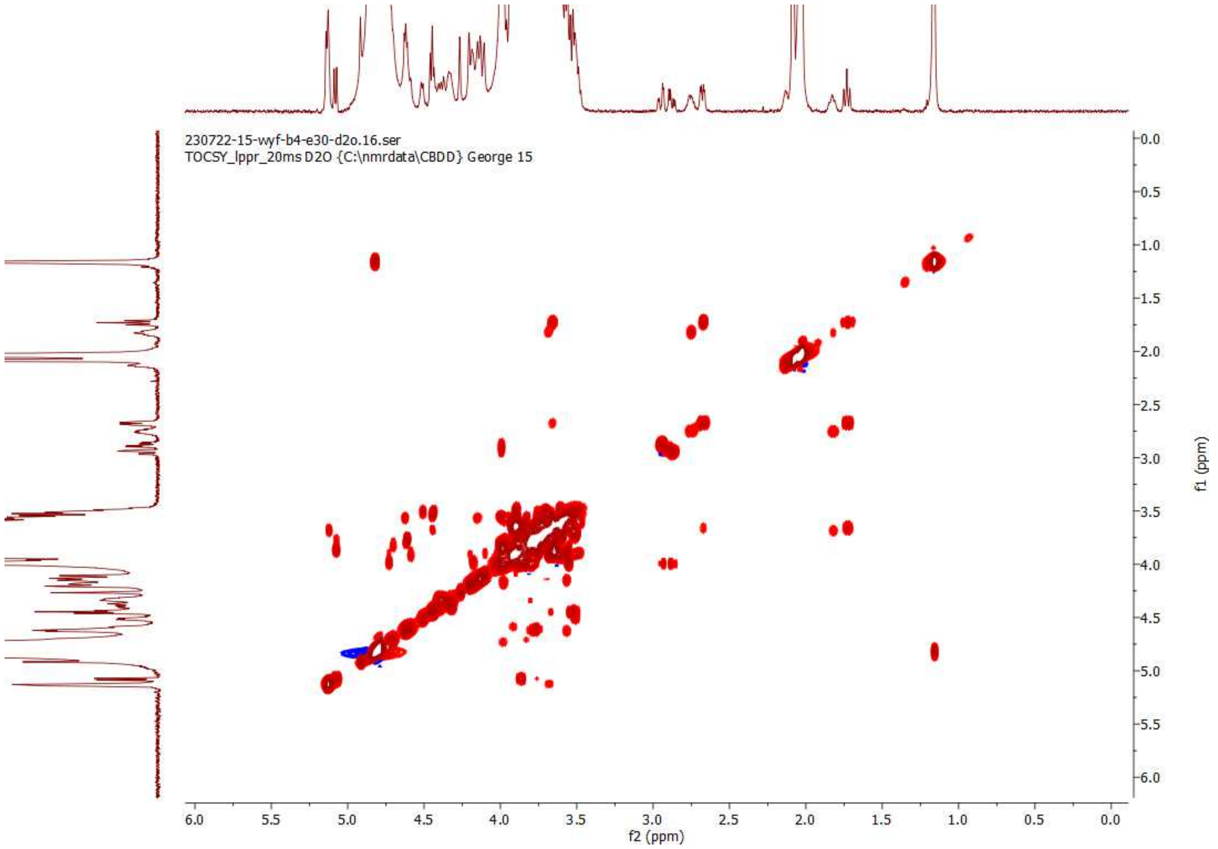

TOCSY (20 ms) of 53; 600MHz; D_2O

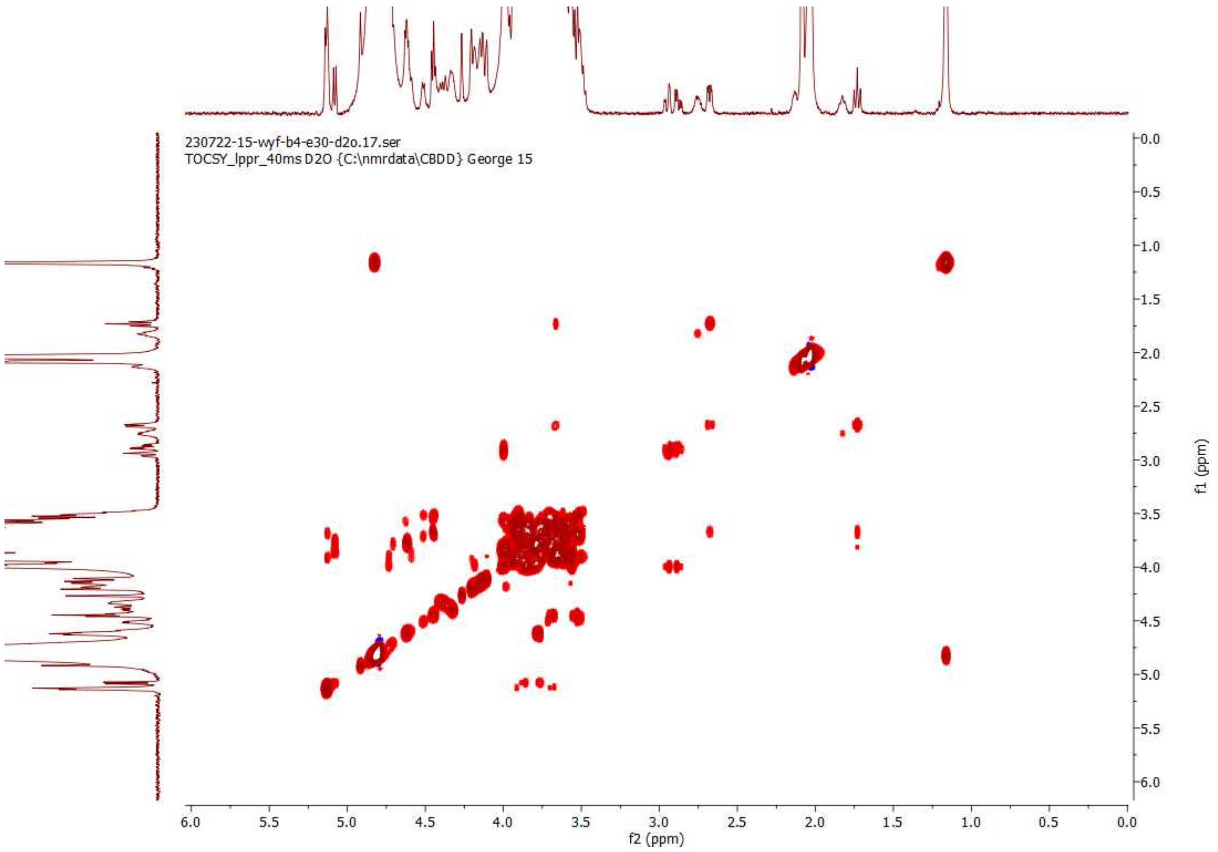

TOCSY (40 ms) of 53; 600MHz; D_2O

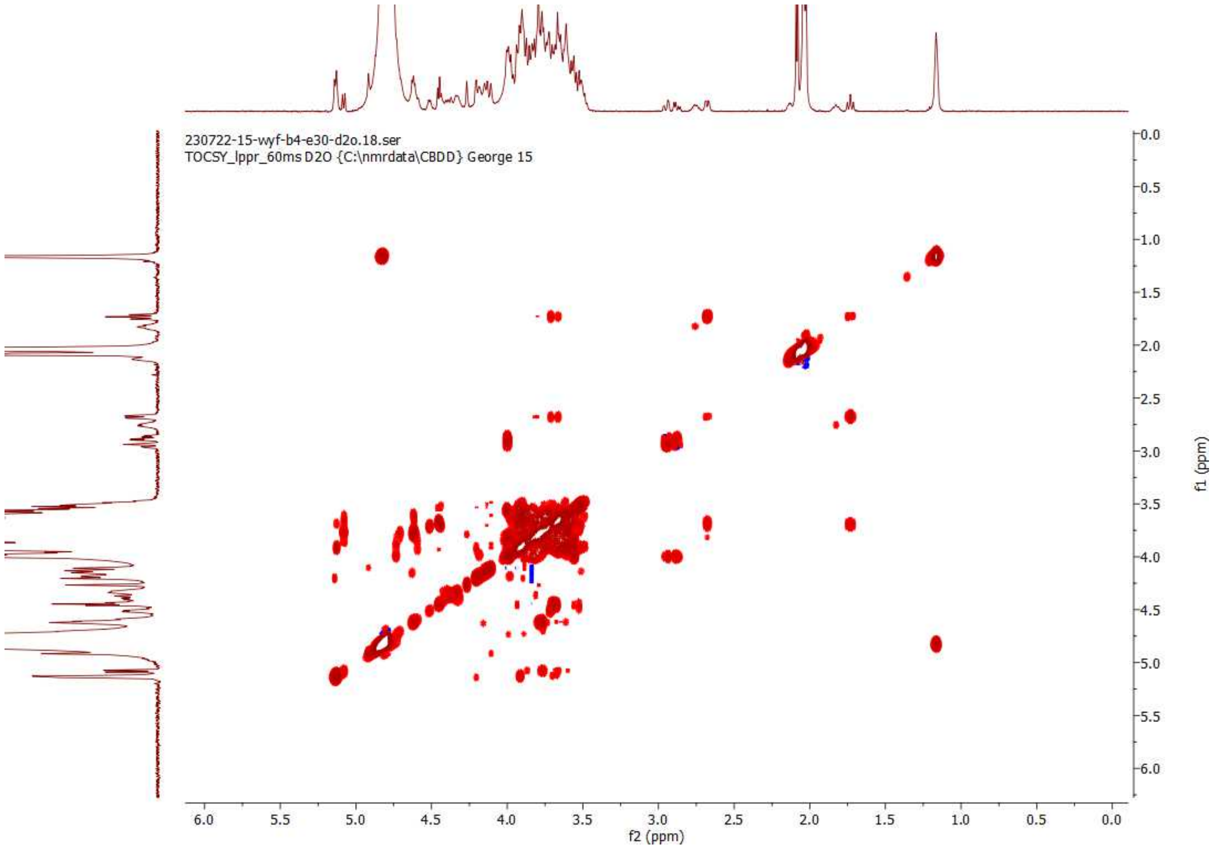

TOCSY (60 ms) of 53; 600MHz; D_2O

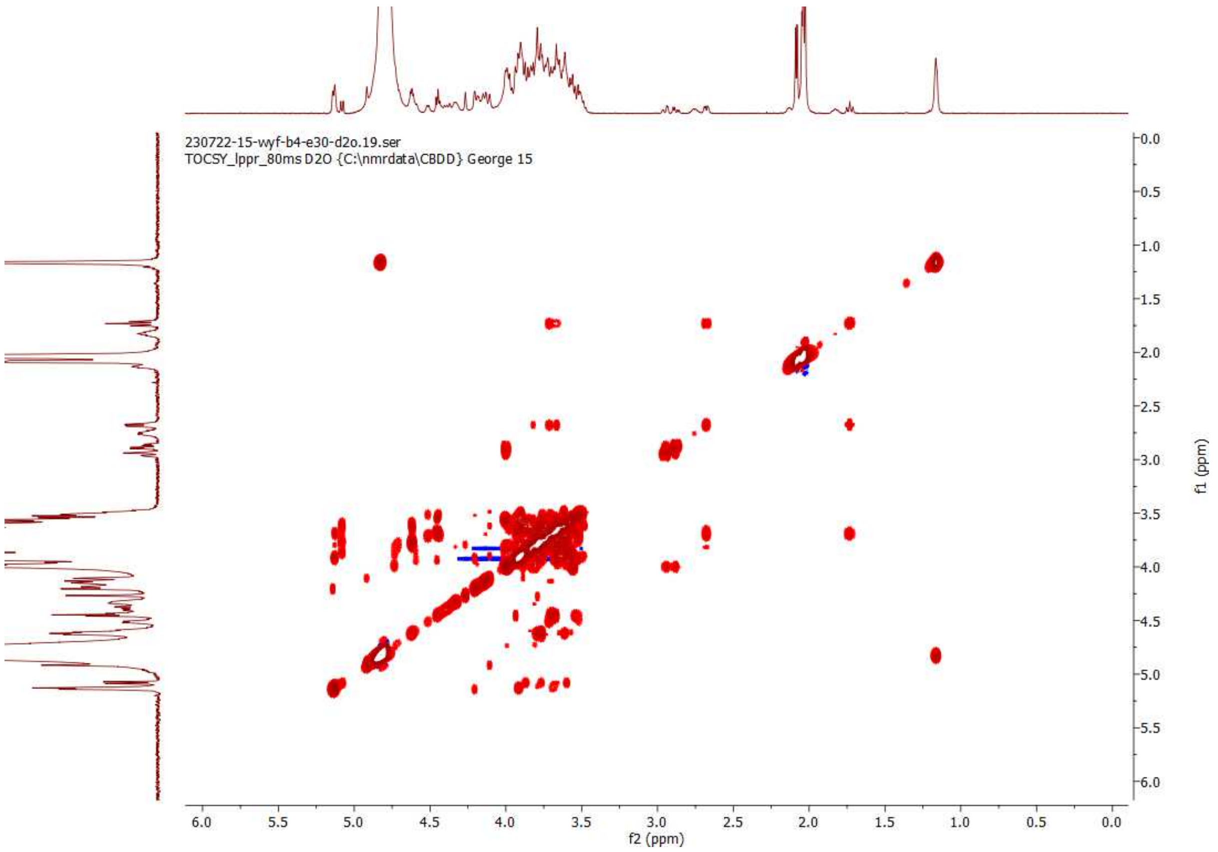

TOCSY (80 ms) of 53; 600MHz; D_2O

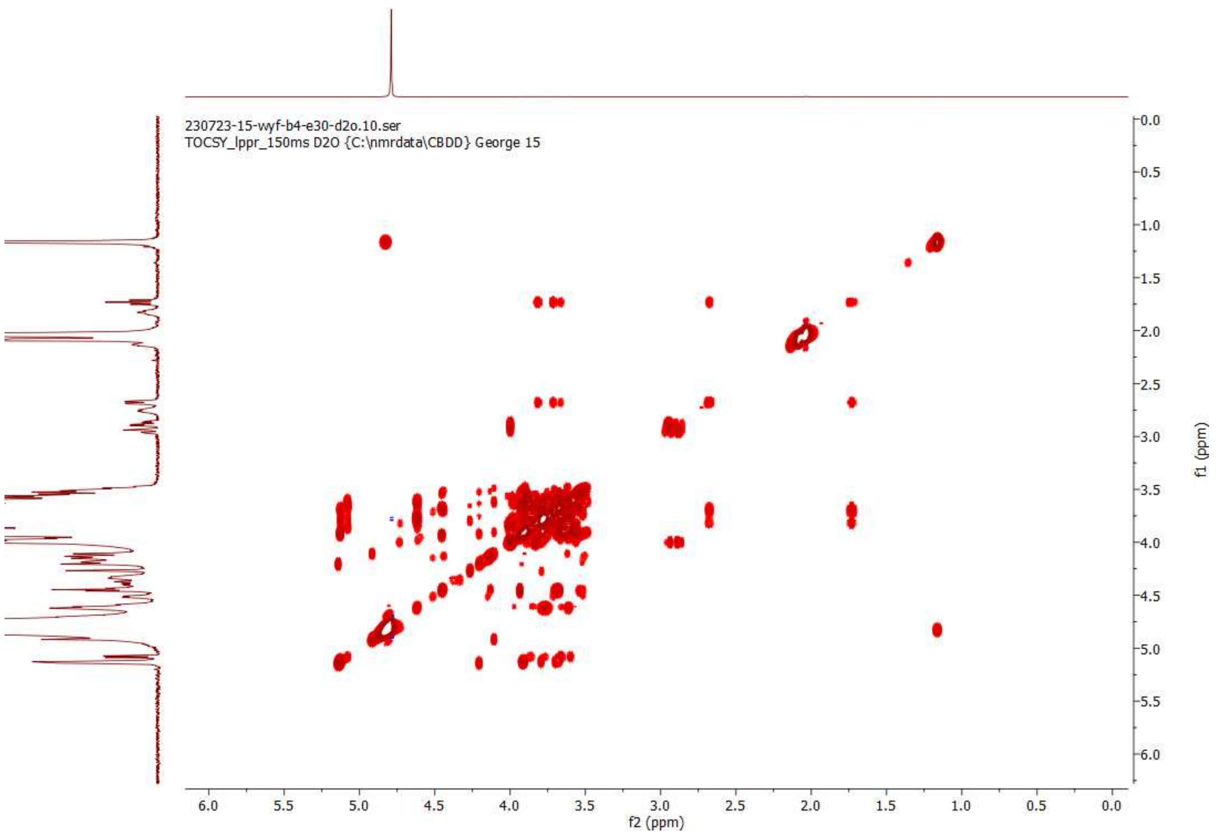

TOCSY (150 ms) of 53; 600MHz; D_2O

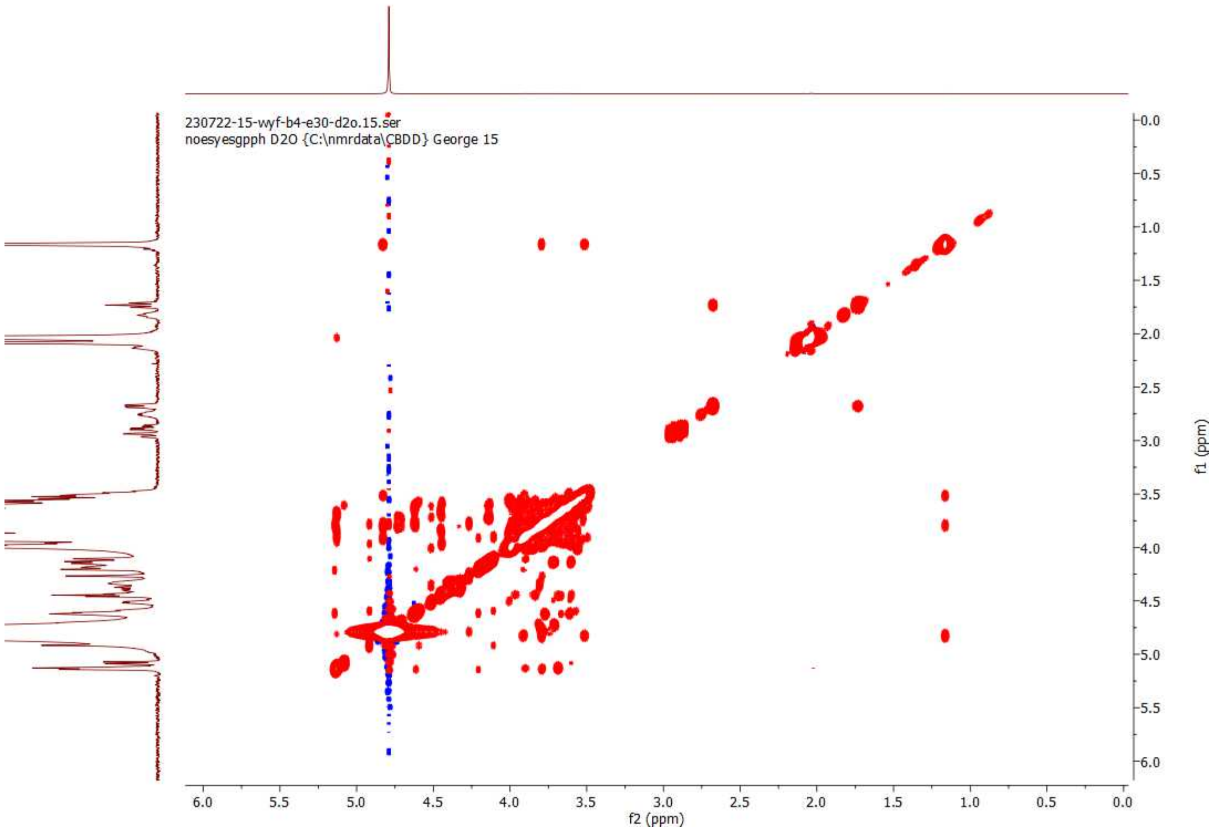

NOESY (300 ms) of 53; 600 MHz, D_2O

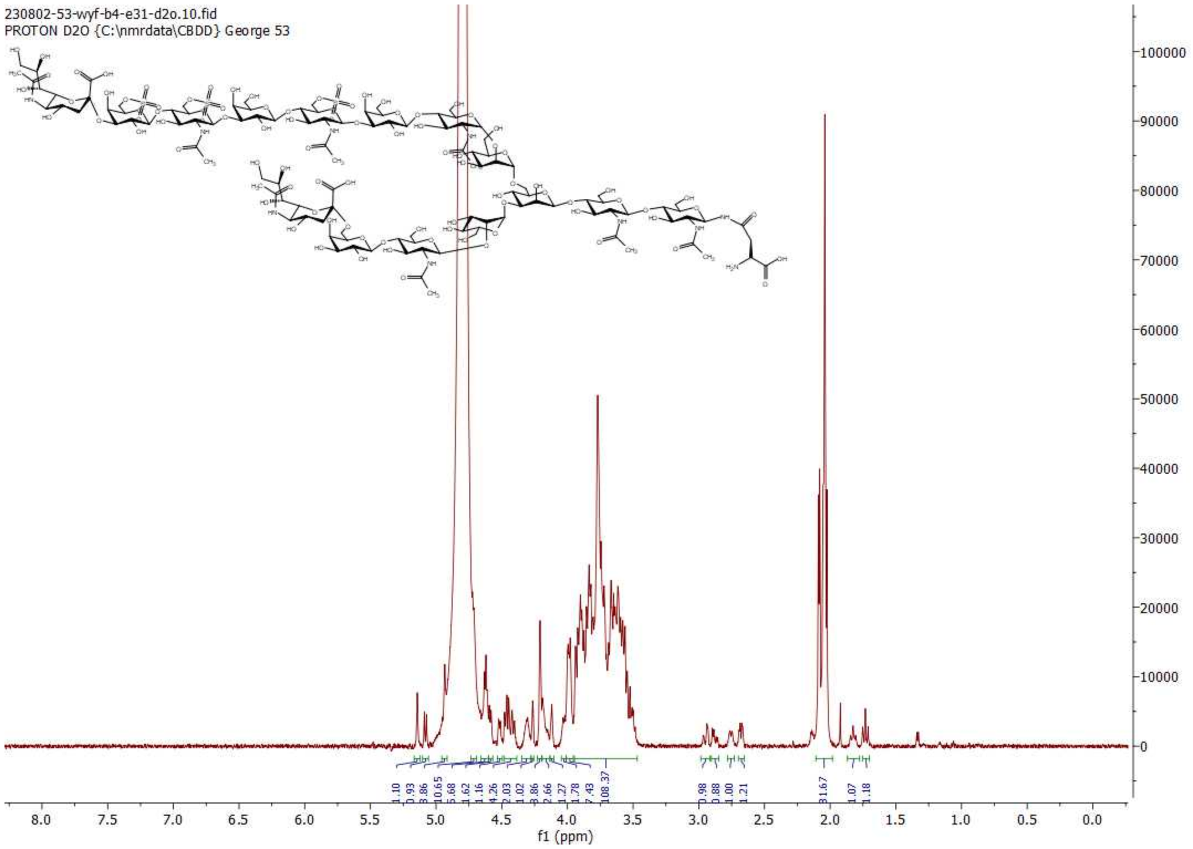

^{1}H NMR of 54; 600MHz; D_2O

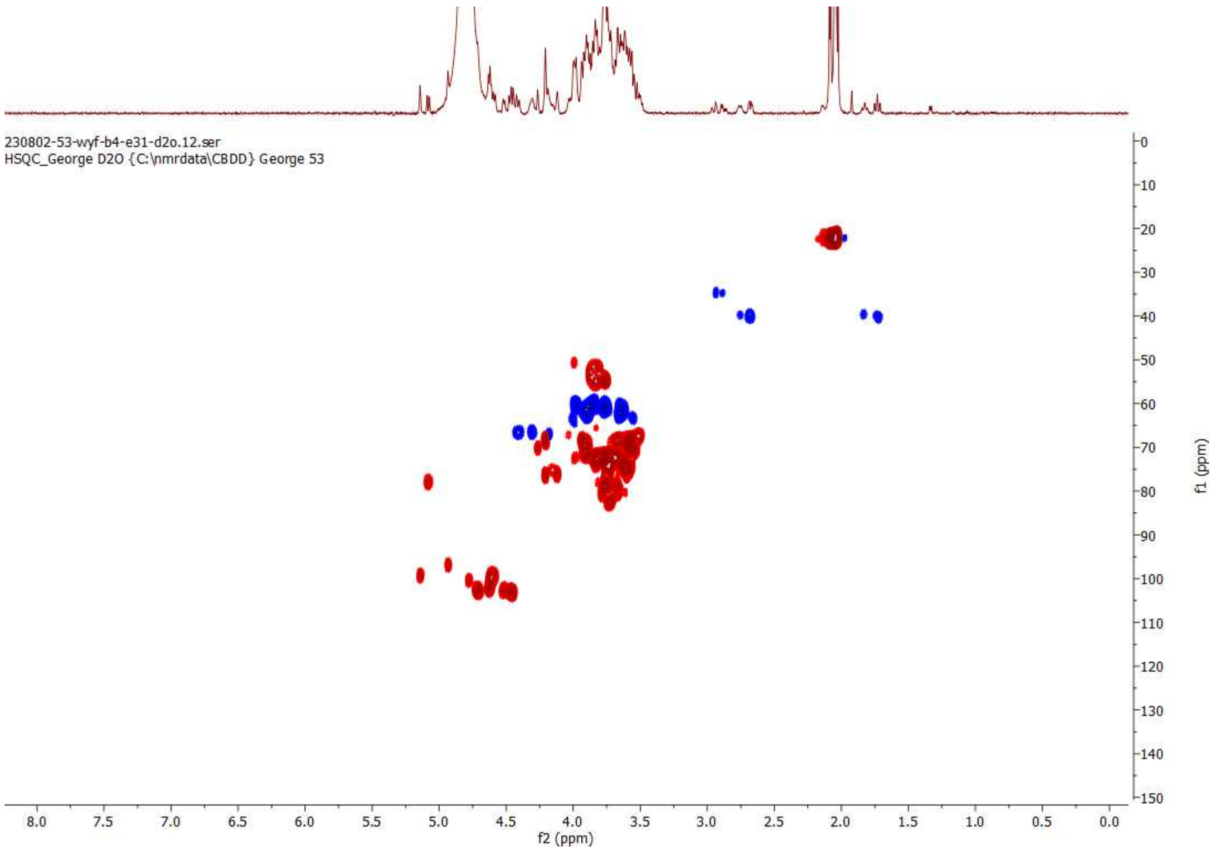

HSQC of 54; 600 MHz/150 MHz, D_2O

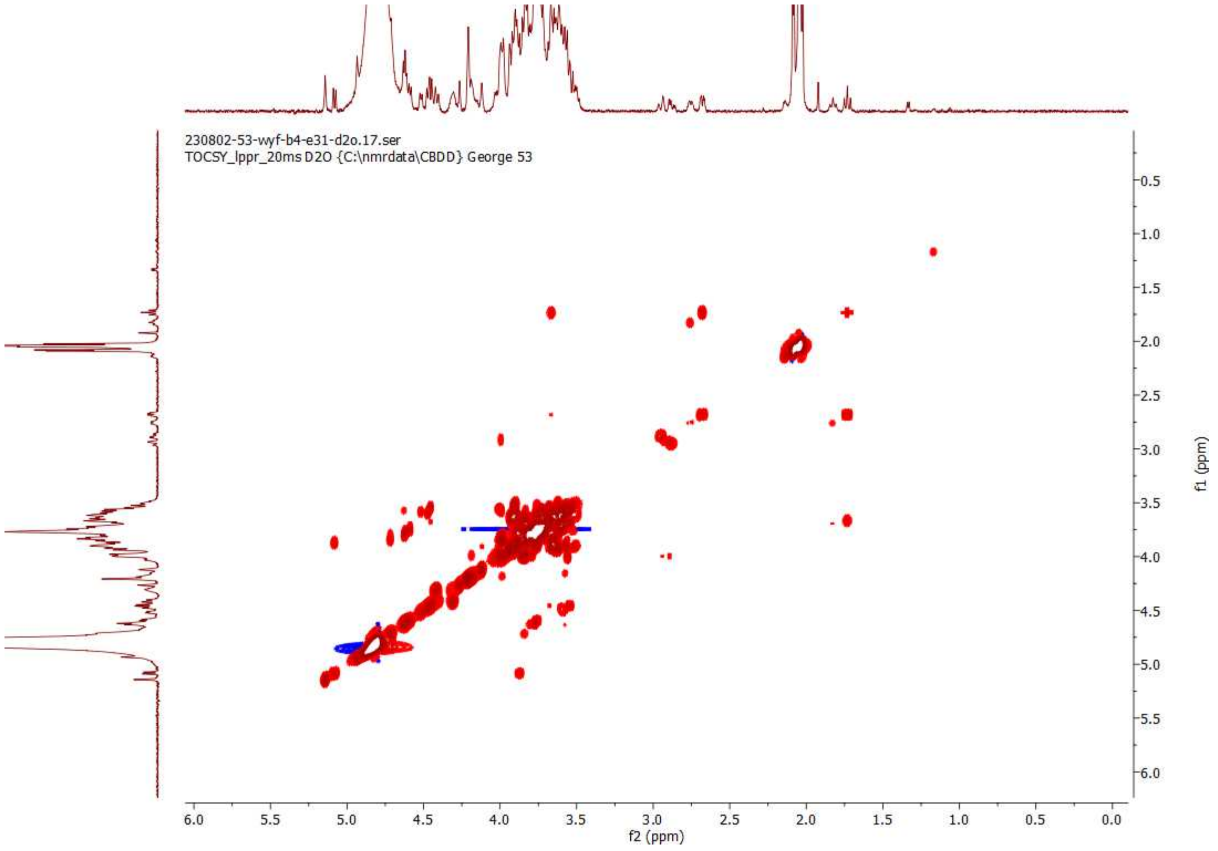

TOCSY (20 ms) of 54; 600MHz; D_2O

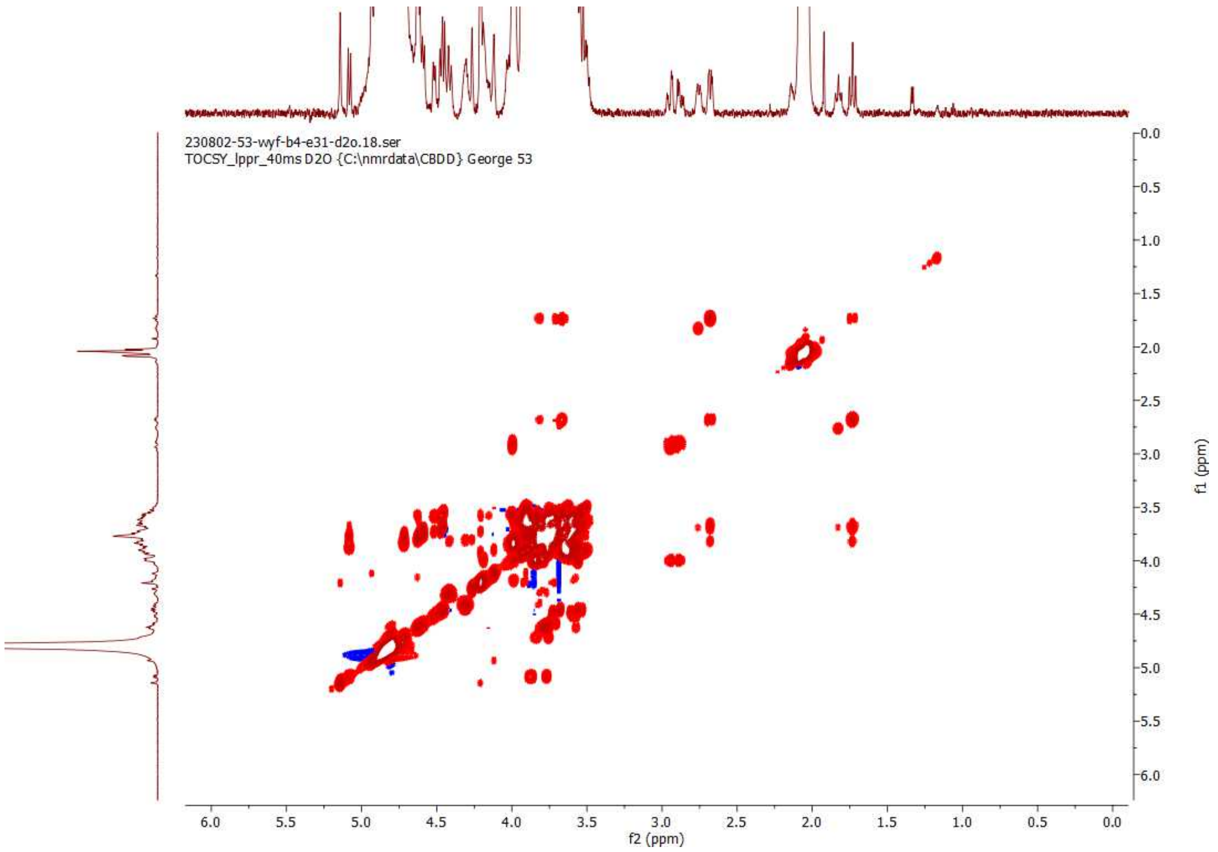

TOCSY (40 ms) of 54; 600MHz; D_2O

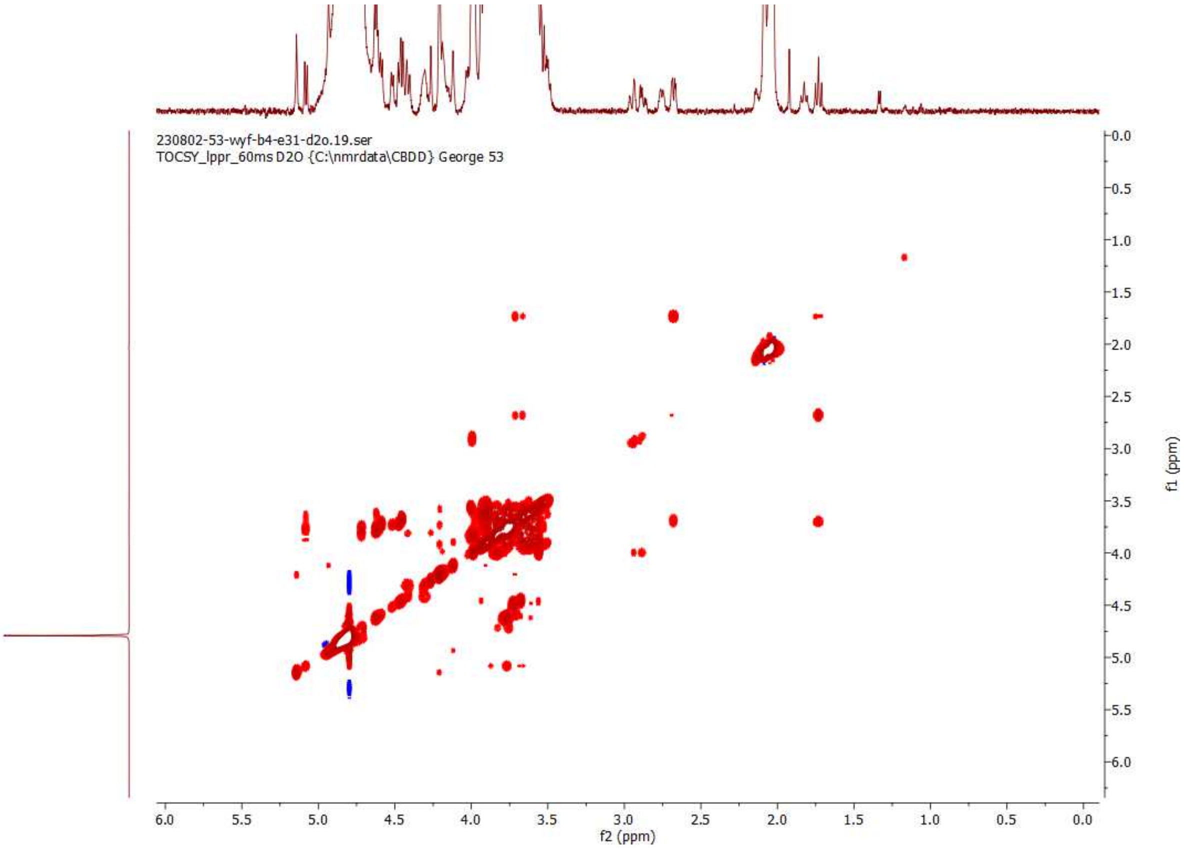

TOCSY (60 ms) of 54; 600MHz; D$_2$O

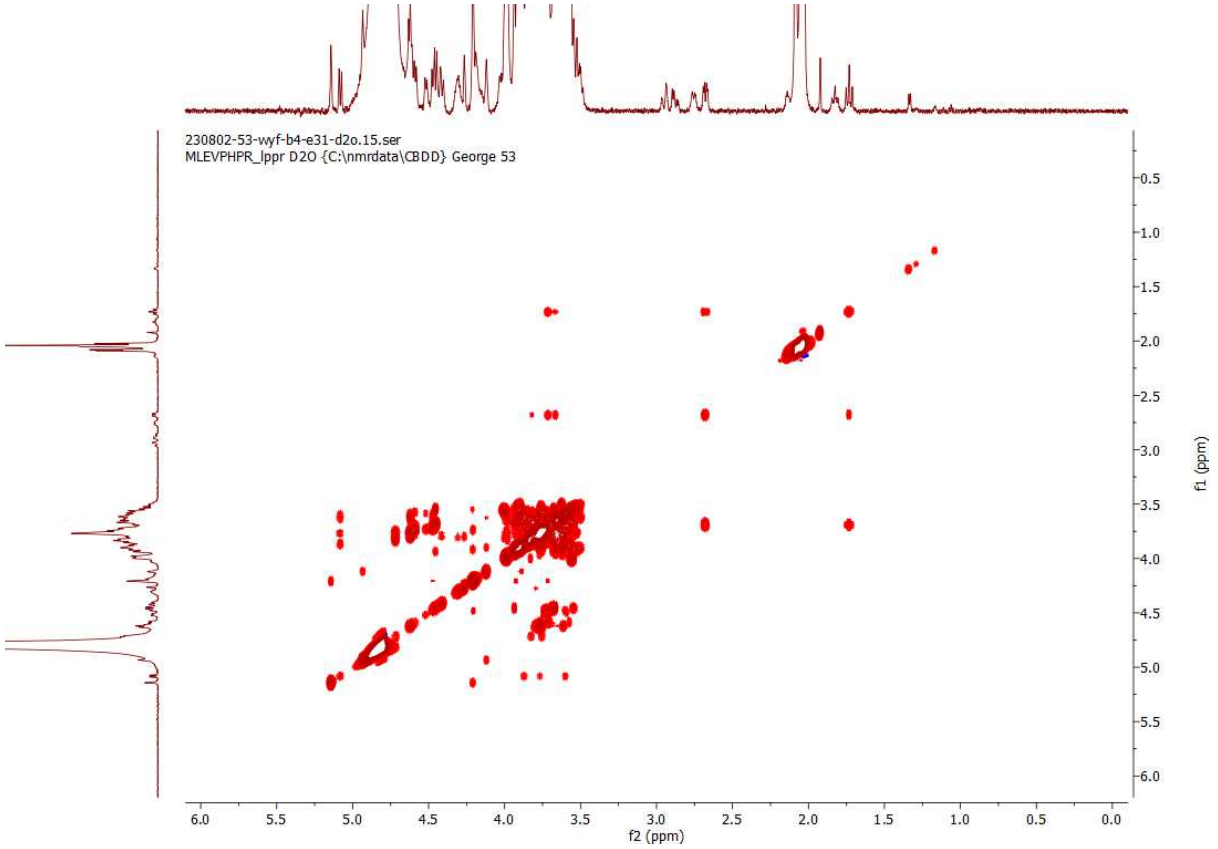

TOCSY (80 ms) of 54; 600MHz; D$_2$O

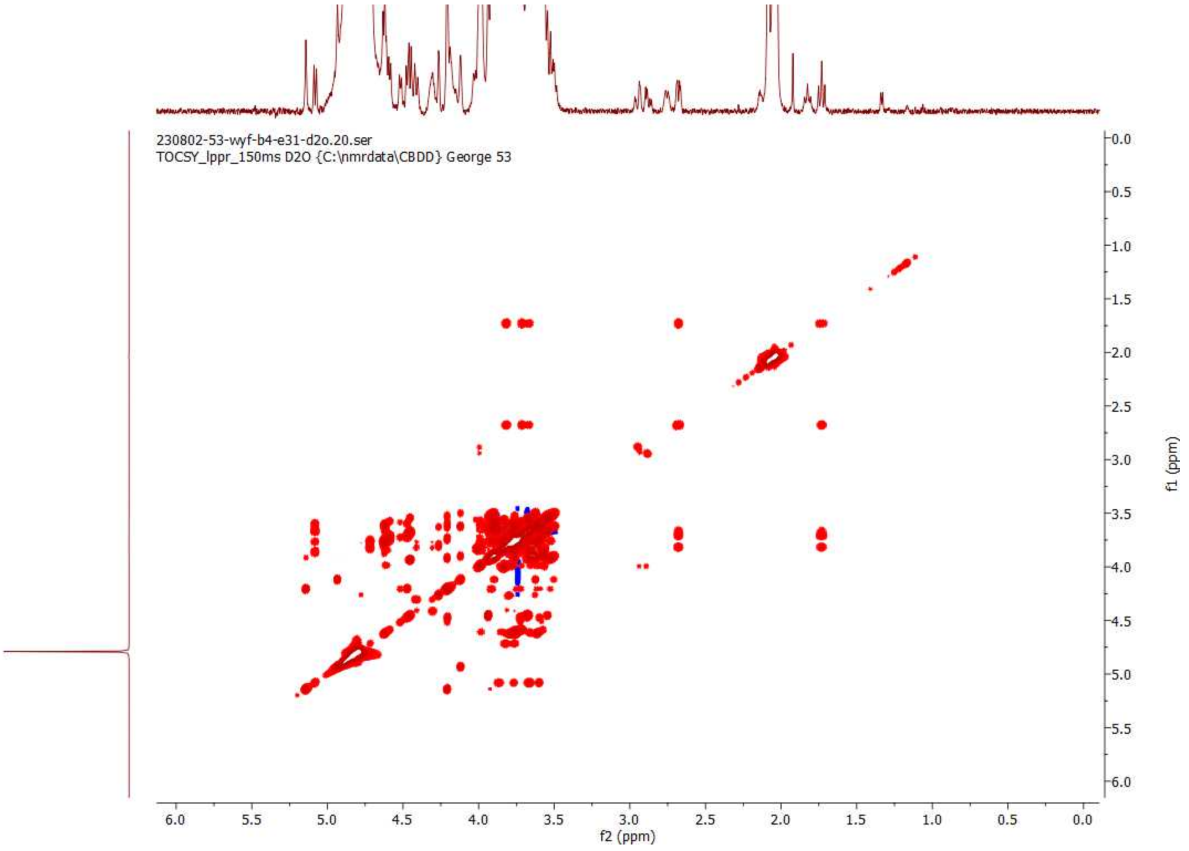

TOCSY (150 ms) of 54; 600MHz; D_2O

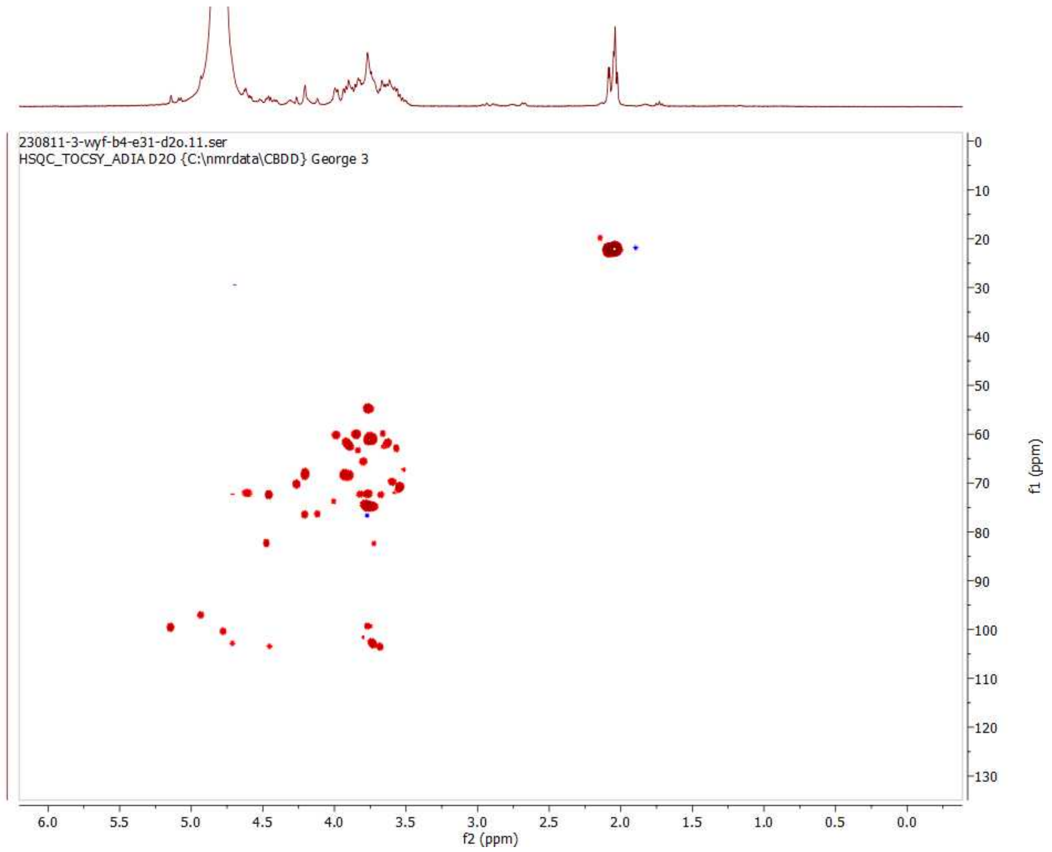

TOCSY-HSQC of 54; 600 MHz/150 MHz, D_2O

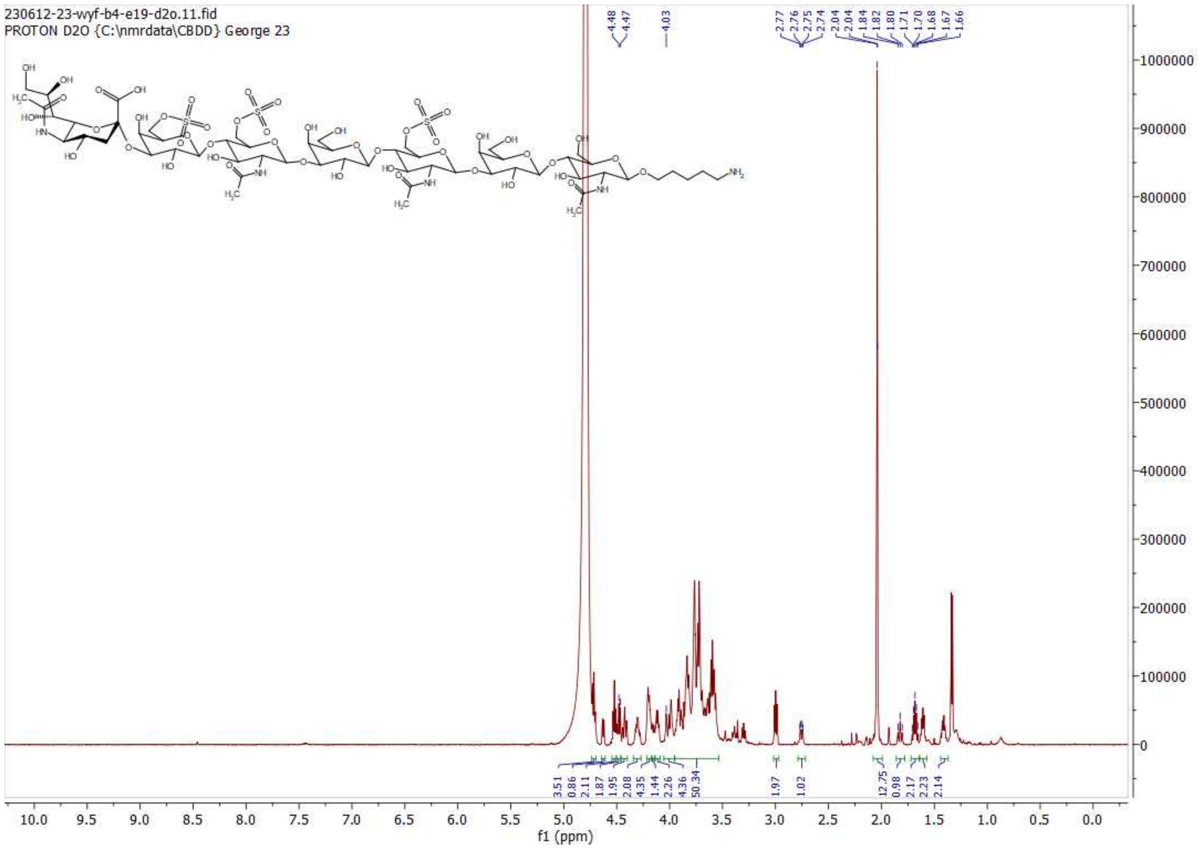

^{1}H NMR of S19; 600MHz; D₂O

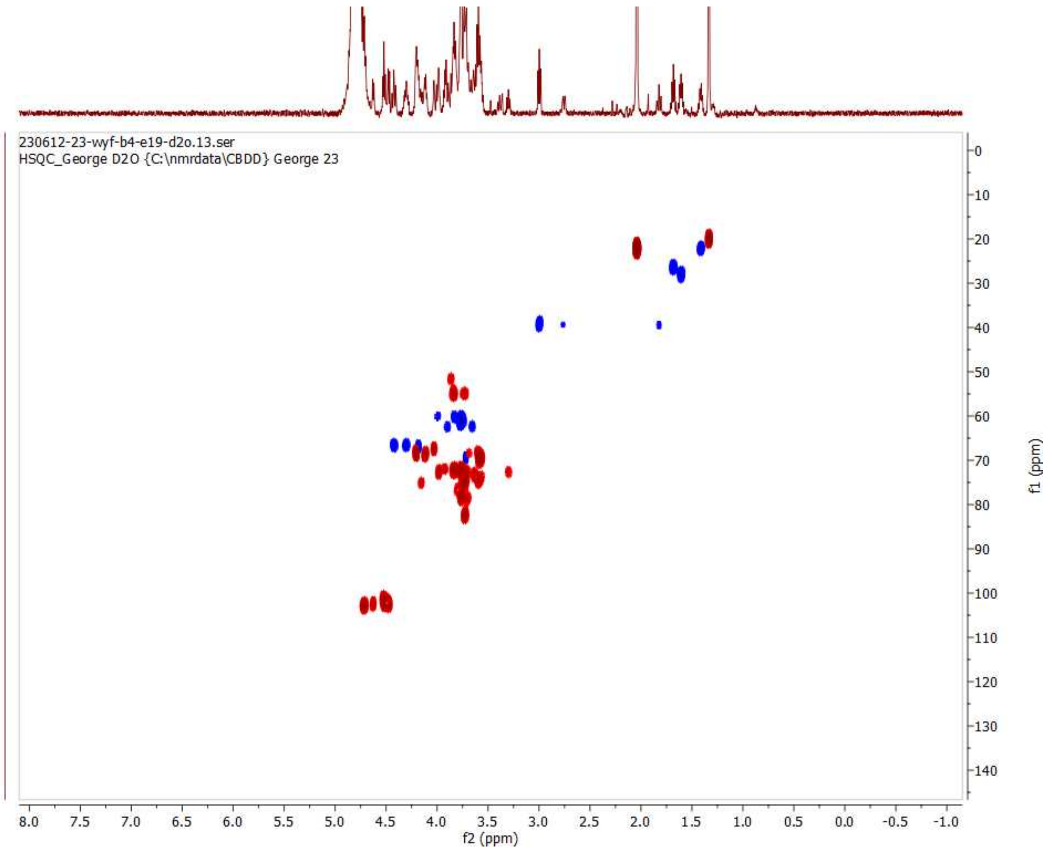

HSQC of S19; 600 MHz/150 MHz, D_2O

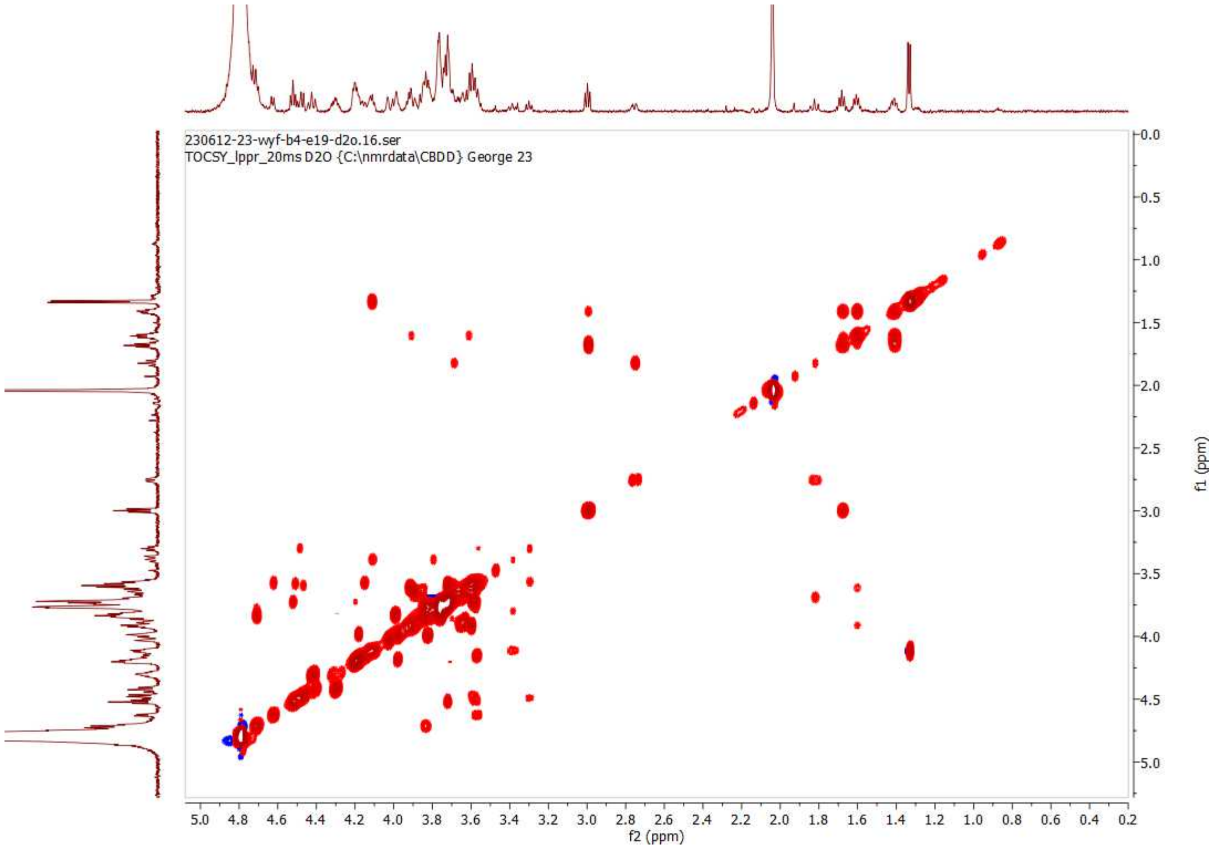

TOCSY (20 ms) of S19; 600MHz; D_2O

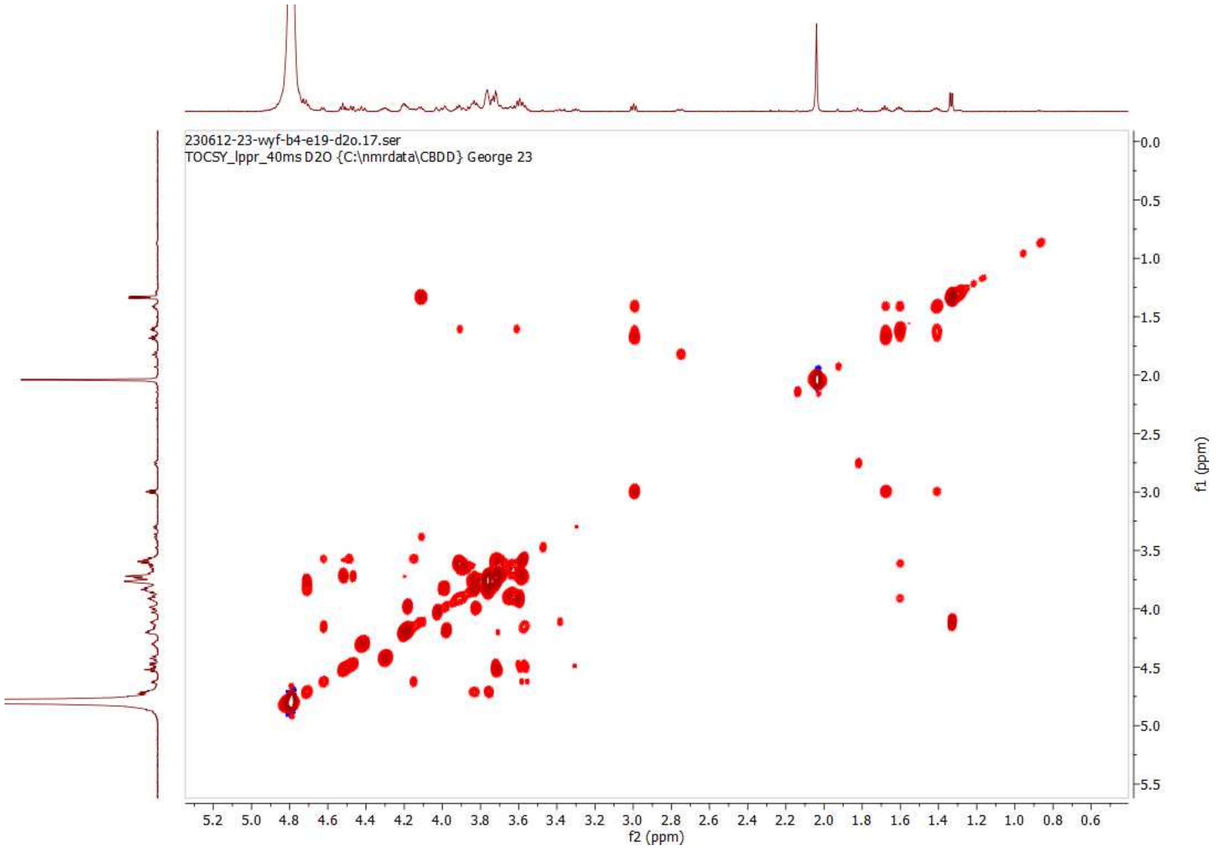

TOCSY (40 ms) of S19; 600MHz; D_2O

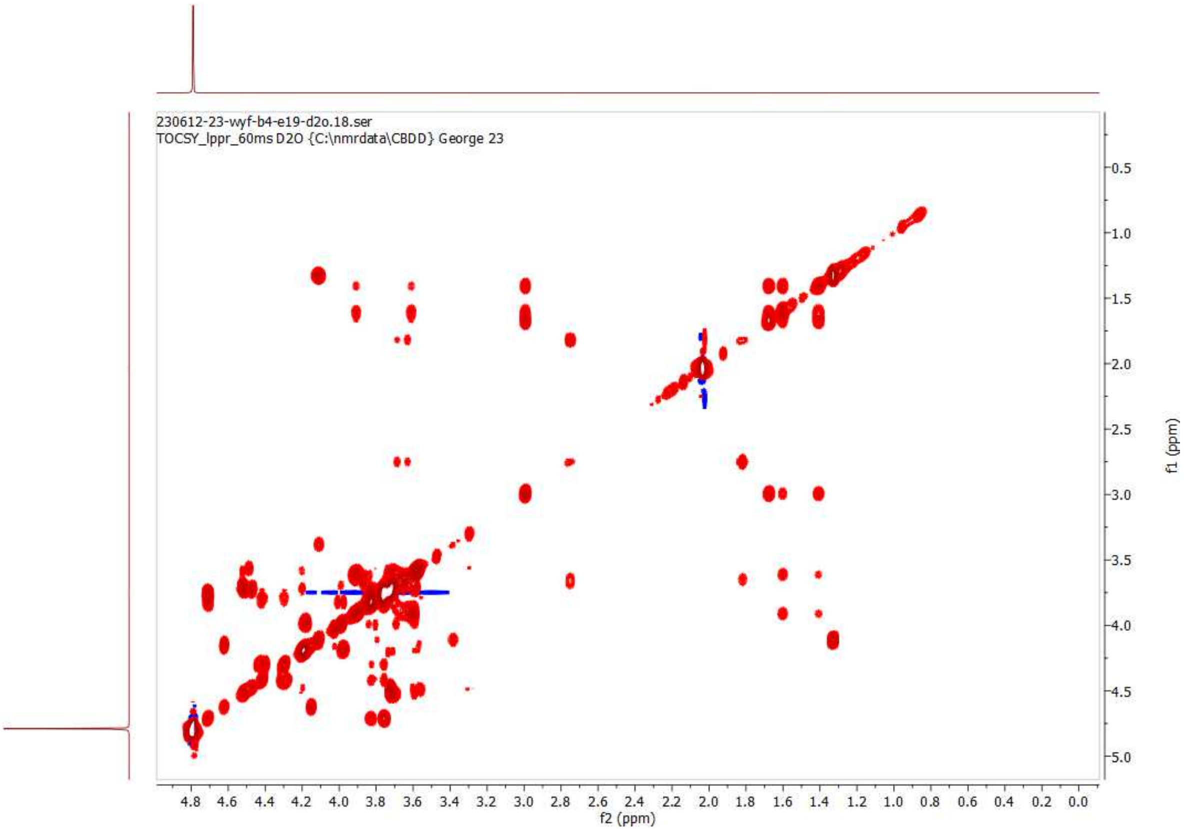

TOCSY (60 ms) of S19; 600MHz; D_2O

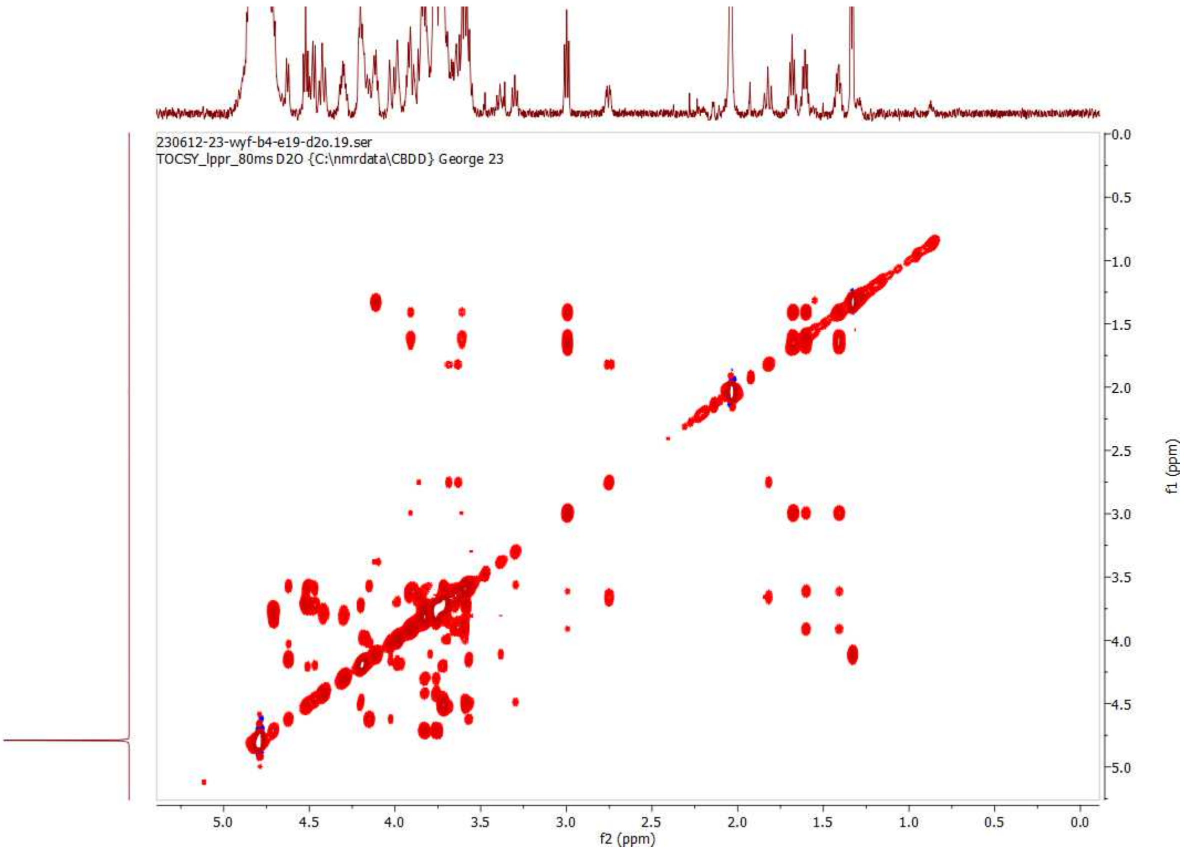

TOCSY (80 ms) of S19; 600MHz; D_2O

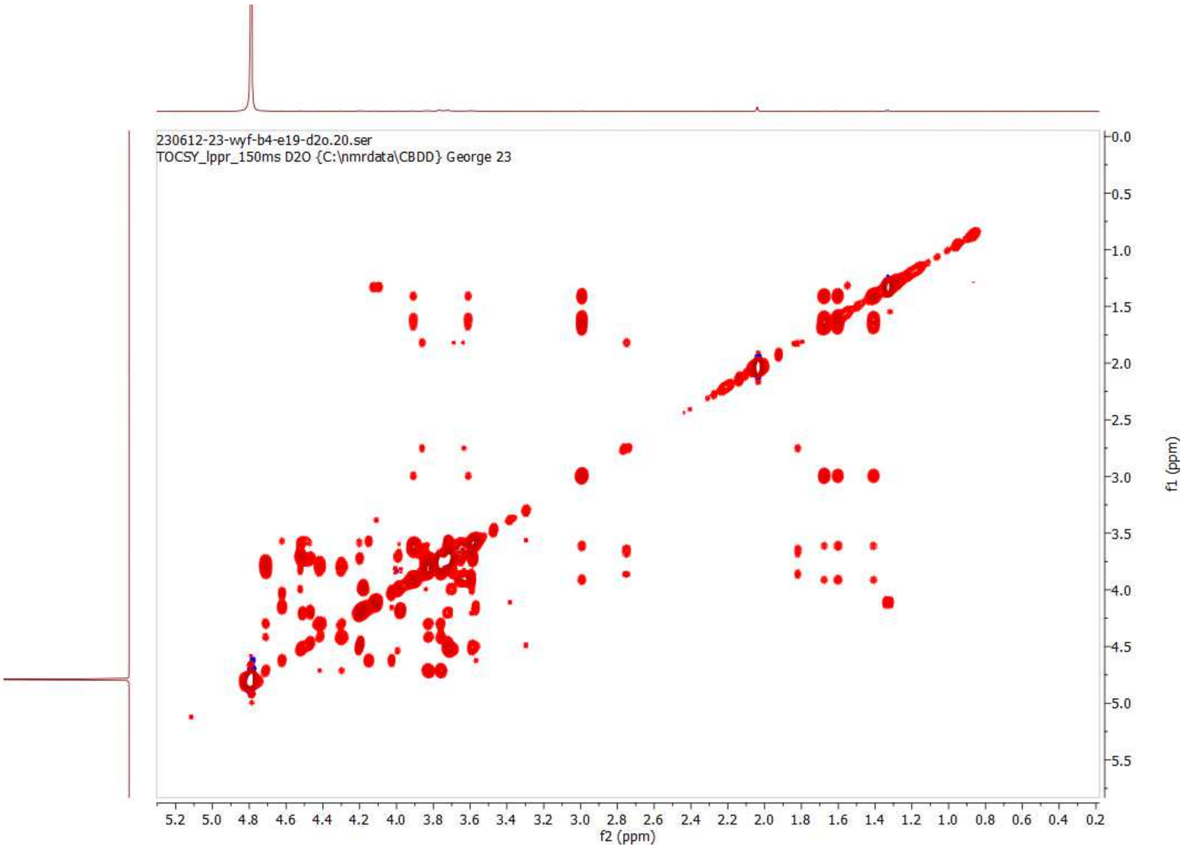

TOCSY (150 ms) of S19; 600MHz; D_2O

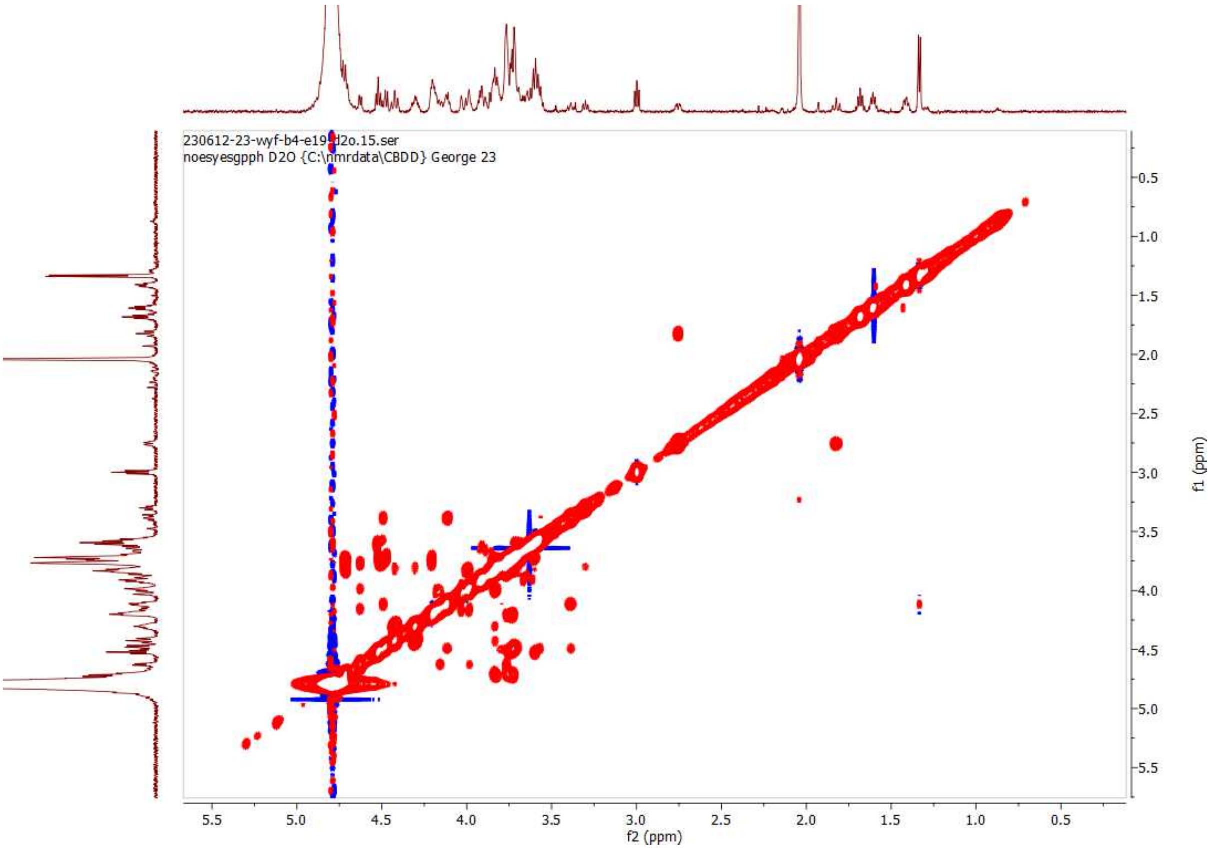

NOESY (300 ms) of S19; 600 MHz, D_2O

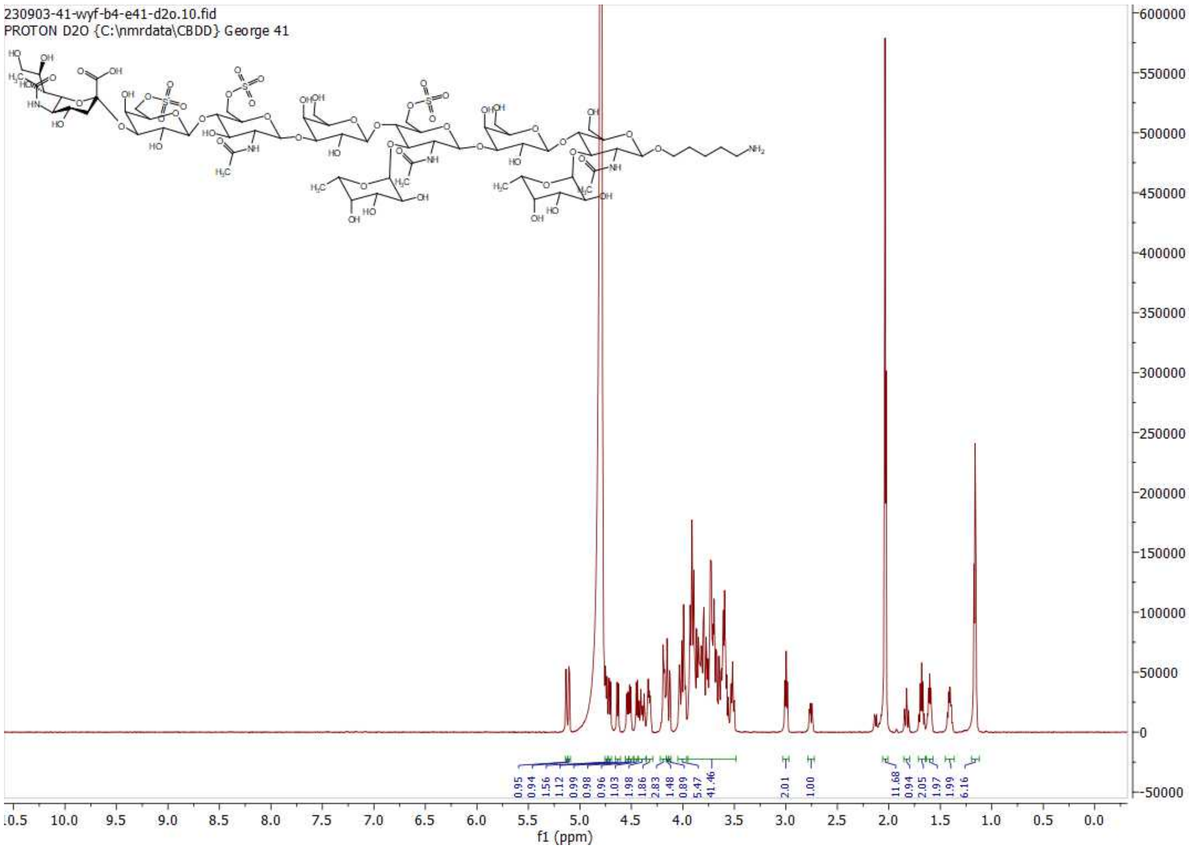

^{1}H NMR of S20; 600MHz; D_2O

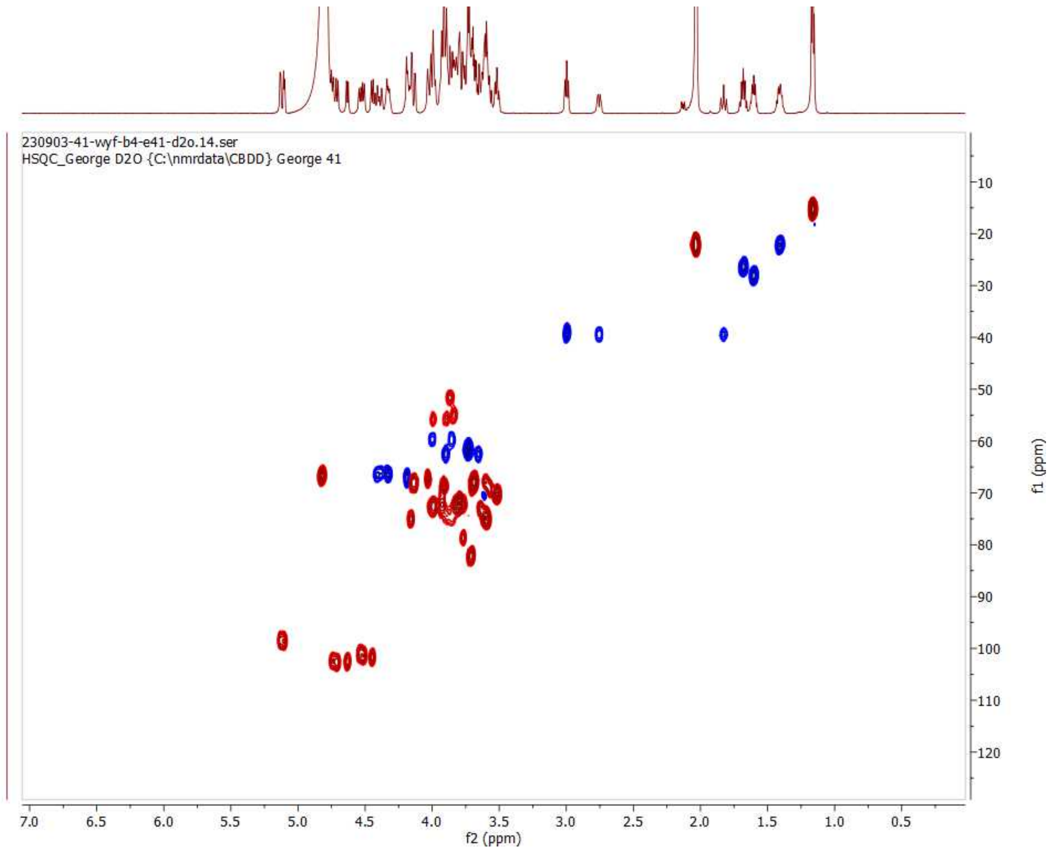

HSQC of S20; 600 MHz/150 MHz, D_2O

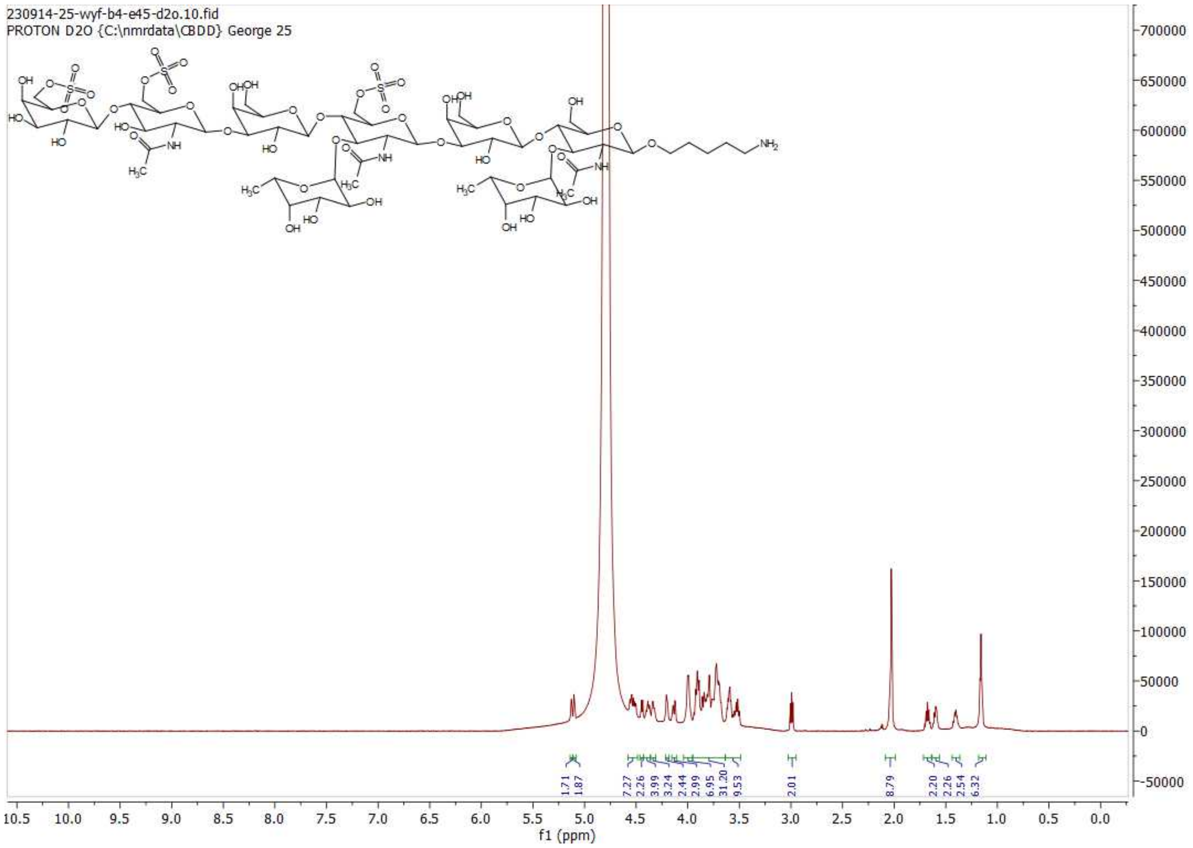

^{1}H NMR of S21; 600MHz; D_2O

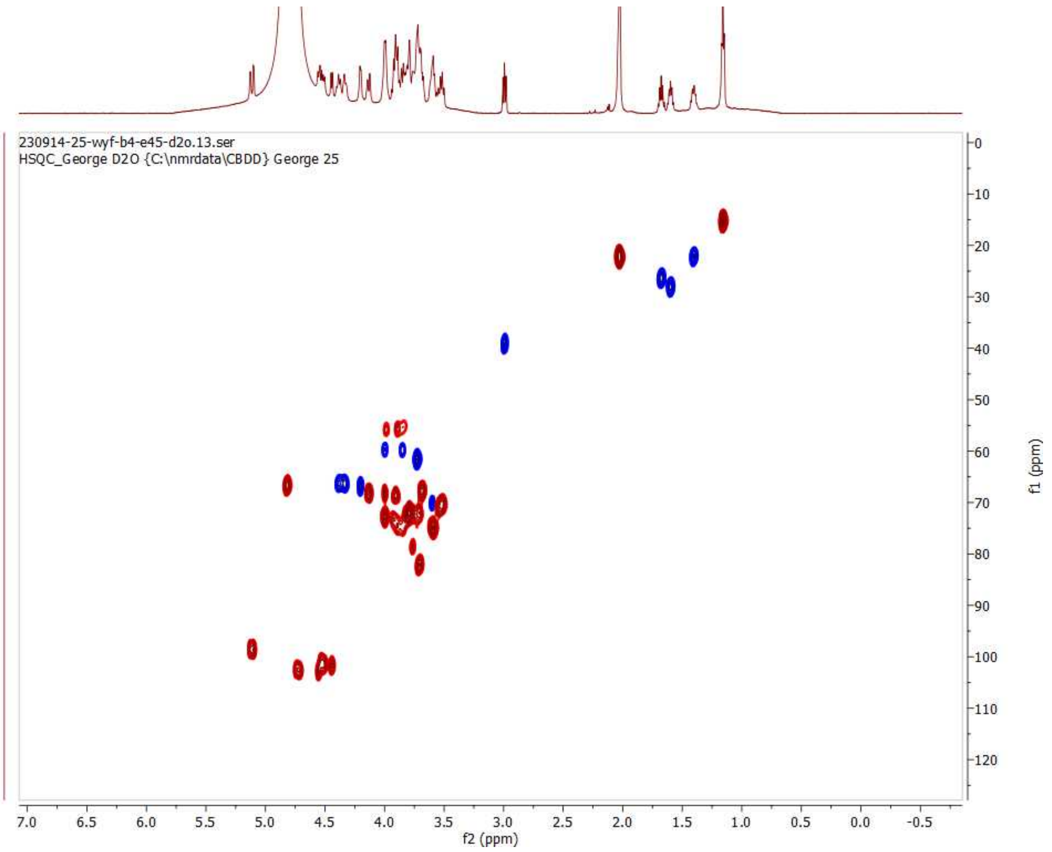

HSQC of S21; 600 MHz/150 MHz, D_2O

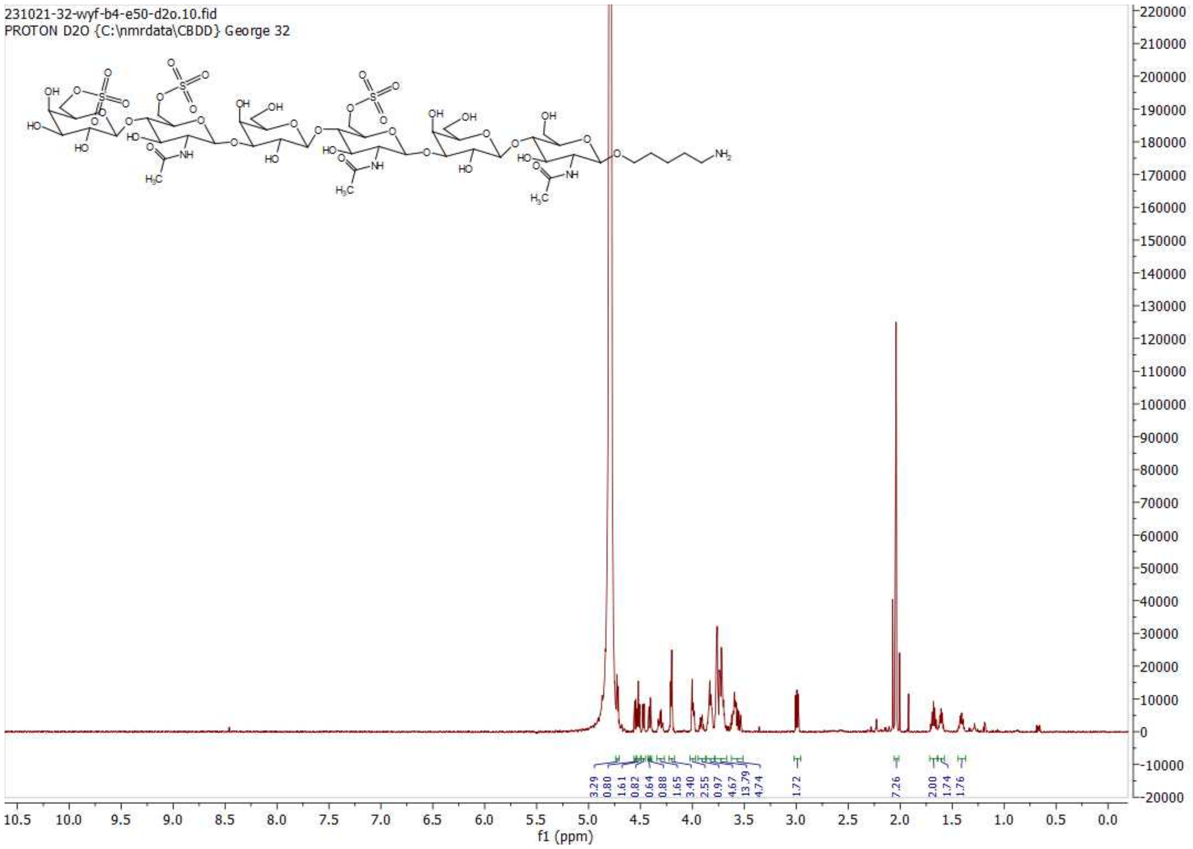

^{1}H NMR of S22; 600MHz; D$_2$O

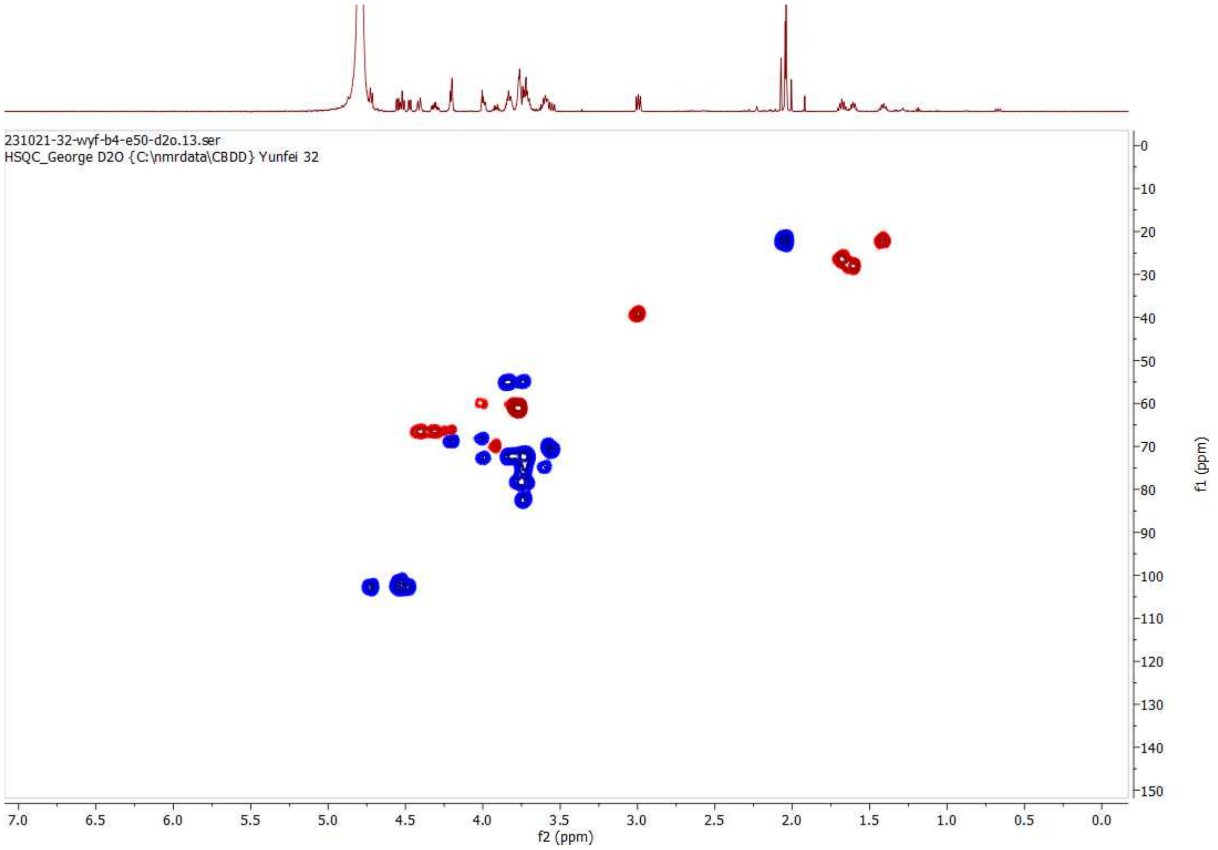

HSQC of S22; 600 MHz/150 MHz, D$_2$O

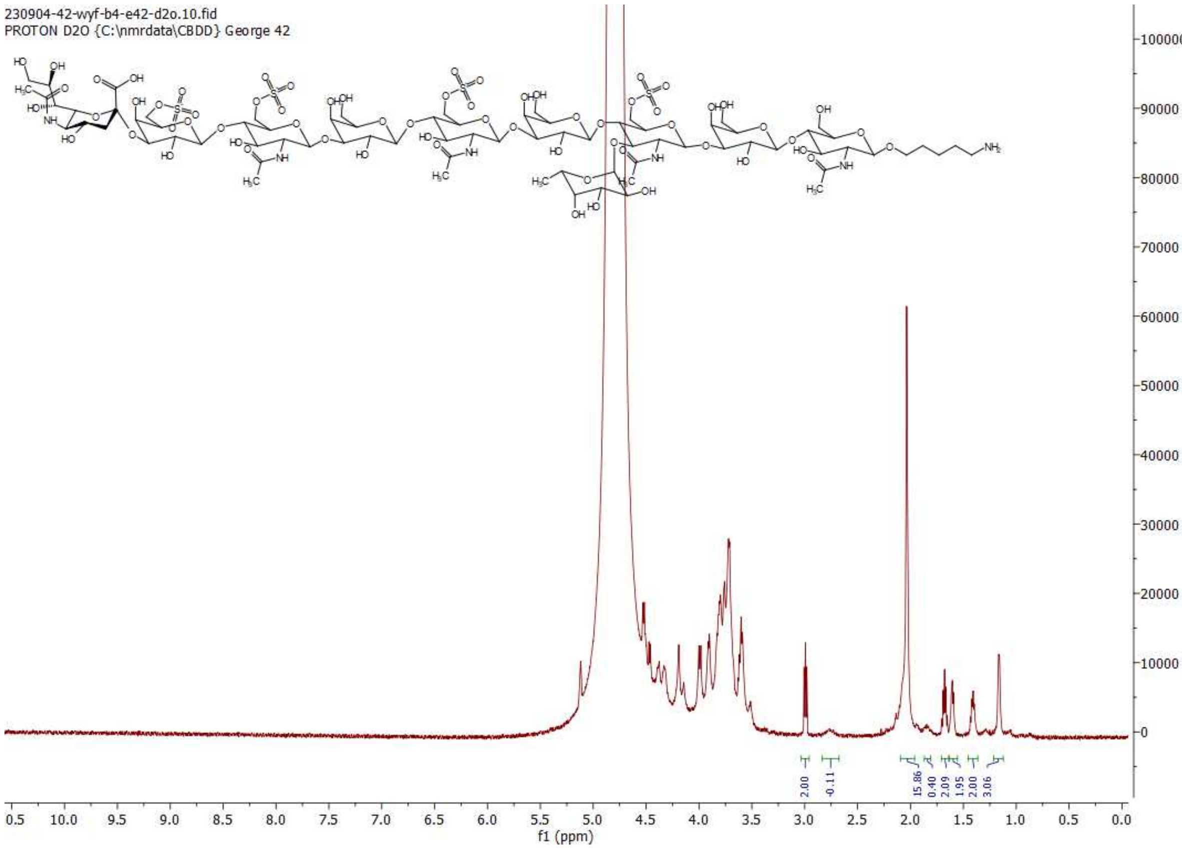

^{1}H NMR of S23; 600MHz; D_2O

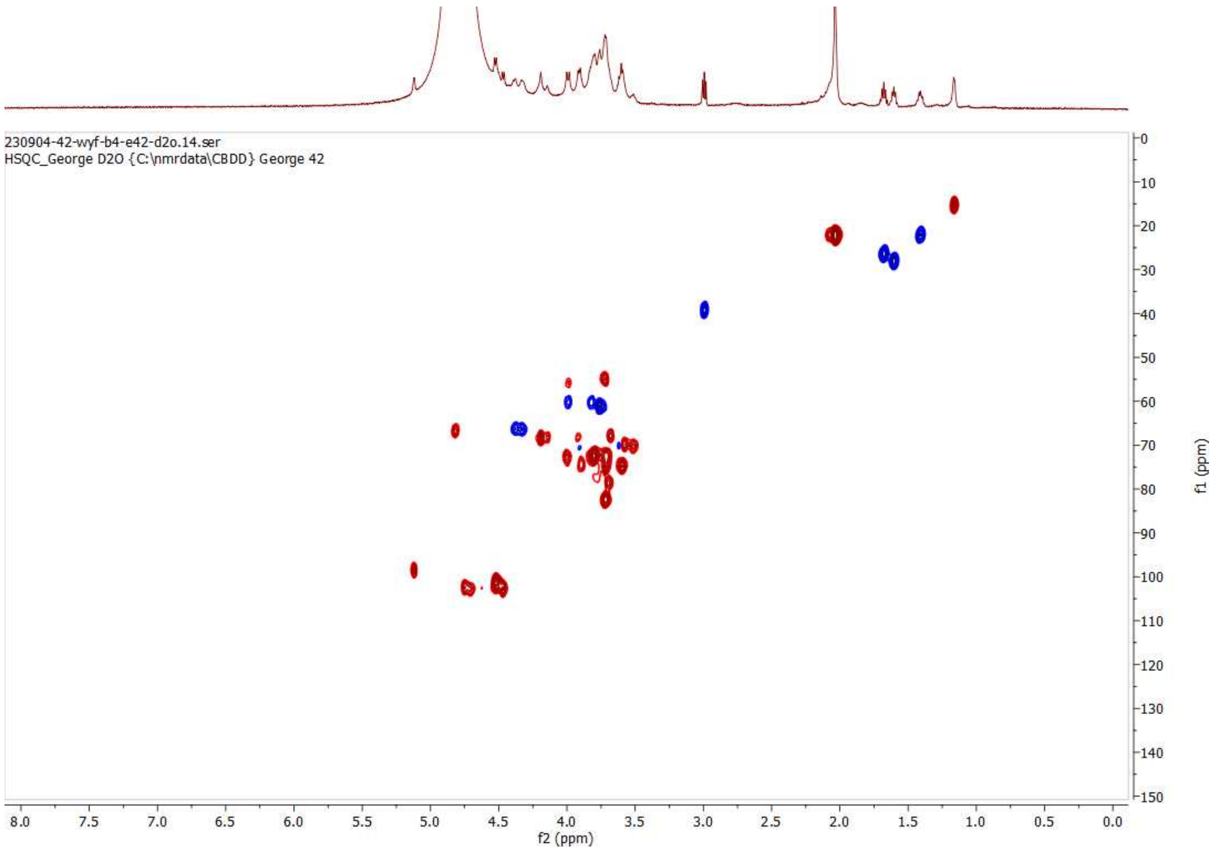

HSQC of S23; 600 MHz/150 MHz, D_2O

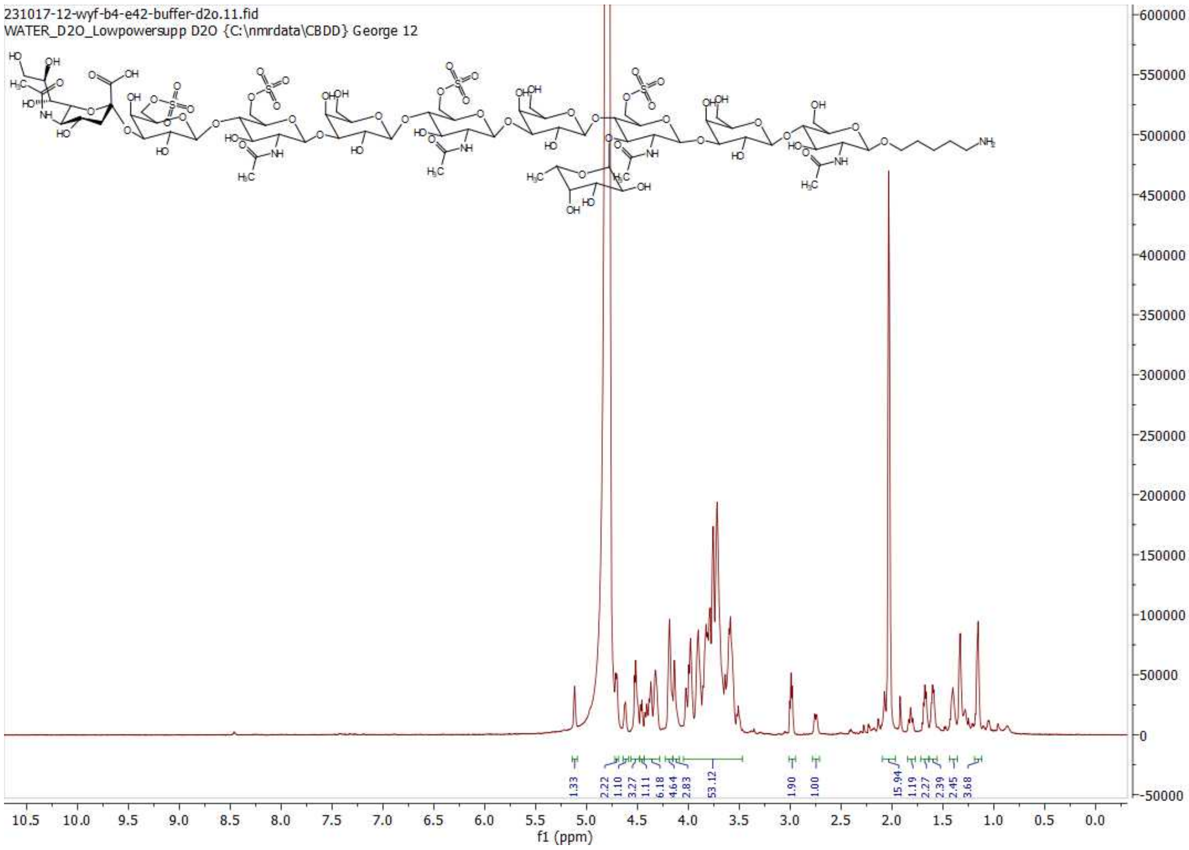

^{1}H NMR of S23; 600MHz; PBS D_2O buffer (40 mM, pH=6.5)

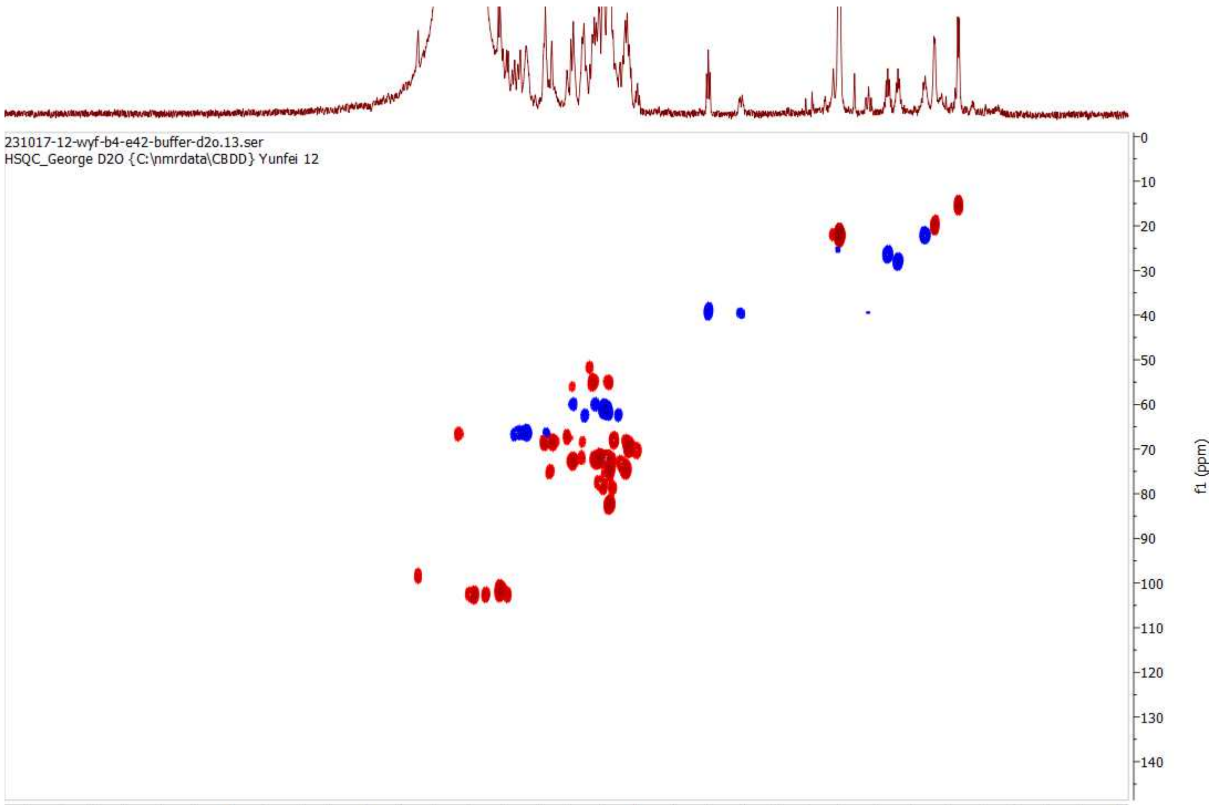

HSQC of S23; 600 MHz/150 MHz, PBS D_2O buffer (40 mM, pH=6.5)

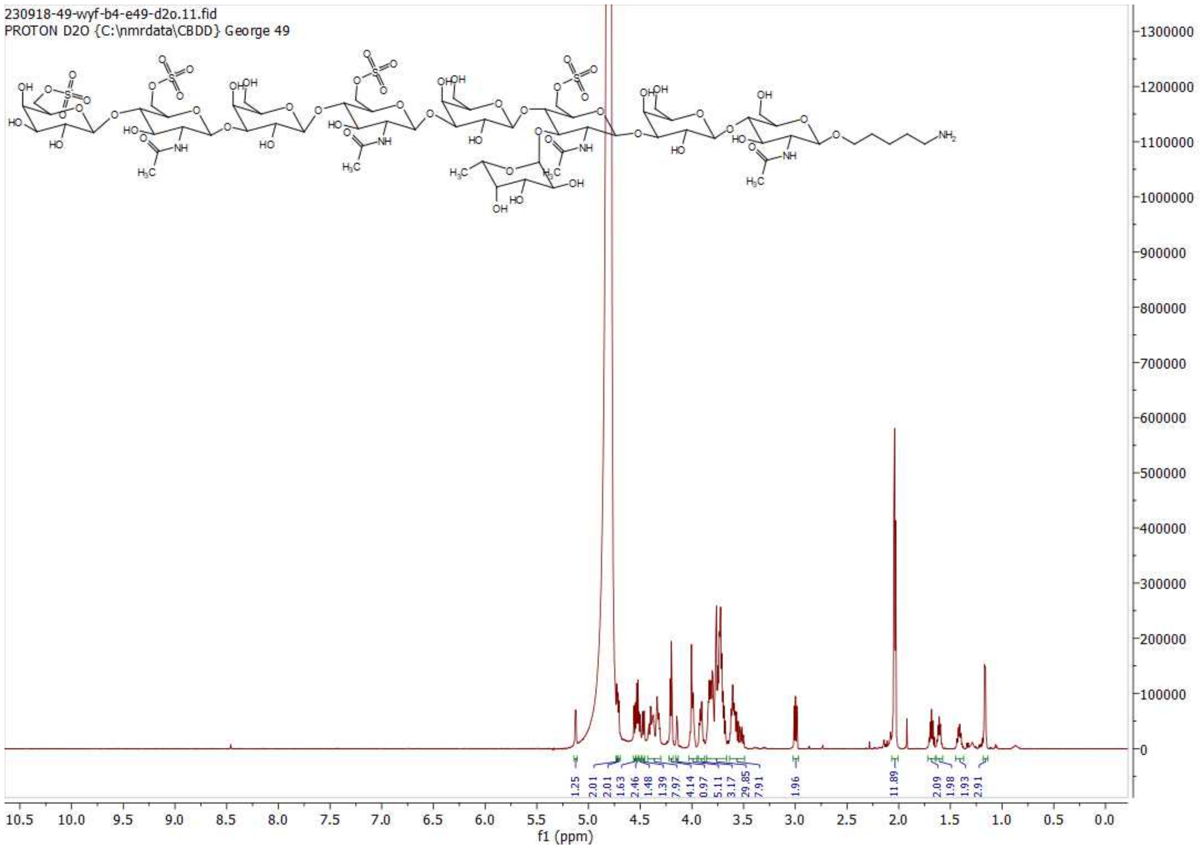

^{1}H NMR of S24; 600MHz; D_2O

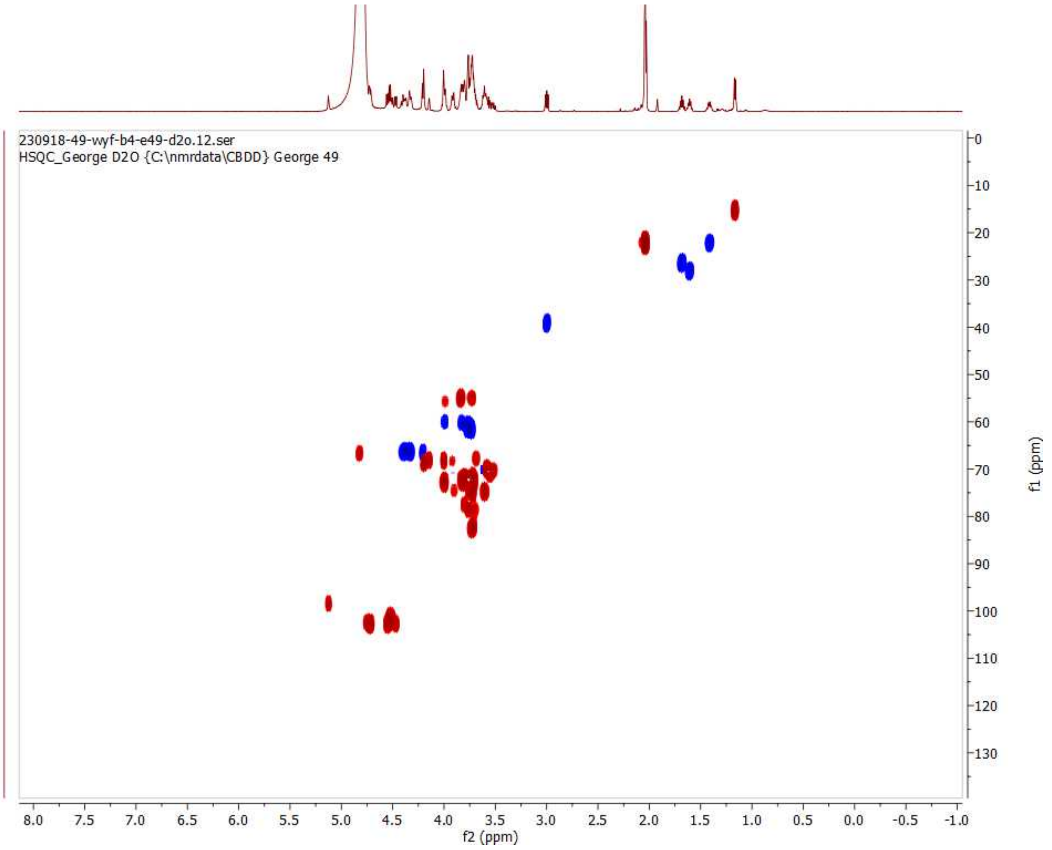

HSQC of S24; 600 MHz/150 MHz, D_2O

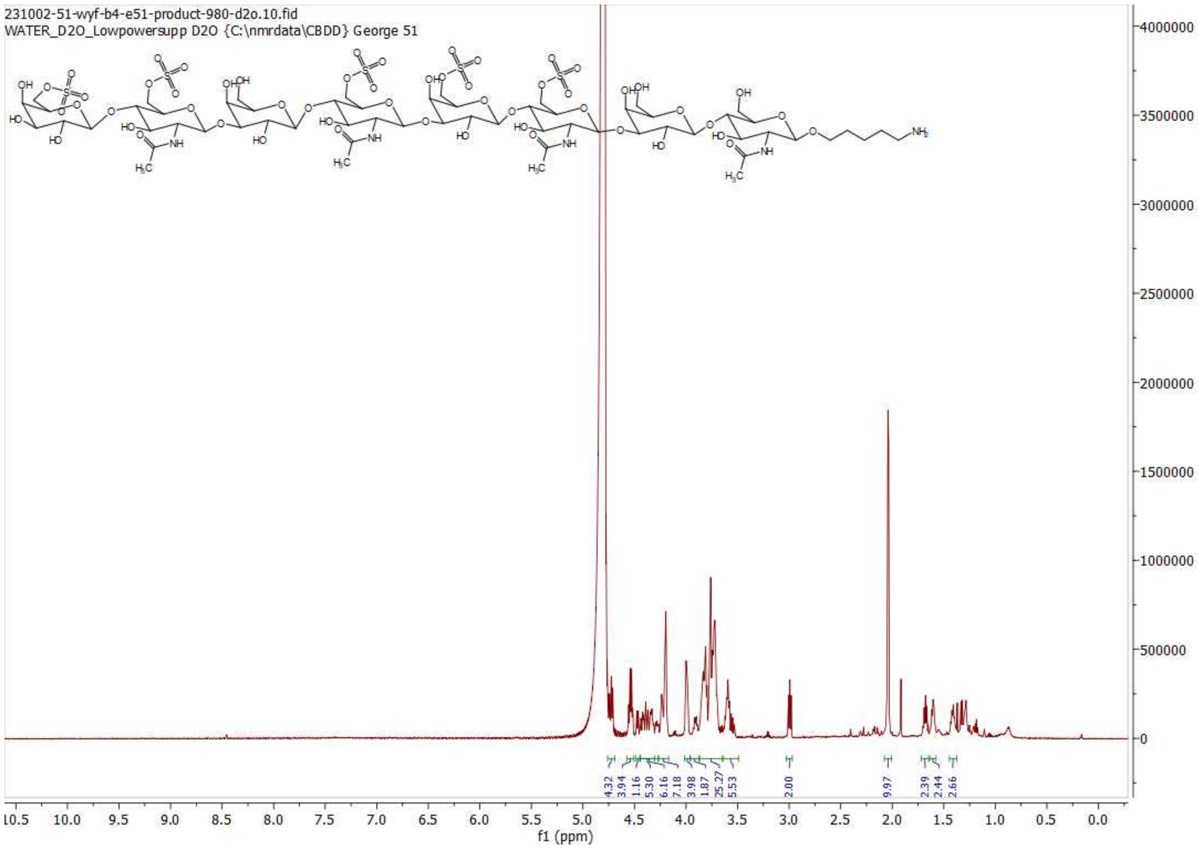

^{1}H NMR of S25; 600MHz; D_2O

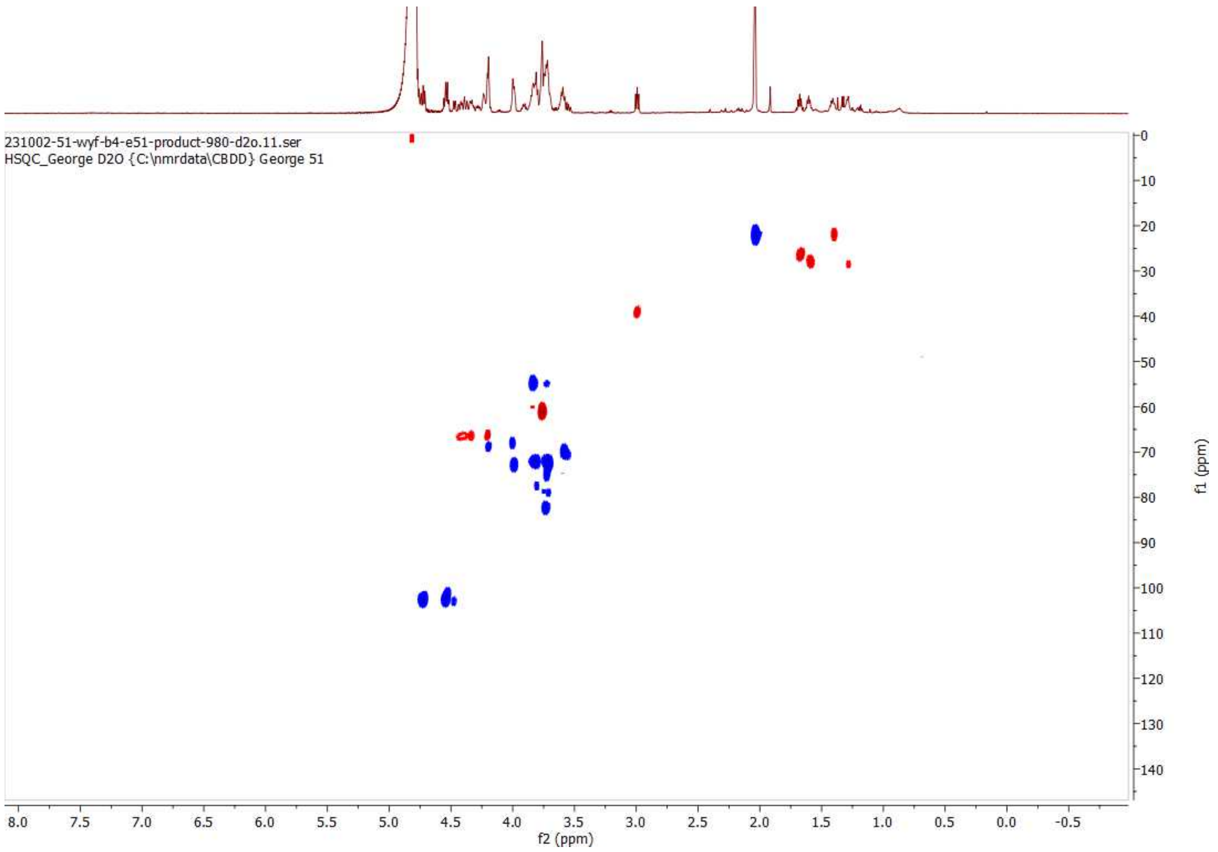

HSQC of S25; 600 MHz/150 MHz, D_2O

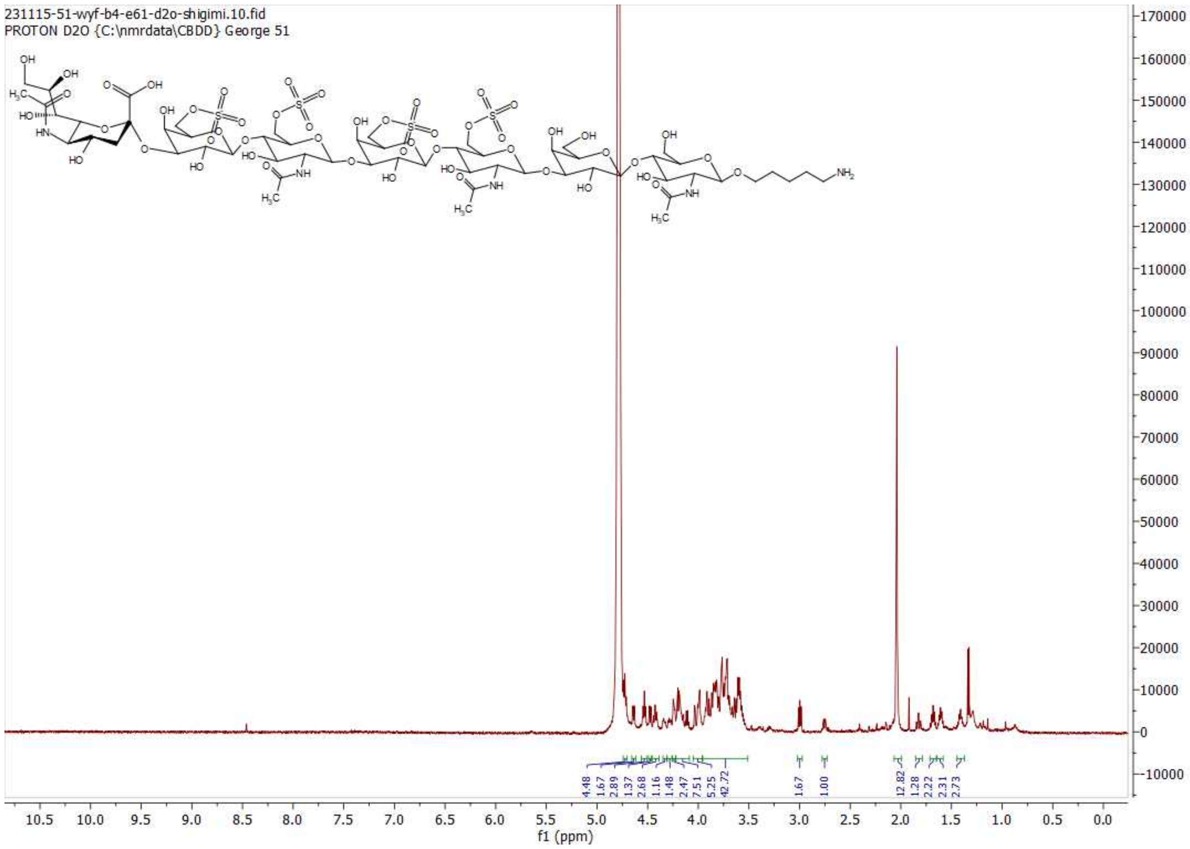

^{1}H NMR of S26; 600MHz; D$_2$O

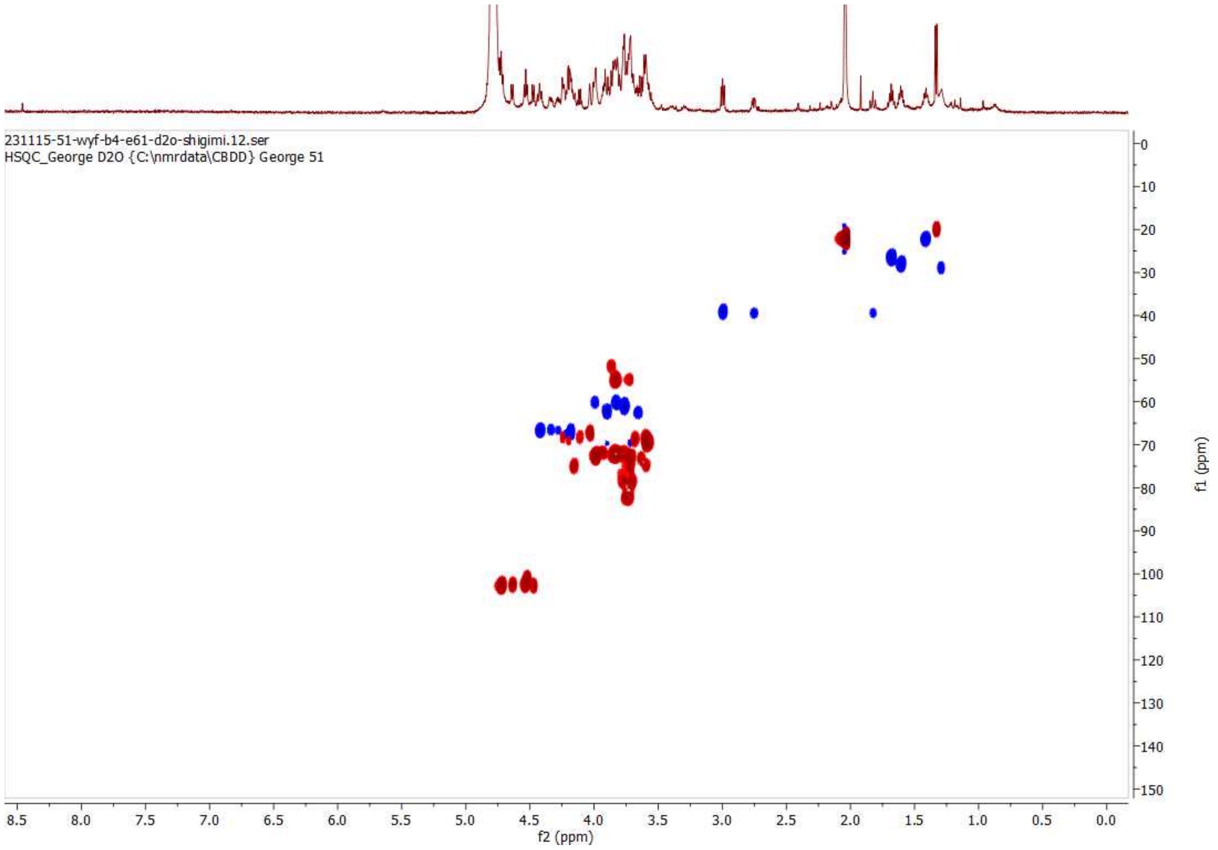

HSQC of S26; 600 MHz/150 MHz, D$_2$O
